# Red versus green leaves: transcriptomic comparison of foliar senescence between two *Prunus cerasifera* genotypes

**DOI:** 10.1038/s41598-020-58878-8

**Published:** 2020-02-06

**Authors:** Alberto Vangelisti, Lucia Guidi, Andrea Cavallini, Lucia Natali, Ermes Lo Piccolo, Marco Landi, Giacomo Lorenzini, Fernando Malorgio, Rossano Massai, Cristina Nali, Elisa Pellegrini, Giovanni Rallo, Damiano Remorini, Paolo Vernieri, Tommaso Giordani

**Affiliations:** 1 Department of Agriculture, Food and Environment, University of Pisa, Via del Borghetto 80, 56124 Pisa, Italy; 2 CIRSEC, Centre for Climate Change Impact, University of Pisa, Via del Borghetto 80, 56124 Pisa, Italy

**Keywords:** Plant cell biology, Plant genetics, Plant sciences, Plant physiology

## Abstract

The final stage of leaf ontogenesis is represented by senescence, a highly regulated process driven by a sequential cellular breakdown involving, as the first step, chloroplast dismantling with consequent reduction of photosynthetic efficiency. Different processes, such as pigment accumulation, could protect the vulnerable photosynthetic apparatus of senescent leaves. Although several studies have produced transcriptomic data on foliar senescence, just few works have attempted to explain differences in red and green leaves throughout ontogenesis. In this work, a transcriptomic approach was used on green and red leaves of *Prunus cerasifera* to unveil molecular differences from leaf maturity to senescence. Our analysis revealed a higher gene regulation in red leaves compared to green ones, during leaf transition. Most of the observed DEGs were shared and involved in transcription factor activities, senescing processes and cell wall remodelling. Significant differences were detected in cellular functions: genes related to photosystem I and II were highly down-regulated in the green genotype, whereas transcripts involved in flavonoid biosynthesis, such as *UDP glucose-flavonoid-3-O-glucosyltransferase* (UFGT) were exclusively up-regulated in red leaves. In addition, cellular functions involved in stress response (*glutathione-S-transferase*, *Pathogen-Related*) and sugar metabolism, such as three *threalose-6-phosphate synthases*, were activated in senescent red leaves. In conclusion, data suggests that *P. cerasifera* red genotypes can regulate a set of genes and molecular mechanisms that cope with senescence, promoting more advantages during leaf ontogenesis than compared to the green ones.

## Introduction

A fully developed and functional photosynthetic apparatus is the most important prerequisite for plant life, ensuring CO_2_ assimilation and synthesis of molecules involved in the growth of the whole plant. Senescence represents the final stage of leaf development by a highly regulated process of ontogenesis preceding cell death and during which the sequential breakdown of cellular components occurs. Leaf senescence is a biological transition, which requires complex biological processes such as a range of degradative, biosynthetic and regulatory mechanisms coordinated by changes in gene expression aimed to remobilise and reutilise molecules until the progress of senescence culminates in tissue death^1–5^.

Senescence is accompanied by visual changes in leaf pigmentation^6^, with a preferential degradation of chlorophylls compared to carotenoids and, in some plant species, by the synthesis of red-coloured pigments like anthocyanins^4,7,8^. Strong decreases in chlorophyll content and disassembling and degradation of the photosynthetic apparatus (chlorophyll and chloroplast protein degradation) is due to a regulated activity of specific enzymes and proteases that ultimately induces a decrease in photosynthetic CO_2_ assimilation^2,6,9,10^. Consequently, senescent leaves are potentially exposed to an excess of excitation energy due to a reduction in the utilisation of NADPH and ATP. This event induces an increase in the production of reactive oxygen species (ROS)^11,12^ that are not counterbalanced by the action of antioxidant systems^13^. On the other hand, ROS accumulation induces an oxidative burst (i.e. oxidation of pigments, proteins and lipids), a process necessary for photosynthetic protein degradation and nutrient remobilisation^14^. Levels of ROS are controlled until the end of the remobilisation and the late phase of senescence that occurs with cell death^15,16^. For this reason, during chloroplast senescence, dismantling has to be finely regulated and photoprotection of the photosynthetic apparatus represents a key feature in the control of the progression of leaf senescence^16^. Concerning the regulation of metabolic network during ontogenesis, overall sugar content increases, while amino acid concentration declines in senescing leaves^1,17^. Interestingly, sugars have also been recognised as regulatory molecules involved in signalling function, translation and protein stability^17^. Sugars, especially hexose, are able to activate several cellular functions and transcription factors involved in typical senescence-associated genes such as *SAG12*^18^; Over-expression of genes involved in sugar translocation, such as those encoding invertases, can also delay senescing processes^19^. In addition, a finely tuned up-regulation of anthocyanin biosynthesis promoted by sugar accumulation has been clearly demonstrated^20^.

Many plant species show genotypes with different constitutive leaf pigmentation, e.g. *Prunus cerasifera* genotypes with permanent red or green leaves. In genotypes with red leaves, the red colour is associated with the production of anthocyanins, a soluble pigment class^10,21^. Anthocyanins confer an enhanced photoprotection from excess solar irradiation in several plant species^16,22^, as already has been observed in *P. cerasifera, Jathropha curcas, Juglans regia* and *Ocimum basilicum*^10,23–25^. When studying ontogenetic differences between green and anthocyanin-rich leaves, the ideal strategy is using isogenic lines for this character, in order to exclude differences related to the genetic background. Nevertheless, this would require a number of backcrosses and would be very time-consuming because the length of the life cycle of a tree species. For this reason, several studies compare two cultivars of the same species differing in leaf colour phenotype^10^. Other differences between red and green leaves have been detected for leaf thickness^21,26^, wax formation, flavonoid production, sugar translocation^10,27^ and transcription factor activities of MYB and WD40 families^23,25^.

During the last decades, differences between genotypes with green or red leaves have been widely studied at morphological, physiological and genetic levels, and several works have concerned differences throughout ontogenesis at the physiological and biochemical levels^10,28,29^, but not at the gene expression level.

Next-generation sequencing (NGS) and whole-transcriptome sequencing (RNA-seq) have become powerful tools to study gene expression in both model and non-model species. To date, studies have focused on many physiological aspects of annual and perennials species including development phase transition studies^5,30–33^. Recently, RNA-Seq technology was applied on two cultivars of sweet basil that differed in leaf colour, providing genomic resources for the functional role of anthocyanins in photoprotection^27^.

In this light, gene expression analysis and changes in global gene expression patterns during leaf senescence between green and red morphs add pivotal information about cell functions and metabolism alterations that underline the developmental transition from a photosynthetic active leaf to a senescing organ, which is a source of mobilisable nutrients before the breakdown of chloroplasts.

*Prunus cerasifera* is a fast-growing tree appreciated worldwide as ornamental species for its fruit production and as a rootstock for fruit trees^34,35^. This species usually grows in several regions, spanning from the Caucasian Mountains to central Asia^35,36^, showing genetic and morphological differences and counting more than 20 varieties^35^. From a genomic point of view, *P. cerasifera* is a diploid species with n = 8, but with different ploidy levels amongst cultivars^35–37^. Morphologically, *P. cerasifera* can have different phenotypes, as in the case of ‘Pissardii’ variety, which displays reddish rather than green leaves. The molecular mechanisms of leaf senescence have been well studied in model plants as well as in major food crops; however, currently research on leaf senescence in *Prunus* species is still limited. As a genetically controlled development process, leaf senescence can be regulated by gene mutations, genetic transformation and molecular marker assisted breeding; nevertheless, there is still a long way to go to elucidate its complex mechanisms.

Here, for the first time, leaf transcriptome analyses were carried out on permanently green (clone ‘29C’) and red (var. ‘Pissardii’) leaves of *P. cerasifera* and comparing these morphs at two stages, i.e. mature and early senescent leaves. The aim of our study was to explore differences at the molecular level that could explain possible advantages to coping with senescence between green and anthocyanin-rich leaves of *Prunus*. The accumulation of epidermal anthocyanin can indeed effectively sunscreen the *P. cerasifera* var. ‘Pissardii’ leaves reducing the light which burdens to chloroplast, therefore being beneficial in terms of photoprotection compared to anthocyanin-less leaves. In fact, it has been proposed that these pigments may act as an efficient sunscreen, protecting the leaf from excessive light radiation of green-yellow wavelengths of the solar spectrum and preventing photosynthesis inhibition during leaf senescence^7,8,10^. In this study, we identified genes and cellular functions involved in pigment formation, photosynthetic process, sugar metabolism and stress response that differentially regulate green and red leaves throughout leaf aging. Our dataset represents a useful tool for further studies aimed at assessing the eco-physiological role of red-pigmented senescing leaves. In this light, the study of molecular components and their functionality could represent a key to the development of hypotheses concerning the organisation and regulation of metabolic networks during leaf senescence.

## Results

### cDNA sequencing and aligning on reference transcriptome

A total of 394,782,702 single-end reads each of 75 bp from twelve libraries of *P. cerasifera* red (R) and green (G) leaf morphs at two time points of ontogenesis (M, S) were collected (Table. 1). The number of trimmed reads (length = 60 bp) per library spanned from 16,280,829 to 32,776,054, enough to establish a differential expression analysis^38^. After trimming, high quality reads were aligned on the reference transcriptome of *P. persica*, a closely related species that has been often used as reference in other RNA-seq studies on *Prunus* species^39,40^; on average, 84.23% of reads per sample successfully mapped on the reference transcripts (Table. 1).

**Table 1:** Summary statistics for the Illumina sequencing and mapping against *Prunus persica* reference transcriptome.

	Number of raw reads per library	Number of trimmed reads per library	Number of aligned reads on *P. persica* reference transcriptome	Percentage of aligned reads on *P. persica* reference transcriptome
MR (1)	31,759,654	23,077,507	19,570,380	84.8
MR (2)	38,777,662	29,012,760	25,667,849	88.47
MR (3)	25,763,508	18,449,318	16,118,901	87.37
SR (1)	25,504,344	20,016,215	17,380,180	86.83
SR (2)	43,076,093	32,776,054	25,180,060	76.82
SR (3)	36,026,636	25,443,369	19,742,844	77.6
MG (1)	37,441,250	28,200,520	23,819,254	84.46
MG (2)	40,493,557	30,598,845	26,088,959	85.26
MG (3)	29,219,435	21,250,572	18,837,724	88.65
SG (1)	21,997,985	16,280,829	13,741,440	84.4
SG (2)	25,511,700	18,764,806	15,901,124	84.74
SG (3)	39,210,878	28,763,070	23,429,268	81.46

M = mature leaf; S = senescent leaf; R = *Prunus cerasifera* red morph G = *Prunus cerasifera* green morph.

Illumina reads from green and red mature and senescent leaves of *P. cerasifera* were deposited on SRA under bio project accession PRJNA593381.

### Analysis on differentially expressed genes of red and green *P. cerasifera* senescent leaves

**Figure 1:**
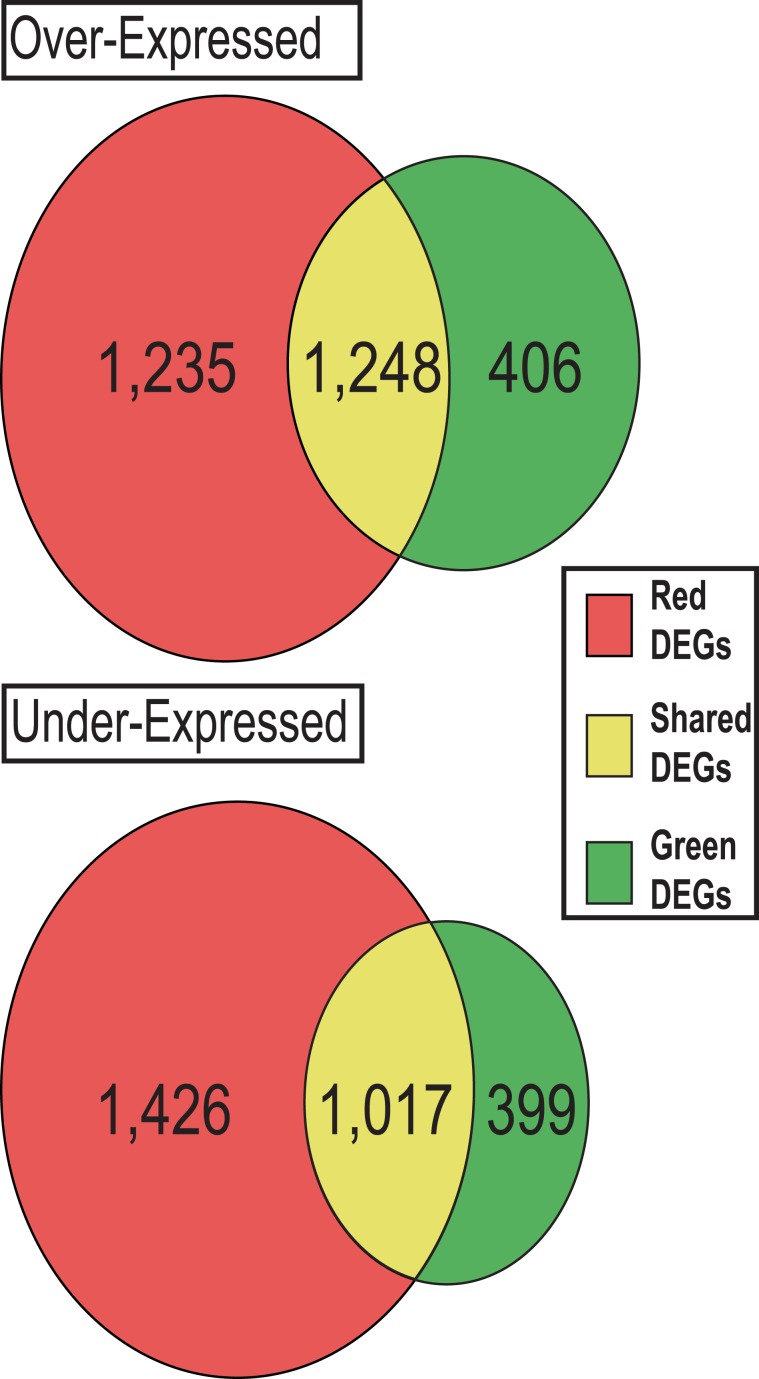
Venn diagram for over and under-expressed DEGs of *Prunus cerasifera* red and green leaves during ontogenesis. Red circle represents differentially expressed genes (DEGs) in the red morph whereas green area is for DEGs of green leaves. Shared genes are in the yellow intersection.

After filtering, 19,496 expressed transcripts with RPKM > 1 in at least one library were kept and corresponding expression values were statistically tested in order to detect DEGs of red and green leaves during senescence. Overall, we detected 3,070 DEGs during green leaf ontogenesis, of which 1,654 were over-expressed (OE), and 1,416 were under-expressed (UE). Concerning the red genotype, 4,925 DEGs were retrieved with 2,483 and 2,442 over- and under-regulated genes, respectively (Fig. 1; Supplementary Table. 1).

Comparison between OE genes of the two morphs during ontogenesis showed that DEGs were mostly shared, except for 406 and 1,235 transcripts that were specifically activated by green and red leaves during senescence, respectively (Fig. 1; Supplementary Table. 1). Considering the genes showing the 15 highest fold changes in expression, among DEGs activated in green morphs, we identified transcripts related to transcription factor activity (encoding a “*WRKY family protein*”) and in cell wall remodelling (encoding one “*laccase*”). Concerning DEGs with the highest over-expression fold change in red leaves, many were involved in cell metabolism (encoding three “*beta-glucosidases*” and one “*invertase*”) and in cell stress prevention such as one encoding a “*cytochrome P450 oxidase*” (Supplementary Table. 1).

Regarding UE genes, the comparison between morphs showed that green leaves turned off 399 genes specifically during senescence, whereas 1,426 transcripts were specifically down-regulated in red leaves (Fig. 1). Concerning genes showing the highest fold changes during ontogenesis, in green morphs they were mostly related to transcription factor activity (encoding “*MYB domain protein*” and “*bHLH DNA binding domain protein*”). Highly under-expressed DEGs of the red morph were mainly involved in metabolic processes (e.g. three genes encoding “*GDSL-like lipases*”). A complete set of DEGs in green and red leaves of *P. cerasifera* during ontogenesis is shown in Supplementary Table. 1.

Under- and over-regulated DEGs in green and red leaves during senescence were analysed by gene ontology. The distribution of GO terms in DEGs was very similar for green and red leaves throughout ontogenesis, with red leaves showing higher GO counts for all GO terms (Supplementary Fig. 1). For OE genes of the two morphs, the most abundant GO terms in cellular components were “Membrane” (GO:0016020), “Cell” (GO:0005623) and “Intracellular” (GO:0005622). Concerning molecular function, we detected many genes involved into “Catalytic activity” (GO:0003824), “Transferase activity” (GO:0016740) and “Protein binding” (GO:0005515). Finally, considering biological processes, we retrieved mostly “ Metabolic process” (GO:0008152); “Cellular process” (GO:0009987) and “Localization” (GO:0051179) terms (Supplementary Fig. 1).

Concerning UE DEGs in green and red leaves, the GO distribution of cellular components concerned mainly “Membrane” (GO:0016020), “Cell” (GO:0005623) and “Organelle” (GO:0043226) terms. For molecular function, the most abundant GO terms were “Catalytic activity” (GO:0003824) and “Protein binding” (GO:0005515). Regarding biological processes, GO terms related to “Metabolic process” (GO:0008152) and “Cellular process” (GO:0009987) were mainly found (Supplementary Fig. 1).

### GO enrichment analysis for specific DEGs of red and green leaves throughout senescence

**Figure 2:**
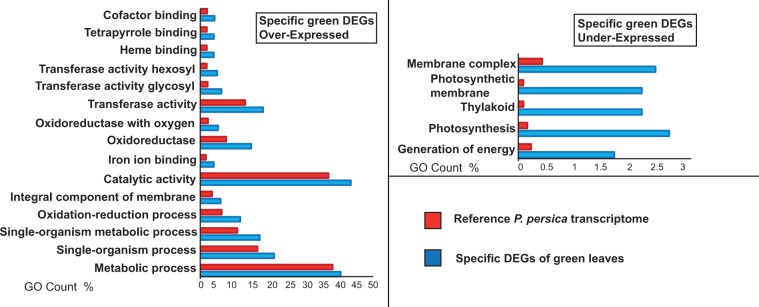
GO enrichment analysis for specific genes activated and repressed only by green leaves of *Prunus cerasifera* during senescence. Blue bars represent percentage of GO terms in DEGs. Red bars are the percentage of GO terms in reference transcriptome. GO terms summarized by REVIGO are shown.

**Figure 3:**
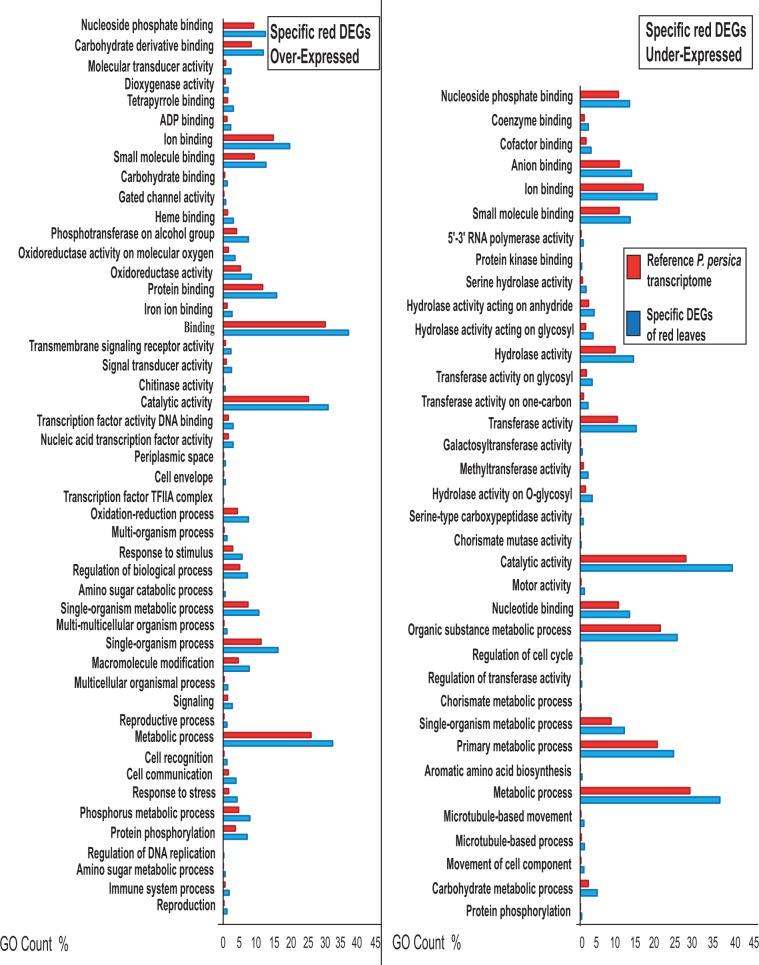
GO enrichment analysis for specific genes activated and repressed only by red leaves of *Prunus cerasifera* during senescence. Blue bars represent percentage of GO terms in DEGs. Red bars are the percentage of GO terms in reference transcriptome. GO terms summarized by REVIGO are shown.

GO enrichment analyses were performed on DEGs activated or repressed specifically in green and red leaves during ontogenesis. For over-expressed DEGs, Fisher tests were run on 406 and 1,235 activated genes from green and red leaves, respectively (Fig. 1). Fifteen terms were significantly GO-enriched in OE transcripts of green leaves, involved, for example, in “Transferase activity” (GO: 0016740), “Catalytic activity” (GO:0003824), “Oxidoreductase activity” (GO: 0016491) and “Metabolic process” (GO:0008152) (Fig. 2). In OE genes of red morphs, we found 95 enriched GO terms, involved, for example, in “Response to stress” (GO: 0006950), “Response to stimulus” (GO: 0050896) and “Carbohydrate binding” (GO: GO:0030246) (Fig. 3). Similarly, GO-enrichment was performed for 399 and 1,426 under-regulated genes of green and red leaves throughout senescence, respectively (Fig. 1). Only 5 enriched GO terms were found in under-regulated DEGs of green leaves, and all were involved into photosynthesis regulation (Fig. 2). Thirty-six enriched GO terms were found for under regulated DEGs of red morphs, they were involved, for example, in “Carbohydrate metabolic process” (GO: 0005975), “Chorismate mutase” (GO:0004106) and “Serine-type carboxypeptidase activity” (GO:0004185) (Fig. 3).

### MapMan and KEGG analysis on DEGs of red and green morphs during senescence

**Figure 4:**
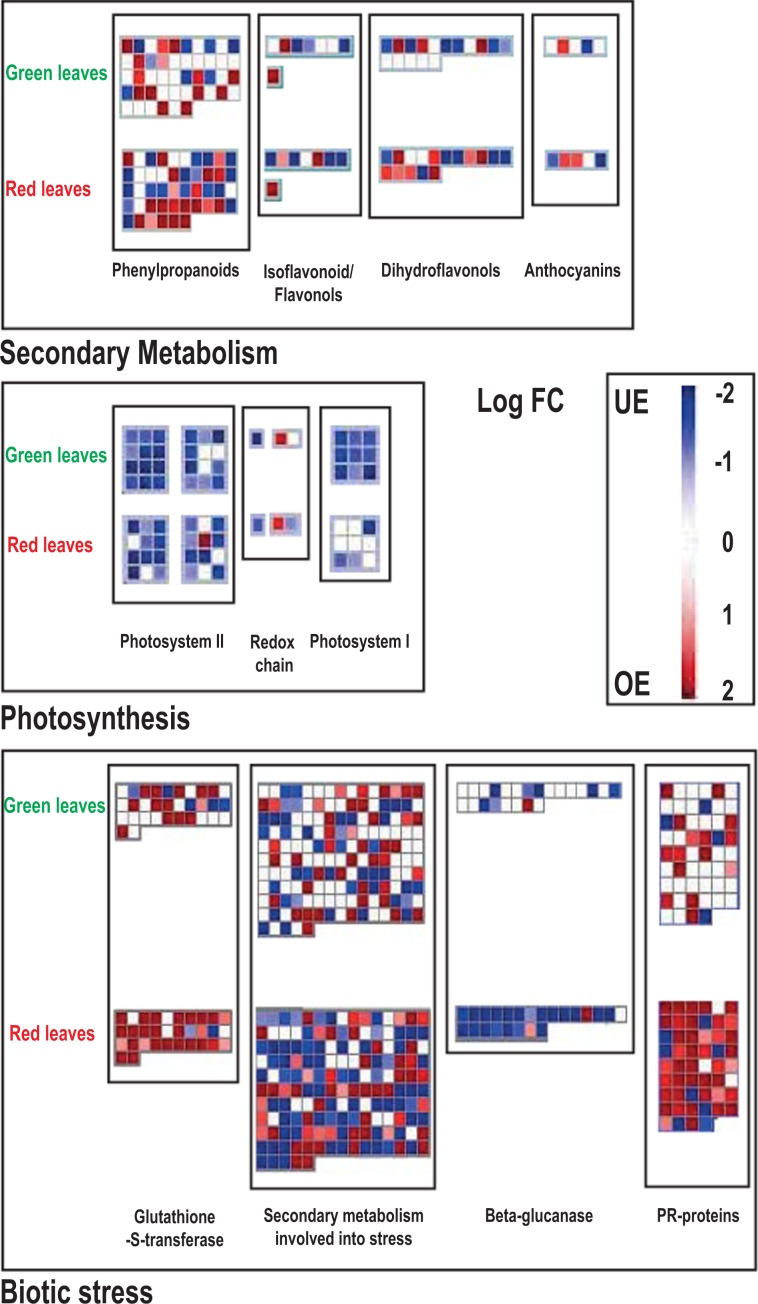
MapMan analysis for differentially expressed genes both in red and green *Prunus cerasifera* leaves throughout ontogenesis. Maps for secondary metabolism, photosynthesis and biotic stress were shown. Red dots are the over-expressed genes whereas blue dots are under-expressed ones. White dots indicate genes which were not differentially expressed in one morph but in the other. The scale, based on gene fold change, span from dark blue (Log FC = −2) to dark red (Log FC = 2).

MapMan was run on all DEGs of both morphs in order to compare functional classes during senescence. More genes related to the secondary metabolism were regulated in red compared to green leaves (Fig. 4). Considering phenylpropanoids, overall 37 DEGs were retrieved in red leaves (17 UE, 20 OE) compared to 22 in green leaves (8 UE, 14 OE) (Fig. 4). Amongst these, we detected three “*Cinnamyl alcohol dehydrogenase*” encoding transcripts that were specifically activated in red leaves and a “*Hydroxycinnamoyl-CoA shikimate*” protein, which was expressed only during the ontogenesis of green leaves.

Regarding genes involved in flavonoid and anthocyanin biosynthesis throughout senescence in the red morph, we retrieved 13 and 12 OE and UE transcripts, respectively (Fig. 4). For example, a HXXXD-type acyl-transferase encoding transcript, homologous to the *Prunus mume UDP glucose-flavonoid-3-O-glucosyltransferase* (*UFGT*) was up-regulated. For the green morph, 16 DEGs were included in these functional classes and most of them were down-regulated such as a transcript encoding another member of the *HXXXD-type acyl-transferase family* (Fig. 4 and Supplementary Table. 1).

Analysis on the “Photosynthesis” map, focusing on genes involved in light reactions, showed that a major under-regulation of this functional class occurred during ontogenesis in both genotypes, and especially in green leaves (Fig. 4). Differences between genotypes were mostly found in the regulation of genes involved in photosystem I, such as those encoding “*Subunits G, I, and H-1*” and over-expression of a “*PsbQ-like 1*” transcript.

Finally, exploring the “biotic stress” bin in MapMan and considering functional classes such as “Glutathione-S-transferase”, “Secondary metabolites involved in stress”, “Beta-glucanase” and “Pathogen-Related proteins“ (PR), we retrieved a major regulation of the red morph throughout senescence with overall 230 vs. 129 DEGs, respectively (Fig. 4). Functional classes, such as glutathione-S-transferase, PR-proteins and beta-glucanases, showed substantial differences in gene regulation between genotypes, as shown in Fig. 4.

**Figure 5:**
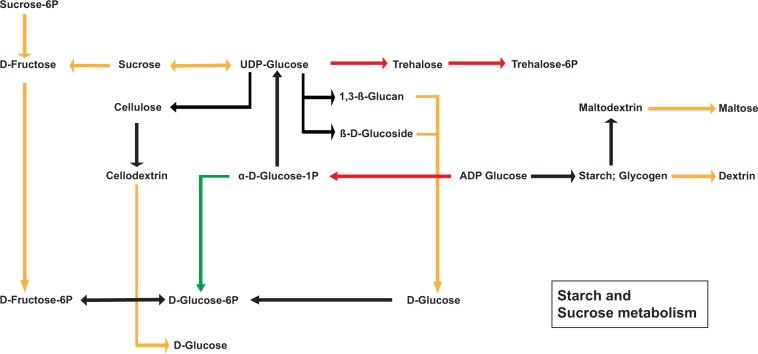
Summarized sketch for starch and sucrose map from KEGG^74^. Red and green arrows are the enzymatic pathway for over-expressed DEGs in respectively red and green *Prunus cerasifera* morphs during senescence. Pathway activated from both leaf morphs are yellow underlined. Not activated pathways are represented by black arrow.

KEGG analysis was performed in order to analyse carbohydrate pathways for over-regulated DEGs during senescence. Concerning the “Starch and sucrose metabolism”, we detected 13 activated pathways, among these 9 were shared between the two morphs (Fig. 5). A gene encoding the enzyme that converts glucose 1-P to glucose-6-P was specifically activated in green leaves, whereas enzymes implied into trehalose and ADP-glucose synthesis were exclusively activated in our red morph. These enzymes encode for a “*Phosphoglucomutase*” in green leaves and three “*Trehalose 6-phosphate synthases*” and a “*Nudix hydrolase isoform 14*” in the red morph. No differences were detected for this pathway for under-regulated DEGs of green and red leaves during ontogenesis.

## Discussion

The process of senescence is the final stage of leaf development, involving a massive degradation of cellular components with nutrient resorption and ROS accumulation on senescent leaves through highly regulated molecular processes^1,6,15,16^.

*P. cerasifera* green and red morphs showed physiological differences during senescence in terms of photosynthetic rates, carbon metabolism and photoprotective mechanisms^10^. In addition, it was suggested that, besides a photoabatement role, anthocyanins might also be synthesised to avoid a sugar accumulation that could trigger leaf senescence phenomena, thus extending the leaf lifespan in the red morph^10^.

In this study, for the first time, a transcriptome analysis of *P. cerasifera* leaves was performed to analyse differences in gene expression and cell functions between red and green leaves genotypes during the process of senescence. In addition, RNA-Seq analyses were conducted on the same saplings used by Lo Piccolo *et al*.^10^; hence, molecular regulation of transcripts underpins relevant physiological and biochemical data already observed, completing the biological investigation of leaf senescence process in two different genotypes of *P. cerasifera*.

Transcriptomic approach evidenced a general higher gene regulation of red leaves compared to green ones during ontogenesis; nevertheless, many DEGs were shared between the two morphs as shown in Fig. 1 and Fig. 4. The occurrence of commonly regulated genes between genotypes can be explained considering that both leaf morphs cope with the same process of senescence. In fact, many of the common genes are related to leaf aging. Among these genes, we retrieved transcription factors (of the *NAC*, *MYB*, *AP2*, *YIPPEE* and *WRKY* families) which are known to regulate senescence in leaves, *SAG* genes, whose expression increases during leaf aging controlling developmental process, cell wall loosening/remodelling-related transcripts (*expansins*, *pectin-methyltransferases*, *exostosin*) and genes related to nutrient remobilisation, such as *vacuolar sorting receptor proteins*, *xanthine/uracile permeases* and *purple acid permease* (see Supplementary Table. 1)^2,5,9,30^.

Although many transcripts were shared between red and green morphs during leaf senescence, while some others showed remarkable differences. Red leaves showed more DEGs involved in phenylpropanoid synthesis throughout. Some transcripts related to this pathway are involved in leaf stress prevention (Fig. 4), such as those encoding *cinnamyl alcohol dehydrogenase* (*CAD*), which produces monolignols involved in plant defence and activated during senescence in *Arabidopsis*^41,42^. Concerning cell stress response genes differentially expressed during ontogenesis, we detected considerable differences between green and red leaves, with an extensive over-expression of *glutathione-S-transferase* genes (*GST*) and *Pathogen-Related* (*PR*) genes in green leaves and an extensive down-regulation of *beta-glucanase* and *serine-type carboxipeptidases* transcripts in our red morph (Figs. 3 and 4). The *GST* and *PR* families are induced by several stresses and are known to be upregulated during leaf senescence in several species^9,43,44^. These enzymes play pivotal roles in both cell detoxification and defence against pathogens^44,45^. On the contrary, *beta-glucanases* and *serine-type carboxypeptidases* are mainly involved in cell wall loosening during leaf senescence^46–48^. These data may suggest that the red morph copes with leaf aging by activating the expression of genes involved in stress response and ensures cell wall integrity, maintaining more “healthy” leaves compared to the green one.

Surprisingly, genes involved in the shikimate pathway (e.g. encoding *chorismate mutase* and *hydroxycinnamoyl-CoA shikimate transferase*), which are related to defence mechanism and aromatic amino acid (AAA) production^49–51^, seem to be activated in green leaves, but down-regulated in the red morph. Recently, it has been established that shikimate metabolism is far more active in red leaves of basil compared to green ones^27^. Nevertheless, the gene expression of this pathway during ontogenesis in different morphs is little known.

In our experiments, we also retrieved more regulated specific genes of the flavonoid pathway in red morph leaf ontogenesis compared to the green morph (Fig. 4), suggesting a possible transcriptional regulation of anthocyanins synthesis occurring in red leaves throughout the senescence process, as observed at the physiological and biochemical levels by Lo Piccolo *et al*.^10^. In this sense, a transcript encoding a homolog to *UDP glucose-flavonoid-3-O-glucosyltransferase* (*UFGT*) was specifically up-regulated only in the red morph (Fig. 4, Supplementary Table. 1). This protein plays a key role in anthocyanin stability and solubility, transferring a glucosyl group from UDP-glucose to the 3-hydroxyl anthocyanidins^52^. In addition, many beta-glucosidase transcripts encoding enzymes involved in anthocyanins turn-over and stability^53^ were differentially expressed in both genotypes; however, two transcripts related to these enzymes were specifically over-expressed with a high fold change in red leaves (Supplementary Table. 1). Worthy of notice is the differential expression of three *threalose-6-phosphate synthases*, detected exclusively in the red morph (Fig. 5). These enzymes play a key role in threalose-6-phosphate production, an essential signal metabolite that is essential together with sucrose to stimulate anthocyanin synthesis^54^. In fact, anthocyanins might further act as sugar sinks^7,8^, thereby helping to avoid a carbohydrate accumulation in leaves and the consequent sugar-induced senescence^10^. Moreover, other genes involved in sugar metabolism were retrieved during the experiment, e.g. *GDSL-like lipases* that are involved in lipid breakdown. Their expression has been shown in senescing leaves of *Arabidopsis*^55^. We showed a large down-regulation (with high fold change) of these genes in red leaves, with a consequent possible reduction of carbohydrate mobilisation. The earliest and most dramatic sign of senescence in leaves is the degradation of chloroplasts with consequent breakdown of chlorophyll and the down-regulation of genes related to photosynthesis^1,2,9,56^. We retrieved an overall major down-regulation of photosynthetic functions of green leaves in *P. cerasifera* compared to red ones, as shown both by the GO terms and by MapMan analysis (Figs. 2 and 4). In our experiment, green leaves during early senescence showed a more extensive decrease of gene expression related to photosystem I (PSI) than red leaves, especially for *subunits G, I and H*, and partially to photosystem II (PSII). A faster degradation of PSI compared to PSII during leaf senescence has been established in several plants such as in rice and beans^57,58^ (Fig. 4). In addition, a gene encoding for *Pheophorbide A Oxygenase* (*PAO*), a key enzyme for chlorophyll breakdown, was detected as being over-expressed only in green leaves but not in the red morph (See Supplementary Table. 1). This gene is highly activated during cell senescence and is involved into chlorophyll breakdown, and mutants for this enzyme have shown an enhanced resistance to aging^2,59,60^. On the contrary, the red morph showed fewer under-regulated genes involved in photosynthesis (Fig. 4). Especially in ‘Pissardii’, we detected an over-expression of *PsbQ-like 1* transcript, encoding for a protein involved in the function of chloroplast NAD(P)H Dehydrogenase complex in *Arabidopsis*; mutants for these genes lead to a reduction of photosynthetic rate^61^. These data are in line with our previous physiological work, in which 13-week-old leaves of the red morph showed about a 26% increase in net photosynthetic rate compared to those of green morph^10^ and with that found in other species^21,26,62^, suggesting a strong delay in the senescing process in red leaves. Interestingly, transcripts involved in carotenoid biosynthesis, like those encoding *lycopene cyclase*, were also strongly up-regulated, especially in green leaves (Supplementary Table. 1), suggesting a possible necessity of carotenoid synthesis during the senescence of green leaves of *Prunus*, as reported by Lo Piccolo *et al*.^10^.

In conclusion, *P. cerasifera* green and red leaves during ontogenesis showed substantial differences. Although many transcripts were shared, the transcriptome of the red morph was highly regulated with differences, compared to the green one, in cellular functions, such as photosynthesis, the anthocyanin pathway, cell stress prevention and nutrient metabolism. Our results suggest that red morphs could regulate a set of genes that can cope with maturity-to-senescence transition leading to a delay of senescence processes.

This dataset offers clear evidence of inherent molecular differences between red and green leaves of *Prunus* during leaf senescence. In particular, the onset of senescence occurring in red leaves resulted in the activation of anthocyanin-related transcripts and some genes connected to sugar metabolism, which both (sunscreen and sugar-buffering effect of anthocyanins) could explain the higher photoprotection found in these leaves and their delayed senescence when compared to the green counterparts. Indeed, green leaves had higher levels of transcript related to carotenoid biosynthesis, but altogether a more extensive decrease of gene expression related to PSI and PSII degradation and chlorophyll breakdown, which are supportive for an early senescence of the leaf. Anthocyanins accumulate in the range of µM concentration in *Prunus* leaves during leaf senescence (see the anthocyanin index reported by Lo Piccolo *et al*.^10^) and at those concentrations, anthocyanins can abate a considerable amount of light (25–35% of incident light)^7^. Consequently, anthocyanins efficiently photoprotect the red leaves during foliar senescence.

The present study demonstrates the benefits of being red during the vulnerable phase of leaf senescence and represents the first attempt to summarise molecular changes occurring in foliar senescence in green and red genotypes, thus providing basic information for future research aimed at assessing the eco-physiological roles of anthocyanins and establishing the molecular origin of this intriguing class of flavonoids.

## Materials and Methods

### Experimental protocol and samplings

Saplings are the same reported by Lo Piccolo *et al*.^10^; in particular three-year-old *P. cerasifera* saplings (clone ‘29C’ with green leaves; var. ‘Pissardii’, with red leaves) were purchased from an Italian nursery (Vivai Battistini, Cesena, Italy). Stems of both morphs were grafted onto ‘29C’ rootstock in November 2016. One month after grafting, saplings were transplanted to 6.5-L pots in a growing medium containing a mixture of Einhetserde Topfsubstrat ED 63 standard soil (peat and clay, 34% organic C, 0.2% organic N and pH 5.8–6.0) and sand (3.5:1 in volume). Saplings of both morphs were maintained under greenhouse conditions until March 2017, when they were transferred to the field. One week after leaf emergence (early July 2017), homogeneous leaves from saplings of both morphs were marked to be followed throughout ontogenesis (sampled at 1, 7 and 13 weeks after leaf emergence). Leaf samples at 7 and 13 weeks (mature and senescent leaves, respectively) were used for molecular analyses^10^.

### RNA isolation and sequencing

Red (R) and green (G) leaves 7 and 13 weeks after emergence (hereafter called mature, M, and early senescent, S, respectively) were collected. Overall, three biological replicates were chosen for each morph, and time point and RNA isolation was performed using the Spectrum Plant Total RNA Kit (Sigma-Aldrich, St. Louis, MO, USA) following manufacturer’s instructions, but adding 2% PVP (Wt 40000, Sigma-Aldrich, St. Louis, MO, USA) to Lysis Solution. After elution, DNAse I (Roche) digestion was performed in order to remove genomic DNA traces. Hence, RNA was purified by phenol/chloroform extraction and ethanol precipitation following standard procedures^63^. Twelve RNA-seq libraries were obtained using the TruSeq RNAseq Sample Prep Kit, according to manufacturer’s protocol (Illumina, San Diego, CA, USA). The poly-A tail was used to isolate the mRNA fraction from total RNA. Then, mRNA was chemically fragmented and subsequently converted to cDNA. Adapters were ligated to the cDNA, and 200 ± 25 bp fragments were gel purified and enriched by PCR. Libraries were analysed by Bioanalyzer 2100 (Agilent Technologies, Santa Clara, CA, USA) and sequenced with Illumina HiSeq. 2000 (Illumina) using version 3 reagents. Single-end read sequences of 75 bp length were collected. Overall quality was checked by FastQC (v. 0.11.5) and improved by Trimmomatic^64^ (v. 0.36) using the following parameters: SLIDINGWINDOW: 4:20; HEADCROP: 15; MINLEN: 60. Any ribosomal traces were removed from libraries using CLC Genomic Workbench (v. 9.5.3, CLC-BIO, Aarhus, Denmark) aligning reads on *Prunus* rRNA obtained from the SILVA repository^65^.

### Differential expression and gene ontology analysis

High quality trimmed reads were aligned on the reference transcriptome of *P. persica* (version 2.1, https://phytozome.jgi.doe.gov/pz/portal.html#!info?alias = Org_Ppersica) using CLC Genomic Workbench as follows: mismatch cost = 2; insertion/deletion cost = 3, length fraction = 0.8, similarity fraction = 0.8. CLC Genomic Workbench distributes multi-reads among similar sequences up to ten sequences; this strategy allows a correct estimation of closely paralogous genes^66^.

The R package “edgeR” was used to analyse raw counts per transcripts (Robinson *et al*. 2010); gene expression was calculated as reads per kilobase per million reads mapped^66^ (RPKM), and only genes with RPKM > 1 in at least one library were retained. Statistical tests by “edgeR”^67^ were performed between counts of mapped reads for each gene in mature and senescent leaves samples of both genotypes (MG vs. SG; MR vs. SR) using the quasi-likelihood test as described by the user’s guide instructions and as suggested by Anders *et al*.^68^. Resulting P-values were corrected with the False Discovery Rate^69^ (FDR) and genes with FDR < 0.05 were selected as significant.

A gene was considered differentially expressed when the RPKM value in the senescent leaves was at least two fold higher or lower than in mature leaves. In this way, we were able to distinguish differentially expressed genes in two groups: up- and down-regulated.

Annotations and GO terms for each gene were downloaded from the phytozome database of *P. persica*. GO enrichment was performed using Blast2GO^70^ with the Fisher exact test comparing GO term distribution in the DEGs vs. the whole transcriptome GO id annotation of *P. persica*. Subsequently P-values were filtered by FDR (corrected P-value < 0.05). REVIGO^71^ was performed to reduce GO complexity using the parameter “tiny similarity”. In addition, “GO slim” was run by Blast2GO^70^ in order to identify major ontology classes.

MapMan was used for the functional analysis of DEGs; bins of *P. persica* were supplied with the software^72^. KO id codes for DEGs were obtained using KAAS^73^, and corresponding codes were submitted to KEGG^74^ for pathway network analysis (Kyoto Encyclopaedia of Genes and Genomes); KAAS alignment was performed by default parameters except for “single directional best-hit” (SBH), similarly to the work described by Torre *et al*.^27^.

## Supporting Information

Supplementary information.

## References

[1] Masclaux C, Valadier MH, Brugière N, Morot-Gaudry JF, Hirel B (2000). Characterization of the sink/source transition in tobacco (*Nicotiana tabacum* L.) shoots in relation to nitrogen management and leaf senescence. Planta.

[2] Kim, H. J., Lim, P. O. & Nam, H. G. Molecular regulation of senescence in *Annual Plant Reviews* (ed. Gan, S.) 180–181 (2007).

[3] Wu XY, Kuai BK, Jia JZ, Jing HC (2012). Regulation of leaf senescence and crop genetic improvement. J. Integr Plant Biol..

[4] Woo HR (2016). Programming of plant leaf senescence with temporal and inter-organellar coordination of transcriptome in *Arabidopsis*. Plant. Physiol..

[5] Kim J, Wuo HR, Nam HG (2016). Toward systems understanding of leaf senescence: an integrated multi-omics perspective on leaf senescence research. Mol. Plant.

[6] Keskitalo J, Bergquist G, Gardeström P, Jansson S (2005). A cellular timetable of autumn senescence. Plant. Physiol..

[7] Landi M, Tattini M, Gould KS (2015). Multiple functional roles of anthocyanins in plant-environment interactions. Environ. Exp. Bot..

[8] Gould KS, Jay-Allemand C, Logan BA, Baissac Y, Bidel LPR (2018). When are foliar anthocyanins useful to plants? Re-evaluation of the photoprotection hypothesis using *Arabidopsis thaliana* mutants that differ in anthocyanin accumulation. Environ. Exp. Bot..

[9] Lim PO, Kim HJ, Nam HG (2007). Leaf senescence. Annu. Rev. Plant. Biol..

[10] Lo Piccolo E (2018). Multiple consequences induced by epidermally-located anthocyanins in young, mature and senescent leaves of *Prunus*. Front. Plant. Sci..

[11] Bhattacharjee S (2005). Reactive oxygen species and oxidative burst: roles in stress, senescence and signal transduction in plants. Curr. Sci. India.

[12] Khanna-Chopra R (2011). Leaf senescence and abiotic stresses share reactive oxygen species-mediated chloroplast degradation. Protoplasma.

[13] Pintó-Marijuan M, Munné-Bosch S (2014). Photo-oxidative stress markers as a measure of abiotic stress-induced leaf senescence: advantages and limitations. J. Exp. Bot..

[14] Hörtensteiner S, Feller U (2002). Nitrogen metabolism and remobilization during senescence. J. Exp. Bot..

[15] Zimmermann P, Zentgraf U (2005). The correlation between oxidative stress and leaf senescence during plant development. Cell. Mol. Biol. Lett..

[16] Juvany M, Müller M, Munné-Bosch S (2013). Photo-oxidative stress in emerging and senescing leaves: a mirror image?. J. Exp. Bot..

[17] Wingler A, Roitsch T (2008). Metabolic regulation of leaf senescence: interactions of sugar signaling with biotic and abiotic stress responses. Plant Biol..

[18] Parrot DL, McInnerney K, Feller U, Fischer AM (2007). Steam-girdling of barley (*Hordeum vulgare*) leaves leads to carbohydrate accumulation and accelerated leaf senescence, facilitating transcriptomic analysis of senescence-associated genes. New Phytol..

[19] Lara MEB (2004). Extracellular invertase is an essential component of cytokinin-mediated delay of senescence. Plant Cell.

[20] Solfanelli C, Poggi A, Loreti E, Alpi A, Perata P (2006). Sucrose-specific induction of the anthocyanin biosynthetic pathway in *Arabidopsis*. Plant Physiol..

[21] Kyparissis A, Grammatikopoulos G, Manetas Y (2007). Leaf morphological and physiological adjustments to the spectrally selective shade imposed by anthocyanins in *Prunus cerasifera*. Tree Physiol..

[22] Hoch WA, Singsaas EL, McCown BH (2003). Resorption protection. Anthocyanins facilitate nutrient recovery in autumn by shielding leaves from potentially damaging light levels. Plant Physiol..

[23] Zhou Y (2014). Transcriptome analysis and transient transformation suggest an ancient duplicated MYB transcription factor as a candidate gene for leaf red coloration in peach. BMC Plant. Biol..

[24] Tattini M (2017). Dissecting molecular and physiological response mechanism to high solar radiation in cyanic and acyanic leaves: a case study on red and green basil. J. Exp. Bot..

[25] Li Y (2018). Comparative transcriptome analysis of genes involved in anthocyanin biosynthesis in red and green walnut (*Juglans regia* L.). Molecules.

[26] Wang C, Zhou J, Jiang K, Liu J (2017). Differences in leaf functional traits and allelopathic effects on seed germination and growth of *Lactuca sativa* between red and green leaves of *Rhus typhina*. S. Afr. J. Bot..

[27] Torre S (2016). De novo assembly and comparative transcriptome analyses of red and green morphs of sweet basil grown in full sunlight. PLoS One.

[28] Gould KS, McKelvie J, Markham KR (2002). Do anthocyanins function as antioxidants in leaves? Imaging of H 2 O 2 in red and green leaves after mechanical injury. Plant Cell. Environ..

[29] Neill SO, Gould KS, Kilmartin PA, Mitchell KA, Markham KR (2002). Antioxidant activities of red versus green leaves in *Elatostema rugosum*. Plant Cell Environ..

[30] Guo Y, Gan SS (2012). Convergence and divergence in gene expression profiles induced by leaf senescence and 27 senescence promoting hormonal, pathological and environmental stress treatments. Plant Cell Environ..

[31] Kong X (2013). Gene expression profiles dechiphering leaf senescence variation between early- and late-senescence cotton lines. PLoS One.

[32] Lin M (2015). Global analysis of the *Gossypium hirsutum* L. Transcriptome during leaf senescence by RNA-Seq. BMC Plant Biol..

[33] Wen CH, Lin SS, Chu FH (2015). Transcriptome analysis of a subtropical deciduous tree: autumn leaf senescence gene expression profile of formosan gum. Plant Cell Physiol..

[34] Eremin VG (1978). Genetic potential of species *Prunus cerasifera* Ehrh., and its use in breeding. Acta Hortic..

[35] Horvath A, Christmann E, Laigret F (2008). Genetic diversity and relationships among *Prunus cerasifera* (cherry plum) clones. Botany.

[36] Faust, M. & Suranyi, D. Origin and dissemination of plums in *Horticultural Review* (ed. Janick, J.) 179–231 (Wiley, 1999).

[37] Zohary D (1992). Is the European plum, *Prunus domestica* L., a *P. cerasifera* Ehrh. *P. spinosa* L. allo-polyploid?. Euphytica.

[38] Morin RD (2008). Comparative analysis of the small RNA transcriptomes of *Pinus contorta* and *Oryza sativa*. Genome Res..

[39] Arismendi MJ (2015). Transcriptome sequencing of *Prunus* sp. rootstocks roots to identify candidate genes involved in the response to root hypoxia. Tree Genet. Genomes..

[40] Collum TD, Lutton E, Raines CD, Dardick C, Culver JN (2019). Identification of phloem-associated translatome alterations during leaf development in *Prunus domestica* L. Hortic. Res..

[41] Quirino BF, Normanly J, Amasino RM (1999). Diverse range of gene activity during *Arabidopsis thaliana* leaf senescence includes pathogen-independent induction of defense-related genes. Plant Mol. Biol..

[42] Kim YH, Bae JM, Huh GH (2010). Transcriptional regulation of the cinnamyl alcohol dehydrogenase gene from sweet potato in response to plant developmental stage and environmental stress. Plant Cell Rep..

[43] Guo Y, Cai Z, Gan S (2004). Transcriptome of *Arabidopsis* leaf senescence. Plant Cell Environ..

[44] Edreva A (2005). Pathogenesis-related proteins: research progress in the last 15 years. Gen. Appl. Plant Physiology.

[45] Cobbett C, Goldsbrough P (2002). Phytochelatins and metallothioneins: roles in heavy metal detoxification and homeostasis. Annu. Rev. Plant Biol..

[46] Hanfrey C, Fife M, Buchanan-Wollaston V (1996). Leaf senescence in *Brassica napus*: expression of genes encoding pathogenesis-related proteins. Plant Mol. Biol..

[47] Palma JM (2002). Plant proteases, protein degradation, and oxidative stress: role of peroximsomes. Plant Physiol. Bioch..

[48] Zoran M (2008). Physiological roles of plant glycoside hydrolases. Planta.

[49] Weaver LM, Herrmann KM (1997). Dynamics of the shikimate pathway in plants. Trends Plant Sci..

[50] Herrmann KM, Weaver LM (1999). The Shikimate pathway. Annu. Rev. Plant Phys..

[51] Maeda H, Dudareva N (2012). The shikimate pathway and aromatic amino acid biosynthesis in plants. Annu. Rev. Plant Biol..

[52] Yoshihara N (2005). cDNA cloning and characterization of UDP-glucose: Anthocyanidin 3-O-glucosyltransferase in *Iris hollandica*. Plant Sci..

[53] Oren-Shamir M (2009). Does anthocyanin degradation play a significant role in determining pigment concentration in plants?. Plant Sci..

[54] Wingler A (2012). Trehalose 6-Phosphate is required for the onset of leaf senescence associated with high carbon availability. Plant Physiol..

[55] Troncoso-Ponce MA, Cao X, Yang Z, Ohlrogge JB (2013). Lipid turnover during senescence. Plant Sci..

[56] Matile, P. Chloroplast senescence in *Baker NR* (ed. Thomas, H.) 413–440 (Elsevier, 1992).

[57] Jenkins GI, Baker NR, Woolhouse HW (1981). Changes in chlorophyll content and organization during senescence of the primary leaves of *Phaseolus vulgaris* L. in relation to photosynthetic electron transport. J. Exp. Bot..

[58] Tang Y, Wen X, Lu C (2005). Differential changes in degradation of chlorophyll–protein complexes of photosystem I and photosystem II during flag leaf senescence of rice. Plant Physiol. Bioch..

[59] Pružinská A, Tanner G, Anders I, Roca M, Hörtensteiner S (2003). Chlorophyll breakdown: Pheophorbide a oxygenase is a Rieske-type iron–sulfur protein, encoded by the accelerated cell death 1 gene. Pnas.

[60] Schelbert S (2009). Pheophytin pheophorbide hydrolase (pheophytinase) is involved in chlorophyll breakdown during leaf senescence in *Arabidopsis*. Plant Cell.

[61] Yabuta S (2010). Three PsbQ-like proteins are required for the function of the chloroplast NAD(P)H dehydrogenase complex in. Arabidopsis. Plant Cell Physiol..

[62] Ranjan S, Singh R, Singh R, Pathre UV, Shirke PA (2014). Characterizing photoinhibition and photosynthesis in juvenile-red versus mature-green leaves of *Jatropha curcas* L. Plant Physiol. Bioch..

[63] Giordani T (2016). Genome-wide analysis of LTR-retrotransposon expression in leaves of *Populus x canadensis* water-deprived plants. Tree Genet. Genomes.

[64] Bolger AM, Lohse M, Usadel B (2014). Trimmomatic: a flexible trimmer for Illumina sequence data. Bioinformatics.

[65] Quast C (2013). The SILVA ribosomal RNA gene database project: improved data processing and web-based tools. Nucleic Acids Res..

[66] Mortazavi A, Williams BA, McCue K, Schaeffer L, Wold B (2008). Mapping and quantifying mammalian transcriptomes by RNA-Seq. Nat. Methods.

[67] Robinson MD, McCarthy DJ, Smyth GK (2010). EdgeR: a Bioconductor package for differential expression analysis of digital gene expression data. Bioinformatics.

[68] Anders S (2013). Count-based differential expression analysis of RNA sequencing data using R and Bioconductor. Nat. Protoc..

[69] Benjamini Y, Hocberg Y (1995). Controlling the false discovery rate: a pratical and powerful approach to multiple testing. J. R. Stat. Soc..

[70] Conesa A (2005). Blast2GO: a universal tool for annotation, visualization and analysis in functional genomics research. Bioinformatics.

[71] Supek F, Bošnjak M, Škunca N, Šmuc T (2011). REVIGO summarizes and visualizes long lists of gene ontology terms. PLoS One.

[72] Thimm O (2004). Mapman: a user‐driven tool to display genomics data sets onto diagrams of metabolic pathways and other biological processes. Plant J..

[73] Moriya Y, Itoh M, Okuda S, Yoshizawa AC, Kanehisa M (2007). KAAS:an automatic genome annotation and pathway reconstruction server. Nucleic Acids Res..

[74] Kanehisa M, Goto S (2000). KEGG: Kyoto Encyclopedia of Genes and Genomes. Nucleic Acids Res..

