# Supplementary material for: Red versus green leaves: transcriptomic comparison of foliar senescence between two Prunus cerasifera genotypes
Source: Sci Rep. 2020 Feb 6;10:1959. doi: 10.1038/s41598-020-58878-8 (PMC7005320; doi:10.1038/s41598-020-58878-8)
Supplement: Supplementary file 1 — Supplementary information. [file 41598_2020_58878_MOESM1_ESM.pdf]

Red versus green leaves: transcriptomic comparison of foliar senescence between two *Prunus cerasifera* genotypes.

Alberto Vangelisti[a], Lucia Guidi[a,b], Andrea Cavallini[a], Lucia Natali[a,b], Ermes Lo Piccolo[a], Marco Landi[a], Giacomo Lorenzini[a,b], Fernando Malorgio[a], Rossano Massai[a], Cristina Nali[a,b], Elisa Pellegrini[a,b], Giovanni Rallo[a,b], Damiano Remorini[a,b], Paolo Vernieri[a,b], Tommaso Giordani[a*]

[a] Department of Agriculture, Food and Environment, University of Pisa, Via del Borghetto 80 - 56124 Pisa, Italy

[b] CIRSEC, Centre for Climate Change Impact, University of Pisa, Via del Borghetto 80 - 56124 Pisa, Italy

***Corresponding author: Tommaso Giordani, Email: tommaso.giordani@unipi.it**

Supplementary Fig.1: Distribution of GO-slim terms in *Prunus cerasifera* green (green bars) and red (red bars) genotypes during ontogenesis. Over-expressed and under-expressed GO terms distribution are shown for each macro-category: cellular component (CC), molecular function (MF) and biological process (BP).

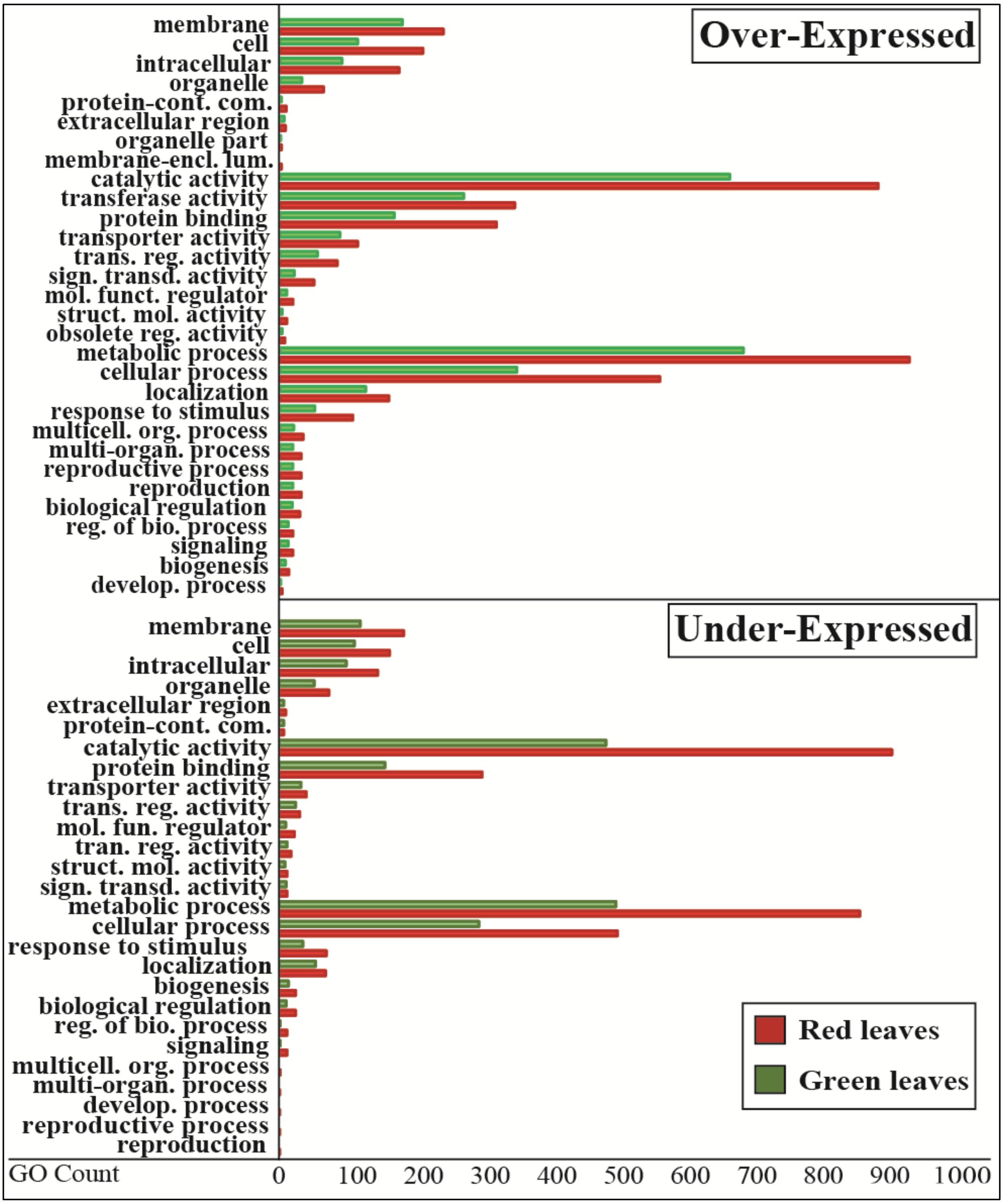

Supplementary Tab.1: Complete dataset of differentially expressed genes in Prunus cerasifera red and green morph during ontogenesis.

genes	logFC	FDR	Description	Red or green ontogenesis	Functional classes as detected by MapMan
ppa004103m	11.28	0.00827001	FAD-binding Berberine family protein	Green ontogenesis	
ppa003578m	11.01	0.021880331	Plant invertase/pectin methylesterase inhibitor superfamily	Green ontogenesis	
ppa015912m	10.64	0.00353236	Serine protease inhibitor, potato inhibitor I-type family protein	Green ontogenesis	
ppa025045m	10.41	0.02301413	allene oxide synthase	Green ontogenesis	
ppa019615m	10.01	0.00629101	phospholipase A 2A	Green ontogenesis	
ppa025067m	9.88	0.016626277	beta glucosidase 12	Green ontogenesis	
ppa025013m	9.69	0.008013007	WRKY family transcription factor	Green ontogenesis	
ppa003587m	9.54	0.00027599	laccase 7	Green ontogenesis	Biotic Stress / Secondary metabolism involved into stress
ppa004021m	9.48	0.001225256	FAD-binding Berberine family protein	Green ontogenesis	
ppa021329m	9.41	0.001356283	lycopene cyclase	Green ontogenesis	Biotic Stress / Secondary metabolism involved into stress
ppa021446m	9.37	0.003850454	Plant invertase/pectin methylesterase inhibitor superfamily	Green ontogenesis	
ppa025296m	9.12	0.021378387	cytochrome P450, family 81, subfamily D, polypeptide 8	Green ontogenesis	
ppa011310m	9.04	0.007157546		Green ontogenesis	
ppa014163m	8.79	0.019844071		Green ontogenesis	
ppa018214m	8.76	0.000132814	Late embryogenesis abundant protein (LEA) family protein	Green ontogenesis	
ppa026320m	8.71	0.008865798		Green ontogenesis	
ppa018404m	8.64	0.022179004	beta glucosidase 13	Green ontogenesis	
ppa027123m	8.59	0.016937617	F-box family protein with a domain of unknown function (DUF295)	Green ontogenesis	
ppa022609m	8.56	0.025072173		Green ontogenesis	
ppa026148m	8.51	0.002443863		Green ontogenesis	
ppa024321m	8.47	0.003295397	Exostosin family protein	Green ontogenesis	
ppa015297m	8.39	0.004482878	Ribonuclease H-like superfamily protein	Green ontogenesis	
ppa004592m	8.32	0.045938424	cytochrome P450, family 94, subfamily C, polypeptide 1	Green ontogenesis	
ppa013725m	8.31	0.014804134		Green ontogenesis	
ppa023749m	8.03	0.009342472	Late embryogenesis abundant (LEA) hydroxyproline-rich glycopro	Green ontogenesis	
ppa023146m	7.98	0.00220748	cation/hydrogen exchanger 15	Green ontogenesis	
ppa022596m	7.92	0.010151915	RmlC-like cupins superfamily protein	Green ontogenesis	
ppa013922m	7.92	0.006739393		Green ontogenesis	
ppa019803m	7.80	0.047182746	FAD-binding Berberine family protein	Green ontogenesis	
ppa017638m	7.77	0.002314377		Green ontogenesis	
ppa007061m	7.71	0.006203574	nodulin MtN21 /EamA-like transporter family protein	Green ontogenesis	
ppa010943m	7.69	0.002682662	Protein of unknown function (DUF679)	Green ontogenesis	
ppa017234m	7.67	0.005734965	Putative membrane lipoprotein	Green ontogenesis	
ppa004413m	7.65	0.034060411	Plant invertase/pectin methylesterase inhibitor superfamily	Green ontogenesis	
ppa020923m	7.57	0.007125118		Green ontogenesis	
ppa006071m	7.55	0.003936381	Eukaryotic aspartyl protease family protein	Green ontogenesis	
ppa017791m	7.47	0.025923511	basic helix-loop-helix (bHLH) DNA-binding superfamily protein	Green ontogenesis	
ppa016302m	7.36	0.002266721	blue-copper-binding protein	Green ontogenesis	
ppa026628m	7.36	0.012004496	Cytochrome P450 superfamily protein	Green ontogenesis	
ppa018203m	7.28	0.001797555	Phosphorylase superfamily protein	Green ontogenesis	
ppa017792m	7.24	0.009985452	Acyl-CoA N-acyltransferases (NAT) superfamily protein	Green ontogenesis	
ppa008191m	7.17	0.004648784	RmlC-like cupins superfamily protein	Green ontogenesis	
ppa026872m	7.11	0.015455923	phospholipase A 2A	Green ontogenesis	
ppa022990m	7.05	0.009334579	Uncharacterised protein family (UPF0497)	Green ontogenesis	
ppa006455m	7.04	0.022293597	plant U-box 24	Green ontogenesis	
ppa012174m	7.03	6.16E-05	Ribulose bisphosphate carboxylase (small chain) family protein	Green ontogenesis	
ppa022216m	6.98	0.001013823	FAD-binding Berberine family protein	Green ontogenesis	
ppa023604m	6.98	0.02462496	Peroxidase superfamily protein	Green ontogenesis	
ppa013192m	6.91	0.00898284		Green ontogenesis	
ppa026968m	6.79	0.020618608		Green ontogenesis	
ppa004108m	6.77	0.005338751	beta glucosidase 13	Green ontogenesis	
ppa023987m	6.75	0.001152163	methyl esterase 3	Green ontogenesis	
ppa015635m	6.73	0.00042149	methyl esterase 3	Green ontogenesis	
ppa020030m	6.71	0.000300697	bidirectional amino acid transporter 1	Green ontogenesis	
ppa014784m	6.70	0.004759398		Green ontogenesis	
ppa011068m	6.67	0.027313687		Green ontogenesis	
ppa1027169m	6.62	0.002306433	plant U-box 23	Green ontogenesis	
ppa007477m	6.61	0.014745887	2-oxoglutarate (2OG) and Fe(II)-dependent oxygenase superfami	Green ontogenesis	
ppa022181m	6.61	0.021308868	Uncharacterised protein family (UPF0041)	Green ontogenesis	
ppa017962m	6.61	0.03034136	PLAT/LH2 domain-containing lipoxygenase family protein	Green ontogenesis	
ppa020910m	6.59	0.008233103	Calcium-binding EF-hand family protein	Green ontogenesis	
ppa019283m	6.59	0.000309766	NB-ARC domain-containing disease resistance protein	Green ontogenesis	
ppa007396m	6.58	0.003913056	Glycosyl hydrolase family protein with chitinase insertion domain	Green ontogenesis	
ppb013610m	6.55	0.004098903		Green ontogenesis	
ppa004639m	6.54	0.000929961	cytochrome P450, family 76, subfamily C, polypeptide 4	Green ontogenesis	
ppa020067m	6.52	0.003702776	beta glucosidase 13	Green ontogenesis	
ppa022066m	6.38	0.022315825	C2H2-type zinc finger family protein	Green ontogenesis	
ppa020820m	6.37	0.018608069	UDP-glucosyl transferase 85A2	Green ontogenesis	
ppa002986m	6.37	0.009969838	Auxin-responsive GH3 family protein	Green ontogenesis	
ppa023226m	6.35	0.031891924	Protein kinase superfamily protein	Green ontogenesis	
ppa013728m	6.35	0.002824816	flowering promoting factor 1	Green ontogenesis	
ppa016280m	6.31	0.003717706	carboxyesterase 17	Green ontogenesis	
ppa015017m	6.30	0.034393557	GRAM domain family protein	Green ontogenesis	
ppa005711m	6.14	0.011694069	ARM repeat superfamily protein	Green ontogenesis	
ppa022045m	6.14	0.003232681	beta glucosidase 15	Green ontogenesis	
ppa006110m	6.14	6.59E-06	beta glucosidase 17	Green ontogenesis	
ppa009165m	6.13	0.000237564	NAD(P)-binding Rossmann-fold superfamily protein	Green ontogenesis	
ppa009047m	6.10	0.000227171	inositol polyphosphate 5-phosphatase 11	Green ontogenesis	
ppa016724m	6.09	0.024006353	Uncharacterised conserved protein UCP031279	Green ontogenesis	
ppa011448m	6.08	0.000969173	Kunitz family trypsin and protease inhibitor protein	Green ontogenesis	Biotic Stress / PR-proteins
ppa020605m	6.07	0.034995981	Integrase-type DNA-binding superfamily protein	Green ontogenesis	
ppa019880m	6.04	0.01217053	laccase 1	Green ontogenesis	Biotic Stress / Secondary metabolism involved into stress
ppa006324m	6.04	0.002673072		Green ontogenesis	
ppa009400m	5.98	0.000696543	methyl esterase 3	Green ontogenesis	
ppa022911m	5.97	0.0337879		Green ontogenesis	
ppa022513m	5.96	1.76E-05	beta glucosidase 13	Green ontogenesis	
ppa024928m	5.95	0.031298619	Pyridoxal phosphate (PLP)-dependent transferases superfamily p	Green ontogenesis	
ppa005094m	5.95	0.007404304	ACT domain repeat 1	Green ontogenesis	
ppa025645m	5.88	0.003420424		Green ontogenesis	
ppa003134m	5.87	0.00361244	Auxin-responsive GH3 family protein	Green ontogenesis	
ppa024790m	5.86	0.016283053	Concanavalin A-like lectin protein kinase family protein	Green ontogenesis	
ppa018984m	5.79	0.000242014	NAD(P)-binding Rossmann-fold superfamily protein	Green ontogenesis	
ppa018391m	5.79	0.001823737	Serine protease inhibitor, potato inhibitor I-type family protein	Green ontogenesis	
ppa024233m	5.79	0.021308868	tubulin beta-1 chain	Green ontogenesis	
ppa021087m	5.78	0.032426574	RmlC-like cupins superfamily protein	Green ontogenesis	
ppa015133m	5.78	0.000847276	UDP-glucosyl transferase 73B3	Green ontogenesis	
ppa004288m	5.76	0.012807575	beta glucosidase 15	Green ontogenesis	
ppa009089m	5.75	1.46E-05	early nodulin-like protein 14	Green ontogenesis	
ppa026827m	5.75	0.005239223	GDSL lipase 1	Green ontogenesis	
ppa008019m	5.69	0.04984386	plant intracellular ras group-related LRR 6	Green ontogenesis	
ppa019661m	5.69	0.000595187	Late embryogenesis abundant (LEA) hydroxyproline-rich glycopro	Green ontogenesis	
ppa024604m	5.67	0.002596451		Green ontogenesis	
ppa026111m	5.67	0.000896741	NB-ARC domain-containing disease resistance protein	Green ontogenesis	
ppa024027m	5.67	0.004622112	WRKY DNA-binding protein 28	Green ontogenesis	
ppa026982m	5.66	0.004075636	RAD-like 6	Green ontogenesis	
ppa019212m	5.64	0.000763273	Late embryogenesis abundant (LEA) hydroxyproline-rich glycopro	Green ontogenesis	
ppa011242m	5.64	0.005054094	glutathione S-transferase TAU 19	Green ontogenesis	Biotic Stress / Glutathione-S-Transferase
ppa014515m	5.64	0.009967242		Green ontogenesis	
ppa024500m	5.62	0.001659782	Serine protease inhibitor, potato inhibitor I-type family protein	Green ontogenesis	
ppa024352m	5.61	0.000506889	glutamate receptor 2.7	Green ontogenesis	
ppa005837m	5.60	0.001928443	Cupredoxin superfamily protein	Green ontogenesis	Biotic Stress / Secondary metabolism involved into stress
ppa017130m	5.58	0.005363739	UDP-glucosyl transferase 85A3	Green ontogenesis	
ppa017556m	5.55	0.002418203	Protein of unknown function (DUF630 and DUF632)	Green ontogenesis	
ppa000232m	5.54	0.000131152	pleiotropic drug resistance 11	Green ontogenesis	
ppa014920m	5.52	0.000190029	Chlorophyll A-B binding family protein	Green ontogenesis	
ppa018862m	5.51	0.000916598	Late embryogenesis abundant (LEA) hydroxyproline-rich glycopro	Green ontogenesis	
ppa025205m	5.47	0.001606805		Green ontogenesis	
ppa019653m	5.46	0.007605522		Green ontogenesis	
ppa020915m	5.45	0.000475695	Late embryogenesis abundant (LEA) hydroxyproline-rich glycopro	Green ontogenesis	
ppa011773m	5.45	0.000761436	Late embryogenesis abundant (LEA) hydroxyproline-rich glycopro	Green ontogenesis	
ppa008986m	5.43	6.82E-05		Green ontogenesis	
ppa019339m	5.42	0.014099975	VQ motif-containing protein	Green ontogenesis	
ppa011097m	5.38	0.003070371	related to AP2 6l	Green ontogenesis	
ppa014398m	5.37	0.005184854	magnesium-chelatase subunit chlH, chloroplast, putative / Mg-pro	Green ontogenesis	
ppa000233m	5.36	0.000278532	pleiotropic drug resistance 6	Green ontogenesis	
ppa021010m	5.36	0.000131152		Green ontogenesis	
ppa022042m	5.35	0.017701286	Protein of unknown function (DUF567)	Green ontogenesis	
ppa025672m	5.30	0.024359588	myb domain protein 108	Green ontogenesis	
ppa007162m	5.30	0.00321474	galacturonosyltransferase-like 10	Green ontogenesis	
ppb020933m	5.28	0.005287373	Leucine-rich repeat (LRR) family protein	Green ontogenesis	
ppa010909m	5.27	0.003952086	C-repeat/DRE binding factor 2	Green ontogenesis	
ppa005715m	5.27	0.025804232	HXXXD-type acyl-transferase family protein	Green ontogenesis	Secondary Metabolism / Phenylpropanoids - Biotic Stress / Secondary metabolism involved into stress
ppb015086m	5.26	0.044478985	Protein kinase superfamily protein	Green ontogenesis	
ppa023672m	5.24	0.002511476	plant U-box 23	Green ontogenesis	
ppa010199m	5.21	0.000713688	Late embryogenesis abundant (LEA) hydroxyproline-rich glycopro	Green ontogenesis	
ppa020689m	5.21	0.008005268	Predicted AT-hook DNA-binding family protein	Green ontogenesis	
ppa016936m	5.20	0.031761212	Protein phosphatase 2C family protein	Green ontogenesis	
ppa010355m	5.14	0.000399037	Late embryogenesis abundant (LEA) hydroxyproline-rich glycopro	Green ontogenesis	
ppb019918m	5.11	0.000561887	Chlorophyll A-B binding family protein	Green ontogenesis	
ppa007856m	5.10	0.018408478	2-oxoglutarate (2OG) and Fe(II)-dependent oxygenase superfami	Green ontogenesis	
ppa012242m	5.10	0.010110676	C2H2-type zinc finger family protein	Green ontogenesis	
ppa020888m	5.09	0.029929486	S-locus protein kinase, putative	Green ontogenesis	
ppa011524m	5.09	0.00030057	blue-copper-binding protein	Green ontogenesis	
ppb021349m	5.09	0.005338795	wall associated kinase 3	Green ontogenesis	
ppa010345m	5.05	0.011608705	Integrase-type DNA-binding superfamily protein	Green ontogenesis	
ppa014628m	5.04	0.001732361	C-repeat-binding factor 4	Green ontogenesis	
ppa026591m	5.01	0.008013007	alpha/beta-Hydrolases superfamily protein	Green ontogenesis	

ppa027018m	5.00	0.000944883	Late embryogenesis abundant (LEA) hydroxyproline-rich glycoprc	Green ontogenesis	
ppa021921m	5.00	0.012460629	myb domain protein 15	Green ontogenesis	
ppa020590m	5.00	0.009287367	BON association protein 2	Green ontogenesis	
ppb015892m	5.00	0.026765927	UDP-glucosyl transferase 85A2	Green ontogenesis	
ppa008301m	4.95	0.005240269	NAC (No Apical Meristem) domain transcriptional regulator super	Green ontogenesis	
ppb022421m	4.95	0.048572901	myb domain protein 15	Green ontogenesis	
ppa003718m	4.95	3.18E-05	beta glucosidase 15	Green ontogenesis	
ppa010586m	4.93	0.001390383	GRAM domain family protein	Green ontogenesis	
ppa023986m	4.93	0.000431196	S-adenosyl-L-methionine-dependent methyltransferases superfar	Green ontogenesis	
ppa022706m	4.91	1.76E-05	plant natriuretic peptide A	Green ontogenesis	
ppa019709m	4.91	0.017400881	calmodulin-like 38	Green ontogenesis	
ppa014464m	4.90	8.56E-05	Late embryogenesis abundant protein (LEA) family protein	Green ontogenesis	
ppa027148m	4.89	0.006708838	cinnamyl alcohol dehydrogenase 9	Green ontogenesis	Secondary Metabolism / Phenylpropanoids - Biotic Stress / Secondary metabolism involved into stress
ppa025047m	4.89	0.008953894	mitogen-activated protein kinase kinase kinase 15	Green ontogenesis	
ppa017730m	4.87	0.000896741		Green ontogenesis	
ppa013513m	4.86	0.025373918		Green ontogenesis	
ppa008412m	4.84	0.003562927	carboxyesterase 17	Green ontogenesis	
ppa025128m	4.83	0.008213071	Pyridoxal phosphate (PLP)-dependent transferases superfamily p	Green ontogenesis	
ppa014242m	4.83	0.038709317		Green ontogenesis	
ppa016345m	4.82	2.15E-05	Receptor-like protein kinase-related family protein	Green ontogenesis	
ppa004245m	4.81	0.012226838	flavin-dependent monooxygenase 1	Green ontogenesis	
ppa005427m	4.80	0.037665537	UDP-Glycosyltransferase superfamily protein	Green ontogenesis	
ppa022668m	4.79	0.008157405	phloem protein 2-B15	Green ontogenesis	
ppa026298m	4.77	0.005420577	Cysteine/Histidine-rich C1 domain family protein	Green ontogenesis	
ppa025011m	4.77	0.024204842	Late embryogenesis abundant (LEA) hydroxyproline-rich glycoprc	Green ontogenesis	
ppa018499m	4.75	0.042351887	Late embryogenesis abundant (LEA) hydroxyproline-rich glycoprc	Green ontogenesis	
ppa018079m	4.74	0.000442104	Late embryogenesis abundant (LEA) hydroxyproline-rich glycoprc	Green ontogenesis	
ppa011079m	4.72	0.000536059	glutathione S-transferase TAU 8	Green ontogenesis	Biotic Stress / Glutathione-S-Transferase
ppa000937m	4.70	1.76E-05	H(+)-ATPase 2	Green ontogenesis	
ppa024368m	4.69	0.005595346	MLP-like protein 423	Green ontogenesis	
ppa007615m	4.67	0.001875878	elicitor-activated gene 3-1	Green ontogenesis	Secondary Metabolism / Phenylpropanoids - Biotic Stress / Secondary metabolism involved into stress
ppa019252m	4.66	2.65E-05	Receptor-like protein kinase-related family protein	Green ontogenesis	
ppa022109m	4.65	0.025481413	cysteine-rich RLK (RECEPTOR-like protein kinase) 10	Green ontogenesis	
ppa022455m	4.64	0.016141016	Eukaryotic aspartyl protease family protein	Green ontogenesis	
ppa011370m	4.64	0.009287624	TIFY domain/Divergent CCT motif family protein	Green ontogenesis	
ppa020943m	4.63	0.007335849	Abscisic acid-responsive (TB2/DP1, HVA22) family protein	Green ontogenesis	
ppa011581m	4.63	0.000205856		Green ontogenesis	
ppa022514m	4.59	0.036394288	matrix metalloproteinase	Green ontogenesis	
ppa010337m	4.59	0.003010139	NAC domain containing protein 90	Green ontogenesis	
ppa000839m	4.59	0.000131152	glutamate receptor 2.8	Green ontogenesis	
ppa007327m	4.59	0.004256977	GroES-like zinc-binding alcohol dehydrogenase family protein	Green ontogenesis	
ppa011993m	4.59	0.045938424	maternal effect embryo arrest 60	Green ontogenesis	
ppa009084m	4.58	0.019132936	NAD(P)-binding Rossmann-fold superfamily protein	Green ontogenesis	
ppa022770m	4.53	0.018087793	F-box family protein with a domain of unknown function (DUF295)	Green ontogenesis	
ppa015305m	4.52	0.002263417	HVA22-like protein G	Green ontogenesis	
ppb018359m	4.52	0.038000801		Green ontogenesis	
ppa014675m	4.51	0.000944883	heat shock transcription factor B3	Green ontogenesis	
ppa003270m	4.51	0.001343687	cationic amino acid transporter 6	Green ontogenesis	
ppa017982m	4.49	2.65E-05	expansin-like B1	Green ontogenesis	
ppa003065m	4.46	0.000389259	Auxin-responsive GH3 family protein	Green ontogenesis	
ppa021232m	4.45	0.000610249	elicitor-activated gene 3-1	Green ontogenesis	Secondary Metabolism / Phenylpropanoids - Biotic Stress / Secondary metabolism involved into stress
ppa011598m	4.45	0.000138468	nitrate transmembrane transporters	Green ontogenesis	
ppa013267m	4.45	0.019140051		Green ontogenesis	
ppa023775m	4.45	0.008925	indeterminate(ID)-domain 2	Green ontogenesis	
ppa012991m	4.43	0.00383498	pathogenesis-related 4	Green ontogenesis	
ppa022754m	4.42	0.003173116	syntaxin of plants 121	Green ontogenesis	
ppa026291m	4.42	0.03617293	beta-hydroxylase 1	Green ontogenesis	Biotic Stress / Secondary metabolism involved into stress
ppa017934m	4.42	0.036592258	Exostosin family protein	Green ontogenesis	
ppa017278m	4.42	0.001051317		Green ontogenesis	
ppa016729m	4.41	0.000670637		Green ontogenesis	
ppa009530m	4.37	0.002742652	NAC-like, activated by AP3/PI	Green ontogenesis	
ppa013329m	4.37	0.00299488		Green ontogenesis	
ppa020043m	4.36	0.000446946	GRAS family transcription factor	Green ontogenesis	
ppa005406m	4.35	0.000117715	UDP-Glycosyltransferase superfamily protein	Green ontogenesis	
ppa012646m	4.35	0.000333876	MLP-like protein 423	Green ontogenesis	
ppa017530m	4.33	0.001507339	sulfur E2	Green ontogenesis	
ppa001718m	4.32	0.004307133	beta-xylosidase 1	Green ontogenesis	
ppa011660m	4.31	0.000123207	RAB GTPase homolog 1A	Green ontogenesis	
ppa012458m	4.30	0.009287624		Green ontogenesis	
ppa010287m	4.29	0.001832929	Aluminium induced protein with YGL and LRDR motifs	Green ontogenesis	
ppa012496m	4.28	0.003420424	Uridine diphosphate glycosyltransferase 74E2	Green ontogenesis	
ppa006365m	4.25	0.001356283	RING/U-box superfamily protein	Green ontogenesis	
ppa003417m	4.25	0.022704019	inositol transporter 4	Green ontogenesis	
ppa004390m	4.25	0.001268447	cytochrome P450, family 71, subfamily B, polypeptide 36	Green ontogenesis	
ppa017927m	4.24	9.30E-05	glutamate receptor 2.7	Green ontogenesis	
ppa023006m	4.24	0.037641947	Calcium-binding EF-hand family protein	Green ontogenesis	
ppb016539m	4.23	0.003293067	UDP-glycosyltransferase 73B4	Green ontogenesis	
ppa016459m	4.22	0.021471001	WRKY DNA-binding protein 75	Green ontogenesis	
ppa016466m	4.21	0.000155769	cytochrome P450, family 87, subfamily A, polypeptide 2	Green ontogenesis	
ppa005475m	4.21	0.004813285	Uridine diphosphate glycosyltransferase 74E2	Green ontogenesis	
ppa019486m	4.21	0.000745095		Green ontogenesis	
ppa009647m	4.20	0.001696612	WRKY DNA-binding protein 65	Green ontogenesis	
ppa021960m	4.20	0.000442104	Late embryogenesis abundant (LEA) hydroxyproline-rich glycoprc	Green ontogenesis	
ppa017028m	4.20	0.000233137		Green ontogenesis	
ppa013181m	4.20	0.000205856		Green ontogenesis	
ppa016206m	4.19	0.000658788	UDP-Glycosyltransferase superfamily protein	Green ontogenesis	
ppa012062m	4.19	0.001163111	Disease resistance-responsive (dirigent-like protein) family protei	Green ontogenesis	Biotic Stress / PR-proteins
ppa016301m	4.18	0.000351911	Copper amine oxidase family protein	Green ontogenesis	
ppa021414m	4.17	3.18E-05	UDP-glucosyl transferase 85A2	Green ontogenesis	
ppb016872m	4.16	0.006232608	UDP-glucosyl transferase 85A2	Green ontogenesis	
ppa015580m	4.15	0.029987245	galactinol synthase 1	Green ontogenesis	
ppa017380m	4.15	0.029466408	Late embryogenesis abundant (LEA) hydroxyproline-rich glycoprc	Green ontogenesis	
ppa024933m	4.15	0.004511084	ATPase E1-E2 type family protein / haloacid dehalogenase-like h	Green ontogenesis	
ppa022758m	4.13	0.006758966	WRKY family transcription factor	Green ontogenesis	
ppa021408m	4.13	0.001136153	calmodulin-like 41	Green ontogenesis	
ppa010181m	4.13	0.041790001	Nodulin MtN3 family protein	Green ontogenesis	
ppa021018m	4.13	0.036895494	Cupredoxin superfamily protein	Green ontogenesis	
ppa018589m	4.13	0.004622112	Serine protease inhibitor, potato inhibitor I-type family protein	Green ontogenesis	
ppa025969m	4.12	0.024068719	alpha/beta-Hydrolases superfamily protein	Green ontogenesis	
ppa002334m	4.12	0.022026088	Glycosyl hydrolases family 32 protein	Green ontogenesis	
ppa003257m	4.12	0.007553276		Green ontogenesis	
ppa023887m	4.12	0.009836353	S-adenosyl-L-methionine-dependent methyltransferases superfar	Green ontogenesis	
ppa009924m	4.10	0.00375265	salt tolerance zinc finger	Green ontogenesis	
ppa014321m	4.10	0.006694596	cryptdin protein-related	Green ontogenesis	
ppa020834m	4.10	0.018985797	Transducin/WD40 repeat-like superfamily protein	Green ontogenesis	
ppa012642m	4.10	0.000205856	MLP-like protein 423	Green ontogenesis	
ppa016708m	4.10	0.043488749	MYB-like 102	Green ontogenesis	
ppa002973m	4.09	0.000386778	Protein kinase family protein	Green ontogenesis	
ppa022472m	4.09	0.000278126	2-oxoglutarate (2OG) and Fe(II)-dependent oxygenase superfami	Green ontogenesis	
ppa008738m	4.07	0.000136527	RING/U-box superfamily protein	Green ontogenesis	
ppa003358m	4.07	0.000152648	AZA-guanine resistant1	Green ontogenesis	
ppa011804m	4.05	0.044736281	heat shock transcription factor B2A	Green ontogenesis	
ppa023127m	4.02	1.80E-05	S-locus lectin protein kinase family protein	Green ontogenesis	
ppa025532m	4.02	0.001544541	bidirectional amino acid transporter 1	Green ontogenesis	
ppa024396m	4.01	0.000412683	D-mannose binding lectin protein with Apple-like carbohydrate-bir	Green ontogenesis	
ppa008905m	4.01	0.000249397	NAD(P)-linked oxidoreductase superfamily protein	Green ontogenesis	
ppa005172m	4.00	0.03521989	alpha/beta-Hydrolases superfamily protein	Green ontogenesis	
ppa014245m	3.98	0.001302322		Green ontogenesis	
ppa002471m	3.97	0.000944883	O-fucosyltransferase family protein	Green ontogenesis	
ppa010728m	3.97	0.000670637		Green ontogenesis	
ppa023067m	3.97	0.014085458	BON association protein 2	Green ontogenesis	
ppa012632m	3.96	3.18E-05	MLP-like protein 423	Green ontogenesis	
ppa015518m	3.96	0.000963589	2-oxoglutarate (2OG) and Fe(II)-dependent oxygenase superfami	Green ontogenesis	
ppa026171m	3.96	0.000132814	glutathione S-transferase tau 7	Green ontogenesis	Biotic Stress / Glutathione-S-Transferase
ppa017192m	3.95	0.000527772	glutathione S-transferase tau 7	Green ontogenesis	Biotic Stress / Glutathione-S-Transferase
ppa012649m	3.95	0.000182969	MLP-like protein 423	Green ontogenesis	
ppa002979m	3.94	0.000411192	wall associated kinase-like 4	Green ontogenesis	
ppa018138m	3.93	0.002673072	Late embryogenesis abundant (LEA) hydroxyproline-rich glycoprc	Green ontogenesis	
ppa007883m	3.93	0.040977325	NAC domain containing protein 100	Green ontogenesis	
ppa012928m	3.90	0.000134629	glutathione S-transferase tau 7	Green ontogenesis	Biotic Stress / Glutathione-S-Transferase
ppa018675m	3.88	0.00075669	2-oxoglutarate (2OG) and Fe(II)-dependent oxygenase superfami	Green ontogenesis	
ppa026001m	3.87	0.001321857	2-oxoglutarate (2OG) and Fe(II)-dependent oxygenase superfami	Green ontogenesis	
ppa004496m	3.85	0.000117885	cytochrome P450, family 714, subfamily A, polypeptide 1	Green ontogenesis	
ppa015182m	3.85	0.003101229	glutathione S-transferase tau 7	Green ontogenesis	Biotic Stress / Glutathione-S-Transferase
ppa026587m	3.85	0.007990351	Chlorophyll A-B binding family protein	Green ontogenesis	
ppa015203m	3.84	0.007096582	wall associated kinase 5	Green ontogenesis	
ppa025529m	3.84	0.042027966	phospholipase A 2A	Green ontogenesis	
ppa007445m	3.83	0.003322809	NAC domain containing protein 47	Green ontogenesis	
ppa013276m	3.83	0.000745095	sigma factor binding protein 1	Green ontogenesis	
ppa006359m	3.82	0.03615506	Plant protein of unknown function (DUF828)	Green ontogenesis	
ppa011835m	3.82	0.001835024	Late embryogenesis abundant (LEA) hydroxyproline-rich glycoprc	Green ontogenesis	
ppa007840m	3.81	0.001300747	2-oxoglutarate (2OG) and Fe(II)-dependent oxygenase superfami	Green ontogenesis	
ppa011688m	3.81	0.030771591		Green ontogenesis	
ppa022164m	3.81	0.011099807	cryptdin protein-related	Green ontogenesis	
ppa010053m	3.81	0.003449834		Green ontogenesis	
ppa011191m	3.80	0.036088517		Green ontogenesis	
ppa011306m	3.79	0.011506168	Acyl-CoA N-acyltransferases (NAT) superfamily protein	Green ontogenesis	
ppa011701m	3.79	0.000724297	Calcium-binding EF-hand family protein	Green ontogenesis	

ppa026609m	3.78	0.002816563	*Ankyrin repeat family protein*	Green ontogenesis	
ppa014729m	3.78	0.0012693	*UDP-glucosyl transferase 73C1*	Green ontogenesis	
ppa015248m	3.77	0.001987609	*ZCF37*	Green ontogenesis	
ppa016380m	3.76	0.001194915	*UDP-glucosyl transferase 85A5*	Green ontogenesis	
ppa016853m	3.76	0.001152163	*Major facilitator superfamily protein*	Green ontogenesis	
ppa004465m	3.75	0.000168147	*Major facilitator superfamily protein*	Green ontogenesis	
ppa021294m	3.75	0.002108748	*TOXICOS EN LEVADURA 4*	Green ontogenesis	
ppa022672m	3.73	0.000937966	*2-oxoglutarate (2OG) and Fe(II)-dependent oxygenase superfami*	Green ontogenesis	
ppb012927m	3.72	0.000188323	*MLP-like protein 423*	Green ontogenesis	
ppa006741m	3.72	0.000177936		Green ontogenesis	
ppa002895m	3.72	0.000784343	*cysteine-rich RLK (RECEPTOR-like protein kinase) 3*	Green ontogenesis	
ppa021944m	3.72	0.01771093		Green ontogenesis	
ppa025431m	3.71	0.002386922	*calmodulin-like 38*	Green ontogenesis	
ppa025882m	3.71	0.00111901	*2-oxoglutarate (2OG) and Fe(II)-dependent oxygenase superfami*	Green ontogenesis	Secondary Metabolism / Dihydroflavonols / Flavonols-Isoflavonoid - Biotic Stress / Secondary metabolism involved into stress
ppa015136m	3.70	0.006570654	*DVL family protein*	Green ontogenesis	
ppa015483m	3.69	0.005236428		Green ontogenesis	
ppa021625m	3.69	0.0017335	*2-oxoglutarate (2OG) and Fe(II)-dependent oxygenase superfami*	Green ontogenesis	
ppa024488m	3.68	0.015296669	*Seven transmembrane MLO family protein*	Green ontogenesis	
ppa023786m	3.68	0.008827818	*cytochrome P450, family 81, subfamily D, polypeptide 8*	Green ontogenesis	
ppa002615m	3.67	0.004695941	*cysteine-rich RLK (RECEPTOR-like protein kinase) 6*	Green ontogenesis	
ppa022085m	3.67	0.003110718	*bidirectional amino acid transporter 1*	Green ontogenesis	
ppa022234m	3.66	0.020551654	*Predicted AT-hook DNA-binding family protein*	Green ontogenesis	
ppa025263m	3.66	0.001928443	*NAC domain containing protein 42*	Green ontogenesis	
ppa004009m	3.66	0.011484731	*WRKY DNA-binding protein 33*	Green ontogenesis	
ppa006816m	3.66	0.004983547	*NAD(P)-binding Rossmann-fold superfamily protein*	Green ontogenesis	
ppa004341m	3.65	0.000373537	*cytochrome P450, family 714, subfamily A, polypeptide 1*	Green ontogenesis	
ppa005290m	3.65	0.00063489	*UDP-glucosyl transferase 71B6*	Green ontogenesis	
ppb024583m	3.65	0.048271664	*glutamate receptor 2.7*	Green ontogenesis	
ppa013574m	3.65	0.010473204		Green ontogenesis	
ppa007811m	3.64	0.002778435	*2-oxoglutarate (2OG) and Fe(II)-dependent oxygenase superfami*	Green ontogenesis	
ppa018454m	3.64	0.000123207	*UDP-glucosyl transferase 73C1*	Green ontogenesis	
ppa018582m	3.63	0.003959515	*2-oxoglutarate (2OG) and Fe(II)-dependent oxygenase superfami*	Green ontogenesis	
ppa025666m	3.63	0.000144188	*cytochrome P450, family 716, subfamily A, polypeptide 1*	Green ontogenesis	
ppa026799m	3.63	6.59E-06	*gibberellin 2-oxidase 8*	Green ontogenesis	
ppa005447m	3.63	0.00529853	*UDP-Glycosyltransferase superfamily protein*	Green ontogenesis	
ppa012761m	3.63	0.006018153	*VQ motif-containing protein*	Green ontogenesis	
ppa005092m	3.62	0.030671702	*alanine:glyoxylate aminotransferase 3*	Green ontogenesis	
ppa018343m	3.61	1.36E-05	*UDP-glucosyl transferase 85A2*	Green ontogenesis	
ppa003663m	3.61	0.000316677	*laccase 14*	Green ontogenesis	Biotic Stress / Secondary metabolism involved into stress
ppb010973m	3.60	0.008098273	*2-oxoglutarate (2OG) and Fe(II)-dependent oxygenase superfami*	Green ontogenesis	
ppa005408m	3.59	0.00049866	*Transmembrane amino acid transporter family protein*	Green ontogenesis	
ppa026957m	3.59	0.002280326	*RING/U-box superfamily protein*	Green ontogenesis	
ppa016223m	3.59	0.004987322	*P-loop containing nucleoside triphosphate hydrolases superfamily*	Green ontogenesis	
ppa008521m	3.58	0.001797555	*galactinol synthase 2*	Green ontogenesis	
ppa018361m	3.57	0.025710469	*Wall-associated kinase family protein*	Green ontogenesis	
ppa023494m	3.57	0.001398452	*F-box family protein*	Green ontogenesis	
ppa017430m	3.57	0.020960916	*Eukaryotic aspartyl protease family protein*	Green ontogenesis	
ppa021447m	3.57	0.001241497	*O-methyltransferase 1*	Green ontogenesis	
ppa026315m	3.56	0.000547379	*myb domain protein 63*	Green ontogenesis	
ppa011060m	3.56	0.005886816	*salt tolerance zinc finger*	Green ontogenesis	
ppa020792m	3.55	0.016158519	*ARM repeat superfamily protein*	Green ontogenesis	
ppa016514m	3.55	0.000540186	*beta HLH protein 93*	Green ontogenesis	
ppb010518m	3.55	0.00317925	*MLP-like protein 423*	Green ontogenesis	
ppa009992m	3.54	0.019368204	*Receptor-like protein kinase-related family protein*	Green ontogenesis	
ppa022653m	3.54	0.001954667	*GRAS family transcription factor*	Green ontogenesis	
ppa021195m	3.54	0.00063489	*WRKY DNA-binding protein 61*	Green ontogenesis	
ppb011093m	3.53	0.027779584	*arabinogalactan protein 1*	Green ontogenesis	
ppa005470m	3.53	0.006545361	*methionine gamma-lyase*	Green ontogenesis	
ppa014173m	3.53	0.004940232		Green ontogenesis	
ppa024674m	3.53	0.003959515	*2-oxoglutarate (2OG) and Fe(II)-dependent oxygenase superfami*	Green ontogenesis	
ppa011142m	3.52	0.001281064	*glutathione S-transferase tau 7*	Green ontogenesis	Biotic Stress / Glutathione-S-Transferase
ppa024619m	3.52	7.05E-06	*methyl esterase 10*	Green ontogenesis	
ppa005277m	3.51	0.000724297	*UDP-glucosyl transferase 71B6*	Green ontogenesis	
ppa021111m	3.51	0.004983547	*NAD(P)-binding Rossmann-fold superfamily protein*	Green ontogenesis	
ppa005059m	3.51	0.000822458	*cytochrome P450, family 707, subfamily A, polypeptide 1*	Green ontogenesis	
ppa002830m	3.50	0.00186233	*FAD-dependent oxidoreductase family protein*	Green ontogenesis	
ppa008474m	3.50	0.000230389	*polygalacturonase inhibiting protein 1*	Green ontogenesis	
ppa025103m	3.49	0.048942451	*NAC domain containing protein 90*	Green ontogenesis	
ppa006448m	3.49	0.021063312	*plant U-box 29*	Green ontogenesis	
ppa001821m	3.48	0.006871446	*alpha/beta-Hydrolases superfamily protein*	Green ontogenesis	
ppa003424m	3.48	0.000747704	*O-fucosyltransferase family protein*	Green ontogenesis	
ppa018490m	3.46	0.003850521	*Plant protein 1589 of unknown function*	Green ontogenesis	
ppa012629m	3.46	0.000188323	*MLP-like protein 423*	Green ontogenesis	
ppa009330m	3.45	0.002421208	*NAD(P)-binding Rossmann-fold superfamily protein*	Green ontogenesis	
ppa025974m	3.45	0.042846056	*cytochrome P450, family 705, subfamily A, polypeptide 27*	Green ontogenesis	
ppa1027203m	3.44	0.027481735		Green ontogenesis	
ppa018914m	3.44	0.000331596	*phosphate transporter 3;2*	Green ontogenesis	
ppa014958m	3.44	0.000397282	*glutathione S-transferase tau 7*	Green ontogenesis	Biotic Stress / Glutathione-S-Transferase
ppa011672m	3.43	0.006391302		Green ontogenesis	
ppa008358m	3.43	0.001334446	*Oxidoreductase, zinc-binding dehydrogenase family protein*	Green ontogenesis	
ppa016992m	3.43	0.04275326	*serine carboxypeptidase-like 2*	Green ontogenesis	
ppa009438m	3.43	0.014218821	*NAC (No Apical Meristem) domain transcriptional regulator super*	Green ontogenesis	
ppa008125m	3.42	0.017922437	*gibberellin 2-oxidase 8*	Green ontogenesis	
ppa020127m	3.42	0.000447364	*Calcium-dependent lipid-binding (CaLB domain) family protein*	Green ontogenesis	
ppa026707m	3.42	0.001377353	*cytochrome P450, family 714, subfamily A, polypeptide 2*	Green ontogenesis	
ppa013848m	3.41	0.001087016		Green ontogenesis	
ppa008310m	3.41	0.007642683	*gibberellin 2-oxidase 6*	Green ontogenesis	
ppa000961m	3.41	0.000561887	*NB-ARC domain-containing disease resistance protein*	Green ontogenesis	
ppa003583m	3.40	0.000168147	*Laccase/Diphenol oxidase family protein*	Green ontogenesis	Biotic Stress / Secondary metabolism involved into stress
ppa012261m	3.40	0.000917304	*Late embryogenesis abundant (LEA) hydroxyproline-rich glycopro*	Green ontogenesis	
ppa001181m	3.39	0.000106669	*inosine-uridine preferring nucleoside hydrolase family protein*	Green ontogenesis	
ppa024342m	3.39	0.000155769	*S-domain-1 29*	Green ontogenesis	
ppa000853m	3.39	0.001129143	*Clp ATPase*	Green ontogenesis	
ppa021485m	3.39	0.00991855	*Late embryogenesis abundant (LEA) hydroxyproline-rich glycopro*	Green ontogenesis	
ppa024281m	3.39	0.000832133	*glutathione S-transferase tau 7*	Green ontogenesis	Biotic Stress / Glutathione-S-Transferase
ppa015115m	3.38	0.008926816	*blue-copper-binding protein*	Green ontogenesis	
ppa005662m	3.37	0.001044603	*lysine histidine transporter 1*	Green ontogenesis	
ppa003547m	3.36	0.003861359	*Eukaryotic aspartyl protease family protein*	Green ontogenesis	
ppa007059m	3.36	0.000872438	*Regulator of Vps4 activity in the MVB pathway protein*	Green ontogenesis	
ppa004530m	3.36	0.000563789	*nitrate transporter2.5*	Green ontogenesis	
ppa008603m	3.36	7.21E-05	*NAD(P)-linked oxidoreductase superfamily protein*	Green ontogenesis	
ppa011230m	3.35	0.000937966	*Peroxisomal membrane 22 kDa (Mpv17/PMP22) family protein*	Green ontogenesis	
ppa010361m	3.35	0.025780908	*2-oxoglutarate (2OG) and Fe(II)-dependent oxygenase superfami*	Green ontogenesis	
ppa021244m	3.35	0.00016382	*Late embryogenesis abundant (LEA) hydroxyproline-rich glycopro*	Green ontogenesis	
ppa003209m	3.34	0.000248234	*peptide transporter 3*	Green ontogenesis	
ppa022051m	3.34	0.015436594	*Uncharacterised protein family (UPF0114)*	Green ontogenesis	
ppa021840m	3.34	0.047334816	*Leucine-rich receptor-like protein kinase family protein*	Green ontogenesis	
ppa008801m	3.33	0.002010855	*NAC domain containing protein 1*	Green ontogenesis	
ppa022983m	3.33	0.01544422	*Late embryogenesis abundant (LEA) hydroxyproline-rich glycopro*	Green ontogenesis	
ppa023100m	3.33	0.001384146	*cytochrome P450, family 72, subfamily A, polypeptide 9*	Green ontogenesis	
ppa024291m	3.31	0.005576065	*NAD(P)-linked oxidoreductase superfamily protein*	Green ontogenesis	Biotic Stress / Secondary metabolism involved into stress
ppa020068m	3.31	0.015518977		Green ontogenesis	
ppa003580m	3.31	0.000423154	*laccase 14*	Green ontogenesis	Biotic Stress / Secondary metabolism involved into stress
ppa025788m	3.30	0.001136281	*Protein of unknown function (DUF594)*	Green ontogenesis	
ppa008174m	3.30	0.000197511	*farnesyl diphosphate synthase 1*	Green ontogenesis	Biotic Stress / Secondary metabolism involved into stress
ppa018075m	3.30	0.000620316	*WRKY family transcription factor*	Green ontogenesis	
ppa014020m	3.30	0.001248979		Green ontogenesis	
ppa018059m	3.30	0.000315635	*UDP-glucosyl transferase 73B3*	Green ontogenesis	
ppa024359m	3.30	0.018933211	*ARM repeat superfamily protein*	Green ontogenesis	
ppa012789m	3.29	0.017791763		Green ontogenesis	
ppa005520m	3.29	0.042885806	*UDP-Glycosyltransferase superfamily protein*	Green ontogenesis	
ppa018910m	3.29	0.000124561	*ARM repeat superfamily protein*	Green ontogenesis	
ppa000941m	3.29	0.001235387	*Protein kinase family protein with leucine-rich repeat domain*	Green ontogenesis	
ppa021949m	3.29	0.008827818	*VQ motif-containing protein*	Green ontogenesis	
ppa024941m	3.28	0.008036829	*homolog of carrot EP3-3 chitinase*	Green ontogenesis	
ppa010729m	3.28	0.003014256	*pathogenesis-related family protein*	Green ontogenesis	
ppa026262m	3.27	0.00115899	*HXXXD-type acyl-transferase family protein*	Green ontogenesis	Secondary Metabolism / Phenylpropanoids - Biotic Stress / Secondary metabolism involved into stress
ppa017452m	3.26	0.024618403	*cinnamyl alcohol dehydrogenase 9*	Green ontogenesis	Secondary Metabolism / Phenylpropanoids - Biotic Stress / Secondary metabolism involved into stress
ppa025240m	3.25	0.000217016	*PYR1-like 4*	Green ontogenesis	
ppa023736m	3.25	0.019006612	*Integrase-type DNA-binding superfamily protein*	Green ontogenesis	
ppa023229m	3.24	0.00345156	*Major facilitator superfamily protein*	Green ontogenesis	
ppa013758m	3.24	0.00299246	*CLAVATA3/ESR-RELATED 43*	Green ontogenesis	
ppa010059m	3.23	0.017202282	*RING/U-box superfamily protein*	Green ontogenesis	
ppa016848m	3.22	0.00451668	*polyubiquitin 10*	Green ontogenesis	
ppa007512m	3.22	0.006203574	*Aldolase-type TIM barrel family protein*	Green ontogenesis	
ppa026475m	3.22	0.008808109	*2-oxoglutarate (2OG) and Fe(II)-dependent oxygenase superfami*	Green ontogenesis	
ppa007685m	3.22	0.012395577	*xyloglucan endotransglucosylase/hydrolase 30*	Green ontogenesis	
ppa001535m	3.22	0.000907834	*sucrose synthase 4*	Green ontogenesis	
ppa021861m	3.21	2.24E-05	*carboxyesterase 13*	Green ontogenesis	
ppa009944m	3.21	0.001467512	*NAD(P)-binding Rossmann-fold superfamily protein*	Green ontogenesis	
ppa007374m	3.20	0.002151337	*GroES-like zinc-binding alcohol dehydrogenase family protein*	Green ontogenesis	
ppa023716m	3.20	0.00350487	*glutathione S-transferase TAU 8*	Green ontogenesis	Biotic Stress / Glutathione-S-Transferase
ppa026144m	3.20	0.009985452	*formin 8*	Green ontogenesis	
ppa023543m	3.19	0.006645545	*Plant calmodulin-binding protein-related*	Green ontogenesis	
ppa019963m	3.18	0.041348258		Green ontogenesis	
ppa014306m	3.18	0.017774711		Green ontogenesis	
ppa018534m	3.18	0.012384628		Green ontogenesis	
ppa012627m	3.18	0.003811017	*MLP-like protein 423*	Green ontogenesis	

ppa008956m	3.17	0.000136506	Coatomer, beta\' subunit	Green ontogenesis	
ppa012907m	3.17	0.003181237	calmodulin-like 11	Green ontogenesis	
ppa015480m	3.17	0.005660005	WRKY DNA-binding protein 51	Green ontogenesis	
ppa018260m	3.16	0.001117902	polyubiquitin 10	Green ontogenesis	
ppb009629m	3.15	0.049239168		Green ontogenesis	
ppa023469m	3.15	3.18E-05	alpha/beta-Hydrolases superfamily protein	Green ontogenesis	
ppb021358m	3.15	0.004045575	UDP-glucosyl transferase 85A3	Green ontogenesis	
ppa009788m	3.15	4.51E-05	non-yellowing 1	Green ontogenesis	
ppa023881m	3.13	0.000916598	phytosulfokine 4 precursor	Green ontogenesis	
ppa025469m	3.13	0.004680894	Calmodulin binding protein-like	Green ontogenesis	
ppa022370m	3.13	0.000641204		Green ontogenesis	
ppa011666m	3.12	0.000508993	Late embryogenesis abundant (LEA) hydroxyproline-rich glycopro	Green ontogenesis	
ppa003639m	3.12	0.002777424	Plant invertase/pectin methylesterase inhibitor superfamily	Green ontogenesis	
ppa023893m	3.12	0.002306433	Disease resistance-responsive (dirigent-like protein) family protei	Green ontogenesis	Biotic Stress / PR-proteins
ppa009711m	3.12	0.024237945	atypical CYS HIS rich thioredoxin 5	Green ontogenesis	
ppa021128m	3.11	0.000389194	glutamate receptor 2.9	Green ontogenesis	
ppa010822m	3.11	6.59E-06	K-box region and MADS-box transcription factor family protein	Green ontogenesis	
ppa014274m	3.11	0.007814623		Green ontogenesis	
ppa017661m	3.10	0.000577195	Coatomer, beta\' subunit	Green ontogenesis	
ppa014940m	3.10	0.006748878	2-oxoglutarate (2OG) and Fe(II)-dependent oxygenase superfami	Green ontogenesis	
ppa016693m	3.10	0.028825337	mitogen-activated protein kinase kinase kinase 14	Green ontogenesis	
ppa022752m	3.10	0.000373537	Cytochrome P450 superfamily protein	Green ontogenesis	
ppa003269m	3.10	0.000462685	Calmodulin-binding protein	Green ontogenesis	
ppb017968m	3.10	0.00100309	cytochrome P450, family 709, subfamily B, polypeptide 1	Green ontogenesis	
ppa003812m	3.08	9.56E-05	chloroplast beta-amylase	Green ontogenesis	
ppa018265m	3.07	0.003123557	RING/U-box superfamily protein	Green ontogenesis	
ppa020333m	3.07	0.036274445	Uncharacterised conserved protein UCP015417, vWA	Green ontogenesis	
ppa022054m	3.06	0.004131338	gibberellin 2-oxidase 8	Green ontogenesis	
ppa016800m	3.06	0.001327599	cytochrome P450, family 72, subfamily A, polypeptide 9	Green ontogenesis	
ppa009163m	3.05	0.002224418	cytidine deaminase 1	Green ontogenesis	
ppa022765m	3.05	0.005518555	calmodulin-binding family protein	Green ontogenesis	
ppa001810m	3.05	0.002042876	Terpenoid cyclases family protein	Green ontogenesis	Biotic Stress / Secondary metabolism involved into stress
ppa005203m	3.05	0.000230428	F-box family protein	Green ontogenesis	
ppa014659m	3.05	1.76E-05	glutamate receptor 2.7	Green ontogenesis	
ppa022987m	3.04	0.0085828	arogenate dehydrogenase	Green ontogenesis	
ppa019104m	3.04	3.18E-05	methyl esterase 3	Green ontogenesis	
ppa004722m	3.03	3.27E-05	Methylenetetrahydrofolate reductase family protein	Green ontogenesis	
ppa013078m	3.02	0.003443498		Green ontogenesis	
ppa022572m	3.02	0.00782869	wall-associated kinase 2	Green ontogenesis	
ppa002734m	3.01	0.012122178	calmodulin-domain protein kinase cdpk isoform 2	Green ontogenesis	
ppa011132m	3.01	0.007792366	Protein of unknown function (DUF567)	Green ontogenesis	
ppa007923m	3.01	0.000399943	UDP-N-acetylglucosamine (UAA) transporter family	Green ontogenesis	
ppa007918m	3.01	0.001823737	phosphate transporter 3;2	Green ontogenesis	
ppa017714m	3.00	0.005880251	Protein of unknown function (DUF677)	Green ontogenesis	
ppa026931m	3.00	3.27E-05	NAD(P)-binding Rossmann-fold superfamily protein	Green ontogenesis	
ppa010429m	3.00	0.005376196	cold regulated gene 27	Green ontogenesis	
ppa024840m	3.00	9.98E-05	HCO3- transporter family	Green ontogenesis	
ppa004811m	2.99	0.000646703	alanine:glyoxylate aminotransferase 2	Green ontogenesis	
ppa022043m	2.99	0.00040677	NAC domain containing protein 42	Green ontogenesis	
ppa012272m	2.98	3.18E-05	Chaperone DnaJ-domain superfamily protein	Green ontogenesis	
ppa018538m	2.98	0.021612228	alpha/beta-Hydrolases superfamily protein	Green ontogenesis	
ppa004319m	2.98	0.004265899	cytochrome P450, family 72, subfamily A, polypeptide 9	Green ontogenesis	
ppb020734m	2.98	0.017608865	disease resistance family protein / LRR family protein	Green ontogenesis	
ppa002513m	2.97	0.001145798	S-adenosyl-L-methionine-dependent methyltransferases superfar	Green ontogenesis	
ppa014817m	2.96	0.004950164	alternative oxidase 1A	Green ontogenesis	
ppa012452m	2.96	0.015131782		Green ontogenesis	
ppa016347m	2.94	0.005613719	Thioredoxin superfamily protein	Green ontogenesis	
ppa007009m	2.94	0.005904664	phospholipase A 2A	Green ontogenesis	
ppa014656m	2.94	0.002109983	cytochrome P450, family 76, subfamily C, polypeptide 3	Green ontogenesis	
ppa008902m	2.94	0.007817331	carboxyesterase 13	Green ontogenesis	
ppa019724m	2.94	0.017018242	Concanavalin A-like lectin protein kinase family protein	Green ontogenesis	
ppa021699m	2.93	0.037892806	Ankyrin repeat family protein	Green ontogenesis	
ppa021909m	2.93	0.027779584	CRINKLY4 related 4	Green ontogenesis	
ppa002036m	2.92	0.000526087	multifunctional protein 2	Green ontogenesis	
ppa027031m	2.90	0.006690754	DNAse I-like superfamily protein	Green ontogenesis	
ppa001952m	2.90	3.84E-05	cellulose synthase like E1	Green ontogenesis	
ppa002700m	2.90	0.002111027	purple acid phosphatase 27	Green ontogenesis	
ppa019833m	2.90	0.004983547	Basic-leucine zipper (bZIP) transcription factor family protein	Green ontogenesis	
ppa006485m	2.90	0.021181742	mitogen-activated protein kinase kinase kinase 15	Green ontogenesis	
ppa011008m	2.89	1.76E-05	DNAJ heat shock N-terminal domain-containing protein	Green ontogenesis	
ppa011401m	2.89	0.023156517		Green ontogenesis	
ppa025554m	2.89	0.001784963	alpha/beta-Hydrolases superfamily protein	Green ontogenesis	
ppa010479m	2.89	0.01334709	Pathogenesis-related thaumatin superfamily protein	Green ontogenesis	
ppa024049m	2.89	0.020095537	brassinosteroid-responsive RING-H2	Green ontogenesis	
ppa003437m	2.89	0.026746314	Seven transmembrane MLO family protein	Green ontogenesis	
ppa004957m	2.89	0.000237249	UDP-Glycosyltransferase superfamily protein	Green ontogenesis	
ppa004523m	2.89	0.000282095	beta-glucosidase 45	Green ontogenesis	
ppa011082m	2.88	0.009016489		Green ontogenesis	
ppa004221m	2.88	0.00027599	pheophytinase	Green ontogenesis	
ppa019288m	2.87	0.004558892	Ankyrin repeat family protein	Green ontogenesis	
ppa019739m	2.87	0.041473711	VQ motif-containing protein	Green ontogenesis	
ppa010471m	2.87	0.025228749	Pathogenesis-related thaumatin superfamily protein	Green ontogenesis	
ppa027036m	2.87	0.001543593		Green ontogenesis	
ppa013811m	2.87	0.00384776	peptidoglycan-binding LysM domain-containing protein	Green ontogenesis	
ppa006909m	2.86	0.000841883	WRKY DNA-binding protein 48	Green ontogenesis	
ppa023256m	2.85	0.010076283	WRKY DNA-binding protein 21	Green ontogenesis	
ppa004004m	2.85	0.000337882	phosphate transporter 1;5	Green ontogenesis	
ppa002863m	2.84	4.60E-05	flavin-binding, kelch repeat, f box 1	Green ontogenesis	
ppa018340m	2.84	0.003443498	Glycosyl hydrolase family protein with chitinase insertion domain	Green ontogenesis	
ppa005384m	2.84	0.000313173	Regulator of chromosome condensation (RCC1) family protein	Green ontogenesis	
ppa008079m	2.84	0.012771438	Esterase/lipase/thioesterase family protein	Green ontogenesis	
ppa022996m	2.84	0.015061871	Integrase-type DNA-binding superfamily protein	Green ontogenesis	
ppa002309m	2.84	0.02570429	cysteine-rich RLK (RECEPTOR-like protein kinase) 29	Green ontogenesis	
ppa005368m	2.84	0.000110138	serine carboxypeptidase-like 34	Green ontogenesis	
ppa020396m	2.83	0.007045925		Green ontogenesis	
ppa026236m	2.83	0.008511435		Green ontogenesis	
ppa008772m	2.83	0.016131431	Integrase-type DNA-binding superfamily protein	Green ontogenesis	
ppa006969m	2.83	0.036366886		Green ontogenesis	
ppa022943m	2.82	0.002424648		Green ontogenesis	
ppa003470m	2.82	0.001390383	Glycosyl hydrolases family 32 protein	Green ontogenesis	
ppa010473m	2.82	0.009337399	Pathogenesis-related thaumatin superfamily protein	Green ontogenesis	
ppa021540m	2.81	0.00317328	Homeodomain-like superfamily protein	Green ontogenesis	
ppa000440m	2.81	0.010234948	RAD3-like DNA-binding helicase protein	Green ontogenesis	
ppa024400m	2.80	0.000168886	detoxifying efflux carrier 35	Green ontogenesis	
ppa011221m	2.80	0.01281596	homeobox 7	Green ontogenesis	
ppa025302m	2.79	0.007033807	MLP-like protein 423	Green ontogenesis	
ppa010647m	2.79	0.017870965	homeobox 7	Green ontogenesis	
ppa026623m	2.78	0.000831342	carboxyesterase 20	Green ontogenesis	
ppa019006m	2.78	0.004256977	UDP-Glycosyltransferase superfamily protein	Green ontogenesis	
ppa019189m	2.78	0.015382116		Green ontogenesis	
ppa002831m	2.77	0.009629637	Leucine-rich repeat protein kinase family protein	Green ontogenesis	
ppa025895m	2.77	0.001659782	Wall-associated kinase family protein	Green ontogenesis	
ppa021727m	2.76	0.024388536	UDP-Glycosyltransferase superfamily protein	Green ontogenesis	
ppa024723m	2.76	0.00800644	RING/U-box superfamily protein	Green ontogenesis	
ppa004825m	2.76	9.98E-05	UDP-Glycosyltransferase superfamily protein	Green ontogenesis	
ppa006860m	2.76	0.006226707	Protein of unknown function (DUF1262)	Green ontogenesis	
ppa013082m	2.76	0.011564652		Green ontogenesis	
ppa019699m	2.75	0.00013645	UDP-glucosyl transferase 73B3	Green ontogenesis	
ppa005194m	2.74	0.008946766	beta-glucosidase 47	Green ontogenesis	
ppa004463m	2.74	0.002691651	Cytochrome P450 superfamily protein	Green ontogenesis	
ppa017487m	2.74	0.033129793	xyloglucan endotransglucosylase/hydrolase 16	Green ontogenesis	
ppa015245m	2.74	0.048942451	Peroxidase superfamily protein	Green ontogenesis	
ppa022546m	2.74	0.0359871	Acyl-CoA N-acyltransferases (NAT) superfamily protein	Green ontogenesis	
ppa008799m	2.73	0.008873016	2-oxoglutarate (2OG) and Fe(II)-dependent oxygenase superfami	Green ontogenesis	
ppa010522m	2.73	0.028732485	Pathogenesis-related thaumatin superfamily protein	Green ontogenesis	
ppa005514m	2.72	0.021181742	cold-regulated 47	Green ontogenesis	
ppa000420m	2.72	0.007223274	ATPase E1-E2 type family protein / haloacid dehalogenase-like h	Green ontogenesis	
ppa015877m	2.72	0.001285896	S-locus lectin protein kinase family protein	Green ontogenesis	
ppa006697m	2.72	0.011808017	Protein of unknown function (DUF1645)	Green ontogenesis	
ppa024612m	2.72	0.000155194	UDP-Glycosyltransferase superfamily protein	Green ontogenesis	
ppa020190m	2.72	0.008926816	myb domain protein 15	Green ontogenesis	
ppa005770m	2.72	0.002685706	Arabidopsis NAC domain containing protein 87	Green ontogenesis	
ppa1027121m	2.71	0.003135797	glutamate receptor 2.7	Green ontogenesis	
ppa009233m	2.71	7.44E-05	Phenazine biosynthesis PhzC/PhzF protein	Green ontogenesis	
ppa013670m	2.71	0.003114922	Uncharacterised protein family (UPF0041)	Green ontogenesis	
ppa000895m	2.71	0.0051045	Leucine-rich receptor-like protein kinase family protein	Green ontogenesis	
ppa023071m	2.71	0.00048556	UDP-Glycosyltransferase superfamily protein	Green ontogenesis	
ppa005724m	2.71	0.001732361	RING domain ligase1	Green ontogenesis	
ppa004574m	2.70	1.80E-05	dehydroquinate dehydratase, putative / shikimate dehydrogenase	Green ontogenesis	
ppa008877m	2.70	0.005096482	myb domain protein 116	Green ontogenesis	
ppa001730m	2.70	0.003562927	seed imbibition 2	Green ontogenesis	
ppa019847m	2.69	0.001502369	Ankyrin repeat family protein	Green ontogenesis	
ppb009860m	2.69	0.002193119		Green ontogenesis	
ppa008732m	2.69	0.004190735	Duplicated homeodomain-like superfamily protein	Green ontogenesis	
ppa007577m	2.68	0.00139412	NAC domain containing protein 2	Green ontogenesis	
ppa020614m	2.67	0.009143126	Dynein light chain type 1 family protein	Green ontogenesis	
ppa009966m	2.67	0.003464417	myb domain protein 20	Green ontogenesis	
ppa009274m	2.67	0.000577195	heat shock factor 4	Green ontogenesis	

ppa025793m	2.66	0.000944883	Leucine-rich repeat protein kinase family protein	Green ontogenesis	
ppa026640m	2.66	0.001310185	myb domain protein 113	Green ontogenesis	
ppa005683m	2.66	0.000254766	Major facilitator superfamily protein	Green ontogenesis	
ppa025959m	2.66	0.010687395	alpha 1,4-glycosyltransferase family protein	Green ontogenesis	
ppa009624m	2.66	0.03348062	Esterase/lipase/thioesterase family protein	Green ontogenesis	
ppa025081m	2.66	0.001743886	FAD-binding Berberine family protein	Green ontogenesis	
ppa006887m	2.66	0.004544104	methyl esterase 13	Green ontogenesis	
ppa015588m	2.65	0.000117613	Cytochrome P450 superfamily protein	Green ontogenesis	
ppa024555m	2.65	0.022750714	disease resistance family protein / LRR family protein	Green ontogenesis	Biotic Stress / PR-proteins
ppa002243m	2.65	0.002105242	S-locus lectin protein kinase family protein	Green ontogenesis	
ppa011762m	2.65	0.001129143	C2H2-type zinc finger family protein	Green ontogenesis	
ppa008025m	2.64	0.000611311	NAD(P)-linked oxidoreductase superfamily protein	Green ontogenesis	
ppa026417m	2.63	0.002449938	cytochrome P450, family 71, subfamily B, polypeptide 34	Green ontogenesis	
ppa009849m	2.63	0.004379709	catalytic LigB subunit of aromatic ring-opening dioxygenase famil	Green ontogenesis	
ppa013354m	2.63	0.042117208		Green ontogenesis	
ppa003617m	2.63	0.04318744	AMP-dependent synthetase and ligase family protein	Green ontogenesis	
ppa018212m	2.62	0.008400976	acyl-CoA sterol acyl transferase 1	Green ontogenesis	Biotic Stress / Secondary metabolism involved into stress
ppa018629m	2.61	0.000768596	Subtilase family protein	Green ontogenesis	
ppa005448m	2.61	0.046551266	Curculin-like (mannose-binding) lectin family protein	Green ontogenesis	
ppa004988m	2.61	3.84E-05	cytochrome P450, family 716, subfamily A, polypeptide 1	Green ontogenesis	
ppa016741m	2.61	0.007553276	phosphoserine aminotransferase	Green ontogenesis	
ppa004565m	2.61	0.000371535	Cytochrome P450 superfamily protein	Green ontogenesis	
ppa020804m	2.61	5.96E-05	Uridine diphosphate glycosyltransferase 74E2	Green ontogenesis	
ppa019526m	2.61	0.034294015	plant U-box 24	Green ontogenesis	
ppa005330m	2.60	0.003124959	Sulfite exporter TauE/SafE family protein	Green ontogenesis	
ppa012014m	2.60	0.003853005	ethylene responsive element binding factor 2	Green ontogenesis	
ppa012940m	2.60	0.00111901	Late embryogenesis abundant protein	Green ontogenesis	
ppa006013m	2.59	0.001587159	phosphoserine aminotransferase	Green ontogenesis	
ppa026180m	2.59	0.004647936	alpha/beta-Hydrolases superfamily protein	Green ontogenesis	
ppa013511m	2.59	9.82E-05		Green ontogenesis	
ppa017664m	2.58	8.44E-06	S-locus lectin protein kinase family protein	Green ontogenesis	
ppa005728m	2.58	0.016233526	Plant protein of unknown function (DUF247)	Green ontogenesis	
ppa011123m	2.58	2.65E-05	AGAMOUS-like 24	Green ontogenesis	
ppa016689m	2.58	0.043945419	receptor-like protein kinase-related family protein	Green ontogenesis	
ppa005994m	2.58	0.005525221	brassinosteroid-6-oxidase 1	Green ontogenesis	
ppa008529m	2.57	9.56E-05	NAD(P)-linked oxidoreductase superfamily protein	Green ontogenesis	
ppa006776m	2.57	0.003714023	Auxin-responsive family protein	Green ontogenesis	
ppa020089m	2.57	0.000162058	Leucine-rich repeat protein kinase family protein	Green ontogenesis	
ppa015501m	2.56	0.000690027		Green ontogenesis	
ppa024217m	2.56	0.011101529	cinnamyl-alcohol dehydrogenase	Green ontogenesis	Secondary Metabolism / Phenylpropanoids - Biotic Stress / Secondary metabolism involved into stress
ppa006491m	2.56	0.010007236	Heavy metal transport/detoxification superfamily protein	Green ontogenesis	
ppa018820m	2.56	0.000132814	Leucine-rich repeat protein kinase family protein	Green ontogenesis	
ppa018477m	2.56	0.009985452	Transducin/WD40 repeat-like superfamily protein	Green ontogenesis	
ppa021766m	2.56	0.002742652	VQ motif-containing protein	Green ontogenesis	
ppa026125m	2.55	0.004695941	WRKY DNA-binding protein 70	Green ontogenesis	
ppa015481m	2.55	0.000462685	NAD(P)-linked oxidoreductase superfamily protein	Green ontogenesis	
ppa018053m	2.55	0.000547379	CCT motif family protein	Green ontogenesis	
ppa007298m	2.55	0.000188323	protein kinase 2B	Green ontogenesis	
ppa017073m	2.55	0.027293314	RING/U-box superfamily protein	Green ontogenesis	
ppa011092m	2.55	0.000206818	Protein of unknown function (DUF567)	Green ontogenesis	
ppa014158m	2.54	0.044051346		Green ontogenesis	
ppa024931m	2.54	0.016303288	Calcium-binding EF-hand family protein	Green ontogenesis	
ppa023588m	2.52	0.005807016	alpha/beta-Hydrolases superfamily protein	Green ontogenesis	
ppa006701m	2.52	3.27E-05	ureide permease 2	Green ontogenesis	
ppa025256m	2.52	0.000418743	Alpha/beta hydrolase related protein	Green ontogenesis	
ppa012765m	2.52	0.034667109	Chaperone DnaJ-domain superfamily protein	Green ontogenesis	
ppa001276m	2.51	4.68E-05	stachyose synthase	Green ontogenesis	
ppa005317m	2.51	0.005352781	Nucleotide-diphospho-sugar transferases superfamily protein	Green ontogenesis	
ppa021901m	2.51	7.21E-05	cytochrome P450, family 72, subfamily A, polypeptide 9	Green ontogenesis	
ppa014816m	2.51	0.000205527	UDP-glucosyl transferase 73B3	Green ontogenesis	
ppa006568m	2.51	0.001863184	FAD-binding Berberine family protein	Green ontogenesis	
ppa003155m	2.51	0.000269032	Major facilitator superfamily protein	Green ontogenesis	
ppa019145m	2.51	0.001063194	UDP-glucosyl transferase 85A3	Green ontogenesis	
ppa002656m	2.51	8.92E-05	L-aspartate oxidase	Green ontogenesis	
ppa025579m	2.50	0.000497826	FAD-binding Berberine family protein	Green ontogenesis	
ppa007810m	2.50	0.043678996	Zinc-binding dehydrogenase family protein	Green ontogenesis	
ppa007227m	2.50	0.016740274	matrix metalloproteinase	Green ontogenesis	
ppa013857m	2.49	0.006043615	Low temperature and salt responsive protein family	Green ontogenesis	
ppa022618m	2.49	0.010794213		Green ontogenesis	
ppa015311m	2.49	0.001983852	NAD(P)-linked oxidoreductase superfamily protein	Green ontogenesis	
ppa000999m	2.48	0.00076589	disease resistance family protein / LRR family protein	Green ontogenesis	
ppa004132m	2.48	0.018877209	Major facilitator superfamily protein	Green ontogenesis	
ppb022807m	2.48	0.006306691	cytochrome P450, family 76, subfamily C, polypeptide 4	Green ontogenesis	
ppa007414m	2.47	0.001225256	Glycosyl hydrolase family protein with chitinase insertion domain	Green ontogenesis	
ppa005810m	2.47	0.013173053	galacturonosyltransferase 15	Green ontogenesis	
ppa008924m	2.47	0.043011433	Protein of unknown function (DUF793)	Green ontogenesis	
ppa022941m	2.46	0.002077732	phloem protein 2-A1	Green ontogenesis	
ppa008150m	2.45	0.007033807	RING/U-box superfamily protein	Green ontogenesis	
ppa013533m	2.44	0.002306433		Green ontogenesis	
ppa018846m	2.44	0.01200695	zinc finger (C3HC4-type RING finger) family protein	Green ontogenesis	
ppa011699m	2.44	0.00761725	Plant invertase/pectin methylesterase inhibitor superfamily protei	Green ontogenesis	
ppa019019m	2.44	0.037732196	DNA-binding storekeeper protein-related transcriptional regulator	Green ontogenesis	
ppa006915m	2.44	0.030817416	methionine adenosyltransferase 3	Green ontogenesis	
ppa025305m	2.43	0.002821247	lysine decarboxylase family protein	Green ontogenesis	
ppa002988m	2.43	0.001232829	Calmodulin-binding protein	Green ontogenesis	
ppa010658m	2.43	0.033160329	pathogenesis-related gene 5	Green ontogenesis	
ppa002715m	2.43	0.00049786	hydroxy methylglutaryl CoA reductase 1	Green ontogenesis	Biotic Stress / Secondary metabolism involved into stress
ppa010590m	2.43	0.005189992	Ankyrin repeat family protein	Green ontogenesis	
ppa002027m	2.42	0.044063115	S-locus lectin protein kinase family protein	Green ontogenesis	
ppa001518m	2.42	0.000205856	U-box domain-containing protein kinase family protein	Green ontogenesis	
ppa023567m	2.42	0.020994479	PLANT CADMIUM RESISTANCE 2	Green ontogenesis	
ppa019633m	2.42	0.00350487	UDP-glucosyl transferase 73D1	Green ontogenesis	
ppa009382m	2.42	0.002534823	long-chain acyl-CoA synthetase 2	Green ontogenesis	
ppb013632m	2.42	0.02361387		Green ontogenesis	
ppa001744m	2.41	0.000761436	Raffinose synthase family protein	Green ontogenesis	
ppa022795m	2.41	0.000103776	UDP-Glycosyltransferase superfamily protein	Green ontogenesis	
ppa026269m	2.41	0.004098903	folate transporter 1	Green ontogenesis	
ppa002047m	2.41	0.002242796	wall associated kinase-like 1	Green ontogenesis	
ppa018344m	2.40	0.03860919	MLP-like protein 423	Green ontogenesis	
ppa014652m	2.40	0.000219088	NAD(P)-binding Rossmann-fold superfamily protein	Green ontogenesis	
ppa026582m	2.40	0.005660005	NAC domain containing protein 83	Green ontogenesis	
ppa024588m	2.39	0.000103776	NAD(P)-linked oxidoreductase superfamily protein	Green ontogenesis	
ppb018185m	2.39	0.009241374	cyclic nucleotide gated channel 1	Green ontogenesis	
ppa004404m	2.39	0.003190349	cytochrome P450, family 98, subfamily A, polypeptide 3	Green ontogenesis	
ppa000382m	2.39	0.000848711	aminophospholipid ATPase 1	Green ontogenesis	
ppa010208m	2.39	0.029384223	Nodulin MtN3 family protein	Green ontogenesis	
ppa014636m	2.38	0.002616406	disease resistance family protein / LRR family protein	Green ontogenesis	
ppa023872m	2.38	0.000117613	NmrA-like negative transcriptional regulator family protein	Green ontogenesis	Secondary Metabolism / Flavonols-Isoflavonoid -Biotic Stress / Secondary metabolism involved into stress
ppa004507m	2.38	0.041790001	serine carboxypeptidase-like 48	Green ontogenesis	
ppa020294m	2.38	0.005669229	ENTH/ANTH/VHS superfamily protein	Green ontogenesis	
ppa003853m	2.38	0.001589943	organic cation/carnitine transporter4	Green ontogenesis	
ppa008181m	2.38	0.0017335	farnesyl diphosphate synthase 1	Green ontogenesis	Biotic Stress / Secondary metabolism involved into stress
ppa004127m	2.38	4.07E-05	FAD-binding Berberine family protein	Green ontogenesis	
ppa012537m	2.37	0.017314485	Calcium-dependent lipid-binding (CaLB domain) family protein	Green ontogenesis	
ppa007339m	2.37	0.005399012	purine permease 3	Green ontogenesis	
ppa003574m	2.37	0.002534823	WRKY family transcription factor	Green ontogenesis	
ppa006923m	2.37	0.011526925	Protein of unknown function (DUF607)	Green ontogenesis	
ppa002432m	2.36	0.037125787	wall-associated kinase 2	Green ontogenesis	
ppa009057m	2.36	0.000841883	NAD(P)-linked oxidoreductase superfamily protein	Green ontogenesis	
ppa025229m	2.36	0.00782869	disease resistance protein (TIR-NBS-LRR class), putative	Green ontogenesis	Biotic Stress / PR-proteins
ppa012448m	2.36	0.011025565		Green ontogenesis	
ppa011717m	2.35	0.010285876		Green ontogenesis	
ppa006863m	2.35	0.0099641	magnesium transporter 9	Green ontogenesis	
ppa008600m	2.35	0.002538823	O-acetylserine (thiol) lyase (OAS-TL) isoform A1	Green ontogenesis	
ppa004166m	2.35	0.034515575	bidirectional amino acid transporter 1	Green ontogenesis	
ppa003294m	2.35	0.001006662	Major facilitator superfamily protein	Green ontogenesis	
ppa016353m	2.35	0.044227865	RING/U-box superfamily protein	Green ontogenesis	
ppa026113m	2.35	0.004951383	disease resistance family protein / LRR family protein	Green ontogenesis	
ppa013143m	2.35	0.000993686		Green ontogenesis	
ppa011528m	2.34	0.046161631	LOB domain-containing protein 1	Green ontogenesis	
ppa012676m	2.34	0.000470926	MLP-like protein 423	Green ontogenesis	
ppa022914m	2.34	0.028890665	disease resistance protein (TIR-NBS-LRR class), putative	Green ontogenesis	Biotic Stress / PR-proteins
ppa024274m	2.34	0.000268729	detoxifying efflux carrier 35	Green ontogenesis	
ppa004012m	2.33	8.91E-05	Seven transmembrane MLO family protein	Green ontogenesis	
ppa007498m	2.33	0.00209855	12-oxophytodienoate reductase 2	Green ontogenesis	
ppa022624m	2.32	0.023409734	methyltransferases	Green ontogenesis	
ppa010679m	2.32	0.022241135	K-box region and MADS-box transcription factor family protein	Green ontogenesis	
ppa012602m	2.32	0.015119077	similar to RCD one 2	Green ontogenesis	
ppa020091m	2.32	0.01094648	magnesium transporter 4	Green ontogenesis	
ppa017511m	2.32	0.040609336		Green ontogenesis	
ppa007450m	2.31	0.005886816	2-oxoglutarate (2OG) and Fe(II)-dependent oxygenase superfami	Green ontogenesis	
ppa006516m	2.31	0.001596228	Transmembrane amino acid transporter family protein	Green ontogenesis	
ppa015531m	2.31	0.005080424	BAK1-interacting receptor-like kinase 1	Green ontogenesis	
ppa004822m	2.31	4.53E-05	MATE efflux family protein	Green ontogenesis	
ppa010862m	2.31	0.01544422	NDR1/HIN1-like 3	Green ontogenesis	
ppa024477m	2.31	0.002474415	heat shock cognate protein 70-1	Green ontogenesis	
ppa008580m	2.30	0.002018867	aminoalcoholphosphotransferase 1	Green ontogenesis	
ppa006243m	2.30	0.011760235	AP2/B3 transcription factor family protein	Green ontogenesis	

ppa022188m	2.30	0.021535653		Green ontogenesis	
ppa004389m	2.30	0.026359816	flavin-dependent monooxygenase 1	Green ontogenesis	
ppa004517m	2.30	3.18E-05	cytochrome P450, family 76, subfamily C, polypeptide 4	Green ontogenesis	
ppa014437m	2.30	0.008507916		Green ontogenesis	
ppa020663m	2.30	0.011398353		Green ontogenesis	
ppa008671m	2.30	0.001631799	NAD(P)-binding Rossmann-fold superfamily protein	Green ontogenesis	Secondary Metabolism / Dihydroflavonols -Biotic Stress / Secondary metabolism involved into stress
ppa010896m	2.30	0.001516572	Ribosomal protein L34e superfamily protein	Green ontogenesis	
ppa016190m	2.30	0.010285876		Green ontogenesis	
ppa008664m	2.30	0.01002068	syntaxin of plants 121	Green ontogenesis	
ppa011883m	2.30	0.031742709		Green ontogenesis	
ppa023884m	2.29	0.001054035	Sec14p-like phosphatidylinositol transfer family protein	Green ontogenesis	
ppa015431m	2.29	0.00280873	S-locus lectin protein kinase family protein	Green ontogenesis	
ppa001007m	2.29	0.024393735	NB-ARC domain-containing disease resistance protein	Green ontogenesis	
ppa018702m	2.29	0.00142295	Adenine nucleotide alpha hydrolases-like superfamily protein	Green ontogenesis	
ppb021781m	2.29	0.002977609		Green ontogenesis	
ppa008484m	2.28	0.007553276	Cysteine proteinases superfamily protein	Green ontogenesis	
ppa022630m	2.28	0.003167148	MATE efflux family protein	Green ontogenesis	
ppa012971m	2.28	0.006279616	Chaperone DnaJ-domain superfamily protein	Green ontogenesis	
ppa005140m	2.27	0.000316968	MATE efflux family protein	Green ontogenesis	
ppa007260m	2.27	0.001502712	GroES-like zinc-binding dehydrogenase family protein	Green ontogenesis	
ppa002159m	2.26	0.005184854	Concanavalin A-like lectin protein kinase family protein	Green ontogenesis	
ppa005018m	2.26	0.000132814	MATE efflux family protein	Green ontogenesis	
ppa015243m	2.26	0.006871587	disease resistance family protein / LRR family protein	Green ontogenesis	
ppa011108m	2.26	0.000124561	glutathione S-transferase tau 7	Green ontogenesis	Biotic Stress / Glutathione-S-Transferase
ppa004000m	2.26	0.004946255	SKU5 similar 5	Green ontogenesis	
ppa009161m	2.26	0.018379088	TOXICOS EN LEVADURA 2	Green ontogenesis	
ppa011811m	2.25	0.043355866	PYR1-like 4	Green ontogenesis	
ppa008284m	2.25	0.024967589	Polynucleotidyl transferase, ribonuclease H-like superfamily prote	Green ontogenesis	
ppa005782m	2.25	0.001441726	DHHC-type zinc finger family protein	Green ontogenesis	
ppa004804m	2.25	9.98E-05	don-glucosyltransferase 1	Green ontogenesis	
ppa004646m	2.25	3.27E-05	Cytochrome P450 superfamily protein	Green ontogenesis	
ppa003107m	2.25	0.008842656	amino acid transporter 1	Green ontogenesis	
ppa017389m	2.24	0.01448622	S-locus lectin protein kinase family protein	Green ontogenesis	
ppa011307m	2.24	0.040969373	glutathione S-transferase phi 12	Green ontogenesis	Biotic Stress / Glutathione-S-Transferase
ppa024613m	2.24	0.028644413	receptor like protein 31	Green ontogenesis	
ppa010796m	2.24	0.007906639	WRKY DNA-binding protein 40	Green ontogenesis	
ppa008760m	2.23	0.012635273	peroxisomal adenine nucleotide carrier 1	Green ontogenesis	
ppa001159m	2.23	0.000911985	glutamate receptor 2.7	Green ontogenesis	
ppa017807m	2.23	0.003551152	FAD-dependent oxidoreductase family protein	Green ontogenesis	
ppb016773m	2.23	0.001189758	pleiotropic drug resistance 11	Green ontogenesis	
ppa024811m	2.23	0.009967242		Green ontogenesis	
ppa007377m	2.23	0.000247755	12-oxophytodienoate reductase 2	Green ontogenesis	
ppa022521m	2.23	0.021174045	disease resistance protein (TIR-NBS-LRR class), putative	Green ontogenesis	Biotic Stress / PR-proteins
ppa012715m	2.23	0.006368595		Green ontogenesis	
ppa018496m	2.22	0.010110676	Nucleotide-sugar transporter family protein	Green ontogenesis	
ppa013356m	2.22	0.009154732	photosystem II BY	Green ontogenesis	
ppa007343m	2.22	0.025023254	GroES-like zinc-binding alcohol dehydrogenase family protein	Green ontogenesis	
ppa005716m	2.22	0.002118342	ACT domain repeat 4	Green ontogenesis	
ppa020532m	2.21	0.000878265		Green ontogenesis	
ppa025693m	2.21	0.00100309	S-locus lectin protein kinase family protein	Green ontogenesis	
ppa003057m	2.21	0.04254468	ankyrin repeat family protein	Green ontogenesis	
ppa004913m	2.21	0.002042876	with no lysine (K) kinase 4	Green ontogenesis	
ppa026853m	2.21	0.006232608	glutamate receptor 2.8	Green ontogenesis	
ppa013627m	2.21	0.033346291	amino acid permease 7	Green ontogenesis	
ppa016869m	2.21	0.027301754	disease resistance family protein / LRR family protein	Green ontogenesis	
ppa010137m	2.20	0.003859734	S-adenosyl-L-methionine-dependent methyltransferases superfar	Green ontogenesis	
ppa002340m	2.20	0.014842022	ARM repeat superfamily protein	Green ontogenesis	
ppa004555m	2.20	0.001184711	aldehyde dehydrogenase 2C4	Green ontogenesis	
ppa008532m	2.20	0.000275217	RmlC-like cupins superfamily protein	Green ontogenesis	
ppa016264m	2.19	0.001925237	Transmembrane amino acid transporter family protein	Green ontogenesis	
ppa004870m	2.19	0.0286543	serine/threonine protein kinase 2	Green ontogenesis	
ppa016343m	2.19	0.012701221	Protein kinase superfamily protein	Green ontogenesis	
ppa021725m	2.19	0.013680183	NAD(P)-linked oxidoreductase superfamily protein	Green ontogenesis	
ppa005058m	2.19	0.002792365	Pyridoxal phosphate (PLP)-dependent transferases superfamily p	Green ontogenesis	Biotic Stress / Secondary metabolism involved into stress
ppa004902m	2.19	0.0051045	alanine:glyoxylate aminotransferase 2	Green ontogenesis	
ppa023846m	2.19	0.008261194		Green ontogenesis	
ppa007708m	2.19	0.007051345	WRKY family transcription factor	Green ontogenesis	
ppa021494m	2.19	0.017676514		Green ontogenesis	
ppa024243m	2.19	0.004804127	Calcium-dependent lipid-binding (CaLB domain) family protein	Green ontogenesis	
ppa001216m	2.19	0.031761212	lipoxygenase 1	Green ontogenesis	
ppa019306m	2.19	0.006748878	BTB/POZ domain with WD40/YVTN repeat-like protein	Green ontogenesis	
ppa010077m	2.18	0.021479146	C2H2 and C2HC zinc fingers superfamily protein	Green ontogenesis	
ppa014141m	2.18	0.008561696	Wound-responsive family protein	Green ontogenesis	
ppa017329m	2.18	0.000373537	cytochrome P450, family 76, subfamily C, polypeptide 4	Green ontogenesis	
ppa013228m	2.18	0.027444619		Green ontogenesis	
ppb014879m	2.18	0.002866305	Pectinacetylesterase family protein	Green ontogenesis	
ppa005211m	2.17	0.001141283	Ankyrin repeat family protein	Green ontogenesis	
ppa019532m	2.17	0.00427156	phospholipase A 2A	Green ontogenesis	
ppa003286m	2.17	0.010274587	NAD(P)H dehydrogenase B3	Green ontogenesis	
ppa009343m	2.17	0.00345156		Green ontogenesis	
ppa002533m	2.16	0.000450331	Leucine-rich repeat protein kinase family protein	Green ontogenesis	
ppa007606m	2.16	0.004866414	Integrase-type DNA-binding superfamily protein	Green ontogenesis	
ppa001941m	2.16	0.001061233	cellulose synthase like E1	Green ontogenesis	
ppa020051m	2.15	0.000831785	zinc transporter 1 precursor	Green ontogenesis	
ppa022410m	2.14	0.032196206		Green ontogenesis	
ppa004087m	2.14	0.023115994	FAD-binding Berberine family protein	Green ontogenesis	
ppa007488m	2.14	0.007328351	12-oxophytodienoate reductase 2	Green ontogenesis	
ppa001867m	2.14	0.003110718	cellulose synthase like G2	Green ontogenesis	
ppa003854m	2.14	0.012470203	4-coumarate:CoA ligase 2	Green ontogenesis	Secondary Metabolism / Phenylpropanoids - Biotic Stress / Secondary metabolism involved into stress
ppa005725m	2.14	0.000470926	alpha/beta-Hydrolases superfamily protein	Green ontogenesis	
ppa022971m	2.14	0.034193217	wall associated kinase-like 2	Green ontogenesis	
ppa002593m	2.13	0.000205527	alpha/beta-Hydrolases superfamily protein	Green ontogenesis	
ppa010607m	2.13	0.000668152	Sec14p-like phosphatidylinositol transfer family protein	Green ontogenesis	
ppb011907m	2.13	0.048572901		Green ontogenesis	
ppa003699m	2.13	0.003959515	ARM repeat superfamily protein	Green ontogenesis	
ppa006714m	2.13	0.020087878	Protein kinase superfamily protein	Green ontogenesis	
ppa008326m	2.13	0.006708838	UDP-Glycosyltransferase superfamily protein	Green ontogenesis	
ppa002946m	2.12	0.013788691	exocyst subunit exo70 family protein H2	Green ontogenesis	
ppa009963m	2.12	0.001497785	ribonuclease 2	Green ontogenesis	
ppa004860m	2.12	0.000399037	6-phosphogluconate dehydrogenase family protein	Green ontogenesis	
ppa016022m	2.11	0.048271664	Oxidoreductase, zinc-binding dehydrogenase family protein	Green ontogenesis	
ppa018576m	2.11	0.018408478	AAA-ATPase 1	Green ontogenesis	
ppa009584m	2.11	0.020876585	EID1-like 3	Green ontogenesis	
ppa018253m	2.11	0.004201106	Protein kinase superfamily protein	Green ontogenesis	
ppa001861m	2.11	0.000655284	cellulose synthase like G2	Green ontogenesis	
ppa004250m	2.11	0.000236353	methyltransferases	Green ontogenesis	
ppa004328m	2.10	0.00142295	Cytochrome P450 superfamily protein	Green ontogenesis	
ppa010313m	2.10	0.009051866	NAD(P)-binding Rossmann-fold superfamily protein	Green ontogenesis	
ppa007079m	2.10	0.001136153	Glycosyl hydrolase superfamily protein	Green ontogenesis	Biotic Stress / Betaglucanase
ppa004071m	2.09	0.000175421	magnesium/proton exchanger	Green ontogenesis	
ppa010244m	2.09	0.000305106	VIRB2-interacting protein 2	Green ontogenesis	
ppa015797m	2.09	0.008587072	disease resistance family protein / LRR family protein	Green ontogenesis	
ppb015490m	2.09	0.001152163	UDP-glucosyl transferase 85A2	Green ontogenesis	
ppa008854m	2.09	0.007291995	alpha/beta-Hydrolases superfamily protein	Green ontogenesis	
ppa026046m	2.09	0.04353928	related to ABI3/VP1 2	Green ontogenesis	
ppa020628m	2.08	0.017713847	Regulator of chromosome condensation (RCC1) family with FYVE	Green ontogenesis	
ppa010497m	2.08	0.010616368	S-adenosyl-L-methionine-dependent methyltransferases superfar	Green ontogenesis	Secondary Metabolism / Phenylpropanoids - Biotic Stress / Secondary metabolism involved into stress
ppa015821m	2.08	0.007704364	FAD-binding Berberine family protein	Green ontogenesis	
ppa010303m	2.08	0.030879233	indole-3-acetic acid 7	Green ontogenesis	
ppa006913m	2.08	0.036895494	ADP/ATP carrier 2	Green ontogenesis	
ppa001161m	2.08	0.001388366	Leucine-rich repeat transmembrane protein kinase	Green ontogenesis	
ppa015527m	2.07	0.025557061		Green ontogenesis	
ppa018737m	2.07	0.014294403		Green ontogenesis	
ppa004927m	2.07	0.005858453		Green ontogenesis	
ppa018724m	2.07	0.0001494	Cytochrome P450 superfamily protein	Green ontogenesis	
ppa006368m	2.07	1.46E-05	Tyrosine transaminase family protein	Green ontogenesis	
ppa018866m	2.07	0.010861233	methyl esterase 3	Green ontogenesis	
ppa011663m	2.07	0.000124561	cold-regulated 413-plasma membrane 2	Green ontogenesis	
ppa011245m	2.07	0.007234481	Putative lysine decarboxylase family protein	Green ontogenesis	
ppa011049m	2.07	0.004296715	BAK1-interacting receptor-like kinase 1	Green ontogenesis	
ppa025538m	2.06	0.006681175	Yippee family putative zinc-binding protein	Green ontogenesis	
ppa008466m	2.06	0.005075304	Staphylococcal nuclease homologue	Green ontogenesis	
ppa007381m	2.06	0.002846214	12-oxophytodienoate reductase 2	Green ontogenesis	
ppa007308m	2.06	0.000282055		Green ontogenesis	
ppa006940m	2.05	0.00490607	Serinc-domain containing serine and sphingolipid biosynthesis pro	Green ontogenesis	
ppa023692m	2.05	0.007317315	Cytochrome P450 superfamily protein	Green ontogenesis	
ppa007860m	2.05	0.046296138	C2H2-type zinc finger family protein	Green ontogenesis	
ppa020322m	2.05	0.001587538	lectin receptor kinase a4.1	Green ontogenesis	
ppa009289m	2.05	0.004193429	RmlC-like cupins superfamily protein	Green ontogenesis	
ppa012040m	2.05	0.001136281	F-box family protein	Green ontogenesis	
ppa008837m	2.05	0.013292855	Homeodomain-like superfamily protein	Green ontogenesis	
ppa007462m	2.05	0.000172633	Aldolase-type TIM barrel family protein	Green ontogenesis	
ppa005959m	2.05	0.004147776	alpha/beta-Hydrolases superfamily protein	Green ontogenesis	
ppa018327m	2.04	0.000537017	S-locus lectin protein kinase family protein	Green ontogenesis	
ppa025014m	2.04	0.026681255	Late embryogenesis abundant (LEA) hydroxyproline-rich glycopro	Green ontogenesis	
ppa005595m	2.04	0.016083725		Green ontogenesis	
ppa016463m	2.04	0.01048987	UDP-glucosyl transferase 74B1	Green ontogenesis	
ppa014950m	2.03	0.041790001	P-loop containing nucleoside triphosphate hydrolases superfamily	Green ontogenesis	

ppa005199m	2.03	0.000399037	*Granulin repeat cysteine protease family protein*	Green ontogenesis	
ppa005064m	2.03	0.00474749	*Protein kinase protein with tetratricopeptide repeat domain*	Green ontogenesis	
ppa001003m	2.03	0.015956537	*NB-ARC domain-containing disease resistance protein*	Green ontogenesis	
ppa020074m	2.03	0.013332524	*Outer arm dynein light chain 1 protein*	Green ontogenesis	
ppa016270m	2.02	0.001047863	*cationic amino acid transporter 5*	Green ontogenesis	
ppa022354m	2.02	0.00016382	*Putative lysine decarboxylase family protein*	Green ontogenesis	
ppa004208m	2.02	0.004828722	*bidirectional amino acid transporter 1*	Green ontogenesis	
ppa021591m	2.02	0.025310105	*methyl esterase 3*	Green ontogenesis	
ppa022271m	2.02	0.019146701	*disease resistance family protein / LRR family protein*	Green ontogenesis	
ppa015392m	2.02	0.009043473	*Integrase-type DNA-binding superfamily protein*	Green ontogenesis	
ppa006933m	2.01	0.001232829	*aminoalcoholphosphotransferase 1*	Green ontogenesis	
ppa003466m	2.01	0.000753057	*Seven transmembrane MLO family protein*	Green ontogenesis	
ppa005425m	2.01	0.00111901		Green ontogenesis	
ppa011617m	2.01	0.000446946	*Quinone reductase family protein*	Green ontogenesis	
ppa006890m	2.01	0.000872438	*Protein kinase superfamily protein*	Green ontogenesis	
ppa007294m	2.01	0.000316968	*S-adenosylmethionine decarboxylase*	Green ontogenesis	
ppa010772m	2.00	0.007553276	*Rubber elongation factor protein (REF)*	Green ontogenesis	
ppa026759m	2.00	0.002096342	*exocyst subunit exo70 family protein E2*	Green ontogenesis	
ppa022500m	2.00	0.006749842	*fatty acid desaturase A*	Green ontogenesis	
ppa015608m	1.99	0.000768548	*receptor kinase 3*	Green ontogenesis	
ppa004843m	1.99	0.000353142	*BR-signaling kinase 3*	Green ontogenesis	
ppa010714m	1.99	0.001924272	*K-box region and MADS-box transcription factor family protein*	Green ontogenesis	
ppa000380m	1.98	0.00100309	*ATPase E1-E2 type family protein / haloacid dehalogenase-like h*	Green ontogenesis	
ppa001414m	1.97	0.000628813	*S-locus lectin protein kinase family protein*	Green ontogenesis	
ppa001921m	1.97	9.48E-05	*quinolinate synthase*	Green ontogenesis	
ppa008480m	1.97	0.017794574	*WRKY DNA-binding protein 57*	Green ontogenesis	
ppa017527m	1.97	0.001720439	*NAD(P)-binding Rossmann-fold superfamily protein*	Green ontogenesis	Biotic Stress / Secondary metabolism involved into stress
ppa024391m	1.97	0.002596451	*senescence-associated gene 101*	Green ontogenesis	
ppa004219m	1.97	0.000484292	*sugar transporter 1*	Green ontogenesis	
ppa004317m	1.96	0.000197511	*FAD-binding Berberine family protein*	Green ontogenesis	
ppa010878m	1.96	0.039062021	*Protein of unknown function (DUF1640)*	Green ontogenesis	
ppa004430m	1.96	0.018652447	*myo-inositol-1-phosphate synthase 2*	Green ontogenesis	
ppa003890m	1.96	0.008313967	*phospholipase C 2*	Green ontogenesis	
ppa001689m	1.96	0.024024493	*subtilase family protein*	Green ontogenesis	
ppa013075m	1.96	0.011926178	*plant natriuretic peptide A*	Green ontogenesis	
ppa002738m	1.96	0.031724729	*exocyst subunit exo70 family protein H4*	Green ontogenesis	
ppa012457m	1.95	0.000615025		Green ontogenesis	
ppa007549m	1.95	0.000652427	*Protein of unknown function, DUF593*	Green ontogenesis	
ppa011570m	1.95	0.003635976	*AUX/IAA transcriptional regulator family protein*	Green ontogenesis	
ppa024490m	1.95	0.047374845		Green ontogenesis	
ppa015844m	1.95	0.001168629	*phospholipase A 2A*	Green ontogenesis	
ppa022731m	1.95	0.005132335	*SBP (S-ribonuclease binding protein) family protein*	Green ontogenesis	
ppa022569m	1.95	0.000440665	*MATE efflux family protein*	Green ontogenesis	
ppa022492m	1.93	0.001221407	*purine permease 3*	Green ontogenesis	
ppa004260m	1.93	0.030359569	*ferulic acid 5-hydroxylase 1*	Green ontogenesis	
ppa003950m	1.93	0.0093592	*cytochrome P450, family 711, subfamily A, polypeptide 1*	Green ontogenesis	
ppa009959m	1.93	0.002718051	*NAD(P)-binding Rossmann-fold superfamily protein*	Green ontogenesis	
ppa005067m	1.93	0.001301406	*ferrochelatase 1*	Green ontogenesis	
ppa011859m	1.93	0.042192182		Green ontogenesis	
ppa011833m	1.93	0.002539136	*RING-H2 finger B1A*	Green ontogenesis	
ppa001724m	1.93	0.012226838	*K+ uptake permease 6*	Green ontogenesis	
ppa002534m	1.92	0.016363726	*tolB protein-related*	Green ontogenesis	
ppa026594m	1.92	0.002349391	*disease resistance family protein / LRR family protein*	Green ontogenesis	
ppa012603m	1.92	0.006960606	*Putative lysine decarboxylase family protein*	Green ontogenesis	
ppa004461m	1.92	0.014008519	*aldehyde dehydrogenase 7B4*	Green ontogenesis	
ppa024764m	1.92	0.014729317	*NB-ARC domain-containing disease resistance protein*	Green ontogenesis	Biotic Stress / PR-proteins
ppa004691m	1.92	0.025023254	*AAA-ATPase 1*	Green ontogenesis	
ppa005856m	1.92	0.006618789	*Galactose oxidase/kelch repeat superfamily protein*	Green ontogenesis	
ppa009844m	1.92	0.027345248	*homolog of carrot EP3-3 chitinase*	Green ontogenesis	
ppa010186m	1.92	0.013321272	*ethylene-responsive element binding factor 13*	Green ontogenesis	
ppa006014m	1.91	0.000561887	*Histone H3 K4-specific methyltransferase SET7/9 family protein*	Green ontogenesis	
ppa002849m	1.91	0.002151548	*VACUOLAR SORTING RECEPTOR 7*	Green ontogenesis	
ppa026874m	1.91	0.004536747	*cytochrome P450, family 72, subfamily A, polypeptide 7*	Green ontogenesis	
ppa008297m	1.91	0.026329596	*Mitochondrial substrate carrier family protein*	Green ontogenesis	
ppa012678m	1.91	0.005091737	*MLP-like protein 423*	Green ontogenesis	
ppa000828m	1.91	0.013128026	*disease resistance protein (TIR-NBS-LRR class)*	Green ontogenesis	Biotic Stress / PR-proteins
ppa003732m	1.91	0.011721277	*acyl activating enzyme 5*	Green ontogenesis	
ppa009859m	1.91	0.013661909	*GNS1/SUR4 membrane protein family*	Green ontogenesis	
ppa021907m	1.90	0.001873193	*carboxyesterase 13*	Green ontogenesis	
ppa007938m	1.90	0.000781317	*F-box and associated interaction domains-containing protein*	Green ontogenesis	
ppa023857m	1.90	0.025679367	*GroES-like zinc-binding dehydrogenase family protein*	Green ontogenesis	
ppa006290m	1.90	0.000784343	*GHMP kinase family protein*	Green ontogenesis	Biotic Stress / Secondary metabolism involved into stress
ppa005896m	1.90	0.002457684	*Mannose-6-phosphate isomerase, type I*	Green ontogenesis	
ppa021744m	1.90	0.040477592	*ATPase E1-E2 type family protein / haloacid dehalogenase-like h*	Green ontogenesis	
ppa009297m	1.90	0.015223836	*NAD(P)-binding Rossmann-fold superfamily protein*	Green ontogenesis	
ppa007779m	1.90	0.001293437	*zinc transporter 1 precursor*	Green ontogenesis	
ppa003207m	1.89	0.025026815	*Seven transmembrane MLO family protein*	Green ontogenesis	
ppa010754m	1.89	0.011039083	*zinc transporter 11 precursor*	Green ontogenesis	
ppa008695m	1.89	0.00076589	*non-intrinsic ABC protein 9*	Green ontogenesis	
ppa008039m	1.89	0.042609538	*Protein of unknown function (DUF1645)*	Green ontogenesis	
ppa025037m	1.89	0.000202279	*phosphate transporter 1;9*	Green ontogenesis	
ppa023836m	1.88	0.046129185	*lipid transfer protein 1*	Green ontogenesis	
ppa020096m	1.88	0.016360803	*BED zinc finger ;hAT family dimerisation domain*	Green ontogenesis	
ppa011611m	1.88	0.002414148	*serine-rich protein-related*	Green ontogenesis	
ppa017124m	1.88	0.006211218	*UDP-glucosyl transferase 76E2*	Green ontogenesis	
ppa013490m	1.88	0.008036829		Green ontogenesis	
ppa018704m	1.87	0.001149792	*related to AP2.7*	Green ontogenesis	
ppa001241m	1.87	0.006050857	*K+ transporter 1*	Green ontogenesis	
ppa026048m	1.87	0.018134402	*MAP kinase substrate 1*	Green ontogenesis	
ppa014995m	1.86	0.000168147	*S-locus lectin protein kinase family protein*	Green ontogenesis	
ppa000572m	1.86	0.014954422	*phospholipase D P1*	Green ontogenesis	
ppa003339m	1.86	0.000298243	*peptide transporter 2*	Green ontogenesis	
ppa006098m	1.86	0.003041936	*Protein kinase superfamily protein*	Green ontogenesis	
ppa013446m	1.86	0.049066275		Green ontogenesis	
ppa003447m	1.86	0.02637794	*Auxin-responsive GH3 family protein*	Green ontogenesis	
ppa002847m	1.86	0.011848969	*Plant neutral invertase family protein*	Green ontogenesis	
ppa000623m	1.86	0.029384223	*C2 calcium/lipid-binding plant phosphoribosyltransferase family pı*	Green ontogenesis	
ppa016737m	1.86	0.02842968	*Concanavalin A-like lectin protein kinase family protein*	Green ontogenesis	
ppa015802m	1.86	0.014842022		Green ontogenesis	
ppa023323m	1.86	0.046025379	*Transducin/WD40 repeat-like superfamily protein*	Green ontogenesis	
ppa002142m	1.85	0.0008032	*P450 reductase 2*	Green ontogenesis	Secondary Metabolism / Phenylpropanoids - Biotic Stress / Secondary metabolism involved into stress
ppa006723m	1.85	0.016906861	*Core-2/I-branching beta-1,6-N-acetylglucosaminyltransferase fam*	Green ontogenesis	
ppa002508m	1.85	0.006211218	*glutaredoxin-related*	Green ontogenesis	
ppa004742m	1.85	0.004215141	*S-adenosyl-L-methionine-dependent methyltransferases superfar*	Green ontogenesis	
ppa000723m	1.85	0.016651319	*ARM repeat superfamily protein*	Green ontogenesis	
ppa002407m	1.85	0.031755507	*Restriction endonuclease, type II-like superfamily protein*	Green ontogenesis	
ppa009662m	1.84	0.000418246	*O-acetylserine (thiol) lyase (OAS-TL) isoform A1*	Green ontogenesis	
ppa000239m	1.84	0.037139623	*pleiotropic drug resistance 6*	Green ontogenesis	
ppa019028m	1.84	0.000275217	*Protein kinase superfamily protein*	Green ontogenesis	
ppb001660m	1.84	0.000418246	*receptor kinase 3*	Green ontogenesis	
ppa009290m	1.84	0.000527772	*NAD(P)-binding Rossmann-fold superfamily protein*	Green ontogenesis	
ppa015981m	1.84	0.042174995		Green ontogenesis	
ppa007600m	1.84	0.005553602	*senescence-related gene 1*	Green ontogenesis	
ppa005262m	1.84	0.02070327	*E2F transcription factor 3*	Green ontogenesis	
ppa022087m	1.83	0.000399037	*Calcium-dependent phosphotriesterase superfamily protein*	Green ontogenesis	Biotic Stress / Secondary metabolism involved into stress
ppa021722m	1.83	0.037528665	*cell wall-associated kinase*	Green ontogenesis	
ppa024869m	1.83	0.023009563	*NAD(P)-binding Rossmann-fold superfamily protein*	Green ontogenesis	Secondary Metabolism / Dihydroflavonols -Biotic Stress / Secondary metabolism involved into stress
ppa000489m	1.82	0.018559253	*disease resistance protein (TIR-NBS-LRR class), putative*	Green ontogenesis	Biotic Stress / PR-proteins
ppa003995m	1.82	0.000742765	*FAD/NAD(P)-binding oxidoreductase family protein*	Green ontogenesis	
ppa005935m	1.82	0.000226291	*calmodulin-binding receptor-like cytoplasmic kinase 2*	Green ontogenesis	
ppa004989m	1.82	0.000237872	*UDP-glucosyl transferase 85A3*	Green ontogenesis	
ppa010338m	1.82	0.00220748	*thiamin pyrophosphokinase1*	Green ontogenesis	
ppa017572m	1.82	0.000263303	*receptor kinase 3*	Green ontogenesis	
ppa1027190m	1.82	0.018933211	*Disease resistance protein (CC-NBS-LRR class) family*	Green ontogenesis	Biotic Stress / PR-proteins
ppa010812m	1.81	0.018290212		Green ontogenesis	
ppa007741m	1.81	0.0008032	*nudix hydrolase homolog 2*	Green ontogenesis	
ppa002246m	1.81	0.007653424	*catalytics*	Green ontogenesis	
ppa011200m	1.81	0.047621076	*nudix hydrolase homolog 15*	Green ontogenesis	
ppa019102m	1.81	0.009738624	*RING/U-box superfamily protein*	Green ontogenesis	
ppb010273m	1.81	0.009760837		Green ontogenesis	
ppa023464m	1.81	0.000359249		Green ontogenesis	
ppa008037m	1.80	0.005103312	*choline kinase 1*	Green ontogenesis	
ppa014999m	1.80	0.012790305	*PR5-like receptor kinase*	Green ontogenesis	
ppa014998m	1.80	0.000188323	*Disease resistance protein (CC-NBS-LRR class) family*	Green ontogenesis	Biotic Stress / PR-proteins
ppa010683m	1.80	0.002586647	*indole-3-acetic acid inducible 29*	Green ontogenesis	
ppa017413m	1.80	0.01553773		Green ontogenesis	
ppa002102m	1.80	0.020045456	*RELA/SPOT homolog 3*	Green ontogenesis	
ppa026893m	1.80	0.016020443		Green ontogenesis	
ppa003398m	1.80	0.028890665	*Major facilitator superfamily protein*	Green ontogenesis	
ppa010700m	1.79	0.019650129	*delta(3), delta(2)-enoyl CoA isomerase 1*	Green ontogenesis	
ppa002516m	1.79	0.00361244	*cysteine-rich RLK (RECEPTOR-like protein kinase) 25*	Green ontogenesis	
ppa007421m	1.79	0.013661288	*homeobox from Arabidopsis thaliana*	Green ontogenesis	
ppa003747m	1.79	0.002753661	*4-coumarate:CoA ligase 2*	Green ontogenesis	Secondary Metabolism / Phenylpropanoids - Biotic Stress / Secondary metabolism involved into stress
ppa013035m	1.79	0.038908681		Green ontogenesis	
ppa004344m	1.79	0.00159037	*sugar transporter 14*	Green ontogenesis	
ppa026676m	1.79	0.005060223	*ATPase, F1 complex, gamma subunit protein*	Green ontogenesis	Photosynthesis / Redox chain
ppa014240m	1.79	0.046602357		Green ontogenesis	
ppa002688m	1.79	0.00115899	*SCARECROW-like 8*	Green ontogenesis	

ppa007490m	1.79	0.001136281	12-oxophytodienoate reductase 2	Green ontogenesis	
ppa011729m	1.79	0.002596451	Uncharacterised protein family (UPF0497)	Green ontogenesis	
ppa002585m	1.78	0.013854501	Glycosyl hydrolase superfamily protein	Green ontogenesis	
ppa004503m	1.78	0.034907224	cytochrome P450, family 76, subfamily C, polypeptide 4	Green ontogenesis	
ppa015494m	1.78	0.003037627	senescence-associated gene 101	Green ontogenesis	
ppa025237m	1.78	0.03974002		Green ontogenesis	
ppa022686m	1.77	0.015102726	receptor serine/threonine kinase, putative	Green ontogenesis	
ppa012573m	1.77	0.00118508	MLP-like protein 423	Green ontogenesis	
ppa000657m	1.77	0.026189885	lysine-ketoglutarate reductase/saccharopine dehydrogenase bifu	Green ontogenesis	
ppa021995m	1.77	0.017346489	glutamate receptor 2.7	Green ontogenesis	
ppa021058m	1.77	0.027301754	UDP-glucosyl transferase 85A7	Green ontogenesis	
ppa012579m	1.77	0.00059868	RING-H2 finger A2A	Green ontogenesis	
ppa016153m	1.76	0.002426982	WRKY DNA-binding protein 75	Green ontogenesis	
ppa007829m	1.76	0.014731525	zinc finger (C3HC4-type RING finger) family protein	Green ontogenesis	
ppa003881m	1.76	0.001035247	Calcium-dependent lipid-binding (CaLB domain) family protein	Green ontogenesis	
ppa002186m	1.76	0.019545464	Protein kinase superfamily protein	Green ontogenesis	
ppa025607m	1.76	0.016651319	Wall-associated kinase family protein	Green ontogenesis	
ppa002570m	1.76	0.000768548	Purple acid phosphatases superfamily protein	Green ontogenesis	
ppa016634m	1.76	0.007200057	disease resistance protein (TIR-NBS-LRR class), putative	Green ontogenesis	Biotic Stress / PR-proteins
ppa011272m	1.76	0.000832133	Late embryogenesis abundant (LEA) hydroxyproline-rich glycopro	Green ontogenesis	
ppa017528m	1.76	0.000182969	Leucine-rich receptor-like protein kinase family protein	Green ontogenesis	
ppa012449m	1.76	0.005503048	Adenine nucleotide alpha hydrolases-like superfamily protein	Green ontogenesis	
ppa010998m	1.75	0.004098744	pyrophosphorylase 4	Green ontogenesis	
ppa006043m	1.75	0.001245281	cation exchanger 1	Green ontogenesis	
ppa004086m	1.75	0.001696612	phosphofructokinase 5	Green ontogenesis	
ppa008589m	1.75	0.001631799	Cysteine proteinases superfamily protein	Green ontogenesis	
ppa001539m	1.75	0.001768487	OSBP(oxysterol binding protein)-related protein 1C	Green ontogenesis	
ppa012929m	1.75	0.022258492	calmodulin-like 11	Green ontogenesis	
ppa005735m	1.75	0.006595396		Green ontogenesis	
ppa017377m	1.74	0.01285991	Remorin family protein	Green ontogenesis	
ppa005266m	1.74	0.008775002	hydroxymethylglutaryl-CoA synthase / HMG-CoA synthase / 3-hy	Green ontogenesis	Biotic Stress / Secondary metabolism involved into stress
ppa006132m	1.74	0.004695941	reticulata-related 1	Green ontogenesis	
ppa011916m	1.74	0.001797555		Green ontogenesis	
ppa011011m	1.73	0.004490337	Cysteine proteinases superfamily protein	Green ontogenesis	
ppa013342m	1.73	0.019681096	C-terminal cysteine residue is changed to a serine 1	Green ontogenesis	
ppa022847m	1.72	0.000724297	Seven transmembrane MLO family protein	Green ontogenesis	
ppa023148m	1.72	0.017243104	Leucine-rich receptor-like protein kinase family protein	Green ontogenesis	
ppa007934m	1.72	0.003688755	succinate dehydrogenase 2-2	Green ontogenesis	
ppa011747m	1.72	0.001425605	Chlorophyll A-B binding family protein	Green ontogenesis	
ppa003904m	1.72	0.010234948	cysteine-rich RLK (RECEPTOR-like protein kinase) 25	Green ontogenesis	
ppa013829m	1.72	0.01414853		Green ontogenesis	
ppb011375m	1.72	0.043910042	2-oxoglutarate (2OG) and Fe(II)-dependent oxygenase superfam	Green ontogenesis	
ppa026506m	1.71	0.02882318	UDP-glucosyl transferase 85A3	Green ontogenesis	
ppa011746m	1.71	0.00331226		Green ontogenesis	
ppa003549m	1.71	0.001129143	dicarboxylate transporter 1	Green ontogenesis	
ppa004984m	1.71	0.002510676	cytochrome P450, family 716, subfamily A, polypeptide 1	Green ontogenesis	
ppa007800m	1.71	0.000138416	Nodulin MtN21 /EamA-like transporter family protein	Green ontogenesis	
ppa006789m	1.71	0.000520894	NAD(P)-binding Rossmann-fold superfamily protein	Green ontogenesis	
ppa011094m	1.71	0.025373918	RAB GTPase homolog A6A	Green ontogenesis	
ppa001842m	1.71	0.000265115	S-locus lectin protein kinase family protein	Green ontogenesis	
ppa007964m	1.71	0.04984386	Protein of unknown function (DUF761)	Green ontogenesis	
ppa006160m	1.71	0.000298243	alpha-amylase-like	Green ontogenesis	
ppa008494m	1.71	0.016303288	pfkB-like carbohydrate kinase family protein	Green ontogenesis	
ppa008613m	1.70	0.002538823	WRKY DNA-binding protein 7	Green ontogenesis	
ppa001372m	1.70	0.001863184	lectin protein kinase family protein	Green ontogenesis	
ppa011080m	1.70	0.040651652	serine-rich protein-related	Green ontogenesis	
ppa008165m	1.70	0.000927187	pfkB-like carbohydrate kinase family protein	Green ontogenesis	
ppa011682m	1.70	0.01037097	glutathione peroxidase 3	Green ontogenesis	
ppa012949m	1.69	0.001141283	Late embryogenesis abundant protein	Green ontogenesis	
ppa1027192m	1.69	0.008214002	12-oxophytodienoate reductase 2	Green ontogenesis	
ppa025089m	1.69	0.038162273	Protein kinase superfamily protein	Green ontogenesis	
ppa005891m	1.69	0.000443007	RING/U-box superfamily protein	Green ontogenesis	
ppa019900m	1.69	0.015804368	disease resistance family protein / LRR family protein	Green ontogenesis	
ppa009733m	1.69	0.007335849	calcium-binding EF hand family protein	Green ontogenesis	
ppa002110m	1.69	0.0249804	hypothetical protein 1	Green ontogenesis	
ppa003977m	1.69	0.041264286	3-ketoacyl-CoA synthase 1	Green ontogenesis	
ppa009035m	1.68	0.000412683	phosphate transporter 3;3	Green ontogenesis	
ppa005583m	1.68	0.000836964	Transmembrane amino acid transporter family protein	Green ontogenesis	
ppa018319m	1.68	0.002596451	Major facilitator superfamily protein	Green ontogenesis	
ppa005575m	1.68	0.012226838	CBL-interacting protein kinase 25	Green ontogenesis	
ppa004036m	1.68	0.0003519	aldehyde dehydrogenase 2B4	Green ontogenesis	
ppa014824m	1.68	0.041790001	wall associated kinase-like 1	Green ontogenesis	
ppa011329m	1.67	0.038996895	SSXT family protein	Green ontogenesis	
ppa004484m	1.67	0.000165125	beta glucosidase 11	Green ontogenesis	
ppa004652m	1.67	0.002151548	Major facilitator superfamily protein	Green ontogenesis	
ppa006989m	1.67	0.000399037	zinc transporter 4 precursor	Green ontogenesis	
ppa009011m	1.67	0.026890281	F-box family protein	Green ontogenesis	
ppa007040m	1.66	0.004190735	NADPH-dependent thioredoxin reductase A	Green ontogenesis	
ppa025196m	1.66	0.00048556	Cytochrome P450 superfamily protein	Green ontogenesis	
ppa011602m	1.66	0.026746819	Ribosomal protein L34e superfamily protein	Green ontogenesis	
ppa008128m	1.66	0.009559612	alpha/beta-Hydrolases superfamily protein	Green ontogenesis	
ppa009218m	1.65	0.001309569	P-loop containing nucleoside triphosphate hydrolases superfamily	Green ontogenesis	
ppa005605m	1.65	0.01138787	DegP protease 1	Green ontogenesis	
ppa008210m	1.65	0.001873193	adenosine kinase 2	Green ontogenesis	
ppa009160m	1.64	0.028480938	alpha/beta-Hydrolases superfamily protein	Green ontogenesis	
ppa020466m	1.64	0.001631799	FAD/NAD(P)-binding oxidoreductase family protein	Green ontogenesis	
ppa001417m	1.64	0.036892716	ARM repeat superfamily protein	Green ontogenesis	
ppa006458m	1.64	0.045471937	glutamate dehydrogenase 2	Green ontogenesis	
ppa020866m	1.64	0.038120158	embryo defective 2170	Green ontogenesis	
ppa009106m	1.64	0.020707009	Drug/metabolite transporter superfamily protein	Green ontogenesis	
ppa004845m	1.63	0.043351175	ammonium transporter 2	Green ontogenesis	
ppa004608m	1.63	0.022321012	Senescence/dehydration-associated protein-related	Green ontogenesis	
ppa025186m	1.63	0.001480703	Protein kinase superfamily protein	Green ontogenesis	
ppa004133m	1.63	0.014980357	allene oxide synthase	Green ontogenesis	
ppa003247m	1.63	0.008483011	senescence-associated gene 101	Green ontogenesis	
ppa008795m	1.63	0.043006038	alpha/beta-Hydrolases superfamily protein	Green ontogenesis	
ppa001577m	1.62	0.003959515	lectin protein kinase family protein	Green ontogenesis	
ppa003335m	1.62	0.002103065	GLN phosphoribosyl pyrophosphate amidotransferase 2	Green ontogenesis	
ppa005268m	1.62	0.003775176	polyamine oxidase 4	Green ontogenesis	
ppa007064m	1.62	0.000872438	Protein kinase superfamily protein	Green ontogenesis	
ppa008441m	1.62	0.02432553	winged-helix DNA-binding transcription factor family protein	Green ontogenesis	
ppa001462m	1.61	0.002319613	Protein of unknown function (DUF3741)	Green ontogenesis	
ppa005574m	1.61	0.009287624	fatty acid desaturase 8	Green ontogenesis	
ppa006144m	1.61	0.001596228	pyrimidine 1	Green ontogenesis	
ppa005752m	1.61	0.004267433	peptidase M20/M25/M40 family protein	Green ontogenesis	
ppa002082m	1.61	0.015102726	auxin response factor 16	Green ontogenesis	
ppa005456m	1.61	0.003650188	SecY protein transport family protein	Green ontogenesis	
ppa018486m	1.61	0.011733708	UDP-glucosyl transferase 85A5	Green ontogenesis	
ppa022600m	1.60	0.0017335	Protein kinase superfamily protein	Green ontogenesis	
ppa003354m	1.60	0.008826275	Phosphoglucomutase/phosphomannomutase family protein	Green ontogenesis	
ppa018651m	1.60	0.033392238		Green ontogenesis	
ppa002155m	1.60	0.029211158	P-loop containing nucleoside triphosphate hydrolases superfamily	Green ontogenesis	
ppa013344m	1.60	0.003295397		Green ontogenesis	
ppa005421m	1.60	0.008123246	peroxisomal 3-ketoacyl-CoA thiolase 3	Green ontogenesis	
ppa022428m	1.60	0.032222955	DNA replication protein-related	Green ontogenesis	
ppa005005m	1.59	0.006378718	AAA-ATPase 1	Green ontogenesis	
ppa006733m	1.59	0.011884012	Protein phosphatase 2C family protein	Green ontogenesis	
ppa009500m	1.59	0.017564411	Protein of unknown function (DUF1645)	Green ontogenesis	
ppa005910m	1.59	0.018559253	hydroxycinnamoyl-CoA shikimate/quinate hydroxycinnamoyl trans	Green ontogenesis	Secondary Metabolism / Phenylpropanoids - Biotic Stress / Secondary metabolism involved into stress
ppa002793m	1.59	0.007051345	Plant protein of unknown function (DUF827)	Green ontogenesis	
ppa012858m	1.59	0.015056779	Bifunctional inhibitor/lipid-transfer protein/seed storage 2S albumi	Green ontogenesis	
ppa002122m	1.59	0.003268427	oligopeptide transporter 7	Green ontogenesis	
ppa015751m	1.58	0.001136153	UDP-Glycosyltransferase superfamily protein	Green ontogenesis	
ppa006338m	1.58	0.013520906	UDP-Glycosyltransferase superfamily protein	Green ontogenesis	
ppa005144m	1.58	0.012854035	Major facilitator superfamily protein	Green ontogenesis	
ppa006780m	1.58	0.000927187	NAD(P)-binding Rossmann-fold superfamily protein	Green ontogenesis	
ppa005784m	1.58	0.002151548	Major facilitator superfamily protein	Green ontogenesis	
ppa011044m	1.58	0.001244055	F-box/RNI-like superfamily protein	Green ontogenesis	
ppa006322m	1.58	0.009969838	alpha/beta-Hydrolases superfamily protein	Green ontogenesis	
ppa024083m	1.58	0.000724297	UDP-glucosyl transferase 71D1	Green ontogenesis	
ppa023851m	1.57	0.007733624	UDP-glucosyl transferase 85A2	Green ontogenesis	
ppa005052m	1.57	0.010110676	UDP-glucosyl transferase 71B6	Green ontogenesis	
ppa015127m	1.57	0.007008449	UDP-glucosyl transferase 88A1	Green ontogenesis	
ppa025335m	1.57	0.04523078	response regulator 9	Green ontogenesis	
ppa005326m	1.57	0.040664143	PATATIN-like protein 6	Green ontogenesis	
ppa018037m	1.57	0.000742765	cationic amino acid transporter 5	Green ontogenesis	
ppa007078m	1.57	0.011326144	Calcineurin-like metallo-phosphoesterase superfamily protein	Green ontogenesis	
ppa004658m	1.56	0.006694596	Major facilitator superfamily protein	Green ontogenesis	
ppa018960m	1.56	0.024024493	NAD(P)-linked oxidoreductase superfamily protein	Green ontogenesis	
ppa027017m	1.56	0.004622112	RING/FYVE/PHD zinc finger superfamily protein	Green ontogenesis	
ppa010207m	1.56	0.006084035	alanine-tRNA ligases;nucleic acid binding;ligases, forming aminoa	Green ontogenesis	
ppa019904m	1.55	0.005180441	HXXXD-type acyl-transferase family protein	Green ontogenesis	Secondary metabolism / Anthocyanins - Biotic Stress / Secondary metabolism involved into stress
ppa010301m	1.55	0.003959515	VQ motif-containing protein	Green ontogenesis	
ppa017510m	1.55	0.008233103	Protein kinase superfamily protein	Green ontogenesis	
ppa005271m	1.55	0.010666715	Ankyrin repeat family protein	Green ontogenesis	
ppa014019m	1.55	0.021181742		Green ontogenesis	
ppa006004m	1.55	0.030482821	Galactose oxidase/kelch repeat superfamily protein	Green ontogenesis	
ppa003203m	1.54	0.001641332	MAC/Perforin domain-containing protein	Green ontogenesis	

ppa007820m	1.54	0.00270284	Alternative oxidase family protein	Green ontogenesis	
ppa024222m	1.54	0.009422216	RNA-binding (RRM/RBD/RNP motifs) family protein	Green ontogenesis	
ppa013537m	1.54	0.015131782	Protein of unknown function (DUF3511)	Green ontogenesis	
ppa006635m	1.54	0.004215141	FAD/NAD(P)-binding oxidoreductase family protein	Green ontogenesis	
ppa026639m	1.54	0.009483696	phospholipase D beta 1	Green ontogenesis	
ppa003061m	1.53	0.005567343	purple acid phosphatase 27	Green ontogenesis	
ppa025178m	1.53	0.019211268	basic helix-loop-helix (bHLH) DNA-binding superfamily protein	Green ontogenesis	
ppa013498m	1.53	0.043182602		Green ontogenesis	
ppa013570m	1.53	0.006211218	SPIRAL1-like1	Green ontogenesis	
ppa003236m	1.53	0.006018153	Domain of unknown function (DUF23)	Green ontogenesis	
ppa000736m	1.53	0.025026815	lipase class 3 family protein	Green ontogenesis	
ppa012897m	1.53	0.043945419		Green ontogenesis	
ppa009357m	1.53	0.000263303	Sec14p-like phosphatidylinositol transfer family protein	Green ontogenesis	
ppa013296m	1.53	0.027101239		Green ontogenesis	
ppa006627m	1.53	0.046025379	alpha/beta-Hydrolases superfamily protein	Green ontogenesis	
ppa011502m	1.52	0.00622889	basic helix-loop-helix (bHLH) DNA-binding superfamily protein	Green ontogenesis	
ppa011300m	1.52	0.013768439	Calcium-dependent lipid-binding (CaLB domain) family protein	Green ontogenesis	
ppa004999m	1.52	0.000768548	cytochrome P450, family 716, subfamily A, polypeptide 1	Green ontogenesis	
ppa012801m	1.52	0.031999238	farnesylated protein 6	Green ontogenesis	
ppa001113m	1.52	0.010050112	nitrate reductase 1	Green ontogenesis	
ppa004147m	1.52	0.033706822	Major facilitator superfamily protein	Green ontogenesis	
ppa019354m	1.51	0.000821882	UDP-glucosyl transferase 73B3	Green ontogenesis	
ppa023930m	1.51	0.002156459	PR5-like receptor kinase	Green ontogenesis	
ppa014789m	1.51	0.005872477	SCARECROW-like 14	Green ontogenesis	
ppa008361m	1.51	0.040703531	C2H2 and C2HC zinc fingers superfamily protein	Green ontogenesis	
ppa000817m	1.51	0.000399037	TUDOR-SN protein 1	Green ontogenesis	
ppa018458m	1.50	0.024618403	Long-chain fatty alcohol dehydrogenase family protein	Green ontogenesis	
ppa011274m	1.50	0.046875758	Protein of unknown function (DUF1442)	Green ontogenesis	
ppa014227m	1.50	0.008827818		Green ontogenesis	
ppa001858m	1.50	0.000841883		Green ontogenesis	
ppa003607m	1.50	0.005240269	AGC (cAMP-dependent, cGMP-dependent and protein kinase C)	Green ontogenesis	
ppa019965m	1.50	0.00208723	cytochrome P450, family 81, subfamily D, polypeptide 8	Green ontogenesis	
ppa007463m	1.50	0.008137531	Mitochondrial substrate carrier family protein	Green ontogenesis	
ppa011525m	1.50	0.032683516	salt tolerance homolog2	Green ontogenesis	
ppa021403m	1.50	0.0492128	Concanavalin A-like lectin protein kinase family protein	Green ontogenesis	
ppa015982m	1.50	0.00556618	Leucine-rich repeat transmembrane protein kinase	Green ontogenesis	
ppa005750m	1.50	0.007328351	XB3 ortholog 1 in Arabidopsis thaliana	Green ontogenesis	
ppa002702m	1.50	0.004324641	Protein kinase superfamily protein	Green ontogenesis	
ppa004936m	1.50	0.002778435	amino acid permease 3	Green ontogenesis	
ppa001891m	1.49	0.016233526	glutamate receptor 2.1	Green ontogenesis	
ppa024441m	1.49	0.022883297	HXXXD-type acyl-transferase family protein	Green ontogenesis	Secondary Metabolism / Phenylpropanoids - Biotic Stress / Secondary metabolism involved into stress
ppa002540m	1.49	0.008507916	AMP-dependent synthetase and ligase family protein	Green ontogenesis	
ppa015538m	1.49	0.006479243	mitogen-activated protein kinase phosphatase 1	Green ontogenesis	
ppa005851m	1.49	0.004849997	phytoene desaturation 1	Green ontogenesis	Biotic Stress / Secondary metabolism involved into stress
ppa017182m	1.49	0.001129143	Zinc-binding dehydrogenase family protein	Green ontogenesis	
ppa000741m	1.49	0.010110676	Leucine-rich repeat transmembrane protein kinase	Green ontogenesis	
ppa012075m	1.49	0.010788933	RING/U-box superfamily protein	Green ontogenesis	
ppa008549m	1.48	0.008873016	Calcineurin-like metallo-phosphoesterase superfamily protein	Green ontogenesis	
ppa026850m	1.48	0.031501383	Polyketide cyclase/dehydrase and lipid transport superfamily prot	Green ontogenesis	
ppa019342m	1.48	0.008261194	wall associated kinase-like 1	Green ontogenesis	
ppa023976m	1.48	0.025488053	Major facilitator superfamily protein	Green ontogenesis	
ppa005670m	1.48	0.015650814	Protein kinase superfamily protein	Green ontogenesis	
ppa019738m	1.48	0.024216313	Pheophorbide a oxygenase family protein with Rieske [2Fe-2S] d	Green ontogenesis	
ppa003884m	1.48	0.006018153	Leucine-rich repeat transmembrane protein kinase	Green ontogenesis	
ppa008566m	1.48	0.033127701	WRKY DNA-binding protein 40	Green ontogenesis	
ppa010504m	1.48	0.000316182		Green ontogenesis	
ppa018557m	1.48	0.014838433	UDP-glucosyl transferase 71B6	Green ontogenesis	
ppa014548m	1.47	0.001659782	Low temperature and salt responsive protein family	Green ontogenesis	
ppa008605m	1.47	0.041790001		Green ontogenesis	
ppa012698m	1.47	0.041797236	Calcium-binding EF-hand family protein	Green ontogenesis	
ppa007307m	1.47	0.008873016	isocitrate dehydrogenase 1	Green ontogenesis	
ppa007030m	1.47	0.023696292	OSBP(oxysterol binding protein)-related protein 4C	Green ontogenesis	
ppa011001m	1.47	0.010233567	Proteasome component (PCI) domain protein	Green ontogenesis	
ppa005043m	1.47	0.002782129	UDP-glucosyl transferase 85A2	Green ontogenesis	
ppa012552m	1.47	0.024070127	Acid phosphatase/vanadium-dependent haloperoxidase-related p	Green ontogenesis	
ppa008998m	1.47	0.0099641	GNS1/SUR4 membrane protein family	Green ontogenesis	
ppa003835m	1.47	0.010008478	NRAMP metal ion transporter 6	Green ontogenesis	
ppa003509m	1.46	0.002035718	plant U-box 13	Green ontogenesis	
ppa004833m	1.46	0.001662528	sodium/calcium exchanger family protein / calcium-binding EF ha	Green ontogenesis	
ppa006114m	1.46	0.018588631	Pectinacetylesterase family protein	Green ontogenesis	
ppa005561m	1.46	0.044227865	BTB/POZ domain with WD40/YVTN repeat-like protein	Green ontogenesis	
ppa004069m	1.46	0.001401745	calcium-dependent protein kinase 19	Green ontogenesis	
ppa000676m	1.46	0.01296326	Homeodomain-like protein	Green ontogenesis	
ppa005845m	1.46	0.023944911	Aldolase-type TIM barrel family protein	Green ontogenesis	
ppa003184m	1.46	0.001656463	methylenetetrahydrofolate reductase 2	Green ontogenesis	
ppa006420m	1.46	0.002596451	squalene synthase 1	Green ontogenesis	
ppa013101m	1.46	0.029832416		Green ontogenesis	
ppa002376m	1.46	0.000684419	hypothetical protein 1	Green ontogenesis	
ppa006413m	1.46	0.003859734	Auxin efflux carrier family protein	Green ontogenesis	
ppa000313m	1.45	0.033160329	P-glycoprotein 11	Green ontogenesis	
ppa019782m	1.45	0.016768974	Leucine-rich receptor-like protein kinase family protein	Green ontogenesis	
ppa005106m	1.45	0.001564867	UDP-Glycosyltransferase superfamily protein	Green ontogenesis	
ppa005879m	1.45	0.039614323	Magnesium transporter CorA-like family protein	Green ontogenesis	
ppa019352m	1.44	0.003327448	Malectin/receptor-like protein kinase family protein	Green ontogenesis	
ppa003278m	1.44	0.020162497	nitrate transporter 1:2	Green ontogenesis	
ppa002801m	1.44	0.015215923	S-adenosyl-L-methionine-dependent methyltransferases superfar	Green ontogenesis	
ppa007041m	1.44	0.009260967	high-affinity nickel-transport family protein	Green ontogenesis	
ppa009698m	1.44	0.025914257	NOD26-like intrinsic protein 1;2	Green ontogenesis	
ppa023486m	1.44	0.047218131	disease resistance protein (TIR-NBS-LRR class), putative	Green ontogenesis	Biotic Stress / PR-proteins
ppa008832m	1.43	0.014700481	RING/U-box superfamily protein	Green ontogenesis	
ppa018732m	1.43	0.024577892	HVA22 homologue A	Green ontogenesis	
ppa008890m	1.43	0.002703317	alpha/beta-Hydrolases superfamily protein	Green ontogenesis	
ppa012643m	1.43	0.010978846	eukaryotic elongation factor 5A-1	Green ontogenesis	
ppa013497m	1.42	0.038295653	Calcium-binding EF-hand family protein	Green ontogenesis	
ppa019013m	1.42	0.008098273	alpha/beta-Hydrolases superfamily protein	Green ontogenesis	
ppa021380m	1.42	0.041790001	tetratricopeptide repeat (TPR)-containing protein	Green ontogenesis	
ppa012489m	1.42	0.029327598		Green ontogenesis	
ppa016609m	1.42	0.011551616	Leucine-rich receptor-like protein kinase family protein	Green ontogenesis	
ppa025175m	1.42	0.003476401		Green ontogenesis	
ppa006905m	1.42	0.031094625	DNA-binding storekeeper protein-related transcriptional regulator	Green ontogenesis	
ppa003142m	1.42	0.005001196	NADP-malic enzyme 1	Green ontogenesis	
ppa004199m	1.42	0.000497826	cytochrome P450, family 87, subfamily A, polypeptide 6	Green ontogenesis	
ppa005088m	1.42	0.017701286		Green ontogenesis	
ppa007098m	1.42	0.021171861	fatty acid desaturase 2	Green ontogenesis	
ppa002311m	1.42	0.03703815	Cellulose-synthase-like C6	Green ontogenesis	
ppa013232m	1.41	0.003406751	cytochrome B5 isoform E	Green ontogenesis	
ppa004237m	1.41	0.017094022	alpha/beta-Hydrolases superfamily protein	Green ontogenesis	
ppa009455m	1.41	0.024394878	NAC domain containing protein 36	Green ontogenesis	
ppa011755m	1.41	0.015844922	AUX/IAA transcriptional regulator family protein	Green ontogenesis	
ppa005830m	1.41	0.000653388	UDP-Glycosyltransferase superfamily protein	Green ontogenesis	
ppa010660m	1.41	0.018290212	pathogenesis-related family protein	Green ontogenesis	
ppa015620m	1.41	0.000768596	Major facilitator superfamily protein	Green ontogenesis	
ppa012302m	1.40	0.008163501	DREB and EAR motif protein 3	Green ontogenesis	
ppb013789m	1.40	0.039035169	suppressor of npr1-1 constitutive 4	Green ontogenesis	
ppa015238m	1.40	0.020087878	autoinhibited Ca(2+)-ATPase, isoform 4	Green ontogenesis	
ppa009883m	1.40	0.014856038	Ras-related small GTP-binding family protein	Green ontogenesis	
ppa005590m	1.40	0.006018153	sterol methyltransferase 2	Green ontogenesis	
ppa005673m	1.40	0.044739208	Transducin family protein / WD-40 repeat family protein	Green ontogenesis	
ppa006242m	1.40	0.000831342	Auxin efflux carrier family protein	Green ontogenesis	
ppa002282m	1.40	0.016194296	acyl-CoA oxidase 2	Green ontogenesis	
ppa010258m	1.40	0.004544104	sphingoid base hydroxylase 2	Green ontogenesis	
ppa007470m	1.39	0.010110676	cation exchanger 5	Green ontogenesis	
ppa002085m	1.39	0.04984386	Tetratricopeptide repeat (TPR)-like superfamily protein	Green ontogenesis	
ppa006356m	1.39	0.005089874	cytosolic NADP+-dependent isocitrate dehydrogenase	Green ontogenesis	
ppa007193m	1.39	0.014700481	related to AP2 4	Green ontogenesis	
ppa006715m	1.39	0.029345025	Homeodomain-like superfamily protein	Green ontogenesis	
ppa001739m	1.38	0.024237945	Subtilase family protein	Green ontogenesis	
ppa012756m	1.38	0.035040179	Calcium-dependent lipid-binding (CaLB domain) family protein	Green ontogenesis	
ppa023414m	1.38	0.049180985		Green ontogenesis	
ppa019648m	1.38	0.00556618		Green ontogenesis	
ppa005550m	1.38	0.007033807	RNA-binding (RRM/RBD/RNP motifs) family protein	Green ontogenesis	
ppa009372m	1.38	0.000872438	atypical CYS HIS rich thioredoxin 4	Green ontogenesis	
ppa009526m	1.38	0.002742652	NAD(P)-binding Rossmann-fold superfamily protein	Green ontogenesis	
ppa004581m	1.38	0.004828722	fucosyltransferase 12	Green ontogenesis	
ppa000486m	1.38	0.030777766	Outer arm dynein light chain 1 protein	Green ontogenesis	
ppa002468m	1.38	0.049685603	OBP3-responsive gene 1	Green ontogenesis	
ppa003967m	1.38	0.006203574	DNA photolyase family protein	Green ontogenesis	
ppa008979m	1.38	0.013862504	myb domain protein r1	Green ontogenesis	
ppa013611m	1.37	0.014838433	Ggamma-subunit 1	Green ontogenesis	
ppa002619m	1.37	0.010240202	WRKY family transcription factor	Green ontogenesis	
ppa010530m	1.37	0.012701221	DNA-binding storekeeper protein-related	Green ontogenesis	
ppa004119m	1.37	0.038390573	Major facilitator superfamily protein	Green ontogenesis	
ppa003933m	1.37	0.009287367	exocyst subunit exo70 family protein F1	Green ontogenesis	
ppa003315m	1.37	0.01222021	nitrite reductase 1	Green ontogenesis	
ppa009794m	1.36	0.032426574	Phosphoglycerate mutase family protein	Green ontogenesis	
ppa008824m	1.36	0.023038189	EamA-like transporter family	Green ontogenesis	
ppa002524m	1.36	0.001596228	YELLOW STRIPE like 1	Green ontogenesis	
ppa002818m	1.36	0.010390279	receptor like protein 32	Green ontogenesis	

ppa001233m	1.36	0.000784343	*aminopeptidase M1*	Green ontogenesis	
ppa000250m	1.36	0.010487617		Green ontogenesis	
ppa007511m	1.36	0.004190735	*O-methyltransferase 1*	Green ontogenesis	
ppa005204m	1.35	0.001636449		Green ontogenesis	
ppa002701m	1.35	0.020152869	*SCARECROW-like 14*	Green ontogenesis	
ppa001267m	1.35	0.007765812	*U-box domain-containing protein kinase family protein*	Green ontogenesis	
ppa027068m	1.35	0.005734965	*transcription factor-related*	Green ontogenesis	
ppa004046m	1.35	0.008520341	*importin alpha isoform 4*	Green ontogenesis	
ppa005073m	1.35	0.007985871	*Family of unknown function (DUF716)*	Green ontogenesis	
ppa004679m	1.35	0.019957395	*Uncharacterised conserved protein UCP031088, alpha/beta hydro*	Green ontogenesis	
ppa006010m	1.35	0.004245952	*solanesyl diphosphate synthase 2*	Green ontogenesis	Biotic Stress / Secondary metabolism involved into stress
ppa004599m	1.34	0.030817416	*Transducin/WD40 repeat-like superfamily protein*	Green ontogenesis	
ppa001988m	1.34	0.0417195		Green ontogenesis	
ppa018416m	1.34	0.010274587		Green ontogenesis	
ppa005085m	1.34	0.01628468	*BTB/POZ domain-containing protein*	Green ontogenesis	
ppa009450m	1.34	0.003863501	*small G protein family protein / RhoGAP family protein*	Green ontogenesis	
ppa000526m	1.34	0.002674361	*Regulator of chromosome condensation (RCC1) family with FYVE*	Green ontogenesis	
ppa003978m	1.34	0.006479243	*SKP1/ASK1-interacting protein 2*	Green ontogenesis	
ppa004030m	1.33	0.004527935	*plant intracellular ras group-related LRR 4*	Green ontogenesis	
ppa012651m	1.33	0.005376196	*MLP-like protein 423*	Green ontogenesis	
ppa013719m	1.33	0.035951348		Green ontogenesis	
ppa007591m	1.33	0.024089543	*Protein of unknown function (DUF1644)*	Green ontogenesis	
ppa005471m	1.33	0.049066275	*UDP-Glycosyltransferase superfamily protein*	Green ontogenesis	
ppa017667m	1.33	0.008163501	*Protein kinase superfamily protein*	Green ontogenesis	
ppa003527m	1.33	0.007967779	*Major facilitator superfamily protein*	Green ontogenesis	
ppa024188m	1.33	0.025026815		Green ontogenesis	
ppa005219m	1.32	0.029578621	*homogentisate 1,2-dioxygenase*	Green ontogenesis	
ppa006974m	1.32	0.025831704	*ABI five binding protein 2*	Green ontogenesis	
ppa001607m	1.32	0.001622986	*ATPase, AAA-type, CDC48 protein*	Green ontogenesis	
ppa009997m	1.32	0.023038189		Green ontogenesis	
ppa008269m	1.32	0.027611539	*2-oxoglutarate (2OG) and Fe(II)-dependent oxygenase superfami*	Green ontogenesis	
ppa006347m	1.32	0.0106433	*Protein kinase superfamily protein*	Green ontogenesis	
ppa003240m	1.31	0.003939106	*phosphoinositide 4-kinase gamma 4*	Green ontogenesis	
ppa016716m	1.31	0.033160329		Green ontogenesis	
ppa018218m	1.31	0.006506402	*alpha/beta-Hydrolases superfamily protein*	Green ontogenesis	
ppa009596m	1.31	0.005558571	*tetraspanin3*	Green ontogenesis	
ppa007482m	1.31	0.014058244	*endoplasmic reticulum oxidoreductins 1*	Green ontogenesis	
ppa002064m	1.31	0.009980484	*Protein kinase superfamily protein*	Green ontogenesis	
ppa027196m	1.31	0.046407189	*2-oxoglutarate (2OG) and Fe(II)-dependent oxygenase superfami*	Green ontogenesis	
ppa013626m	1.31	0.002364658	*cytochrome c-2*	Green ontogenesis	
ppa026411m	1.31	0.008400976	*Plant protein of unknown function (DUF247)*	Green ontogenesis	
ppa006596m	1.31	0.001823737	*PHYTOENE SYNTHASE*	Green ontogenesis	Biotic Stress / Secondary metabolism involved into stress
ppa004032m	1.31	0.017334182	*IQ-domain 13*	Green ontogenesis	
ppa004808m	1.31	0.006492096	*Protein kinase superfamily protein*	Green ontogenesis	
ppa007825m	1.31	0.001823737	*Peroxisomal membrane 22 kDa (Mpv17/PMP22) family protein*	Green ontogenesis	
ppa006178m	1.31	0.018402046	*Histone H3 K4-specific methyltransferase SET7/9 family protein*	Green ontogenesis	
ppa025893m	1.30	0.01222021	*glutamate receptor 1.4*	Green ontogenesis	
ppa012254m	1.30	0.043528968	*CAMV movement protein interacting protein 7*	Green ontogenesis	
ppa003923m	1.30	0.002774255	*dihydrosphingosine phosphate lyase*	Green ontogenesis	
ppa013495m	1.30	0.006949093	*Protein of unknown function (DUF3511)*	Green ontogenesis	
ppa022950m	1.30	0.030533505	*Protein kinase family protein with leucine-rich repeat domain*	Green ontogenesis	
ppa021086m	1.30	0.005981281	*oligopeptide transporter 4*	Green ontogenesis	
ppa019430m	1.30	0.001652525	*UDP-glucosyl transferase 78D2*	Green ontogenesis	
ppa025698m	1.30	0.002449548	*presequence protease 1*	Green ontogenesis	
ppa018536m	1.30	0.019485181	*GRAM domain-containing protein / ABA-responsive protein-relate*	Green ontogenesis	
ppa002575m	1.30	0.036793334	*hydroxyproline-rich glycoprotein family protein*	Green ontogenesis	
ppa011736m	1.29	0.003476401		Green ontogenesis	
ppa012490m	1.29	0.008746605	*Adenine nucleotide alpha hydrolases-like superfamily protein*	Green ontogenesis	
ppa012679m	1.29	0.013964521	*Iron-sulphur cluster biosynthesis family protein*	Green ontogenesis	
ppa007663m	1.29	0.016626277	*phytochrome-associated protein 1*	Green ontogenesis	
ppa007190m	1.29	0.003904003	*Protein phosphatase 2C family protein*	Green ontogenesis	
ppa005892m	1.29	0.00854102	*Major facilitator superfamily protein*	Green ontogenesis	
ppa017679m	1.29	0.012217693	*Ribosomal RNA processing Brix domain protein*	Green ontogenesis	
ppa004544m	1.29	0.00665915	*cinnamate-4-hydroxylase*	Green ontogenesis	
ppa015276m	1.28	0.021801792	*nodulin MtN21 /EamA-like transporter family protein*	Green ontogenesis	
ppa013593m	1.28	0.006749842	*RPM1-interacting protein 4 (RIN4) family protein*	Green ontogenesis	
ppa003959m	1.28	0.004983547	*phosphate transporter 1;7*	Green ontogenesis	
ppa004792m	1.28	0.014722034	*photolyase 1*	Green ontogenesis	
ppa002321m	1.28	0.01281596	*Protein kinase superfamily protein*	Green ontogenesis	
ppa011228m	1.28	0.030743716	*Tetraspanin family protein*	Green ontogenesis	
ppa010020m	1.28	0.001928443		Green ontogenesis	
ppa010087m	1.27	0.022868501	*Thioredoxin superfamily protein*	Green ontogenesis	
ppa003986m	1.27	0.001641016	*phosphofructokinase 4*	Green ontogenesis	
ppa006652m	1.27	0.006286591	*pyruvate dehydrogenase complex E1 alpha subunit*	Green ontogenesis	
ppa005933m	1.27	0.003681314	*Zn-dependent exopeptidases superfamily protein*	Green ontogenesis	
ppa019886m	1.27	0.002538838	*cytochrome P450, family 81, subfamily D, polypeptide 8*	Green ontogenesis	
ppa004673m	1.27	0.004336305	*glutathione-disulfide reductase*	Green ontogenesis	
ppa006058m	1.27	0.014218821	*Protein phosphatase 2C family protein*	Green ontogenesis	
ppa009511m	1.26	0.007328351	*secretory carrier 3*	Green ontogenesis	
ppa007342m	1.26	0.043638424	*2-oxoglutarate (2OG) and Fe(II)-dependent oxygenase superfami*	Green ontogenesis	
ppa001698m	1.26	0.029929929	*Copper amine oxidase family protein*	Green ontogenesis	
ppa010192m	1.26	0.015223639	*SGNH hydrolase-type esterase superfamily protein*	Green ontogenesis	
ppa005270m	1.26	0.002112393	*6-phosphogluconate dehydrogenase family protein*	Green ontogenesis	
ppa010999m	1.26	0.003449834	*Calcium-binding EF-hand family protein*	Green ontogenesis	
ppa015545m	1.26	0.008404357	*glucose-6-phosphate dehydrogenase 2*	Green ontogenesis	
ppa005916m	1.26	0.01771093	*acyl-CoA oxidase 4*	Green ontogenesis	
ppa008771m	1.25	0.024691103	*Protein kinase superfamily protein*	Green ontogenesis	
ppa007869m	1.25	0.017713847	*UDP-glucosyl transferase 71C3*	Green ontogenesis	
ppa007348m	1.25	0.005673141	*SPFH/Band 7/PHB domain-containing membrane-associated pro*	Green ontogenesis	
ppa013046m	1.25	0.006714653	*basic region/leucine zipper motif 53*	Green ontogenesis	
ppa020329m	1.25	0.011342191	*PR5-like receptor kinase*	Green ontogenesis	
ppa010978m	1.25	0.008434875	*RING/U-box superfamily protein*	Green ontogenesis	
ppa012585m	1.24	0.01448622	*Adenine nucleotide alpha hydrolases-like superfamily protein*	Green ontogenesis	
ppa000235m	1.24	0.007620004	*pleiotropic drug resistance 6*	Green ontogenesis	
ppa008163m	1.24	0.008980774	*NAD(P)-linked oxidoreductase superfamily protein*	Green ontogenesis	
ppa005187m	1.24	0.003107752	*UDP-Glycosyltransferase superfamily protein*	Green ontogenesis	
ppa022273m	1.24	0.018087793	*cation exchanger 2*	Green ontogenesis	
ppa006293m	1.24	0.019340177	*Senescence/dehydration-associated protein-related*	Green ontogenesis	
ppa000948m	1.24	0.003110718	*ABC1 family protein*	Green ontogenesis	
ppa021651m	1.24	0.046662244	*Toll-Interleukin-Resistance (TIR) domain family protein*	Green ontogenesis	
ppa003678m	1.24	0.002414148	*glutathione reductase*	Green ontogenesis	
ppa006881m	1.24	0.011534092	*zinc finger (C3HC4-type RING finger) family protein*	Green ontogenesis	
ppa011248m	1.23	0.009104666		Green ontogenesis	
ppa025712m	1.23	0.024665715	*receptor kinase 3*	Green ontogenesis	
ppa004805m	1.23	0.029736546	*MATE efflux family protein*	Green ontogenesis	
ppa002916m	1.23	0.01397337	*Protein of unknown function (DUF668)*	Green ontogenesis	
ppa004113m	1.23	0.003038657	*MATE efflux family protein*	Green ontogenesis	
ppa006840m	1.23	0.003181723	*ubiquitin-specific protease 12*	Green ontogenesis	
ppa022623m	1.23	0.009043473	*glutamate receptor 2.8*	Green ontogenesis	
ppa000672m	1.23	0.014214761	*autoinhibited Ca2+-ATPase 11*	Green ontogenesis	
ppa009881m	1.23	0.016853149	*RING/FYVE/PHD zinc finger superfamily protein*	Green ontogenesis	
ppa007118m	1.23	0.012918131	*Protein of unknown function (DUF793)*	Green ontogenesis	
ppa002315m	1.23	0.004872145	*glutamine-fructose-6-phosphate transaminase (isomerizing)s;sug*	Green ontogenesis	
ppa007090m	1.22	0.002820431	*NAC domain containing protein 28*	Green ontogenesis	
ppa018615m	1.22	0.04913784	*Major facilitator superfamily protein*	Green ontogenesis	
ppa006466m	1.22	0.024873976	*phospholipase A 2A*	Green ontogenesis	
ppa008131m	1.22	0.01253152	*Protein of unknown function (DUF803)*	Green ontogenesis	
ppa005800m	1.22	0.003553862	*Transducin family protein / WD-40 repeat family protein*	Green ontogenesis	
ppa010419m	1.22	0.00257221	*Rubber elongation factor protein (REF)*	Green ontogenesis	
ppa017955m	1.22	0.020308693	*Leucine-rich repeat receptor-like protein kinase family protein*	Green ontogenesis	
ppa001152m	1.21	0.019160065	*Leucine-rich repeat transmembrane protein kinase*	Green ontogenesis	
ppa005644m	1.21	0.004695941	*tubulin beta-1 chain*	Green ontogenesis	
ppa018232m	1.21	0.002778435	*NAD(P)-binding Rossmann-fold superfamily protein*	Green ontogenesis	
ppa000499m	1.21	0.042516331	*Leucine-rich repeat receptor-like protein kinase family protein*	Green ontogenesis	
ppa002693m	1.21	0.008972294	*receptor serine/threonine kinase, putative*	Green ontogenesis	
ppa005756m	1.21	0.011827908	*DNAJ heat shock N-terminal domain-containing protein*	Green ontogenesis	
ppa010940m	1.21	0.031900111	*Calcium-binding EF-hand family protein*	Green ontogenesis	
ppa020318m	1.21	0.009738624	*Ribosomal RNA processing Brix domain protein*	Green ontogenesis	
ppa021437m	1.21	0.019446275	*Transmembrane amino acid transporter family protein*	Green ontogenesis	
ppa019851m	1.21	0.037717487	*xylosyltransferase 1*	Green ontogenesis	
ppa005909m	1.21	0.004987322	*Protein of unknown function (DUF1350)*	Green ontogenesis	
ppa006806m	1.21	0.006018153	*Pyridoxal phosphate (PLP)-dependent transferases superfamily p*	Green ontogenesis	
ppa007456m	1.21	0.020112529	*Core-2/I-branching beta-1,6-N-acetylglucosaminyltransferase fam*	Green ontogenesis	
ppa002977m	1.21	0.010487617	*ABC transporter 1*	Green ontogenesis	
ppa007664m	1.21	0.014729317	*NAD(P)-linked oxidoreductase superfamily protein*	Green ontogenesis	
ppa000531m	1.21	0.041599251	*DegP protease 7*	Green ontogenesis	
ppa002133m	1.21	0.006885816	*Protein kinase superfamily protein*	Green ontogenesis	
ppa004374m	1.21	0.032426574	*P-loop containing nucleoside triphosphate hydrolases superfamily*	Green ontogenesis	
ppa004890m	1.21	0.03198184	*long chain base2*	Green ontogenesis	
ppa003364m	1.20	0.008307117	*peptide transporter 5*	Green ontogenesis	
ppa002696m	1.20	0.004872268	*MIRO-related GTP-ase 2*	Green ontogenesis	
ppa019630m	1.20	0.029466408	*Malectin/receptor-like protein kinase family protein*	Green ontogenesis	
ppa013226m	1.20	0.036568664		Green ontogenesis	
ppa006476m	1.20	0.014248731	*Nucleotide-diphospho-sugar transferases superfamily protein*	Green ontogenesis	
ppa002604m	1.20	0.047538544	*POX (plant homeobox) family protein*	Green ontogenesis	
ppa001010m	1.20	0.00361244	*Leucine-rich receptor-like protein kinase family protein*	Green ontogenesis	
ppa012013m	1.20	0.0489891		Green ontogenesis	
ppa002166m	1.19	0.032883779	*SCARECROW-like 14*	Green ontogenesis	

ppa011096m	1.19	0.013264638	*Putative thiol-disulphide oxidoreductase DCC*	Green ontogenesis	
ppa003310m	1.19	0.005103312	*SCARECROW-like 1*	Green ontogenesis	
ppa000737m	1.19	0.021914707	*Protein kinase family protein with leucine-rich repeat domain*	Green ontogenesis	
ppa008404m	1.18	0.039438535	*homocysteine S-methyltransferase 3*	Green ontogenesis	
ppa009906m	1.18	0.046135658	*NAD(P)-binding Rossmann-fold superfamily protein*	Green ontogenesis	
ppa013551m	1.18	0.006630492	*membrane-anchored ubiquitin-fold protein 1 precursor*	Green ontogenesis	
ppa008653m	1.18	0.042297228	*peroxisomal adenine nucleotide carrier 1*	Green ontogenesis	
ppa007772m	1.18	0.007832176	*glutamine synthase clone R1*	Green ontogenesis	
ppa000963m	1.18	0.00430345		Green ontogenesis	
ppa008675m	1.18	0.010815873	*BES1/BZR1 homolog 4*	Green ontogenesis	
ppa000921m	1.18	0.019245134	*Leucine-rich repeat protein kinase family protein*	Green ontogenesis	
ppa011412m	1.17	0.004482878	*RAB GTPase homolog B1C*	Green ontogenesis	
ppa004215m	1.17	0.039437724	*AMP-dependent synthetase and ligase family protein*	Green ontogenesis	
ppa006708m	1.16	0.010007236	*Mitochondrial substrate carrier family protein*	Green ontogenesis	
ppa003670m	1.16	0.017969012	*cytosolic invertase 2*	Green ontogenesis	
ppa003103m	1.16	0.021386631	*CDPK-related kinase 3*	Green ontogenesis	
ppa005221m	1.16	0.019146701	*high affinity nitrate transporter 2.7*	Green ontogenesis	
ppa009114m	1.16	0.010110676	*protein phosphatase 2A-2*	Green ontogenesis	
ppa006411m	1.15	0.004913121	*Glucose-6-phosphate/phosphate translocator-related*	Green ontogenesis	
ppa008145m	1.15	0.020462061	*NAD(P)-linked oxidoreductase superfamily protein*	Green ontogenesis	
ppa005193m	1.15	0.004695941	*Citrate synthase family protein*	Green ontogenesis	
ppa009700m	1.15	0.019545464	*WRKY DNA-binding protein 11*	Green ontogenesis	
ppa007710m	1.15	0.010846066	*Protein kinase superfamily protein*	Green ontogenesis	
ppa009115m	1.15	0.045898657	*Nucleotide-sugar transporter family protein*	Green ontogenesis	
ppa003104m	1.15	0.022659359	*ENTH/VHS/GAT family protein*	Green ontogenesis	
ppa009560m	1.15	0.004695941	*NAD(P)-binding Rossmann-fold superfamily protein*	Green ontogenesis	
ppa026644m	1.15	0.033968353		Green ontogenesis	
ppa009324m	1.15	0.026615996	*short-chain dehydrogenase-reductase B*	Green ontogenesis	
ppa004265m	1.15	0.012134352	*Eukaryotic aspartyl protease family protein*	Green ontogenesis	
ppa011854m	1.14	0.008601011	*Cytidine/deoxycytidylate deaminase family protein*	Green ontogenesis	
ppa006398m	1.14	0.038193886	*glutamate decarboxylase*	Green ontogenesis	
ppa005694m	1.14	0.011114153	*Fatty acid/sphingolipid desaturase*	Green ontogenesis	
ppa004655m	1.14	0.020429789	*Glycosyltransferase family 61 protein*	Green ontogenesis	
ppa013234m	1.14	0.024216313	*cytochrome B5 isoform E*	Green ontogenesis	
ppa003147m	1.14	0.006832762	*Tetratricopeptide repeat (TPR)-like superfamily protein*	Green ontogenesis	
ppa006074m	1.14	0.013353805	*CBS domain-containing protein with a domain of unknown functio*	Green ontogenesis	
ppa003675m	1.14	0.043084224	*GRAS family transcription factor*	Green ontogenesis	
ppa005315m	1.14	0.017400881	*aspartate aminotransferase 3*	Green ontogenesis	
ppa008853m	1.13	0.035775112	*PLAC8 family protein*	Green ontogenesis	
ppa023287m	1.13	0.009288864	*alpha/beta-Hydrolases superfamily protein*	Green ontogenesis	
ppa001980m	1.13	0.019213058	*two-pore channel 1*	Green ontogenesis	
ppa009008m	1.13	0.011342191	*fatty acid desaturase A*	Green ontogenesis	
ppa008606m	1.13	0.008775002	*O-acetylserine (thiol) lyase (OAS-TL) isoform A1*	Green ontogenesis	
ppa005666m	1.13	0.039851892	*Transmembrane amino acid transporter family protein*	Green ontogenesis	
ppa002453m	1.13	0.010068286	*rhamnose biosynthesis 1*	Green ontogenesis	
ppa004836m	1.13	0.006219281	*zinc finger (C3HC4-type RING finger) family protein*	Green ontogenesis	
ppa019188m	1.12	0.044968024	*Protein of unknown function (DUF1262)*	Green ontogenesis	
ppa006424m	1.12	0.040226984	*heptahelical protein 4*	Green ontogenesis	
ppa002208m	1.12	0.007814623	*mitogen-activated protein kinase kinase kinase 5*	Green ontogenesis	
ppa005977m	1.12	0.023038189	*Melibiase family protein*	Green ontogenesis	
ppa001215m	1.11	0.026615996	*extra-large GTP-binding protein 3*	Green ontogenesis	
ppa001400m	1.11	0.021530748	*protein kinase family protein*	Green ontogenesis	
ppa011226m	1.11	0.021667733	*RAB GTPase homolog A1C*	Green ontogenesis	
ppa007019m	1.11	0.020373133	*Protein kinase superfamily protein*	Green ontogenesis	
ppa004045m	1.11	0.018559253	*galacturonosyltransferase 14*	Green ontogenesis	
ppa010070m	1.11	0.006517098	*methylthioadenosine nucleosidase 1*	Green ontogenesis	
ppa006582m	1.11	0.032683516	*alpha/beta-Hydrolases superfamily protein*	Green ontogenesis	
ppa002174m	1.11	0.006445507	*Protein kinase superfamily protein*	Green ontogenesis	
ppa003575m	1.11	0.00656793		Green ontogenesis	
ppa004809m	1.11	0.011827908	*magnesium transporter 4*	Green ontogenesis	
ppa009039m	1.11	0.038979679	*seven in absentia of Arabidopsis 2*	Green ontogenesis	
ppa017212m	1.11	0.015484705	*ABC2 homolog 9*	Green ontogenesis	
ppa011371m	1.11	0.022806771	*glutathione S-transferase TAU 19*	Green ontogenesis	Biotic Stress / Glutathione-S-Transferase
ppa003820m	1.10	0.032222955	*eukaryotic translation initiation factor 4B1*	Green ontogenesis	
ppa003795m	1.10	0.011163596	*calcium-dependent protein kinase 1*	Green ontogenesis	
ppa003745m	1.10	0.021174045	*sodium hydrogen exchanger 2*	Green ontogenesis	
ppa021762m	1.10	0.007047683	*polyol/monosaccharide transporter 5*	Green ontogenesis	
ppa006831m	1.10	0.019859021	*Aldolase superfamily protein*	Green ontogenesis	
ppa004230m	1.10	0.006758966		Green ontogenesis	
ppa011411m	1.10	0.030273299	*Rer1 family protein*	Green ontogenesis	
ppa005378m	1.10	0.020934517	*HOPW1-1-interacting 1*	Green ontogenesis	
ppa014377m	1.10	0.02442925		Green ontogenesis	
ppa011892m	1.10	0.011163596	*Protein of unknown function (DUF1685)*	Green ontogenesis	
ppa019571m	1.10	0.01200695	*Protein kinase family protein with leucine-rich repeat domain*	Green ontogenesis	
ppa023611m	1.10	0.031371364	*Major facilitator superfamily protein*	Green ontogenesis	
ppa006979m	1.09	0.015215923	*mevalonate kinase*	Green ontogenesis	Biotic Stress / Secondary metabolism involved into stress
ppa004388m	1.09	0.007653424	*Major Facilitator Superfamily with SPX (SYG1/Pho81/XPR1) dom*	Green ontogenesis	
ppa008027m	1.09	0.008460683	*serine carboxypeptidase-like 42*	Green ontogenesis	
ppa003468m	1.09	0.033799086	*cytochrome P450, family 78, subfamily A, polypeptide 7*	Green ontogenesis	
ppa004662m	1.09	0.039619466	*Tetratricopeptide repeat (TPR)-like superfamily protein*	Green ontogenesis	
ppa009683m	1.09	0.015036516	*Protein phosphatase 2C family protein*	Green ontogenesis	
ppa002083m	1.09	0.014456545	*FtsH extracellular protease family*	Green ontogenesis	
ppa008068m	1.09	0.009288864	*NAD(P)-linked oxidoreductase superfamily protein*	Green ontogenesis	
ppa001157m	1.09	0.008214002	*Malectin/receptor-like protein kinase family protein*	Green ontogenesis	
ppa011037m	1.09	0.014783772	*RING/U-box superfamily protein*	Green ontogenesis	
ppa010058m	1.09	0.01544422	*Galactose oxidase/kelch repeat superfamily protein*	Green ontogenesis	
ppa004616m	1.09	0.021880692	*hydroxyproline-rich glycoprotein family protein*	Green ontogenesis	
ppa003121m	1.09	0.008908473	*Protein of unknown function DUF2359, transmembrane*	Green ontogenesis	
ppa008875m	1.09	0.008077451	*alpha/beta-Hydrolases superfamily protein*	Green ontogenesis	
ppa011633m	1.09	0.013559266	*RAB GTPase homolog 1C*	Green ontogenesis	
ppa005640m	1.09	0.042936168	*HXXXD-type acyl-transferase family protein*	Green ontogenesis	Secondary Metabolism / Phenylpropanoids - Biotic Stress / Secondary metabolism involved into stress
ppa010445m	1.08	0.010110676	*PLATZ transcription factor family protein*	Green ontogenesis	
ppa005893m	1.08	0.012849108	*Transducin/WD40 repeat-like superfamily protein*	Green ontogenesis	
ppa006092m	1.08	0.01339701	*EamA-like transporter family*	Green ontogenesis	
ppa005883m	1.08	0.0359871	*Peptidase M20/M25/M40 family protein*	Green ontogenesis	
ppa002872m	1.07	0.046551266	*protein kinase family protein / peptidoglycan-binding LysM domai*	Green ontogenesis	
ppa012162m	1.07	0.013353805		Green ontogenesis	
ppa008032m	1.07	0.014553931	*UDP-D-glucose/UDP-D-galactose 4-epimerase 5*	Green ontogenesis	
ppa004411m	1.07	0.034401704	*Nucleotide-diphospho-sugar transferases superfamily protein*	Green ontogenesis	
ppa026732m	1.07	0.02955368	*cofactor of nitrate reductase and xanthine dehydrogenase 2*	Green ontogenesis	
ppa009234m	1.07	0.037686705	*Leucine-rich repeat (LRR) family protein*	Green ontogenesis	
ppa018029m	1.07	0.008980774	*NAD(P)-binding Rossmann-fold superfamily protein*	Green ontogenesis	
ppa005566m	1.07	0.010285876	*Major facilitator superfamily protein*	Green ontogenesis	
ppa009876m	1.07	0.009355779	*Ubiquitin system component Cue protein*	Green ontogenesis	
ppa023436m	1.07	0.008916329	*phosphate transporter 4;5*	Green ontogenesis	
ppa004703m	1.07	0.019940423	*Pyruvate kinase family protein*	Green ontogenesis	
ppa006468m	1.07	0.011601598	*alpha/beta-Hydrolases superfamily protein*	Green ontogenesis	
ppa003139m	1.06	0.044051346	*plant U-box 14*	Green ontogenesis	
ppa004097m	1.06	0.009294332	*Actin-like ATPase superfamily protein*	Green ontogenesis	
ppa001761m	1.06	0.009183705	*S-locus lectin protein kinase family protein*	Green ontogenesis	
ppa011254m	1.06	0.015056779	*soluble N-ethylmaleimide-sensitive factor adaptor protein 33*	Green ontogenesis	
ppa008434m	1.06	0.013332524		Green ontogenesis	
ppa004366m	1.06	0.032238489	*Actin cross-linking protein*	Green ontogenesis	
ppa005736m	1.06	0.017131629	*mannose-1-phosphate guanylyltransferase (GDP)s;GDP-galactos*	Green ontogenesis	
ppa000742m	1.06	0.045083432	*Leucine-rich repeat transmembrane protein kinase*	Green ontogenesis	
ppa010825m	1.06	0.016476265	*FKBP-like peptidyl-prolyl cis-trans isomerase family protein*	Green ontogenesis	
ppa003169m	1.06	0.015260558	*Phosphoinositide phosphatase family protein*	Green ontogenesis	
ppa008523m	1.06	0.018559253		Green ontogenesis	
ppa006141m	1.06	0.024661287	*EXS (ERD1/XPR1/SYG1) family protein*	Green ontogenesis	
ppa013773m	1.05	0.02034771	*Eukaryotic protein of unknown function (DUF872)*	Green ontogenesis	
ppa004016m	1.05	0.046714383	*non-specific phospholipase C1*	Green ontogenesis	
ppa004206m	1.05	0.022112105	*AFG1-like ATPase family protein*	Green ontogenesis	
ppa007265m	1.05	0.027336382	*2-oxoglutarate (2OG) and Fe(II)-dependent oxygenase superfami*	Green ontogenesis	
ppa003568m	1.05	0.024664717	*phosphatidate cytidylyltransferase family protein*	Green ontogenesis	
ppa009017m	1.05	0.014912999	*RP non-ATPase subunit 8A*	Green ontogenesis	
ppa008318m	1.05	0.013583273	*homeobox protein 6*	Green ontogenesis	
ppa005608m	1.05	0.027031595	*G-protein coupled receptors;GTPase activators*	Green ontogenesis	
ppa002105m	1.05	0.048271664	*Fibronectin type III domain-containing protein*	Green ontogenesis	
ppa005325m	1.05	0.013731991	*Major facilitator superfamily protein*	Green ontogenesis	
ppa023167m	1.05	0.021801792	*COP1-interacting protein 7*	Green ontogenesis	Biotic Stress / PR-proteins
ppa025214m	1.05	0.024664717	*F-box and associated interaction domains-containing protein*	Green ontogenesis	
ppa002752m	1.05	0.038211849	*ARP protein (REF)*	Green ontogenesis	
ppa008313m	1.04	0.033845396	*Nucleotide-diphospho-sugar transferases superfamily protein*	Green ontogenesis	
ppa005016m	1.04	0.017835981	*digalactosyl diacylglycerol deficient 2*	Green ontogenesis	
ppa004329m	1.04	0.033392238	*Cytochrome P450 superfamily protein*	Green ontogenesis	
ppa002714m	1.04	0.047124914	*NADP-malic enzyme 4*	Green ontogenesis	
ppa007292m	1.04	0.047124914	*cysteine synthase C1*	Green ontogenesis	
ppa011982m	1.04	0.032232406	*CTC-interacting domain 5*	Green ontogenesis	
ppa000977m	1.04	0.032002489	*H(+)-ATPase 5*	Green ontogenesis	
ppa008915m	1.04	0.021685533	*Integrase-type DNA-binding superfamily protein*	Green ontogenesis	
ppa004816m	1.04	0.011783668	*UDP-Glycosyltransferase superfamily protein*	Green ontogenesis	
ppa002515m	1.04	0.048572901	*ABA Overly-Sensitive 5*	Green ontogenesis	
ppa001138m	1.03	0.014322117	*aconitase 1*	Green ontogenesis	
ppa021469m	1.03	0.011756542	*polyol/monosaccharide transporter 5*	Green ontogenesis	
ppa003857m	1.03	0.045866541	*O-acetyltransferase family protein*	Green ontogenesis	
ppa004981m	1.03	0.01222021	*UDP-glucose 6-dehydrogenase family protein*	Green ontogenesis	
ppa009672m	1.03	0.012239259	*Protein phosphatase 2C family protein*	Green ontogenesis	
ppa015221m	1.03	0.016658462	*UDP-glucosyl transferase 78D2*	Green ontogenesis	

ppa020110m	1.03	0.031142496	PR5-like receptor kinase	Green ontogenesis	
ppa007329m	1.03	0.01414853	Peptidase S24/S26A/S26B/S26C family protein	Green ontogenesis	
ppa004526m	1.03	0.011855536	Amino acid permease family protein	Green ontogenesis	
ppa003241m	1.03	0.016083725	Protein kinase superfamily protein	Green ontogenesis	
ppa010450m	1.03	0.018277198	RPM1 interacting protein 4	Green ontogenesis	
ppb016552m	1.02	0.0489891		Green ontogenesis	
ppa005145m	1.02	0.024384276	Protein of unknown function (DUF1336)	Green ontogenesis	
ppa008380m	1.02	0.013286465	RING/FYVE/PHD zinc finger superfamily protein	Green ontogenesis	
ppa008405m	1.02	0.013173053	potassium channel beta subunit 1	Green ontogenesis	
ppa005173m	1.02	0.013111976	UDP-Glycosyltransferase superfamily protein	Green ontogenesis	
ppa002135m	1.01	0.013292855	cyclic nucleotide gated channel 1	Green ontogenesis	
ppa005852m	1.01	0.013559266	RING domain ligase2	Green ontogenesis	
ppa018817m	1.01	0.042027966	Wall-associated kinase family protein	Green ontogenesis	
ppa003188m	1.01	0.01628468	Sec1/munc18-like (SM) proteins superfamily	Green ontogenesis	
ppa008094m	1.01	0.028115807	NAD(P)-linked oxidoreductase superfamily protein	Green ontogenesis	
ppa002968m	1.01	0.016764651	S-adenosyl-L-methionine-dependent methyltransferases superfar	Green ontogenesis	
ppa009292m	1.01	0.024052575	NAD(P)-binding Rossmann-fold superfamily protein	Green ontogenesis	
ppa005972m	1.01	0.014518693	Protein of unknown function (DUF1005)	Green ontogenesis	
ppa012970m	1.01	0.024110547	Protein of unknown function (DUF581)	Green ontogenesis	
ppa011436m	1.01	0.015637073	RAB GTPASE HOMOLOG B18	Green ontogenesis	
ppa009476m	1.00	0.016020443	Protein of unknown function (DUF1295)	Green ontogenesis	
ppa010815m	1.00	0.042027966	Modifier of rudimentary (Mod(r)) protein	Green ontogenesis	
ppa009341m	1.00	0.014635191	RNA-binding KH domain-containing protein	Green ontogenesis	
ppa003855m	1.00	0.014729317	ENTH/ANTH/VHS superfamily protein	Green ontogenesis	
ppa018778m	1.00	0.017386895	permease, cytosine/purines, uracil, thiamine, allantoin family prote	Green ontogenesis	
ppa008849m	1.00	0.02291193	annexin 5	Green ontogenesis	
ppa007588m	1.00	0.034973164	reversibly glycosylated polypeptide 3	Green ontogenesis	
ppa004066m	-1.00	0.04457493	aluminum-activated, malate transporter 12	Green ontogenesis	
ppa011668m	-1.00	0.016626277		Green ontogenesis	
ppa009998m	-1.00	0.049066275	Heavy metal transport/detoxification superfamily protein	Green ontogenesis	
ppa000302m	-1.00	0.039670153	COP1-interacting protein-related	Green ontogenesis	
ppa022507m	-1.01	0.018127919	peroxin 11B	Green ontogenesis	
ppa006786m	-1.01	0.015402091	purple acid phosphatase 29	Green ontogenesis	
ppa013074m	-1.01	0.01953566	multiprotein bridging factor 1A	Green ontogenesis	
ppa011323m	-1.01	0.039614323		Green ontogenesis	
ppa013382m	-1.01	0.020112529	Chaperone DnaJ-domain superfamily protein	Green ontogenesis	
ppa003541m	-1.02	0.017713847	Transcriptional factor B3 family protein	Green ontogenesis	
ppa012083m	-1.02	0.025228749	Heavy metal transport/detoxification superfamily protein	Green ontogenesis	
ppa014253m	-1.02	0.020081916	Uncharacterised protein family SERF	Green ontogenesis	
ppa005776m	-1.02	0.013780648	tetratricopeptide repeat (TPR)-containing protein	Green ontogenesis	
ppa009696m	-1.02	0.020864252	RNA-binding CRS1 / YhbY (CRM) domain protein	Green ontogenesis	
ppa005707m	-1.02	0.034517341	GTP binding Elongation factor Tu family protein	Green ontogenesis	
ppa010399m	-1.02	0.018968545	Protein of unknown function (DUF3353)	Green ontogenesis	
ppa006586m	-1.03	0.040563089	RNA-binding (RRM/RBD/RNP motifs) family protein	Green ontogenesis	
ppa025640m	-1.03	0.012130262	2-oxoglutarate (2OG) and Fe(II)-dependent oxygenase superfami	Green ontogenesis	
ppa013839m	-1.03	0.02835143	membrane-associated progesterone binding protein 2	Green ontogenesis	
ppa017494m	-1.03	0.020864252	Ribosomal protein L9/RNase H1	Green ontogenesis	
ppa009729m	-1.03	0.027681036	copper/zinc superoxide dismutase 2	Green ontogenesis	
ppa004227m	-1.03	0.036522971	membrane protein, putative	Green ontogenesis	
ppa004683m	-1.04	0.031477188	arginosuccinate synthase family	Green ontogenesis	
ppa022110m	-1.04	0.030817416	PAK-box/P21-Rho-binding family protein	Green ontogenesis	
ppa008508m	-1.04	0.034812041	Mitochondrial transcription termination factor family protein	Green ontogenesis	
ppa006512m	-1.04	0.018147781	Glycine cleavage T-protein family	Green ontogenesis	
ppa011692m	-1.04	0.028814791		Green ontogenesis	
ppa005039m	-1.04	0.041421247	UDP-Glycosyltransferase superfamily protein	Green ontogenesis	
ppa014506m	-1.04	0.039620018	metallothionein 3	Green ontogenesis	
ppa012199m	-1.05	0.032664484	Thioredoxin superfamily protein	Green ontogenesis	
ppb017681m	-1.05	0.043850841	serine carboxypeptidase-like 18	Green ontogenesis	
ppa000806m	-1.05	0.013277979	phosphoenolpyruvate carboxylase 4	Green ontogenesis	
ppa012989m	-1.05	0.02402405	outer plastid envelope protein 16-1	Green ontogenesis	
ppa004899m	-1.05	0.039038874	Tetratricopeptide repeat (TPR)-like superfamily protein	Green ontogenesis	
ppa007787m	-1.05	0.009985452	RING/U-box superfamily protein	Green ontogenesis	
ppa008232m	-1.06	0.009630022	Amino acid kinase family protein	Green ontogenesis	
ppa001540m	-1.06	0.012701221	BTB/POZ domain-containing protein	Green ontogenesis	
ppa018503m	-1.06	0.013960403	Tetratricopeptide repeat (TPR)-like superfamily protein	Green ontogenesis	
ppa011992m	-1.06	0.018379088	HSP20-like chaperones superfamily protein	Green ontogenesis	
ppa013295m	-1.06	0.040361423	ralf-like 24	Green ontogenesis	
ppa005031m	-1.06	0.015323908	Phosphoglycerate kinase family protein	Green ontogenesis	
ppa009107m	-1.06	0.009539673	NAD(P)-binding Rossmann-fold superfamily protein	Green ontogenesis	Secondary Metabolism / Dihydroflavonols -Biotic Stress / Secondary metabolism involved into stress
ppa008899m	-1.06	0.015926673	NAD(P)-linked oxidoreductase superfamily protein	Green ontogenesis	
ppa004884m	-1.07	0.018559253	tubulin beta 8	Green ontogenesis	
ppa001339m	-1.07	0.025072173	C2 calcium/lipid-binding plant phosphoribosyltransferase family pı	Green ontogenesis	
ppa020766m	-1.07	0.0286543		Green ontogenesis	
ppa002825m	-1.07	0.012482213	phosphoglucomutase	Green ontogenesis	
ppa010370m	-1.07	0.009985452	Mog1/PsbP/DUF1795-like photosystem II reaction center PsbP fa	Green ontogenesis	Photosynthesis / Photosystem II
ppa006995m	-1.07	0.012701221	15-cis-zeta-carotene isomerase	Green ontogenesis	
ppa008425m	-1.07	0.035694193	myb-like transcription factor family protein	Green ontogenesis	
ppa013022m	-1.07	0.0359871	HSP20-like chaperones superfamily protein	Green ontogenesis	
ppa006017m	-1.07	0.047124914	mitochondrial 28S ribosomal protein S29-related	Green ontogenesis	
ppa1027225m	-1.07	0.016985954	PENTATRICOPEPTIDE REPEAT 596	Green ontogenesis	
ppa012541m	-1.08	0.037874008	Protein of unknown function, DUF584	Green ontogenesis	
ppa003314m	-1.08	0.008214002	CTP synthase family protein	Green ontogenesis	
ppa010218m	-1.08	0.009473798	Thioredoxin superfamily protein	Green ontogenesis	
ppa012825m	-1.08	0.01414853	ribosomal protein S17	Green ontogenesis	
ppa001963m	-1.08	0.009480311	with no lysine (K) kinase 1	Green ontogenesis	
ppa005521m	-1.08	0.008039428	Pyridoxal phosphate (PLP)-dependent transferases superfamily p	Green ontogenesis	Secondary Metabolism / Phenylpropanoids - Biotic Stress / Secondary metabolism involved into stress
ppa013384m	-1.08	0.039062021	Cytochrome c	Green ontogenesis	
ppa003196m	-1.09	0.008214002	Major facilitator superfamily protein	Green ontogenesis	
ppa006746m	-1.09	0.020045456	fructose-bisphosphate aldolase 2	Green ontogenesis	
ppa008420m	-1.09	0.015604991	photosystem II subunit O-2	Green ontogenesis	Photosynthesis / Photosystem II
ppa001332m	-1.09	0.009244061	ARM repeat superfamily protein	Green ontogenesis	
ppa003821m	-1.09	0.020618608	CYTOCHROME P450 51G1	Green ontogenesis	
ppa005375m	-1.09	0.015142087	O-Glycosyl hydrolases family 17 protein	Green ontogenesis	Biotic Stress / Betaglucanase
ppa010672m	-1.10	0.007534579	Protein of unknown function, DUF599	Green ontogenesis	
ppa003145m	-1.10	0.019215987	S-adenosyl-L-methionine-dependent methyltransferases superfar	Green ontogenesis	
ppa005537m	-1.10	0.007947079	plant U-box 9	Green ontogenesis	
ppa001266m	-1.10	0.018277198		Green ontogenesis	
ppa012710m	-1.10	0.029736546	EF hand calcium-binding protein family	Green ontogenesis	
ppa014610m	-1.11	0.021521204		Green ontogenesis	
ppa013536m	-1.11	0.026242601	plastid-specific ribosomal protein 4	Green ontogenesis	
ppa008173m	-1.11	0.0489891	putative endonuclease or glycosyl hydrolase with C2H2-type zinc	Green ontogenesis	
ppa011477m	-1.11	0.023944911	Ribosomal protein L34	Green ontogenesis	
ppa009356m	-1.11	0.01200695	histone deacetylase 2C	Green ontogenesis	
ppa006109m	-1.11	0.013713882	nine-cis-epoxycarotenoid dioxygenase 4	Green ontogenesis	
ppa022366m	-1.12	0.010110676	photosystem I subunit D-2	Green ontogenesis	Photosynthesis / Photosystem I
ppa008202m	-1.12	0.015061871	S-adenosyl-L-methionine-dependent methyltransferases superfar	Green ontogenesis	Biotic Stress / Secondary metabolism involved into stress
ppa007978m	-1.12	0.007248116	DNAJ heat shock family protein	Green ontogenesis	
ppa001936m	-1.12	0.030229382	cellulose synthase like E1	Green ontogenesis	
ppa005226m	-1.12	0.022624309	cytochrome P450, family 707, subfamily A, polypeptide 4	Green ontogenesis	
ppa008168m	-1.13	0.007335231	S-adenosyl-L-methionine-dependent methyltransferases superfar	Green ontogenesis	Biotic Stress / Secondary metabolism involved into stress
ppa010359m	-1.13	0.027832382	chaperonin 20	Green ontogenesis	
ppa009275m	-1.13	0.013296538	winged-helix DNA-binding transcription factor family protein	Green ontogenesis	
ppa000566m	-1.13	0.032192619	Leucine-rich receptor-like protein kinase family protein	Green ontogenesis	
ppa008475m	-1.13	0.00556862	Protein kinase superfamily protein	Green ontogenesis	
ppa024803m	-1.13	0.012924305	Heavy metal transport/detoxification superfamily protein	Green ontogenesis	
ppa008531m	-1.13	0.005565382	Late embryogenesis abundant protein, group 2	Green ontogenesis	
ppa003779m	-1.14	0.024339057		Green ontogenesis	
ppa008220m	-1.14	0.012482213	NmrA-like negative transcriptional regulator family protein	Green ontogenesis	Secondary Metabolism / Flavonols-Isoflavonoid -Biotic Stress / Secondary metabolism involved into stress
ppa012378m	-1.14	0.014308785	glutathione peroxidase 4	Green ontogenesis	
ppa009971m	-1.14	0.015061871		Green ontogenesis	
ppa013351m	-1.14	0.031869628		Green ontogenesis	
ppa011392m	-1.14	0.037161611	subunit NDH-M of NAD(P)H:plastoquinone dehydrogenase compl	Green ontogenesis	
ppa010078m	-1.14	0.009355779	photosystem II light harvesting complex gene 2.2	Green ontogenesis	Photosynthesis / Photosystem II
ppa004857m	-1.14	0.030234301	Pyridoxal-dependent decarboxylase family protein	Green ontogenesis	
ppa006279m	-1.14	0.014152338	Core-2/I-branching beta-1,6-N-acetylglucosaminyltransferase fam	Green ontogenesis	
ppa013940m	-1.14	0.040826859		Green ontogenesis	
ppa014599m	-1.15	0.034393557	GBF\'s pro-rich region-interacting factor 1	Green ontogenesis	
ppa002906m	-1.15	0.017904654	Prefoldin chaperone subunit family protein	Green ontogenesis	
ppa000651m	-1.15	0.021219925	P-loop containing nucleoside triphosphate hydrolases superfamily	Green ontogenesis	
ppa026297m	-1.15	0.013606048		Green ontogenesis	
ppa004257m	-1.15	0.013661909	Seryl-tRNA synthetase	Green ontogenesis	
ppa012917m	-1.15	0.016457416		Green ontogenesis	
ppa004692m	-1.15	0.011846605	LIM domain-containing protein	Green ontogenesis	
ppa009109m	-1.16	0.018408478	2-phosphoglycolate phosphatase 1	Green ontogenesis	
ppa018071m	-1.16	0.010285876		Green ontogenesis	
ppa016725m	-1.16	0.007200057	binding	Green ontogenesis	
ppa005564m	-1.16	0.043488749	CVP2 like 1	Green ontogenesis	
ppa003982m	-1.16	0.03012191	Eukaryotic aspartyl protease family protein	Green ontogenesis	
ppa005081m	-1.16	0.004324641	monodehydroascorbate reductase 4	Green ontogenesis	
ppa009103m	-1.17	0.041088367	Duplicated homeodomain-like superfamily protein	Green ontogenesis	
ppa012961m	-1.17	0.042044411	maternal effect embryo arrest 9	Green ontogenesis	
ppa007703m	-1.17	0.004071533	gametophytic factor 2	Green ontogenesis	
ppa014460m	-1.17	0.037866481		Green ontogenesis	
ppa012095m	-1.17	0.045371025	Phosphoglycerate mutase family protein	Green ontogenesis	
ppa007769m	-1.17	0.030181777	peroxisomal NAD-malate dehydrogenase 1	Green ontogenesis	
ppa006135m	-1.17	0.029919735	Phototropic-responsive NPH3 family protein	Green ontogenesis	
ppa006752m	-1.17	0.008251917	abscisic acid responsive elements-binding factor 3	Green ontogenesis	
ppa006131m	-1.18	0.013525815	RING/U-box superfamily protein	Green ontogenesis	

ppa013983m	-1.18	0.013956335	Calcium-binding EF-hand family protein	Green ontogenesis	
ppa024953m	-1.18	0.007231937	enzyme binding;tetrapyrrole binding	Green ontogenesis	
ppa011725m	-1.18	0.021171861	photosystem II BY	Green ontogenesis	Photosynthesis / Photosystem II
ppa006993m	-1.18	0.015447471	hydroxypyruvate reductase	Green ontogenesis	
ppa005957m	-1.18	0.027832382	Transmembrane amino acid transporter family protein	Green ontogenesis	
ppa009639m	-1.18	0.006694697	translationally controlled tumor protein	Green ontogenesis	
ppa005096m	-1.18	0.018331309	Protein of unknown function (DUF604)	Green ontogenesis	
ppa001029m	-1.18	0.043945419	Leucine-rich repeat protein kinase family protein	Green ontogenesis	
ppa010801m	-1.19	0.021308868	Rubber elongation factor protein (REF)	Green ontogenesis	
ppa002897m	-1.19	0.023720539	NSP-interacting kinase 1	Green ontogenesis	
ppa002150m	-1.19	0.018028386	STELAR K+ outward rectifier	Green ontogenesis	
ppa004353m	-1.19	0.018319902	Glycinamide ribonucleotide (GAR) synthetase	Green ontogenesis	
ppa008378m	-1.19	0.025702236	S-adenosyl-L-methionine-dependent methyltransferases superfar	Green ontogenesis	
ppa007434m	-1.20	0.044227865	alpha/beta-Hydrolases superfamily protein	Green ontogenesis	
ppa010543m	-1.20	0.03974002	fibrillarin 2	Green ontogenesis	
ppa002486m	-1.20	0.035887475	FAR1-related sequence 6	Green ontogenesis	
ppa006451m	-1.20	0.014838433	Polyketide cyclase/dehydrase and lipid transport superfamily prot	Green ontogenesis	
ppa000818m	-1.20	0.011608705	Telomerase activating protein Est1	Green ontogenesis	
ppa001909m	-1.20	0.018606606	cellulose synthase-like B4	Green ontogenesis	
ppa001590m	-1.20	0.027204998	Chaperone protein htpG family protein	Green ontogenesis	
ppa004090m	-1.20	0.042192182	serine hydroxymethyltransferase 3	Green ontogenesis	
ppa009739m	-1.21	0.003853005	Ribose 5-phosphate isomerase, type A protein	Green ontogenesis	
ppa012788m	-1.21	0.003775176	Single hybrid motif superfamily protein	Green ontogenesis	
ppa012021m	-1.22	0.014085458		Green ontogenesis	
ppa002520m	-1.22	0.024643855	RNA-binding CRS1 / YhbY (CRM) domain protein	Green ontogenesis	
ppa009490m	-1.22	0.008460683	phosphoenolpyruvate (pep)/phosphate translocator 2	Green ontogenesis	
ppa004196m	-1.22	0.006391302	ADP glucose pyrophosphorylase 1	Green ontogenesis	
ppa008026m	-1.22	0.024524469	Plant protein 1589 of unknown function	Green ontogenesis	
ppa008292m	-1.22	0.005576065	catalytics;hydrolases	Green ontogenesis	
ppa009250m	-1.22	0.01153922	nitrilase-like protein 1	Green ontogenesis	
ppa002659m	-1.23	0.035621744	mechanosensitive channel of small conductance-like 10	Green ontogenesis	
ppa012053m	-1.23	0.012797213	Lactoylglutathione lyase / glyoxalase I family protein	Green ontogenesis	
ppa002448m	-1.23	0.005822079	Rhodanese/Cell cycle control phosphatase superfamily protein	Green ontogenesis	
ppa012720m	-1.24	0.043488749	Ribosomal protein L12/ ATP-dependent Clp protease adaptor pro	Green ontogenesis	
ppa024664m	-1.24	0.006570654	elongation factor P (EF-P) family protein	Green ontogenesis	
ppa011919m	-1.24	0.018408478		Green ontogenesis	
ppa011734m	-1.24	0.021129933	Ribosomal protein L2 family	Green ontogenesis	
ppa013236m	-1.24	0.002569358	Dormancy/auxin associated family protein	Green ontogenesis	
ppa012252m	-1.24	0.004983547	AIG2-like (avirulence induced gene) family protein	Green ontogenesis	
ppa011545m	-1.24	0.021171861	plasmodesmata callose-binding protein 3	Green ontogenesis	Biotic Stress / Betaglucanase
ppa006875m	-1.24	0.01661972	NAD(P)-binding Rossmann-fold superfamily protein	Green ontogenesis	
ppa010177m	-1.25	0.038193886	DOMAIN OF UNKNOWN FUNCTION 724 7	Green ontogenesis	
ppa007671m	-1.25	0.037264947	D-aminoacid aminotransferase-like PLP-dependent enzymes sup	Green ontogenesis	
ppa010347m	-1.25	0.004857528	alfin-like 5	Green ontogenesis	
ppa009055m	-1.25	0.036868873	alpha/beta-Hydrolases superfamily protein	Green ontogenesis	
ppa007052m	-1.25	0.006046756	ATP phosphoribosyl transferase 2	Green ontogenesis	
ppa006717m	-1.25	0.013731991	calcium sensing receptor	Green ontogenesis	
ppa013281m	-1.25	0.045189211	DnaJ/Hsp40 cysteine-rich domain superfamily protein	Green ontogenesis	
ppa006497m	-1.25	0.008824115	shaggy-like kinase 13	Green ontogenesis	
ppa001111m	-1.25	0.039062021	CRS1 / YhbY (CRM) domain-containing protein	Green ontogenesis	
ppa007814m	-1.26	0.01238618	basic leucine zipper 9	Green ontogenesis	
ppa019443m	-1.26	0.048167584	cytochrome P450, family 706, subfamily A, polypeptide 4	Green ontogenesis	
ppa013023m	-1.26	0.043295416	Glyceraldehyde-3-phosphate dehydrogenase-like family protein	Green ontogenesis	
ppa007523m	-1.26	0.022117573	Sodium Bile acid symporter family	Green ontogenesis	
ppa000809m	-1.26	0.045938424	lon protease 1	Green ontogenesis	
ppa001074m	-1.26	0.016651319	starch synthase 3	Green ontogenesis	
ppa003168m	-1.26	0.006230365	Leucine-rich repeat protein kinase family protein	Green ontogenesis	
ppa002092m	-1.26	0.008732267	STRUBBELIG-receptor family 6	Green ontogenesis	
ppa013293m	-1.27	0.024204842	Yippee family putative zinc-binding protein	Green ontogenesis	
ppa021685m	-1.27	0.02453315	Homeodomain-like superfamily protein	Green ontogenesis	
ppa009087m	-1.27	0.006712093	glutathione transferase lambda 2	Green ontogenesis	Biotic Stress / Glutathione-S-Transferase
ppa005282m	-1.27	0.008702024	APS reductase 3	Green ontogenesis	
ppa010265m	-1.27	0.006386344	wall associated kinase 4	Green ontogenesis	
ppa006330m	-1.28	0.003199469	DNAJ homologue 2	Green ontogenesis	
ppa008584m	-1.28	0.024096514	DNAJ heat shock family protein	Green ontogenesis	
ppa004052m	-1.28	0.017346489	Ribonuclease III family protein	Green ontogenesis	
ppa016099m	-1.28	0.023283697	calmodulin-like 41	Green ontogenesis	
ppa009703m	-1.28	0.00257221	ferritin 2	Green ontogenesis	
ppa009335m	-1.28	0.0019245	pyrophosphorylase 6	Green ontogenesis	
ppa004657m	-1.29	0.032222955	cytochrome BC1 synthesis	Green ontogenesis	
ppa010344m	-1.29	0.021429425	carbonic anhydrase 2	Green ontogenesis	
ppa002816m	-1.29	0.024250054	Autophagy-related protein 13	Green ontogenesis	
ppa003792m	-1.29	0.02613286		Green ontogenesis	
ppa009686m	-1.29	0.029489501	light harvesting complex photosystem II	Green ontogenesis	Photosynthesis / Photosystem II
ppa012123m	-1.29	0.016442714	Ribulose bisphosphate carboxylase (small chain) family protein	Green ontogenesis	
ppa016103m	-1.30	0.001378504	SC35-like splicing factor 33	Green ontogenesis	
ppa013492m	-1.30	0.022687106	Lactoylglutathione lyase / glyoxalase I family protein	Green ontogenesis	
ppa004412m	-1.30	0.010857128	DA1-related protein 2	Green ontogenesis	
ppa003193m	-1.30	0.027947735	Major facilitator superfamily protein	Green ontogenesis	
ppa007209m	-1.30	0.002596451	Peroxisomal membrane 22 kDa (Mpv17/PMP22) family protein	Green ontogenesis	
ppa010872m	-1.31	0.02442925	nucleoside diphosphate kinase 2	Green ontogenesis	
ppa012039m	-1.31	0.01072308	low psii accumulation2	Green ontogenesis	Photosynthesis / Photosystem II
ppa013619m	-1.31	0.021532249		Green ontogenesis	
ppa001312m	-1.31	0.042639464	starch branching enzyme 2.2	Green ontogenesis	
ppa020460m	-1.31	0.035364277	2-oxoglutarate (2OG) and Fe(II)-dependent oxygenase superfami	Green ontogenesis	
ppa018798m	-1.31	0.021429425	Protein kinase family protein	Green ontogenesis	
ppa005680m	-1.31	0.018606606	cytochrome P450, family 722, subfamily A, polypeptide 1	Green ontogenesis	
ppa019849m	-1.32	0.006937435	cytochrome P450, family 706, subfamily A, polypeptide 4	Green ontogenesis	
ppa007919m	-1.32	0.005040321	basic helix-loop-helix (bHLH) DNA-binding superfamily protein	Green ontogenesis	
ppa012483m	-1.32	0.008214002	Ribosomal protein S13/S18 family	Green ontogenesis	
ppa018938m	-1.32	0.020961458	5\'-AMP-activated protein kinase-related	Green ontogenesis	
ppa006250m	-1.32	0.045641704	Glutaredoxin family protein	Green ontogenesis	
ppa004262m	-1.33	0.008908473	Amidase family protein	Green ontogenesis	
ppa011229m	-1.33	0.002459635	photosystem I subunit l	Green ontogenesis	Photosynthesis / Photosystem I
ppa003376m	-1.33	0.001356283		Green ontogenesis	
ppa013219m	-1.33	0.041580343	CLAVATA3/ESR-RELATED 44	Green ontogenesis	
ppa008661m	-1.33	0.008520341	Oxidoreductase, zinc-binding dehydrogenase family protein	Green ontogenesis	
ppa001779m	-1.33	0.020256404	mechanosensitive channel of small conductance-like 4	Green ontogenesis	
ppa025338m	-1.33	0.029827423	Exostosin family protein	Green ontogenesis	
ppa004068m	-1.34	0.02569304	Class-II DAHP synthetase family protein	Green ontogenesis	
ppa010511m	-1.34	0.010285876	photosystem I light harvesting complex gene 1	Green ontogenesis	Photosynthesis / Photosystem II
ppa1027211m	-1.34	0.001954667	chloroplastic drought-induced stress protein of 32 kD	Green ontogenesis	
ppa015574m	-1.34	0.038900295	HXXXD-type acyl-transferase family protein	Green ontogenesis	
ppa012892m	-1.34	0.025340993	UDP-Glycosyltransferase superfamily protein	Green ontogenesis	
ppa006533m	-1.35	0.008400976	ATP-dependent caseinolytic (Clp) protease/crotonase family prote	Green ontogenesis	
ppa025117m	-1.35	0.027365731	D-isomer specific 2-hydroxyacid dehydrogenase family protein	Green ontogenesis	
ppa006433m	-1.36	0.025072173	Radical SAM superfamily protein	Green ontogenesis	
ppa008633m	-1.36	0.001903748	GroES-like zinc-binding alcohol dehydrogenase family protein	Green ontogenesis	
ppa003741m	-1.36	0.021986488	hydroxyproline-rich glycoprotein family protein	Green ontogenesis	
ppa025673m	-1.36	0.01566613		Green ontogenesis	
ppa012395m	-1.36	0.001596228	photosystem I P subunit	Green ontogenesis	
ppa010498m	-1.36	0.005717601	Plastid-lipid associated protein PAP / fibrillin family protein	Green ontogenesis	
ppa007350m	-1.36	0.015650814	CYCLIN D3;2	Green ontogenesis	
ppa005780m	-1.36	0.002126783	Uridine diphosphate glycosyltransferase 74E2	Green ontogenesis	
ppa005061m	-1.36	0.001356283	DC1 domain-containing protein	Green ontogenesis	
ppa018863m	-1.36	0.006628878	thylakoid rhodanese-like	Green ontogenesis	
ppa003274m	-1.37	0.005518555	Ankyrin repeat family protein	Green ontogenesis	
ppa024185m	-1.37	0.00273149		Green ontogenesis	
ppa019589m	-1.37	0.035951348		Green ontogenesis	
ppa004666m	-1.37	0.006796102	Major facilitator superfamily protein	Green ontogenesis	
ppa010920m	-1.37	0.016853149		Green ontogenesis	
ppa003329m	-1.37	0.015192966	Protein kinase family protein	Green ontogenesis	
ppa012542m	-1.38	0.009104666	plastid transcriptionally active7	Green ontogenesis	
ppa005677m	-1.38	0.010007236	Fatty acid/sphingolipid desaturase	Green ontogenesis	
ppa009043m	-1.38	0.043617335		Green ontogenesis	
ppa007744m	-1.38	0.01604268	Aldolase superfamily protein	Green ontogenesis	
ppa008147m	-1.38	0.015464711	purple acid phosphatase 3	Green ontogenesis	
ppa002873m	-1.38	0.029271861	NSP-interacting kinase 2	Green ontogenesis	
ppa010227m	-1.38	0.004536747	translocase of the outer mitochondrial membrane 40	Green ontogenesis	
ppa014754m	-1.39	0.017436772	Heavy metal transport/detoxification superfamily protein	Green ontogenesis	
ppa018625m	-1.39	0.00063489	Dynamin related protein 4C	Green ontogenesis	
ppa019796m	-1.39	0.019522734		Green ontogenesis	
ppa007940m	-1.39	0.014913986	heptahelical transmembrane protein2	Green ontogenesis	
ppa013681m	-1.39	0.005706167	Dynein light chain type 1 family protein	Green ontogenesis	
ppa004921m	-1.39	0.001129143	O-fucosyltransferase family protein	Green ontogenesis	
ppa012362m	-1.39	0.006131947	photosystem I reaction center subunit PSI-N, chloroplast, putative	Green ontogenesis	Photosynthesis / Photosystem I
ppa024411m	-1.40	0.033861123	Eukaryotic aspartyl protease family protein	Green ontogenesis	
ppa003081m	-1.40	0.010850233	receptor like protein 4	Green ontogenesis	
ppa005169m	-1.40	0.035710185	Arabidopsis thaliana protein of unknown function (DUF821)	Green ontogenesis	
ppa010848m	-1.40	0.006286591	FKBP-like peptidyl-prolyl cis-trans isomerase family protein	Green ontogenesis	
ppa003813m	-1.40	0.038059254	glucuronidase 2	Green ontogenesis	
ppa014101m	-1.40	0.035968471		Green ontogenesis	
ppa003336m	-1.40	0.001377353	XS domain-containing protein / XS zinc finger domain-containing	Green ontogenesis	
ppa012110m	-1.40	0.008743567	Protein of unknown function (DUF3049)	Green ontogenesis	
ppa010398m	-1.41	0.030923972	Mog1/PsbP/DUF1795-like photosystem II reaction center PsbP fa	Green ontogenesis	
ppa008622m	-1.41	0.009969838	GroES-like zinc-binding alcohol dehydrogenase family protein	Green ontogenesis	
ppa013016m	-1.41	0.003248661	multiprotein bridging factor 1C	Green ontogenesis	
ppa010364m	-1.42	0.010485766	tonoplast intrinsic protein 1;3	Green ontogenesis	

ppa003283m	-1.42	0.00837628	*like SEX4 1*	Green ontogenesis	
ppa004073m	-1.42	0.040609336	*plastid transcriptionally active 12*	Green ontogenesis	
ppa007108m	-1.42	0.032222955	*histone deacetylase 8*	Green ontogenesis	
ppa008969m	-1.42	0.038193886	*bifunctional nuclease i*	Green ontogenesis	
ppa011922m	-1.43	0.034812041	*Expressed protein*	Green ontogenesis	
ppa007738m	-1.43	0.008637784	*leucoanthocyanidin dioxygenase*	Green ontogenesis	
ppa001064m	-1.43	0.042621075	*PLAT/LH2 domain-containing lipoxygenase family protein*	Green ontogenesis	
ppa003656m	-1.43	0.015697941	*FAD-linked oxidases family protein*	Green ontogenesis	
ppa023958m	-1.43	0.042137585		Green ontogenesis	
ppa011575m	-1.43	0.008177669	*chlororespiratory reduction 3*	Green ontogenesis	
ppa005518m	-1.43	0.012934662	*P-loop containing nucleoside triphosphate hydrolases superfamily*	Green ontogenesis	
ppa006025m	-1.43	0.015223836	*glutamine synthetase 2*	Green ontogenesis	
ppa013021m	-1.43	0.004416065	*photosystem I subunit H-1*	Green ontogenesis	Photosynthesis / Photosystem I
ppa022650m	-1.44	0.005749722	*phosphoglycerate/bisphosphoglycerate mutase family protein*	Green ontogenesis	
ppa011210m	-1.44	0.036128421	*Polyketide cyclase/dehydrase and lipid transport superfamily prot*	Green ontogenesis	
ppa013379m	-1.44	0.002266721	*2-phosphoglycolate phosphatase 1*	Green ontogenesis	
ppa010611m	-1.44	0.036592258	*ARF-GAP domain 15*	Green ontogenesis	
ppa010357m	-1.44	0.014842022	*rotamase CYP 4*	Green ontogenesis	
ppa013310m	-1.44	0.012134352	*Ribosomal protein S26e family protein*	Green ontogenesis	
ppa009545m	-1.44	0.043416038	*auxin-induced protein 13*	Green ontogenesis	
ppa002776m	-1.45	0.001832929	*XH/XS domain-containing protein*	Green ontogenesis	
ppa006379m	-1.45	0.043345793		Green ontogenesis	
ppa006575m	-1.45	0.010502537	*Protein of unknown function (DUF506)*	Green ontogenesis	
ppa000811m	-1.46	0.030985265	*poly(ADP-ribose) polymerase 2*	Green ontogenesis	
ppa010953m	-1.46	0.008679452	*photosystem I subunit F*	Green ontogenesis	Photosynthesis / Photosystem I
ppa007723m	-1.46	0.030817416	*dicarboxylate diiron protein, putative (Crd1)*	Green ontogenesis	
ppa018848m	-1.46	0.005228527	*monooxygenase 1*	Green ontogenesis	
ppa009910m	-1.47	0.041622975	*Dihydrodipicolinate reductase, bacterial/plant*	Green ontogenesis	
ppa016245m	-1.47	0.019859021	*Heavy metal transport/detoxification superfamily protein*	Green ontogenesis	
ppa010066m	-1.47	0.001636449	*chlorophyll A/B binding protein 1*	Green ontogenesis	Photosynthesis / Photosystem II
ppa013494m	-1.47	0.002644103		Green ontogenesis	
ppa010038m	-1.47	0.004622112	*dehydroascorbate reductase 1*	Green ontogenesis	
ppa012650m	-1.47	0.003258821	*MLP-like protein 423*	Green ontogenesis	
ppa009960m	-1.48	0.005525221	*2-cysteine peroxiredoxin B*	Green ontogenesis	
ppa008693m	-1.48	0.010757856	*Tetratricopeptide repeat (TPR)-like superfamily protein*	Green ontogenesis	
ppa006606m	-1.48	0.007335231	*chloroplast stem-loop binding protein of 41 kDa*	Green ontogenesis	
ppa008255m	-1.48	0.020834362	*glyoxylate reductase 2*	Green ontogenesis	
ppa004548m	-1.48	0.013126669	*C2H2-like zinc finger protein*	Green ontogenesis	
ppa021377m	-1.48	0.004695941	*Sugar isomerase (SIS) family protein*	Green ontogenesis	
ppa003051m	-1.48	0.03921317	*Tetratricopeptide repeat (TPR)-like superfamily protein*	Green ontogenesis	
ppa007597m	-1.49	0.03521989	*senescence-related gene 1*	Green ontogenesis	
ppa000739m	-1.49	0.035621744	*Leucine-rich receptor-like protein kinase family protein*	Green ontogenesis	
ppa011918m	-1.49	0.004946255	*adenine phosphoribosyltransferase 5*	Green ontogenesis	
ppa008264m	-1.49	0.038059254	*galactinol synthase 4*	Green ontogenesis	
ppa006879m	-1.50	0.008214002	*Homeodomain-like superfamily protein*	Green ontogenesis	
ppa003996m	-1.50	0.014058244	*Major facilitator superfamily protein*	Green ontogenesis	
ppa022557m	-1.50	0.049066275	*receptor lectin kinase*	Green ontogenesis	
ppa006978m	-1.50	0.008317277	*UBX domain-containing protein*	Green ontogenesis	
ppa003443m	-1.50	0.040401463		Green ontogenesis	
ppa003723m	-1.50	0.044478985	*acyl-activating enzyme 14*	Green ontogenesis	Secondary Metabolism / Phenylpropanoids - Biotic Stress / Secondary metabolism involved into stress
ppa001136m	-1.51	0.046025379	*P-loop containing nucleoside triphosphate hydrolases superfamily*	Green ontogenesis	
ppa011986m	-1.51	0.00431269	*RmlC-like cupins superfamily protein*	Green ontogenesis	
ppa011175m	-1.51	0.004245952	*Protein-tyrosine phosphatase-like, PTPLA*	Green ontogenesis	
ppa015470m	-1.51	0.005446585	*pfkB-like carbohydrate kinase family protein*	Green ontogenesis	
ppa006616m	-1.51	0.00142295	*non-specific phospholipase C6*	Green ontogenesis	
ppa010691m	-1.51	0.00536134	*DNAJ heat shock family protein*	Green ontogenesis	
ppa012372m	-1.51	0.002903957	*thioredoxin F-type 1*	Green ontogenesis	
ppa024043m	-1.51	0.048268166	*Embryo-specific protein 3, (ATS3)*	Green ontogenesis	
ppa022227m	-1.52	0.001136153	*non-intrinsic ABC protein 3*	Green ontogenesis	
ppa015619m	-1.52	0.021181742	*beta glucosidase 40*	Green ontogenesis	
ppa011635m	-1.52	0.002011992		Green ontogenesis	
ppa002098m	-1.52	0.002765669	*delta 1-pyrroline-5-carboxylate synthase 2*	Green ontogenesis	
ppa001793m	-1.52	0.0286543	*Protein of unknown function (DUF288)*	Green ontogenesis	
ppa005774m	-1.52	0.025548979	*HXXXD-type acyl-transferase family protein*	Green ontogenesis	Secondary Metabolism / Phenylpropanoids - Biotic Stress / Secondary metabolism involved into stress
ppa019477m	-1.52	0.001309569		Green ontogenesis	
ppa003697m	-1.52	0.021515799	*methylesterase PCR A*	Green ontogenesis	
ppa018535m	-1.53	0.028801371	*AUX/IAA transcriptional regulator family protein*	Green ontogenesis	
ppa015564m	-1.53	0.0389037	*Leucine-rich repeat transmembrane protein kinase*	Green ontogenesis	
ppa005057m	-1.53	0.00321474	*Transmembrane amino acid transporter family protein*	Green ontogenesis	
ppa003464m	-1.53	0.006369787	*Plasma-membrane choline transporter family protein*	Green ontogenesis	
ppa017093m	-1.53	0.018331309	*AMP-dependent synthetase and ligase family protein*	Green ontogenesis	Secondary Metabolism / Phenylpropanoids - Biotic Stress / Secondary metabolism involved into stress
ppa022256m	-1.53	0.044449164	*Eukaryotic aspartyl protease family protein*	Green ontogenesis	
ppa016365m	-1.54	0.028711854	*UDP-Glycosyltransferase superfamily protein*	Green ontogenesis	Secondary Metabolism / Dihydroflavonols -Biotic Stress / Secondary metabolism involved into stress
ppa010195m	-1.54	0.002872534	*light harvesting complex photosystem II subunit 6*	Green ontogenesis	Photosynthesis / Photosystem II
ppa009363m	-1.54	0.047045295	*Mitochondrial inner membrane translocase complex, subunit Tim4*	Green ontogenesis	
ppa012668m	-1.54	0.010110676		Green ontogenesis	
ppa002599m	-1.55	0.022726958	*pumilio 24*	Green ontogenesis	
ppa026117m	-1.55	0.013173053	*Amino acid dehydrogenase family protein*	Green ontogenesis	
ppa012823m	-1.55	0.048150406	*photosystem II 11 kDa protein-related*	Green ontogenesis	Photosynthesis / Photosystem II
ppa026994m	-1.55	0.009359344		Green ontogenesis	
ppa025847m	-1.55	0.006018153		Green ontogenesis	
ppa006663m	-1.55	0.016484332	*FAD-dependent oxidoreductase family protein*	Green ontogenesis	
ppa003275m	-1.55	0.030879233	*SKU5 similar 3*	Green ontogenesis	
ppa005432m	-1.55	0.000724297	*4-hydroxy-3-methylbut-2-enyl diphosphate reductase*	Green ontogenesis	Biotic Stress / Secondary metabolism involved into stress
ppa005691m	-1.55	0.038059254		Green ontogenesis	
ppa003798m	-1.55	0.015637073	*FAD-binding Berberine family protein*	Green ontogenesis	
ppa000127m	-1.56	0.040675472	*Outer arm dynein light chain 1 protein*	Green ontogenesis	
ppa003440m	-1.56	0.013786945	*heat shock protein 60-3A*	Green ontogenesis	
ppa006973m	-1.56	0.000305193	*beta-1,4-N-acetylglucosaminyltransferase family protein*	Green ontogenesis	
ppa008288m	-1.56	0.004689769	*arginase*	Green ontogenesis	
ppa010093m	-1.56	0.00519458	*photosystem II subunit P-1*	Green ontogenesis	Photosynthesis / Photosystem II
ppa003803m	-1.57	0.013583273	*FAD-linked oxidases family protein*	Green ontogenesis	
ppa009589m	-1.57	0.001835024	*alpha/beta-Hydrolases superfamily protein*	Green ontogenesis	
ppa003999m	-1.57	0.000132814	*Xanthine/uracil permease family protein*	Green ontogenesis	
ppa001469m	-1.58	0.011576389	*subtilisin-like serine protease 3*	Green ontogenesis	
ppa017055m	-1.58	0.006119653	*UDP-glucosyl transferase 85A2*	Green ontogenesis	
ppa023083m	-1.58	0.012954309	*S-adenosyl-L-methionine-dependent methyltransferases superfar*	Green ontogenesis	
ppa014212m	-1.58	0.003904995	*metallothionein 2B*	Green ontogenesis	
ppa013173m	-1.59	0.042244518	*Histone superfamily protein*	Green ontogenesis	
ppb017585m	-1.59	0.046887875	*AGAMOUS-like 24*	Green ontogenesis	
ppa022268m	-1.59	0.015711038	*PsbP-like protein 2*	Green ontogenesis	Photosynthesis / Photosystem II
ppa000269m	-1.59	0.021530748	*ATP binding cassette subfamily B1*	Green ontogenesis	
ppa013566m	-1.59	0.046602587		Green ontogenesis	
ppa007518m	-1.59	0.004689769	*high mobility group B1*	Green ontogenesis	
ppa002337m	-1.59	0.009969838	*DNA photolyases;DNA photolyases*	Green ontogenesis	
ppa002621m	-1.59	0.007317315	*CTP synthase family protein*	Green ontogenesis	
ppb008343m	-1.60	0.008587072	*Far-red impaired responsive (FAR1) family protein*	Green ontogenesis	
ppa009046m	-1.60	0.040636969	*Uncharacterised protein family UPF0090*	Green ontogenesis	
ppa006977m	-1.60	0.001444851	*GroES-like zinc-binding alcohol dehydrogenase family protein*	Green ontogenesis	
ppa008250m	-1.60	0.001390667	*glyceraldehyde-3-phosphate dehydrogenase C subunit 1*	Green ontogenesis	
ppa001056m	-1.60	0.035777778	*Leucine-rich repeat protein kinase family protein*	Green ontogenesis	
ppa021761m	-1.60	0.022868501	*P-loop containing nucleoside triphosphate hydrolases superfamily*	Green ontogenesis	
ppa005545m	-1.60	0.017108753	*serine carboxypeptidase-like 20*	Green ontogenesis	
ppa011251m	-1.60	0.017108753		Green ontogenesis	
ppa001750m	-1.60	0.00762732	*S-adenosyl-L-methionine-dependent methyltransferases superfar*	Green ontogenesis	
ppa014651m	-1.60	0.029578621	*Dynamin related protein 4C*	Green ontogenesis	
ppa007221m	-1.61	0.023627168	*Haloacid dehalogenase-like hydrolase (HAD) superfamily protein*	Green ontogenesis	
ppa005450m	-1.61	0.010263505	*beta-amylase 4*	Green ontogenesis	
ppa016424m	-1.62	0.030817416	*Agenet domain-containing protein*	Green ontogenesis	
ppa002111m	-1.62	0.000167497	*4-hydroxy-3-methylbut-2-enyl diphosphate synthase*	Green ontogenesis	Biotic Stress / Secondary metabolism involved into stress
ppa010942m	-1.62	0.001753907		Green ontogenesis	
ppa006341m	-1.62	0.003181723	*O-fucosyltransferase family protein*	Green ontogenesis	
ppa005939m	-1.63	0.025923511	*ankyrin repeat family protein*	Green ontogenesis	
ppa012088m	-1.63	0.005407169	*Rhodanese/Cell cycle control phosphatase superfamily protein*	Green ontogenesis	
ppa007237m	-1.63	0.002510676	*Nucleotide/sugar transporter family protein*	Green ontogenesis	
ppa013564m	-1.63	0.038754047	*Protein Transporter, Pam16*	Green ontogenesis	
ppa025967m	-1.63	0.038472915		Green ontogenesis	
ppa007235m	-1.63	0.002196359	*jasmonate-zim-domain protein 3*	Green ontogenesis	
ppa008698m	-1.63	0.001708755	*Peroxidase superfamily protein*	Green ontogenesis	
ppa026728m	-1.63	0.02291193	*Rhodanese/Cell cycle control phosphatase superfamily protein*	Green ontogenesis	
ppa006728m	-1.63	0.001925505	*BCL-2-associated athanogene 7*	Green ontogenesis	
ppa001102m	-1.64	0.015436875	*heat shock protein 101*	Green ontogenesis	
ppa011639m	-1.64	0.01414853	*Glycoprotein membrane precursor GPI-anchored*	Green ontogenesis	
ppa006194m	-1.64	0.034812041	*cytochrome P450, family 77, subfamily A, polypeptide 5 pseudoge*	Green ontogenesis	
ppa007480m	-1.64	0.041305717	*Eukaryotic aspartyl protease family protein*	Green ontogenesis	
ppa002901m	-1.64	0.007313197	*DHHC-type zinc finger family protein*	Green ontogenesis	
ppa013911m	-1.64	0.002151548		Green ontogenesis	
ppa003234m	-1.65	0.004115195	*DC1 domain-containing protein*	Green ontogenesis	
ppa025000m	-1.65	0.004096934	*2-oxoglutarate (2OG) and Fe(II)-dependent oxygenase superfami*	Green ontogenesis	
ppa019898m	-1.65	0.006275728	*Protein of unknown function (DUF3464)*	Green ontogenesis	
ppa004949m	-1.65	0.01397337	*Transmembrane amino acid transporter family protein*	Green ontogenesis	
ppa026402m	-1.65	0.015195073	*Ankyrin repeat family protein*	Green ontogenesis	
ppa025396m	-1.65	0.046266085	*glycosyl hydrolase family 81 protein*	Green ontogenesis	
ppa005829m	-1.66	0.000740036	*cryptochrome-interacting basic-helix-loop-helix 1*	Green ontogenesis	
ppa006760m	-1.66	0.046486257	*Aluminium activated malate transporter family protein*	Green ontogenesis	
ppa011053m	-1.66	0.044751561	*osmotin 34*	Green ontogenesis	
ppa010341m	-1.66	0.005400761	*Mitochondrial glycoprotein family protein*	Green ontogenesis	
ppa001503m	-1.66	0.028110751	*HEAT SHOCK PROTEIN 89.1*	Green ontogenesis	

ppa011743m	-1.67	0.004147776	*plastid division2*	Green ontogenesis	
ppa007251m	-1.67	0.0175475		Green ontogenesis	
ppa009110m	-1.67	0.008400976	*photosystem I light harvesting complex gene 3*	Green ontogenesis	Photosynthesis / Photosystem II
ppa019577m	-1.67	0.023273698	*Eukaryotic aspartyl protease family protein*	Green ontogenesis	
ppa012567m	-1.67	0.005228527	*photosystem I subunit G*	Green ontogenesis	Photosynthesis / Photosystem I
ppa012082m	-1.67	0.028991022	*Thioesterase superfamily protein*	Green ontogenesis	
ppa021176m	-1.68	0.002349391	*IQ-domain 11*	Green ontogenesis	
ppb011119m	-1.68	0.033841615	*NTF2-like*	Green ontogenesis	
ppa013789m	-1.68	0.016041081		Green ontogenesis	
ppa008069m	-1.68	0.000131152	*dihydroflavonol 4-reductase*	Green ontogenesis	Secondary Metabolism / Dihydroflavonols -Biotic Stress / Secondary metabolism involved into stress
ppa000838m	-1.68	0.029929929	*Leucine-rich receptor-like protein kinase family protein*	Green ontogenesis	
ppa000357m	-1.68	0.015402091	*ketose-bisphosphate aldolase class-II family protein*	Green ontogenesis	
ppa005063m	-1.68	0.002193119	*VALINE-TOLERANT 1*	Green ontogenesis	
ppa006566m	-1.68	0.022334902	*POX (plant homeobox) family protein*	Green ontogenesis	
ppa011202m	-1.69	0.00739681	*glutathione S-transferase TAU 19*	Green ontogenesis	Biotic Stress / Glutathione-S-Transferase
ppa001826m	-1.69	0.024528759	*Protein kinase superfamily protein*	Green ontogenesis	
ppa009517m	-1.69	0.011520875	*Uncharacterized conserved protein (DUF2358)*	Green ontogenesis	
ppa005356m	-1.69	0.019392198	*O-Glycosyl hydrolases family 17 protein*	Green ontogenesis	Biotic Stress / Betaglucanase
ppa008655m	-1.70	0.027204998	*magnesium-protoporphyrin IX methyltransferase*	Green ontogenesis	
ppa011822m	-1.70	0.007402349	*Uncharacterised BCR, YbaB family COG0718*	Green ontogenesis	
ppa011104m	-1.70	0.012504742		Green ontogenesis	
ppa012570m	-1.70	0.000929961		Green ontogenesis	
ppa002417m	-1.70	0.010485766	*rhamnose biosynthesis 1*	Green ontogenesis	
ppa012090m	-1.70	0.004482558	*Ribosomal protein L22p/L17e family protein*	Green ontogenesis	
ppa016194m	-1.71	0.026628057	*receptor lectin kinase*	Green ontogenesis	
ppa001524m	-1.71	0.001563552	*RNA-dependent RNA polymerase 1*	Green ontogenesis	
ppa015696m	-1.71	0.010641834	*wall associated kinase 3*	Green ontogenesis	
ppa013037m	-1.71	0.002434798	*RNA polymerase Rpb6*	Green ontogenesis	
ppa000968m	-1.71	0.034404479	*lipoxygenase 2*	Green ontogenesis	
ppa021045m	-1.71	0.014656084	*Mitochondrial transcription termination factor family protein*	Green ontogenesis	
ppa007775m	-1.72	0.001596228	*2-oxoglutarate (2OG) and Fe(II)-dependent oxygenase superfami*	Green ontogenesis	
ppa007097m	-1.72	0.006885816	*Mitochondrial transcription termination factor family protein*	Green ontogenesis	
ppa023231m	-1.72	0.001390383		Green ontogenesis	
ppa018530m	-1.72	0.016734077	*cytochrome P450, family 714, subfamily A, polypeptide 1*	Green ontogenesis	
ppa005824m	-1.73	0.015422357		Green ontogenesis	
ppa004027m	-1.73	0.021103819	*calcium-dependent protein kinase 29*	Green ontogenesis	
ppa004549m	-1.73	0.032274743	*Protein kinase superfamily protein*	Green ontogenesis	
ppa012923m	-1.73	0.001903748	*photosystem I subunit E-2*	Green ontogenesis	Photosynthesis / Photosystem I
ppa1027173m	-1.73	0.041262877		Green ontogenesis	
ppa000550m	-1.74	0.015711038	*Leucine-rich receptor-like protein kinase family protein*	Green ontogenesis	
ppa005455m	-1.74	0.010285876	*HXXXD-type acyl-transferase family protein*	Green ontogenesis	Secondary Metabolism / Phenylpropanoids - Biotic Stress / Secondary metabolism involved into stress
ppa001766m	-1.74	0.005965299	*Protein kinase protein with adenine nucleotide alpha hydrolases-li*	Green ontogenesis	
ppa001081m	-1.74	0.022069705	*RECQ helicase L4B*	Green ontogenesis	
ppa023926m	-1.74	0.012036731	*Homeodomain-like superfamily protein*	Green ontogenesis	
ppa024722m	-1.74	0.007814623	*Dynamin related protein 4C*	Green ontogenesis	
ppa014259m	-1.74	0.001834188	*Uncharacterised protein family SERF*	Green ontogenesis	
ppa000573m	-1.75	0.010487617	*Leucine-rich receptor-like protein kinase family protein*	Green ontogenesis	
ppa012248m	-1.75	0.033160329	*early nodulin-like protein 17*	Green ontogenesis	
ppa005405m	-1.75	0.001136281	*Pyridoxal phosphate (PLP)-dependent transferases superfamily p*	Green ontogenesis	Biotic Stress / Secondary metabolism involved into stress
ppa020579m	-1.76	0.036793334	*Glucose-methanol-choline (GMC) oxidoreductase family protein*	Green ontogenesis	
ppa008713m	-1.76	0.027561095	*NAD(P)-binding Rossmann-fold superfamily protein*	Green ontogenesis	
ppa005381m	-1.76	0.025228749		Green ontogenesis	
ppa000905m	-1.77	0.013353805	*limit dextrinase*	Green ontogenesis	
ppa015954m	-1.77	0.013518168	*myb domain protein 48*	Green ontogenesis	
ppa026228m	-1.77	0.020618608	*glycosyl hydrolase 9A1*	Green ontogenesis	
ppa008215m	-1.77	0.020683931	*Protein kinase superfamily protein*	Green ontogenesis	
ppa003634m	-1.78	0.020522982	*Protein of unknown function, DUF547*	Green ontogenesis	
ppa014503m	-1.78	0.007335849	*arabinogalactan protein 15*	Green ontogenesis	
ppa001005m	-1.78	0.002151548	*Leucine-rich repeat transmembrane protein kinase*	Green ontogenesis	
ppa004997m	-1.79	0.029445514	*Sulfite exporter TauE/SafE family protein*	Green ontogenesis	
ppa001903m	-1.79	0.005352781		Green ontogenesis	
ppa021498m	-1.79	0.009473175	*fatty alcohol oxidase 3*	Green ontogenesis	
ppa009101m	-1.79	0.004773435	*NAD(P)-binding Rossmann-fold superfamily protein*	Green ontogenesis	
ppa004889m	-1.79	0.022917206	*sphere organelles protein-related*	Green ontogenesis	
ppa000641m	-1.79	0.015061871	*cellulose synthase A4*	Green ontogenesis	
ppa002751m	-1.79	0.001280519	*HAD-superfamily hydrolase, subfamily IG, 5\'-nucleotidase*	Green ontogenesis	
ppa003264m	-1.79	0.013959141	*Protein kinase superfamily protein*	Green ontogenesis	
ppa013890m	-1.80	0.007033807	*Bifunctional inhibitor/lipid-transfer protein/seed storage 2S albumi*	Green ontogenesis	
ppa013084m	-1.80	0.005504705	*RNA-binding (RRM/RBD/RNP motifs) family protein*	Green ontogenesis	
ppa023214m	-1.80	0.016363726		Green ontogenesis	
ppa002046m	-1.80	0.003378741	*ABC-2 type transporter family protein*	Green ontogenesis	
ppa011941m	-1.80	0.00536134	*histone H1-3*	Green ontogenesis	
ppa007034m	-1.81	0.001716618	*Galactose oxidase/kelch repeat superfamily protein*	Green ontogenesis	
ppa025477m	-1.81	0.003947305	*hydroxyproline-rich glycoprotein family protein*	Green ontogenesis	
ppa007969m	-1.81	0.000509355	*2-oxoglutarate (2OG) and Fe(II)-dependent oxygenase superfami*	Green ontogenesis	
ppa007489m	-1.81	0.006667297	*Rhomboid-related intramembrane serine protease family protein*	Green ontogenesis	
ppa013006m	-1.81	0.010302772	*RmlC-like cupins superfamily protein*	Green ontogenesis	
ppa010371m	-1.82	0.0144879	*RING/U-box superfamily protein*	Green ontogenesis	
ppa022562m	-1.82	0.01898451	*AMP-dependent synthetase and ligase family protein*	Green ontogenesis	Secondary Metabolism / Phenylpropanoids - Biotic Stress / Secondary metabolism involved into stress
ppa003374m	-1.82	0.0002673	*DC1 domain-containing protein*	Green ontogenesis	
ppa004867m	-1.82	0.014229099	*adenylate kinase family protein*	Green ontogenesis	
ppa000827m	-1.83	0.007792366	*Glycosyl transferase, family 35*	Green ontogenesis	
ppa004124m	-1.83	0.025507167	*Protein kinase superfamily protein*	Green ontogenesis	
ppa003924m	-1.83	0.017445235	*aspartic proteinase A1*	Green ontogenesis	
ppa008295m	-1.83	0.000256403	*NAD(P)-binding Rossmann-fold superfamily protein*	Green ontogenesis	Secondary Metabolism / Dihydroflavonols -Biotic Stress / Secondary metabolism involved into stress
ppa007149m	-1.83	0.005734965	*alpha/beta-Hydrolases superfamily protein*	Green ontogenesis	
ppa025097m	-1.83	0.025023254	*isoamylase 1*	Green ontogenesis	
ppa001635m	-1.84	0.00036796	*nucleolar protein gar2-related*	Green ontogenesis	
ppa015939m	-1.84	0.013250697	*UDP-glucosyl transferase 78D2*	Green ontogenesis	
ppa010901m	-1.84	0.008098273	*Nucleic acid-binding, OB-fold-like protein*	Green ontogenesis	
ppa004962m	-1.84	0.001354532	*Rho GTPase activating protein with PAK-box/P21-Rho-binding do*	Green ontogenesis	
ppa022266m	-1.84	0.048268166	*ABA-responsive element binding protein 3*	Green ontogenesis	
ppa024837m	-1.84	0.007832965		Green ontogenesis	
ppa009908m	-1.85	0.024010589	*Haloacid dehalogenase-like hydrolase (HAD) superfamily protein*	Green ontogenesis	
ppa018248m	-1.85	0.046486257		Green ontogenesis	
ppa000855m	-1.85	0.008851466	*casein lytic proteinase B3*	Green ontogenesis	
ppa013406m	-1.85	0.006211218	*photosystem II subunit X*	Green ontogenesis	
ppa006608m	-1.85	0.042174995	*glucose-6-phosphate/phosphate translocator 2*	Green ontogenesis	
ppa002521m	-1.85	0.020864252	*Family of unknown function (DUF566)*	Green ontogenesis	
ppa000901m	-1.86	0.018968545	*ubiquitin-specific protease 15*	Green ontogenesis	
ppa021182m	-1.86	0.000467231	*Leucine-rich repeat transmembrane protein kinase*	Green ontogenesis	
ppa002531m	-1.86	0.000144691	*AMP-dependent synthetase and ligase family protein*	Green ontogenesis	
ppa018548m	-1.87	0.013959141	*Protein phosphatase 2C family protein*	Green ontogenesis	
ppa011390m	-1.87	0.001488317	*dehydroascorbate reductase 2*	Green ontogenesis	
ppa003391m	-1.87	0.000308322	*heat shock protein 60*	Green ontogenesis	
ppa010480m	-1.88	0.02288866	*LOB domain-containing protein 38*	Green ontogenesis	
ppa013064m	-1.88	0.005420577	*photosystem I subunit O*	Green ontogenesis	Photosynthesis / Photosystem I
ppa014222m	-1.88	0.018586871	*Heavy metal transport/detoxification superfamily protein*	Green ontogenesis	
ppa013112m	-1.88	0.001564867	*Calcium-binding EF-hand family protein*	Green ontogenesis	
ppa024785m	-1.88	0.000896741		Green ontogenesis	
ppa011991m	-1.88	0.04318744		Green ontogenesis	
ppa002387m	-1.88	0.001947563	*Exostosin family protein*	Green ontogenesis	
ppa010081m	-1.88	0.005407169	*chlorophyll A/B-binding protein 2*	Green ontogenesis	Photosynthesis / Photosystem II
ppa013330m	-1.88	0.006905958		Green ontogenesis	
ppa021923m	-1.89	0.032388576		Green ontogenesis	
ppa002230m	-1.89	0.010679156	*auxin response factor 11*	Green ontogenesis	
ppa026381m	-1.89	0.025679367	*Cytochrome b561/ferric reductase transmembrane protein family*	Green ontogenesis	
ppa005866m	-1.89	0.029185084	*TRICHOME BIREFRINGENCE-LIKE 25*	Green ontogenesis	
ppa010877m	-1.89	0.01200695	*Phospholipase A2 family protein*	Green ontogenesis	
ppa026371m	-1.90	0.006810025	*receptor-like kinase in flowers 1*	Green ontogenesis	
ppa005502m	-1.90	0.014058244	*HXXXD-type acyl-transferase family protein*	Green ontogenesis	Secondary metabolism / Anthocyanins - Biotic Stress / Secondary metabolism involved into stress
ppa009771m	-1.90	0.007066558	*RING/U-box superfamily protein*	Green ontogenesis	
ppa008556m	-1.90	0.020961458	*TARGET OF MONOPTEROS 6*	Green ontogenesis	
ppa009846m	-1.90	0.022214291	*phosphoenolpyruvate carboxylase kinase 1*	Green ontogenesis	
ppa017809m	-1.91	0.000257908	*AGAMOUS-like 24*	Green ontogenesis	
ppa008680m	-1.91	0.035372642	*Oxidoreductase, zinc-binding dehydrogenase family protein*	Green ontogenesis	
ppa014035m	-1.91	0.001285896		Green ontogenesis	
ppa023648m	-1.91	0.010274587		Green ontogenesis	
ppa017629m	-1.92	0.028478037	*PLAC8 family protein*	Green ontogenesis	
ppa010099m	-1.92	0.002306433	*chlorophyll A/B binding protein 1*	Green ontogenesis	Photosynthesis / Photosystem II
ppa003008m	-1.93	0.001136281	*glucan synthase-like 12*	Green ontogenesis	
ppa019316m	-1.93	0.023218112	*cyclic nucleotide gated channel 1*	Green ontogenesis	
ppa000430m	-1.93	0.014632403	*ATPase E1-E2 type family protein / haloacid dehalogenase-like h*	Green ontogenesis	
ppa022693m	-1.93	0.014149458	*Pyridoxal phosphate (PLP)-dependent transferases superfamily p*	Green ontogenesis	Biotic Stress / Secondary metabolism involved into stress
ppa024737m	-1.94	0.005532415	*Sulfite exporter TauE/SafE family protein*	Green ontogenesis	
ppa009901m	-1.94	0.008946766	*myo-inositol oxygenase 1*	Green ontogenesis	
ppa001382m	-1.94	0.004351805	*beta galactosidase 1*	Green ontogenesis	
ppa007147m	-1.94	0.001207475		Green ontogenesis	
ppa000491m	-1.94	0.021429425	*phytochrome E*	Green ontogenesis	
ppa005835m	-1.95	0.033573497	*cation exchanger 3*	Green ontogenesis	
ppb009745m	-1.95	0.002958088	*sphingoid base hydroxylase 1*	Green ontogenesis	
ppa012425m	-1.95	0.005484861	*photosystem II family protein*	Green ontogenesis	Photosynthesis / Photosystem II
ppa009014m	-1.96	0.008318027	*UDP-3-O-acyl N-acetylglycosamine deacetylase family protein*	Green ontogenesis	
ppa026998m	-1.96	0.013862504	*DNA binding*	Green ontogenesis	
ppa000872m	-1.96	0.03703815	*Protein kinase superfamily protein with octicosapeptide/Phox/Bem*	Green ontogenesis	
ppa022274m	-1.96	0.00074902	*K-box region and MADS-box transcription factor family protein*	Green ontogenesis	
ppa016888m	-1.97	0.000848711		Green ontogenesis	
ppa011054m	-1.97	0.015354497	*response regulator 9*	Green ontogenesis	

ppa002648m	-1.97	0.025667083	sulfate transporter 3;1	Green ontogenesis	
ppa009691m	-1.97	0.005273033	plasma membrane intrinsic protein 3	Green ontogenesis	
ppa017507m	-1.98	0.039064519		Green ontogenesis	
ppa011347m	-1.98	0.011163596		Green ontogenesis	
ppa003891m	-1.99	0.019638892	beta glucosidase 13	Green ontogenesis	
ppa020175m	-1.99	0.007903212		Green ontogenesis	
ppb022980m	-1.99	0.01771093	root hair specific 16	Green ontogenesis	
ppa022951m	-1.99	0.023475016	receptor serine/threonine kinase, putative	Green ontogenesis	
ppa013313m	-2.00	0.0087058	photosystem I subunit K	Green ontogenesis	Photosynthesis / Photosystem I
ppa009588m	-2.00	0.000124561	plasma membrane intrinsic protein 1B	Green ontogenesis	
ppa001638m	-2.01	0.002778435	potassium transporter 2	Green ontogenesis	
ppa012696m	-2.01	0.024922259		Green ontogenesis	
ppa008617m	-2.01	0.014406142	Vacuolar iron transporter (VIT) family protein	Green ontogenesis	
ppa001207m	-2.01	5.91E-05	PLAT/LH2 domain-containing lipoxygenase family protein	Green ontogenesis	
ppa010382m	-2.01	0.02945541	expansin A8	Green ontogenesis	
ppa004757m	-2.01	0.000181206	Exostosin family protein	Green ontogenesis	
ppa019531m	-2.02	0.002011992	BTB/POZ domain-containing protein	Green ontogenesis	
ppa014332m	-2.02	0.005399012		Green ontogenesis	
ppa012844m	-2.02	0.005103312	histone H2A 12	Green ontogenesis	
ppa005609m	-2.02	0.000249397	aldehyde dehydrogenase 3I1	Green ontogenesis	
ppa017228m	-2.03	0.02934532	phytochrome interacting factor 4	Green ontogenesis	
ppa012465m	-2.03	0.00782869	Lactoylglutathione lyase / glyoxalase I family protein	Green ontogenesis	
ppa007813m	-2.03	0.001732361	triosephosphate isomerase	Green ontogenesis	
ppa020844m	-2.03	0.01301438	alpha/beta-Hydrolases superfamily protein	Green ontogenesis	
ppa011777m	-2.03	0.009603062		Green ontogenesis	
ppa006845m	-2.03	0.006803011	Oxidoreductase, zinc-binding dehydrogenase family protein	Green ontogenesis	
ppa026687m	-2.03	0.028607121	Leucine-rich repeat protein kinase family protein	Green ontogenesis	
ppa004083m	-2.04	0.026777439	cytochrome P450, family 78, subfamily A, polypeptide 10	Green ontogenesis	
ppa010961m	-2.04	0.002682979	APS kinase	Green ontogenesis	
ppa023197m	-2.04	0.048572901	NAC 014	Green ontogenesis	
ppa004177m	-2.04	0.000905965		Green ontogenesis	
ppa020264m	-2.04	0.006749842	BURP domain-containing protein	Green ontogenesis	
ppa020132m	-2.04	0.009186664		Green ontogenesis	
ppa000606m	-2.04	0.011855536	aspartate kinase-homoserine dehydrogenase ii	Green ontogenesis	
ppa013538m	-2.05	0.002096342		Green ontogenesis	
ppa026592m	-2.05	0.043671485	Leucine-rich repeat transmembrane protein kinase protein	Green ontogenesis	
ppa016047m	-2.05	0.021429425	Calcium-binding EF-hand family protein	Green ontogenesis	
ppa015904m	-2.05	0.035569776	nodulin MtN21 /EamA-like transporter family protein	Green ontogenesis	
ppa003399m	-2.06	0.009920635	FKBP-type peptidyl-prolyl cis-trans isomerase family protein	Green ontogenesis	
ppa010226m	-2.06	0.044503361	Barwin-like endoglucanases superfamily protein	Green ontogenesis	
ppa018480m	-2.06	0.018749595		Green ontogenesis	
ppa012574m	-2.06	0.000891653	Protein of unknown function, DUF538	Green ontogenesis	
ppa024164m	-2.07	0.020627531	Protein kinase superfamily protein	Green ontogenesis	
ppa021648m	-2.07	0.00055197	Ankyrin repeat family protein	Green ontogenesis	
ppa016016m	-2.08	0.038519843	highly ABA-induced PP2C gene 2	Green ontogenesis	
ppa017576m	-2.08	0.024096514	bZIP protein	Green ontogenesis	
ppa004864m	-2.08	0.008679452	Protein of unknown function (DUF3754)	Green ontogenesis	
ppa012096m	-2.08	0.00447508	high mobility group A	Green ontogenesis	
ppa015959m	-2.08	0.033547651	Calcium-binding EF-hand family protein	Green ontogenesis	
ppa027024m	-2.08	0.006785101	NAC domain containing protein 83	Green ontogenesis	
ppa007423m	-2.09	0.005352781	Protein phosphatase 2C family protein	Green ontogenesis	
ppa017688m	-2.09	0.009982307	cytochrome P450, family 71, subfamily A, polypeptide 14	Green ontogenesis	
ppa017046m	-2.09	0.02920356	Phosphatidylinositol 3- and 4-kinase ;Ubiquitin family protein	Green ontogenesis	
ppa007107m	-2.09	0.012335679	GDSL-like Lipase/Acylhydrolase superfamily protein	Green ontogenesis	
ppa006788m	-2.09	0.003906136	protochlorophyllide oxidoreductase A	Green ontogenesis	
ppa013690m	-2.09	0.004125394	response to low sulfur 2	Green ontogenesis	
ppa010291m	-2.09	0.00100478	light-harvesting chlorophyll-protein complex I subunit A4	Green ontogenesis	Photosynthesis / Photosystem II
ppa010174m	-2.09	0.039880392	NAD(P)-binding Rossmann-fold superfamily protein	Green ontogenesis	
ppa014749m	-2.10	0.01938406		Green ontogenesis	
ppa006626m	-2.10	0.042621075	Galactosyltransferase family protein	Green ontogenesis	
ppa010278m	-2.10	0.003010139		Green ontogenesis	
ppa002011m	-2.10	0.036651236	oligopeptide transporter 2	Green ontogenesis	
ppa022089m	-2.10	0.004648784		Green ontogenesis	
ppa024251m	-2.10	0.003199469		Green ontogenesis	
ppa011853m	-2.10	0.000866576		Green ontogenesis	
ppa025547m	-2.10	0.043802771	Calcium-binding EF-hand family protein	Green ontogenesis	
ppa006348m	-2.10	0.001447883	Trypsin family protein with PDZ domain	Green ontogenesis	
ppa010963m	-2.10	0.005880251	Thioredoxin superfamily protein	Green ontogenesis	
ppa009133m	-2.10	0.00656793	RNA-binding (RRM/RBD/RNP motifs) family protein	Green ontogenesis	
ppa010560m	-2.10	0.008214002	alpha-crystallin domain 32.1	Green ontogenesis	
ppa025565m	-2.11	0.003295397	HXXXD-type acyl-transferase family protein	Green ontogenesis	Secondary Metabolism / Phenylpropanoids - Biotic Stress / Secondary metabolism involved into stress
ppa024543m	-2.11	0.039566154	osmotin 34	Green ontogenesis	
ppa020832m	-2.11	0.004647936	Phototropic-responsive NPH3 family protein	Green ontogenesis	
ppa002898m	-2.12	0.004005914	Protein of unknown function (DUF3049)	Green ontogenesis	
ppa011968m	-2.12	0.000269177	squamosa promoter binding protein-like 4	Green ontogenesis	
ppa015639m	-2.12	0.009967242	Cyclophilin-like peptidyl-prolyl cis-trans isomerase family protein	Green ontogenesis	
ppa011163m	-2.12	0.001889582	arabinogalactan protein 18	Green ontogenesis	
ppa022743m	-2.12	0.014722034	Protein of unknown function (DUF 3339)	Green ontogenesis	
ppa023527m	-2.13	0.001596228	Major facilitator superfamily protein	Green ontogenesis	
ppa000107m	-2.13	0.001641332	Kinase interacting (KIP1-like) family protein	Green ontogenesis	
ppa012521m	-2.14	0.000592624	plastocyanin 1	Green ontogenesis	Photosynthesis / Redox chain
ppa000958m	-2.14	0.033076476	Glycosyl transferase, family 35	Green ontogenesis	
ppa002869m	-2.15	0.005518555	glycosyltransferase family protein 47	Green ontogenesis	
ppa006774m	-2.15	0.000205856	Protein of unknown function (DUF506)	Green ontogenesis	
ppa011874m	-2.16	0.003811017		Green ontogenesis	
ppb020590m	-2.16	0.023573007	Galactose oxidase/kelch repeat superfamily protein	Green ontogenesis	
ppa009088m	-2.16	0.036780084	NmrA-like negative transcriptional regulator family protein	Green ontogenesis	Secondary Metabolism / Flavonols-Isoflavonoid -Biotic Stress / Secondary metabolism involved into stress
ppa009387m	-2.16	0.035874766	xyloglucan endotransglucosylase/hydrolase 5	Green ontogenesis	
ppa008391m	-2.16	0.006137374	alpha/beta-Hydrolases superfamily protein	Green ontogenesis	
ppa025594m	-2.17	0.000787198	Ankyrin repeat family protein	Green ontogenesis	
ppa016034m	-2.17	0.034198255		Green ontogenesis	
ppa008515m	-2.17	0.003041936	Pseudouridine synthase family protein	Green ontogenesis	
ppa010430m	-2.17	0.013451633	Protein of unknown function (DUF179)	Green ontogenesis	
ppa004142m	-2.19	0.007317315	Protein of unknown function (DUF604)	Green ontogenesis	
ppa007654m	-2.19	0.004473718	peroxidase 2	Green ontogenesis	
ppa005698m	-2.19	0.001549893	tubulin beta 8	Green ontogenesis	
ppa011732m	-2.19	0.007328351	NDH-dependent cyclic electron flow 1	Green ontogenesis	
ppa026947m	-2.20	0.001903748	nitrate excretion transporter1	Green ontogenesis	
ppa006812m	-2.20	0.003181723	Homeodomain-like superfamily protein	Green ontogenesis	
ppa013127m	-2.20	0.001106509	NADH-ubiquinone oxidoreductase-related	Green ontogenesis	
ppa000546m	-2.21	0.046169218	Myosin heavy chain-related protein	Green ontogenesis	
ppa005633m	-2.21	0.000822458	tubulin alpha-4 chain	Green ontogenesis	
ppa010781m	-2.21	0.015447688	Pathogenesis-related thaumatin superfamily protein	Green ontogenesis	
ppa004799m	-2.22	0.000183407	hydroperoxide lyase 1	Green ontogenesis	
ppa010243m	-2.22	0.01263243		Green ontogenesis	
ppa025759m	-2.23	0.025228749	Heavy metal transport/detoxification superfamily protein	Green ontogenesis	
ppa012417m	-2.23	0.001222526	HVA22 homologue A	Green ontogenesis	
ppa013033m	-2.23	0.001222526	actin depolymerizing factor 5	Green ontogenesis	
ppa010180m	-2.24	0.03921317	expansin A4	Green ontogenesis	
ppa025405m	-2.24	0.020594829		Green ontogenesis	
ppa020555m	-2.24	0.000373537	Uncharacterised protein family SERF	Green ontogenesis	
ppa011749m	-2.24	0.019545464	Polynucleotidyl transferase, ribonuclease H-like superfamily prote	Green ontogenesis	
ppa004373m	-2.24	0.001761169	cytochrome P450, family 77, subfamily A, polypeptide 4	Green ontogenesis	
ppa012366m	-2.25	0.019132936	early nodulin-like protein 17	Green ontogenesis	
ppa020872m	-2.25	0.008136511	Heavy metal transport/detoxification superfamily protein	Green ontogenesis	
ppa013987m	-2.25	0.005668058	Protein of unknown function (DUF3511)	Green ontogenesis	
ppa007278m	-2.25	0.033003463	BREVIS RADIX-like 4	Green ontogenesis	
ppa020222m	-2.25	0.024618403	Ankyrin repeat family protein	Green ontogenesis	
ppa009058m	-2.26	0.004616051	syntaxin of plants 131	Green ontogenesis	
ppa002735m	-2.26	0.000155769	Plant U-Box 15	Green ontogenesis	
ppa005472m	-2.26	0.013431626	P-loop containing nucleoside triphosphate hydrolases superfamily	Green ontogenesis	
ppa007891m	-2.26	0.003859734	xylem cysteine peptidase 1	Green ontogenesis	
ppa001671m	-2.26	0.028610836	Leucine-rich repeat protein kinase family protein	Green ontogenesis	
ppa022985m	-2.26	0.0285523		Green ontogenesis	
ppa017769m	-2.26	0.009602749	BURP domain-containing protein	Green ontogenesis	
ppa008874m	-2.27	0.024136129	BURP domain-containing protein	Green ontogenesis	
ppa008302m	-2.27	0.029803917	chloroplastic lipocalin	Green ontogenesis	
ppa017025m	-2.28	0.002349391	sigma factor 4	Green ontogenesis	
ppa006607m	-2.28	0.036592258		Green ontogenesis	
ppa005399m	-2.28	0.000919376	Plant protein of unknown function (DUF828) with plant pleckstrin I	Green ontogenesis	
ppa006511m	-2.30	0.005040321	NAD(P)-binding Rossmann-fold superfamily protein	Green ontogenesis	
ppa019480m	-2.30	0.003489886	osmotin 34	Green ontogenesis	
ppa009498m	-2.31	0.014842022	Homeobox-leucine zipper protein family	Green ontogenesis	
ppa002364m	-2.32	0.009945582	FTSH protease 6	Green ontogenesis	
ppa008991m	-2.32	0.001448624	Mitochondrial transcription termination factor family protein	Green ontogenesis	
ppa1027126m	-2.33	0.003775176	Ankyrin repeat family protein	Green ontogenesis	
ppa009649m	-2.33	0.000924519		Green ontogenesis	
ppa013898m	-2.33	0.000787198	GroES-like family protein	Green ontogenesis	
ppa013214m	-2.33	0.009602995		Green ontogenesis	
ppa009462m	-2.33	0.003589689	glyoxalase I homolog	Green ontogenesis	
ppa003185m	-2.34	0.004648784	Mannose-binding lectin superfamily protein	Green ontogenesis	
ppa026134m	-2.34	0.043713662	Leucine-rich receptor-like protein kinase family protein	Green ontogenesis	
ppa010395m	-2.34	0.007335849	Undecaprenyl pyrophosphate synthetase family protein	Green ontogenesis	
ppa010296m	-2.34	0.005261752	RING/U-box superfamily protein	Green ontogenesis	
ppa012978m	-2.34	0.000177107	Heavy metal transport/detoxification superfamily protein	Green ontogenesis	
ppa009903m	-2.35	0.005103312	photosystem I light harvesting complex gene 2	Green ontogenesis	Photosynthesis / Photosystem II
ppa009259m	-2.35	0.018989755	NagB/RpiA/CoA transferase-like superfamily protein	Green ontogenesis	
ppa010171m	-2.35	0.010487617	expansin-like A1	Green ontogenesis	

ppa006718m	-2.36	0.001136281	*Pectinacetylesterase family protein*	Green ontogenesis	
ppa014176m	-2.36	0.004983547		Green ontogenesis	
ppa004486m	-2.36	0.000787198	*Saposin-like aspartyl protease family protein*	Green ontogenesis	
ppa002885m	-2.36	0.00058789	*inter-alpha-trypsin inhibitor heavy chain-related*	Green ontogenesis	Biotic Stress / PR-proteins
ppa014221m	-2.36	0.006368595		Green ontogenesis	
ppa008517m	-2.37	0.001309569	*N-acetyl-l-glutamate kinase*	Green ontogenesis	
ppa026023m	-2.37	0.016906861	*protein kinase 2B*	Green ontogenesis	
ppa004498m	-2.38	0.002103065	*glycerol-3-phosphate acyltransferase 6*	Green ontogenesis	
ppa009496m	-2.39	0.003947305	*light harvesting complex of photosystem II 5*	Green ontogenesis	Photosynthesis / Photosystem II
ppa000942m	-2.39	0.000879999	*transmembrane kinase 1*	Green ontogenesis	
ppa009419m	-2.39	0.001563532	*homeobox protein 6*	Green ontogenesis	
ppa003519m	-2.40	0.002173511	*homeobox gene 1*	Green ontogenesis	
ppa012218m	-2.40	0.034086885	*Bifunctional inhibitor/lipid-transfer protein/seed storage 2S albumi*	Green ontogenesis	
ppa002955m	-2.40	0.006796102	*UDP-Glycosyltransferase superfamily protein*	Green ontogenesis	
ppa010426m	-2.40	0.001957779	*ascorbate peroxidase 2*	Green ontogenesis	
ppa024687m	-2.40	0.026793348	*Zinc-finger domain of monoamine-oxidase A repressor R1*	Green ontogenesis	
ppa009254m	-2.41	0.000876137	*indoleacetic acid-induced protein 16*	Green ontogenesis	
ppa003411m	-2.41	0.002193119	*Plant L-ascorbate oxidase*	Green ontogenesis	Biotic Stress / Secondary metabolism involved into stress
ppa004814m	-2.41	0.000353142	*O-Glycosyl hydrolases family 17 protein*	Green ontogenesis	Biotic Stress / Betaglucanase
ppa001082m	-2.41	0.028327578	*lipoxygenase 3*	Green ontogenesis	
ppa025053m	-2.42	0.047124914	*nodulin MtN21 /EamA-like transporter family protein*	Green ontogenesis	
ppa002356m	-2.42	0.004983547	*Leucine-rich repeat protein kinase family protein*	Green ontogenesis	
ppa016008m	-2.42	0.007005994	*nuclear factor Y, subunit A3*	Green ontogenesis	
ppa004732m	-2.43	0.005786488	*O-Glycosyl hydrolases family 17 protein*	Green ontogenesis	Biotic Stress / Betaglucanase
ppa012625m	-2.43	0.010110676	*RHO guanyl-nucleotide exchange factor 11*	Green ontogenesis	
ppa011791m	-2.44	0.00761725	*GLNB1 homolog*	Green ontogenesis	
ppb020370m	-2.44	0.007564199		Green ontogenesis	
ppa025159m	-2.44	0.019650129	*glycosyl hydrolase 9B18*	Green ontogenesis	
ppa013712m	-2.45	0.004987702	*Photosystem II 5 kD protein*	Green ontogenesis	
ppa023215m	-2.45	0.024359588	*asparaginyl-tRNA synthetase 2*	Green ontogenesis	
ppa003289m	-2.45	0.004381422	*S-adenosyl-L-methionine-dependent methyltransferases superfar*	Green ontogenesis	
ppa017935m	-2.45	0.02414721		Green ontogenesis	
ppa011240m	-2.45	0.007832965	*RING/U-box superfamily protein*	Green ontogenesis	
ppa004282m	-2.45	0.000116194	*non-specific phospholipase C2*	Green ontogenesis	
ppa024006m	-2.46	0.007047683	*Emsy N Terminus (ENT)/ plant Tudor-like domains-containing pro*	Green ontogenesis	
ppa003163m	-2.46	0.040694249	*Auxin-responsive GH3 family protein*	Green ontogenesis	
ppa004334m	-2.46	9.98E-05	*beta-amylase 6*	Green ontogenesis	
ppa010863m	-2.46	0.009723242	*response regulator 3*	Green ontogenesis	
ppa003360m	-2.47	0.024096514	*nitrate transporter 1:2*	Green ontogenesis	
ppa005531m	-2.47	0.001356283	*Protein kinase superfamily protein*	Green ontogenesis	
ppa002939m	-2.47	0.009472456	*glycosyl hydrolase 9C2*	Green ontogenesis	
ppa009599m	-2.48	3.84E-05	*Aldolase-type TIM barrel family protein*	Green ontogenesis	
ppa011095m	-2.49	0.03729472	*basic helix-loop-helix (bHLH) DNA-binding superfamily protein*	Green ontogenesis	
ppa024994m	-2.49	0.030234301	*Thioredoxin superfamily protein*	Green ontogenesis	
ppa018697m	-2.49	0.004680894	*UDP-glucosyl transferase 85A2*	Green ontogenesis	
ppa014209m	-2.49	0.000202279		Green ontogenesis	
ppa014177m	-2.49	0.002005583		Green ontogenesis	
ppa000511m	-2.50	0.000475695	*BCL-2-associated athanogene 6*	Green ontogenesis	
ppa019522m	-2.50	0.000993686	*myb domain protein 4*	Green ontogenesis	
ppa025262m	-2.50	0.042192182	*plastid transcriptionally active 17*	Green ontogenesis	
ppa022063m	-2.50	0.002294302	*Disease resistance-responsive (dirigent-like protein) family protei*	Green ontogenesis	Biotic Stress / PR-proteins
ppa003987m	-2.50	0.000831342	*Pre-mRNA splicing Prp18-interacting factor*	Green ontogenesis	
ppa005914m	-2.50	0.000237872	*microtubule-associated proteins 65-1*	Green ontogenesis	
ppa005869m	-2.50	0.027695173	*Protein of unknown function (DUF3411)*	Green ontogenesis	
ppa014957m	-2.50	0.004211532		Green ontogenesis	
ppa001340m	-2.51	0.001602832	*beta-galactosidase 8*	Green ontogenesis	
ppa009168m	-2.51	0.024326853	*Protein of unknown function (DUF579)*	Green ontogenesis	
ppa015700m	-2.52	0.007947079	*TRICHOME BIREFRINGENCE-LIKE 6*	Green ontogenesis	
ppa008881m	-2.52	0.000232773	*annexin 2*	Green ontogenesis	
ppa007056m	-2.52	0.003902017	*squamosa promoter binding protein-like 9*	Green ontogenesis	
ppa024453m	-2.53	0.010285876	*UDP-Glycosyltransferase superfamily protein*	Green ontogenesis	
ppa017289m	-2.53	0.011058051	*terpene synthase 21*	Green ontogenesis	Biotic Stress / Secondary metabolism involved into stress
ppa018224m	-2.53	0.004647936	*Pectin lyase-like superfamily protein*	Green ontogenesis	
ppa002341m	-2.53	0.043617335	*Protein of unknown function (DUF740)*	Green ontogenesis	
ppa005613m	-2.53	0.003248661	*tubulin alpha-4 chain*	Green ontogenesis	
ppa006510m	-2.53	0.00517658	*FAD-dependent oxidoreductase family protein*	Green ontogenesis	
ppa007562m	-2.53	0.001981886	*myb domain protein 31*	Green ontogenesis	
ppa009987m	-2.54	0.000612646	*light-harvesting chlorophyll B-binding protein 3*	Green ontogenesis	Photosynthesis / Photosystem II
ppa005895m	-2.54	0.003551152	*Class I glutamine amidotransferase-like superfamily protein*	Green ontogenesis	
ppa010394m	-2.54	0.002151548	*Nodulin MtN3 family protein*	Green ontogenesis	
ppa007004m	-2.54	0.033973189	*beta-hydroxyisobutyryl-CoA hydrolase 1*	Green ontogenesis	
ppa012243m	-2.55	0.00429102	*Protein of unknown function, DUF538*	Green ontogenesis	
ppa027099m	-2.55	0.006694596	*carboxyesterase 13*	Green ontogenesis	
ppa023795m	-2.55	0.001507339	*Zinc-binding alcohol dehydrogenase family protein*	Green ontogenesis	
ppa001023m	-2.56	0.014058244		Green ontogenesis	
ppa005486m	-2.56	0.007814623	*basic helix-loop-helix (bHLH) DNA-binding superfamily protein*	Green ontogenesis	
ppb013299m	-2.56	0.001938114	*terpene synthase 21*	Green ontogenesis	Biotic Stress / Secondary metabolism involved into stress
ppa013932m	-2.56	0.000454379		Green ontogenesis	
ppa012753m	-2.56	0.00048556		Green ontogenesis	
ppa002287m	-2.57	0.03310491	*Leucine-rich repeat protein kinase family protein*	Green ontogenesis	
ppa002670m	-2.57	0.000132814	*terpene synthase 14*	Green ontogenesis	Biotic Stress / Secondary metabolism involved into stress
ppa002893m	-2.58	0.004949546	*Fatty acid hydroxylase superfamily*	Green ontogenesis	Biotic Stress / Secondary metabolism involved into stress
ppa008483m	-2.58	0.000970401	*Pathogenesis-related thaumatin superfamily protein*	Green ontogenesis	
ppa011288m	-2.59	0.002151548	*PHD finger family protein / bromo-adjacent homology (BAH) doma*	Green ontogenesis	
ppa004023m	-2.59	0.034612024	*ARM repeat superfamily protein*	Green ontogenesis	
ppa013650m	-2.59	3.84E-05		Green ontogenesis	
ppa025853m	-2.60	0.022210841	*RING/U-box superfamily protein*	Green ontogenesis	
ppa018757m	-2.60	0.007814623	*Protein of unknown function (DUF761)*	Green ontogenesis	
ppa009253m	-2.61	0.003808867	*ARM repeat superfamily protein*	Green ontogenesis	
ppa018930m	-2.61	0.004224639	*GCK domain-containing protein*	Green ontogenesis	
ppa002413m	-2.62	0.048426341	*Zinc finger (CCCH-type) family protein / RNA recognition motif (R*	Green ontogenesis	
ppa012767m	-2.62	0.015184964	*Protein of unknown function (DUF581)*	Green ontogenesis	
ppa005557m	-2.62	0.008682423	*Cyclin A1;1*	Green ontogenesis	
ppa005992m	-2.62	0.017094022	*Glycosyl hydrolase superfamily protein*	Green ontogenesis	
ppa020996m	-2.62	0.009939938	*Fatty acid hydroxylase superfamily*	Green ontogenesis	Biotic Stress / Secondary metabolism involved into stress
ppa021800m	-2.63	0.010332964		Green ontogenesis	
ppa007187m	-2.63	0.039670153	*TRICHOME BIREFRINGENCE-LIKE 36*	Green ontogenesis	
ppa008167m	-2.63	0.043910042	*Nucleotide-sugar transporter family protein*	Green ontogenesis	
ppa022205m	-2.63	0.017794574	*myb domain protein 111*	Green ontogenesis	
ppa002824m	-2.64	0.014406142	*Fatty acid hydroxylase superfamily*	Green ontogenesis	Biotic Stress / Secondary metabolism involved into stress
ppa019128m	-2.65	0.011601598	*PLAC8 family protein*	Green ontogenesis	
ppa012052m	-2.65	0.043859299	*RING/U-box superfamily protein*	Green ontogenesis	
ppa001717m	-2.65	0.003859734	*Leucine-rich repeat protein kinase family protein*	Green ontogenesis	
ppa012409m	-2.65	0.007903212	*Chaperonin-like RbcX protein*	Green ontogenesis	
ppa008774m	-2.65	0.000567773	*homeobox protein 2*	Green ontogenesis	
ppa014596m	-2.66	0.03554699	*Cytochrome P450 superfamily protein*	Green ontogenesis	
ppa008510m	-2.67	7.39E-05	*plasma membrane intrinsic protein 1B*	Green ontogenesis	
ppa026283m	-2.67	0.005576065		Green ontogenesis	
ppa013043m	-2.68	0.001044603	*HSP20-like chaperones superfamily protein*	Green ontogenesis	
ppa009534m	-2.68	0.000185489	*NmrA-like negative transcriptional regulator family protein*	Green ontogenesis	Secondary Metabolism / Flavonols-Isoflavonoid -Biotic Stress / Secondary metabolism involved into stress
ppa003968m	-2.68	0.037020084	*Tetratricopeptide repeat (TPR)-like superfamily protein*	Green ontogenesis	
ppa007574m	-2.71	0.007958678	*GDSL-like Lipase/Acylhydrolase superfamily protein*	Green ontogenesis	
ppa012284m	-2.71	0.048384608	*LOB domain-containing protein 21*	Green ontogenesis	
ppa020538m	-2.71	0.001526743	*heat shock protein 70*	Green ontogenesis	
ppa003256m	-2.72	0.001126811	*IQ-domain 31*	Green ontogenesis	
ppa004915m	-2.72	0.000446532	*Major facilitator superfamily protein*	Green ontogenesis	
ppa022150m	-2.72	0.042372031	*F-box and associated interaction domains-containing protein*	Green ontogenesis	
ppa009489m	-2.73	0.006226707	*Protein of unknown function (DUF506)*	Green ontogenesis	
ppa020548m	-2.73	0.032610424		Green ontogenesis	
ppa023800m	-2.73	0.015561142		Green ontogenesis	
ppa004866m	-2.73	0.000768596	*Seven transmembrane MLO family protein*	Green ontogenesis	
ppa000340m	-2.74	0.009355779	*P-glycoprotein 2*	Green ontogenesis	
ppa025020m	-2.74	0.026999307	*O-acyltransferase (WSD1-like) family protein*	Green ontogenesis	
ppa022897m	-2.75	0.003548919		Green ontogenesis	
ppa007586m	-2.75	0.003037627	*Undecaprenyl pyrophosphate synthetase family protein*	Green ontogenesis	
ppa010820m	-2.76	0.024700367	*early nodulin-like protein 18*	Green ontogenesis	
ppa012595m	-2.76	0.002958966	*histidine-containing phosphotransmitter 1*	Green ontogenesis	
ppa002598m	-2.76	0.00063489	*heat shock protein 70*	Green ontogenesis	
ppa016850m	-2.77	0.005822079	*BURP domain-containing protein*	Green ontogenesis	
ppa017529m	-2.77	0.04162263	*REF4-related 1*	Green ontogenesis	
ppa017311m	-2.77	0.003107182	*BTB/POZ domain-containing protein*	Green ontogenesis	
ppa001770m	-2.77	0.001835024	*Subtilisin-like serine endopeptidase family protein*	Green ontogenesis	
ppa003918m	-2.78	0.019729806	*cellulose synthase-like A02*	Green ontogenesis	
ppa004823m	-2.78	0.000136527	*sodium/calcium exchanger family protein / calcium-binding EF ha*	Green ontogenesis	
ppa011379m	-2.78	0.000923751	*Protein of unknown function (DUF567)*	Green ontogenesis	
ppa002007m	-2.79	0.002223309		Green ontogenesis	
ppa013010m	-2.79	0.042755267	*Bifunctional inhibitor/lipid-transfer protein/seed storage 2S albumi*	Green ontogenesis	
ppa021836m	-2.79	0.032317882	*3-ketoacyl-CoA synthase 3*	Green ontogenesis	
ppa002259m	-2.79	0.000410934	*ferric reduction oxidase 7*	Green ontogenesis	
ppa011249m	-2.79	0.000231017	*Glutathione S-transferase family protein*	Green ontogenesis	Biotic Stress / Glutathione-S-Transferase
ppa011587m	-2.80	0.000547379		Green ontogenesis	
ppa003709m	-2.83	0.028644413	*Plant protein of unknown function (DUF827)*	Green ontogenesis	
ppa002088m	-2.83	0.01448622	*STRUBBELIG-receptor family 8*	Green ontogenesis	
ppa004243m	-2.83	0.040969373	*3-ketoacyl-CoA synthase 2*	Green ontogenesis	
ppa020549m	-2.83	0.019350144	*Protein phosphatase 2C family protein*	Green ontogenesis	
ppa014687m	-2.84	0.008214002	*ROP-interactive CRIB motif-containing protein 4*	Green ontogenesis	
ppa008267m	-2.84	0.014745887	*Cysteine proteinases superfamily protein*	Green ontogenesis	
ppa007395m	-2.84	0.001606805	*proline-rich protein 2*	Green ontogenesis	

ppa015930m	-2.84	0.00059776		Green ontogenesis	
ppa014473m	-2.85	0.002151337		Green ontogenesis	
ppa005184m	-2.85	0.002344551	*rubisco activase*	Green ontogenesis	
ppa004726m	-2.85	0.001141283	*Eukaryotic aspartyl protease family protein*	Green ontogenesis	
ppa025377m	-2.85	0.015056779		Green ontogenesis	
ppa021245m	-2.85	0.045408601	*Plant protein of unknown function (DUF828) with plant pleckstrin I*	Green ontogenesis	
ppa017173m	-2.86	0.010285876		Green ontogenesis	
ppa019292m	-2.86	0.01604268	*3-ketoacyl-CoA synthase 19*	Green ontogenesis	
ppa023764m	-2.86	0.000132814	*ADP glucose pyrophosphorylase large subunit 1*	Green ontogenesis	
ppa003131m	-2.86	0.026189885	*ABC-2 type transporter family protein*	Green ontogenesis	
ppa018776m	-2.86	0.009057953	*voltage dependent anion channel 1*	Green ontogenesis	
ppa002579m	-2.86	0.001434191	*Leucine-rich repeat protein kinase family protein*	Green ontogenesis	
ppa001386m	-2.87	0.008411757	*homeobox gene 8*	Green ontogenesis	
ppb013860m	-2.88	0.008520341		Green ontogenesis	
ppa001662m	-2.88	0.009288864	*EXS (ERD1/XPR1/SYG1) family protein*	Green ontogenesis	
ppa002215m	-2.88	0.007906639	*mechanosensitive channel of small conductance-like 10*	Green ontogenesis	
ppa022290m	-2.88	0.001589943	*BRI1-like 2*	Green ontogenesis	
ppa010884m	-2.88	0.000352421	*photosystem II subunit Q-2*	Green ontogenesis	Photosynthesis / Photosystem II
ppa005629m	-2.89	0.016113279	*basic helix-loop-helix (bHLH) DNA-binding superfamily protein*	Green ontogenesis	
ppa020997m	-2.90	0.014439235	*SPFH/Band 7/PHB domain-containing membrane-associated pro*	Green ontogenesis	
ppa025458m	-2.90	0.000724297	*Embryo-specific protein 3, (ATS3)*	Green ontogenesis	
ppa002781m	-2.91	0.015893896	*Leucine-rich repeat protein kinase family protein*	Green ontogenesis	
ppa020301m	-2.91	0.002821247	*RING/U-box superfamily protein*	Green ontogenesis	
ppa002275m	-2.91	0.026440733	*Cellulose-synthase-like C5*	Green ontogenesis	
ppa002978m	-2.91	0.027503567	*basic helix-loop-helix (bHLH) DNA-binding superfamily protein*	Green ontogenesis	
ppa004223m	-2.91	0.048710307	*Transmembrane amino acid transporter family protein*	Green ontogenesis	
ppa001579m	-2.91	0.001474634		Green ontogenesis	
ppa009969m	-2.92	0.043488749	*Tetraspanin family protein*	Green ontogenesis	
ppa023074m	-2.92	0.026096995	*Glycosyl hydrolase family protein with chitinase insertion domain*	Green ontogenesis	
ppa004238m	-2.93	0.013332524	*Domain of unknown function (DUF23)*	Green ontogenesis	
ppa005153m	-2.93	0.036763594	*Protein of Unknown Function (DUF239)*	Green ontogenesis	
ppa020243m	-2.94	0.000462685	*2-oxoglutarate (2OG) and Fe(II)-dependent oxygenase superfami*	Green ontogenesis	
ppa018358m	-2.95	0.041551008		Green ontogenesis	
ppa007096m	-2.95	0.004983547	*PQ-loop repeat family protein / transmembrane family protein*	Green ontogenesis	
ppa004043m	-2.95	0.001152163	*galacturonosyltransferase 12*	Green ontogenesis	
ppa005789m	-2.95	0.033978896	*Eukaryotic aspartyl protease family protein*	Green ontogenesis	
ppa022356m	-2.96	0.003181723	*UDP-Glycosyltransferase superfamily protein*	Green ontogenesis	
ppa027105m	-2.96	0.000131152	*Myzus persicae-induced lipase 1*	Green ontogenesis	
ppa010643m	-2.96	0.001309569	*Protein of unknown function (DUF674)*	Green ontogenesis	
ppa001918m	-2.96	0.000797658	*Subtilisin-like serine endopeptidase family protein*	Green ontogenesis	
ppa004191m	-2.97	0.030786854	*P-loop containing nucleoside triphosphate hydrolases superfamily*	Green ontogenesis	
ppa007155m	-2.97	0.011405587	*Protein of unknown function (DUF3049)*	Green ontogenesis	
ppa008481m	-2.97	0.001390383	*BURP domain-containing protein*	Green ontogenesis	
ppa004971m	-2.98	0.000963589	*aldehyde dehydrogenase 3F1*	Green ontogenesis	
ppa002595m	-2.98	0.000373537	*heat shock protein 70B*	Green ontogenesis	
ppa022777m	-2.98	0.005104247	*Cytochrome P450 superfamily protein*	Green ontogenesis	
ppa012806m	-2.99	0.000313173	*HSP20-like chaperones superfamily protein*	Green ontogenesis	
ppa017766m	-2.99	0.006148438	*Protein kinase superfamily protein*	Green ontogenesis	
ppa020645m	-2.99	0.042972492	*Haloacid dehalogenase-like hydrolase (HAD) superfamily protein*	Green ontogenesis	
ppa009785m	-3.00	0.004133906	*methyl esterase 17*	Green ontogenesis	
ppa013388m	-3.01	0.000547379	*Heavy metal transport/detoxification superfamily protein*	Green ontogenesis	
ppa016854m	-3.01	0.010687395		Green ontogenesis	
ppa017136m	-3.01	0.010842757	*myb domain protein 12*	Green ontogenesis	
ppa021753m	-3.02	0.013332524	*cell wall / vacuolar inhibitor of fructosidase 2*	Green ontogenesis	
ppa007755m	-3.02	0.000373537	*decapping 1*	Green ontogenesis	
ppa004078m	-3.03	0.000315635	*cytochrome P450, family 86, subfamily A, polypeptide 8*	Green ontogenesis	
ppa017874m	-3.03	0.00497105	*PLAC8 family protein*	Green ontogenesis	
ppa024148m	-3.03	0.012002202	*FAD-binding Berberine family protein*	Green ontogenesis	
ppa011843m	-3.04	0.004342991	*AUX/IAA transcriptional regulator family protein*	Green ontogenesis	
ppa005150m	-3.04	0.013679985	*6-phosphogluconate dehydrogenase family protein*	Green ontogenesis	
ppa020149m	-3.04	0.006391302	*alpha dioxygenase*	Green ontogenesis	
ppa023171m	-3.05	0.038350798	*isopentenyltransferase 3*	Green ontogenesis	
ppa002732m	-3.05	0.000636336	*Glycosyl hydrolases family 32 protein*	Green ontogenesis	
ppa000363m	-3.05	0.002164371	*P-glycoprotein 13*	Green ontogenesis	
ppa002536m	-3.05	0.032112673	*receptor-like kinase 1*	Green ontogenesis	
ppa008289m	-3.06	0.0019245	*carbonic anhydrase 1*	Green ontogenesis	
ppa025334m	-3.07	0.002151548	*Transmembrane amino acid transporter family protein*	Green ontogenesis	
ppa024312m	-3.08	0.000131152	*Sec14p-like phosphatidylinositol transfer family protein*	Green ontogenesis	
ppa008733m	-3.08	0.000259824		Green ontogenesis	
ppa004192m	-3.08	0.004470847	*Glucose-1-phosphate adenylyltransferase family protein*	Green ontogenesis	
ppa002903m	-3.09	0.016525088	*Fatty acid hydroxylase superfamily*	Green ontogenesis	Biotic Stress / Secondary metabolism involved into stress
ppa001828m	-3.09	0.046290906	*Subtilisin-like serine endopeptidase family protein*	Green ontogenesis	
ppa026717m	-3.09	0.019925953		Green ontogenesis	
ppa018533m	-3.09	0.002096342	*DNA binding;ATP binding*	Green ontogenesis	
ppa022171m	-3.09	0.010318671	*heat shock protein 70*	Green ontogenesis	
ppa010456m	-3.10	7.44E-05	*Heavy metal transport/detoxification superfamily protein*	Green ontogenesis	
ppa015101m	-3.10	0.049475783	*O-methyltransferase family protein*	Green ontogenesis	
ppa019426m	-3.10	0.015711038	*ADP-glucose pyrophosphorylase small subunit 2*	Green ontogenesis	
ppa024725m	-3.11	0.024204842	*cellulose synthase family protein*	Green ontogenesis	
ppa011610m	-3.12	0.000497826	*Late embryogenesis abundant (LEA) hydroxyproline-rich glycopro*	Green ontogenesis	
ppa001168m	-3.12	0.00126566	*alpha-xylosidase 1*	Green ontogenesis	
ppa003537m	-3.12	0.030234301	*Protein of unknown function (DUF668)*	Green ontogenesis	
ppa014063m	-3.12	0.01200695	*Plant EC metallothionein-like protein, family 15*	Green ontogenesis	
ppa018164m	-3.13	0.031761212		Green ontogenesis	
ppa000925m	-3.15	0.011355593	*ATP binding microtubule motor family protein*	Green ontogenesis	
ppa012792m	-3.15	0.006984153		Green ontogenesis	
ppa016415m	-3.15	0.000379686	*Heavy metal transport/detoxification superfamily protein*	Green ontogenesis	
ppa016605m	-3.15	0.031828964	*zeaxanthin epoxidase (ZEP) (ABA1)*	Green ontogenesis	
ppa014256m	-3.16	0.000249397	*Uncharacterised protein family SERF*	Green ontogenesis	
ppa004515m	-3.16	0.0019245	*dehydroquinate dehydratase, putative / shikimate dehydrogenase*	Green ontogenesis	
ppa007085m	-3.16	0.03264987	*PATATIN-like protein 9*	Green ontogenesis	
ppa021980m	-3.16	0.018559253	*3-ketoacyl-CoA synthase 19*	Green ontogenesis	
ppb011681m	-3.16	0.030234301	*Major facilitator superfamily protein*	Green ontogenesis	
ppa011989m	-3.16	0.000282055	*Lipase/lipooxygenase, PLAT/LH2 family protein*	Green ontogenesis	
ppa016220m	-3.17	0.031869628	*Basic helix-loop-helix (bHLH) DNA-binding family protein*	Green ontogenesis	
ppa022028m	-3.17	0.030404573	*Ankyrin repeat family protein*	Green ontogenesis	
ppa003796m	-3.17	0.001006662	*Phototropic-responsive NPH3 family protein*	Green ontogenesis	
ppa1027188m	-3.18	5.96E-05	*Phototropic-responsive NPH3 family protein*	Green ontogenesis	
ppa010695m	-3.18	0.02815925	*plasmodesmata callose-binding protein 3*	Green ontogenesis	Biotic Stress / Betaglucanase
ppa005901m	-3.18	0.003135797	*Amino acid permease family protein*	Green ontogenesis	
ppa013120m	-3.18	0.003300153	*Bifunctional inhibitor/lipid-transfer protein/seed storage 2S albumi*	Green ontogenesis	
ppa007640m	-3.20	0.021699478	*Nucleotide-diphospho-sugar transferases superfamily protein*	Green ontogenesis	
ppa005581m	-3.20	0.000462685	*HXXXD-type acyl-transferase family protein*	Green ontogenesis	
ppa026075m	-3.20	0.021516389	*Carbohydrate-binding X8 domain superfamily protein*	Green ontogenesis	Biotic Stress / Betaglucanase
ppa009245m	-3.21	0.002147264	*xyloglucan endotransglucosylase/hydrolase 8*	Green ontogenesis	
ppa018700m	-3.22	0.000916598	*Plant basic secretory protein (BSP) family protein*	Green ontogenesis	
ppa019781m	-3.22	0.00991855	*TRAM, LAG1 and CLN8 (TLC) lipid-sensing domain containing pr*	Green ontogenesis	
ppa003769m	-3.23	0.010780086	*rho guanyl-nucleotide exchange factor 1*	Green ontogenesis	
ppa012360m	-3.23	8.91E-05	*Polyketide cyclase/dehydrase and lipid transport superfamily prot*	Green ontogenesis	
ppa012712m	-3.23	0.044227865	*HVA22-like protein F*	Green ontogenesis	
ppa016399m	-3.23	0.000831342	*heat shock protein 70*	Green ontogenesis	
ppa013271m	-3.25	0.005407169		Green ontogenesis	
ppa004380m	-3.25	0.01205993	*B-S glucosidase 44*	Green ontogenesis	
ppa017485m	-3.25	0.003067522	*ubiquitin-specific protease 12*	Green ontogenesis	
ppa008352m	-3.25	0.034876953	*Nucleotide/sugar transporter family protein*	Green ontogenesis	
ppa021929m	-3.25	0.032728465	*chromatin remodeling 4*	Green ontogenesis	
ppa026448m	-3.26	0.01934855	*beta glucosidase 17*	Green ontogenesis	
ppa024899m	-3.26	0.015061871	*Gibberellin-regulated family protein*	Green ontogenesis	
ppa022210m	-3.27	0.038497023	*IQ-domain 19*	Green ontogenesis	
ppa007765m	-3.28	0.008881237	*Leucine-rich repeat (LRR) family protein*	Green ontogenesis	
ppa006355m	-3.28	0.001797555		Green ontogenesis	
ppa004056m	-3.28	0.034686832	*TRICHOME BIREFRINGENCE-LIKE 6*	Green ontogenesis	
ppa011460m	-3.28	0.022287138	*germin 3*	Green ontogenesis	
ppa003860m	-3.29	0.000927104	*SKU5 similar 5*	Green ontogenesis	
ppa025166m	-3.31	0.007008449	*cytochrome P450, family 94, subfamily C, polypeptide 1*	Green ontogenesis	
ppa010472m	-3.31	0.000841883	*delta tonoplast integral protein*	Green ontogenesis	
ppa008626m	-3.31	0.020856434		Green ontogenesis	
ppa007863m	-3.32	0.026738905	*nodulin MtN21 /EamA-like transporter family protein*	Green ontogenesis	
ppa022380m	-3.32	0.008163501	*B-box zinc finger family protein*	Green ontogenesis	
ppa017673m	-3.32	0.009969838	*kinesin-like protein 1*	Green ontogenesis	
ppa005790m	-3.32	0.03136435	*Transmembrane amino acid transporter family protein*	Green ontogenesis	
ppa015726m	-3.33	0.042674616		Green ontogenesis	
ppa025804m	-3.33	0.018889364	*cytokinin response factor 2*	Green ontogenesis	
ppa023443m	-3.34	0.005553602		Green ontogenesis	
ppa003572m	-3.34	0.010110676	*laccase 11*	Green ontogenesis	Biotic Stress / Secondary metabolism involved into stress
ppa003135m	-3.34	0.023995737	*NIMA-related kinase 2*	Green ontogenesis	
ppa026780m	-3.34	0.033547651		Green ontogenesis	
ppa004109m	-3.34	0.002539664	*Leucine-rich repeat (LRR) family protein*	Green ontogenesis	
ppa001656m	-3.35	0.039304171	*beta-xylosidase 2*	Green ontogenesis	
ppa017738m	-3.35	0.000316968	*heat shock protein 70*	Green ontogenesis	
ppa019321m	-3.36	0.01687727	*DNA binding;ATP binding*	Green ontogenesis	
ppa016532m	-3.36	0.038334607	*myb domain protein 82*	Green ontogenesis	
ppa022265m	-3.36	0.037173747	*Myzus persicae-induced lipase 1*	Green ontogenesis	
ppa015817m	-3.36	0.014954422	*heat shock protein 70*	Green ontogenesis	
ppa019278m	-3.36	0.003165754	*wall-associated kinase 2*	Green ontogenesis	
ppa011816m	-3.37	0.00208723		Green ontogenesis	
ppa017084m	-3.37	0.011849628	*Peroxidase superfamily protein*	Green ontogenesis	
ppa013602m	-3.38	0.00321474	*arabinogalactan protein 1*	Green ontogenesis	

ppa026307m	-3.38	0.018895016	*Rhamnogalacturonate lyase family protein*	Green ontogenesis	
ppa002368m	-3.39	4.68E-05	*YELLOW STRIPE like 7*	Green ontogenesis	
ppa009108m	-3.39	0.035186586		Green ontogenesis	
ppa025376m	-3.40	0.022795297	*UDP-Glycosyltransferase superfamily protein*	Green ontogenesis	
ppa002591m	-3.40	3.76E-05	*heat shock protein 70*	Green ontogenesis	
ppa022440m	-3.41	0.044460297	*Laccase/Diphenol oxidase family protein*	Green ontogenesis	Biotic Stress / Secondary metabolism involved into stress
ppa012607m	-3.41	0.000327533	*squamosa promoter binding protein-like 3*	Green ontogenesis	
ppa026419m	-3.42	0.029929929		Green ontogenesis	
ppa025195m	-3.42	0.000221564	*fructose-bisphosphate aldolase 2*	Green ontogenesis	
ppa011466m	-3.42	0.000527578	*HSP20-like chaperones superfamily protein*	Green ontogenesis	
ppa021488m	-3.43	0.007765812		Green ontogenesis	
ppa003643m	-3.43	0.007096582	*RHO guanyl-nucleotide exchange factor 14*	Green ontogenesis	
ppa010905m	-3.43	0.004243163	*Plant-specific transcription factor YABBY family protein*	Green ontogenesis	
ppa009823m	-3.43	0.026765927	*Protein kinase superfamily protein*	Green ontogenesis	
ppa016999m	-3.43	0.001129143	*P-loop containing nucleoside triphosphate hydrolases superfamily*	Green ontogenesis	
ppa012060m	-3.44	2.09E-05	*Bifunctional inhibitor/lipid-transfer protein/seed storage 2S albumi*	Green ontogenesis	
ppa023552m	-3.45	0.001543593	*alkenal reductase*	Green ontogenesis	
ppa006363m	-3.46	0.012171592	*tubby like protein 6*	Green ontogenesis	
ppa002803m	-3.47	0.001384146	*Phototropic-responsive NPH3 family protein*	Green ontogenesis	
ppa020782m	-3.48	0.000109333	*transducin family protein / WD-40 repeat family protein*	Green ontogenesis	
ppa022667m	-3.49	0.004983547	*IQ calmodulin-binding motif family protein*	Green ontogenesis	
ppa010555m	-3.50	0.016467777	*Cytochrome b561/ferric reductase transmembrane protein family*	Green ontogenesis	
ppa023757m	-3.50	0.046025379	*S-adenosyl-L-methionine-dependent methyltransferases superfar*	Green ontogenesis	
ppa025447m	-3.51	0.004882685	*potassium transporter 1*	Green ontogenesis	
ppa010306m	-3.51	0.006570654	*Immunoglobulin E-set superfamily protein*	Green ontogenesis	
ppa003540m	-3.51	0.007619441	*polyamine oxidase 5*	Green ontogenesis	
ppa009645m	-3.52	0.000282055	*general regulatory factor 11*	Green ontogenesis	
ppa022655m	-3.52	0.006796102		Green ontogenesis	
ppa010373m	-3.52	0.003273923	*response regulator 9*	Green ontogenesis	
ppa008503m	-3.52	0.001407513	*Peroxidase superfamily protein*	Green ontogenesis	
ppa002612m	-3.52	0.018147781	*Integrase-type DNA-binding superfamily protein*	Green ontogenesis	
ppa023678m	-3.53	0.03012191		Green ontogenesis	
ppa009815m	-3.53	0.003553862	*GDSL-like Lipase/Acylhydrolase superfamily protein*	Green ontogenesis	
ppa022264m	-3.54	0.001352218	*vascular related NAC-domain protein 1*	Green ontogenesis	
ppa001363m	-3.54	0.00384776	*beta-galactosidase 3*	Green ontogenesis	
ppa004291m	-3.55	0.0021542	*MATE efflux family protein*	Green ontogenesis	
ppa001116m	-3.56	0.002118342	*formin homology 1*	Green ontogenesis	
ppa018788m	-3.57	0.002414148	*Plant protein of unknown function (DUF247)*	Green ontogenesis	
ppa017434m	-3.58	0.001631799	*Ankyrin repeat family protein*	Green ontogenesis	
ppa011735m	-3.58	0.031360066	*Integrase-type DNA-binding superfamily protein*	Green ontogenesis	
ppa018641m	-3.59	0.013368121	*Pectinacetylesterase family protein*	Green ontogenesis	
ppa012383m	-3.60	0.033697934	*Family of unknown function (DUF662)*	Green ontogenesis	
ppa008244m	-3.60	0.003218245	*NAD(P)-binding Rossmann-fold superfamily protein*	Green ontogenesis	Secondary Metabolism / Dihydroflavonols -Biotic Stress / Secondary metabolism involved into stress
ppa008896m	-3.61	0.000419946	*Peroxidase superfamily protein*	Green ontogenesis	
ppa026358m	-3.62	0.000422557	*beta glucosidase 15*	Green ontogenesis	
ppa015713m	-3.62	0.004828722	*Xyloglucan endotransglucosylase/hydrolase family protein*	Green ontogenesis	
ppa006621m	-3.63	0.000495785	*ethylene-dependent gravitropism-deficient and yellow-green-like*	Green ontogenesis	
ppa003791m	-3.63	0.005930183	*Heavy metal transport/detoxification superfamily protein*	Green ontogenesis	
ppa016309m	-3.64	0.007716298	*Ankyrin repeat family protein*	Green ontogenesis	
ppa020402m	-3.64	0.000177936	*heat shock protein 70*	Green ontogenesis	
ppb024555m	-3.65	0.000604791	*Myzus persicae-induced lipase 1*	Green ontogenesis	
ppa025611m	-3.66	0.006467631	*Myzus persicae-induced lipase 1*	Green ontogenesis	
ppa003034m	-3.66	0.001903748	*ROP interactive partner 3*	Green ontogenesis	
ppa002812m	-3.66	0.014299915	*Leucine-rich repeat protein kinase family protein*	Green ontogenesis	
ppa014775m	-3.66	0.00105883	*Leucine-rich repeat (LRR) family protein*	Green ontogenesis	
ppa005749m	-3.67	0.018379088	*purple acid phosphatase 22*	Green ontogenesis	
ppa018789m	-3.67	0.002164371	*Leucine-rich receptor-like protein kinase family protein*	Green ontogenesis	
ppa010958m	-3.67	0.006899574	*Bifunctional inhibitor/lipid-transfer protein/seed storage 2S albumi*	Green ontogenesis	
ppa002995m	-3.67	0.000724297	*UDP-Glycosyltransferase superfamily protein*	Green ontogenesis	
ppa004724m	-3.68	0.003416073	*ROP guanine nucleotide exchange factor 5*	Green ontogenesis	
ppa003285m	-3.69	0.000299927	*pectin methylesterase 3*	Green ontogenesis	
ppa023379m	-3.69	0.031891924	*FASCICLIN-like arabinogalactan-protein 12*	Green ontogenesis	
ppa003433m	-3.70	0.029271861	*pectin methylesterase 61*	Green ontogenesis	
ppa025552m	-3.70	0.036023302	*zinc finger (C2H2 type) family protein*	Green ontogenesis	
ppa005430m	-3.70	0.004983547	*COBRA-like extracellular glycosyl-phosphatidyl inositol-anchored*	Green ontogenesis	
ppa022094m	-3.71	0.016360803		Green ontogenesis	
ppa014618m	-3.71	0.000433236	*Protein of Unknown Function (DUF239)*	Green ontogenesis	
ppa015783m	-3.72	0.0008032		Green ontogenesis	
ppa004251m	-3.72	0.000440126	*heat shock protein 70*	Green ontogenesis	
ppa022172m	-3.73	0.002820431	*Protein kinase superfamily protein*	Green ontogenesis	
ppa011523m	-3.73	0.010729187	*BURP domain-containing protein*	Green ontogenesis	
ppa016669m	-3.73	0.012539684	*P-loop containing nucleoside triphosphate hydrolases superfamily*	Green ontogenesis	
ppa018373m	-3.73	0.005498934	*Plant protein of unknown function (DUF247)*	Green ontogenesis	
ppa023388m	-3.73	0.00800644	*PAK-box/P21-Rho-binding family protein*	Green ontogenesis	Biotic Stress / PR-proteins
ppa019861m	-3.75	0.025548979	*P-loop containing nucleoside triphosphate hydrolases superfamily*	Green ontogenesis	
ppa017898m	-3.75	0.005199178	*ankyrin repeat family protein*	Green ontogenesis	
ppa000370m	-3.76	0.028890665		Green ontogenesis	
ppa026470m	-3.76	0.000765741	*BTB/POZ domain-containing protein*	Green ontogenesis	
ppa018603m	-3.77	0.024359588	*RNA-binding (RRM/RBD/RNP motifs) family protein*	Green ontogenesis	
ppa009543m	-3.77	0.040636969	*BIG PETAL P*	Green ontogenesis	
ppa007966m	-3.78	0.000596145	*EXORDIUM like 5*	Green ontogenesis	
ppb010066m	-3.79	0.003562927		Green ontogenesis	
ppa004428m	-3.79	0.01376687	*Eukaryotic aspartyl protease family protein*	Green ontogenesis	
ppa013300m	-3.79	0.00021527	*Stress responsive A/B Barrel Domain*	Green ontogenesis	
ppa016155m	-3.81	0.000399037	*Plant protein of unknown function (DUF247)*	Green ontogenesis	
ppa012692m	-3.82	0.000106309	*HSP20-like chaperones superfamily protein*	Green ontogenesis	
ppa022833m	-3.82	0.031070621	*18S pre-ribosomal assembly protein gar2-related*	Green ontogenesis	
ppa010908m	-3.83	0.015025204	*myb domain protein 66*	Green ontogenesis	
ppa012702m	-3.84	9.56E-05	*HSP20-like chaperones superfamily protein*	Green ontogenesis	
ppa002373m	-3.85	0.000931983	*Leucine-rich repeat protein kinase family protein*	Green ontogenesis	
ppa012494m	-3.85	0.000136284	*Bifunctional inhibitor/lipid-transfer protein/seed storage 2S albumi*	Green ontogenesis	
ppa019976m	-3.88	0.007442625		Green ontogenesis	
ppa016247m	-3.89	0.001658487	*wall-associated kinase 2*	Green ontogenesis	
ppa017614m	-3.90	0.00208723		Green ontogenesis	
ppa001379m	-3.91	0.000526087	*potassium transport 2/3*	Green ontogenesis	
ppa001484m	-3.92	0.000518736	*Ribonuclease P protein subunit P38-related*	Green ontogenesis	
ppa006868m	-3.92	0.01953566	*NAD(P)-binding Rossmann-fold superfamily protein*	Green ontogenesis	
ppa001571m	-3.92	0.006885816	*Exostosin family protein*	Green ontogenesis	
ppa017225m	-3.93	0.041374526		Green ontogenesis	
ppa006773m	-3.93	0.02389585	*CYCLIN D3;1*	Green ontogenesis	
ppa012620m	-3.93	0.000433909	*HSP20-like chaperones superfamily protein*	Green ontogenesis	
ppa020173m	-3.94	0.019132936	*dsRNA-binding protein 5*	Green ontogenesis	
ppa006883m	-3.95	0.017068032	*Protein of unknown function (DUF620)*	Green ontogenesis	
ppa023990m	-3.95	0.046237721	*UDP-glucosyltransferase 74F2*	Green ontogenesis	
ppa006158m	-3.96	0.002151548	*FASCICLIN-like arabinogalactan protein 8*	Green ontogenesis	
ppa011144m	-3.97	0.002594078	*RmlC-like cupins superfamily protein*	Green ontogenesis	
ppa024071m	-3.97	0.007313197	*cyclic nucleotide gated channel 8*	Green ontogenesis	
ppa010264m	-3.98	0.00018919	*NC domain-containing protein-related*	Green ontogenesis	
ppa023539m	-3.99	0.013525815	*amino acid permease 2*	Green ontogenesis	
ppa020184m	-3.99	0.014846084	*Aldolase-type TIM barrel family protein*	Green ontogenesis	
ppa017486m	-3.99	0.001390667	*Plant protein of unknown function (DUF247)*	Green ontogenesis	
ppa013480m	-4.00	0.005830971	*GAST1 protein homolog 1*	Green ontogenesis	
ppa008643m	-4.01	0.004622112	*chitinase-like protein 2*	Green ontogenesis	
ppa022695m	-4.02	0.004321453	*Plant protein of unknown function (DUF247)*	Green ontogenesis	
ppa017879m	-4.02	3.52E-05	*TRICHOME BIREFRINGENCE-LIKE 6*	Green ontogenesis	
ppa011156m	-4.02	0.000547379	*17.6 kDa class II heat shock protein*	Green ontogenesis	
ppa004500m	-4.03	0.003589689	*3-ketoacyl-CoA synthase 4*	Green ontogenesis	
ppa012311m	-4.04	0.000716809		Green ontogenesis	
ppa019380m	-4.04	0.008817703	*Duplicated homeodomain-like superfamily protein*	Green ontogenesis	
ppa015344m	-4.05	6.59E-06	*HSP20-like chaperones superfamily protein*	Green ontogenesis	
ppa011935m	-4.05	0.001136153	*indole-3-acetic acid inducible 19*	Green ontogenesis	
ppa023908m	-4.07	0.014838433	*Integrase-type DNA-binding superfamily protein*	Green ontogenesis	
ppa002298m	-4.08	0.000311721	*white-brown complex homolog protein 11*	Green ontogenesis	
ppb024787m	-4.08	0.039078792		Green ontogenesis	
ppa003735m	-4.10	0.01921438	*transmembrane kinase-like 1*	Green ontogenesis	
ppa019215m	-4.13	0.045908269	*Cytochrome P450 superfamily protein*	Green ontogenesis	
ppa004101m	-4.13	0.000816433	*BURP domain-containing protein*	Green ontogenesis	
ppa013476m	-4.15	0.000269177	*thioredoxin H-type 1*	Green ontogenesis	
ppa014614m	-4.15	0.008587072	*Uncharacterised protein family (UPF0497)*	Green ontogenesis	
ppa000889m	-4.15	0.006278198	*Leucine-rich repeat protein kinase family protein*	Green ontogenesis	
ppa001661m	-4.16	0.005064733	*Subtilase family protein*	Green ontogenesis	
ppa010379m	-4.17	0.007365742	*expansin A1*	Green ontogenesis	
ppa001674m	-4.18	0.000655284	*Subtilase family protein*	Green ontogenesis	
ppa018773m	-4.18	0.006803011	*BTB/POZ domain-containing protein*	Green ontogenesis	
ppa001174m	-4.19	0.026092316	*Leucine-rich repeat protein kinase family protein*	Green ontogenesis	
ppa019518m	-4.19	0.006696599	*beta glucosidase 15*	Green ontogenesis	
ppa022053m	-4.20	0.01253152	*O-acyltransferase (WSD1-like) family protein*	Green ontogenesis	
ppa026745m	-4.21	0.007328351	*Peptidase M28 family protein*	Green ontogenesis	
ppa020287m	-4.21	0.001421454	*GRAM domain family protein*	Green ontogenesis	
ppa006779m	-4.21	0.036780084	*Pectin lyase-like superfamily protein*	Green ontogenesis	
ppa019992m	-4.22	0.000311538	*BURP domain-containing protein*	Green ontogenesis	
ppb012141m	-4.22	0.009371205	*alpha/beta-Hydrolases superfamily protein*	Green ontogenesis	
ppa021557m	-4.22	0.021401341	*Transducin/WD40 repeat-like superfamily protein*	Green ontogenesis	
ppa020929m	-4.23	0.004948865	*myosin-like protein XIF*	Green ontogenesis	
ppa023913m	-4.25	0.012782774	*GDSL-like Lipase/Acylhydrolase superfamily protein*	Green ontogenesis	
ppa005457m	-4.25	0.015061871	*serine carboxypeptidase-like 45*	Green ontogenesis	
ppa018952m	-4.25	0.004773435		Green ontogenesis	
ppa014608m	-4.26	0.008980774	*NAD(P)-binding Rossmann-fold superfamily protein*	Green ontogenesis	

ppa017822m	-4.26	0.009720246	Ankyrin repeat family protein	Green ontogenesis	
ppa023999m	-4.26	0.004470847	FASCICLIN-like arabinogalactan-protein 11	Green ontogenesis	
ppa006443m	-4.27	0.00060255	SEC14 cytosolic factor family protein / phosphoglyceride transfer	Green ontogenesis	
ppa025618m	-4.27	0.016658462	O-methyltransferase family protein	Green ontogenesis	
ppa016265m	-4.28	0.002511476	Plant protein of unknown function (DUF247)	Green ontogenesis	
ppa006039m	-4.28	0.004695941	zinc finger protein-related	Green ontogenesis	
ppa010997m	-4.29	0.007033807		Green ontogenesis	
ppa026680m	-4.29	0.011981571		Green ontogenesis	
ppa022423m	-4.31	0.000923605		Green ontogenesis	
ppa006620m	-4.31	0.004125394	BURP domain-containing protein	Green ontogenesis	
ppa013553m	-4.34	0.000202279		Green ontogenesis	
ppa025624m	-4.34	0.001044603	glucuronidase 1	Green ontogenesis	
ppa012444m	-4.34	0.004124253	Plant invertase/pectin methylesterase inhibitor superfamily protein	Green ontogenesis	
ppa003501m	-4.35	1.73E-05	heat-shock protein 70T-2	Green ontogenesis	
ppa010836m	-4.35	0.001594153	HSP20-like chaperones superfamily protein	Green ontogenesis	
ppa023082m	-4.35	0.017041433	Leucine-rich repeat (LRR) family protein	Green ontogenesis	
ppa015092m	-4.35	0.000561887	Ankyrin repeat family protein	Green ontogenesis	
ppb013713m	-4.36	0.008965363		Green ontogenesis	
ppa014773m	-4.36	0.018147781		Green ontogenesis	
ppa002624m	-4.36	0.042174995		Green ontogenesis	
ppa009338m	-4.37	0.000472789	HMG (high mobility group) box protein with ARID/BRIGHT DNA-b	Green ontogenesis	
ppa012975m	-4.39	0.007248116		Green ontogenesis	
ppa012222m	-4.40	4.82E-05	HVA22 homologue C	Green ontogenesis	
ppa006799m	-4.40	0.048572901	Cytochrome b561/ferric reductase transmembrane with DOMON	Green ontogenesis	
ppa010249m	-4.41	0.000114299	K-box region and MADS-box transcription factor family protein	Green ontogenesis	
ppa004311m	-4.42	0.008023014	cytochrome P450, family 94, subfamily B, polypeptide 3	Green ontogenesis	
ppa003971m	-4.43	0.000116259	glycerol-3-phosphate acyltransferase 3	Green ontogenesis	
ppa024900m	-4.44	0.001132983	2-oxoglutarate (2OG) and Fe(II)-dependent oxygenase superfami	Green ontogenesis	
ppa019187m	-4.46	0.015079006	Ankyrin repeat family protein	Green ontogenesis	
ppa002683m	-4.47	0.000205527	Leucine-rich repeat protein kinase family protein	Green ontogenesis	
ppa026145m	-4.47	0.009969838		Green ontogenesis	
ppa005764m	-4.47	0.001473692		Green ontogenesis	
ppa021606m	-4.50	0.006866994	Serine/threonine-protein kinase WNK (With No Lysine)-related	Green ontogenesis	
ppa003646m	-4.50	0.005087366	Laccase/Diphenol oxidase family protein	Green ontogenesis	Biotic Stress / Secondary metabolism involved into stress
ppa010654m	-4.50	0.014439235	expansin A15	Green ontogenesis	
ppa001123m	-4.51	0.011484834	Leucine-rich repeat protein kinase family protein	Green ontogenesis	
ppa026605m	-4.51	0.033046792	cytochrome p450 79a2	Green ontogenesis	
ppa020715m	-4.52	0.000944883		Green ontogenesis	
ppa013960m	-4.53	0.004773435	Plant protein 1589 of unknown function	Green ontogenesis	
ppa002358m	-4.54	0.000739906	ABC-2 type transporter family protein	Green ontogenesis	
ppa023532m	-4.55	0.006264091	terpene synthase-like sequence-1,8-cineole	Green ontogenesis	Biotic Stress / Secondary metabolism involved into stress
ppa005373m	-4.55	0.003875091	Flavin-binding monooxygenase family protein	Green ontogenesis	
ppa025830m	-4.56	0.013717281	Ovate family protein	Green ontogenesis	
ppa014737m	-4.57	0.003406751	PAPA-1-like family protein / zinc finger (HIT type) family protein	Green ontogenesis	
ppa025098m	-4.57	0.015061871	Pectin lyase-like superfamily protein	Green ontogenesis	
ppa006814m	-4.58	0.001129143	Protein of unknown function, DUF642	Green ontogenesis	
ppa012775m	-4.59	0.002151548		Green ontogenesis	
ppa002187m	-4.60	0.001410772	heat shock protein 90.1	Green ontogenesis	
ppa008717m	-4.60	0.003296041		Green ontogenesis	
ppa019700m	-4.61	0.004190735	Aldolase-type TIM barrel family protein	Green ontogenesis	
ppa024745m	-4.61	0.010274587	Plant protein of unknown function (DUF827)	Green ontogenesis	
ppa024566m	-4.66	0.01241921	heat shock cognate protein 70-1	Green ontogenesis	
ppa018693m	-4.66	0.020128292	TRICHOME BIREFRINGENCE-LIKE 43	Green ontogenesis	
ppa012687m	-4.67	0.025781623	Ran BP2/NZF zinc finger-like superfamily protein	Green ontogenesis	
ppa011275m	-4.67	0.007817331	Cupredoxin superfamily protein	Green ontogenesis	
ppa003101m	-4.68	0.013138271	Protein of unknown function, DUF547	Green ontogenesis	
ppa008080m	-4.68	0.003352755	BURP domain-containing protein	Green ontogenesis	
ppa012290m	-4.69	0.000132814		Green ontogenesis	
ppa010954m	-4.71	0.000567585	ferredoxin-related	Green ontogenesis	
ppa010831m	-4.72	6.59E-05	Glutathione S-transferase family protein	Green ontogenesis	Biotic Stress / Glutathione-S-Transferase
ppa014426m	-4.73	0.003135797		Green ontogenesis	
ppa026726m	-4.74	0.001954667	D-isomer specific 2-hydroxyacid dehydrogenase family protein	Green ontogenesis	
ppa023468m	-4.74	0.00063489	wall associated kinase 4	Green ontogenesis	
ppa023784m	-4.76	0.04749626	Pectin lyase-like superfamily protein	Green ontogenesis	
ppa005388m	-4.76	0.002668538	alpha/beta-Hydrolases superfamily protein	Green ontogenesis	
ppa001798m	-4.77	0.01168129	Subtilase family protein	Green ontogenesis	
ppa025896m	-4.77	0.004124253	HSP20-like chaperones superfamily protein	Green ontogenesis	
ppb018294m	-4.77	0.001823737	O-acyltransferase (WSD1-like) family protein	Green ontogenesis	
ppa009407m	-4.78	0.01253152	NAD(P)-binding Rossmann-fold superfamily protein	Green ontogenesis	
ppa008985m	-4.79	0.022239233	ubiquitin-specific protease 12	Green ontogenesis	
ppa026062m	-4.80	0.00100309		Green ontogenesis	
ppb015877m	-4.81	0.028478037	SAUR-like auxin-responsive protein family	Green ontogenesis	
ppa021579m	-4.81	0.025279318	fatty acid amide hydrolase	Green ontogenesis	
ppa023338m	-4.82	0.042538558	Cupredoxin superfamily protein	Green ontogenesis	
ppa026139m	-4.82	0.000313173	Integrase-type DNA-binding superfamily protein	Green ontogenesis	
ppa020139m	-4.82	0.030527828	NAC 007	Green ontogenesis	
ppa012898m	-4.85	0.000132814	Polyketide cyclase/dehydrase and lipid transport superfamily prot	Green ontogenesis	
ppa013837m	-4.86	0.020153928	SAUR-like auxin-responsive protein family	Green ontogenesis	
ppa010342m	-4.87	0.000440126	indole-3-acetic acid 7	Green ontogenesis	
ppa015563m	-4.88	0.007765812	Ankyrin repeat family protein	Green ontogenesis	
ppa005849m	-4.88	0.000155769	Glycosyl hydrolase superfamily protein	Green ontogenesis	
ppa013195m	-4.90	0.003589689	ralf-like 34	Green ontogenesis	
ppa003380m	-4.91	0.013447016	terpene synthase 14	Green ontogenesis	Biotic Stress / Secondary metabolism involved into stress
ppa006897m	-4.93	3.76E-05	GDSL-like Lipase/Acylhydrolase superfamily protein	Green ontogenesis	
ppa021630m	-4.96	0.017160597	Protein kinase superfamily protein	Green ontogenesis	
ppa006581m	-4.96	0.008077451	Xanthine/uracil permease family protein	Green ontogenesis	
ppa020044m	-4.96	0.001390383		Green ontogenesis	
ppa008654m	-4.98	0.001480703	Ankyrin repeat family protein	Green ontogenesis	
ppa016923m	-4.98	0.02637794	myb domain protein 83	Green ontogenesis	
ppa011519m	-4.99	8.40E-05	Lactoylglutathione lyase / glyoxalase I family protein	Green ontogenesis	
ppa003520m	-5.01	0.000205527	cell elongation protein / DWARF1 / DIMINUTO (DIM)	Green ontogenesis	
ppa014722m	-5.02	0.046714383	Reticulon family protein	Green ontogenesis	
ppa004236m	-5.02	0.003327448	Plant protein of unknown function (DUF828)	Green ontogenesis	
ppa024465m	-5.09	0.034393557	COBRA-like extracellular glycosyl-phosphatidyl inositol-anchored	Green ontogenesis	
ppa006117m	-5.09	0.000439269	HXXXD-type acyl-transferase family protein	Green ontogenesis	
ppa025911m	-5.11	0.019160065	Ankyrin repeat family protein	Green ontogenesis	
ppa013805m	-5.11	0.004440069	Thioredoxin superfamily protein	Green ontogenesis	
ppa003524m	-5.12	0.005400761	acyl-CoA oxidase 1	Green ontogenesis	
ppa012112m	-5.12	0.009287367	Pollen Ole e 1 allergen and extensin family protein	Green ontogenesis	
ppa008634m	-5.13	0.023627168	Peroxidase family protein	Green ontogenesis	
ppa003308m	-5.16	0.033099124	laccase 17	Green ontogenesis	Biotic Stress / Secondary metabolism involved into stress
ppa023313m	-5.18	0.026440733	hydroxyproline-rich glycoprotein family protein	Green ontogenesis	
ppa016050m	-5.19	0.011983318	cytochrome P450, family 82, subfamily G, polypeptide 1	Green ontogenesis	
ppa026934m	-5.19	0.035614505	HXXXD-type acyl-transferase family protein	Green ontogenesis	Secondary Metabolism / Phenylpropanoids - Biotic Stress / Secondary metabolism involved into stress
ppa021144m	-5.22	0.000725739	alpha carbonic anhydrase 1	Green ontogenesis	
ppa010418m	-5.24	0.029578621	Pathogenesis-related thaumatin superfamily protein	Green ontogenesis	
ppa017323m	-5.25	0.00042149	Cupredoxin superfamily protein	Green ontogenesis	
ppa019383m	-5.25	0.014796189	Pollen Ole e 1 allergen and extensin family protein	Green ontogenesis	
ppa014086m	-5.26	0.001136153	Gibberellin-regulated family protein	Green ontogenesis	
ppa022906m	-5.27	0.033587179	Ankyrin-repeat containing protein	Green ontogenesis	
ppa015166m	-5.27	0.014417324	L-O-methylthreonine resistant 1	Green ontogenesis	
ppa020626m	-5.27	0.00506557	Ankyrin repeat family protein	Green ontogenesis	
ppa011533m	-5.28	4.50E-05	germin-like protein 7	Green ontogenesis	
ppa011761m	-5.29	0.003701681	GATA type zinc finger transcription factor family protein	Green ontogenesis	
ppa022385m	-5.30	0.022169508	Basic-leucine zipper (bZIP) transcription factor family protein	Green ontogenesis	
ppa003926m	-5.31	0.002548803	SKU5 similar 17	Green ontogenesis	
ppa016744m	-5.31	0.03214616	fatty acid desaturase 5	Green ontogenesis	
ppa003856m	-5.34	0.00641543	beta glucosidase 16	Green ontogenesis	
ppa019396m	-5.35	0.007733624	D-isomer specific 2-hydroxyacid dehydrogenase family protein	Green ontogenesis	
ppa017806m	-5.36	0.038868412	Cupredoxin superfamily protein	Green ontogenesis	
ppa006041m	-5.37	0.004245952	basic helix-loop-helix (bHLH) DNA-binding superfamily protein	Green ontogenesis	
ppa017145m	-5.38	0.005729235	Transducin/WD40 repeat-like superfamily protein	Green ontogenesis	
ppa012205m	-5.39	0.003982375		Green ontogenesis	
ppa007982m	-5.40	0.000922004	galactinol synthase 1	Green ontogenesis	
ppa020983m	-5.42	0.007765812		Green ontogenesis	
ppa023384m	-5.44	0.004208546	nitrate excretion transporter1	Green ontogenesis	
ppa021693m	-5.45	0.024110547	Ankyrin repeat family protein	Green ontogenesis	
ppa012022m	-5.46	0.000124561	glycosylphosphatidylinositol-anchored lipid protein transfer 1	Green ontogenesis	
ppa021457m	-5.47	0.001356283	GDSL-like Lipase/Acylhydrolase superfamily protein	Green ontogenesis	
ppa021281m	-5.48	0.006384079	Pollen Ole e 1 allergen and extensin family protein	Green ontogenesis	
ppb019226m	-5.49	0.01128516	Plant invertase/pectin methylesterase inhibitor superfamily protein	Green ontogenesis	
ppa002391m	-5.52	0.002414148		Green ontogenesis	
ppa013440m	-5.52	0.000658788	CAP (Cysteine-rich secretory proteins, Antigen 5, and Pathogene	Green ontogenesis	
ppa012538m	-5.53	1.66E-05	HSP20-like chaperones superfamily protein	Green ontogenesis	
ppa010275m	-5.55	0.016320213	HAD superfamily, subfamily IIIB acid phosphatase	Green ontogenesis	
ppa011396m	-5.56	0.005338751	alpha/beta-Hydrolases superfamily protein	Green ontogenesis	
ppa021240m	-5.56	0.001067516	xylem serine peptidase 1	Green ontogenesis	
ppa007777m	-5.59	0.016151732	galacturonosyltransferase-like 2	Green ontogenesis	
ppa018616m	-5.59	0.014850188	GDSL-like Lipase/Acylhydrolase superfamily protein	Green ontogenesis	
ppa025923m	-5.60	0.004828722	NAD(P)-binding Rossmann-fold superfamily protein	Green ontogenesis	
ppa023973m	-5.60	0.008454731	Ankyrin repeat family protein	Green ontogenesis	
ppa005391m	-5.61	0.008511435	Pectin lyase-like superfamily protein	Green ontogenesis	
ppa022307m	-5.62	0.000136527	HXXXD-type acyl-transferase family protein	Green ontogenesis	
ppa014138m	-5.63	1.36E-05		Green ontogenesis	
ppa021513m	-5.66	3.79E-05	Glycosyl hydrolase family 38 protein	Green ontogenesis	
ppa009061m	-5.67	0.018581285	fatty acid desaturase 5	Green ontogenesis	
ppa004172m	-5.67	0.0286543	glycosyl hydrolase 9B8	Green ontogenesis	
ppa023818m	-5.68	0.017713847		Green ontogenesis	

ppa026120m	-5.71	0.000399037	*Ankyrin repeat family protein*	Green ontogenesis	
ppa009792m	-5.72	0.000658788	*xyloglucan endotransglucosylase/hydrolase 15*	Green ontogenesis	
ppa023537m	-5.73	0.000601515	*alpha-L-fucosidase 1*	Green ontogenesis	
ppa025143m	-5.73	0.046887875	*Uncharacterised protein family (UPF0497)*	Green ontogenesis	
ppa007950m	-5.73	0.012504742	*S-adenosyl-L-methionine-dependent methyltransferases superfar*	Green ontogenesis	
ppa013286m	-5.76	0.000113887	*Bifunctional inhibitor/lipid-transfer protein/seed storage 2S albumi*	Green ontogenesis	
ppa012691m	-5.77	0.023823337	*HSP20-like chaperones superfamily protein*	Green ontogenesis	
ppa011467m	-5.78	5.91E-05	*germin 3*	Green ontogenesis	
ppa020598m	-5.79	0.017238289	*beta-hydroxyisobutyryl-CoA hydrolase 1*	Green ontogenesis	
ppa012828m	-5.80	0.000124561	*Uncharacterised protein family (UPF0497)*	Green ontogenesis	
ppa010152m	-5.80	0.001812249	*HAD superfamily, subfamily IIIB acid phosphatase*	Green ontogenesis	
ppa009350m	-5.83	0.004695941	*xyloglucan endotransglucosylase/hydrolase 9*	Green ontogenesis	
ppa018980m	-5.85	0.000155769	*RmlC-like cupins superfamily protein*	Green ontogenesis	
ppa009528m	-5.87	1.36E-05	*GDSL-like Lipase/Acylhydrolase superfamily protein*	Green ontogenesis	
ppa015151m	-5.89	7.05E-06	*HSP20-like chaperones superfamily protein*	Green ontogenesis	
ppa012348m	-5.94	0.000232773	*Protein of unknown function, DUF538*	Green ontogenesis	
ppa004126m	-5.96	4.96E-05	*Galactose oxidase/kelch repeat superfamily protein*	Green ontogenesis	
ppa006758m	-5.98	0.000113436	*3-ketoacyl-CoA synthase 6*	Green ontogenesis	
ppa012179m	-5.98	0.005498934	*RING/U-box superfamily protein*	Green ontogenesis	
ppa011954m	-5.99	0.000131152	*myb domain protein 4*	Green ontogenesis	
ppa025761m	-5.99	0.032982485	*proteasome inhibitor-related*	Green ontogenesis	
ppa019384m	-6.00	0.004038469	*germin-like protein 10*	Green ontogenesis	
ppa013833m	-6.06	0.016525088	*Bifunctional inhibitor/lipid-transfer protein/seed storage 2S albumi*	Green ontogenesis	
ppa019204m	-6.15	0.02070327	*Gibberellin-regulated family protein*	Green ontogenesis	
ppa020970m	-6.16	0.001377353	*Peroxidase superfamily protein*	Green ontogenesis	
ppa003422m	-6.18	0.000636336	*Glucose-methanol-choline (GMC) oxidoreductase family protein*	Green ontogenesis	
ppa020936m	-6.19	0.000720812	*BURP domain-containing protein*	Green ontogenesis	
ppa004037m	-6.22	0.000237564	*Nucleotide-diphospho-sugar transferases superfamily protein*	Green ontogenesis	
ppa021795m	-6.25	0.007665922		Green ontogenesis	
ppa012403m	-6.26	0.001030666	*alpha/beta-Hydrolases superfamily protein*	Green ontogenesis	
ppa015037m	-6.28	0.000315635	*Glycosyl hydrolase family protein*	Green ontogenesis	
ppa013981m	-6.28	0.028070442	*SAUR-like auxin-responsive protein family*	Green ontogenesis	
ppa012886m	-6.28	0.002126783	*Polyketide cyclase/dehydrase and lipid transport superfamily prot*	Green ontogenesis	
ppa019658m	-6.29	0.00490607	*Disease resistance-responsive (dirigent-like protein) family protei*	Green ontogenesis	Biotic Stress / PR-proteins
ppa001604m	-6.29	0.00137115	*Leucine-rich repeat receptor-like protein kinase family protein*	Green ontogenesis	
ppa015159m	-6.30	0.000391111	*Plant basic secretory protein (BSP) family protein*	Green ontogenesis	
ppa007610m	-6.33	0.003034945	*Protein of Unknown Function (DUF239)*	Green ontogenesis	
ppa022006m	-6.39	0.003735829	*HPT phosphotransmitter 4*	Green ontogenesis	
ppa012737m	-6.42	0.013583273	*Ran BP2/NZF zinc finger-like superfamily protein*	Green ontogenesis	
ppa021817m	-6.43	0.000174339	*Protein phosphatase 2A regulatory B subunit family protein*	Green ontogenesis	
ppa023135m	-6.45	0.014218291		Green ontogenesis	
ppa004968m	-6.45	0.000790717	*UDP-glucosyl transferase 85A2*	Green ontogenesis	
ppa007530m	-6.48	0.036128421	*Leucine-rich repeat (LRR) family protein*	Green ontogenesis	
ppa002261m	-6.49	0.024985665	*tetratricopeptide repeat (TPR)-containing protein*	Green ontogenesis	
ppa019268m	-6.50	0.002665667		Green ontogenesis	
ppa010387m	-6.51	0.010274424	*vacuolar iron transporter 1*	Green ontogenesis	
ppa010302m	-6.56	4.51E-05	*carbonic anhydrase 2*	Green ontogenesis	
ppa027182m	-6.62	0.002079695	*Basic helix-loop-helix (bHLH) DNA-binding family protein*	Green ontogenesis	
ppa016501m	-6.66	3.76E-05	*subtilase family protein*	Green ontogenesis	
ppa024769m	-6.70	0.000916598	*terpene synthase 03*	Green ontogenesis	Biotic Stress / Secondary metabolism involved into stress
ppa027143m	-6.71	0.003538287	*subtilase family protein*	Green ontogenesis	
ppa021568m	-6.73	0.002173511	*myb domain protein 85*	Green ontogenesis	
ppa010289m	-6.74	0.002151548	*Plant protein of unknown function (DUF828)*	Green ontogenesis	
ppa015093m	-6.85	0.002997443	*Cyclin D6;1*	Green ontogenesis	
ppa023961m	-6.89	0.000106669	*Subtilase family protein*	Green ontogenesis	
ppa011656m	-6.89	1.36E-05	*HSP20-like chaperones superfamily protein*	Green ontogenesis	
ppa009531m	-6.92	0.000249397	*GDSL-like Lipase/Acylhydrolase superfamily protein*	Green ontogenesis	
ppa010133m	-6.97	0.010919835	*NAD(P)-binding Rossmann-fold superfamily protein*	Green ontogenesis	Secondary Metabolism / Dihydroflavonols -Biotic Stress / Secondary metabolism involved into stress
ppa011450m	-7.01	0.007619441		Green ontogenesis	
ppa010762m	-7.04	3.72E-05	*heat shock protein 21*	Green ontogenesis	
ppa012890m	-7.14	0.000253764	*Polyketide cyclase/dehydrase and lipid transport superfamily prot*	Green ontogenesis	
ppa010517m	-7.14	0.001797852	*TRICHOME BIREFRINGENCE-LIKE 43*	Green ontogenesis	
ppa004844m	-7.21	0.005365245	*Exostosin family protein*	Green ontogenesis	
ppa021567m	-7.22	0.010502537	*GDSL-like Lipase/Acylhydrolase superfamily protein*	Green ontogenesis	
ppa017777m	-7.23	0.038900295	*matrix metalloproteinase*	Green ontogenesis	
ppa007768m	-7.24	0.000787198	*ARIA-interacting double AP2 domain protein*	Green ontogenesis	
ppa011385m	-7.26	0.000916429	*germin 3*	Green ontogenesis	
ppa020897m	-7.34	0.010285876		Green ontogenesis	
ppa020465m	-7.36	0.011254864	*homeobox 3*	Green ontogenesis	
ppa020220m	-7.37	0.000652427	*glutathione S-transferase TAU 19*	Green ontogenesis	Biotic Stress / Glutathione-S-Transferase
ppa004011m	-7.50	3.72E-05	*3-ketoacyl-CoA synthase 10*	Green ontogenesis	
ppa002172m	-7.52	0.001189758	*subtilase family protein*	Green ontogenesis	
ppa016157m	-7.58	0.029929929		Green ontogenesis	
ppa024832m	-7.64	0.002356244	*Ankyrin repeat family protein*	Green ontogenesis	
ppa025052m	-7.71	0.01200695	*terpene synthase 02*	Green ontogenesis	Biotic Stress / Secondary metabolism involved into stress
ppa011103m	-7.71	3.76E-05	*heat shock protein 21*	Green ontogenesis	
ppa004600m	-7.72	0.014842022	*Terpenoid cyclases/Protein prenyltransferases superfamily protei*	Green ontogenesis	Biotic Stress / Secondary metabolism involved into stress
ppa016030m	-7.77	0.022373273	*basic helix-loop-helix (bHLH) DNA-binding superfamily protein*	Green ontogenesis	
ppa010951m	-7.79	0.000916598	*WUSCHEL related homeobox 4*	Green ontogenesis	
ppa012312m	-7.79	4.51E-05	*Pollen Ole e 1 allergen and extensin family protein*	Green ontogenesis	
ppa017473m	-7.79	0.000190029	*Bifunctional inhibitor/lipid-transfer protein/seed storage 2S albumi*	Green ontogenesis	
ppa020430m	-7.86	7.05E-06	*subtilase family protein*	Green ontogenesis	
ppa007452m	-7.93	0.000832133	*Protein of unknown function, DUF642*	Green ontogenesis	
ppa024914m	-7.96	0.000433431		Green ontogenesis	
ppa007991m	-8.05	0.000316968	*GDSL-like Lipase/Acylhydrolase superfamily protein*	Green ontogenesis	
ppa013113m	-8.08	8.91E-05	*Nuclear transport factor 2 (NTF2) family protein*	Green ontogenesis	
ppa005699m	-8.10	0.000205856	*BURP domain-containing protein*	Green ontogenesis	
ppa012795m	-8.15	3.84E-05	*HSP20-like chaperones superfamily protein*	Green ontogenesis	
ppa019092m	-8.29	0.000134629	*GDSL-like Lipase/Acylhydrolase superfamily protein*	Green ontogenesis	
ppa017557m	-8.30	0.002920651	*GDSL-motif lipase/hydrolase 6*	Green ontogenesis	
ppa014598m	-8.32	0.000399037		Green ontogenesis	
ppa011192m	-8.36	0.002792365		Green ontogenesis	
ppa005535m	-8.39	1.77E-05	*BURP domain-containing protein*	Green ontogenesis	
ppa013554m	-8.42	0.000547379	*lipid transfer protein 3*	Green ontogenesis	
ppa026712m	-8.43	0.000131152	*beta glucosidase 31*	Green ontogenesis	
ppa007958m	-8.47	3.84E-05	*GDSL-like Lipase/Acylhydrolase superfamily protein*	Green ontogenesis	
ppa023110m	-8.49	0.000655073	*lipid transfer protein 1*	Green ontogenesis	
ppa007650m	-8.68	3.18E-05	*GDSL-like Lipase/Acylhydrolase superfamily protein*	Green ontogenesis	
ppa023335m	-8.71	0.001925237	*Cytochrome P450 superfamily protein*	Green ontogenesis	
ppa005351m	-8.91	3.36E-05	*Pollen Ole e 1 allergen and extensin family protein*	Green ontogenesis	
ppa022739m	-8.94	0.000163893	*squamosa promoter binding protein-like 5*	Green ontogenesis	
ppa013615m	-9.04	0.000185489		Green ontogenesis	
ppa002363m	-9.08	8.31E-06		Green ontogenesis	
ppa003816m	-9.18	0.000313173	*NRAMP metal ion transporter 6*	Green ontogenesis	
ppa015634m	-9.45	0.000260177	*Basic helix-loop-helix (bHLH) DNA-binding family protein*	Green ontogenesis	
ppa021107m	-9.52	0.000462685	*HSP20-like chaperones superfamily protein*	Green ontogenesis	
ppa023240m	-9.69	0.000182969	*MLP-like protein 28*	Green ontogenesis	
ppa003831m	-9.87	3.30E-05	*beta glucosidase 15*	Green ontogenesis	
ppa009439m	-10.35	0.000434471	*myb domain protein 7*	Green ontogenesis	
ppa012809m	-10.98	1.36E-05	*MLP-like protein 423*	Green ontogenesis	
ppa012742m	-12.07	4.82E-05	*MLP-like protein 28*	Green ontogenesis	
ppa019615m	14.09	0.002005598	*phospholipase A 2A*	Red ontogenesis	
ppa004103m	13.73	0.00170203	*FAD-binding Berberine family protein*	Red ontogenesis	
ppa015887m	12.49	0.022134394	*beta glucosidase 12*	Red ontogenesis	
ppa003126m	12.47	0.016072354	*Auxin-responsive GH3 family protein*	Red ontogenesis	
ppa025067m	12.31	0.003545031	*beta glucosidase 12*	Red ontogenesis	
ppa026111m	11.87	7.01E-05	*NB-ARC domain-containing disease resistance protein*	Red ontogenesis	
ppa025045m	11.74	0.008969334	*allene oxide synthase*	Red ontogenesis	
ppa024368m	11.66	0.000295299	*MLP-like protein 423*	Red ontogenesis	
ppa003578m	11.38	0.013076944	*Plant invertase/pectin methylesterase inhibitor superfamily*	Red ontogenesis	
ppa022337m	11.20	0.019688283	*Eukaryotic aspartyl protease family protein*	Red ontogenesis	
ppa026628m	11.13	0.001462382	*Cytochrome P450 superfamily protein*	Red ontogenesis	
ppa020368m	11.10	0.044059313	*beta glucosidase 12*	Red ontogenesis	
ppa015669m	10.89	0.043438637	*Late embryogenesis abundant (LEA) hydroxyproline-rich glycopro*	Red ontogenesis	
ppa013922m	10.85	0.000982113		Red ontogenesis	
ppa013725m	10.73	0.002474435		Red ontogenesis	
ppa025013m	10.53	0.003290482	*WRKY family transcription factor*	Red ontogenesis	
ppa018404m	10.42	0.005986703	*beta glucosidase 13*	Red ontogenesis	
ppa003587m	10.42	5.64E-05	*laccase 7*	Red ontogenesis	Biotic Stress / Secondary metabolism involved into stress
ppa021232m	10.26	7.58E-06	*elicitor-activated gene 3-1*	Red ontogenesis	Secondary Metabolism / Phenylpropanoids - Biotic Stress / Secondary metabolism involved into stress
ppa022164m	10.13	0.000166862	*cryptdin protein-related*	Red ontogenesis	
ppa018203m	10.13	0.003308672	*Phosphorylase superfamily protein*	Red ontogenesis	
ppa022216m	10.05	7.30E-05	*FAD-binding Berberine family protein*	Red ontogenesis	
ppa016459m	9.99	0.000860438	*WRKY DNA-binding protein 75*	Red ontogenesis	
ppa004774m	9.98	0.017276081	*ACC synthase 1*	Red ontogenesis	
ppa018829m	9.94	0.019939882	*elicitor-activated gene 3-2*	Red ontogenesis	Secondary Metabolism / Phenylpropanoids - Biotic Stress / Secondary metabolism involved into stress
ppa019068m	9.91	0.005108492	*cytochrome P450, family 71, subfamily B, polypeptide 36*	Red ontogenesis	
ppa017791m	9.78	0.007906245	*basic helix-loop-helix (bHLH) DNA-binding superfamily protein*	Red ontogenesis	
ppa004389m	9.75	0.000121226	*flavin-dependent monooxygenase 1*	Red ontogenesis	
ppa008671m	9.70	1.57E-06	*NAD(P)-binding Rossmann-fold superfamily protein*	Red ontogenesis	Secondary Metabolism / Dihydroflavonols -Biotic Stress / Secondary metabolism involved into stress
ppa014628m	9.69	0.00020087	*C-repeat-binding factor 4*	Red ontogenesis	
ppa010355m	9.66	8.54E-06	*Late embryogenesis abundant (LEA) hydroxyproline-rich glycopro*	Red ontogenesis	
ppa019880m	9.56	0.000495402	*laccase 1*	Red ontogenesis	Biotic Stress / Secondary metabolism involved into stress
ppa011310m	9.52	0.002257519		Red ontogenesis	
ppa015182m	9.47	3.74E-05	*glutathione S-transferase tau 7*	Red ontogenesis	Biotic Stress / Glutathione-S-Transferase
ppa016294m	9.44	0.003223492	*FAD-binding Berberine family protein*	Red ontogenesis	
ppa026827m	9.43	0.000370104	*GDSL lipase 1*	Red ontogenesis	
ppa024790m	9.40	0.001893425	*Concanavalin A-like lectin protein kinase family protein*	Red ontogenesis	

ppa015133m	9.40	7.32E-05	UDP-glucosyl transferase 73B3	Red ontogenesis	
ppb024519m	9.39	0.01178786	osmotin 34	Red ontogenesis	
ppb021268m	9.32	0.033136016		Red ontogenesis	
ppa015161m	9.31	0.018240019	beta glucosidase 12	Red ontogenesis	
ppa016302m	9.27	0.000326782	blue-copper-binding protein	Red ontogenesis	
ppa016522m	9.24	0.030954858	Lactate/malate dehydrogenase family protein	Red ontogenesis	
ppa011581m	9.22	3.92E-06		Red ontogenesis	
ppa026262m	9.21	7.05E-06	HXXXD-type acyl-transferase family protein	Red ontogenesis	Secondary Metabolism / Phenylpropanoids - Biotic Stress / Secondary metabolism involved into stress
ppa016345m	9.20	5.23E-07	Receptor-like protein kinase-related family protein	Red ontogenesis	
ppa018391m	9.14	7.80E-05	Serine protease inhibitor, potato inhibitor I-type family protein	Red ontogenesis	
ppa019643m	9.09	0.004733454		Red ontogenesis	
ppa023604m	9.08	0.004525075	Peroxidase superfamily protein	Red ontogenesis	
ppa015912m	9.03	0.000598437	Serine protease inhibitor, potato inhibitor I-type family protein	Red ontogenesis	
ppa018214m	9.03	0.000401902	Late embryogenesis abundant protein (LEA) family protein	Red ontogenesis	
ppa024049m	8.98	0.000316334	brassinosteroid-responsive RING-H2	Red ontogenesis	
ppa011524m	8.97	9.88E-06	blue-copper-binding protein	Red ontogenesis	
ppa010952m	8.95	0.002081081	homolog of carrot EP3-3 chitinase	Red ontogenesis	
ppa009089m	8.95	6.83E-07	early nodulin-like protein 14	Red ontogenesis	
ppa016401m	8.92	0.002037951	WRKY DNA-binding protein 9	Red ontogenesis	
ppa024500m	8.89	6.49E-05	Serine protease inhibitor, potato inhibitor I-type family protein	Red ontogenesis	
ppa011448m	8.86	8.54E-05	Kunitz family trypsin and protease inhibitor protein	Red ontogenesis	Biotic Stress / PR-proteins
ppa023146m	8.85	0.000688913	cation/hydrogen exchanger 15	Red ontogenesis	
ppa027123m	8.84	0.004074541	F-box family protein with a domain of unknown function (DUF295)	Red ontogenesis	
ppa004413m	8.81	0.004472239	Plant invertase/pectin methylesterase inhibitor superfamily	Red ontogenesis	
ppa026481m	8.80	0.038245999	ralf-like 4	Red ontogenesis	
ppa025882m	8.77	1.56E-05	2-oxoglutarate (2OG) and Fe(II)-dependent oxygenase superfami	Red ontogenesis	Secondary Metabolism / Dihydroflavonols / Flavonols-Isoflavonoid - Biotic Stress / Secondary metabolism involved into stress
ppa017919m	8.69	0.029681847	WRKY DNA-binding protein 72	Red ontogenesis	
ppa006071m	8.67	0.000751999	Eukaryotic aspartyl protease family protein	Red ontogenesis	
ppa021727m	8.65	0.000175287	UDP-Glycosyltransferase superfamily protein	Red ontogenesis	
ppa007061m	8.64	0.011769888	nodulin MtN21 /EamA-like transporter family protein	Red ontogenesis	
ppa016216m	8.62	0.003922642	ankyrin repeat family protein	Red ontogenesis	
ppa008986m	8.62	2.00E-06		Red ontogenesis	
ppa023987m	8.58	0.000172511	methyl esterase 3	Red ontogenesis	
ppa020048m	8.57	0.011130666	PHF5-like protein	Red ontogenesis	
ppa016466m	8.51	3.15E-06	cytochrome P450, family 87, subfamily A, polypeptide 2	Red ontogenesis	
ppa020605m	8.51	0.006691873	Integrase-type DNA-binding superfamily protein	Red ontogenesis	
ppa011773m	8.45	7.66E-05	Late embryogenesis abundant (LEA) hydroxyproline-rich glycopro	Red ontogenesis	
ppa021057m	8.44	0.005978564	Cysteine/Histidine-rich C1 domain family protein	Red ontogenesis	
ppa016650m	8.43	0.005329673		Red ontogenesis	
ppa019415m	8.37	0.008108107	2-oxoglutarate (2OG) and Fe(II)-dependent oxygenase superfami	Red ontogenesis	
ppa021018m	8.35	0.001813499	Cupredoxin superfamily protein	Red ontogenesis	
ppa018589m	8.34	7.67E-05	Serine protease inhibitor, potato inhibitor I-type family protein	Red ontogenesis	
ppa018374m	8.33	0.022807533	Wall-associated kinase family protein	Red ontogenesis	
ppa022042m	8.33	0.002607958	Protein of unknown function (DUF567)	Red ontogenesis	
ppa018664m	8.33	0.03048973	Peroxidase superfamily protein	Red ontogenesis	
ppa023867m	8.31	0.025889884	nudix hydrolase 1	Red ontogenesis	
ppa016458m	8.30	0.019112039	1-aminocyclopropane-1-carboxylic acid (acc) synthase 6	Red ontogenesis	
ppa000233m	8.30	1.39E-05	pleiotropic drug resistance 6	Red ontogenesis	
ppa019653m	8.29	0.000781707		Red ontogenesis	
ppa002608m	8.28	0.049320264	sulfate transporter 3;5	Red ontogenesis	
ppa009400m	8.24	7.69E-05	methyl esterase 3	Red ontogenesis	
ppa014321m	8.24	0.000180888	cryptdin protein-related	Red ontogenesis	
ppa026872m	8.22	0.003082833	phospholipase A 2A	Red ontogenesis	
ppa023495m	8.22	0.011586847	cytochrome P450, family 82, subfamily C, polypeptide 4	Red ontogenesis	
ppa015973m	8.21	0.009056255	myb domain protein 2	Red ontogenesis	
ppa012174m	8.18	8.54E-06	Ribulose bisphosphate carboxylase (small chain) family protein	Red ontogenesis	
ppa000232m	8.18	8.18E-06	pleiotropic drug resistance 11	Red ontogenesis	
ppa026021m	8.17	0.038029244	Cupredoxin superfamily protein	Red ontogenesis	
ppa020162m	8.10	0.019189501	RmlC-like cupins superfamily protein	Red ontogenesis	
ppa018675m	8.09	1.01E-05	2-oxoglutarate (2OG) and Fe(II)-dependent oxygenase superfami	Red ontogenesis	
ppa004592m	8.09	0.00849651	cytochrome P450, family 94, subfamily C, polypeptide 1	Red ontogenesis	
ppa014808m	8.07	0.02444037	Oxidoreductase, zinc-binding dehydrogenase family protein	Red ontogenesis	
ppa024321m	8.07	0.002541954	Exostosin family protein	Red ontogenesis	
ppa019252m	8.06	6.83E-07	Receptor-like protein kinase-related family protein	Red ontogenesis	
ppa007630m	8.04	0.022432132	D-mannose binding lectin protein with Apple-like carbohydrate-bin	Red ontogenesis	
ppa025296m	8.03	0.00734767	cytochrome P450, family 81, subfamily D, polypeptide 8	Red ontogenesis	
ppa026298m	7.96	0.000263919	Cysteine/Histidine-rich C1 domain family protein	Red ontogenesis	
ppa016301m	7.95	6.48E-06	Copper amine oxidase family protein	Red ontogenesis	
ppa004166m	7.95	0.000119836	bidirectional amino acid transporter 1	Red ontogenesis	
ppa020371m	7.95	0.039434107	Seed maturation protein	Red ontogenesis	
ppa019637m	7.94	7.26E-07	Disease resistance protein (TIR-NBS-LRR class) family	Red ontogenesis	Biotic Stress / PR-proteins
ppa004689m	7.92	0.008302338	Pyridoxal phosphate (PLP)-dependent transferases superfamily p	Red ontogenesis	
ppa014820m	7.92	0.04332079	ZCF37	Red ontogenesis	
ppa006110m	7.87	4.41E-07	beta glucosidase 17	Red ontogenesis	
ppa005408m	7.86	4.11E-06	Transmembrane amino acid transporter family protein	Red ontogenesis	
ppa015375m	7.84	0.0136557	FAR1-related sequence 5	Red ontogenesis	
ppa017143m	7.82	0.003880049	Wall-associated kinase family protein	Red ontogenesis	
ppa019803m	7.82	0.010380403	FAD-binding Berberine family protein	Red ontogenesis	
ppa011018m	7.77	0.03788928	MLP-like protein 423	Red ontogenesis	
ppa023302m	7.73	6.41E-05	PLC-like phosphodiesterases superfamily protein	Red ontogenesis	
ppa006455m	7.69	0.006600457	plant U-box 24	Red ontogenesis	
ppa021195m	7.66	6.94E-06	WRKY DNA-binding protein 61	Red ontogenesis	
ppa025645m	7.66	0.000707766		Red ontogenesis	
ppa023378m	7.66	0.000252201		Red ontogenesis	
ppa007615m	7.63	7.53E-05	elicitor-activated gene 3-1	Red ontogenesis	Secondary Metabolism / Phenylpropanoids - Biotic Stress / Secondary metabolism involved into stress
ppa014371m	7.63	0.000750321	Serine protease inhibitor, potato inhibitor I-type family protein	Red ontogenesis	
ppa004021m	7.60	0.000424938	FAD-binding Berberine family protein	Red ontogenesis	
ppb010518m	7.59	4.02E-05	MLP-like protein 423	Red ontogenesis	
ppa014515m	7.51	0.001865416		Red ontogenesis	
ppa007757m	7.51	0.000928136	Zinc-binding dehydrogenase family protein	Red ontogenesis	
ppa019709m	7.51	0.002064781	calmodulin-like 38	Red ontogenesis	
ppa020689m	7.49	0.000777908	Predicted AT-hook DNA-binding family protein	Red ontogenesis	
ppa010909m	7.48	0.000683156	C-repeat/DRE binding factor 2	Red ontogenesis	
ppa024352m	7.47	4.13E-05	glutamate receptor 2.7	Red ontogenesis	
ppb022421m	7.40	0.005860694	myb domain protein 15	Red ontogenesis	
ppa006816m	7.39	7.66E-05	NAD(P)-binding Rossmann-fold superfamily protein	Red ontogenesis	
ppa007811m	7.39	5.91E-05	2-oxoglutarate (2OG) and Fe(II)-dependent oxygenase superfami	Red ontogenesis	
ppa012646m	7.38	7.99E-06	MLP-like protein 423	Red ontogenesis	
ppa022513m	7.37	1.72E-05	beta glucosidase 13	Red ontogenesis	
ppa022911m	7.32	0.005168547		Red ontogenesis	
ppa024005m	7.30	0.003174319	RING/U-box superfamily protein	Red ontogenesis	
ppa015297m	7.27	0.001170576	Ribonuclease H-like superfamily protein	Red ontogenesis	
ppa023893m	7.27	3.48E-05	Disease resistance-responsive (dirigent-like protein) family protei	Red ontogenesis	Biotic Stress / PR-proteins
ppa007810m	7.25	0.000215956	Zinc-binding dehydrogenase family protein	Red ontogenesis	
ppa022223m	7.24	0.030014205	DNAJ-like 20	Red ontogenesis	
ppa014590m	7.23	0.006125381	PHYTOENE SYNTHASE	Red ontogenesis	Biotic Stress / Secondary metabolism involved into stress
ppa005837m	7.12	0.000427748	Cupredoxin superfamily protein	Red ontogenesis	Biotic Stress / Secondary metabolism involved into stress
ppa012649m	7.11	2.79E-06	MLP-like protein 423	Red ontogenesis	
ppa023749m	7.11	0.011470488	Late embryogenesis abundant (LEA) hydroxyproline-rich glycopro	Red ontogenesis	
ppb016539m	7.08	0.000257144	UDP-glycosyltransferase 73B4	Red ontogenesis	
ppa014675m	7.05	5.32E-05	heat shock transcription factor B3	Red ontogenesis	
ppa025302m	7.05	2.92E-05	MLP-like protein 423	Red ontogenesis	
ppa011934m	7.01	0.021431472		Red ontogenesis	
ppa012642m	7.00	4.60E-06	MLP-like protein 423	Red ontogenesis	
ppa001181m	7.00	7.47E-07	inosine-uridine preferring nucleoside hydrolase family protein	Red ontogenesis	
ppa014920m	7.00	2.04E-05	Chlorophyll A-B binding family protein	Red ontogenesis	
ppa020067m	6.99	0.00135218	beta glucosidase 13	Red ontogenesis	
ppa013703m	6.98	0.017412232		Red ontogenesis	
ppa014494m	6.98	0.009822796	Late embryogenesis abundant protein (LEA) family protein	Red ontogenesis	
ppb021417m	6.97	0.048550538	cytokinin oxidase 3	Red ontogenesis	
ppa019212m	6.97	0.000104719	Late embryogenesis abundant (LEA) hydroxyproline-rich glycopro	Red ontogenesis	
ppa017657m	6.97	0.004025877	Protein kinase superfamily protein	Red ontogenesis	
ppa024786m	6.92	0.015527865	NAD(P)-binding Rossmann-fold superfamily protein	Red ontogenesis	
ppa004245m	6.90	0.001845142	flavin-dependent monooxygenase 1	Red ontogenesis	
ppa017792m	6.87	0.009506709	Acyl-CoA N-acyltransferases (NAT) superfamily protein	Red ontogenesis	
ppa012991m	6.87	0.000208337	pathogenesis-related 4	Red ontogenesis	
ppa022609m	6.87	0.023403525		Red ontogenesis	
ppa017638m	6.86	0.000630002		Red ontogenesis	
ppa009992m	6.84	0.000405071	Receptor-like protein kinase-related family protein	Red ontogenesis	
ppa025969m	6.83	0.001902041	alpha/beta-Hydrolases superfamily protein	Red ontogenesis	
ppa015851m	6.83	0.02182295	Late embryogenesis abundant (LEA) hydroxyproline-rich glycopro	Red ontogenesis	
ppa009165m	6.78	5.22E-05	NAD(P)-binding Rossmann-fold superfamily protein	Red ontogenesis	
ppa013138m	6.77	0.034021275	Stigma-specific Stig1 family protein	Red ontogenesis	
ppa021446m	6.76	0.00581261	Plant invertase/pectin methylesterase inhibitor superfamily	Red ontogenesis	
ppa005094m	6.75	0.001866458	ACT domain repeat 1	Red ontogenesis	
ppa008191m	6.74	0.002239376	RmlC-like cupins superfamily protein	Red ontogenesis	
ppa004012m	6.73	2.36E-07	Seven transmembrane MLO family protein	Red ontogenesis	
ppa023100m	6.73	1.50E-05	cytochrome P450, family 72, subfamily A, polypeptide 9	Red ontogenesis	
ppa021008m	6.71	0.001374156	MuDR family transposase	Red ontogenesis	
ppa025263m	6.71	4.50E-05	NAC domain containing protein 42	Red ontogenesis	
ppa020068m	6.68	0.000346498		Red ontogenesis	
ppa019935m	6.67	0.026930184	F-box/RNI-like superfamily protein	Red ontogenesis	
ppa019283m	6.67	0.000215956	NB-ARC domain-containing disease resistance protein	Red ontogenesis	
ppa021949m	6.67	0.000137125	VQ motif-containing protein	Red ontogenesis	
ppa021447m	6.63	2.08E-05	O-methyltransferase 1	Red ontogenesis	
ppa008294m	6.63	5.53E-06	galactinol synthase 2	Red ontogenesis	
ppa026148m	6.62	0.001187145		Red ontogenesis	
ppa009530m	6.62	0.000214978	NAC-like, activated by AP3/PI	Red ontogenesis	

ppa015459m	6.61	0.035002203	DNA binding;ATP binding	Red ontogenesis	
ppa026171m	6.61	3.22E-06	glutathione S-transferase tau 7	Red ontogenesis	Biotic Stress / Glutathione-S-Transferase
ppa012632m	6.60	6.87E-07	MLP-like protein 423	Red ontogenesis	
ppa017192m	6.60	1.24E-05	glutathione S-transferase tau 7	Red ontogenesis	Biotic Stress / Glutathione-S-Transferase
ppa013228m	6.59	0.000131272		Red ontogenesis	
ppa010947m	6.59	0.015925211	homolog of carrot EP3-3 chitinase	Red ontogenesis	
ppa018265m	6.59	3.25E-05	RING/U-box superfamily protein	Red ontogenesis	
ppa017484m	6.57	0.01604078	beta glucosidase 13	Red ontogenesis	
ppa016149m	6.57	0.025403973	MAP kinase substrate 1	Red ontogenesis	
ppa010473m	6.55	5.32E-05	Pathogenesis-related thaumatin superfamily protein	Red ontogenesis	
ppa022066m	6.55	0.010971349	C2H2-type zinc finger family protein	Red ontogenesis	
ppa018310m	6.55	0.01890604		Red ontogenesis	
ppa021393m	6.53	0.005912531	Pathogenesis-related thaumatin superfamily protein	Red ontogenesis	
ppa011804m	6.51	0.007376776	heat shock transcription factor B2A	Red ontogenesis	
ppa026957m	6.51	5.41E-05	RING/U-box superfamily protein	Red ontogenesis	
ppa025047m	6.49	0.001670754	mitogen-activated protein kinase kinase kinase 15	Red ontogenesis	
ppa020915m	6.48	7.50E-05	Late embryogenesis abundant (LEA) hydroxyproline-rich glycopro	Red ontogenesis	
ppa026497m	6.46	0.000782452	galactinol synthase 2	Red ontogenesis	
ppa021223m	6.45	0.034347563	Protein kinase superfamily protein	Red ontogenesis	
ppa025128m	6.45	0.00173942	Pyridoxal phosphate (PLP)-dependent transferases superfamily p	Red ontogenesis	
ppa024281m	6.45	7.81E-06	glutathione S-transferase tau 7	Red ontogenesis	Biotic Stress / Glutathione-S-Transferase
ppa019339m	6.45	0.004491988	VQ motif-containing protein	Red ontogenesis	
ppa010471m	6.44	0.000268331	Pathogenesis-related thaumatin superfamily protein	Red ontogenesis	
ppa000839m	6.44	7.06E-06	glutamate receptor 2.8	Red ontogenesis	
ppa024359m	6.44	0.000413819	ARM repeat superfamily protein	Red ontogenesis	
ppb019918m	6.44	0.000107721	Chlorophyll A-B binding family protein	Red ontogenesis	
ppa020923m	6.44	0.006177395		Red ontogenesis	
ppa008738m	6.37	7.43E-06	RING/U-box superfamily protein	Red ontogenesis	
ppa022682m	6.37	0.024448879	purple acid phosphatase 10	Red ontogenesis	
ppa010479m	6.35	0.000119475	Pathogenesis-related thaumatin superfamily protein	Red ontogenesis	
ppa011142m	6.34	1.28E-05	glutathione S-transferase tau 7	Red ontogenesis	Biotic Stress / Glutathione-S-Transferase
ppa008732m	6.34	1.91E-05	Duplicated homeodomain-like superfamily protein	Red ontogenesis	
ppa017073m	6.31	0.000147297	RING/U-box superfamily protein	Red ontogenesis	
ppa008412m	6.31	0.000661398	carboxyesterase 17	Red ontogenesis	
ppa018777m	6.31	0.025549731	beta glucosidase 12	Red ontogenesis	
ppa024941m	6.31	0.000144589	homolog of carrot EP3-3 chitinase	Red ontogenesis	
ppa023881m	6.30	1.01E-05	phytosulfokine 4 precursor	Red ontogenesis	
ppa017982m	6.29	1.07E-06	expansin-like B1	Red ontogenesis	
ppa019661m	6.28	0.000134419	Late embryogenesis abundant (LEA) hydroxyproline-rich glycopro	Red ontogenesis	
ppa009047m	6.27	8.30E-05	inositol polyphosphate 5-phosphatase 11	Red ontogenesis	
ppa016014m	6.26	0.004561811	carotenoid cleavage dioxygenase 1	Red ontogenesis	
ppa009430m	6.26	0.046614545	Protein of unknown function (DUF506)	Red ontogenesis	
ppa015707m	6.24	0.033430727	Pollen Ole e 1 allergen and extensin family protein	Red ontogenesis	
ppa002973m	6.23	1.56E-05	Protein kinase family protein	Red ontogenesis	
ppa004390m	6.23	0.000103787	cytochrome P450, family 71, subfamily B, polypeptide 36	Red ontogenesis	
ppa003257m	6.23	0.000478153		Red ontogenesis	
ppa022109m	6.23	0.006560626	cysteine-rich RLK (RECEPTOR-like protein kinase) 10	Red ontogenesis	
ppa011242m	6.23	0.002300854	glutathione S-transferase TAU 19	Red ontogenesis	Biotic Stress / Glutathione-S-Transferase
ppa015017m	6.22	0.026478283	GRAM domain family protein	Red ontogenesis	
ppa017347m	6.21	0.005682696	Chitinase family protein	Red ontogenesis	
ppa010522m	6.18	0.000288413	Pathogenesis-related thaumatin superfamily protein	Red ontogenesis	
ppa014958m	6.16	7.05E-06	glutathione S-transferase tau 7	Red ontogenesis	Biotic Stress / Glutathione-S-Transferase
ppa023887m	6.16	0.000833877	S-adenosyl-L-methionine-dependent methyltransferases superfar	Red ontogenesis	
ppa003270m	6.15	0.000115259	cationic amino acid transporter 6	Red ontogenesis	
ppa026968m	6.14	0.010539187		Red ontogenesis	
ppa022706m	6.13	1.32E-06	plant natriuretic peptide A	Red ontogenesis	
ppa014027m	6.12	1.36E-05		Red ontogenesis	
ppa004664m	6.12	0.018774146	Cytochrome P450 superfamily protein	Red ontogenesis	
ppa023127m	6.12	6.87E-07	S-locus lectin protein kinase family protein	Red ontogenesis	
ppa017028m	6.11	1.26E-05		Red ontogenesis	
ppa016853m	6.11	5.18E-05	Major facilitator superfamily protein	Red ontogenesis	
ppa005448m	6.10	0.000424938	Curculin-like (mannose-binding) lectin family protein	Red ontogenesis	
ppa013442m	6.10	1.10E-05		Red ontogenesis	
ppa005427m	6.08	0.0105915	UDP-Glycosyltransferase superfamily protein	Red ontogenesis	
ppa014642m	6.06	0.004723008	Pectin lyase-like superfamily protein	Red ontogenesis	
ppa025538m	6.05	3.29E-06	Yippee family putative zinc-binding protein	Red ontogenesis	
ppa016206m	6.05	4.76E-05	UDP-Glycosyltransferase superfamily protein	Red ontogenesis	
ppa010199m	6.03	8.67E-05	Late embryogenesis abundant (LEA) hydroxyproline-rich glycopro	Red ontogenesis	
ppa022770m	6.03	0.002297309	F-box family protein with a domain of unknown function (DUF295)	Red ontogenesis	
ppa027018m	6.03	0.000151865	Late embryogenesis abundant (LEA) hydroxyproline-rich glycopro	Red ontogenesis	
ppb015086m	6.02	0.012016603	Protein kinase superfamily protein	Red ontogenesis	
ppa010943m	6.02	0.002215881	Protein of unknown function (DUF679)	Red ontogenesis	
ppa015248m	6.01	8.78E-05	ZCF37	Red ontogenesis	
ppa018468m	6.01	0.033750584	elicitor-activated gene 3-1	Red ontogenesis	Secondary Metabolism / Phenylpropanoids - Biotic Stress / Secondary metabolism involved into stress
ppa005711m	6.00	0.005641238	ARM repeat superfamily protein	Red ontogenesis	
ppa026320m	6.00	0.00958753		Red ontogenesis	
ppa010728m	6.00	3.29E-05		Red ontogenesis	
ppa025666m	5.98	3.30E-06	cytochrome P450, family 716, subfamily A, polypeptide 1	Red ontogenesis	
ppa009084m	5.98	0.006298232	NAD(P)-binding Rossmann-fold superfamily protein	Red ontogenesis	
ppa021625m	5.98	7.53E-05	2-oxoglutarate (2OG) and Fe(II)-dependent oxygenase superfami	Red ontogenesis	
ppa002734m	5.97	0.000220764	calmodulin-domain protein kinase cdpk isoform 2	Red ontogenesis	
ppa013329m	5.97	0.000316123		Red ontogenesis	
ppa022754m	5.96	0.000314402	syntaxin of plants 121	Red ontogenesis	
ppb010973m	5.95	0.000449047	2-oxoglutarate (2OG) and Fe(II)-dependent oxygenase superfami	Red ontogenesis	
ppa020043m	5.93	4.69E-05	GRAS family transcription factor	Red ontogenesis	
ppa018490m	5.92	0.000248287	Plant protein 1589 of unknown function	Red ontogenesis	
ppa016724m	5.91	0.0107225	Uncharacterised conserved protein UCP031279	Red ontogenesis	
ppa013276m	5.90	2.95E-05	sigma factor binding protein 1	Red ontogenesis	
ppa013533m	5.90	8.18E-06		Red ontogenesis	
ppa021111m	5.89	0.000244503	NAD(P)-binding Rossmann-fold superfamily protein	Red ontogenesis	
ppa018075m	5.89	1.21E-05	WRKY family transcription factor	Red ontogenesis	
ppa026001m	5.89	7.66E-05	2-oxoglutarate (2OG) and Fe(II)-dependent oxygenase superfami	Red ontogenesis	
ppa008603m	5.89	8.59E-07	NAD(P)-linked oxidoreductase superfamily protein	Red ontogenesis	
ppa026305m	5.88	0.002422251	SEC14-like 12	Red ontogenesis	
ppa021921m	5.88	0.003630576	myb domain protein 15	Red ontogenesis	
ppa018394m	5.87	0.010156394	Ribonuclease H-like superfamily protein	Red ontogenesis	
ppa011079m	5.85	5.50E-05	glutathione S-transferase TAU 8	Red ontogenesis	Biotic Stress / Glutathione-S-Transferase
ppa018669m	5.85	0.009699298	F-box and associated interaction domains-containing protein	Red ontogenesis	
ppb009345m	5.84	2.04E-06	glutathione S-transferase TAU 19	Red ontogenesis	Biotic Stress / Glutathione-S-Transferase
ppa022596m	5.83	0.013884415	RmlC-like cupins superfamily protein	Red ontogenesis	
ppa015472m	5.82	0.001739746	HXXXD-type acyl-transferase family protein	Red ontogenesis	Secondary Metabolism / Phenylpropanoids - Biotic Stress / Secondary metabolism involved into stress
ppa021244m	5.81	2.61E-06	Late embryogenesis abundant (LEA) hydroxyproline-rich glycopro	Red ontogenesis	
ppa024027m	5.80	0.001101288	WRKY DNA-binding protein 28	Red ontogenesis	
ppa017278m	5.80	0.000104947		Red ontogenesis	
ppa013513m	5.78	0.00901934		Red ontogenesis	
ppa014707m	5.78	0.040878971	PYR1-like 11	Red ontogenesis	
ppa008019m	5.77	0.024829448	plant intracellular ras group-related LRR 6	Red ontogenesis	
ppa026191m	5.76	0.002782598	F-box family protein	Red ontogenesis	
ppa014521m	5.75	5.75E-05		Red ontogenesis	
ppa016800m	5.75	1.74E-05	cytochrome P450, family 72, subfamily A, polypeptide 9	Red ontogenesis	
ppa027021m	5.74	0.029304311	drought-induced 21	Red ontogenesis	
ppa005659m	5.74	0.010708611	ARM repeat superfamily protein	Red ontogenesis	
ppa017079m	5.73	0.002049073		Red ontogenesis	
ppa018454m	5.73	3.15E-06	UDP-glucosyl transferase 73C1	Red ontogenesis	
ppa002979m	5.70	2.92E-05	wall associated kinase-like 4	Red ontogenesis	
ppa027156m	5.69	0.025197531	TCP family transcription factor	Red ontogenesis	
ppa010586m	5.69	0.000346651	GRAM domain family protein	Red ontogenesis	
ppa024604m	5.68	0.001347503		Red ontogenesis	
ppa011132m	5.67	0.000171336	Protein of unknown function (DUF567)	Red ontogenesis	
ppa013574m	5.66	0.00080588		Red ontogenesis	
ppa025826m	5.66	0.004304275	RING/U-box superfamily protein	Red ontogenesis	
ppa024830m	5.66	0.001668599	Integrin-linked protein kinase family	Red ontogenesis	
ppa021485m	5.65	0.000556439	Late embryogenesis abundant (LEA) hydroxyproline-rich glycopro	Red ontogenesis	
ppa004332m	5.65	0.002173573	cytochrome P450, family 71, subfamily A, polypeptide 25	Red ontogenesis	
ppa006887m	5.64	3.33E-05	methyl esterase 13	Red ontogenesis	
ppa013192m	5.64	0.007134903		Red ontogenesis	
ppa005277m	5.63	2.13E-05	UDP-glucosyl transferase 71B6	Red ontogenesis	
ppa014623m	5.63	0.015929549	dicarboxylate carrier 2	Red ontogenesis	
ppa012723m	5.62	0.005537489	hemoglobin 1	Red ontogenesis	
ppa015483m	5.60	0.000358049		Red ontogenesis	
ppa004319m	5.60	7.21E-05	cytochrome P450, family 72, subfamily A, polypeptide 9	Red ontogenesis	
ppa017234m	5.60	0.006266962	Putative membrane lipoprotein	Red ontogenesis	
ppa012339m	5.60	0.02724031	Adenine nucleotide alpha hydrolases-like superfamily protein	Red ontogenesis	
ppa012242m	5.59	0.00389534	C2H2-type zinc finger family protein	Red ontogenesis	
ppa000941m	5.58	2.62E-05	Protein kinase family protein with leucine-rich repeat domain	Red ontogenesis	
ppb012927m	5.58	1.06E-05	MLP-like protein 423	Red ontogenesis	
ppa019526m	5.58	0.000894473	plant U-box 24	Red ontogenesis	
ppa010729m	5.57	8.55E-05	pathogenesis-related family protein	Red ontogenesis	
ppa016218m	5.57	0.004843247	glutathione S-transferase tau 7	Red ontogenesis	Biotic Stress / Glutathione-S-Transferase
ppa014940m	5.57	0.000157767	2-oxoglutarate (2OG) and Fe(II)-dependent oxygenase superfami	Red ontogenesis	
ppa014833m	5.57	0.017818476	pectin methylesterase 44	Red ontogenesis	
ppa005290m	5.56	2.58E-05	UDP-glucosyl transferase 71B6	Red ontogenesis	
ppa009661m	5.54	6.37E-05	homolog of carrot EP3-3 chitinase	Red ontogenesis	
ppa019739m	5.53	0.001674763	VQ motif-containing protein	Red ontogenesis	
ppa003065m	5.52	5.83E-05	Auxin-responsive GH3 family protein	Red ontogenesis	
ppa022758m	5.52	0.000579892	WRKY family transcription factor	Red ontogenesis	
ppa023469m	5.51	5.23E-07	alpha/beta-Hydrolases superfamily protein	Red ontogenesis	
ppa003874m	5.49	0.000183477	fucosyltransferase 1	Red ontogenesis	

ppa010208m	5.47	0.000377472	*Nodulin MtN3 family protein*	Red ontogenesis	
ppa010287m	5.46	0.000193084	*Aluminium induced protein with YGL and LRDR motifs*	Red ontogenesis	
ppa008801m	5.46	6.36E-05	*NAC domain containing protein 1*	Red ontogenesis	
ppa018710m	5.46	0.001502683	*related to AP2 11*	Red ontogenesis	
ppb012406m	5.46	0.001813499	*S-domain-1 29*	Red ontogenesis	
ppa1027169m	5.45	0.001994817	*plant U-box 23*	Red ontogenesis	
ppa017120m	5.45	0.000640294	*2-oxoglutarate (2OG) and Fe(II)-dependent oxygenase superfami*	Red ontogenesis	
ppa025318m	5.44	0.009561327		Red ontogenesis	
ppa008808m	5.44	0.006493946	*Peroxidase superfamily protein*	Red ontogenesis	
ppa018960m	5.44	1.41E-05	*NAD(P)-linked oxidoreductase superfamily protein*	Red ontogenesis	
ppa022502m	5.42	0.023138647		Red ontogenesis	
ppa024382m	5.41	0.011164387	*cytochrome P450, family 87, subfamily A, polypeptide 2*	Red ontogenesis	
ppa004341m	5.40	1.54E-05	*cytochrome P450, family 714, subfamily A, polypeptide 1*	Red ontogenesis	
ppa015392m	5.40	1.73E-05	*Integrase-type DNA-binding superfamily protein*	Red ontogenesis	
ppa022043m	5.39	5.57E-06	*NAC domain containing protein 42*	Red ontogenesis	
ppb009629m	5.39	0.003538844		Red ontogenesis	
ppa004288m	5.38	0.025355863	*beta glucosidase 15*	Red ontogenesis	
ppb023048m	5.38	0.00273694		Red ontogenesis	
ppa020969m	5.37	0.00205872	*Protein of unknown function, DUF599*	Red ontogenesis	
ppa018499m	5.36	0.01939579	*Late embryogenesis abundant (LEA) hydroxyproline-rich glycopro*	Red ontogenesis	
ppa022413m	5.36	7.30E-05	*S-locus lectin protein kinase family protein*	Red ontogenesis	
ppa012907m	5.36	5.78E-05	*calmodulin-like 11*	Red ontogenesis	
ppa006365m	5.35	0.000179979	*RING/U-box superfamily protein*	Red ontogenesis	
ppa011386m	5.35	0.000906281	*oxidative stress 3*	Red ontogenesis	
ppa025431m	5.35	0.000156024	*calmodulin-like 38*	Red ontogenesis	
ppa020888m	5.34	0.008003235	*S-locus protein kinase, putative*	Red ontogenesis	
ppa024029m	5.34	0.012388828	*cytochrome P450, family 715, subfamily A, polypeptide 1*	Red ontogenesis	
ppa004465m	5.32	9.41E-06	*Major facilitator superfamily protein*	Red ontogenesis	
ppa022651m	5.32	0.03500983	*Phosphoglycerate mutase family protein*	Red ontogenesis	
ppa020943m	5.32	0.003444898	*Abscisic acid-responsive (TB2/DP1, HVA22) family protein*	Red ontogenesis	
ppa014464m	5.31	1.46E-05	*Late embryogenesis abundant protein (LEA) family protein*	Red ontogenesis	
ppa005059m	5.31	3.62E-05	*cytochrome P450, family 707, subfamily A, polypeptide 1*	Red ontogenesis	
ppa011672m	5.30	0.000304792		Red ontogenesis	
ppa025011m	5.30	0.005640154	*Late embryogenesis abundant (LEA) hydroxyproline-rich glycopro*	Red ontogenesis	
ppa017927m	5.29	7.83E-06	*glutamate receptor 2.7*	Red ontogenesis	
ppa021960m	5.29	4.67E-05	*Late embryogenesis abundant (LEA) hydroxyproline-rich glycopro*	Red ontogenesis	
ppb022773m	5.29	0.016739899	*RING/U-box superfamily protein*	Red ontogenesis	
ppa018800m	5.28	0.002277091	*Concanavalin A-like lectin protein kinase family protein*	Red ontogenesis	
ppa024342m	5.28	5.57E-06	*S-domain-1 29*	Red ontogenesis	
ppa015069m	5.27	0.001302242	*purple acid phosphatase 10*	Red ontogenesis	
ppa023229m	5.27	0.000128154	*Major facilitator superfamily protein*	Red ontogenesis	
ppa012149m	5.26	0.013774179	*RING/U-box superfamily protein*	Red ontogenesis	
ppb011907m	5.25	0.000784604		Red ontogenesis	
ppa017452m	5.24	0.0012313	*cinnamyl alcohol dehydrogenase 9*	Red ontogenesis	Secondary Metabolism / Phenylpropanoids - Biotic Stress / Secondary metabolism involved into stress
ppa011008m	5.24	2.36E-07	*DNAJ heat shock N-terminal domain-containing protein*	Red ontogenesis	
ppa011598m	5.24	1.94E-05	*nitrate transmembrane transporters*	Red ontogenesis	
ppa021769m	5.24	0.033520541	*RING/U-box superfamily protein*	Red ontogenesis	
ppa019638m	5.24	0.027921445	*RING/U-box superfamily protein*	Red ontogenesis	
ppa018862m	5.24	0.000590445	*Late embryogenesis abundant (LEA) hydroxyproline-rich glycopro*	Red ontogenesis	
ppa019036m	5.23	0.0037165	*C2H2 and C2HC zinc fingers superfamily protein*	Red ontogenesis	
ppa011762m	5.23	9.81E-06	*C2H2-type zinc finger family protein*	Red ontogenesis	
ppa023775m	5.23	0.005614872	*indeterminate(ID)-domain 2*	Red ontogenesis	
ppa023786m	5.23	0.000777908	*cytochrome P450, family 81, subfamily D, polypeptide 8*	Red ontogenesis	
ppa018344m	5.23	0.000494345	*MLP-like protein 423*	Red ontogenesis	
ppa011835m	5.22	0.000143468	*Late embryogenesis abundant (LEA) hydroxyproline-rich glycopro*	Red ontogenesis	
ppa024264m	5.22	0.00369261	*Disease resistance-responsive (dirigent-like protein) family protei*	Red ontogenesis	Biotic Stress / PR-proteins
ppa026831m	5.21	3.95E-05	*WRKY DNA-binding protein 28*	Red ontogenesis	
ppa021010m	5.18	3.72E-05		Red ontogenesis	
ppa025939m	5.16	0.02210924	*MuDR family transposase*	Red ontogenesis	
ppa005662m	5.16	4.16E-05	*lysine histidine transporter 1*	Red ontogenesis	
ppa012629m	5.14	6.94E-06	*MLP-like protein 423*	Red ontogenesis	
ppa011701m	5.13	5.39E-05	*Calcium-binding EF-hand family protein*	Red ontogenesis	
ppa018079m	5.13	0.000111957	*Late embryogenesis abundant (LEA) hydroxyproline-rich glycopro*	Red ontogenesis	
ppa016238m	5.12	0.048967362	*receptor kinase 3*	Red ontogenesis	
ppa017905m	5.11	0.009022293		Red ontogenesis	
ppb017968m	5.09	3.85E-05	*cytochrome P450, family 709, subfamily B, polypeptide 1*	Red ontogenesis	
ppa010303m	5.08	0.000215956	*indole-3-acetic acid 7*	Red ontogenesis	
ppa025895m	5.06	3.05E-05	*Wall-associated kinase family protein*	Red ontogenesis	
ppa014784m	5.06	0.012984093		Red ontogenesis	
ppa004496m	5.06	7.83E-06	*cytochrome P450, family 714, subfamily A, polypeptide 1*	Red ontogenesis	
ppa024619m	5.04	2.70E-07	*methyl esterase 10*	Red ontogenesis	
ppa025486m	5.02	0.00227757		Red ontogenesis	
ppa009711m	5.02	0.001518472	*atypical CYS HIS rich thioredoxin 5*	Red ontogenesis	
ppa013728m	5.01	0.013442103	*flowering promoting factor 1*	Red ontogenesis	
ppa008358m	5.01	8.27E-05	*Oxidoreductase, zinc-binding dehydrogenase family protein*	Red ontogenesis	
ppa022672m	5.00	7.79E-05	*2-oxoglutarate (2OG) and Fe(II)-dependent oxygenase superfami*	Red ontogenesis	
ppa007162m	4.99	0.002127723	*galacturonosyltransferase-like 10*	Red ontogenesis	
ppb013202m	4.99	0.024599142		Red ontogenesis	
ppa021318m	4.98	0.002083609	*cytochrome P450, family 81, subfamily D, polypeptide 2*	Red ontogenesis	
ppa025846m	4.98	0.013582234	*F-box family protein with a domain of unknown function (DUF295)*	Red ontogenesis	
ppa021744m	4.97	0.000146611	*ATPase E1-E2 type family protein / haloacid dehalogenase-like h*	Red ontogenesis	
ppa019189m	4.96	0.000544617		Red ontogenesis	
ppa004009m	4.96	0.00160097	*WRKY DNA-binding protein 33*	Red ontogenesis	
ppa020333m	4.94	0.002485766	*Uncharacterised conserved protein UCP015417, vWA*	Red ontogenesis	
ppa022971m	4.94	0.000282948	*wall associated kinase-like 2*	Red ontogenesis	
ppa024217m	4.94	0.000144428	*cinnamyl-alcohol dehydrogenase*	Red ontogenesis	Secondary Metabolism / Phenylpropanoids - Biotic Stress / Secondary metabolism involved into stress
ppb013610m	4.93	0.006648758		Red ontogenesis	
ppa007741m	4.92	8.37E-07	*nudix hydrolase homolog 2*	Red ontogenesis	
ppa026236m	4.91	0.000516187		Red ontogenesis	
ppa024928m	4.91	0.038572061	*Pyridoxal phosphate (PLP)-dependent transferases superfamily p*	Red ontogenesis	
ppa015518m	4.90	0.000129738	*2-oxoglutarate (2OG) and Fe(II)-dependent oxygenase superfami*	Red ontogenesis	
ppa004709m	4.89	0.046115964	*glyoxal oxidase-related protein*	Red ontogenesis	
ppa026012m	4.88	0.032940453	*Pyridoxal phosphate (PLP)-dependent transferases superfamily p*	Red ontogenesis	
ppa003417m	4.88	0.008756949	*inositol transporter 4*	Red ontogenesis	
ppa014462m	4.87	0.002813657		Red ontogenesis	
ppa015635m	4.87	0.000364052	*methyl esterase 3*	Red ontogenesis	
ppa025206m	4.87	0.047491399		Red ontogenesis	
ppa014409m	4.87	0.022552061		Red ontogenesis	
ppa022668m	4.86	0.004756471	*phloem protein 2-B15*	Red ontogenesis	
ppa003437m	4.86	0.001248877	*Seven transmembrane MLO family protein*	Red ontogenesis	
ppa025888m	4.85	0.001047964	*LOB domain-containing protein 11*	Red ontogenesis	
ppa025103m	4.85	0.010488299	*NAC domain containing protein 90*	Red ontogenesis	
ppa012761m	4.85	0.00081922	*VQ motif-containing protein*	Red ontogenesis	
ppa016693m	4.85	0.002069814	*mitogen-activated protein kinase kinase kinase 14*	Red ontogenesis	
ppa003209m	4.85	1.06E-05	*peptide transporter 3*	Red ontogenesis	
ppa020825m	4.84	0.014137762	*xyloglucan endotransglucosylase/hydrolase 16*	Red ontogenesis	
ppa002513m	4.84	2.92E-05	*S-adenosyl-L-methionine-dependent methyltransferases superfar*	Red ontogenesis	
ppa013821m	4.81	0.000226042		Red ontogenesis	
ppa005447m	4.80	0.000758258	*UDP-Glycosyltransferase superfamily protein*	Red ontogenesis	
ppa023986m	4.79	0.000132275	*S-adenosyl-L-methionine-dependent methyltransferases superfar*	Red ontogenesis	
ppa014697m	4.79	0.005379942	*Bifunctional inhibitor/lipid-transfer protein/seed storage 2S albumi*	Red ontogenesis	
ppa007059m	4.77	5.32E-05	*Regulator of Vps4 activity in the MVB pathway protein*	Red ontogenesis	
ppa008646m	4.76	0.005416017	*basic chitinase*	Red ontogenesis	
ppa018984m	4.76	0.000275053	*NAD(P)-binding Rossmann-fold superfamily protein*	Red ontogenesis	
ppa011528m	4.74	0.001114309	*LOB domain-containing protein 1*	Red ontogenesis	
ppa022653m	4.72	0.000346498	*GRAS family transcription factor*	Red ontogenesis	
ppa006909m	4.72	1.96E-05	*WRKY DNA-binding protein 48*	Red ontogenesis	
ppa009330m	4.72	0.000248426	*NAD(P)-binding Rossmann-fold superfamily protein*	Red ontogenesis	
ppa008529m	4.71	8.15E-07	*NAD(P)-linked oxidoreductase superfamily protein*	Red ontogenesis	
ppa022159m	4.71	0.021573181	*glyoxalase I homolog*	Red ontogenesis	
ppa008350m	4.69	0.00089712	*alternative oxidase 2*	Red ontogenesis	
ppa023456m	4.68	0.036406595		Red ontogenesis	
ppa000234m	4.68	0.023342875	*pleiotropic drug resistance 12*	Red ontogenesis	
ppa026843m	4.68	1.72E-05	*NAC (No Apical Meristem) domain transcriptional regulator super*	Red ontogenesis	
ppa025205m	4.68	0.001253085		Red ontogenesis	
ppa014141m	4.67	6.24E-05	*Wound-responsive family protein*	Red ontogenesis	
ppa013181m	4.67	5.97E-05		Red ontogenesis	
ppa014020m	4.66	7.57E-05		Red ontogenesis	
ppa026853m	4.65	3.93E-05	*glutamate receptor 2.8*	Red ontogenesis	
ppa010679m	4.64	0.000804066	*K-box region and MADS-box transcription factor family protein*	Red ontogenesis	
ppa009328m	4.64	0.000225047	*chitinase A*	Red ontogenesis	
ppa001730m	4.64	7.59E-05	*seed imbibition 2*	Red ontogenesis	
ppa023989m	4.63	0.013545269	*LOB domain-containing protein 1*	Red ontogenesis	
ppa011637m	4.63	0.000316123	*dehydrin xero 1*	Red ontogenesis	
ppa002497m	4.63	4.00E-05	*S-domain-1 29*	Red ontogenesis	
ppb023577m	4.63	0.000654263	*cytochrome P450, family 709, subfamily B, polypeptide 2*	Red ontogenesis	
ppa000309m	4.62	0.00132617	*Transducin/WD40 repeat-like superfamily protein*	Red ontogenesis	
ppa003547m	4.62	0.000355551	*Eukaryotic aspartyl protease family protein*	Red ontogenesis	
ppa026874m	4.62	1.40E-05	*cytochrome P450, family 72, subfamily A, polypeptide 7*	Red ontogenesis	
ppb021827m	4.61	0.030208582	*cinnamyl alcohol dehydrogenase 6*	Red ontogenesis	Secondary Metabolism / Phenylpropanoids - Biotic Stress / Secondary metabolism involved into stress
ppa015305m	4.60	0.002102054	*HVA22-like protein G*	Red ontogenesis	
ppa008799m	4.60	0.00026197	*2-oxoglutarate (2OG) and Fe(II)-dependent oxygenase superfami*	Red ontogenesis	
ppa025607m	4.59	4.02E-05	*Wall-associated kinase family protein*	Red ontogenesis	
ppa004655m	4.59	3.00E-06	*Glycosyltransferase family 61 protein*	Red ontogenesis	
ppa024584m	4.59	0.031472863		Red ontogenesis	
ppa012678m	4.58	1.11E-05	*MLP-like protein 423*	Red ontogenesis	
ppa024173m	4.58	0.034739127	*ethylene response factor 1*	Red ontogenesis	
ppa000239m	4.58	0.000285326	*pleiotropic drug resistance 6*	Red ontogenesis	

ppa016264m	4.56	5.57E-06	Transmembrane amino acid transporter family protein	Red ontogenesis	
ppa015115m	4.55	0.001084495	blue-copper-binding protein	Red ontogenesis	
ppa011097m	4.55	0.004611053	related to AP2 6l	Red ontogenesis	
ppa1027121m	4.55	7.66E-05	glutamate receptor 2.7	Red ontogenesis	
ppa012062m	4.54	0.000281518	Disease resistance-responsive (dirigent-like protein) family protei	Red ontogenesis	Biotic Stress / PR-proteins
ppa003107m	4.54	8.54E-05	amino acid transporter 1	Red ontogenesis	
ppa017569m	4.53	0.000682073	ankyrin repeat family protein	Red ontogenesis	
ppa001861m	4.53	2.86E-06	cellulose synthase like G2	Red ontogenesis	
ppa023491m	4.53	0.047539838	Uncharacterised protein family (UPF0497)	Red ontogenesis	
ppa009290m	4.52	8.34E-07	NAD(P)-binding Rossmann-fold superfamily protein	Red ontogenesis	
ppa021913m	4.52	0.015675541		Red ontogenesis	
ppa018340m	4.51	0.000105576	Glycosyl hydrolase family protein with chitinase insertion domain	Red ontogenesis	
ppa011794m	4.51	0.005340099	Plant invertase/pectin methylesterase inhibitor superfamily protei	Red ontogenesis	
ppb010273m	4.50	3.09E-05		Red ontogenesis	
ppa007835m	4.49	0.028909043	Protein kinase superfamily protein	Red ontogenesis	
ppa026707m	4.49	0.000144806	cytochrome P450, family 714, subfamily A, polypeptide 2	Red ontogenesis	
ppa014104m	4.49	0.00429198	B12D protein	Red ontogenesis	
ppa007685m	4.48	0.001601869	xyloglucan endotransglucosylase/hydrolase 30	Red ontogenesis	
ppa019965m	4.48	1.25E-06	cytochrome P450, family 81, subfamily D, polypeptide 8	Red ontogenesis	
ppa016347m	4.47	0.000208434	Thioredoxin superfamily protein	Red ontogenesis	
ppa016280m	4.47	0.007184576	carboxyesterase 17	Red ontogenesis	
ppa012261m	4.47	8.04E-05	Late embryogenesis abundant (LEA) hydroxyproline-rich glycopro	Red ontogenesis	
ppa023632m	4.47	0.000260349		Red ontogenesis	
ppa021901m	4.47	7.26E-07	cytochrome P450, family 72, subfamily A, polypeptide 9	Red ontogenesis	
ppa010337m	4.46	0.001813499	NAC domain containing protein 90	Red ontogenesis	
ppa012651m	4.46	1.47E-06	MLP-like protein 423	Red ontogenesis	
ppa023672m	4.45	0.001826102	plant U-box 23	Red ontogenesis	
ppa023006m	4.45	0.036764146	Calcium-binding EF-hand family protein	Red ontogenesis	
ppa019724m	4.44	0.001316968	Concanavalin A-like lectin protein kinase family protein	Red ontogenesis	
ppa016268m	4.44	5.57E-06	osmotin 34	Red ontogenesis	
ppa017934m	4.44	0.011014659	Exostosin family protein	Red ontogenesis	
ppa016936m	4.44	0.025663061	Protein phosphatase 2C family protein	Red ontogenesis	
ppa023494m	4.44	0.000200269	F-box family protein	Red ontogenesis	
ppa026269m	4.43	4.08E-05	folate transporter 1	Red ontogenesis	
ppa019211m	4.42	0.005511767		Red ontogenesis	
ppb011659m	4.41	0.000267989		Red ontogenesis	
ppa016235m	4.41	0.028881268	Disease resistance protein (TIR-NBS-LRR class) family	Red ontogenesis	Biotic Stress / PR-proteins
ppa020091m	4.41	0.000225047	magnesium transporter 4	Red ontogenesis	
ppb011093m	4.41	0.005011573	arabinogalactan protein 1	Red ontogenesis	
ppa003470m	4.41	4.63E-05	Glycosyl hydrolases family 32 protein	Red ontogenesis	
ppa019104m	4.40	8.34E-07	methyl esterase 3	Red ontogenesis	
ppa011666m	4.40	2.69E-05	Late embryogenesis abundant (LEA) hydroxyproline-rich glycopro	Red ontogenesis	
ppa004825m	4.40	1.88E-06	UDP-Glycosyltransferase superfamily protein	Red ontogenesis	
ppa025628m	4.40	0.020837368	Expressed protein	Red ontogenesis	
ppa008521m	4.40	0.001096167	galactinol synthase 2	Red ontogenesis	
ppa020792m	4.39	0.003833599	ARM repeat superfamily protein	Red ontogenesis	
ppa014695m	4.39	0.012369398	Bifunctional inhibitor/lipid-transfer protein/seed storage 2S albumi	Red ontogenesis	
ppa012765m	4.38	0.001269303	Chaperone DnaJ-domain superfamily protein	Red ontogenesis	
ppa024816m	4.38	0.022667288	AGAMOUS-like 29	Red ontogenesis	
ppa022752m	4.38	2.30E-05	Cytochrome P450 superfamily protein	Red ontogenesis	
ppa018724m	4.37	6.83E-07	Cytochrome P450 superfamily protein	Red ontogenesis	
ppa012929m	4.37	8.30E-05	calmodulin-like 11	Red ontogenesis	
ppa015588m	4.37	1.85E-06	Cytochrome P450 superfamily protein	Red ontogenesis	
ppa020590m	4.37	0.005676931	BON association protein 2	Red ontogenesis	
ppa008902m	4.36	0.00065183	carboxyesterase 13	Red ontogenesis	
ppa005475m	4.36	0.002170803	Uridine diphosphate glycosyltransferase 74E2	Red ontogenesis	
ppa012496m	4.36	0.001613806	Uridine diphosphate glycosyltransferase 74E2	Red ontogenesis	
ppa004804m	4.36	6.87E-07	don-glucosyltransferase 1	Red ontogenesis	
ppa008301m	4.36	0.005631363	NAC (No Apical Meristem) domain transcriptional regulator super	Red ontogenesis	
ppa010186m	4.36	6.11E-05	ethylene-responsive element binding factor 13	Red ontogenesis	
ppa018582m	4.34	0.000983507	2-oxoglutarate (2OG) and Fe(II)-dependent oxygenase superfami	Red ontogenesis	
ppa004463m	4.34	0.000114487	Cytochrome P450 superfamily protein	Red ontogenesis	
ppa024699m	4.33	0.030214941	Plant protein of unknown function (DUF247)	Red ontogenesis	
ppa004984m	4.32	4.25E-06	cytochrome P450, family 716, subfamily A, polypeptide 1	Red ontogenesis	
ppa022619m	4.31	0.036576353	ABI five binding protein 3	Red ontogenesis	
ppa014729m	4.31	0.000260349	UDP-glucosyl transferase 73C1	Red ontogenesis	
ppa009297m	4.30	7.66E-05	NAD(P)-binding Rossmann-fold superfamily protein	Red ontogenesis	
ppa014245m	4.30	0.00032168		Red ontogenesis	
ppa009438m	4.29	0.002865817	NAC (No Apical Meristem) domain transcriptional regulator super	Red ontogenesis	
ppa010821m	4.28	0.000604042	S-adenosyl-L-methionine-dependent methyltransferases superfa	Red ontogenesis	
ppa023839m	4.28	0.040917247	ethylene response factor 1	Red ontogenesis	
ppa000961m	4.28	7.55E-05	NB-ARC domain-containing disease resistance protein	Red ontogenesis	
ppa007668m	4.28	0.018166136	elicitor-activated gene 3-1	Red ontogenesis	Secondary Metabolism / Phenylpropanoids - Biotic Stress / Secondary metabolism involved into stress
ppa006913m	4.27	0.000746587	ADP/ATP carrier 2	Red ontogenesis	
ppa016729m	4.27	0.000316123		Red ontogenesis	
ppa007577m	4.26	4.90E-05	NAC domain containing protein 2	Red ontogenesis	
ppa014817m	4.26	0.000353871	alternative oxidase 1A	Red ontogenesis	
ppa025672m	4.25	0.030328401	myb domain protein 108	Red ontogenesis	
ppa014659m	4.25	6.83E-07	glutamate receptor 2.7	Red ontogenesis	
ppa022188m	4.25	0.000474456		Red ontogenesis	
ppa012897m	4.25	3.33E-05		Red ontogenesis	
ppa005728m	4.24	0.001024886	Plant protein of unknown function (DUF247)	Red ontogenesis	
ppa008504m	4.24	0.008250611	Peroxidase superfamily protein	Red ontogenesis	
ppa004646m	4.23	2.70E-07	Cytochrome P450 superfamily protein	Red ontogenesis	
ppa020127m	4.23	5.32E-05	Calcium-dependent lipid-binding (CaLB domain) family protein	Red ontogenesis	
ppa010053m	4.23	0.001018919		Red ontogenesis	
ppa017730m	4.23	0.000874147		Red ontogenesis	
ppa018147m	4.22	0.029102184	Plant protein of unknown function (DUF247)	Red ontogenesis	
ppa001941m	4.22	7.99E-06	cellulose synthase like E1	Red ontogenesis	
ppa012676m	4.22	5.11E-06	MLP-like protein 423	Red ontogenesis	
ppa018138m	4.22	0.001006336	Late embryogenesis abundant (LEA) hydroxyproline-rich glycopro	Red ontogenesis	
ppa007298m	4.22	3.21E-06	protein kinase 2B	Red ontogenesis	
ppa012449m	4.21	1.35E-05	Adenine nucleotide alpha hydrolases-like superfamily protein	Red ontogenesis	
ppa022996m	4.21	0.001099345	Integrase-type DNA-binding superfamily protein	Red ontogenesis	
ppa013370m	4.20	0.010138558	basic pathogenesis-related protein 1	Red ontogenesis	
ppa021284m	4.20	0.024998115	Regulator of Vps4 activity in the MVB pathway protein	Red ontogenesis	
ppa004979m	4.19	1.88E-06	UDP-glucosyl transferase 85A2	Red ontogenesis	
ppa016353m	4.19	0.001673107	RING/U-box superfamily protein	Red ontogenesis	
ppa005406m	4.19	4.17E-05	UDP-Glycosyltransferase superfamily protein	Red ontogenesis	
ppa023045m	4.19	0.016349304	cysteine-rich RLK (RECEPTOR-like protein kinase) 29	Red ontogenesis	
ppa018396m	4.18	0.006648758	eukaryotic initiation factor 4A-III	Red ontogenesis	
ppa004530m	4.18	6.65E-05	nitrate transporter2.5	Red ontogenesis	
ppa026623m	4.18	3.29E-05	carboxyesterase 20	Red ontogenesis	
ppa013670m	4.18	0.000156289	Uncharacterised protein family (UPF0041)	Red ontogenesis	
ppa014274m	4.17	0.00081922		Red ontogenesis	
ppa002863m	4.17	1.43E-06	flavin-binding, kelch repeat, f box 1	Red ontogenesis	
ppa015974m	4.16	0.030505457	ankyrin repeat family protein	Red ontogenesis	
ppa008772m	4.15	0.001565238	Integrase-type DNA-binding superfamily protein	Red ontogenesis	
ppa010935m	4.15	0.02931516	C-repeat-binding factor 4	Red ontogenesis	
ppa013082m	4.15	0.000759102		Red ontogenesis	
ppa023148m	4.14	3.62E-05	Leucine-rich receptor-like protein kinase family protein	Red ontogenesis	
ppa021967m	4.14	0.022447148		Red ontogenesis	
ppb016383m	4.14	0.001093913	nucleic acid binding;zinc ion binding	Red ontogenesis	
ppa016463m	4.13	0.0001131	UDP-glucosyl transferase 74B1	Red ontogenesis	
ppa016616m	4.12	0.000366437	germin-like protein 2	Red ontogenesis	
ppa011660m	4.12	4.11E-05	RAB GTPase homolog 1A	Red ontogenesis	
ppa014273m	4.12	0.000422515		Red ontogenesis	
ppa020879m	4.12	0.001578142	MLP-like protein 423	Red ontogenesis	
ppa020780m	4.12	0.025174849	WRKY DNA-binding protein 72	Red ontogenesis	
ppa007600m	4.11	1.53E-05	senescence-related gene 1	Red ontogenesis	
ppa012458m	4.10	0.005113905		Red ontogenesis	
ppa024723m	4.10	0.000688214	RING/U-box superfamily protein	Red ontogenesis	
ppa026475m	4.08	0.001578937	2-oxoglutarate (2OG) and Fe(II)-dependent oxygenase superfami	Red ontogenesis	
ppa017380m	4.07	0.018619492	Late embryogenesis abundant (LEA) hydroxyproline-rich glycopro	Red ontogenesis	
ppa022640m	4.07	0.034448329	ankyrin repeat family protein	Red ontogenesis	
ppa003431m	4.07	0.005786104	Haem oxygenase-like, multi-helical	Red ontogenesis	
ppa017413m	4.07	8.86E-05		Red ontogenesis	
ppa015750m	4.07	0.032726824	RING/U-box superfamily protein	Red ontogenesis	
ppa024749m	4.07	0.003322857	Ribosomal protein L25/Gln-tRNA synthetase, anti-codon-binding c	Red ontogenesis	
ppa016153m	4.06	6.95E-06	WRKY DNA-binding protein 75	Red ontogenesis	
ppa014824m	4.06	7.19E-05	wall associated kinase-like 1	Red ontogenesis	
ppa011753m	4.05	0.000158832	acylaminoacyl-peptidase-related	Red ontogenesis	
ppa015981m	4.05	0.000370104		Red ontogenesis	
ppa024913m	4.05	0.019620665	Protein kinase superfamily protein	Red ontogenesis	
ppa013811m	4.05	0.000291001	peptidoglycan-binding LysM domain-containing protein	Red ontogenesis	
ppa018059m	4.05	3.75E-05	UDP-glucosyl transferase 73B3	Red ontogenesis	
ppa015136m	4.04	0.002643248	DVL family protein	Red ontogenesis	
ppa027148m	4.04	0.007074301	cinnamyl alcohol dehydrogenase 9	Red ontogenesis	Secondary Metabolism / Phenylpropanoids - Biotic Stress / Secondary metabolism involved into stress
ppa008310m	4.03	0.002377521	gibberellin 2-oxidase 6	Red ontogenesis	
ppa023393m	4.03	0.001274327	2-oxoglutarate (2OG) and Fe(II)-dependent oxygenase superfami	Red ontogenesis	
ppa018629m	4.03	2.46E-05	Subtilase family protein	Red ontogenesis	
ppa009610m	4.02	0.000561683	xyloglucan endotransglycosylase 6	Red ontogenesis	
ppa022234m	4.02	0.004662679	Predicted AT-hook DNA-binding family protein	Red ontogenesis	
ppa002334m	4.01	0.014820382	Glycosyl hydrolases family 32 protein	Red ontogenesis	
ppa026315m	4.00	0.000116331	myb domain protein 63	Red ontogenesis	
ppa019483m	4.00	0.017167522		Red ontogenesis	
ppa020571m	3.99	0.001569926	Leucine-rich repeat protein kinase family protein	Red ontogenesis	
ppa021909m	3.99	0.004333354	CRINKLY4 related 4	Red ontogenesis	

ppa003134m	3.99	0.010097303	Auxin-responsive GH3 family protein	Red ontogenesis	
ppa012452m	3.99	0.002321114		Red ontogenesis	
ppa026275m	3.99	0.000690042		Red ontogenesis	
ppa004999m	3.99	8.08E-07	cytochrome P450, family 716, subfamily A, polypeptide 1	Red ontogenesis	
ppa017581m	3.96	0.007893289	TTF-type zinc finger protein with HAT dimerisation domain	Red ontogenesis	
ppa026609m	3.96	0.007767546	Ankyrin repeat family protein	Red ontogenesis	
ppa002615m	3.95	0.001799813	cysteine-rich RLK (RECEPTOR-like protein kinase) 6	Red ontogenesis	
ppa018914m	3.95	5.44E-05	phosphate transporter 3;2	Red ontogenesis	
ppa019699m	3.95	5.52E-06	UDP-glucosyl transferase 73B3	Red ontogenesis	
ppa020294m	3.95	0.000138863	ENTH/ANTH/VHS superfamily protein	Red ontogenesis	
ppa022472m	3.94	0.000108352	2-oxoglutarate (2OG) and Fe(II)-dependent oxygenase superfami	Red ontogenesis	
ppa020389m	3.93	0.026129652		Red ontogenesis	
ppa007918m	3.92	0.00017998	phosphate transporter 3;2	Red ontogenesis	
ppa021614m	3.92	0.009056255		Red ontogenesis	
ppa018783m	3.91	0.0012313	Leucine-rich receptor-like protein kinase family protein	Red ontogenesis	
ppb020933m	3.91	0.00741948	Leucine-rich repeat (LRR) family protein	Red ontogenesis	
ppa014306m	3.91	0.003241199		Red ontogenesis	
ppa023067m	3.91	0.011288716	BON association protein 2	Red ontogenesis	
ppa009647m	3.90	0.00121133	WRKY DNA-binding protein 65	Red ontogenesis	
ppa004956m	3.89	6.22E-06	UDP-glucosyl transferase 85A2	Red ontogenesis	
ppa016829m	3.88	0.038321596	Integrase-type DNA-binding superfamily protein	Red ontogenesis	
ppa023716m	3.88	0.000589843	glutathione S-transferase TAU 8	Red ontogenesis	Biotic Stress / Glutathione-S-Transferase
ppa021408m	3.88	0.001232953	calmodulin-like 41	Red ontogenesis	
ppa006165m	3.88	0.024819927	CYS, MET, PRO, and GLY protein 2	Red ontogenesis	
ppa007840m	3.87	0.000665161	2-oxoglutarate (2OG) and Fe(II)-dependent oxygenase superfami	Red ontogenesis	
ppa006448m	3.87	0.007819594	plant U-box 29	Red ontogenesis	
ppa005368m	3.87	5.11E-06	serine carboxypeptidase-like 34	Red ontogenesis	
ppb015892m	3.87	0.043725949	UDP-glucosyl transferase 85A2	Red ontogenesis	
ppa024840m	3.87	7.06E-06	HCO3- transporter family	Red ontogenesis	
ppa026286m	3.87	0.022073603	Polynucleotidyl transferase, ribonuclease H-like superfamily prote	Red ontogenesis	
ppa001293m	3.87	0.008505748	lipoxygenase 1	Red ontogenesis	
ppa027069m	3.86	0.001742179		Red ontogenesis	
ppa008877m	3.86	0.000305146	myb domain protein 116	Red ontogenesis	
ppa015845m	3.86	0.036570902	UDP-glucosyl transferase 71B5	Red ontogenesis	
ppa003269m	3.85	4.90E-05	Calmodulin-binding protein	Red ontogenesis	
ppa016615m	3.84	0.027873171	Transmembrane amino acid transporter family protein	Red ontogenesis	
ppa014652m	3.83	5.11E-06	NAD(P)-binding Rossmann-fold superfamily protein	Red ontogenesis	
ppa002895m	3.82	0.000313285	cysteine-rich RLK (RECEPTOR-like protein kinase) 3	Red ontogenesis	
ppa015511m	3.82	0.004947286	RING/U-box superfamily protein	Red ontogenesis	
ppa023607m	3.82	0.027623438	NOD26-like major intrinsic protein 1	Red ontogenesis	
ppa011306m	3.82	0.006587823	Acyl-CoA N-acyltransferases (NAT) superfamily protein	Red ontogenesis	
ppa021329m	3.81	0.043317752	lycopene cyclase	Red ontogenesis	Biotic Stress / Secondary metabolism involved into stress
ppa021861m	3.81	1.92E-06	carboxyesterase 13	Red ontogenesis	
ppb021349m	3.81	0.012836673	wall associated kinase 3	Red ontogenesis	
ppa016848m	3.81	0.001670754	polyubiquitin 10	Red ontogenesis	
ppa027036m	3.80	0.000134419		Red ontogenesis	
ppa021540m	3.80	0.000248192	Homeodomain-like superfamily protein	Red ontogenesis	
ppa017389m	3.80	0.000815993	S-locus lectin protein kinase family protein	Red ontogenesis	
ppa024607m	3.79	0.009811549		Red ontogenesis	
ppa023567m	3.78	0.001631533	PLANT CADMIUM RESISTANCE 2	Red ontogenesis	
ppa022943m	3.78	0.000193898		Red ontogenesis	
ppa018534m	3.78	0.003116057		Red ontogenesis	
ppa015075m	3.77	0.031066651	Receptor-like protein kinase-related family protein	Red ontogenesis	
ppa015877m	3.77	9.60E-05	S-locus lectin protein kinase family protein	Red ontogenesis	
ppa017487m	3.76	0.00515885	xyloglucan endotransglucosylase/hydrolase 16	Red ontogenesis	
ppa014862m	3.76	0.008509365	Protein kinase superfamily protein	Red ontogenesis	
ppa004523m	3.75	2.52E-05	beta-glucosidase 45	Red ontogenesis	
ppa007445m	3.75	0.002112901	NAC domain containing protein 47	Red ontogenesis	
ppa002432m	3.75	0.002000928	wall-associated kinase 2	Red ontogenesis	
ppa009372m	3.75	6.83E-07	atypical CYS HIS rich thioredoxin 4	Red ontogenesis	
ppa023118m	3.74	0.017317509	NB-ARC domain-containing disease resistance protein	Red ontogenesis	
ppa025240m	3.74	3.73E-05	PYR1-like 4	Red ontogenesis	
ppa010647m	3.74	0.002582709	homeobox 7	Red ontogenesis	
ppa007606m	3.74	9.63E-05	Integrase-type DNA-binding superfamily protein	Red ontogenesis	
ppa016223m	3.73	0.002474435	P-loop containing nucleoside triphosphate hydrolases superfamily	Red ontogenesis	
ppa010660m	3.72	7.65E-05	pathogenesis-related family protein	Red ontogenesis	
ppa024588m	3.72	1.91E-06	NAD(P)-linked oxidoreductase superfamily protein	Red ontogenesis	
ppa003180m	3.72	3.65E-05	Major facilitator superfamily protein	Red ontogenesis	
ppa005959m	3.71	5.44E-05	alpha/beta-Hydrolases superfamily protein	Red ontogenesis	
ppa014989m	3.70	0.001007113	senescence-related gene 1	Red ontogenesis	
ppa020356m	3.70	0.00020718	Barwin-related endoglucanase	Red ontogenesis	
ppa015501m	3.70	2.84E-05		Red ontogenesis	
ppa011108m	3.70	1.66E-06	glutathione S-transferase tau 7	Red ontogenesis	Biotic Stress / Glutathione-S-Transferase
ppa005062m	3.70	0.009098301	SLAC1 homologue 3	Red ontogenesis	
ppa001241m	3.69	1.72E-05	K+ transporter 1	Red ontogenesis	
ppa003777m	3.69	0.012941972	calmodulin-binding family protein	Red ontogenesis	
ppa002027m	3.69	0.00465053	S-locus lectin protein kinase family protein	Red ontogenesis	
ppa017664m	3.68	2.70E-07	S-locus lectin protein kinase family protein	Red ontogenesis	
ppa020392m	3.68	0.006551708	Ankyrin repeat family protein	Red ontogenesis	
ppa012928m	3.68	6.94E-05	glutathione S-transferase tau 7	Red ontogenesis	Biotic Stress / Glutathione-S-Transferase
ppa024674m	3.68	0.00135479	2-oxoglutarate (2OG) and Fe(II)-dependent oxygenase superfami	Red ontogenesis	
ppa002988m	3.68	4.08E-05	Calmodulin-binding protein	Red ontogenesis	
ppa013829m	3.67	0.00010301		Red ontogenesis	
ppa009038m	3.67	7.83E-06		Red ontogenesis	
ppa025857m	3.66	0.03771441	Bifunctional inhibitor/lipid-transfer protein/seed storage 2S albumi	Red ontogenesis	
ppa022686m	3.66	0.000121394	receptor serine/threonine kinase, putative	Red ontogenesis	
ppa022671m	3.66	0.022194512		Red ontogenesis	
ppa005724m	3.66	0.000132275	RING domain ligase1	Red ontogenesis	
ppa004957m	3.66	2.08E-05	UDP-Glycosyltransferase superfamily protein	Red ontogenesis	
ppb022522m	3.65	0.006072482		Red ontogenesis	
ppa014158m	3.65	0.00508219		Red ontogenesis	
ppa023285m	3.65	0.04160865	Ankyrin repeat family protein	Red ontogenesis	
ppa020972m	3.64	0.004026668	basic helix-loop-helix (bHLH) DNA-binding family protein	Red ontogenesis	
ppa018910m	3.64	2.04E-05	ARM repeat superfamily protein	Red ontogenesis	
ppa009584m	3.64	0.000692263	EID1-like 3	Red ontogenesis	
ppa026582m	3.64	0.000228124	NAC domain containing protein 83	Red ontogenesis	
ppa008956m	3.64	1.95E-05	Coatomer, beta\' subunit	Red ontogenesis	
ppa021105m	3.63	0.041029017	General transcription factor 2-related zinc finger protein	Red ontogenesis	
ppa001889m	3.63	1.13E-05	cellulose synthase-like B3	Red ontogenesis	
ppa008552m	3.62	0.001694393	alpha/beta-Hydrolases superfamily protein	Red ontogenesis	
ppa024554m	3.62	0.002388616	disease resistance family protein / LRR family protein	Red ontogenesis	
ppa021725m	3.61	0.000562283	NAD(P)-linked oxidoreductase superfamily protein	Red ontogenesis	
ppa017528m	3.61	7.26E-07	Leucine-rich receptor-like protein kinase family protein	Red ontogenesis	
ppa003236m	3.61	1.58E-05	Domain of unknown function (DUF23)	Red ontogenesis	
ppa010429m	3.60	0.001118249	cold regulated gene 27	Red ontogenesis	
ppa020322m	3.60	1.67E-05	lectin receptor kinase a4.1	Red ontogenesis	
ppa023004m	3.60	0.001444312	calmodulin-like 11	Red ontogenesis	
ppa001718m	3.59	0.006874609	beta-xylosidase 1	Red ontogenesis	
ppa008025m	3.59	3.64E-05	NAD(P)-linked oxidoreductase superfamily protein	Red ontogenesis	
ppa022259m	3.58	0.001066526	Calcium-binding EF-hand family protein	Red ontogenesis	
ppa017661m	3.58	0.000128978	Coatomer, beta\' subunit	Red ontogenesis	
ppa005064m	3.58	3.78E-05	Protein kinase protein with tetratricopeptide repeat domain	Red ontogenesis	
ppa015132m	3.58	0.030967264	threonine aldolase 2	Red ontogenesis	
ppa017836m	3.58	0.024151242	AP2/B3-like transcriptional factor family protein	Red ontogenesis	
ppa025637m	3.57	0.011520785	pinoid-binding protein 1	Red ontogenesis	
ppa014479m	3.57	0.000467065		Red ontogenesis	
ppa008480m	3.56	0.000359888	WRKY DNA-binding protein 57	Red ontogenesis	
ppa021290m	3.56	0.005919979		Red ontogenesis	
ppa021469m	3.55	4.62E-07	polyol/monosaccharide transporter 5	Red ontogenesis	
ppa011230m	3.54	0.000313285	Peroxisomal membrane 22 kDa (Mpv17/PMP22) family protein	Red ontogenesis	
ppa008573m	3.54	0.002195088	WRKY family transcription factor	Red ontogenesis	
ppa003639m	3.54	0.000736777	Plant invertase/pectin methylesterase inhibitor superfamily	Red ontogenesis	
ppa004555m	3.53	2.69E-05	aldehyde dehydrogenase 2C4	Red ontogenesis	
ppa026669m	3.53	9.91E-05	Ubiquitin fusion degradation UFD1 family protein	Red ontogenesis	
ppa017617m	3.53	0.000270511	Protein kinase superfamily protein	Red ontogenesis	
ppa026587m	3.52	0.020996286	Chlorophyll A-B binding family protein	Red ontogenesis	
ppa024291m	3.52	0.007525695	NAD(P)-linked oxidoreductase superfamily protein	Red ontogenesis	Biotic Stress / Secondary metabolism involved into stress
ppa003583m	3.51	5.22E-05	Laccase/Diphenol oxidase family protein	Red ontogenesis	Biotic Stress / Secondary metabolism involved into stress
ppa025836m	3.51	0.035207676		Red ontogenesis	
ppa006967m	3.51	8.34E-07	purine permease 10	Red ontogenesis	
ppa004132m	3.50	0.001872839	Major facilitator superfamily protein	Red ontogenesis	
ppa022821m	3.50	0.009269632	Protein kinase superfamily protein	Red ontogenesis	
ppa008125m	3.49	0.011755543	gibberellin 2-oxidase 8	Red ontogenesis	
ppa020910m	3.49	0.024403373	Calcium-binding EF-hand family protein	Red ontogenesis	
ppa015844m	3.49	1.31E-05	phospholipase A 2A	Red ontogenesis	
ppa020109m	3.49	0.036533165	NAD(P)-binding Rossmann-fold superfamily protein	Red ontogenesis	
ppa018353m	3.48	0.00045116		Red ontogenesis	
ppa008039m	3.47	0.001292338	Protein of unknown function (DUF1645)	Red ontogenesis	
ppa017572m	3.47	1.66E-06	receptor kinase 3	Red ontogenesis	
ppa023736m	3.46	0.007802495	Integrase-type DNA-binding superfamily protein	Red ontogenesis	
ppa001414m	3.46	3.99E-06	S-locus lectin protein kinase family protein	Red ontogenesis	
ppb011375m	3.45	0.000508561	2-oxoglutarate (2OG) and Fe(II)-dependent oxygenase superfami	Red ontogenesis	
ppa018817m	3.45	1.72E-05	Wall-associated kinase family protein	Red ontogenesis	
ppa014856m	3.44	0.013442103		Red ontogenesis	
ppa014931m	3.44	0.000999503	plant U-box 23	Red ontogenesis	
ppa008811m	3.44	0.037233621	UDP-Glycosyltransferase superfamily protein	Red ontogenesis	
ppa021460m	3.44	0.026480622	geranylgeranyl pyrophosphate synthase 1	Red ontogenesis	Biotic Stress / Secondary metabolism involved into stress
ppa018504m	3.44	1.43E-06	Leucine-rich repeat receptor-like protein kinase family protein	Red ontogenesis	

ppa023071m	3.44	4.65E-05	UDP-Glycosyltransferase superfamily protein	Red ontogenesis	
ppa024408m	3.44	0.031254701	wall associated kinase-like 7	Red ontogenesis	
ppa001663m	3.43	7.08E-05	S-locus lectin protein kinase family protein	Red ontogenesis	
ppa018053m	3.43	3.73E-05	CCT motif family protein	Red ontogenesis	
ppa011907m	3.43	0.007559707	Chlorophyll A-B binding family protein	Red ontogenesis	
ppa016109m	3.42	0.039586184	related to AP2 11	Red ontogenesis	
ppa019731m	3.41	1.01E-05	cytochrome P450, family 82, subfamily C, polypeptide 4	Red ontogenesis	
ppa017937m	3.41	0.045935238	Disease resistance protein (TIR-NBS-LRR class) family	Red ontogenesis	Biotic Stress / PR-proteins
ppa020399m	3.41	0.018312682	Protein of unknown function (DUF569)	Red ontogenesis	
ppa011082m	3.41	0.002207808		Red ontogenesis	
ppa024933m	3.41	0.007001489	ATPase E1-E2 type family protein / haloacid dehalogenase-like h	Red ontogenesis	
ppa010683m	3.40	2.04E-05	indole-3-acetic acid inducible 29	Red ontogenesis	
ppa010345m	3.39	0.047264616	Integrase-type DNA-binding superfamily protein	Red ontogenesis	
ppa012476m	3.39	0.024172005	Late Embryogenesis Abundant 4-5	Red ontogenesis	
ppa011329m	3.38	0.000169895	SSXT family protein	Red ontogenesis	
ppa007336m	3.38	0.003686112	gibberellin 3-oxidase 1	Red ontogenesis	
ppa020030m	3.38	0.004015377	bidirectional amino acid transporter 1	Red ontogenesis	
ppa022455m	3.38	0.029477723	Eukaryotic aspartyl protease family protein	Red ontogenesis	
ppa020720m	3.37	0.000377604	Calcium-binding EF-hand family protein	Red ontogenesis	
ppa015527m	3.37	0.00237355		Red ontogenesis	
ppa023275m	3.37	0.006025666	Ankyrin repeat family protein	Red ontogenesis	
ppa012272m	3.36	4.06E-06	Chaperone DnaJ-domain superfamily protein	Red ontogenesis	
ppa010059m	3.35	0.00832562	RING/U-box superfamily protein	Red ontogenesis	
ppa026571m	3.35	0.007551596	AGAMOUS-like 62	Red ontogenesis	
ppa007339m	3.35	0.000417493	purine permease 3	Red ontogenesis	
ppa014816m	3.34	1.26E-05	UDP-glucosyl transferase 73B3	Red ontogenesis	
ppa023708m	3.34	0.00132617	cullin 1	Red ontogenesis	
ppa002322m	3.33	0.001023354	ARM repeat superfamily protein	Red ontogenesis	
ppa003358m	3.33	0.000172353	AZA-guanine resistant1	Red ontogenesis	
ppa012715m	3.33	0.000312936		Red ontogenesis	
ppa019102m	3.33	0.000135459	RING/U-box superfamily protein	Red ontogenesis	
ppa017233m	3.32	0.002007359	2-oxoglutarate (2OG) and Fe(II)-dependent oxygenase superfami	Red ontogenesis	
ppa018361m	3.32	0.021665885	Wall-associated kinase family protein	Red ontogenesis	
ppa007669m	3.32	0.001457091	HSP40/DnaJ peptide-binding protein	Red ontogenesis	
ppa012014m	3.32	0.000470854	ethylene responsive element binding factor 2	Red ontogenesis	
ppa010137m	3.31	0.00015663	S-adenosyl-L-methionine-dependent methyltransferases superfar	Red ontogenesis	
ppa025491m	3.31	0.000148692	disease resistance family protein / LRR family protein	Red ontogenesis	
ppa025256m	3.31	3.09E-05	Alpha/beta hydrolase related protein	Red ontogenesis	
ppa005514m	3.31	0.005962284	cold-regulated 47	Red ontogenesis	
ppa014312m	3.30	3.72E-05		Red ontogenesis	
ppa005683m	3.30	2.40E-05	Major facilitator superfamily protein	Red ontogenesis	
ppa004722m	3.30	5.10E-06	Methylenetetrahydrofolate reductase family protein	Red ontogenesis	
ppa018275m	3.30	0.005530508	osmotin 34	Red ontogenesis	
ppa017530m	3.30	0.003971525	sulfur E2	Red ontogenesis	
ppa001397m	3.29	0.001018919	trehalose-phosphatase/synthase 9	Red ontogenesis	
ppa016970m	3.29	0.040961657	ascorbic acid mannose pathway regulator 1	Red ontogenesis	
ppa023836m	3.29	0.001902041	lipid transfer protein 1	Red ontogenesis	
ppa006398m	3.29	4.20E-05	glutamate decarboxylase	Red ontogenesis	
ppa020980m	3.29	0.0040008	ankyrin repeat family protein	Red ontogenesis	
ppa026954m	3.28	0.0007834	2-oxoglutarate (2OG) and Fe(II)-dependent oxygenase superfami	Red ontogenesis	
ppa002188m	3.28	9.81E-06	pseudo-response regulator 5	Red ontogenesis	
ppa018836m	3.27	0.005823232	UDP-Glycosyltransferase superfamily protein	Red ontogenesis	
ppa009844m	3.27	0.000924359	homolog of carrot EP3-3 chitinase	Red ontogenesis	
ppa004537m	3.27	0.015496218	bZIP transcription factor family protein	Red ontogenesis	
ppb023904m	3.26	0.003119719	Leucine-rich receptor-like protein kinase family protein	Red ontogenesis	
ppa021591m	3.25	0.001335299	methyl esterase 3	Red ontogenesis	
ppa012627m	3.24	0.0019509	MLP-like protein 423	Red ontogenesis	
ppa019080m	3.24	0.00300581	Protein kinase family protein with leucine-rich repeat domain	Red ontogenesis	
ppa024931m	3.24	0.003874976	Calcium-binding EF-hand family protein	Red ontogenesis	
ppa022765m	3.24	0.002271436	calmodulin-binding family protein	Red ontogenesis	
ppa004004m	3.24	5.64E-05	phosphate transporter 1;5	Red ontogenesis	
ppa022963m	3.24	0.000373158	phytochrome-interacting factor7	Red ontogenesis	
ppa022463m	3.23	0.000351356	isopentenyltransferase 5	Red ontogenesis	
ppa013848m	3.23	0.000723344		Red ontogenesis	
ppa016510m	3.23	0.00734767	homeobox from Arabidopsis thaliana	Red ontogenesis	
ppa003580m	3.23	0.000247537	laccase 14	Red ontogenesis	Biotic Stress / Secondary metabolism involved into stress
ppa024446m	3.22	0.009161455	Acyl transferase/acyl hydrolase/lysophospholipase superfamily pr	Red ontogenesis	
ppa011684m	3.22	0.010341117	Uncharacterised protein family (UPF0497)	Red ontogenesis	
ppa019288m	3.22	0.001356593	Ankyrin repeat family protein	Red ontogenesis	
ppa009057m	3.22	5.20E-05	NAD(P)-linked oxidoreductase superfamily protein	Red ontogenesis	
ppa018260m	3.22	0.000717764	polyubiquitin 10	Red ontogenesis	
ppa026931m	3.22	6.22E-06	NAD(P)-binding Rossmann-fold superfamily protein	Red ontogenesis	
ppa011544m	3.22	0.046132705	Protein of unknown function (DUF567)	Red ontogenesis	
ppa008163m	3.22	1.19E-05	NAD(P)-linked oxidoreductase superfamily protein	Red ontogenesis	
ppa022051m	3.22	0.011539717	Uncharacterised protein family (UPF0114)	Red ontogenesis	
ppa021658m	3.22	0.005648387	Reticulan like protein B13	Red ontogenesis	
ppa006516m	3.21	4.44E-05	Transmembrane amino acid transporter family protein	Red ontogenesis	
ppa022386m	3.21	0.03546547	Peroxidase superfamily protein	Red ontogenesis	
ppa010822m	3.21	7.26E-07	K-box region and MADS-box transcription factor family protein	Red ontogenesis	
ppa015431m	3.20	0.000206602	S-locus lectin protein kinase family protein	Red ontogenesis	
ppa020990m	3.20	0.039252452		Red ontogenesis	
ppa012970m	3.20	7.58E-06	Protein of unknown function (DUF581)	Red ontogenesis	
ppa010714m	3.19	4.45E-05	K-box region and MADS-box transcription factor family protein	Red ontogenesis	
ppa019532m	3.19	0.000205953	phospholipase A 2A	Red ontogenesis	
ppa017040m	3.19	0.000194731		Red ontogenesis	
ppa014950m	3.19	0.004469169	P-loop containing nucleoside triphosphate hydrolases superfamily	Red ontogenesis	
ppa019182m	3.19	0.003571454	12-oxophytodienoate reductase 2	Red ontogenesis	
ppa015290m	3.19	0.014823045	Oxidoreductase, zinc-binding dehydrogenase family protein	Red ontogenesis	
ppa000937m	3.19	3.32E-05	H(+)-ATPase 2	Red ontogenesis	
ppa015481m	3.18	4.43E-05	NAD(P)-linked oxidoreductase superfamily protein	Red ontogenesis	
ppa007999m	3.18	0.005921087	Glycosyl hydrolase superfamily protein	Red ontogenesis	Biotic Stress / Betaglucanase
ppa020329m	3.18	1.72E-05	PR5-like receptor kinase	Red ontogenesis	
ppa016018m	3.17	0.001041135	PR5-like receptor kinase	Red ontogenesis	
ppa024831m	3.17	0.04310612	Disease resistance protein (TIR-NBS-LRR class) family	Red ontogenesis	Biotic Stress / PR-proteins
ppa026709m	3.17	0.001864653	Protein of unknown function, DUF599	Red ontogenesis	
ppa020614m	3.16	0.002779296	Dynein light chain type 1 family protein	Red ontogenesis	
ppa005770m	3.15	0.000520915	Arabidopsis NAC domain containing protein 87	Red ontogenesis	
ppa001867m	3.15	0.00014311	cellulose synthase like G2	Red ontogenesis	
ppa006969m	3.15	0.014460089		Red ontogenesis	
ppa025335m	3.15	0.000753127	response regulator 9	Red ontogenesis	
ppa011793m	3.15	0.012361994		Red ontogenesis	
ppa011115m	3.14	3.80E-05	glutathione S-transferase tau 7	Red ontogenesis	Biotic Stress / Glutathione-S-Transferase
ppa014106m	3.14	0.005191319		Red ontogenesis	
ppa012435m	3.14	0.000134336		Red ontogenesis	
ppa013059m	3.14	1.53E-05	CCR-like	Red ontogenesis	
ppa021762m	3.14	3.00E-06	polyol/monosaccharide transporter 5	Red ontogenesis	
ppa011228m	3.14	5.11E-06	Tetraspanin family protein	Red ontogenesis	
ppa018676m	3.14	0.006619921	MATE efflux family protein	Red ontogenesis	
ppa011401m	3.14	0.00915477		Red ontogenesis	
ppa019328m	3.13	0.001922894	S-locus lectin protein kinase family protein	Red ontogenesis	
ppa010465m	3.13	0.000590597	RING/U-box superfamily protein	Red ontogenesis	
ppa022461m	3.12	5.51E-05	Leucine-rich repeat receptor-like protein kinase family protein	Red ontogenesis	
ppa014655m	3.12	0.00642559	glutamate receptor 1.3	Red ontogenesis	
ppa001891m	3.12	0.000114909	glutamate receptor 2.1	Red ontogenesis	
ppa027031m	3.11	0.002847014	DNAse I-like superfamily protein	Red ontogenesis	
ppa012940m	3.11	0.000153486	Late embryogenesis abundant protein	Red ontogenesis	
ppa004936m	3.10	1.26E-05	amino acid permease 3	Red ontogenesis	
ppa005625m	3.09	1.42E-05	HXXXD-type acyl-transferase family protein	Red ontogenesis	Secondary Metabolism / Phenylpropanoids - Biotic Stress / Secondary metabolism involved into stress
ppa011092m	3.09	2.13E-05	Protein of unknown function (DUF567)	Red ontogenesis	
ppa022914m	3.09	0.004947286	disease resistance protein (TIR-NBS-LRR class), putative	Red ontogenesis	Biotic Stress / PR-proteins
ppa022021m	3.09	0.034068558	Rhamnogalacturonate lyase family protein	Red ontogenesis	
ppa020051m	3.09	3.62E-05	zinc transporter 1 precursor	Red ontogenesis	
ppa009106m	3.09	0.000163967	Drug/metabolite transporter superfamily protein	Red ontogenesis	
ppa007986m	3.09	0.045523676	WRKY family transcription factor	Red ontogenesis	
ppa013714m	3.09	0.008237542	GAST1 protein homolog 1	Red ontogenesis	
ppa007379m	3.09	0.001670754	12-oxophytodienoate reductase 2	Red ontogenesis	
ppa006485m	3.09	0.008197816	mitogen-activated protein kinase kinase kinase 15	Red ontogenesis	
ppa011283m	3.08	8.90E-06		Red ontogenesis	
ppa001311m	3.08	0.022370764	lipoxygenase 1	Red ontogenesis	
ppa010765m	3.08	0.000784604	alpha/beta-Hydrolases superfamily protein	Red ontogenesis	
ppa009871m	3.07	0.008277044	xyloglucan endotransglucosylase/hydrolase 16	Red ontogenesis	
ppa018601m	3.07	0.019153317	Integrase-type DNA-binding superfamily protein	Red ontogenesis	
ppa026526m	3.07	0.031085315	Calcium-dependent lipid-binding (CaLB domain) plant phosphorib	Red ontogenesis	
ppb016122m	3.06	0.015235396		Red ontogenesis	
ppa013490m	3.06	0.000226042		Red ontogenesis	
ppa004802m	3.06	7.03E-05	selenium-binding protein 2	Red ontogenesis	
ppb024827m	3.06	1.34E-05	glutathione S-transferase TAU 8	Red ontogenesis	Biotic Stress / Glutathione-S-Transferase
ppa025692m	3.06	0.009035018	disease resistance protein (TIR-NBS-LRR class), putative	Red ontogenesis	Biotic Stress / PR-proteins
ppa018846m	3.06	0.00168694	zinc finger (C3HC4-type RING finger) family protein	Red ontogenesis	
ppa000963m	3.05	4.88E-06		Red ontogenesis	
ppa025693m	3.05	5.88E-05	S-locus lectin protein kinase family protein	Red ontogenesis	
ppa023323m	3.05	0.002366482	Transducin/WD40 repeat-like superfamily protein	Red ontogenesis	
ppa020309m	3.05	0.004335268	Protein kinase superfamily protein	Red ontogenesis	
ppa006014m	3.05	1.12E-05	Histone H3 K4-specific methyltransferase SET7/9 family protein	Red ontogenesis	
ppa010796m	3.04	0.000805628	WRKY DNA-binding protein 40	Red ontogenesis	
ppa006776m	3.04	0.000723919	Auxin-responsive family protein	Red ontogenesis	
ppa019847m	3.03	0.000358847	Ankyrin repeat family protein	Red ontogenesis	
ppa023930m	3.03	1.12E-05	PR5-like receptor kinase	Red ontogenesis	

ppa002243m	3.02	0.000367547	*S-locus lectin protein kinase family protein*	Red ontogenesis	
ppa014444m	3.02	0.004292726		Red ontogenesis	
ppa011221m	3.02	0.0061034	*homeobox 7*	Red ontogenesis	
ppa025469m	3.01	0.003392053	*Calmodulin binding protein-like*	Red ontogenesis	
ppa016784m	3.01	0.00150854	*cysteine-rich RLK (RECEPTOR-like protein kinase) 29*	Red ontogenesis	
ppa010658m	3.01	0.007863183	*pathogenesis-related gene 5*	Red ontogenesis	
ppa026730m	3.00	0.014820475		Red ontogenesis	
ppa002716m	3.00	0.000436475	*Uncharacterised conserved protein UCP015417, vWA*	Red ontogenesis	
ppa008924m	3.00	0.011681055	*Protein of unknown function (DUF793)*	Red ontogenesis	
ppa003835m	3.00	4.58E-05	*NRAMP metal ion transporter 6*	Red ontogenesis	
ppa013687m	2.99	0.040678322		Red ontogenesis	
ppa014398m	2.99	0.046283385	*magnesium-chelatase subunit chlH, chloroplast, putative / Mg-pro*	Red ontogenesis	
ppa004329m	2.98	1.10E-05	*Cytochrome P450 superfamily protein*	Red ontogenesis	
ppa014483m	2.98	0.027418849		Red ontogenesis	
ppa006715m	2.97	0.00020087	*Homeodomain-like superfamily protein*	Red ontogenesis	
ppa021636m	2.97	0.010256423	*Transducin/WD40 repeat-like superfamily protein*	Red ontogenesis	
ppa005052m	2.97	8.73E-05	*UDP-glucosyl transferase 71B6*	Red ontogenesis	
ppa020211m	2.96	0.000509267	*ubiquitin-conjugating enzyme 32*	Red ontogenesis	
ppa014007m	2.96	0.045183556		Red ontogenesis	
ppa016653m	2.96	5.34E-05	*glutathione S-transferase tau 7*	Red ontogenesis	Biotic Stress / Glutathione-S-Transferase
ppa020335m	2.96	9.53E-05	*Leucine-rich repeat receptor-like protein kinase family protein*	Red ontogenesis	
ppa013218m	2.96	0.000643738		Red ontogenesis	
ppa007996m	2.95	0.002165525	*Galactose oxidase/kelch repeat superfamily protein*	Red ontogenesis	
ppa002471m	2.95	0.00215539	*O-fucosyltransferase family protein*	Red ontogenesis	
ppa022354m	2.95	5.11E-06	*Putative lysine decarboxylase family protein*	Red ontogenesis	
ppa009233m	2.94	1.19E-05	*Phenazine biosynthesis PhzC/PhzF protein*	Red ontogenesis	
ppa015322m	2.94	0.002891821	*disease resistance family protein / LRR family protein*	Red ontogenesis	
ppa019354m	2.94	5.10E-06	*UDP-glucosyl transferase 73B3*	Red ontogenesis	
ppa005425m	2.93	4.08E-05		Red ontogenesis	
ppa004796m	2.93	0.000347445	*glutamate decarboxylase*	Red ontogenesis	
ppa009966m	2.93	0.000916645	*myb domain protein 20*	Red ontogenesis	
ppa010862m	2.93	0.002580314	*NDR1/HIN1-like 3*	Red ontogenesis	
ppa011268m	2.93	0.001988141	*glutathione S-transferase tau 7*	Red ontogenesis	Biotic Stress / Glutathione-S-Transferase
ppa005956m	2.93	0.000717764	*Purple acid phosphatases superfamily protein*	Red ontogenesis	
ppa000895m	2.92	0.001856513	*Leucine-rich receptor-like protein kinase family protein*	Red ontogenesis	
ppa007374m	2.92	0.001778147	*GroES-like zinc-binding alcohol dehydrogenase family protein*	Red ontogenesis	
ppa025554m	2.92	0.000851069	*alpha/beta-Hydrolases superfamily protein*	Red ontogenesis	
ppa001287m	2.92	0.027724341	*lipoxygenase 1*	Red ontogenesis	
ppa016442m	2.92	0.002354512	*beta glucosidase 11*	Red ontogenesis	
ppa009581m	2.91	0.000357407		Red ontogenesis	
ppa006324m	2.91	0.032166282		Red ontogenesis	
ppa024908m	2.91	0.008250611	*Protein kinase superfamily protein*	Red ontogenesis	
ppa026113m	2.90	0.000773829	*disease resistance family protein / LRR family protein*	Red ontogenesis	
ppa004108m	2.90	0.037229952	*beta glucosidase 13*	Red ontogenesis	
ppa012081m	2.90	0.021486928		Red ontogenesis	
ppa022767m	2.90	0.000683459	*phytosulfokine 2 precursor*	Red ontogenesis	
ppa001761m	2.90	1.43E-06	*S-locus lectin protein kinase family protein*	Red ontogenesis	
ppa021071m	2.90	1.96E-05	*S-locus lectin protein kinase family protein*	Red ontogenesis	
ppa007699m	2.89	0.00938385	*metacaspase 1*	Red ontogenesis	
ppa014636m	2.89	0.000388535	*disease resistance family protein / LRR family protein*	Red ontogenesis	
ppa013385m	2.89	1.36E-05		Red ontogenesis	
ppa008600m	2.88	0.000378061	*O-acetylserine (thiol) lyase (OAS-TL) isoform A1*	Red ontogenesis	
ppa024923m	2.88	0.006369287	*pathogenesis-related gene 5*	Red ontogenesis	
ppa024672m	2.88	0.001585996	*Protein kinase superfamily protein*	Red ontogenesis	
ppa001159m	2.87	7.94E-05	*glutamate receptor 2.7*	Red ontogenesis	
ppa013035m	2.87	0.002167798		Red ontogenesis	
ppa021354m	2.87	0.008734551	*basic region/leucine zipper motif 27*	Red ontogenesis	
ppa011053m	2.87	0.001778147	*osmotin 34*	Red ontogenesis	
ppa016167m	2.87	0.014892235		Red ontogenesis	
ppa009924m	2.87	0.011606676	*salt tolerance zinc finger*	Red ontogenesis	
ppa013510m	2.87	7.45E-06	*dormancy-associated protein-like 1*	Red ontogenesis	
ppa005203m	2.86	0.000116119	*F-box family protein*	Red ontogenesis	
ppa026594m	2.86	8.27E-05	*disease resistance family protein / LRR family protein*	Red ontogenesis	
ppa008609m	2.85	0.000702827	*GRAM domain family protein*	Red ontogenesis	
ppa009118m	2.85	0.034904862	*nudix hydrolase homolog 14*	Red ontogenesis	
ppa016939m	2.85	5.72E-05	*Leucine-rich receptor-like protein kinase family protein*	Red ontogenesis	
ppa026799m	2.85	3.15E-06	*gibberellin 2-oxidase 8*	Red ontogenesis	
ppa007923m	2.85	0.000215956	*UDP-N-acetylglucosamine (UAA) transporter family*	Red ontogenesis	
ppa018866m	2.85	0.001069952	*methyl esterase 3*	Red ontogenesis	
ppa025118m	2.84	0.000285009	*acetyl CoA:(Z)-3-hexen-1-ol acetyltransferase*	Red ontogenesis	
ppa021414m	2.84	8.01E-05	*UDP-glucosyl transferase 85A2*	Red ontogenesis	
ppa1027196m	2.84	0.008070743	*pathogenesis-related gene 1*	Red ontogenesis	
ppa022095m	2.83	0.000723919	*cysteine-rich RLK (RECEPTOR-like protein kinase) 26*	Red ontogenesis	
ppa002217m	2.83	0.001530529	*poltergeist like 4*	Red ontogenesis	
ppa021350m	2.82	0.006434793	*PQ-loop repeat family protein / transmembrane family protein*	Red ontogenesis	
ppa008366m	2.82	0.001504559	*myb domain protein 94*	Red ontogenesis	
ppa021086m	2.82	2.18E-05	*oligopeptide transporter 4*	Red ontogenesis	
ppa001462m	2.82	2.84E-05	*Protein of unknown function (DUF3741)*	Red ontogenesis	
ppa007708m	2.82	0.000928136	*WRKY family transcription factor*	Red ontogenesis	
ppa017667m	2.81	3.78E-05	*Protein kinase superfamily protein*	Red ontogenesis	
ppa011094m	2.81	0.001087565	*RAB GTPase homolog A6A*	Red ontogenesis	
ppa024091m	2.81	5.72E-05	*Leucine-rich receptor-like protein kinase family protein*	Red ontogenesis	
ppa013659m	2.80	1.42E-05	*Dynein light chain type 1 family protein*	Red ontogenesis	
ppa019900m	2.80	0.000495402	*disease resistance family protein / LRR family protein*	Red ontogenesis	
ppa025569m	2.80	0.000487016	*ankyrin repeat family protein*	Red ontogenesis	
ppa007779m	2.80	4.67E-05	*zinc transporter 1 precursor*	Red ontogenesis	
ppa023779m	2.79	0.001170576	*wall associated kinase 3*	Red ontogenesis	
ppa025229m	2.79	0.001765343	*disease resistance protein (TIR-NBS-LRR class), putative*	Red ontogenesis	Biotic Stress / PR-proteins
ppa007888m	2.79	0.022290731	*DNA-binding bromodomain-containing protein*	Red ontogenesis	
ppa014391m	2.79	0.000340743		Red ontogenesis	
ppa021766m	2.79	0.000803821	*VQ motif-containing protein*	Red ontogenesis	
ppa005058m	2.78	0.000316123	*Pyridoxal phosphate (PLP)-dependent transferases superfamily p*	Red ontogenesis	Biotic Stress / Secondary metabolism involved into stress
ppa021319m	2.78	0.002751595	*P-loop containing nucleoside triphosphate hydrolases superfamily*	Red ontogenesis	
ppa006290m	2.78	2.69E-05	*GHMP kinase family protein*	Red ontogenesis	Biotic Stress / Secondary metabolism involved into stress
ppa004574m	2.78	3.32E-06	*dehydroquinate dehydratase, putative / shikimate dehydrogenase*	Red ontogenesis	
ppa000588m	2.78	0.027623442	*chromatin remodeling 31*	Red ontogenesis	
ppa021058m	2.77	0.001778147	*UDP-glucosyl transferase 85A7*	Red ontogenesis	
ppa023932m	2.77	0.004138514	*SEUSS-like 2*	Red ontogenesis	
ppa018600m	2.77	0.011681055	*disease resistance family protein / LRR family protein*	Red ontogenesis	
ppa005140m	2.77	3.35E-05	*MATE efflux family protein*	Red ontogenesis	
ppa008566m	2.77	0.000668221	*WRKY DNA-binding protein 40*	Red ontogenesis	
ppa020804m	2.77	1.12E-05	*Uridine diphosphate glycosyltransferase 74E2*	Red ontogenesis	
ppa025893m	2.76	3.39E-05	*glutamate receptor 1.4*	Red ontogenesis	
ppa006043m	2.76	3.72E-05	*cation exchanger 1*	Red ontogenesis	
ppa016549m	2.76	0.002448636	*prenylated RAB acceptor 1.F4*	Red ontogenesis	
ppa005194m	2.76	0.005191577	*beta-glucosidase 47*	Red ontogenesis	
ppa022506m	2.76	0.008612523	*ubiquitin-conjugating enzyme 10*	Red ontogenesis	
ppa010140m	2.76	0.006460805	*receptor like protein 1*	Red ontogenesis	
ppa018786m	2.75	0.002442184	*Protein kinase family protein with leucine-rich repeat domain*	Red ontogenesis	
ppa020480m	2.75	0.00110291	*Glutamate receptor family protein*	Red ontogenesis	
ppa024612m	2.75	5.11E-05	*UDP-Glycosyltransferase superfamily protein*	Red ontogenesis	
ppa003959m	2.75	1.72E-05	*phosphate transporter 1;7*	Red ontogenesis	
ppa009922m	2.75	0.000460178	*maternal effect embryo arrest 14*	Red ontogenesis	
ppa025793m	2.74	0.000343582	*Leucine-rich repeat protein kinase family protein*	Red ontogenesis	
ppa003732m	2.74	0.001018919	*acyl activating enzyme 5*	Red ontogenesis	
ppa008890m	2.73	1.89E-05	*alpha/beta-Hydrolases superfamily protein*	Red ontogenesis	
ppa012302m	2.73	6.87E-05	*DREB and EAR motif protein 3*	Red ontogenesis	
ppa004030m	2.73	1.58E-05	*plant intracellular ras group-related LRR 4*	Red ontogenesis	
ppa013560m	2.73	0.005538237		Red ontogenesis	
ppa014826m	2.73	0.046504998	*WRKY DNA-binding protein 55*	Red ontogenesis	
ppa023486m	2.72	0.001090387	*disease resistance protein (TIR-NBS-LRR class), putative*	Red ontogenesis	Biotic Stress / PR-proteins
ppa026938m	2.72	0.000358049	*disease resistance family protein / LRR family protein*	Red ontogenesis	
ppa005782m	2.72	0.00019933	*DHHC-type zinc finger family protein*	Red ontogenesis	
ppa027046m	2.71	0.001641572		Red ontogenesis	
ppa014915m	2.71	0.019925599		Red ontogenesis	
ppa012579m	2.71	1.45E-05	*RING-H2 finger A2A*	Red ontogenesis	
ppa004636m	2.70	0.024951919	*Major facilitator superfamily protein*	Red ontogenesis	
ppa003904m	2.70	0.000389093	*cysteine-rich RLK (RECEPTOR-like protein kinase) 25*	Red ontogenesis	
ppa014082m	2.70	0.032447442		Red ontogenesis	
ppa022941m	2.70	0.000579892	*phloem protein 2-A1*	Red ontogenesis	
ppa004484m	2.70	2.44E-06	*beta glucosidase 11*	Red ontogenesis	
ppa001744m	2.69	0.000176106	*Raffinose synthase family protein*	Red ontogenesis	
ppa002656m	2.68	1.58E-05	*L-aspartate oxidase*	Red ontogenesis	
ppa021076m	2.68	0.000133661	*terpene synthase 21*	Red ontogenesis	
ppa025449m	2.68	0.000146038	*receptor like protein 3*	Red ontogenesis	
ppb024748m	2.67	0.046934771	*Arabidopsis defensin-like protein*	Red ontogenesis	
ppa026181m	2.67	3.84E-05	*glutathione S-transferase TAU 8*	Red ontogenesis	Biotic Stress / Glutathione-S-Transferase
ppa017457m	2.67	0.038758308	*glutamate receptor 2*	Red ontogenesis	
ppa008532m	2.67	2.83E-05	*RmlC-like cupins superfamily protein*	Red ontogenesis	
ppa025575m	2.66	0.000372962	*Protein kinase superfamily protein*	Red ontogenesis	
ppa025305m	2.66	0.000824146	*lysine decarboxylase family protein*	Red ontogenesis	
ppa015004m	2.66	5.72E-05	*Leucine-rich receptor-like protein kinase family protein*	Red ontogenesis	
ppa018253m	2.66	0.000483784	*Protein kinase superfamily protein*	Red ontogenesis	
ppa024222m	2.66	2.69E-05	*RNA-binding (RRM/RBD/RNP motifs) family protein*	Red ontogenesis	
ppa022795m	2.66	1.56E-05	*UDP-Glycosyltransferase superfamily protein*	Red ontogenesis	
ppa004691m	2.65	0.002709102	*AAA-ATPase 1*	Red ontogenesis	
ppa004208m	2.65	0.000528377	*bidirectional amino acid transporter 1*	Red ontogenesis	
ppa008037m	2.65	0.000248426	*choline kinase 1*	Red ontogenesis	

ppa016649m	2.65	0.003335608	receptor like protein 21	Red ontogenesis	
ppa005384m	2.65	0.000190543	Regulator of chromosome condensation (RCC1) family protein	Red ontogenesis	
ppa011060m	2.64	0.01563522	salt tolerance zinc finger	Red ontogenesis	
ppa026790m	2.64	0.02909084	receptor like protein 56	Red ontogenesis	
ppa023588m	2.64	0.002768969	alpha/beta-Hydrolases superfamily protein	Red ontogenesis	
ppa1027191m	2.64	0.012252783	pathogenesis-related gene 1	Red ontogenesis	
ppa019611m	2.64	7.04E-05	Leucine-rich receptor-like protein kinase family protein	Red ontogenesis	
ppa018428m	2.64	0.00022142	glutathione S-transferase TAU 19	Red ontogenesis	Biotic Stress / Glutathione-S-Transferase
ppa017449m	2.63	0.001009877		Red ontogenesis	
ppa017069m	2.63	0.000150521	Leucine-rich receptor-like protein kinase family protein	Red ontogenesis	
ppa017182m	2.63	9.51E-06	Zinc-binding dehydrogenase family protein	Red ontogenesis	
ppa011717m	2.63	0.003563237		Red ontogenesis	
ppa005864m	2.63	0.005655952	Domain of unknown function (DUF966)	Red ontogenesis	
ppa022087m	2.63	1.45E-05	Calcium-dependent phosphotriesterase superfamily protein	Red ontogenesis	Biotic Stress / Secondary metabolism involved into stress
ppa010313m	2.63	0.000703084	NAD(P)-binding Rossmann-fold superfamily protein	Red ontogenesis	
ppa012484m	2.63	2.50E-05	Calcium-dependent lipid-binding (CaLB domain) family protein	Red ontogenesis	
ppa024377m	2.62	0.007228792	LRR and NB-ARC domains-containing disease resistance protein	Red ontogenesis	
ppa016609m	2.62	0.000157207	Leucine-rich receptor-like protein kinase family protein	Red ontogenesis	
ppa015480m	2.62	0.009699853	WRKY DNA-binding protein 51	Red ontogenesis	
ppa004328m	2.62	0.000158832	Cytochrome P450 superfamily protein	Red ontogenesis	
ppa010307m	2.62	4.98E-05	Aluminium induced protein with YGL and LRDR motifs	Red ontogenesis	
ppa019571m	2.61	2.69E-05	Protein kinase family protein with leucine-rich repeat domain	Red ontogenesis	
ppa000380m	2.61	7.19E-05	ATPase E1-E2 type family protein / haloacid dehalogenase-like h	Red ontogenesis	
ppa013815m	2.61	0.035571199		Red ontogenesis	
ppa011839m	2.61	7.66E-05		Red ontogenesis	
ppa017714m	2.61	0.024359334	Protein of unknown function (DUF677)	Red ontogenesis	
ppa021789m	2.61	0.029776299	Protein of unknown function (DUF3133)	Red ontogenesis	
ppb001660m	2.60	1.70E-05	receptor kinase 3	Red ontogenesis	
ppa024563m	2.60	0.00045639	glutamate receptor 2.7	Red ontogenesis	
ppa014211m	2.60	0.040921025		Red ontogenesis	
ppb018185m	2.60	0.006560626	cyclic nucleotide gated channel 1	Red ontogenesis	
ppa007227m	2.59	0.008762016	matrix metalloproteinase	Red ontogenesis	
ppa003986m	2.59	6.28E-06	phosphofructokinase 4	Red ontogenesis	
ppa004036m	2.58	5.52E-06	aldehyde dehydrogenase 2B4	Red ontogenesis	
ppb009860m	2.58	0.001519386		Red ontogenesis	
ppa010367m	2.58	0.002100948	tonoplast intrinsic protein 1;3	Red ontogenesis	
ppb021269m	2.58	0.002368165		Red ontogenesis	
ppa003574m	2.58	0.000787837	WRKY family transcription factor	Red ontogenesis	
ppa021712m	2.58	0.000134336	disease resistance family protein / LRR family protein	Red ontogenesis	
ppa020110m	2.58	4.66E-05	PR5-like receptor kinase	Red ontogenesis	
ppa022410m	2.58	0.00822422		Red ontogenesis	
ppa022503m	2.57	0.00135479	receptor serine/threonine kinase, putative	Red ontogenesis	
ppa022563m	2.57	0.03457255		Red ontogenesis	
ppa006015m	2.57	6.03E-05	UDP-Glycosyltransferase superfamily protein	Red ontogenesis	
ppa022940m	2.57	0.001432742	disease resistance protein (TIR-NBS-LRR class), putative	Red ontogenesis	Biotic Stress / PR-proteins
ppb021358m	2.57	0.009251946	UDP-glucosyl transferase 85A3	Red ontogenesis	
ppa016869m	2.57	0.008134612	disease resistance family protein / LRR family protein	Red ontogenesis	
ppa005211m	2.57	0.000175287	Ankyrin repeat family protein	Red ontogenesis	
ppa027095m	2.56	0.015807075	Aminotransferase-like, plant mobile domain family protein	Red ontogenesis	
ppa017829m	2.56	0.018778467	jasmonic acid carboxyl methyltransferase	Red ontogenesis	
ppa024274m	2.56	4.98E-05	detoxifying efflux carrier 35	Red ontogenesis	
ppa016146m	2.56	0.011142447	S-locus lectin protein kinase family protein	Red ontogenesis	
ppa024396m	2.56	0.002635649	D-mannose binding lectin protein with Apple-like carbohydrate-bir	Red ontogenesis	
ppa022623m	2.56	4.50E-05	glutamate receptor 2.8	Red ontogenesis	
ppa018673m	2.56	0.020063141		Red ontogenesis	
ppa003890m	2.56	0.001048588	phospholipase C 2	Red ontogenesis	
ppa012426m	2.56	0.000383872	Calcium-binding EF-hand family protein	Red ontogenesis	
ppa007656m	2.55	0.006611377	beta HLH protein 93	Red ontogenesis	
ppa026411m	2.55	4.06E-05	Plant protein of unknown function (DUF247)	Red ontogenesis	
ppa018066m	2.55	0.001731763	S-locus lectin protein kinase family protein	Red ontogenesis	
ppa018557m	2.54	0.000385224	UDP-glucosyl transferase 71B6	Red ontogenesis	
ppa010207m	2.54	0.000143486	alanine-tRNA ligases;nucleic acid binding;ligases, forming aminoa	Red ontogenesis	
ppa012300m	2.54	0.001066518	Ribulose bisphosphate carboxylase (small chain) family protein	Red ontogenesis	
ppa008311m	2.54	0.000198062	basic region/leucine zipper motif 60	Red ontogenesis	
ppa011274m	2.54	0.001928427	Protein of unknown function (DUF1442)	Red ontogenesis	
ppa000577m	2.54	0.013044912	Disease resistance protein (TIR-NBS-LRR class) family	Red ontogenesis	Biotic Stress / PR-proteins
ppa016514m	2.53	0.001504559	beta HLH protein 93	Red ontogenesis	
ppa027060m	2.53	0.005938087	disease resistance family protein / LRR family protein	Red ontogenesis	
ppa1027192m	2.53	0.000210495	12-oxophytodienoate reductase 2	Red ontogenesis	
ppa021289m	2.53	0.004335268	HCO3- transporter family	Red ontogenesis	
ppa019971m	2.53	0.037590262	global transcription factor group B1	Red ontogenesis	
ppa016634m	2.53	0.00044163	disease resistance protein (TIR-NBS-LRR class), putative	Red ontogenesis	Biotic Stress / PR-proteins
ppa005994m	2.53	0.003726714	brassinosteroid-6-oxidase 1	Red ontogenesis	
ppa012471m	2.53	0.00741948	Uncharacterised conserved protein UCP031279	Red ontogenesis	
ppa001233m	2.53	3.14E-06	aminopeptidase M1	Red ontogenesis	
ppa007414m	2.53	0.000426716	Glycosyl hydrolase family protein with chitinase insertion domain	Red ontogenesis	
ppa011611m	2.53	0.000175301	serine-rich protein-related	Red ontogenesis	
ppa016100m	2.52	0.015164521	Leucine-rich receptor-like protein kinase family protein	Red ontogenesis	
ppa005836m	2.52	2.99E-05	Peptidase M20/M25/M40 family protein	Red ontogenesis	
ppa002342m	2.52	0.000358066	receptor lectin kinase	Red ontogenesis	
ppa017150m	2.52	0.00488075	Wall-associated kinase family protein	Red ontogenesis	
ppa016270m	2.52	8.01E-05	cationic amino acid transporter 5	Red ontogenesis	
ppa001921m	2.52	5.43E-06	quinolinate synthase	Red ontogenesis	
ppa012770m	2.52	0.000174638	Chaperone DnaJ-domain superfamily protein	Red ontogenesis	
ppa013976m	2.52	0.00043537		Red ontogenesis	
ppa026893m	2.52	0.001518472		Red ontogenesis	
ppa004487m	2.51	0.001848303	MATE efflux family protein	Red ontogenesis	
ppa020866m	2.51	0.00287954	embryo defective 2170	Red ontogenesis	
ppa024400m	2.51	0.000101103	detoxifying efflux carrier 35	Red ontogenesis	
ppa006701m	2.50	8.87E-06	ureide permease 2	Red ontogenesis	
ppa003812m	2.50	9.63E-05	chloroplast beta-amylase	Red ontogenesis	
ppa013730m	2.50	0.000683154		Red ontogenesis	
ppa021699m	2.50	0.049972237	Ankyrin repeat family protein	Red ontogenesis	
ppa007748m	2.50	0.000245443	Peroxidase superfamily protein	Red ontogenesis	
ppa013388m	2.50	0.000804066	Heavy metal transport/detoxification superfamily protein	Red ontogenesis	
ppa015797m	2.49	0.001661823	disease resistance family protein / LRR family protein	Red ontogenesis	
ppa008795m	2.49	0.001320296	alpha/beta-Hydrolases superfamily protein	Red ontogenesis	
ppa025196m	2.49	1.28E-05	Cytochrome P450 superfamily protein	Red ontogenesis	
ppa024660m	2.49	7.46E-05	Cytochrome P450 superfamily protein	Red ontogenesis	
ppa006974m	2.49	0.000378633	ABI five binding protein 2	Red ontogenesis	
ppa027017m	2.49	1.56E-05	RING/FYVE/PHD zinc finger superfamily protein	Red ontogenesis	
ppa020532m	2.48	0.000162949		Red ontogenesis	
ppa020863m	2.48	0.002564105	Leucine-rich receptor-like protein kinase family protein	Red ontogenesis	
ppa009956m	2.48	6.18E-05	RING/FYVE/PHD zinc finger superfamily protein	Red ontogenesis	
ppa008939m	2.48	0.000415686	TARGET OF MONOPTEROS 6	Red ontogenesis	
ppa004265m	2.48	4.98E-05	Eukaryotic aspartyl protease family protein	Red ontogenesis	
ppa006528m	2.47	1.41E-05	Phosphatidic acid phosphatase (PAP2) family protein	Red ontogenesis	
ppa021944m	2.47	0.041294407		Red ontogenesis	
ppa007432m	2.47	0.00941812	Zinc finger C-x8-C-x5-C-x3-H type family protein	Red ontogenesis	
ppa017781m	2.47	7.71E-05	Leucine-rich receptor-like protein kinase family protein	Red ontogenesis	
ppa014062m	2.47	0.045016653	Zinc-binding ribosomal protein family protein	Red ontogenesis	
ppa013258m	2.46	0.018065432		Red ontogenesis	
ppa007964m	2.46	0.005529898	Protein of unknown function (DUF761)	Red ontogenesis	
ppa012549m	2.46	0.000202263		Red ontogenesis	
ppa019006m	2.45	0.004879596	UDP-Glycosyltransferase superfamily protein	Red ontogenesis	
ppa1027137m	2.45	0.002796528	disease resistance protein (TIR-NBS-LRR class), putative	Red ontogenesis	Biotic Stress / PR-proteins
ppa009635m	2.45	0.015721947	methyl esterase 17	Red ontogenesis	
ppa006860m	2.45	0.006853688	Protein of unknown function (DUF1262)	Red ontogenesis	
ppa006347m	2.44	0.000124529	Protein kinase superfamily protein	Red ontogenesis	
ppb016552m	2.44	8.01E-05		Red ontogenesis	
ppa012971m	2.44	0.002227619	Chaperone DnaJ-domain superfamily protein	Red ontogenesis	
ppb008123m	2.43	0.002861963		Red ontogenesis	
ppa002693m	2.43	6.55E-05	receptor serine/threonine kinase, putative	Red ontogenesis	
ppa012949m	2.42	4.67E-05	Late embryogenesis abundant protein	Red ontogenesis	
ppa019874m	2.42	4.69E-05	glutamate receptor 2.7	Red ontogenesis	
ppa010058m	2.42	2.69E-05	Galactose oxidase/kelch repeat superfamily protein	Red ontogenesis	
ppa003241m	2.42	4.00E-05	Protein kinase superfamily protein	Red ontogenesis	
ppa014437m	2.42	0.003888997		Red ontogenesis	
ppa001488m	2.42	0.000781887	receptor like protein 12	Red ontogenesis	
ppa022898m	2.42	0.035064115		Red ontogenesis	
ppb022527m	2.42	0.013855716	PR5-like receptor kinase	Red ontogenesis	
ppb016773m	2.41	0.000316123	pleiotropic drug resistance 11	Red ontogenesis	
ppa025744m	2.41	0.002682066	Protein kinase superfamily protein	Red ontogenesis	
ppa016380m	2.41	0.007769358	UDP-glucosyl transferase 85A5	Red ontogenesis	
ppa010999m	2.41	2.23E-05	Calcium-binding EF-hand family protein	Red ontogenesis	
ppa022145m	2.41	0.00272062		Red ontogenesis	
ppa019819m	2.41	0.003630303	oxidative stress 3	Red ontogenesis	
ppa014976m	2.41	0.008339838	SEC14 cytosolic factor family protein / phosphoglyceride transfer	Red ontogenesis	
ppb011450m	2.41	0.048967362		Red ontogenesis	
ppa013232m	2.41	5.17E-05	cytochrome B5 isoform E	Red ontogenesis	
ppa014998m	2.41	8.90E-06	Disease resistance protein (CC-NBS-LRR class) family	Red ontogenesis	Biotic Stress / PR-proteins
ppa019633m	2.41	0.002183974	UDP-glucosyl transferase 73D1	Red ontogenesis	
ppa013075m	2.40	0.002301578	plant natriuretic peptide A	Red ontogenesis	
ppa018140m	2.40	0.031528058	purine permease 10	Red ontogenesis	
ppa015095m	2.40	0.006874609	C2 calcium/lipid-binding and GRAM domain containing protein	Red ontogenesis	
ppa014217m	2.40	0.029881184		Red ontogenesis	
ppa003466m	2.40	0.000101464	Seven transmembrane MLO family protein	Red ontogenesis	
ppa026759m	2.39	0.000310282	exocyst subunit exo70 family protein E2	Red ontogenesis	

ppa013814m	2.39	0.007449977	senescence-associated gene 21	Red ontogenesis	
ppa022546m	2.39	0.041940425	Acyl-CoA N-acyltransferases (NAT) superfamily protein	Red ontogenesis	
ppa026611m	2.39	0.004061503		Red ontogenesis	
ppa011596m	2.38	0.000932694	high mobility group B2	Red ontogenesis	
ppa009292m	2.38	7.04E-05	NAD(P)-binding Rossmann-fold superfamily protein	Red ontogenesis	
ppa016243m	2.38	0.014338087	receptor like protein 46	Red ontogenesis	
ppa014173m	2.38	0.018697233		Red ontogenesis	
ppa002047m	2.37	0.001277313	wall associated kinase-like 1	Red ontogenesis	
ppa010896m	2.37	0.000601044	Ribosomal protein L34e superfamily protein	Red ontogenesis	
ppa021266m	2.37	0.000210884	ankyrin repeat family protein	Red ontogenesis	
ppa012643m	2.37	0.000279887	eukaryotic elongation factor 5A-1	Red ontogenesis	
ppb013789m	2.37	0.001562905	suppressor of npr1-1 constitutive 4	Red ontogenesis	
ppa022569m	2.36	4.90E-05	MATE efflux family protein	Red ontogenesis	
ppa024815m	2.36	0.047207157	Plant protein of unknown function (DUF936)	Red ontogenesis	
ppa000987m	2.36	0.032166282	calcium ATPase 2	Red ontogenesis	
ppa000501m	2.35	0.000333945	Disease resistance protein (TIR-NBS-LRR class) family	Red ontogenesis	Biotic Stress / PR-proteins
ppa004902m	2.35	0.00192446	alanine:glyoxylate aminotransferase 2	Red ontogenesis	
ppa023692m	2.35	0.001179243	Cytochrome P450 superfamily protein	Red ontogenesis	
ppa001648m	2.35	0.002780149	high affinity K+ transporter 5	Red ontogenesis	
ppa005522m	2.35	0.000283702	COBRA-like protein 1 precursor	Red ontogenesis	
ppa002646m	2.35	0.026848452	heat shock cognate protein 70-1	Red ontogenesis	
ppa008388m	2.35	0.00015663	AT hook motif DNA-binding family protein	Red ontogenesis	
ppa018112m	2.35	0.024318044	glutathione S-transferase TAU 8	Red ontogenesis	Biotic Stress / Glutathione-S-Transferase
ppa018964m	2.35	0.000749137	disease resistance protein (TIR-NBS-LRR class), putative	Red ontogenesis	Biotic Stress / PR-proteins
ppa004917m	2.34	0.00434126		Red ontogenesis	
ppa010154m	2.34	0.007923937	methyl esterase 10	Red ontogenesis	
ppa014999m	2.34	0.001778147	PR5-like receptor kinase	Red ontogenesis	
ppb020966m	2.34	0.00110143	RING/U-box superfamily protein	Red ontogenesis	
ppa007366m	2.34	0.016974468	Protein phosphatase 2C family protein	Red ontogenesis	
ppa005470m	2.34	0.027883188	methionine gamma-lyase	Red ontogenesis	
ppa014145m	2.34	0.022670662		Red ontogenesis	
ppa022516m	2.34	0.000320561	Protein kinase family protein with leucine-rich repeat domain	Red ontogenesis	
ppa014854m	2.34	0.021099014	pinoresinol reductase 1	Red ontogenesis	Secondary Metabolism / Flavonols-Isoflavonoid -Biotic Stress / Secondary metabolism involved into stress
ppa1027147m	2.33	0.00031371	Protein of unknown function (DUF1666)	Red ontogenesis	
ppa008675m	2.33	7.30E-05	BES1/BZR1 homolog 4	Red ontogenesis	
ppa012855m	2.33	0.003513676	Heavy metal transport/detoxification superfamily protein	Red ontogenesis	
ppa002917m	2.33	4.30E-05	Protein kinase superfamily protein	Red ontogenesis	
ppa020351m	2.33	0.049581392	PIF1 helicase	Red ontogenesis	
ppa003447m	2.33	0.006560626	Auxin-responsive GH3 family protein	Red ontogenesis	
ppb020911m	2.32	0.035487918	2-oxoglutarate (2OG) and Fe(II)-dependent oxygenase superfami	Red ontogenesis	
ppa004972m	2.32	0.00373468	UDP-glucosyl transferase 73B3	Red ontogenesis	
ppa012537m	2.32	0.01121534	Calcium-dependent lipid-binding (CaLB domain) family protein	Red ontogenesis	
ppa005583m	2.32	4.00E-05	Transmembrane amino acid transporter family protein	Red ontogenesis	
ppa023851m	2.32	0.000420243	UDP-glucosyl transferase 85A2	Red ontogenesis	
ppa000853m	2.32	0.005046298	Clp ATPase	Red ontogenesis	
ppa024263m	2.32	0.006446839	Transducin/WD40 repeat-like superfamily protein	Red ontogenesis	
ppa013892m	2.32	0.031574705		Red ontogenesis	
ppa017973m	2.31	0.004550015	DNA binding	Red ontogenesis	
ppa008145m	2.31	0.000160463	NAD(P)-linked oxidoreductase superfamily protein	Red ontogenesis	
ppa009788m	2.31	8.95E-05	non-yellowing 1	Red ontogenesis	
ppa018377m	2.31	0.00871767	multidrug resistance-associated protein 3	Red ontogenesis	
ppa000489m	2.31	0.003424325	disease resistance protein (TIR-NBS-LRR class), putative	Red ontogenesis	Biotic Stress / PR-proteins
ppa013627m	2.30	0.023195149	amino acid permease 7	Red ontogenesis	
ppa017560m	2.30	0.049121533		Red ontogenesis	
ppa014377m	2.30	0.00016099		Red ontogenesis	
ppa001858m	2.30	6.22E-06		Red ontogenesis	
ppa004344m	2.30	6.36E-05	sugar transporter 14	Red ontogenesis	
ppa008854m	2.30	0.002603341	alpha/beta-Hydrolases superfamily protein	Red ontogenesis	
ppa004811m	2.29	0.001515073	alanine:glyoxylate aminotransferase 2	Red ontogenesis	
ppa024774m	2.29	0.000299975	alpha/beta-Hydrolases superfamily protein	Red ontogenesis	
ppa024124m	2.29	1.95E-05	Galactose mutarotase-like superfamily protein	Red ontogenesis	
ppa002818m	2.29	0.000245443	receptor like protein 32	Red ontogenesis	
ppa022015m	2.28	0.000820405	2-oxoglutarate (2OG) and Fe(II)-dependent oxygenase superfami	Red ontogenesis	
ppa019342m	2.28	0.000342298	wall associated kinase-like 1	Red ontogenesis	
ppa008011m	2.27	0.00515885	NAD(P)-binding Rossmann-fold superfamily protein	Red ontogenesis	Secondary Metabolism / Dihydroflavonols -Biotic Stress / Secondary metabolism involved into stress
ppa026180m	2.27	0.010697054	alpha/beta-Hydrolases superfamily protein	Red ontogenesis	
ppa000227m	2.27	0.024951919	pleiotropic drug resistance 12	Red ontogenesis	
ppa025531m	2.27	0.007731458	zinc finger (C3HC4-type RING finger) family protein	Red ontogenesis	
ppa011200m	2.27	0.007074176	nudix hydrolase homolog 15	Red ontogenesis	
ppa021907m	2.27	0.000306608	carboxyesterase 13	Red ontogenesis	
ppa017527m	2.27	0.000341979	NAD(P)-binding Rossmann-fold superfamily protein	Red ontogenesis	Biotic Stress / Secondary metabolism involved into stress
ppa006150m	2.26	3.05E-06	MATE efflux family protein	Red ontogenesis	
ppa010754m	2.26	0.002352664	zinc transporter 11 precursor	Red ontogenesis	
ppa009289m	2.26	0.0012313	RmlC-like cupins superfamily protein	Red ontogenesis	
ppa020157m	2.26	0.000323362	Leucine-rich repeat receptor-like protein kinase family protein	Red ontogenesis	
ppa008174m	2.26	0.000768922	farnesyl diphosphate synthase 1	Red ontogenesis	Biotic Stress / Secondary metabolism involved into stress
ppa011916m	2.26	0.000153486		Red ontogenesis	
ppa020190m	2.25	0.014329842	myb domain protein 15	Red ontogenesis	
ppa004430m	2.24	0.005945362	myo-inositol-1-phosphate synthase 2	Red ontogenesis	
ppb025587m	2.24	0.007675463	receptor like protein 33	Red ontogenesis	
ppa006368m	2.24	1.60E-06	Tyrosine transaminase family protein	Red ontogenesis	
ppa017511m	2.24	0.033996082		Red ontogenesis	
ppa004684m	2.23	0.034805291		Red ontogenesis	
ppa012860m	2.23	0.009506709	Chaperone DnaJ-domain superfamily protein	Red ontogenesis	
ppa025237m	2.23	0.009513435		Red ontogenesis	
ppa014120m	2.23	0.031472863		Red ontogenesis	
ppa015751m	2.23	5.09E-05	UDP-Glycosyltransferase superfamily protein	Red ontogenesis	
ppa021904m	2.23	0.039714412	GDSL-like Lipase/Acylhydrolase superfamily protein	Red ontogenesis	
ppa006584m	2.23	0.000212525	Cation efflux family protein	Red ontogenesis	
ppb021781m	2.23	0.001902666		Red ontogenesis	
ppa012221m	2.23	0.000563544	Nucleic acid-binding, OB-fold-like protein	Red ontogenesis	
ppa025186m	2.22	8.55E-05	Protein kinase superfamily protein	Red ontogenesis	
ppa000382m	2.22	0.000604611	aminophospholipid ATPase 1	Red ontogenesis	
ppa011570m	2.22	0.000858002	AUX/IAA transcriptional regulator family protein	Red ontogenesis	
ppa019687m	2.22	8.78E-05	Leucine-rich receptor-like protein kinase family protein	Red ontogenesis	
ppa006413m	2.22	0.000125277	Auxin efflux carrier family protein	Red ontogenesis	
ppa011784m	2.22	0.0003439	Protein of unknown function (DUF962)	Red ontogenesis	
ppa020103m	2.22	0.008339673	Ribonuclease H-like superfamily protein	Red ontogenesis	
ppa007772m	2.21	2.66E-05	glutamine synthase clone R1	Red ontogenesis	
ppa013537m	2.21	0.001126768	Protein of unknown function (DUF3511)	Red ontogenesis	
ppa012448m	2.21	0.009429834		Red ontogenesis	
ppa002534m	2.21	0.005312477	tolB protein-related	Red ontogenesis	
ppa020318m	2.21	0.000112576	Ribosomal RNA processing Brix domain protein	Red ontogenesis	
ppa018051m	2.21	0.000667324	disease resistance family protein / LRR family protein	Red ontogenesis	
ppa010878m	2.21	0.015626106	Protein of unknown function (DUF1640)	Red ontogenesis	
ppa008664m	2.21	0.00746617	syntaxin of plants 121	Red ontogenesis	
ppa015311m	2.20	0.00253011	NAD(P)-linked oxidoreductase superfamily protein	Red ontogenesis	
ppa025520m	2.20	0.04203366		Red ontogenesis	
ppa025943m	2.20	0.017400184	UDP-glucosyl transferase 71B6	Red ontogenesis	
ppa010301m	2.20	0.000211759	VQ motif-containing protein	Red ontogenesis	
ppa010376m	2.20	0.007644614	expansin-like B1	Red ontogenesis	
ppa016919m	2.19	0.010726646	Leucine-rich receptor-like protein kinase family protein	Red ontogenesis	
ppa025037m	2.19	2.56E-05	phosphate transporter 1;9	Red ontogenesis	
ppa012457m	2.19	0.000119069		Red ontogenesis	
ppa007040m	2.19	0.000370104	NADPH-dependent thioredoxin reductase A	Red ontogenesis	
ppa005716m	2.19	0.001367155	ACT domain repeat 4	Red ontogenesis	
ppa014064m	2.18	0.001868113	Wound-responsive family protein	Red ontogenesis	
ppa014995m	2.18	2.01E-05	S-locus lectin protein kinase family protein	Red ontogenesis	
ppa018416m	2.18	0.000284311		Red ontogenesis	
ppa006160m	2.18	2.09E-05	alpha-amylase-like	Red ontogenesis	
ppa000723m	2.17	0.00460775	ARM repeat superfamily protein	Red ontogenesis	
ppa011617m	2.17	0.000105435	Quinone reductase family protein	Red ontogenesis	
ppa026639m	2.17	0.000256308	phospholipase D beta 1	Red ontogenesis	
ppa010445m	2.17	6.55E-05	PLATZ transcription factor family protein	Red ontogenesis	
ppa017401m	2.17	0.003451006	cytochrome P450, family 706, subfamily A, polypeptide 6	Red ontogenesis	
ppa012685m	2.16	0.012984093		Red ontogenesis	
ppa004113m	2.16	9.81E-06	MATE efflux family protein	Red ontogenesis	
ppa009281m	2.16	0.00045913	NAD(P)-binding Rossmann-fold superfamily protein	Red ontogenesis	
ppa013356m	2.16	0.006405073	photosystem II BY	Red ontogenesis	
ppa002174m	2.15	9.41E-06	Protein kinase superfamily protein	Red ontogenesis	
ppa020684m	2.15	0.000932694	P-loop containing nucleoside triphosphate hydrolases superfamily	Red ontogenesis	
ppa022975m	2.15	0.022321997	MuDR family transposase	Red ontogenesis	
ppa018232m	2.15	1.52E-05	NAD(P)-binding Rossmann-fold superfamily protein	Red ontogenesis	
ppa004097m	2.15	4.05E-05	Actin-like ATPase superfamily protein	Red ontogenesis	
ppa003294m	2.15	0.000888453	Major facilitator superfamily protein	Red ontogenesis	
ppa007064m	2.15	5.72E-05	Protein kinase superfamily protein	Red ontogenesis	
ppa022757m	2.15	0.011682849	Concanavalin A-like lectin protein kinase family protein	Red ontogenesis	
ppa015139m	2.15	0.028702004	Cam interacting protein 111	Red ontogenesis	
ppa018343m	2.14	6.11E-05	UDP-glucosyl transferase 85A2	Red ontogenesis	
ppa023423m	2.14	0.003282573	Leucine-rich repeat protein kinase family protein	Red ontogenesis	
ppa014389m	2.14	0.020941213		Red ontogenesis	
ppa013519m	2.14	0.001373633	lipid transfer protein 1	Red ontogenesis	
ppa015802m	2.14	0.003972632		Red ontogenesis	
ppa008824m	2.14	0.000140213	EamA-like transporter family	Red ontogenesis	
ppa012573m	2.14	0.00015327	MLP-like protein 423	Red ontogenesis	
ppa012904m	2.13	0.000206602		Red ontogenesis	
ppa009274m	2.13	0.001029606	heat shock factor 4	Red ontogenesis	
ppa022519m	2.13	0.004350564	Calcium-binding EF-hand family protein	Red ontogenesis	

ppa013052m	2.13	0.009519564	*pathogenesis-related 4*	Red ontogenesis	
ppa023754m	2.13	0.003061376	*disease resistance family protein / LRR family protein*	Red ontogenesis	
ppa013918m	2.13	0.001642732	*EF hand calcium-binding protein family*	Red ontogenesis	
ppa022572m	2.12	0.023739175	*wall-associated kinase 2*	Red ontogenesis	
ppb011482m	2.12	0.001453454		Red ontogenesis	
ppa020063m	2.12	0.009423108	*receptor kinase 1*	Red ontogenesis	
ppa012801m	2.12	0.003755953	*farnesylated protein 6*	Red ontogenesis	
ppb009520m	2.12	0.002562494	*Leucine-rich repeat (LRR) family protein*	Red ontogenesis	
ppa009500m	2.11	0.002217995	*Protein of unknown function (DUF1645)*	Red ontogenesis	
ppa002246m	2.11	0.001584968	*catalytics*	Red ontogenesis	
ppa000266m	2.11	0.008662013	*P-loop containing nucleoside triphosphate hydrolases superfamily*	Red ontogenesis	
ppa000999m	2.11	0.001009877	*disease resistance family protein / LRR family protein*	Red ontogenesis	
ppa024490m	2.10	0.032268278		Red ontogenesis	
ppa016830m	2.10	0.003306427	*receptor like protein 3*	Red ontogenesis	
ppa015767m	2.10	0.000101464	*receptor like protein 6*	Red ontogenesis	
ppa002116m	2.10	0.018462058	*Copper amine oxidase family protein*	Red ontogenesis	
ppa024787m	2.10	0.005955305	*Leucine-rich receptor-like protein kinase family protein*	Red ontogenesis	
ppa022443m	2.10	0.002933894	*Leucine-rich repeat protein kinase family protein*	Red ontogenesis	
ppa019319m	2.10	0.048199702	*cytochrome P450, family 94, subfamily B, polypeptide 1*	Red ontogenesis	
ppa009662m	2.10	6.86E-05	*O-acetylserine (thiol) lyase (OAS-TL) isoform A1*	Red ontogenesis	
ppa020082m	2.09	0.024143153	*receptor like protein 3*	Red ontogenesis	
ppa023213m	2.09	0.018462351	*cytochrome P450, family 76, subfamily G, polypeptide 1*	Red ontogenesis	
ppa012885m	2.09	0.009196784	*SAUR-like auxin-responsive protein family*	Red ontogenesis	
ppa025081m	2.09	0.003513676	*FAD-binding Berberine family protein*	Red ontogenesis	
ppa026218m	2.09	0.041022803	*S-locus lectin protein kinase family protein*	Red ontogenesis	
ppa000420m	2.09	0.017123331	*ATPase E1-E2 type family protein / haloacid dehalogenase-like h*	Red ontogenesis	
ppa018537m	2.09	0.00207264	*lectin protein kinase family protein*	Red ontogenesis	
ppa006591m	2.09	0.01448549	*2-oxoglutarate-dependent dioxygenase family protein*	Red ontogenesis	
ppa018536m	2.09	0.000750321	*GRAM domain-containing protein / ABA-responsive protein-relate*	Red ontogenesis	
ppa022630m	2.09	0.00287954	*MATE efflux family protein*	Red ontogenesis	
ppa011641m	2.09	0.000175203	*SOUL heme-binding family protein*	Red ontogenesis	
ppa011037m	2.08	0.000144032	*RING/U-box superfamily protein*	Red ontogenesis	
ppa022950m	2.08	0.001540825	*Protein kinase family protein with leucine-rich repeat domain*	Red ontogenesis	
ppa000854m	2.08	0.045735729	*Leucine-rich repeat transmembrane protein kinase*	Red ontogenesis	
ppa004221m	2.08	0.000858002	*pheophytinase*	Red ontogenesis	
ppa001295m	2.08	0.011957252	*trehalose phosphate synthase*	Red ontogenesis	
ppa011699m	2.08	0.010726646	*Plant invertase/pectin methylesterase inhibitor superfamily protein*	Red ontogenesis	
ppa016921m	2.08	0.008554273	*Adenine nucleotide alpha hydrolases-like superfamily protein*	Red ontogenesis	
ppa005043m	2.08	0.000147297	*UDP-glucosyl transferase 85A2*	Red ontogenesis	
ppa004822m	2.08	2.50E-05	*MATE efflux family protein*	Red ontogenesis	
ppa004675m	2.08	1.56E-05	*Calmodulin binding protein-like*	Red ontogenesis	
ppa013296m	2.07	0.003518082		Red ontogenesis	
ppa011488m	2.07	0.000595189	*B-box type zinc finger family protein*	Red ontogenesis	
ppa005777m	2.07	0.001939101	*GroES-like zinc-binding alcohol dehydrogenase family protein*	Red ontogenesis	
ppa002722m	2.07	2.20E-05	*Regulator of Vps4 activity in the MVB pathway protein*	Red ontogenesis	
ppa000939m	2.07	0.000267644	*Leucine-rich repeat transmembrane protein kinase*	Red ontogenesis	
ppa021294m	2.07	0.024826193	*TOXICOS EN LEVADURA 4*	Red ontogenesis	
ppa013758m	2.07	0.017393615	*CLAVATA3/ESR-RELATED 43*	Red ontogenesis	
ppa022131m	2.07	0.003421893	*receptor like protein 25*	Red ontogenesis	
ppa002502m	2.07	0.046353424	*roline-rich extensin-like receptor kinase 4*	Red ontogenesis	
ppa008068m	2.06	1.28E-05	*NAD(P)-linked oxidoreductase superfamily protein*	Red ontogenesis	
ppa015620m	2.06	2.48E-05	*Major facilitator superfamily protein*	Red ontogenesis	
ppa021403m	2.06	0.006981324	*Concanavalin A-like lectin protein kinase family protein*	Red ontogenesis	
ppa011811m	2.06	0.046320517	*PYR1-like 4*	Red ontogenesis	
ppa009794m	2.06	0.002299488	*Phosphoglycerate mutase family protein*	Red ontogenesis	
ppa006697m	2.06	0.024373444	*Protein of unknown function (DUF1645)*	Red ontogenesis	
ppa018971m	2.06	0.04749723	*Ubiquitin carboxyl-terminal hydrolase family protein*	Red ontogenesis	
ppa009751m	2.06	0.002887547	*RING/U-box superfamily protein*	Red ontogenesis	
ppa022492m	2.05	0.000370104	*purine permease 3*	Red ontogenesis	
ppa002702m	2.05	0.000275079	*Protein kinase superfamily protein*	Red ontogenesis	
ppa024441m	2.05	0.002580314	*HXXXD-type acyl-transferase family protein*	Red ontogenesis	Secondary Metabolism / Phenylpropanoids - Biotic Stress / Secondary metabolism involved into stress
ppa017854m	2.05	0.002107456	*PsbQ-like 1*	Red ontogenesis	Photosynthesis / Photosystem II
ppa009452m	2.05	0.002649366	*2-oxoglutarate (2OG) and Fe(II)-dependent oxygenase superfami*	Red ontogenesis	
ppa009881m	2.04	0.000458462	*RING/FYVE/PHD zinc finger superfamily protein*	Red ontogenesis	
ppa022003m	2.04	0.024031862	*alpha/beta-Hydrolases superfamily protein*	Red ontogenesis	
ppa017795m	2.04	0.003289613	*cytochrome P450, family 82, subfamily C, polypeptide 4*	Red ontogenesis	
ppa009883m	2.04	0.001024038	*Ras-related small GTP-binding family protein*	Red ontogenesis	
ppa025536m	2.04	0.003439272	*Leucine-rich receptor-like protein kinase family protein*	Red ontogenesis	
ppa015531m	2.04	0.008701911	*BAK1-interacting receptor-like kinase 1*	Red ontogenesis	
ppa002593m	2.04	9.65E-05	*alpha/beta-Hydrolases superfamily protein*	Red ontogenesis	
ppa014025m	2.04	0.023902916		Red ontogenesis	
ppa016697m	2.04	0.002485899	*Leucine-rich receptor-like protein kinase family protein*	Red ontogenesis	
ppa011049m	2.04	0.002567999	*BAK1-interacting receptor-like kinase 1*	Red ontogenesis	
ppa001691m	2.03	0.002802786	*phosphate 1*	Red ontogenesis	
ppa011266m	2.03	0.003359895	*PLATZ transcription factor family protein*	Red ontogenesis	
ppa022618m	2.03	0.017707643		Red ontogenesis	
ppa021128m	2.03	0.002223624	*glutamate receptor 2.9*	Red ontogenesis	
ppa013751m	2.03	0.005095984	*ILI1 binding bHLH 1*	Red ontogenesis	
ppa016435m	2.03	0.004149193	*disease resistance family protein / LRR family protein*	Red ontogenesis	
ppa001816m	2.03	0.002890697	*S-locus lectin protein kinase family protein*	Red ontogenesis	
ppa013521m	2.02	0.006114654		Red ontogenesis	
ppa026071m	2.02	0.011142447	*FAD/NAD(P)-binding oxidoreductase family protein*	Red ontogenesis	
ppa018523m	2.02	0.015844412		Red ontogenesis	
ppa008079m	2.02	0.038854532	*Esterase/lipase/thioesterase family protein*	Red ontogenesis	
ppa009163m	2.02	0.010892883	*cytidine deaminase 1*	Red ontogenesis	
ppa003663m	2.02	0.005016565	*laccase 14*	Red ontogenesis	Biotic Stress / Secondary metabolism involved into stress
ppa007342m	2.02	0.00259607	*2-oxoglutarate (2OG) and Fe(II)-dependent oxygenase superfami*	Red ontogenesis	
ppa012238m	2.02	0.018318548	*mitochondrial ferredoxin 1*	Red ontogenesis	
ppa015953m	2.02	0.001925616		Red ontogenesis	
ppa009511m	2.01	0.000206602	*secretory carrier 3*	Red ontogenesis	
ppa012228m	2.00	0.026480622	*Protein of unknown function (DUF1637)*	Red ontogenesis	
ppa008731m	2.00	0.001501983	*WRKY DNA-binding protein 23*	Red ontogenesis	
ppa005732m	2.00	0.012673819	*biotin F*	Red ontogenesis	Biotic Stress / Secondary metabolism involved into stress
ppa014481m	2.00	0.005337638		Red ontogenesis	
ppa004833m	2.00	9.65E-05	*sodium/calcium exchanger family protein / calcium-binding EF ha*	Red ontogenesis	
ppa023747m	1.99	0.005227216	*Mnd1 family protein*	Red ontogenesis	
ppa004414m	1.99	0.02759567	*cytochrome P450, family 72, subfamily A, polypeptide 15*	Red ontogenesis	
ppa024616m	1.99	0.002135311		Red ontogenesis	
ppa011059m	1.99	0.002226104	*Adenine nucleotide alpha hydrolases-like superfamily protein*	Red ontogenesis	
ppa012182m	1.99	0.000225047	*nuclear factor Y, subunit B8*	Red ontogenesis	
ppa026953m	1.99	0.025231583	*RHO guanyl-nucleotide exchange factor 12*	Red ontogenesis	
ppa017955m	1.99	0.000426716	*Leucine-rich repeat receptor-like protein kinase family protein*	Red ontogenesis	
ppa001301m	1.99	0.004004965	*trehalose-phosphatase/synthase 9*	Red ontogenesis	
ppa010391m	1.98	0.022142079	*K-box region and MADS-box transcription factor family protein*	Red ontogenesis	
ppa020479m	1.98	0.00901934	*Leucine-rich repeat protein kinase family protein*	Red ontogenesis	
ppa002447m	1.98	0.016983211	*P-loop containing nucleoside triphosphate hydrolases superfamily*	Red ontogenesis	
ppa016300m	1.98	0.014453044	*RING/U-box superfamily protein*	Red ontogenesis	
ppa009128m	1.98	0.023386422		Red ontogenesis	
ppa023769m	1.98	0.017360591	*cold, circadian rhythm, and rna binding 2*	Red ontogenesis	
ppa024333m	1.98	0.025379995	*TTF-type zinc finger protein with HAT dimerisation domain*	Red ontogenesis	
ppa021815m	1.98	0.001826102		Red ontogenesis	
ppa025348m	1.98	0.000100836	*Oxidoreductase, zinc-binding dehydrogenase family protein*	Red ontogenesis	
ppa005067m	1.98	0.000526002	*ferrochelatase 1*	Red ontogenesis	
ppa026338m	1.98	0.005552467	*disease resistance family protein / LRR family protein*	Red ontogenesis	
ppa010772m	1.97	0.004770701	*Rubber elongation factor protein (REF)*	Red ontogenesis	
ppa007381m	1.97	0.001670754	*12-oxophytodienoate reductase 2*	Red ontogenesis	
ppa010102m	1.97	0.00792203	*aluminum sensitive 3*	Red ontogenesis	
ppa008613m	1.97	0.000505519	*WRKY DNA-binding protein 7*	Red ontogenesis	
ppa010700m	1.97	0.007625232	*delta(3), delta(2)-enoyl CoA isomerase 1*	Red ontogenesis	
ppa004444m	1.97	0.01563522	*cytochrome P450, family 94, subfamily B, polypeptide 1*	Red ontogenesis	
ppa024764m	1.97	0.009699298	*NB-ARC domain-containing disease resistance protein*	Red ontogenesis	Biotic Stress / PR-proteins
ppa003310m	1.97	0.000101114	*SCARECROW-like 1*	Red ontogenesis	
ppa005752m	1.96	0.000659111	*peptidase M20/M25/M40 family protein*	Red ontogenesis	
ppa019306m	1.96	0.006993573	*BTB/POZ domain with WD40/YVTN repeat-like protein*	Red ontogenesis	
ppa001216m	1.96	0.033756773	*lipoxygenase 1*	Red ontogenesis	
ppa004398m	1.96	0.012165868	*P-loop containing nucleoside triphosphate hydrolases superfamily*	Red ontogenesis	
ppa023625m	1.96	0.026958308	*Leucine-rich receptor-like protein kinase family protein*	Red ontogenesis	
ppa007619m	1.96	0.012970182	*2-oxoglutarate (2OG) and Fe(II)-dependent oxygenase superfami*	Red ontogenesis	
ppa011123m	1.96	3.72E-05	*AGAMOUS-like 24*	Red ontogenesis	
ppa005018m	1.96	0.000107721	*MATE efflux family protein*	Red ontogenesis	
ppa006178m	1.96	0.000406029	*Histone H3 K4-specific methyltransferase SET7/9 family protein*	Red ontogenesis	
ppa009944m	1.95	0.012463131	*NAD(P)-binding Rossmann-fold superfamily protein*	Red ontogenesis	
ppa015821m	1.95	0.006026341	*FAD-binding Berberine family protein*	Red ontogenesis	
ppa019435m	1.95	0.019267949	*related to AP2 1*	Red ontogenesis	
ppa007869m	1.95	0.00069088	*UDP-glucosyl transferase 71C3*	Red ontogenesis	
ppa006635m	1.95	0.000477255	*FAD/NAD(P)-binding oxidoreductase family protein*	Red ontogenesis	
ppa019660m	1.95	0.021298203	*cytochrome P450, family 706, subfamily A, polypeptide 6*	Red ontogenesis	
ppa007488m	1.95	0.006450328	*12-oxophytodienoate reductase 2*	Red ontogenesis	
ppa010785m	1.95	0.000120698	*Aluminium induced protein with YGL and LRDR motifs*	Red ontogenesis	
ppa017042m	1.95	0.005307548	*multidrug resistance-associated protein 3*	Red ontogenesis	
ppa012756m	1.94	0.002087892	*Calcium-dependent lipid-binding (CaLB domain) family protein*	Red ontogenesis	
ppa009450m	1.94	0.000169895	*small G protein family protein / RhoGAP family protein*	Red ontogenesis	
ppa016232m	1.94	0.007985929	*Disease resistance protein (CC-NBS-LRR class) family*	Red ontogenesis	Biotic Stress / PR-proteins
ppb000367m	1.94	0.005011573	*disease resistance family protein / LRR family protein*	Red ontogenesis	
ppa015357m	1.94	0.001018919	*UDP-glucosyl transferase 85A2*	Red ontogenesis	
ppa007415m	1.94	0.00017465	*DCD (Development and Cell Death) domain protein*	Red ontogenesis	
ppa013170m	1.93	0.002636913		Red ontogenesis	
ppa015238m	1.93	0.002109291	*autoinhibited Ca(2+)-ATPase, isoform 4*	Red ontogenesis	

ppa016790m	1.93	0.049121533	Haloacid dehalogenase-like hydrolase (HAD) superfamily protein	Red ontogenesis	
ppa011729m	1.93	0.000836259	Uncharacterised protein family (UPF0497)	Red ontogenesis	
ppa015834m	1.93	0.00018483	Glycosyl hydrolase family 85	Red ontogenesis	
ppa004913m	1.93	0.002446637	with no lysine (K) kinase 4	Red ontogenesis	
ppb020052m	1.93	0.011145239	BED zinc finger ;hAT family dimerisation domain	Red ontogenesis	
ppa006780m	1.93	0.000107757	NAD(P)-binding Rossmann-fold superfamily protein	Red ontogenesis	
ppa010090m	1.93	0.003964209	erf domain protein 9	Red ontogenesis	
ppa019833m	1.93	0.03382855	Basic-leucine zipper (bZIP) transcription factor family protein	Red ontogenesis	
ppa019195m	1.93	0.030446513	Leucine-rich receptor-like protein kinase family protein	Red ontogenesis	
ppa009791m	1.92	0.001571179	methyltransferases	Red ontogenesis	
ppa008875m	1.92	3.95E-05	alpha/beta-Hydrolases superfamily protein	Red ontogenesis	
ppa006429m	1.92	0.002076676	Phosphoribosyltransferase family protein	Red ontogenesis	
ppa001309m	1.92	0.035139296	P-loop containing nucleoside triphosphate hydrolases superfamily	Red ontogenesis	
ppa013522m	1.92	0.002311078	Ubiquitin family protein	Red ontogenesis	
ppa017679m	1.92	0.000593051	Ribosomal RNA processing Brix domain protein	Red ontogenesis	
ppa011723m	1.92	0.000756367	Protein of unknown function (DUF962)	Red ontogenesis	
ppa006789m	1.92	9.46E-05	NAD(P)-binding Rossmann-fold superfamily protein	Red ontogenesis	
ppa013131m	1.92	0.006091864	RHO guanyl-nucleotide exchange factor 11	Red ontogenesis	
ppa006863m	1.92	0.018165789	magnesium transporter 9	Red ontogenesis	
ppa024835m	1.92	0.005798951	Disease resistance protein (CC-NBS-LRR class) family	Red ontogenesis	Biotic Stress / PR-proteins
ppa026506m	1.92	0.011622891	UDP-glucosyl transferase 85A3	Red ontogenesis	
ppa021708m	1.92	0.013270557	MLP-like protein 28	Red ontogenesis	
ppa001498m	1.92	0.018152873	Disease resistance protein (CC-NBS-LRR class) family	Red ontogenesis	Biotic Stress / PR-proteins
ppa000235m	1.91	0.000266296	pleiotropic drug resistance 6	Red ontogenesis	
ppa021729m	1.91	0.045484943	Protein of unknown function (DUF594)	Red ontogenesis	
ppa019145m	1.91	0.002932075	UDP-glucosyl transferase 85A3	Red ontogenesis	
ppa006714m	1.91	0.024084025	Protein kinase superfamily protein	Red ontogenesis	
ppa002166m	1.91	0.001601605	SCARECROW-like 14	Red ontogenesis	
ppa013344m	1.91	0.000560234		Red ontogenesis	
ppa002916m	1.91	0.000581219	Protein of unknown function (DUF668)	Red ontogenesis	
ppa016162m	1.91	0.00958753	Disease resistance protein (TIR-NBS-LRR class) family	Red ontogenesis	Biotic Stress / PR-proteins
ppa003853m	1.90	0.003225235	organic cation/carnitine transporter4	Red ontogenesis	
ppa003398m	1.90	0.013969312	Major facilitator superfamily protein	Red ontogenesis	
ppa019179m	1.90	0.03235119	Protein kinase superfamily protein	Red ontogenesis	
ppa011826m	1.90	0.000125763	ureidoglycolate hydrolases	Red ontogenesis	
ppa000948m	1.90	3.52E-05	ABC1 family protein	Red ontogenesis	
ppa001655m	1.90	0.006396961	pumilio 12	Red ontogenesis	
ppa018576m	1.90	0.02048492	AAA-ATPase 1	Red ontogenesis	
ppa015608m	1.90	0.000458462	receptor kinase 3	Red ontogenesis	
ppa007938m	1.90	0.000336192	F-box and associated interaction domains-containing protein	Red ontogenesis	
ppa019271m	1.90	0.002027511	Leucine-rich repeat receptor-like protein kinase family protein	Red ontogenesis	
ppa013781m	1.90	0.00065183	Histone superfamily protein	Red ontogenesis	
ppa010835m	1.89	0.023386422	Protein of unknown function (DUF1264)	Red ontogenesis	
ppa017048m	1.89	0.00176375	GDSL lipase 1	Red ontogenesis	
ppa005199m	1.89	0.000245555	Granulin repeat cysteine protease family protein	Red ontogenesis	
ppa017031m	1.89	0.009760216	disease resistance family protein / LRR family protein	Red ontogenesis	
ppa003315m	1.89	0.001073764	nitrite reductase 1	Red ontogenesis	
ppa009255m	1.89	0.000994442	Transducin/WD40 repeat-like superfamily protein	Red ontogenesis	
ppa016048m	1.89	0.005531465	voltage dependent anion channel 2	Red ontogenesis	
ppa002159m	1.88	0.006691776	Concanavalin A-like lectin protein kinase family protein	Red ontogenesis	
ppa025583m	1.88	0.003073867	HSP20-like chaperones superfamily protein	Red ontogenesis	
ppa007329m	1.88	5.72E-06	Peptidase S24/S26A/S26B/S26C family protein	Red ontogenesis	
ppa001724m	1.88	0.00902778	K+ uptake permease 6	Red ontogenesis	
ppa004069m	1.88	0.000119836	calcium-dependent protein kinase 19	Red ontogenesis	
ppa019028m	1.88	8.92E-05	Protein kinase superfamily protein	Red ontogenesis	
ppa005622m	1.87	0.00059992	Eukaryotic aspartyl protease family protein	Red ontogenesis	
ppa003952m	1.87	0.025689684	Glutamyl-tRNA reductase family protein	Red ontogenesis	
ppb025621m	1.87	0.045735729	cold, circadian rhythm, and RNA binding 1	Red ontogenesis	
ppa025511m	1.87	0.00741948	receptor like protein 47	Red ontogenesis	
ppa022624m	1.87	0.037198865	methyltransferases	Red ontogenesis	
ppa021362m	1.87	0.01525989	Toll-Interleukin-Resistance (TIR) domain family protein	Red ontogenesis	
ppa002472m	1.87	0.00160097	SCARECROW-like 14	Red ontogenesis	
ppa011011m	1.87	0.001571188	Cysteine proteinases superfamily protein	Red ontogenesis	
ppa017004m	1.87	0.009384491	disease resistance protein (TIR-NBS-LRR class), putative	Red ontogenesis	Biotic Stress / PR-proteins
ppa005649m	1.86	0.000111759	tubulin alpha-3	Red ontogenesis	
ppa008434m	1.86	0.000220764		Red ontogenesis	
ppa003333m	1.86	0.017229772	WRKY DNA-binding protein 33	Red ontogenesis	
ppa025207m	1.86	0.008340829	disease resistance family protein / LRR family protein	Red ontogenesis	
ppa018003m	1.86	0.034017951	NB-ARC domain-containing disease resistance protein	Red ontogenesis	
ppa009625m	1.86	1.26E-05	SPFH/Band 7/PHB domain-containing membrane-associated pro	Red ontogenesis	
ppa013511m	1.86	0.000232778		Red ontogenesis	
ppa001956m	1.86	0.001191498	Subtilase family protein	Red ontogenesis	
ppa015468m	1.86	0.002370479	winged-helix DNA-binding transcription factor family protein	Red ontogenesis	
ppa025075m	1.86	0.038974432	Eukaryotic aspartyl protease family protein	Red ontogenesis	
ppa023530m	1.86	0.010678469	2-oxoglutarate (2OG) and Fe(II)-dependent oxygenase superfami	Red ontogenesis	
ppb016885m	1.86	0.045009061		Red ontogenesis	
ppa013857m	1.86	0.016705531	Low temperature and salt responsive protein family	Red ontogenesis	
ppa020396m	1.86	0.035809213		Red ontogenesis	
ppa001808m	1.86	4.96E-05	S-locus lectin protein kinase family protein	Red ontogenesis	
ppa006708m	1.85	0.000312111	Mitochondrial substrate carrier family protein	Red ontogenesis	
ppa006491m	1.85	0.030618106	Heavy metal transport/detoxification superfamily protein	Red ontogenesis	
ppa011663m	1.85	7.57E-05	cold-regulated 413-plasma membrane 2	Red ontogenesis	
ppa017807m	1.85	0.009636392	FAD-dependent oxidoreductase family protein	Red ontogenesis	
ppa020663m	1.85	0.034782036		Red ontogenesis	
ppa018517m	1.85	0.034044665		Red ontogenesis	
ppa002407m	1.85	0.02263169	Restriction endonuclease, type II-like superfamily protein	Red ontogenesis	
ppa014789m	1.85	0.00083851	SCARECROW-like 14	Red ontogenesis	
ppa012612m	1.85	0.025113001	Calcium-binding EF-hand family protein	Red ontogenesis	
ppa011272m	1.85	0.000256308	Late embryogenesis abundant (LEA) hydroxyproline-rich glycopro	Red ontogenesis	
ppa007076m	1.84	0.000833877	TCV-interacting protein	Red ontogenesis	
ppa001142m	1.84	0.009300155	Leucine-rich repeat receptor-like protein kinase family protein	Red ontogenesis	
ppa006723m	1.84	0.009999022	Core-2/I-branching beta-1,6-N-acetylglucosaminyltransferase fam	Red ontogenesis	
ppa004947m	1.84	0.005663891	cytochrome P450, family 704, subfamily A, polypeptide 2	Red ontogenesis	
ppa006733m	1.84	0.003225437	Protein phosphatase 2C family protein	Red ontogenesis	
ppa012075m	1.84	0.000683386	RING/U-box superfamily protein	Red ontogenesis	
ppa025473m	1.84	0.001903794	transmembrane receptors;ATP binding	Red ontogenesis	Biotic Stress / PR-proteins
ppa021492m	1.83	0.00221811	Inositol 1,3,4-trisphosphate 5/6-kinase family protein	Red ontogenesis	
ppa013446m	1.83	0.03517258		Red ontogenesis	
ppa004578m	1.83	0.021787571	Ankyrin repeat family protein / BTB/POZ domain-containing prote	Red ontogenesis	
ppa024448m	1.83	0.0128009	receptor like protein 7	Red ontogenesis	
ppa020776m	1.83	0.019987345		Red ontogenesis	
ppa008495m	1.83	0.003469795	homeobox 1	Red ontogenesis	
ppa003568m	1.83	0.000226341	phosphatidate cytidylyltransferase family protein	Red ontogenesis	
ppa021494m	1.83	0.039233087		Red ontogenesis	
ppa006216m	1.82	0.015683459	zinc ion binding	Red ontogenesis	
ppa025873m	1.82	0.003981241	MA3 domain-containing protein	Red ontogenesis	
ppa013408m	1.82	0.03380377		Red ontogenesis	
ppa003339m	1.82	0.000128986	peptide transporter 2	Red ontogenesis	
ppa015129m	1.82	0.000280201	receptor like protein 7	Red ontogenesis	
ppa011682m	1.82	0.004415508	glutathione peroxidase 3	Red ontogenesis	
ppa002410m	1.82	0.006366497	RNI-like superfamily protein	Red ontogenesis	
ppa009672m	1.82	0.000102849	Protein phosphatase 2C family protein	Red ontogenesis	
ppa008144m	1.81	0.000377123	NAD(P)-linked oxidoreductase superfamily protein	Red ontogenesis	
ppb015328m	1.81	0.001376944	HXXXD-type acyl-transferase family protein	Red ontogenesis	Secondary Metabolism / Phenylpropanoids - Biotic Stress / Secondary metabolism involved into stress
ppa023976m	1.81	0.003247629	Major facilitator superfamily protein	Red ontogenesis	
ppa020563m	1.81	0.000212162	cold, circadian rhythm, and rna binding 2	Red ontogenesis	
ppa007498m	1.81	0.004041074	12-oxophytodienoate reductase 2	Red ontogenesis	
ppa024050m	1.81	0.047256619	Calcium-binding EF-hand family protein	Red ontogenesis	
ppa019310m	1.80	0.024297817	Peptidase C78, ubiquitin fold modifier-specific peptidase 1/ 2	Red ontogenesis	
ppa011429m	1.80	0.015868298	Tetraspanin family protein	Red ontogenesis	
ppa012533m	1.80	0.008115496		Red ontogenesis	
ppa005883m	1.80	0.00128137	Peptidase M20/M25/M40 family protein	Red ontogenesis	
ppa006890m	1.80	0.000781133	Protein kinase superfamily protein	Red ontogenesis	
ppa011747m	1.80	0.000505438	Chlorophyll A-B binding family protein	Red ontogenesis	
ppa004927m	1.80	0.011278142		Red ontogenesis	
ppa001217m	1.80	0.00470148	Protein kinase superfamily protein	Red ontogenesis	
ppa020772m	1.80	0.001987283	Disease resistance protein (TIR-NBS-LRR class) family	Red ontogenesis	Biotic Stress / PR-proteins
ppa002701m	1.79	0.001427282	SCARECROW-like 14	Red ontogenesis	
ppa006964m	1.79	0.000163921	DHBP synthase RibB-like alpha/beta domain;GTP cyclohydrolase	Red ontogenesis	
ppa017944m	1.79	0.043150716	Disease resistance protein (TIR-NBS-LRR class) family	Red ontogenesis	Biotic Stress / PR-proteins
ppa003413m	1.79	0.002648564	MAC/Perforin domain-containing protein	Red ontogenesis	
ppa013609m	1.79	0.009945546	Dormancy/auxin associated family protein	Red ontogenesis	
ppa012340m	1.79	0.005357772	nudix hydrolase homolog 25	Red ontogenesis	
ppa004374m	1.79	0.00253011	P-loop containing nucleoside triphosphate hydrolases superfamily	Red ontogenesis	
ppa008786m	1.78	0.005701619	homolog of bacterial PANC	Red ontogenesis	
ppa002516m	1.78	0.0021978	cysteine-rich RLK (RECEPTOR-like protein kinase) 25	Red ontogenesis	
ppa012987m	1.78	0.011622891	Cystathionine beta-synthase (CBS) family protein	Red ontogenesis	
ppa008915m	1.78	0.000533768	Integrase-type DNA-binding superfamily protein	Red ontogenesis	
ppa009526m	1.78	0.000256976	NAD(P)-binding Rossmann-fold superfamily protein	Red ontogenesis	
ppa004260m	1.78	0.030888205	ferulic acid 5-hydroxylase 1	Red ontogenesis	
ppa012059m	1.77	0.024056205	maternal effect embryo arrest 59	Red ontogenesis	
ppa023857m	1.77	0.023403995	GroES-like zinc-binding dehydrogenase family protein	Red ontogenesis	
ppa001461m	1.77	0.000536921	ADR1-like 1	Red ontogenesis	Biotic Stress / PR-proteins
ppa005800m	1.77	2.84E-05	Transducin family protein / WD-40 repeat family protein	Red ontogenesis	
ppa022409m	1.77	0.025231583	elicitor-activated gene 3-1	Red ontogenesis	Secondary Metabolism / Phenylpropanoids - Biotic Stress / Secondary metabolism involved into stress
ppa005713m	1.77	0.024595406	B-box type zinc finger protein with CCT domain	Red ontogenesis	
ppa020377m	1.77	0.049192844	Leucine-rich repeat protein kinase family protein	Red ontogenesis	
ppa012497m	1.77	0.008394699		Red ontogenesis	

ppa007462m	1.77	0.000153486	Aldolase-type TIM barrel family protein	Red ontogenesis	
ppa013095m	1.77	0.045250579		Red ontogenesis	
ppa004179m	1.77	0.000624357	cytochrome P450, family 82, subfamily C, polypeptide 4	Red ontogenesis	
ppa001059m	1.77	0.004484808		Red ontogenesis	
ppa021242m	1.76	0.013005975	Ribosomal L18p/L5e family protein	Red ontogenesis	
ppa007377m	1.76	0.000438671	12-oxophytodienoate reductase 2	Red ontogenesis	
ppa006247m	1.76	0.003270332	homolog of yeast CDT1 A	Red ontogenesis	
ppa022271m	1.76	0.024177789	disease resistance family protein / LRR family protein	Red ontogenesis	
ppa005016m	1.76	0.000265333	digalactosyl diacylglycerol deficient 2	Red ontogenesis	
ppa003929m	1.76	0.004818442	Transmembrane amino acid transporter family protein	Red ontogenesis	
ppa012391m	1.76	0.021273724	ubiquitin-conjugating enzyme 32	Red ontogenesis	
ppa007265m	1.76	0.000897724	2-oxoglutarate (2OG) and Fe(II)-dependent oxygenase superfami	Red ontogenesis	
ppa025680m	1.76	0.007638726	Peptidase M20/M25/M40 family protein	Red ontogenesis	
ppa008071m	1.75	0.004004965	pyrimidin 4	Red ontogenesis	
ppa010020m	1.75	0.000108948		Red ontogenesis	
ppa006102m	1.75	0.029541619	P-loop containing nucleoside triphosphate hydrolases superfamily	Red ontogenesis	
ppa020502m	1.75	0.000171336	cold, circadian rhythm, and rna binding 2	Red ontogenesis	
ppa013632m	1.75	0.040451408	Tautomerase/MIF superfamily protein	Red ontogenesis	
ppa004281m	1.75	0.000121394	cytochrome P450, family 706, subfamily A, polypeptide 4	Red ontogenesis	
ppa026886m	1.75	0.018413468	Leucine-rich receptor-like protein kinase family protein	Red ontogenesis	
ppa011087m	1.75	0.001025642	glutathione S-transferase tau 7	Red ontogenesis	Biotic Stress / Glutathione-S-Transferase
ppa007260m	1.75	0.003924839	GroES-like zinc-binding dehydrogenase family protein	Red ontogenesis	
ppa010145m	1.75	0.007023844	2-oxoglutarate (2OG) and Fe(II)-dependent oxygenase superfami	Red ontogenesis	
ppa004624m	1.75	1.58E-05	MATE efflux family protein	Red ontogenesis	
ppa026046m	1.75	0.048883937	related to ABI3/VP1 2	Red ontogenesis	
ppa024883m	1.74	0.04672582	Vacuolar iron transporter (VIT) family protein	Red ontogenesis	
ppa014150m	1.74	0.026396521		Red ontogenesis	
ppa012858m	1.74	0.005701619	Bifunctional inhibitor/lipid-transfer protein/seed storage 2S albumi	Red ontogenesis	
ppa024775m	1.74	0.041793383	Protein of unknown function (DUF707)	Red ontogenesis	
ppa006458m	1.74	0.02460003	glutamate dehydrogenase 2	Red ontogenesis	
ppa006453m	1.74	0.005033974	monogalactosyldiacylglycerol synthase 2	Red ontogenesis	
ppa013678m	1.74	0.0361322	Ribosomal protein L18e/L15 superfamily protein	Red ontogenesis	
ppa004159m	1.74	0.013570107	Arabidopsis thaliana protein of unknown function (DUF821)	Red ontogenesis	
ppa004606m	1.74	0.019002376	cytochrome P450, family 76, subfamily C, polypeptide 4	Red ontogenesis	
ppa009343m	1.74	0.006736238		Red ontogenesis	
ppb020315m	1.73	0.036844983	S-adenosyl-L-methionine-dependent methyltransferases superfar	Red ontogenesis	
ppa002575m	1.73	0.005595402	hydroxyproline-rich glycoprotein family protein	Red ontogenesis	
ppa002122m	1.73	0.001024886	oligopeptide transporter 7	Red ontogenesis	
ppa002872m	1.73	0.002474435	protein kinase family protein / peptidoglycan-binding LysM domai	Red ontogenesis	
ppa022802m	1.73	0.005191319	Integrase-type DNA-binding superfamily protein	Red ontogenesis	
ppa001527m	1.73	0.001971945	cation/H+ exchanger 18	Red ontogenesis	
ppa001952m	1.73	0.000326782	cellulose synthase like E1	Red ontogenesis	
ppa017300m	1.73	0.034162395	cytochrome P450, family 82, subfamily C, polypeptide 4	Red ontogenesis	
ppa018082m	1.73	0.023848051	cytochrome P450, family 705, subfamily A, polypeptide 3	Red ontogenesis	
ppa005315m	1.73	0.000894473	aspartate aminotransferase 3	Red ontogenesis	
ppa006420m	1.72	0.000431063	squalene synthase 1	Red ontogenesis	
ppa007698m	1.72	0.004493066	KNOTTED1-like homeobox gene 3	Red ontogenesis	
ppa003247m	1.72	0.00375264	senescence-associated gene 101	Red ontogenesis	
ppa008971m	1.72	0.005900417	ribonuclease P family protein	Red ontogenesis	
ppa001535m	1.72	0.014241187	sucrose synthase 4	Red ontogenesis	
ppa008142m	1.72	0.000321922	Zinc-binding dehydrogenase family protein	Red ontogenesis	
ppa011973m	1.72	0.000404072	HVA22-like protein K	Red ontogenesis	
ppa007825m	1.72	0.00010647	Peroxisomal membrane 22 kDa (Mpv17/PMP22) family protein	Red ontogenesis	
ppa004301m	1.72	0.000860438	cytochrome P450, family 82, subfamily C, polypeptide 4	Red ontogenesis	
ppa008837m	1.72	0.019153079	Homeodomain-like superfamily protein	Red ontogenesis	
ppa019782m	1.72	0.002563637	Leucine-rich receptor-like protein kinase family protein	Red ontogenesis	
ppa002847m	1.71	0.011596069	Plant neutral invertase family protein	Red ontogenesis	
ppa013983m	1.71	0.000879131	Calcium-binding EF-hand family protein	Red ontogenesis	
ppa007393m	1.71	0.005358359	RING/U-box superfamily protein	Red ontogenesis	
ppa004837m	1.71	0.000471342	MATE efflux family protein	Red ontogenesis	
ppa014424m	1.71	0.037945902		Red ontogenesis	
ppa019950m	1.71	0.006453766	glutamate receptor 2.8	Red ontogenesis	
ppa009234m	1.71	0.000278951	Leucine-rich repeat (LRR) family protein	Red ontogenesis	
ppa010062m	1.70	0.001318083	proliferating cell nuclear antigen 2	Red ontogenesis	
ppa011444m	1.70	0.017276081	pyrophosphorylase 1	Red ontogenesis	
ppa019968m	1.70	0.004514741	chitin elicitor receptor kinase 1	Red ontogenesis	
ppa015760m	1.70	0.027418849	Concanavalin A-like lectin protein kinase family protein	Red ontogenesis	
ppa006338m	1.70	0.006232448	UDP-Glycosyltransferase superfamily protein	Red ontogenesis	
ppb010436m	1.70	0.02993586	topoisomerase 3alpha	Red ontogenesis	
ppa016354m	1.70	0.009196784		Red ontogenesis	
ppa006138m	1.70	0.00020087	SNF1-related protein kinase regulatory subunit gamma 1	Red ontogenesis	
ppa002786m	1.70	0.000384898	Leucine-rich repeat protein kinase family protein	Red ontogenesis	
ppa002696m	1.69	4.08E-05	MIRO-related GTP-ase 2	Red ontogenesis	
ppa026739m	1.69	0.018240351	cold, circadian rhythm, and RNA binding 1	Red ontogenesis	
ppa008905m	1.69	0.013712969	NAD(P)-linked oxidoreductase superfamily protein	Red ontogenesis	
ppa013342m	1.69	0.012522088	C-terminal cysteine residue is changed to a serine 1	Red ontogenesis	
ppb010285m	1.69	0.00414528		Red ontogenesis	
ppa022930m	1.69	0.001504559	cold, circadian rhythm, and rna binding 2	Red ontogenesis	
ppa013593m	1.69	0.000673338	RPM1-interacting protein 4 (RIN4) family protein	Red ontogenesis	
ppa005608m	1.69	0.000226042	G-protein coupled receptors;GTPase activators	Red ontogenesis	
ppa003950m	1.69	0.020613771	cytochrome P450, family 711, subfamily A, polypeptide 1	Red ontogenesis	
ppa001460m	1.69	0.002417653	Protein of unknown function (DUF1666)	Red ontogenesis	
ppa000828m	1.68	0.020213823	disease resistance protein (TIR-NBS-LRR class)	Red ontogenesis	Biotic Stress / PR-proteins
ppa012070m	1.68	0.002410264	calmodulin 5	Red ontogenesis	
ppa020926m	1.68	0.043685512	disease resistance protein (TIR-NBS-LRR class), putative	Red ontogenesis	Biotic Stress / PR-proteins
ppa005666m	1.68	0.000826671	Transmembrane amino acid transporter family protein	Red ontogenesis	
ppa001518m	1.68	0.000888113	U-box domain-containing protein kinase family protein	Red ontogenesis	
ppa006179m	1.68	0.000102272	alpha-galactosidase 1	Red ontogenesis	
ppa005891m	1.68	0.000182493	RING/U-box superfamily protein	Red ontogenesis	
ppa013749m	1.68	0.001849063	G-protein gamma subunit 2	Red ontogenesis	
ppa020606m	1.67	0.037198865	AGD2-like defense response protein 1	Red ontogenesis	
ppa025495m	1.67	0.035617343	cytokinin response factor 4	Red ontogenesis	
ppa008326m	1.67	0.013811487	UDP-Glycosyltransferase superfamily protein	Red ontogenesis	
ppa004158m	1.67	0.00087935	Ankyrin repeat family protein	Red ontogenesis	
ppa007976m	1.67	0.006488051	Lactate/malate dehydrogenase family protein	Red ontogenesis	
ppa017510m	1.67	0.003564926	Protein kinase superfamily protein	Red ontogenesis	
ppa012965m	1.67	5.23E-05		Red ontogenesis	
ppa010250m	1.67	0.029408289		Red ontogenesis	
ppa013462m	1.67	0.001096167		Red ontogenesis	
ppa006881m	1.66	0.001167397	zinc finger (C3HC4-type RING finger) family protein	Red ontogenesis	
ppa015538m	1.66	0.001945843	mitogen-activated protein kinase phosphatase 1	Red ontogenesis	
ppa013226m	1.66	0.004418333		Red ontogenesis	
ppa005221m	1.66	0.000354716	high affinity nitrate transporter 2.7	Red ontogenesis	
ppa013551m	1.66	0.000416019	membrane-anchored ubiquitin-fold protein 1 precursor	Red ontogenesis	
ppa011746m	1.66	0.002207808		Red ontogenesis	
ppa005112m	1.66	0.043232069	UDP-Glycosyltransferase superfamily protein	Red ontogenesis	
ppa022632m	1.65	0.003333222	receptor like protein 34	Red ontogenesis	
ppa026966m	1.65	0.013887752	Ribosomal RNA processing Brix domain protein	Red ontogenesis	
ppa004175m	1.65	0.000835318	cytochrome P450, family 82, subfamily C, polypeptide 4	Red ontogenesis	
ppa006407m	1.65	0.000750321	ARF-GAP domain 5	Red ontogenesis	
ppa004673m	1.65	0.000413819	glutathione-disulfide reductase	Red ontogenesis	
ppa005560m	1.64	0.00652606	Transmembrane amino acid transporter family protein	Red ontogenesis	
ppa006442m	1.64	3.51E-05	glutamate dehydrogenase 2	Red ontogenesis	
ppa014450m	1.64	0.004004965		Red ontogenesis	
ppa022847m	1.64	0.000426716	Seven transmembrane MLO family protein	Red ontogenesis	
ppa022151m	1.64	0.037716573	Protein kinase superfamily protein	Red ontogenesis	
ppa008968m	1.64	0.013265469	CRT (chloroquine-resistance transporter)-like transporter 2	Red ontogenesis	
ppa000416m	1.64	0.031228057	DNA helicase (RECQl4A)	Red ontogenesis	
ppa003747m	1.64	0.002463081	4-coumarate:CoA ligase 2	Red ontogenesis	Secondary Metabolism / Phenylpropanoids - Biotic Stress / Secondary metabolism involved into stress
ppa010766m	1.64	8.78E-05	alpha/beta-Hydrolases superfamily protein	Red ontogenesis	
ppa012329m	1.63	0.000347445	transcription initiation factor IIA gamma chain / TFIIA-gamma (TFI	Red ontogenesis	
ppa007516m	1.63	0.000312737	Protein kinase superfamily protein	Red ontogenesis	
ppa006431m	1.63	0.000203684	PHD finger protein-related	Red ontogenesis	
ppa025004m	1.63	0.042490029	cold, circadian rhythm, and rna binding 2	Red ontogenesis	
ppa006222m	1.63	0.000267819	Major facilitator superfamily protein	Red ontogenesis	
ppa002071m	1.63	0.001887896	cyclic nucleotide gated channel 1	Red ontogenesis	
ppa010389m	1.63	0.005702813	RING membrane-anchor 1	Red ontogenesis	
ppa013009m	1.63	0.007071212		Red ontogenesis	
ppa019885m	1.62	0.009734462	basic transcription factor 3	Red ontogenesis	
ppa008647m	1.62	5.52E-05	Plant protein 1589 of unknown function	Red ontogenesis	
ppa007078m	1.62	0.005621327	Calcineurin-like metallo-phosphoesterase superfamily protein	Red ontogenesis	
ppa009218m	1.62	0.000728814	P-loop containing nucleoside triphosphate hydrolases superfamily	Red ontogenesis	
ppa006933m	1.62	0.002381794	aminoalcoholphosphotransferase 1	Red ontogenesis	
ppa004860m	1.62	0.000966222	6-phosphogluconate dehydrogenase family protein	Red ontogenesis	
ppa004845m	1.62	0.030014205	ammonium transporter 2	Red ontogenesis	
ppa009011m	1.62	0.019994485	F-box family protein	Red ontogenesis	
ppa023572m	1.62	0.005726743	Protein of unknown function (DUF1218)	Red ontogenesis	
ppa009906m	1.62	0.006347417	NAD(P)-binding Rossmann-fold superfamily protein	Red ontogenesis	
ppa018966m	1.61	0.001744774	lectin protein kinase family protein	Red ontogenesis	
ppa009153m	1.61	0.005290953	Haloacid dehalogenase-like hydrolase (HAD) superfamily protein	Red ontogenesis	
ppa005670m	1.61	0.006434793	Protein kinase superfamily protein	Red ontogenesis	
ppa007450m	1.61	0.022815905	2-oxoglutarate (2OG) and Fe(II)-dependent oxygenase superfami	Red ontogenesis	
ppa009036m	1.61	0.013774179		Red ontogenesis	
ppa007710m	1.60	0.000877705	Protein kinase superfamily protein	Red ontogenesis	
ppa004116m	1.60	0.001335299	beta-amylase 3	Red ontogenesis	
ppa003311m	1.60	0.018152606	RADIATION SENSITIVE 17	Red ontogenesis	
ppa022895m	1.60	0.047089179		Red ontogenesis	
ppa012480m	1.60	0.001191498	Calcium-dependent lipid-binding (CaLB domain) family protein	Red ontogenesis	

ppa026676m	1.60	0.005530508	*ATPase, F1 complex, gamma subunit protein*	Red ontogenesis	Photosynthesis / Redox chain
ppa011925m	1.60	0.012484154	*ubiquitin-conjugating enzyme19*	Red ontogenesis	
ppa003136m	1.60	0.000476179	*auxin response factor 17*	Red ontogenesis	
ppa003424m	1.60	0.025197531	*O-fucosyltransferase family protein*	Red ontogenesis	
ppa014887m	1.59	0.037233621	*disease resistance protein (TIR-NBS-LRR class), putative*	Red ontogenesis	Biotic Stress / PR-proteins
ppa002508m	1.59	0.008277044	*glutaredoxin-related*	Red ontogenesis	
ppa011755m	1.59	0.002865817	*AUX/IAA transcriptional regulator family protein*	Red ontogenesis	
ppa008165m	1.59	0.000683459	*pfkB-like carbohydrate kinase family protein*	Red ontogenesis	
ppa012947m	1.59	0.01258318	*cytochrome P450, family 76, subfamily C, polypeptide 1*	Red ontogenesis	
ppa008758m	1.59	0.001702397	*NAD(P)-binding Rossmann-fold superfamily protein*	Red ontogenesis	Secondary Metabolism / Dihydroflavonols -Biotic Stress / Secondary metabolism involved into stress
ppa013418m	1.59	0.039247709		Red ontogenesis	
ppa008181m	1.59	0.009448619	*farnesyl diphosphate synthase 1*	Red ontogenesis	Biotic Stress / Secondary metabolism involved into stress
ppa001372m	1.59	0.001476365	*lectin protein kinase family protein*	Red ontogenesis	
ppa004219m	1.59	0.000820091	*sugar transporter 1*	Red ontogenesis	
ppa004503m	1.59	0.041490395	*cytochrome P450, family 76, subfamily C, polypeptide 4*	Red ontogenesis	
ppa024477m	1.58	0.011366103	*heat shock cognate protein 70-1*	Red ontogenesis	
ppa001539m	1.58	0.001708018	*OSBP(oxysterol binding protein)-related protein 1C*	Red ontogenesis	
ppa009477m	1.58	0.000833877	*phytanoyl-CoA dioxygenase (PhyH) family protein*	Red ontogenesis	
ppa002946m	1.58	0.032818909	*exocyst subunit exo70 family protein H2*	Red ontogenesis	
ppa026889m	1.58	0.006260639	*receptor like protein 7*	Red ontogenesis	
ppa012501m	1.58	0.030446513	*Adenine nucleotide alpha hydrolases-like superfamily protein*	Red ontogenesis	
ppa006513m	1.58	0.001025841	*Thiolase family protein*	Red ontogenesis	Biotic Stress / Secondary metabolism involved into stress
ppa013234m	1.58	0.002521946	*cytochrome B5 isoform E*	Red ontogenesis	
ppa002381m	1.57	0.043585224	*Zinc finger (C3HC4-type RING finger) family protein*	Red ontogenesis	
ppa007348m	1.57	0.000446989	*SPFH/Band 7/PHB domain-containing membrane-associated pro*	Red ontogenesis	
ppa001292m	1.57	0.010292089	*disease resistance family protein / LRR family protein*	Red ontogenesis	
ppa023510m	1.57	0.000151307	*cytochrome P450, family 82, subfamily C, polypeptide 4*	Red ontogenesis	
ppa010530m	1.57	0.003568652	*DNA-binding storekeeper protein-related*	Red ontogenesis	
ppa003158m	1.57	0.001179243	*nitrate transporter 1.5*	Red ontogenesis	
ppa010590m	1.57	0.027979892	*Ankyrin repeat family protein*	Red ontogenesis	
ppa018130m	1.57	0.010479082	*receptor like protein 33*	Red ontogenesis	
ppa004679m	1.57	0.005872425	*Uncharacterised conserved protein UCP031088, alpha/beta hydr*	Red ontogenesis	
ppa014240m	1.57	0.039708463		Red ontogenesis	
ppa010244m	1.57	0.000879131	*VIRB2-interacting protein 2*	Red ontogenesis	
ppa019144m	1.57	0.006581038	*glutamate receptor 2.7*	Red ontogenesis	
ppa004369m	1.57	0.000639159	*Pyridoxal phosphate (PLP)-dependent transferases superfamily p*	Red ontogenesis	
ppa005935m	1.56	0.000228687	*calmodulin-binding receptor-like cytoplasmic kinase 2*	Red ontogenesis	
ppa004127m	1.56	0.000183208	*FAD-binding Berberine family protein*	Red ontogenesis	
ppa008404m	1.56	0.006557724	*homocysteine S-methyltransferase 3*	Red ontogenesis	
ppa015330m	1.56	0.046938312	*beta glucosidase 11*	Red ontogenesis	
ppa014743m	1.56	0.033996082	*PLC-like phosphodiesterases superfamily protein*	Red ontogenesis	
ppa007331m	1.56	0.00468619	*DA1*	Red ontogenesis	
ppa025089m	1.56	0.04240015	*Protein kinase superfamily protein*	Red ontogenesis	
ppa000727m	1.56	0.004292726	*ATPase E1-E2 type family protein / haloacid dehalogenase-like h*	Red ontogenesis	
ppa009476m	1.56	0.000178305	*Protein of unknown function (DUF1295)*	Red ontogenesis	
ppa005392m	1.56	0.023881484	*Tetratricopeptide repeat (TPR)-like superfamily protein*	Red ontogenesis	
ppa024258m	1.56	0.000647573	*Disease resistance protein (TIR-NBS-LRR class) family*	Red ontogenesis	Biotic Stress / PR-proteins
ppa003207m	1.56	0.04080487	*Seven transmembrane MLO family protein*	Red ontogenesis	
ppa008760m	1.55	0.044026034	*peroxisomal adenine nucleotide carrier 1*	Red ontogenesis	
ppa002797m	1.55	0.000133893	*SCARECROW-like 14*	Red ontogenesis	
ppa024137m	1.55	0.013307792	*RING-H2 group F2A*	Red ontogenesis	
ppa004989m	1.55	0.000282346	*UDP-glucosyl transferase 85A3*	Red ontogenesis	
ppa005590m	1.55	0.001798954	*sterol methyltransferase 2*	Red ontogenesis	
ppa009463m	1.55	0.001922219	*SPFH/Band 7/PHB domain-containing membrane-associated pro*	Red ontogenesis	
ppa020231m	1.55	0.014497683	*autoinhibited Ca2+-ATPase 11*	Red ontogenesis	
ppa026287m	1.55	0.021488364	*Fumarylacetoacetate (FAA) hydrolase family*	Red ontogenesis	
ppa023846m	1.54	0.046851546		Red ontogenesis	
ppa008695m	1.54	0.001304006	*non-intrinsic ABC protein 9*	Red ontogenesis	
ppa010854m	1.54	0.037604655	*Protein of unknown function, DUF617*	Red ontogenesis	
ppa022616m	1.54	0.044546552	*Protein of unknown function (DUF1262)*	Red ontogenesis	
ppa016511m	1.54	0.00036418	*AGAMOUS-like 20*	Red ontogenesis	
ppa020416m	1.54	0.023725232	*AGD2-like defense response protein 1*	Red ontogenesis	
ppa022428m	1.54	0.02909084	*DNA replication protein-related*	Red ontogenesis	
ppa006568m	1.54	0.01512486	*FAD-binding Berberine family protein*	Red ontogenesis	
ppa005456m	1.54	0.002580902	*SecY protein transport family protein*	Red ontogenesis	
ppa008293m	1.54	0.000767521	*prohibitin 6*	Red ontogenesis	
ppa018037m	1.53	0.000368625	*cationic amino acid transporter 5*	Red ontogenesis	
ppa013973m	1.53	0.003618409		Red ontogenesis	
ppa022167m	1.53	0.01467637	*Protein kinase family protein with leucine-rich repeat domain*	Red ontogenesis	
ppa012891m	1.53	0.001809844	*Cox19-like CHCH family protein*	Red ontogenesis	
ppa002208m	1.53	0.000402679	*mitogen-activated protein kinase kinase kinase 5*	Red ontogenesis	
ppa004795m	1.53	0.007906114	*beta vacuolar processing enzyme*	Red ontogenesis	
ppa024468m	1.53	0.026304035	*receptor like protein 54*	Red ontogenesis	
ppa002619m	1.53	0.003326798	*WRKY family transcription factor*	Red ontogenesis	
ppa003313m	1.53	0.006175134	*Major facilitator superfamily protein*	Red ontogenesis	
ppa010504m	1.53	7.19E-05		Red ontogenesis	
ppa019630m	1.53	0.00520803	*Malectin/receptor-like protein kinase family protein*	Red ontogenesis	
ppa006144m	1.53	0.001113906	*pyrimidine 1*	Red ontogenesis	
ppa008382m	1.53	0.010896347	*NAD(P)-binding Rossmann-fold superfamily protein*	Red ontogenesis	Secondary Metabolism / Dihydroflavonols -Biotic Stress / Secondary metabolism involved into stress
ppa006243m	1.53	0.049012123	*AP2/B3 transcription factor family protein*	Red ontogenesis	
ppa010607m	1.53	0.002644851	*Sec14p-like phosphatidylinositol transfer family protein*	Red ontogenesis	
ppa021685m	1.52	0.006093773	*Homeodomain-like superfamily protein*	Red ontogenesis	
ppa009683m	1.52	0.001281045	*Protein phosphatase 2C family protein*	Red ontogenesis	
ppa004169m	1.52	0.000363261	*cytochrome P450, family 82, subfamily C, polypeptide 4*	Red ontogenesis	
ppa007490m	1.52	0.001378889	*12-oxophytodienoate reductase 2*	Red ontogenesis	
ppa006468m	1.52	0.00018483	*alpha/beta-Hydrolases superfamily protein*	Red ontogenesis	
ppa001538m	1.52	0.041480849	*CRINKLY4 related 3*	Red ontogenesis	
ppa014435m	1.52	0.002785395	*secE/sec61-gamma protein transport protein*	Red ontogenesis	
ppa007375m	1.51	0.013398025	*ABI five binding protein 3*	Red ontogenesis	
ppa010978m	1.51	0.001561425	*RING/U-box superfamily protein*	Red ontogenesis	
ppa009620m	1.51	0.000244503		Red ontogenesis	
ppa002752m	1.51	0.002372946	*ARP protein (REF)*	Red ontogenesis	
ppa1027190m	1.51	0.029881184	*Disease resistance protein (CC-NBS-LRR class) family*	Red ontogenesis	Biotic Stress / PR-proteins
ppa005700m	1.51	0.00044913	*CBL-interacting protein kinase 9*	Red ontogenesis	
ppa009733m	1.51	0.00823843	*calcium-binding EF hand family protein*	Red ontogenesis	
ppa014227m	1.50	0.005358359		Red ontogenesis	
ppa016076m	1.50	0.001698947	*with no lysine (K) kinase 4*	Red ontogenesis	
ppa017634m	1.50	0.020146108	*S-locus lectin protein kinase family protein*	Red ontogenesis	
ppa026644m	1.50	0.002640641		Red ontogenesis	
ppa000596m	1.50	0.036713303	*Disease resistance protein (TIR-NBS-LRR class) family*	Red ontogenesis	Biotic Stress / PR-proteins
ppa010998m	1.50	0.005757321	*pyrophosphorylase 4*	Red ontogenesis	
ppa011982m	1.50	0.00290479	*CTC-interacting domain 5*	Red ontogenesis	
ppa004582m	1.50	0.010558958	*HSP20-like chaperones superfamily protein*	Red ontogenesis	
ppa006058m	1.50	0.003321409	*Protein phosphatase 2C family protein*	Red ontogenesis	
ppa014159m	1.49	0.022823718	*Preprotein translocase Sec, Sec61-beta subunit protein*	Red ontogenesis	
ppa019430m	1.49	0.000153486	*UDP-glucosyl transferase 78D2*	Red ontogenesis	
ppa005694m	1.49	0.001345759	*Fatty acid/sphingolipid desaturase*	Red ontogenesis	
ppa012883m	1.49	0.005530508	*dehydration-induced protein (ERD15)*	Red ontogenesis	
ppa001842m	1.49	0.000245443	*S-locus lectin protein kinase family protein*	Red ontogenesis	
ppa007292m	1.49	0.00508659	*cysteine synthase C1*	Red ontogenesis	
ppa011430m	1.49	0.045009897	*PLANT CADMIUM RESISTANCE 2*	Red ontogenesis	
ppa021995m	1.49	0.026010336	*glutamate receptor 2.7*	Red ontogenesis	
ppa023243m	1.49	0.024171784		Red ontogenesis	
ppa005916m	1.49	0.004509479	*acyl-CoA oxidase 4*	Red ontogenesis	
ppa019076m	1.48	0.041395297	*Disease resistance protein (TIR-NBS-LRR class) family*	Red ontogenesis	Biotic Stress / PR-proteins
ppa008895m	1.48	0.000279887	*Arabidopsis Inositol phosphorylceramide synthase 1*	Red ontogenesis	
ppa023464m	1.48	0.000536921		Red ontogenesis	
ppa012314m	1.48	0.005921087		Red ontogenesis	
ppa019904m	1.48	0.003872337	*HXXXD-type acyl-transferase family protein*	Red ontogenesis	Secondary metabolism / Anthocyanins - Biotic Stress / Secondary metabolism involved into stress
ppa008642m	1.48	0.017360591	*Peroxidase superfamily protein*	Red ontogenesis	
ppa001267m	1.47	0.00273694	*U-box domain-containing protein kinase family protein*	Red ontogenesis	
ppa026143m	1.47	0.012489382	*cyclic nucleotide gated channel 1*	Red ontogenesis	
ppa009039m	1.47	0.006072249	*seven in absentia of Arabidopsis 2*	Red ontogenesis	
ppa002237m	1.47	0.00252114	*leucine-rich repeat transmembrane protein kinase family protein*	Red ontogenesis	
ppa007245m	1.47	0.00066823	*purine permease 11*	Red ontogenesis	
ppa010552m	1.47	0.000589197	*alpha/beta-Hydrolases superfamily protein*	Red ontogenesis	
ppa003527m	1.47	0.002602532	*Major facilitator superfamily protein*	Red ontogenesis	
ppa012922m	1.47	0.006775164	*calmodulin 5*	Red ontogenesis	
ppa006004m	1.46	0.02650702	*Galactose oxidase/kelch repeat superfamily protein*	Red ontogenesis	
ppa003364m	1.46	0.001540825	*peptide transporter 5*	Red ontogenesis	
ppa004721m	1.46	0.019939882	*Transmembrane amino acid transporter family protein*	Red ontogenesis	
ppa001971m	1.46	0.002301578	*cellulose synthase like G3*	Red ontogenesis	
ppa007308m	1.46	0.001102464		Red ontogenesis	
ppa011080m	1.46	0.049505203	*serine-rich protein-related*	Red ontogenesis	
ppa008606m	1.46	0.000340946	*O-acetylserine (thiol) lyase (OAS-TL) isoform A1*	Red ontogenesis	
ppa012319m	1.46	0.000874503	*Adenine nucleotide alpha hydrolases-like superfamily protein*	Red ontogenesis	
ppa010940m	1.46	0.008277044	*Calcium-binding EF-hand family protein*	Red ontogenesis	
ppa016834m	1.46	0.007600245	*disease resistance family protein / LRR family protein*	Red ontogenesis	
ppa005855m	1.46	0.011000661	*UDP-Glycosyltransferase superfamily protein*	Red ontogenesis	
ppa009774m	1.45	0.002292155	*Protein of unknown function (DUF668)*	Red ontogenesis	
ppa017259m	1.45	0.04058043	*ubiquitin-conjugating enzyme 32*	Red ontogenesis	
ppa004511m	1.45	0.011404254	*polyamine oxidase 1*	Red ontogenesis	
ppa002540m	1.45	0.005989	*AMP-dependent synthetase and ligase family protein*	Red ontogenesis	
ppa013353m	1.45	0.009014552		Red ontogenesis	
ppa007815m	1.45	0.002272858	*Nucleotide/sugar transporter family protein*	Red ontogenesis	
ppa017924m	1.45	0.000781133	*Auxin efflux carrier family protein*	Red ontogenesis	
ppa007226m	1.45	0.005835192	*GDSL lipase 1*	Red ontogenesis	
ppa012030m	1.45	0.000341979	*Peroxisomal membrane 22 kDa (Mpv17/PMP22) family protein*	Red ontogenesis	

ppa000741m	1.45	0.00808502	*Leucine-rich repeat transmembrane protein kinase*	Red ontogenesis	
ppa019450m	1.44	0.000496123	*Transmembrane amino acid transporter family protein*	Red ontogenesis	
ppa013046m	1.44	0.001652187	*basic region/leucine zipper motif 53*	Red ontogenesis	
ppa006035m	1.44	0.000605984	*Protein kinase superfamily protein*	Red ontogenesis	
ppa025712m	1.44	0.005756427	*receptor kinase 3*	Red ontogenesis	
ppa013498m	1.44	0.042910504		Red ontogenesis	
ppa010258m	1.44	0.002080171	*sphingoid base hydroxylase 2*	Red ontogenesis	
ppa005105m	1.44	0.000526002	*Sulfite exporter TauE/SafE family protein*	Red ontogenesis	
ppa010129m	1.44	0.018240019	*RING membrane-anchor 1*	Red ontogenesis	
ppa013060m	1.43	0.003421893		Red ontogenesis	
ppa005488m	1.43	0.003456056	*HXXXD-type acyl-transferase family protein*	Red ontogenesis	Secondary metabolism / Anthocyanins - Biotic Stress / Secondary metabolism involved into stress
ppa004464m	1.43	0.010877228	*cytochrome P450, family 82, subfamily C, polypeptide 4*	Red ontogenesis	
ppa010667m	1.43	0.031127336	*B-cell receptor-associated 31-like*	Red ontogenesis	
ppa024099m	1.43	0.002358587	*cytochrome P450, family 706, subfamily A, polypeptide 4*	Red ontogenesis	
ppa010812m	1.42	0.035617343		Red ontogenesis	
ppa008618m	1.42	0.000563544	*NAD(P)-binding Rossmann-fold superfamily protein*	Red ontogenesis	Secondary Metabolism / Dihydroflavonols -Biotic Stress / Secondary metabolism involved into stress
ppa013634m	1.42	0.046399498		Red ontogenesis	
ppa010192m	1.42	0.005327896	*SGNH hydrolase-type esterase superfamily protein*	Red ontogenesis	
ppa002036m	1.42	0.016353906	*multifunctional protein 2*	Red ontogenesis	
ppa012445m	1.42	0.00023691	*calcium-dependent protein kinase 33*	Red ontogenesis	
ppb015490m	1.42	0.006184756	*UDP-glucosyl transferase 85A2*	Red ontogenesis	
ppa004404m	1.42	0.026149478	*cytochrome P450, family 98, subfamily A, polypeptide 3*	Red ontogenesis	
ppa003195m	1.42	0.000261305	*oligopeptide transporter 5*	Red ontogenesis	
ppa013570m	1.42	0.005537489	*SPIRAL1-like1*	Red ontogenesis	
ppa002201m	1.42	0.005016894	*Major Facilitator Superfamily with SPX (SYG1/Pho81/XPR1) dom*	Red ontogenesis	
ppa014118m	1.42	0.026376329		Red ontogenesis	
ppa021437m	1.42	0.00549515	*Transmembrane amino acid transporter family protein*	Red ontogenesis	
ppa004598m	1.42	0.000758169	*S-adenosyl-L-methionine-dependent methyltransferases superfar*	Red ontogenesis	
ppa003286m	1.41	0.047491399	*NAD(P)H dehydrogenase B3*	Red ontogenesis	
ppa007370m	1.41	0.009423108	*mitogen-activated protein kinase 3*	Red ontogenesis	
ppa010698m	1.41	0.004220836	*indole-3-acetic acid inducible 14*	Red ontogenesis	
ppa008809m	1.41	0.021564588	*IAA-leucine resistant (ILR)-like gene 6*	Red ontogenesis	
ppa009560m	1.41	0.000283261	*NAD(P)-binding Rossmann-fold superfamily protein*	Red ontogenesis	
ppa019188m	1.41	0.00652606	*Protein of unknown function (DUF1262)*	Red ontogenesis	
ppa012766m	1.41	0.036896116		Red ontogenesis	
ppa005756m	1.41	0.003031285	*DNAJ heat shock N-terminal domain-containing protein*	Red ontogenesis	
ppa004261m	1.41	0.040734329	*cytochrome P450, family 71, subfamily A, polypeptide 20*	Red ontogenesis	
ppa019013m	1.41	0.005123188	*alpha/beta-Hydrolases superfamily protein*	Red ontogenesis	
ppa008094m	1.41	0.002917714	*NAD(P)-linked oxidoreductase superfamily protein*	Red ontogenesis	
ppa006924m	1.41	0.002207808	*Cysteine proteinases superfamily protein*	Red ontogenesis	
ppa003698m	1.41	0.017389145	*Major facilitator superfamily protein*	Red ontogenesis	
ppa000418m	1.41	0.002902581	*ATPase E1-E2 type family protein / haloacid dehalogenase-like h*	Red ontogenesis	
ppa012038m	1.41	0.00031371	*Transport protein particle (TRAPP) component*	Red ontogenesis	
ppa008853m	1.40	0.007978	*PLAC8 family protein*	Red ontogenesis	
ppa009876m	1.40	0.00104505	*Ubiquitin system component Cue protein*	Red ontogenesis	
ppa000011m	1.40	0.017471223		Red ontogenesis	
ppa005266m	1.40	0.017044593	*hydroxymethylglutaryl-CoA synthase / HMG-CoA synthase / 3-hy*	Red ontogenesis	Biotic Stress / Secondary metabolism involved into stress
ppa005561m	1.40	0.0358178	*BTB/POZ domain with WD40/YVTN repeat-like protein*	Red ontogenesis	
ppa007784m	1.40	0.00135479	*plant adhesion molecule 1*	Red ontogenesis	
ppa003881m	1.40	0.006143153	*Calcium-dependent lipid-binding (CaLB domain) family protein*	Red ontogenesis	
ppa020513m	1.40	0.042869907	*Tetratricopeptide repeat (TPR)-like superfamily protein*	Red ontogenesis	
ppa009562m	1.39	0.000796936	*Thioredoxin superfamily protein*	Red ontogenesis	
ppa022500m	1.39	0.025002868	*fatty acid desaturase A*	Red ontogenesis	
ppa012424m	1.39	0.010302259	*high mobility group B1*	Red ontogenesis	
ppa005879m	1.39	0.034162395	*Magnesium transporter CorA-like family protein*	Red ontogenesis	
ppa020466m	1.39	0.002419168	*FAD/NAD(P)-binding oxidoreductase family protein*	Red ontogenesis	
ppa012367m	1.38	0.009974491	*SAUR-like auxin-responsive protein family*	Red ontogenesis	
ppa007736m	1.38	0.001311173	*GroES-like zinc-binding alcohol dehydrogenase family protein*	Red ontogenesis	Secondary Metabolism / Phenylpropanoids - Biotic Stress / Secondary metabolism involved into stress
ppa008361m	1.38	0.039842736	*C2H2 and C2HC zinc fingers superfamily protein*	Red ontogenesis	
ppa011254m	1.38	0.002071903	*soluble N-ethylmaleimide-sensitive factor adaptor protein 33*	Red ontogenesis	
ppa009549m	1.38	0.013941944	*unknown seed protein like 1*	Red ontogenesis	
ppa011736m	1.38	0.001276596		Red ontogenesis	
ppa005607m	1.37	0.001039061	*Acetamidase/Formamidase family protein*	Red ontogenesis	
ppa012915m	1.37	0.0019907	*dehydration-induced protein (ERD15)*	Red ontogenesis	
ppa000977m	1.37	0.004898299	*H(+)-ATPase 5*	Red ontogenesis	
ppa013005m	1.37	0.002099365		Red ontogenesis	
ppa012679m	1.37	0.005522857	*Iron-sulphur cluster biosynthesis family protein*	Red ontogenesis	
ppa005137m	1.37	0.001104585	*rubisco activase*	Red ontogenesis	
ppa012152m	1.37	0.043285151	*Pseudouridine synthase family protein*	Red ontogenesis	
ppa018816m	1.37	0.022220053	*nudix hydrolase 1*	Red ontogenesis	
ppa005585m	1.37	0.019492407	*bZIP transcription factor family protein*	Red ontogenesis	
ppa013161m	1.36	0.01565393	*thioredoxin H-type 1*	Red ontogenesis	
ppa008771m	1.36	0.011291281	*Protein kinase superfamily protein*	Red ontogenesis	
ppa012490m	1.36	0.003833599	*Adenine nucleotide alpha hydrolases-like superfamily protein*	Red ontogenesis	
ppa018526m	1.36	0.024998115	*BRI1-like 2*	Red ontogenesis	
ppa013717m	1.36	0.002221109	*cAMP-regulated phosphoprotein 19-related protein*	Red ontogenesis	
ppa010874m	1.36	0.00901934	*Protein of unknown function (DUF167)*	Red ontogenesis	
ppa002849m	1.36	0.008629689	*VACUOLAR SORTING RECEPTOR 7*	Red ontogenesis	
ppa005090m	1.36	0.00034818	*Tetratricopeptide repeat (TPR)-like superfamily protein*	Red ontogenesis	
ppa004086m	1.36	0.009626372	*phosphofructokinase 5*	Red ontogenesis	
ppa001614m	1.36	0.013853915	*MUTS-like protein 4*	Red ontogenesis	
ppa011411m	1.36	0.006569585	*Rer1 family protein*	Red ontogenesis	
ppa022349m	1.35	0.014885285	*receptor like protein 33*	Red ontogenesis	
ppa002102m	1.35	0.047881917	*RELA/SPOT homolog 3*	Red ontogenesis	
ppa007970m	1.35	0.018915195	*2-oxoglutarate (2OG) and Fe(II)-dependent oxygenase superfami*	Red ontogenesis	
ppa005271m	1.35	0.013444121	*Ankyrin repeat family protein*	Red ontogenesis	
ppa006596m	1.35	0.00059992	*PHYTOENE SYNTHASE*	Red ontogenesis	Biotic Stress / Secondary metabolism involved into stress
ppa007294m	1.35	0.001777747	*S-adenosylmethionine decarboxylase*	Red ontogenesis	
ppa005421m	1.35	0.012522088	*peroxisomal 3-ketoacyl-CoA thiolase 3*	Red ontogenesis	
ppa007118m	1.35	0.004377037	*Protein of unknown function (DUF793)*	Red ontogenesis	
ppa010791m	1.35	0.002196997	*N-terminal nucleophile aminohydrolases (Ntn hydrolases) superfa*	Red ontogenesis	
ppa007420m	1.35	0.000917441	*O-methyltransferase family protein*	Red ontogenesis	
ppa025579m	1.35	0.009215449	*FAD-binding Berberine family protein*	Red ontogenesis	
ppa007470m	1.35	0.008340829	*cation exchanger 5*	Red ontogenesis	
ppa010219m	1.35	0.005622832	*2-oxoglutarate (2OG) and Fe(II)-dependent oxygenase superfami*	Red ontogenesis	
ppa000355m	1.35	0.000805373	*multidrug resistance-associated protein 3*	Red ontogenesis	
ppa002515m	1.35	0.005718106	*ABA Overly-Sensitive 5*	Red ontogenesis	
ppa009997m	1.35	0.016479654		Red ontogenesis	
ppa008467m	1.34	0.043576181		Red ontogenesis	
ppa004147m	1.34	0.039390219	*Major facilitator superfamily protein*	Red ontogenesis	
ppa009665m	1.34	0.00066907	*Protein phosphatase 2C family protein*	Red ontogenesis	
ppa011709m	1.34	0.010346794	*Ras-related small GTP-binding family protein*	Red ontogenesis	
ppa006564m	1.34	0.005530508	*Galactose oxidase/kelch repeat superfamily protein*	Red ontogenesis	
ppa007842m	1.34	0.01174803	*ATPase family associated with various cellular activities (AAA)*	Red ontogenesis	
ppa010815m	1.34	0.0065365	*Modifier of rudimentary (Mod(r)) protein*	Red ontogenesis	
ppa011285m	1.34	0.003783533	*Thioredoxin superfamily protein*	Red ontogenesis	
ppa009221m	1.34	0.038491021	*zinc knuckle (CCHC-type) family protein*	Red ontogenesis	
ppa003240m	1.34	0.001946846	*phosphoinositide 4-kinase gamma 4*	Red ontogenesis	
ppa012781m	1.33	0.00652606	*ORMDL family protein*	Red ontogenesis	
ppa004366m	1.33	0.006415682	*Actin cross-linking protein*	Red ontogenesis	
ppa004652m	1.33	0.009693235	*Major facilitator superfamily protein*	Red ontogenesis	
ppa004805m	1.33	0.013609833	*MATE efflux family protein*	Red ontogenesis	
ppa010593m	1.33	0.001310667	*nicotinamidase 1*	Red ontogenesis	Secondary Metabolism / Phenylpropanoids - Biotic Stress / Secondary metabolism involved into stress
ppa019541m	1.33	0.046773617	*Transducin/WD40 repeat-like superfamily protein*	Red ontogenesis	
ppa001911m	1.33	0.003278778	*Leucine-rich repeat protein kinase family protein*	Red ontogenesis	
ppa008494m	1.32	0.034448329	*pfkB-like carbohydrate kinase family protein*	Red ontogenesis	
ppa019352m	1.32	0.002949053	*Malectin/receptor-like protein kinase family protein*	Red ontogenesis	
ppa027079m	1.32	0.012074889	*Transmembrane amino acid transporter family protein*	Red ontogenesis	
ppa004647m	1.32	0.005978564	*inositol transporter 1*	Red ontogenesis	
ppa004821m	1.32	0.000506897	*PAM domain (PCI/PINT associated module) protein*	Red ontogenesis	
ppa009357m	1.32	0.000728491	*Sec14p-like phosphatidylinositol transfer family protein*	Red ontogenesis	
ppa005631m	1.32	0.001028836	*ARABIDOPSIS SERIN PROTEASE*	Red ontogenesis	
ppa019445m	1.32	0.030825265	*glucuronidase 3*	Red ontogenesis	
ppa008981m	1.32	0.01346762		Red ontogenesis	
ppa013341m	1.32	0.03374174		Red ontogenesis	
ppa006945m	1.31	0.000830049	*cinnamoyl coa reductase 1*	Red ontogenesis	Secondary Metabolism / Dihydroflavonols -Biotic Stress / Secondary metabolism involved into stress
ppa000967m	1.31	0.003899249	*ABC2 homolog 7*	Red ontogenesis	
ppa008032m	1.31	0.002780091	*UDP-D-glucose/UDP-D-galactose 4-epimerase 5*	Red ontogenesis	
ppa009035m	1.31	0.001432742	*phosphate transporter 3;3*	Red ontogenesis	
ppa002034m	1.31	0.008340829	*arginine decarboxylase 2*	Red ontogenesis	
ppa004388m	1.31	0.000699019	*Major Facilitator Superfamily with SPX (SYG1/Pho81/XPR1) dom*	Red ontogenesis	
ppa025482m	1.31	0.008250611	*with no lysine (K) kinase 5*	Red ontogenesis	
ppa007186m	1.31	0.021273724	*alcohol dehydrogenase 1*	Red ontogenesis	
ppa005524m	1.31	0.000983507	*Oxysterol-binding family protein*	Red ontogenesis	
ppa009017m	1.31	0.002607187	*RP non-ATPase subunit 8A*	Red ontogenesis	
ppa011218m	1.31	0.003151393	*vesicle-associated membrane protein 726*	Red ontogenesis	
ppa012585m	1.31	0.006917685	*Adenine nucleotide alpha hydrolases-like superfamily protein*	Red ontogenesis	
ppa008394m	1.31	0.003872337	*Phosphoglycerate mutase family protein*	Red ontogenesis	
ppa010806m	1.31	0.004128579	*RING/U-box superfamily protein*	Red ontogenesis	
ppa005977m	1.31	0.006529937	*Melibiase family protein*	Red ontogenesis	
ppa012066m	1.30	0.010889164		Red ontogenesis	
ppa002135m	1.30	0.000622993	*cyclic nucleotide gated channel 1*	Red ontogenesis	
ppa001077m	1.30	0.001886028	*extra-large G-protein 1*	Red ontogenesis	
ppa011371m	1.30	0.006276419	*glutathione S-transferase TAU 19*	Red ontogenesis	Biotic Stress / Glutathione-S-Transferase
ppa005145m	1.30	0.003067948	*Protein of unknown function (DUF1336)*	Red ontogenesis	
ppa002133m	1.30	0.002612672	*Protein kinase superfamily protein*	Red ontogenesis	
ppa017116m	1.30	0.000741265	*NAD(P)-binding Rossmann-fold superfamily protein*	Red ontogenesis	Secondary Metabolism / Dihydroflavonols -Biotic Stress / Secondary metabolism involved into stress
ppa006992m	1.29	0.000672967	*Transcription factor IIA, alpha/beta subunit*	Red ontogenesis	

ppa013495m	1.29	0.00428831	*Protein of unknown function (DUF3511)*	Red ontogenesis	
ppa010525m	1.29	0.017707643	*FKBP-like peptidyl-prolyl cis-trans isomerase family protein*	Red ontogenesis	
ppa007658m	1.29	0.005792662	*carboxyesterase 13*	Red ontogenesis	
ppa011694m	1.29	0.002651642	*Ras-related small GTP-binding family protein*	Red ontogenesis	
ppa014192m	1.29	0.010296452		Red ontogenesis	
ppa005871m	1.29	0.001525776	*proline transporter 1*	Red ontogenesis	
ppa004215m	1.29	0.016672014	*AMP-dependent synthetase and ligase family protein*	Red ontogenesis	
ppa026527m	1.29	0.024359334	*Ubiquitin-like superfamily protein*	Red ontogenesis	
ppa012738m	1.29	0.029953396	*Ribosomal protein L18ae family*	Red ontogenesis	
ppa013090m	1.29	0.023730658	*CP12 domain-containing protein 3*	Red ontogenesis	
ppa007307m	1.29	0.011230012	*isocitrate dehydrogenase 1*	Red ontogenesis	
ppa007867m	1.29	0.04482295	*Surfeit locus 1 cytochrome c oxidase biogenesis protein*	Red ontogenesis	
ppa007482m	1.28	0.010442728	*endoplasmic reticulum oxidoreductins 1*	Red ontogenesis	
ppa004071m	1.28	0.008677731	*magnesium/proton exchanger*	Red ontogenesis	
ppa002415m	1.28	0.026396521	*zinc finger (CCCH-type) family protein*	Red ontogenesis	
ppa013649m	1.28	0.006025484	*Yippee family putative zinc-binding protein*	Red ontogenesis	
ppa011809m	1.28	0.007336989	*peptidemethionine sulfoxide reductase 1*	Red ontogenesis	
ppa007866m	1.28	0.001693435	*strictosidine synthase-like 4*	Red ontogenesis	Biotic Stress / Secondary metabolism involved into stress
ppa010731m	1.28	0.013939101	*homolog of carrot EP3-3 chitinase*	Red ontogenesis	
ppa004658m	1.28	0.019633187	*Major facilitator superfamily protein*	Red ontogenesis	
ppa003545m	1.28	0.016067349	*MAC/Perforin domain-containing protein*	Red ontogenesis	
ppa013617m	1.28	0.022998317		Red ontogenesis	
ppa000737m	1.28	0.009798487	*Protein kinase family protein with leucine-rich repeat domain*	Red ontogenesis	
ppa000648m	1.28	0.00518849	*GTP-binding protein Obg/CgtA*	Red ontogenesis	
ppa009324m	1.28	0.010398149	*short-chain dehydrogenase-reductase B*	Red ontogenesis	
ppa001012m	1.28	0.001753924	*Beta-glucosidase, GBA2 type family protein*	Red ontogenesis	
ppa007090m	1.27	0.00118879	*NAC domain containing protein 28*	Red ontogenesis	
ppa010933m	1.27	0.005056551	*Cysteine/Histidine-rich C1 domain family protein*	Red ontogenesis	
ppa011452m	1.27	0.005801476	*adenylate cyclases*	Red ontogenesis	
ppa012578m	1.27	0.001693966		Red ontogenesis	
ppa002282m	1.27	0.017661776	*acyl-CoA oxidase 2*	Red ontogenesis	
ppa010771m	1.27	0.00205872	*glutathione peroxidase 6*	Red ontogenesis	
ppa009700m	1.26	0.007622713	*WRKY DNA-binding protein 11*	Red ontogenesis	
ppa007082m	1.26	0.000988282	*beta-hydroxyisobutyryl-CoA hydrolase 1*	Red ontogenesis	
ppa009115m	1.26	0.020574896	*Nucleotide-sugar transporter family protein*	Red ontogenesis	
ppa000486m	1.26	0.031254701	*Outer arm dynein light chain 1 protein*	Red ontogenesis	
ppa023897m	1.26	0.005471137	*receptor like protein 6*	Red ontogenesis	
ppa001467m	1.26	0.027783376	*Protein kinase superfamily protein*	Red ontogenesis	
ppa011909m	1.26	0.002920803		Red ontogenesis	
ppa005627m	1.26	0.002226183	*Thioesterase/thiol ester dehydrase-isomerase superfamily protein*	Red ontogenesis	
ppa005642m	1.26	0.001712647	*tubulin alpha-3*	Red ontogenesis	
ppa006628m	1.26	0.002249646	*pfkB-like carbohydrate kinase family protein*	Red ontogenesis	
ppa013101m	1.26	0.04672582		Red ontogenesis	
ppa027158m	1.26	0.007336989	*sodium hydrogen exchanger 2*	Red ontogenesis	
ppa008303m	1.25	0.001101288	*Outward rectifying potassium channel protein*	Red ontogenesis	
ppa015127m	1.25	0.023460324	*UDP-glucosyl transferase 88A1*	Red ontogenesis	
ppb020871m	1.25	0.001322278		Red ontogenesis	
ppa007193m	1.25	0.015942744	*related to AP2 4*	Red ontogenesis	
ppa012972m	1.25	0.024224582	*high mobility group B2*	Red ontogenesis	
ppa005909m	1.25	0.002362066	*Protein of unknown function (DUF1350)*	Red ontogenesis	
ppa006098m	1.25	0.040588179	*Protein kinase superfamily protein*	Red ontogenesis	
ppa003104m	1.25	0.00969067	*ENTH/VHS/GAT family protein*	Red ontogenesis	
ppa011876m	1.25	0.044854879	*zinc finger (AN1-like) family protein*	Red ontogenesis	
ppa013497m	1.25	0.045780571	*Calcium-binding EF-hand family protein*	Red ontogenesis	
ppa013773m	1.25	0.005239047	*Eukaryotic protein of unknown function (DUF872)*	Red ontogenesis	
ppa013935m	1.24	0.01010694	*cystatin B*	Red ontogenesis	
ppa006013m	1.24	0.042199619	*phosphoserine aminotransferase*	Red ontogenesis	
ppa012852m	1.24	0.002087461		Red ontogenesis	
ppa008589m	1.24	0.01587458	*Cysteine proteinases superfamily protein*	Red ontogenesis	
ppa000672m	1.24	0.008444367	*autoinhibited Ca2+-ATPase 11*	Red ontogenesis	
ppa008380m	1.24	0.002047497	*RING/FYVE/PHD zinc finger superfamily protein*	Red ontogenesis	
ppa008712m	1.24	0.002363711	*Oxidoreductase, zinc-binding dehydrogenase family protein*	Red ontogenesis	
ppa013855m	1.24	0.033250969		Red ontogenesis	
ppa010816m	1.24	0.001883636	*Glutathione S-transferase family protein*	Red ontogenesis	Biotic Stress / Glutathione-S-Transferase
ppa006261m	1.24	0.008000346	*S-methyl-5-thioribose kinase*	Red ontogenesis	
ppa012235m	1.24	0.004759189	*glycosyltransferase family protein 28*	Red ontogenesis	
ppa005845m	1.24	0.034492933	*Aldolase-type TIM barrel family protein*	Red ontogenesis	
ppa010938m	1.24	0.004415508	*SNF7 family protein*	Red ontogenesis	
ppa007909m	1.24	0.0093312	*Transducin/WD40 repeat-like superfamily protein*	Red ontogenesis	
ppa007780m	1.23	0.001640863	*senescence-associated family protein*	Red ontogenesis	
ppa007426m	1.23	0.020197563	*potassium channel tetramerisation domain-containing protein / pe*	Red ontogenesis	
ppa011044m	1.23	0.003112045	*F-box/RNI-like superfamily protein*	Red ontogenesis	
ppa013273m	1.23	0.030686295	*Heavy metal transport/detoxification superfamily protein*	Red ontogenesis	
ppa012374m	1.23	0.006505698	*Putative thiol-disulphide oxidoreductase DCC*	Red ontogenesis	
ppa010604m	1.23	0.014176305		Red ontogenesis	
ppa012906m	1.23	0.01210409	*RING/U-box superfamily protein*	Red ontogenesis	
ppa010419m	1.23	0.001336521	*Rubber elongation factor protein (REF)*	Red ontogenesis	
ppa008003m	1.23	0.001893425	*D-aminoacid aminotransferase-like PLP-dependent enzymes sup*	Red ontogenesis	
ppa011537m	1.23	0.048177368		Red ontogenesis	
ppa013719m	1.23	0.035621393		Red ontogenesis	
ppa010353m	1.22	0.015107685		Red ontogenesis	
ppa018778m	1.22	0.0018062	*permease, cytosine/purines, uracil, thiamine, allantoin family prot*	Red ontogenesis	
ppa011237m	1.22	0.010404814	*vacuolar protein sorting-associated protein 20.2*	Red ontogenesis	
ppa008131m	1.22	0.008040548	*Protein of unknown function (DUF803)*	Red ontogenesis	
ppa012116m	1.22	0.04706469		Red ontogenesis	
ppa017651m	1.22	0.024748412		Red ontogenesis	
ppa017165m	1.22	0.001601869	*Nodulin MtN3 family protein*	Red ontogenesis	
ppa005552m	1.22	0.025004595	*hydroxyproline-rich glycoprotein family protein*	Red ontogenesis	
ppa005275m	1.21	0.022427293	*purple acid phosphatase 10*	Red ontogenesis	
ppa013143m	1.21	0.021515486		Red ontogenesis	
ppa003995m	1.21	0.004510317	*FAD/NAD(P)-binding oxidoreductase family protein*	Red ontogenesis	
ppa013097m	1.21	0.037415869	*HVA22 homologue D*	Red ontogenesis	
ppa001161m	1.21	0.016709182	*Leucine-rich repeat transmembrane protein kinase*	Red ontogenesis	
ppa006293m	1.21	0.014459598	*Senescence/dehydration-associated protein-related*	Red ontogenesis	
ppa011605m	1.21	0.001544893	*20S proteasome beta subunit D1*	Red ontogenesis	
ppa007549m	1.20	0.018819163	*Protein of unknown function, DUF593*	Red ontogenesis	
ppa010879m	1.20	0.031383077		Red ontogenesis	
ppa010097m	1.20	0.008883305	*mannose-1-phosphate guanylyltransferase (GDP)s;GDP-galactos*	Red ontogenesis	
ppa026350m	1.20	0.004047017	*Major facilitator superfamily protein*	Red ontogenesis	
ppa005771m	1.20	0.004727616	*Transcription elongation factor (TFIIS) family protein*	Red ontogenesis	
ppa010936m	1.20	0.001760075	*Uncharacterized protein family (UPF0016)*	Red ontogenesis	
ppa004559m	1.20	0.020388685	*heat shock factor 3*	Red ontogenesis	
ppa012726m	1.20	0.016546976	*RNA-binding (RRM/RBD/RNP motifs) family protein*	Red ontogenesis	
ppa008401m	1.20	0.005594858	*Oxidoreductase, zinc-binding dehydrogenase family protein*	Red ontogenesis	
ppa009747m	1.20	0.004977763	*homeobox 1*	Red ontogenesis	
ppa008886m	1.20	0.002599917	*LAG1 longevity assurance homolog 3*	Red ontogenesis	
ppa011297m	1.20	0.005358359	*RAB GTPase homolog A2B*	Red ontogenesis	
ppa011573m	1.19	0.005123188	*membrane-associated progesterone binding protein 3*	Red ontogenesis	
ppa010338m	1.19	0.014666063	*thiamin pyrophosphokinase1*	Red ontogenesis	
ppa013788m	1.19	0.004001946	*Protein of unknown function (DUF581)*	Red ontogenesis	
ppa021703m	1.19	0.011596069	*Disease resistance protein (TIR-NBS-LRR class) family*	Red ontogenesis	Biotic Stress / PR-proteins
ppa011763m	1.19	0.036361326	*Disease resistance protein (TIR-NBS-LRR class) family*	Red ontogenesis	Biotic Stress / PR-proteins
ppa025649m	1.19	0.020136129	*Small nuclear ribonucleoprotein family protein*	Red ontogenesis	
ppa023149m	1.19	0.026177896	*Phosphorylase superfamily protein*	Red ontogenesis	
ppa008281m	1.19	0.016369288	*uridine-ribohydrolase 1*	Red ontogenesis	
ppa009078m	1.19	0.008905605	*S-adenosyl-L-methionine-dependent methyltransferases superfa*	Red ontogenesis	
ppa009114m	1.19	0.005358359	*protein phosphatase 2A-2*	Red ontogenesis	
ppa005796m	1.19	0.002035076	*ACT domain repeat 6*	Red ontogenesis	
ppa026535m	1.19	0.049121533	*Leucine-rich repeat transmembrane protein kinase*	Red ontogenesis	
ppa011892m	1.19	0.004391942	*Protein of unknown function (DUF1685)*	Red ontogenesis	
ppa001095m	1.19	0.02853168	*receptor like protein 6*	Red ontogenesis	
ppa018514m	1.19	0.020941213	*Calcium-binding EF-hand family protein*	Red ontogenesis	
ppa008798m	1.19	0.011435197	*SPX (SYG1/Pho81/XPR1) domain-containing protein*	Red ontogenesis	
ppa010236m	1.18	0.002290447	*Thioredoxin superfamily protein*	Red ontogenesis	
ppa010625m	1.18	0.001982799	*SGNH hydrolase-type esterase superfamily protein*	Red ontogenesis	
ppa011760m	1.18	0.003106083	*RAC-like 3*	Red ontogenesis	
ppa019975m	1.18	0.033628267	*ATP-dependent RNA helicase, putative*	Red ontogenesis	
ppa011412m	1.18	0.002341663	*RAB GTPase homolog B1C*	Red ontogenesis	
ppa007934m	1.18	0.017183892	*succinate dehydrogenase 2-2*	Red ontogenesis	
ppa007595m	1.18	0.010393353	*organic cation/carnitine transporter 2*	Red ontogenesis	
ppa013460m	1.18	0.047271967	*Homeodomain-like superfamily protein*	Red ontogenesis	
ppa014374m	1.18	0.020895669	*ubiquinol-cytochrome C reductase UQCRX/QCR9-like family prot*	Red ontogenesis	
ppa002865m	1.17	0.002381794	*BAK1-interacting receptor-like kinase 1*	Red ontogenesis	
ppa010027m	1.17	0.034782036	*enoyl-CoA hydratase/isomerase A*	Red ontogenesis	
ppa007140m	1.17	0.004209546	*RING/U-box superfamily protein*	Red ontogenesis	
ppa010805m	1.17	0.014516766	*N-terminal nucleophile aminohydrolases (Ntn hydrolases) superfa*	Red ontogenesis	
ppa016837m	1.17	0.00871767	*Protein kinase superfamily protein*	Red ontogenesis	
ppa018820m	1.17	0.007641332	*Leucine-rich repeat protein kinase family protein*	Red ontogenesis	
ppa009963m	1.17	0.022465265	*ribonuclease 2*	Red ontogenesis	
ppa011586m	1.17	0.048049781	*Inosine triphosphate pyrophosphatase family protein*	Red ontogenesis	
ppa011950m	1.17	0.016971396	*Zim17-type zinc finger protein*	Red ontogenesis	
ppa004435m	1.17	0.014763368	*nonsense-mediated mRNA decay NMD3 family protein*	Red ontogenesis	
ppa022273m	1.17	0.018109203	*cation exchanger 2*	Red ontogenesis	
ppa014129m	1.16	0.024124102		Red ontogenesis	
ppa009427m	1.16	0.007176184	*Clathrin light chain protein*	Red ontogenesis	
ppa009380m	1.16	0.035818198	*NAC (No Apical Meristem) domain transcriptional regulator super*	Red ontogenesis	
ppa002477m	1.16	0.002341663	*Small GTP-binding protein*	Red ontogenesis	
ppa020358m	1.16	0.002607187	*UDP-Glycosyltransferase superfamily protein*	Red ontogenesis	

ppa005945m	1.16	0.042509003	VQ motif-containing protein	Red ontogenesis	
ppa004771m	1.16	0.00287954	MATE efflux family protein	Red ontogenesis	
ppa015076m	1.16	0.014929705	Homeodomain-like superfamily protein	Red ontogenesis	
ppa004267m	1.16	0.028945081	AGC (cAMP-dependent, cGMP-dependent and protein kinase C)	Red ontogenesis	
ppa007232m	1.16	0.020050529	root FNR 1	Red ontogenesis	
ppa013486m	1.16	0.004384149		Red ontogenesis	
ppa003278m	1.16	0.037247522	nitrate transporter 1:2	Red ontogenesis	
ppa020524m	1.16	0.043563711	G-box binding factor 3	Red ontogenesis	
ppa013072m	1.16	0.040012717	gamma histone variant H2AX	Red ontogenesis	
ppa012050m	1.15	0.011606676	phosphoprotein phosphatase inhibitors	Red ontogenesis	
ppa003994m	1.15	0.002755783	phosphofructokinase 3	Red ontogenesis	
ppa013595m	1.15	0.042155766	ELF4-like 4	Red ontogenesis	
ppa009309m	1.15	0.017141697	NAD(P)-binding Rossmann-fold superfamily protein	Red ontogenesis	
ppa003817m	1.15	0.003321409	plant glycogenin-like starch initiation protein 6	Red ontogenesis	
ppa015709m	1.15	0.002755783	receptor lectin kinase	Red ontogenesis	
ppa018318m	1.15	0.016015698	F-box family protein with a domain of unknown function (DUF295)	Red ontogenesis	
ppa024391m	1.15	0.028653527	senescence-associated gene 101	Red ontogenesis	
ppa002350m	1.15	0.003763928	Protein kinase superfamily protein	Red ontogenesis	
ppa007706m	1.15	0.005012031	2-oxoglutarate (2OG) and Fe(II)-dependent oxygenase superfami	Red ontogenesis	
ppa013836m	1.15	0.011691213		Red ontogenesis	
ppa009391m	1.15	0.003278778	senescence associated gene 18	Red ontogenesis	
ppa005736m	1.15	0.005530508	mannose-1-phosphate guanylyltransferase (GDP)s;GDP-galactos	Red ontogenesis	
ppa009080m	1.15	0.002818532	uricase / urate oxidase / nodulin 35, putative	Red ontogenesis	
ppa023594m	1.15	0.022217937	Methyltransferase-related protein	Red ontogenesis	
ppa005038m	1.14	0.002870239	basic helix-loop-helix (bHLH) DNA-binding superfamily protein	Red ontogenesis	
ppa008545m	1.14	0.002785395	Calcium-dependent phosphotriesterase superfamily protein	Red ontogenesis	
ppa003509m	1.14	0.008288765	plant U-box 13	Red ontogenesis	
ppa003143m	1.14	0.005313807	D-3-phosphoglycerate dehydrogenase	Red ontogenesis	
ppa005550m	1.14	0.019172523	RNA-binding (RRM/RBD/RNP motifs) family protein	Red ontogenesis	
ppa010334m	1.14	0.00401576	Haloacid dehalogenase-like hydrolase (HAD) superfamily protein	Red ontogenesis	
ppa018821m	1.14	0.03788928	homolog of carrot EP3-3 chitinase	Red ontogenesis	
ppa006181m	1.14	0.023458156	Transmembrane amino acid transporter family protein	Red ontogenesis	
ppa007830m	1.14	0.045396343	MAP kinase kinase 2	Red ontogenesis	
ppa006582m	1.14	0.018896801	alpha/beta-Hydrolases superfamily protein	Red ontogenesis	
ppa002525m	1.13	0.002950607	Leucine-rich repeat protein kinase family protein	Red ontogenesis	
ppa001978m	1.13	0.003837834	cellulose synthase like G3	Red ontogenesis	
ppa003142m	1.13	0.010292089	NADP-malic enzyme 1	Red ontogenesis	
ppa010825m	1.13	0.007299355	FKBP-like peptidyl-prolyl cis-trans isomerase family protein	Red ontogenesis	
ppa004809m	1.13	0.006472324	magnesium transporter 4	Red ontogenesis	
ppa002429m	1.13	0.020941213	Protein kinase family protein	Red ontogenesis	
ppa012003m	1.13	0.005640154	maternal effect embryo arrest 59	Red ontogenesis	
ppa009244m	1.13	0.016420649	binding	Red ontogenesis	
ppa009810m	1.13	0.008602897	DHHC-type zinc finger family protein	Red ontogenesis	
ppa010769m	1.13	0.020574896	HSP20-like chaperones superfamily protein	Red ontogenesis	
ppa007190m	1.13	0.006493946	Protein phosphatase 2C family protein	Red ontogenesis	
ppa010783m	1.13	0.003350194		Red ontogenesis	
ppa014425m	1.13	0.018029462	secE/sec61-gamma protein transport protein	Red ontogenesis	
ppa011166m	1.13	0.004498444	Ribosomal protein L16p/L10e family protein	Red ontogenesis	
ppa010535m	1.13	0.008612958	Protein of unknown function DUF106, transmembrane	Red ontogenesis	
ppa007670m	1.13	0.021721114	F-box and associated interaction domains-containing protein	Red ontogenesis	
ppa007463m	1.13	0.022465019	Mitochondrial substrate carrier family protein	Red ontogenesis	
ppa000477m	1.13	0.036607138	Disease resistance protein (TIR-NBS-LRR class) family	Red ontogenesis	Biotic Stress / PR-proteins
ppa007732m	1.12	0.005359354	Adenosylmethionine decarboxylase family protein	Red ontogenesis	
ppa004883m	1.12	0.003295362	diacylglycerol kinase 7	Red ontogenesis	
ppa013697m	1.12	0.043563711	Preprotein translocase Sec, Sec61-beta subunit protein	Red ontogenesis	
ppa006719m	1.12	0.042910504	phosphate transporter traffic facilitator1	Red ontogenesis	
ppa002524m	1.12	0.004128579	YELLOW STRIPE like 1	Red ontogenesis	
ppa026732m	1.12	0.015069543	cofactor of nitrate reductase and xanthine dehydrogenase 2	Red ontogenesis	
ppa007157m	1.12	0.003606027	protein kinase C-like zinc finger protein	Red ontogenesis	
ppa005333m	1.12	0.003733984	S-methyl-5-thioribose kinase	Red ontogenesis	
ppa002315m	1.12	0.004681267	glutamine-fructose-6-phosphate transaminase (isomerizing)s;sug	Red ontogenesis	
ppa006401m	1.12	0.040256985	metal tolerance protein A2	Red ontogenesis	
ppa014283m	1.12	0.00599895		Red ontogenesis	
ppa009780m	1.12	0.027818047		Red ontogenesis	
ppa002793m	1.12	0.027633525	Plant protein of unknown function (DUF827)	Red ontogenesis	
ppa013767m	1.12	0.042353977		Red ontogenesis	
ppa009184m	1.12	0.013427667	Protein of unknown function (DUF506)	Red ontogenesis	
ppa019886m	1.12	0.005046298	cytochrome P450, family 81, subfamily D, polypeptide 8	Red ontogenesis	
ppa006486m	1.12	0.003847505	U2 snRNP auxilliary factor, large subunit, splicing factor	Red ontogenesis	
ppa009361m	1.11	0.00410398	gamma-soluble NSF attachment protein	Red ontogenesis	
ppa007570m	1.11	0.003688292	Protein kinase superfamily protein	Red ontogenesis	
ppa004250m	1.11	0.019970954	methyltransferases	Red ontogenesis	
ppa013840m	1.11	0.00873959		Red ontogenesis	
ppa007014m	1.11	0.004128579	Protein phosphatase 2C family protein	Red ontogenesis	
ppa011964m	1.11	0.033578962	Ubiquitin-like superfamily protein	Red ontogenesis	
ppa013668m	1.11	0.039169033		Red ontogenesis	
ppa018067m	1.11	0.034683739	Tetratricopeptide repeat (TPR)-like superfamily protein	Red ontogenesis	
ppa012162m	1.11	0.006405073		Red ontogenesis	
ppa005464m	1.11	0.007551596	Transmembrane amino acid transporter family protein	Red ontogenesis	
ppa012337m	1.11	0.006849189		Red ontogenesis	
ppa008549m	1.11	0.025869494	Calcineurin-like metallo-phosphoesterase superfamily protein	Red ontogenesis	
ppa013696m	1.10	0.042640789	Expressed protein	Red ontogenesis	
ppa003943m	1.10	0.004786669	sodium hydrogen exchanger 2	Red ontogenesis	
ppa009596m	1.10	0.00889086	tetraspanin3	Red ontogenesis	
ppa012601m	1.10	0.00748312	Protein-tyrosine phosphatase-like, PTPLA	Red ontogenesis	
ppa008027m	1.10	0.004802031	serine carboxypeptidase-like 42	Red ontogenesis	
ppa006979m	1.10	0.009419557	mevalonate kinase	Red ontogenesis	Biotic Stress / Secondary metabolism involved into stress
ppa012356m	1.10	0.006148139	SufE/NifU family protein	Red ontogenesis	
ppa021785m	1.10	0.039716077	DegP protease 9	Red ontogenesis	
ppa005354m	1.10	0.0211454	SCP1-like small phosphatase 5	Red ontogenesis	
ppa010415m	1.10	0.010097303	EAP30/Vps36 family protein	Red ontogenesis	
ppa005231m	1.10	0.008563093	RING/U-box superfamily protein	Red ontogenesis	
ppa012732m	1.10	0.009056255	SKP1-like 11	Red ontogenesis	
ppa013019m	1.10	0.01214914	RNA polymerase Rpb6	Red ontogenesis	
ppa004259m	1.10	0.006874609	sigma factor E	Red ontogenesis	
ppa002741m	1.09	0.004448475	P-loop containing nucleoside triphosphate hydrolases superfamily	Red ontogenesis	
ppa009612m	1.09	0.004770701	30S ribosomal protein, putative	Red ontogenesis	
ppa011382m	1.09	0.005383419		Red ontogenesis	
ppa012841m	1.09	0.017608068	prefoldin 5	Red ontogenesis	
ppa002376m	1.09	0.007071212	hypothetical protein 1	Red ontogenesis	
ppa013479m	1.09	0.010889164	Rab5-interacting family protein	Red ontogenesis	
ppa009991m	1.09	0.009922968		Red ontogenesis	
ppa004016m	1.09	0.027242939	non-specific phospholipase C1	Red ontogenesis	
ppa014453m	1.09	0.01596433	cytochrome-c oxidases;electron carriers	Red ontogenesis	
ppa004703m	1.09	0.011395518	Pyruvate kinase family protein	Red ontogenesis	
ppa006416m	1.09	0.014150612	BTB and TAZ domain protein 2	Red ontogenesis	
ppa006262m	1.09	0.005239871	protein kinase 2B	Red ontogenesis	
ppa004563m	1.09	0.004650865	aldehyde dehydrogenase 10A8	Red ontogenesis	
ppa006220m	1.09	0.026478283		Red ontogenesis	
ppa014254m	1.09	0.029044236	RPM1-interacting protein 4 (RIN4) family protein	Red ontogenesis	
ppa008235m	1.09	0.006036291	Mitochondrial substrate carrier family protein	Red ontogenesis	
ppa012498m	1.09	0.013619437	Acid phosphatase/vanadium-dependent haloperoxidase-related p	Red ontogenesis	
ppa012565m	1.09	0.016872591	Signal recognition particle, SRP9/SRP14 subunit	Red ontogenesis	
ppa019569m	1.09	0.006172345	receptor lectin kinase	Red ontogenesis	
ppa007520m	1.09	0.004948598	Haloacid dehalogenase-like hydrolase (HAD) superfamily protein	Red ontogenesis	
ppa008918m	1.09	0.00741948	alpha/beta-Hydrolases superfamily protein	Red ontogenesis	
ppa009196m	1.08	0.036576353	2-phosphoglycolate phosphatase 2	Red ontogenesis	
ppa007398m	1.08	0.019217552	Calcium-binding EF-hand family protein	Red ontogenesis	
ppa023872m	1.08	0.006127659	NmrA-like negative transcriptional regulator family protein	Red ontogenesis	Secondary Metabolism / Flavonols-Isoflavonoid -Biotic Stress / Secondary metabolism involved into stress
ppa018251m	1.08	0.02669038	Leucine-rich receptor-like protein kinase family protein	Red ontogenesis	
ppa024127m	1.08	0.028517527	S-locus lectin protein kinase family protein	Red ontogenesis	
ppa007511m	1.08	0.008622349	O-methyltransferase 1	Red ontogenesis	
ppa005187m	1.08	0.007102444	UDP-Glycosyltransferase superfamily protein	Red ontogenesis	
ppa012510m	1.07	0.044489982	Tetratricopeptide repeat (TPR)-like superfamily protein	Red ontogenesis	
ppa005566m	1.07	0.006026341	Major facilitator superfamily protein	Red ontogenesis	
ppa018725m	1.07	0.018765036	F-box and associated interaction domains-containing protein	Red ontogenesis	
ppa005353m	1.07	0.040451408	ATP sulfurylase 1	Red ontogenesis	
ppa013169m	1.07	0.032334788	Cornichon family protein	Red ontogenesis	
ppa006768m	1.07	0.011000661	Target of Myb protein 1	Red ontogenesis	
ppa008245m	1.07	0.005963693	RNA-binding (RRM/RBD/RNP motifs) family protein with retroviru	Red ontogenesis	
ppa003421m	1.07	0.024130196	minichromosome maintenance 9	Red ontogenesis	
ppa002064m	1.07	0.027694647	Protein kinase superfamily protein	Red ontogenesis	
ppa013333m	1.07	0.027127412	sulfiredoxin	Red ontogenesis	
ppa005893m	1.07	0.008625787	Transducin/WD40 repeat-like superfamily protein	Red ontogenesis	
ppa007605m	1.06	0.005778607	cinnamyl alcohol dehydrogenase 6	Red ontogenesis	Secondary Metabolism / Phenylpropanoids - Biotic Stress / Secondary metabolism involved into stress
ppa026824m	1.06	0.012127981	Sec14p-like phosphatidylinositol transfer family protein	Red ontogenesis	
ppa010329m	1.06	0.008635226	RING/U-box superfamily protein	Red ontogenesis	
ppa011419m	1.06	0.005794178	Domain of unknown function (DUF298)	Red ontogenesis	
ppa004665m	1.06	0.034137332	calcium-dependent protein kinase 4	Red ontogenesis	
ppa007079m	1.06	0.027710839	Glycosyl hydrolase superfamily protein	Red ontogenesis	Biotic Stress / Betaglucanase
ppa013919m	1.06	0.015945555		Red ontogenesis	
ppa000549m	1.06	0.007525695	ubiquitin-activating enzyme 1	Red ontogenesis	
ppa005147m	1.06	0.020213823	Protein kinase superfamily protein	Red ontogenesis	
ppa004792m	1.06	0.034557692	photolyase 1	Red ontogenesis	
ppa011833m	1.06	0.036607138	RING-H2 finger B1A	Red ontogenesis	
ppa003458m	1.06	0.005819796	Thiamine pyrophosphate dependent pyruvate decarboxylase fam	Red ontogenesis	
ppa005467m	1.06	0.00829426	amino acid permease 7	Red ontogenesis	
ppa002291m	1.06	0.013931779	Concanavalin A-like lectin protein kinase family protein	Red ontogenesis	

ppa024292m	1.06	0.049262532	disease resistance protein (TIR-NBS-LRR class), putative	Red ontogenesis	Biotic Stress / PR-proteins
ppa009953m	1.06	0.016592262	Cyclase family protein	Red ontogenesis	
ppa012136m	1.05	0.016755865		Red ontogenesis	
ppa002542m	1.05	0.006648758	sulfate transporter 1;3	Red ontogenesis	
ppa002688m	1.05	0.013774179	SCARECROW-like 8	Red ontogenesis	
ppa013017m	1.05	0.023333004		Red ontogenesis	
ppa010636m	1.05	0.023558303	20S proteasome beta subunit G1	Red ontogenesis	
ppa009082m	1.05	0.032166282	mucin-related	Red ontogenesis	
ppa003203m	1.05	0.008725417	MAC/Perforin domain-containing protein	Red ontogenesis	
ppa004272m	1.05	0.049272325		Red ontogenesis	
ppa011498m	1.05	0.006965613	adenosine-5\'-phosphosulfate (APS) kinase 3	Red ontogenesis	
ppa009720m	1.05	0.013095596	nuclear factor Y, subunit C2	Red ontogenesis	
ppa001652m	1.05	0.006603986	K+ uptake transporter 3	Red ontogenesis	
ppa012153m	1.05	0.010490643	ADP-ribosylation factor A1F	Red ontogenesis	
ppa003795m	1.05	0.009172422	calcium-dependent protein kinase 1	Red ontogenesis	
ppa008867m	1.05	0.00829426	NAD(P)-binding Rossmann-fold superfamily protein	Red ontogenesis	
ppa003967m	1.05	0.017389145	DNA photolyase family protein	Red ontogenesis	
ppa006404m	1.05	0.008502919	Serinc-domain containing serine and sphingolipid biosynthesis pro	Red ontogenesis	
ppa011765m	1.05	0.028367812	Ubiquitin-like superfamily protein	Red ontogenesis	
ppa003549m	1.05	0.010971349	dicarboxylate transporter 1	Red ontogenesis	
ppa009770m	1.04	0.008385045	SNARE-like superfamily protein	Red ontogenesis	
ppa009180m	1.04	0.017859366	heat shock transcription factor B2A	Red ontogenesis	
ppa005109m	1.04	0.043335795	histone-lysine N-methyltransferase ATXR2	Red ontogenesis	
ppa010450m	1.04	0.010728592	RPM1 interacting protein 4	Red ontogenesis	
ppa013881m	1.04	0.006942319	BTB/POZ domain-containing protein	Red ontogenesis	
ppa010566m	1.04	0.047207157	RNA recognition motif and CCHC-type zinc finger domains contai	Red ontogenesis	
ppa002690m	1.04	0.007445057	high mobility group	Red ontogenesis	
ppa008313m	1.04	0.023764182	Nucleotide-diphospho-sugar transferases superfamily protein	Red ontogenesis	
ppa000794m	1.04	0.017766977	transferases, transferring glycosyl groups	Red ontogenesis	
ppa011335m	1.04	0.009251469	endoplasmic reticulum retention defective 2B	Red ontogenesis	
ppa003662m	1.04	0.007449977	Phosphofructokinase family protein	Red ontogenesis	
ppa012512m	1.03	0.016848366	Ribonuclease E inhibitor RraA/Dimethylmenaquinone methyltrans	Red ontogenesis	
ppa012959m	1.03	0.009384491	microsomal glutathione s-transferase, putative	Red ontogenesis	Biotic Stress / Glutathione-S-Transferase
ppa010002m	1.03	0.031859275	Adenine nucleotide alpha hydrolases-like superfamily protein	Red ontogenesis	
ppa007416m	1.03	0.007823842	NAD(P)H dehydrogenase (quinone)s	Red ontogenesis	
ppa008251m	1.03	0.024148973	SNF1-related protein kinase 2.7	Red ontogenesis	
ppa012768m	1.03	0.009759111	MD-2-related lipid recognition domain-containing protein / ML don	Red ontogenesis	
ppa012249m	1.03	0.008734551	Glutaredoxin family protein	Red ontogenesis	
ppa023884m	1.02	0.0446579	Sec14p-like phosphatidylinositol transfer family protein	Red ontogenesis	
ppa007790m	1.02	0.007767546	adenine nucleotide transporter 1	Red ontogenesis	
ppa010922m	1.02	0.038868095	phosducin-like protein 3 homolog	Red ontogenesis	
ppa014171m	1.02	0.019529442		Red ontogenesis	
ppa011214m	1.02	0.010489185	NFU domain protein 1	Red ontogenesis	
ppa010782m	1.02	0.016974468	Embryo-specific protein 3, (ATS3)	Red ontogenesis	
ppa012043m	1.02	0.012673819	WCRKC thioredoxin 1	Red ontogenesis	
ppa012040m	1.02	0.029625358	F-box family protein	Red ontogenesis	
ppa013684m	1.02	0.013853915	Uncharacterised protein family (UPF0041)	Red ontogenesis	
ppa001880m	1.02	0.008575589	AMP-dependent synthetase and ligase family protein	Red ontogenesis	
ppa010626m	1.02	0.011730293	LisH and RanBPM domains containing protein	Red ontogenesis	
ppa011334m	1.02	0.046851546	glutathione S-transferase TAU 19	Red ontogenesis	Biotic Stress / Glutathione-S-Transferase
ppa001180m	1.02	0.008122069	Topoisomerase II-associated protein PAT1	Red ontogenesis	
ppa011226m	1.02	0.022622011	RAB GTPase homolog A1C	Red ontogenesis	
ppa018380m	1.02	0.009875695	S-locus lectin protein kinase family protein	Red ontogenesis	
ppa005127m	1.01	0.015599996	Integrin-linked protein kinase family	Red ontogenesis	
ppa005083m	1.01	0.047092481	Homeodomain-like superfamily protein	Red ontogenesis	
ppa009150m	1.01	0.00857215	carboxyesterase 13	Red ontogenesis	
ppa005644m	1.01	0.00950915	tubulin beta-1 chain	Red ontogenesis	
ppa002300m	1.01	0.028290678	RNA helicase family protein	Red ontogenesis	
ppa002633m	1.01	0.009320634	exocyst subunit exo70 family protein A1	Red ontogenesis	
ppa008823m	1.01	0.014796694	nuclear encoded CLP protease 5	Red ontogenesis	
ppa011798m	1.01	0.009017952	Isochorismatase family protein	Red ontogenesis	
ppa003139m	1.01	0.039030183	plant U-box 14	Red ontogenesis	
ppa008153m	1.01	0.009562978	Syntaxin/t-SNARE family protein	Red ontogenesis	
ppa003658m	1.01	0.010656506	AMP-dependent synthetase and ligase family protein	Red ontogenesis	Secondary Metabolism / Phenylpropanoids - Biotic Stress / Secondary metabolism involved into stress
ppa009300m	1.01	0.011606676	ribosomal protein L5 B	Red ontogenesis	
ppa011337m	1.01	0.009906993	glutathione S-transferase PHI 9	Red ontogenesis	Biotic Stress / Glutathione-S-Transferase
ppa013624m	1.01	0.010042124	FK506-binding protein 12	Red ontogenesis	
ppa012064m	1.01	0.02866234	Basic-leucine zipper (bZIP) transcription factor family protein	Red ontogenesis	
ppa003388m	1.01	0.010292089	Auxin-responsive GH3 family protein	Red ontogenesis	
ppa013870m	1.00	0.029488957	Hypoxia-responsive family protein	Red ontogenesis	
ppa014550m	1.00	0.029526243	Oligosaccaryltransferase	Red ontogenesis	
ppa004323m	1.00	0.00994837	glucose-6-phosphate dehydrogenase 6	Red ontogenesis	
ppa014081m	1.00	0.011761988		Red ontogenesis	
ppa000284m	1.00	0.012026061	calmodulin binding	Red ontogenesis	
ppa004924m	1.00	0.017515525	UDP-glucosyl transferase 73B3	Red ontogenesis	
ppa003462m	1.00	0.010284326	ACT-like protein tyrosine kinase family protein	Red ontogenesis	
ppa001607m	1.00	0.011659374	ATPase, AAA-type, CDC48 protein	Red ontogenesis	
ppa018565m	1.00	0.02144393		Red ontogenesis	
ppa003210m	-1.00	0.035794834	NADP-malic enzyme 3	Red ontogenesis	
ppa011541m	-1.00	0.049054557	chaperone binding;ATPase activators	Red ontogenesis	
ppa019234m	-1.01	0.022667288	apurinic endonuclease-redox protein	Red ontogenesis	
ppa026016m	-1.01	0.013307792	Tetratricopeptide repeat (TPR)-like superfamily protein	Red ontogenesis	
ppa002448m	-1.01	0.011168838	Rhodanese/Cell cycle control phosphatase superfamily protein	Red ontogenesis	
ppa018503m	-1.01	0.013878621	Tetratricopeptide repeat (TPR)-like superfamily protein	Red ontogenesis	
ppa004683m	-1.01	0.025587605	arginosuccinate synthase family	Red ontogenesis	
ppa004286m	-1.01	0.047491399	Lung seven transmembrane receptor family protein	Red ontogenesis	
ppa000223m	-1.01	0.04017222		Red ontogenesis	
ppa008756m	-1.01	0.012395246	alpha/beta-Hydrolases superfamily protein	Red ontogenesis	
ppa1027211m	-1.01	0.014641156	chloroplastic drought-induced stress protein of 32 kD	Red ontogenesis	
ppa014774m	-1.01	0.045700463		Red ontogenesis	
ppa007747m	-1.01	0.019700596	Tetratricopeptide repeat (TPR)-like superfamily protein	Red ontogenesis	
ppa003128m	-1.01	0.012147783	Microtubule associated protein (MAP65/ASE1) family protein	Red ontogenesis	
ppa004985m	-1.02	0.021654961	Protein kinase superfamily protein	Red ontogenesis	
ppa004660m	-1.02	0.02759567	IQ-domain 23	Red ontogenesis	
ppa005396m	-1.02	0.008050823	PDI-like 2-2	Red ontogenesis	
ppa025640m	-1.02	0.009423108	2-oxoglutarate (2OG) and Fe(II)-dependent oxygenase superfami	Red ontogenesis	
ppa022366m	-1.02	0.012985823	photosystem I subunit D-2	Red ontogenesis	Photosynthesis / Photosystem I
ppa009072m	-1.02	0.043073108	Duplicated homeodomain-like superfamily protein	Red ontogenesis	
ppa009731m	-1.02	0.010156394	dual specificity protein phosphatase (DsPTP1) family protein	Red ontogenesis	
ppa002098m	-1.02	0.014736535	delta 1-pyrroline-5-carboxylate synthase 2	Red ontogenesis	
ppa001847m	-1.02	0.008312046	PLC-like phosphodiesterase family protein	Red ontogenesis	
ppa002031m	-1.03	0.041191623	acyl-activating enzyme 18	Red ontogenesis	
ppa026495m	-1.03	0.043396153		Red ontogenesis	
ppa003228m	-1.03	0.013003481	RAP	Red ontogenesis	
ppa005175m	-1.03	0.038145862	3\'-phosphoinositide-dependent protein kinase 1	Red ontogenesis	
ppa017774m	-1.03	0.015418286	Cysteine proteinases superfamily protein	Red ontogenesis	
ppb020505m	-1.03	0.031668363		Red ontogenesis	
ppa007814m	-1.03	0.030208582	basic leucine zipper 9	Red ontogenesis	
ppa004857m	-1.03	0.032612213	Pyridoxal-dependent decarboxylase family protein	Red ontogenesis	
ppa008539m	-1.03	0.009215449	myb-like transcription factor family protein	Red ontogenesis	
ppa001909m	-1.03	0.024926512	cellulose synthase-like B4	Red ontogenesis	
ppa006382m	-1.03	0.039229854	spliceosome protein-related	Red ontogenesis	
ppa025696m	-1.03	0.021811532	cytochrome P450, family 71, subfamily B, polypeptide 34	Red ontogenesis	
ppa003886m	-1.04	0.007532181	pyridoxin (pyrodoxamine) 5\'-phosphate oxidase	Red ontogenesis	
ppa023366m	-1.04	0.040582777	DNA-binding bromodomain-containing protein	Red ontogenesis	
ppa008746m	-1.04	0.008536786	beta-ketoacyl reductase 1	Red ontogenesis	
ppa003511m	-1.04	0.023206161	Protein kinase superfamily protein	Red ontogenesis	
ppa004017m	-1.04	0.00755291	Amidase family protein	Red ontogenesis	
ppa000558m	-1.04	0.036234187	Double Clp-N motif-containing P-loop nucleoside triphosphate hyd	Red ontogenesis	
ppa011249m	-1.04	0.029532622	Glutathione S-transferase family protein	Red ontogenesis	Biotic Stress / Glutathione-S-Transferase
ppa003717m	-1.04	0.049330391	Ankyrin repeat family protein	Red ontogenesis	
ppa010143m	-1.04	0.04078162	ribosomal protein L15	Red ontogenesis	
ppa006530m	-1.04	0.019153317	Galactosyltransferase family protein	Red ontogenesis	
ppa001733m	-1.04	0.027966934		Red ontogenesis	
ppa000990m	-1.04	0.006803864	Argonaute family protein	Red ontogenesis	
ppa001238m	-1.04	0.019953545	RNA-metabolising metallo-beta-lactamase family protein	Red ontogenesis	
ppa010511m	-1.04	0.023764182	photosystem I light harvesting complex gene 1	Red ontogenesis	Photosynthesis / Photosystem II
ppa005573m	-1.04	0.0164576	TRICHOME BIREFRINGENCE-LIKE 11	Red ontogenesis	
ppa002881m	-1.04	0.027418849	S-adenosyl-L-methionine-dependent methyltransferases superfar	Red ontogenesis	
ppa001575m	-1.05	0.007802495	alpha-amylase-like 3	Red ontogenesis	
ppa000302m	-1.05	0.022355083	COP1-interacting protein-related	Red ontogenesis	
ppa001471m	-1.05	0.008884539	S-adenosyl-L-methionine-dependent methyltransferases superfar	Red ontogenesis	
ppa002564m	-1.05	0.015955377	electron-transfer flavoprotein:ubiquinone oxidoreductase	Red ontogenesis	
ppa008066m	-1.05	0.010094244	UDP-XYL synthase 6	Red ontogenesis	
ppa004257m	-1.05	0.013979689	Seryl-tRNA synthetase	Red ontogenesis	
ppa001931m	-1.05	0.006298232		Red ontogenesis	
ppa014021m	-1.05	0.028242769	acyl-CoA-binding protein 6	Red ontogenesis	
ppa002933m	-1.05	0.025300194	IQ-domain 28	Red ontogenesis	
ppa006467m	-1.05	0.0331625	Domain of unknown function (DUF3598)	Red ontogenesis	
ppa002324m	-1.05	0.032573023	carotenoid isomerase	Red ontogenesis	Biotic Stress / Secondary metabolism involved into stress
ppa004473m	-1.05	0.006154562	cytochrome P450, family 98, subfamily A, polypeptide 3	Red ontogenesis	
ppa008745m	-1.05	0.015721947	Tropomyosin-related	Red ontogenesis	
ppa006606m	-1.05	0.026407816	chloroplast stem-loop binding protein of 41 kDa	Red ontogenesis	
ppa010359m	-1.05	0.025741301	chaperonin 20	Red ontogenesis	
ppa007682m	-1.05	0.024096562	RING/U-box superfamily protein	Red ontogenesis	
ppa000692m	-1.06	0.005926126	glycosyl transferase family 1 protein	Red ontogenesis	
ppa016770m	-1.06	0.012769815	cytochrome P450, family 72, subfamily A, polypeptide 15	Red ontogenesis	
ppa008231m	-1.06	0.027623438	BRI1 kinase inhibitor 1	Red ontogenesis	

ppa003230m	-1.06	0.023600012		Red ontogenesis	
ppa017140m	-1.06	0.008040548	Major facilitator superfamily protein	Red ontogenesis	
ppa005061m	-1.06	0.009215449	DC1 domain-containing protein	Red ontogenesis	
ppa021576m	-1.06	0.048759825	serine carboxypeptidase-like 45	Red ontogenesis	
ppa003864m	-1.06	0.01580899		Red ontogenesis	
ppa003271m	-1.06	0.006866182	ketol-acid reductoisomerase	Red ontogenesis	
ppa003604m	-1.06	0.008602897	RNA-binding CRS1 / YhbY (CRM) domain-containing protein	Red ontogenesis	
ppa002762m	-1.06	0.013334005	basic helix-loop-helix (bHLH) DNA-binding superfamily protein	Red ontogenesis	
ppa011545m	-1.06	0.029463481	plasmodesmata callose-binding protein 3	Red ontogenesis	Biotic Stress / Betaglucanase
ppa009800m	-1.06	0.005630666	anthranilate synthase beta subunit 1	Red ontogenesis	
ppa004943m	-1.06	0.012520914	Major facilitator superfamily protein	Red ontogenesis	
ppa009239m	-1.07	0.039809449		Red ontogenesis	
ppa000138m	-1.07	0.02331926	binding	Red ontogenesis	
ppa007300m	-1.07	0.026835675	heat shock transcription factor A2	Red ontogenesis	
ppa003953m	-1.07	0.005514556	Calcium-dependent lipid-binding (CaLB domain) family protein	Red ontogenesis	
ppa009335m	-1.07	0.006268465	pyrophosphorylase 6	Red ontogenesis	
ppa012104m	-1.07	0.046938312		Red ontogenesis	
ppa007033m	-1.07	0.045811768	riboflavin kinase/FMN hydrolase	Red ontogenesis	
ppa011725m	-1.07	0.022127855	photosystem II BY	Red ontogenesis	Photosynthesis / Photosystem II
ppa000371m	-1.07	0.005113478	ENHANCED DOWNY MILDEW 2	Red ontogenesis	
ppa008508m	-1.07	0.020057466	Mitochondrial transcription termination factor family protein	Red ontogenesis	
ppa007849m	-1.07	0.005104624	multidrug resistance-associated protein 9	Red ontogenesis	
ppa012239m	-1.08	0.027466664	Cold acclimation protein WCOR413 family	Red ontogenesis	
ppa1027142m	-1.08	0.043617408	Homeodomain-like superfamily protein	Red ontogenesis	
ppa017494m	-1.08	0.010086582	Ribosomal protein L9/RNase H1	Red ontogenesis	
ppa006561m	-1.08	0.011273725	Homeodomain-like superfamily protein	Red ontogenesis	
ppa005963m	-1.08	0.039947464	Sucrase/ferredoxin-like family protein	Red ontogenesis	
ppa014035m	-1.08	0.035150131		Red ontogenesis	
ppa001332m	-1.08	0.00599895	ARM repeat superfamily protein	Red ontogenesis	
ppa013123m	-1.08	0.008258703		Red ontogenesis	
ppa019693m	-1.08	0.047999721	RNA-binding CRS1 / YhbY (CRM) domain-containing protein	Red ontogenesis	
ppa004812m	-1.09	0.01229947	Peptidase S41 family protein	Red ontogenesis	
ppa010457m	-1.09	0.048016295	auxin-responsive family protein	Red ontogenesis	
ppa012753m	-1.09	0.044722036		Red ontogenesis	
ppa005324m	-1.09	0.018359894	Pyridine nucleotide-disulphide oxidoreductase family protein	Red ontogenesis	Biotic Stress / Secondary metabolism involved into stress
ppa014511m	-1.09	0.046550415		Red ontogenesis	
ppa010093m	-1.09	0.021665885	photosystem II subunit P-1	Red ontogenesis	Photosynthesis / Photosystem II
ppa004474m	-1.09	0.013329791	cytochrome P450, family 98, subfamily A, polypeptide 3	Red ontogenesis	
ppa011582m	-1.09	0.028665693	Protein of unknown function (DUF565)	Red ontogenesis	
ppa009103m	-1.09	0.037174593	Duplicated homeodomain-like superfamily protein	Red ontogenesis	
ppa022227m	-1.09	0.005430126	non-intrinsic ABC protein 3	Red ontogenesis	
ppa008173m	-1.09	0.034768463	putative endonuclease or glycosyl hydrolase with C2H2-type zinc	Red ontogenesis	
ppa001198m	-1.09	0.049054557	Kinase-related protein of unknown function (DUF1296)	Red ontogenesis	
ppa026896m	-1.10	0.004418333	plastid transcriptionally active 5	Red ontogenesis	
ppa003329m	-1.10	0.037231611	Protein kinase family protein	Red ontogenesis	
ppa005432m	-1.10	0.00686857	4-hydroxy-3-methylbut-2-enyl diphosphate reductase	Red ontogenesis	Biotic Stress / Secondary metabolism involved into stress
ppa000834m	-1.10	0.04336953	Pentatricopeptide repeat (PPR) superfamily protein	Red ontogenesis	
ppa001465m	-1.10	0.027026669	phosphatidyl inositol monophosphate 5 kinase	Red ontogenesis	
ppa002632m	-1.10	0.020574896		Red ontogenesis	
ppa010263m	-1.11	0.008605588	ATP synthase delta-subunit gene	Red ontogenesis	Photosynthesis / Redox chain
ppa001224m	-1.11	0.018462058	Rho GTPase activation protein (RhoGAP) with PH domain	Red ontogenesis	
ppa005440m	-1.11	0.007562046	TPX2 (targeting protein for Xklp2) protein family	Red ontogenesis	
ppa001524m	-1.11	0.032612213	RNA-dependent RNA polymerase 1	Red ontogenesis	
ppa024082m	-1.11	0.032973638		Red ontogenesis	
ppa015031m	-1.11	0.019987345	Domain of unknown function (DUF1995)	Red ontogenesis	
ppa011575m	-1.11	0.019419769	chlororespiratory reduction 3	Red ontogenesis	
ppa007700m	-1.11	0.01580812	Sugar isomerase (SIS) family protein	Red ontogenesis	
ppa002596m	-1.11	0.045011457	Pentatricopeptide repeat (PPR-like) superfamily protein	Red ontogenesis	
ppa003336m	-1.11	0.004118209	XS domain-containing protein / XS zinc finger domain-containing	Red ontogenesis	
ppa004603m	-1.12	0.027079412	P-loop containing nucleoside triphosphate hydrolases superfamily	Red ontogenesis	
ppa007266m	-1.12	0.003530074	Hydrolase-like protein family	Red ontogenesis	
ppa000566m	-1.12	0.019087392	Leucine-rich receptor-like protein kinase family protein	Red ontogenesis	
ppa001811m	-1.12	0.00489293	Protein kinase protein with adenine nucleotide alpha hydrolases-l	Red ontogenesis	
ppa005846m	-1.12	0.013466713	Mannose-6-phosphate isomerase, type I	Red ontogenesis	
ppa001214m	-1.12	0.017881232	RNA-binding (RRM/RBD/RNP motifs) family protein	Red ontogenesis	
ppa012507m	-1.12	0.003686112	basic leucine-zipper 44	Red ontogenesis	
ppa020908m	-1.12	0.033809236		Red ontogenesis	
ppa004851m	-1.12	0.003260613	beta-6 tubulin	Red ontogenesis	
ppa011267m	-1.12	0.013116318	Nucleotide-diphospho-sugar transferases superfamily protein	Red ontogenesis	Biotic Stress / Secondary metabolism involved into stress
ppa007455m	-1.13	0.023755238	Oxidoreductase family protein	Red ontogenesis	
ppa005352m	-1.13	0.004060471	APS reductase 3	Red ontogenesis	
ppa001018m	-1.13	0.034714479	DNA mismatch repair protein MutS, type 2	Red ontogenesis	
ppa022740m	-1.13	0.032492881	protein binding;zinc ion binding	Red ontogenesis	
ppa009703m	-1.13	0.004971348	ferritin 2	Red ontogenesis	
ppa004183m	-1.13	0.005357772	peroxin 14	Red ontogenesis	
ppa012309m	-1.13	0.030245971	rotamase cyclophilin 2	Red ontogenesis	
ppa006786m	-1.13	0.003564925	purple acid phosphatase 29	Red ontogenesis	
ppa008000m	-1.13	0.02756566	Ribosomal RNA adenine dimethylase family protein	Red ontogenesis	
ppa000410m	-1.14	0.008734097	nuclear RNA polymerase D2A	Red ontogenesis	
ppa006533m	-1.14	0.020325994	ATP-dependent caseinolytic (Clp) protease/crotonase family prote	Red ontogenesis	
ppa005808m	-1.14	0.04828652	phosphatidylserine decarboxylase 1	Red ontogenesis	
ppa010421m	-1.14	0.03140309	Rhodanese/Cell cycle control phosphatase superfamily protein	Red ontogenesis	
ppa003338m	-1.14	0.022370764	Leucine-rich repeat (LRR) family protein	Red ontogenesis	
ppa011986m	-1.14	0.011950095	RmlC-like cupins superfamily protein	Red ontogenesis	
ppa014599m	-1.14	0.023403995	GBF\'s pro-rich region-interacting factor 1	Red ontogenesis	
ppa010848m	-1.14	0.011606676	FKBP-like peptidyl-prolyl cis-trans isomerase family protein	Red ontogenesis	
ppa006579m	-1.14	0.004105945	isopropylmalate dehydrogenase 2	Red ontogenesis	
ppa006064m	-1.14	0.004421395	DC1 domain-containing protein	Red ontogenesis	
ppa000554m	-1.14	0.031519349	RNAligase	Red ontogenesis	
ppa010197m	-1.15	0.038012099		Red ontogenesis	
ppa000165m	-1.15	0.044641405	dicer-like 3	Red ontogenesis	
ppa006663m	-1.15	0.045018358	FAD-dependent oxidoreductase family protein	Red ontogenesis	
ppa015696m	-1.15	0.046017492	wall associated kinase 3	Red ontogenesis	
ppa007350m	-1.15	0.023837892	CYCLIN D3;2	Red ontogenesis	
ppa000447m	-1.15	0.022130672	carbamoyl phosphate synthetase B	Red ontogenesis	
ppa016077m	-1.16	0.036192069		Red ontogenesis	
ppa009282m	-1.16	0.005701619	ribosomal protein L5 B	Red ontogenesis	
ppa025570m	-1.16	0.003023761	NDH-dependent cyclic electron flow 5	Red ontogenesis	
ppa007417m	-1.16	0.004622335	Homeodomain-like superfamily protein	Red ontogenesis	
ppa007647m	-1.16	0.027783376	Amino acid dehydrogenase family protein	Red ontogenesis	
ppa012090m	-1.16	0.021230101	Ribosomal protein L22p/L17e family protein	Red ontogenesis	
ppa014668m	-1.16	0.04508846		Red ontogenesis	
ppa024482m	-1.16	0.019516785	GDSL-like Lipase/Acylhydrolase superfamily protein	Red ontogenesis	
ppa010364m	-1.16	0.018858695	tonoplast intrinsic protein 1;3	Red ontogenesis	
ppa003384m	-1.16	0.02179559	HIS HF	Red ontogenesis	
ppa019851m	-1.16	0.031085315	xylosyltransferase 1	Red ontogenesis	
ppa003052m	-1.16	0.002324077	TRF-like 6	Red ontogenesis	
ppa002065m	-1.17	0.003789459	Transcriptional factor B3 family protein / auxin-responsive factor A	Red ontogenesis	
ppa001679m	-1.17	0.037083673	endonucleases	Red ontogenesis	
ppa027223m	-1.17	0.015656915	hydroxycinnamoyl-CoA shikimate/quinate hydroxycinnamoyl trans	Red ontogenesis	Secondary Metabolism / Phenylpropanoids - Biotic Stress / Secondary metabolism involved into stress
ppa005829m	-1.17	0.003085013	cryptochrome-interacting basic-helix-loop-helix 1	Red ontogenesis	
ppa009110m	-1.17	0.031383077	photosystem I light harvesting complex gene 3	Red ontogenesis	Photosynthesis / Photosystem II
ppa006105m	-1.17	0.007212564	Galactose oxidase/kelch repeat superfamily protein	Red ontogenesis	
ppa007437m	-1.17	0.039061032	Galactosyltransferase family protein	Red ontogenesis	
ppa001019m	-1.17	0.016019704	Protein of unknown function (DUF3527)	Red ontogenesis	
ppa007051m	-1.17	0.032109145	pfkB-like carbohydrate kinase family protein	Red ontogenesis	
ppa007466m	-1.17	0.036192069	DNAJ heat shock N-terminal domain-containing protein	Red ontogenesis	
ppa014259m	-1.17	0.010115236	Uncharacterised protein family SERF	Red ontogenesis	
ppa004453m	-1.17	0.013979689	Protein kinase superfamily protein	Red ontogenesis	
ppa011990m	-1.17	0.004941345	Calcium-binding EF-hand family protein	Red ontogenesis	
ppa004850m	-1.18	0.04763061	homolog of mamallian P58IPK	Red ontogenesis	
ppa009860m	-1.18	0.028101733	adenosine monophosphate kinase	Red ontogenesis	
ppa015790m	-1.18	0.035423852	P-loop containing nucleoside triphosphate hydrolases superfamily	Red ontogenesis	
ppa010498m	-1.18	0.007622713	Plastid-lipid associated protein PAP / fibrillin family protein	Red ontogenesis	
ppa004899m	-1.18	0.015682064	Tetratricopeptide repeat (TPR)-like superfamily protein	Red ontogenesis	
ppa010278m	-1.18	0.035621393		Red ontogenesis	
ppa000476m	-1.18	0.021431472	DNA binding	Red ontogenesis	
ppa001846m	-1.18	0.019633187	Signal transduction histidine kinase, hybrid-type, ethylene sensor	Red ontogenesis	
ppa013379m	-1.18	0.004161252	2-phosphoglycolate phosphatase 1	Red ontogenesis	
ppa009517m	-1.18	0.04092133	Uncharacterized conserved protein (DUF2358)	Red ontogenesis	
ppa013384m	-1.18	0.015280807	Cytochrome c	Red ontogenesis	
ppb010253m	-1.18	0.002682066	Adenine nucleotide alpha hydrolases-like superfamily protein	Red ontogenesis	
ppa002940m	-1.18	0.026805385	phosphoglucomutase, putative / glucose phosphomutase, putative	Red ontogenesis	
ppa000186m	-1.18	0.019492407	tRNA arginine adenosine deaminase	Red ontogenesis	
ppa008930m	-1.18	0.043617408	Haloacid dehalogenase-like hydrolase (HAD) superfamily protein	Red ontogenesis	
ppa007940m	-1.19	0.029246248	heptahelical transmembrane protein2	Red ontogenesis	
ppa020197m	-1.19	0.020173571	alpha/beta-Hydrolases superfamily protein	Red ontogenesis	
ppa009520m	-1.19	0.024177789	S-adenosyl-L-methionine-dependent methyltransferases superfan	Red ontogenesis	
ppa009649m	-1.19	0.021592591		Red ontogenesis	
ppa003016m	-1.19	0.018107167	S-adenosyl-L-methionine-dependent methyltransferases superfan	Red ontogenesis	
ppa010397m	-1.19	0.002100948	allene oxide cyclase 3	Red ontogenesis	
ppa006017m	-1.19	0.017202658	mitochondrial 28S ribosomal protein S29-related	Red ontogenesis	
ppa012092m	-1.19	0.002100948	Protein of unknown function (DUF567)	Red ontogenesis	
ppa001313m	-1.19	0.018939738	FAR1-related sequence 5	Red ontogenesis	
ppa007987m	-1.19	0.04604308	mitogen-activated protein kinase kinase kinase 19	Red ontogenesis	
ppa006096m	-1.19	0.019505645	DNA binding;DNA-directed RNA polymerases	Red ontogenesis	
ppa003215m	-1.19	0.02749635	Pentatricopeptide repeat (PPR) superfamily protein	Red ontogenesis	
ppa006294m	-1.19	0.035851242	NAD(P)-binding Rossmann-fold superfamily protein	Red ontogenesis	

ppa018727m	-1.19	0.02195465	*pentatricopeptide (PPR) repeat-containing protein*	Red ontogenesis	
ppa010282m	-1.19	0.00284816	*Nucleotide-diphospho-sugar transferases superfamily protein*	Red ontogenesis	
ppa021377m	-1.20	0.009105377	*Sugar isomerase (SIS) family protein*	Red ontogenesis	
ppa006937m	-1.20	0.027166504	*Calcineurin-like metallo-phosphoesterase superfamily protein*	Red ontogenesis	
ppa012095m	-1.20	0.026118161	*Phosphoglycerate mutase family protein*	Red ontogenesis	
ppa006061m	-1.20	0.016026937	*Protein phosphatase 2C family protein*	Red ontogenesis	
ppa001111m	-1.20	0.032109145	*CRS1 / YhbY (CRM) domain-containing protein*	Red ontogenesis	
ppa006978m	-1.20	0.016739899	*UBX domain-containing protein*	Red ontogenesis	
ppa004074m	-1.20	0.010464376	*Nucleic acid-binding proteins superfamily*	Red ontogenesis	
ppa000962m	-1.20	0.028275457	*FtsH extracellular protease family*	Red ontogenesis	
ppa009182m	-1.21	0.035600257	*Tetratricopeptide repeat (TPR)-like superfamily protein*	Red ontogenesis	
ppa000756m	-1.21	0.03513258	*CCAAT-binding factor*	Red ontogenesis	
ppa014256m	-1.21	0.024148973	*Uncharacterised protein family SERF*	Red ontogenesis	
ppa009010m	-1.21	0.001593388	*3-oxo-5-alpha-steroid 4-dehydrogenase family protein*	Red ontogenesis	
ppa003093m	-1.21	0.025866181	*TCP-1/cpn60 chaperonin family protein*	Red ontogenesis	
ppa018437m	-1.21	0.022904322	*Mitochondrial ATP synthase subunit G protein*	Red ontogenesis	
ppa001850m	-1.22	0.031383077	*Calcium-dependent lipid-binding (CaLB domain) plant phosphorib*	Red ontogenesis	
ppa005032m	-1.22	0.034873953	*aspartate aminotransferase*	Red ontogenesis	
ppa005001m	-1.22	0.005033974	*sulfoquinovosyldiacylglycerol 1*	Red ontogenesis	
ppa004196m	-1.22	0.003773571	*ADP glucose pyrophosphorylase 1*	Red ontogenesis	
ppa001263m	-1.22	0.015955377	*genomes uncoupled 1*	Red ontogenesis	
ppa021964m	-1.23	0.008464523	*serine acetyltransferase 3;2*	Red ontogenesis	
ppa011489m	-1.23	0.009035018	*Protein of unknown function (DUF1639)*	Red ontogenesis	
ppa009474m	-1.23	0.007071212	*biotin/lipoyl attachment domain-containing protein*	Red ontogenesis	
ppa002600m	-1.23	0.002721765	*epsin N-terminal homology (ENTH) domain-containing protein / cl*	Red ontogenesis	
ppa008147m	-1.23	0.017806202	*purple acid phosphatase 3*	Red ontogenesis	
ppa012541m	-1.23	0.012930908	*Protein of unknown function, DUF584*	Red ontogenesis	
ppa005267m	-1.23	0.003365116	*CBL-interacting protein kinase 21*	Red ontogenesis	
ppa002923m	-1.24	0.002221109	*gamma-glutamyl transpeptidase 4*	Red ontogenesis	
ppa023083m	-1.24	0.027284842	*S-adenosyl-L-methionine-dependent methyltransferases superfar*	Red ontogenesis	
ppa008910m	-1.24	0.001750274	*Heavy metal transport/detoxification superfamily protein*	Red ontogenesis	
ppa003704m	-1.24	0.021099014	*Heat shock protein DnaJ with tetratricopeptide repeat*	Red ontogenesis	
ppa003518m	-1.24	0.001807543	*Phosphofructokinase family protein*	Red ontogenesis	
ppa009492m	-1.24	0.005518107	*Aldolase-type TIM barrel family protein*	Red ontogenesis	
ppa017093m	-1.24	0.032447442	*AMP-dependent synthetase and ligase family protein*	Red ontogenesis	Secondary Metabolism / Phenylpropanoids - Biotic Stress / Secondary metabolism involved into stress
ppa010081m	-1.24	0.027783376	*chlorophyll A/B-binding protein 2*	Red ontogenesis	Photosynthesis / Photosystem II
ppa000951m	-1.24	0.038321596	*alpha-amylase-like 3*	Red ontogenesis	
ppa014767m	-1.24	0.01606996	*ferric reduction oxidase 4*	Red ontogenesis	
ppa002169m	-1.25	0.011959494	*pentatricopeptide (PPR) repeat-containing protein*	Red ontogenesis	
ppa004020m	-1.25	0.002887547	*3-deoxy-d-arabino-heptulosonate 7-phosphate synthase*	Red ontogenesis	
ppa008698m	-1.25	0.012044362	*Peroxidase superfamily protein*	Red ontogenesis	
ppa017186m	-1.25	0.002135311	*UDP-glucosyl transferase 88A1*	Red ontogenesis	
ppa003002m	-1.25	0.031437876	*DYNAMIN-like 1E*	Red ontogenesis	
ppa012028m	-1.25	0.034418996	*plant-specific transcription factor YABBY family protein*	Red ontogenesis	
ppa008661m	-1.25	0.00741948	*Oxidoreductase, zinc-binding dehydrogenase family protein*	Red ontogenesis	
ppa012645m	-1.25	0.041395297	*Protein of unknown function (DUF1195)*	Red ontogenesis	
ppa004928m	-1.25	0.003082833	*Ubiquitin-specific protease family C19-related protein*	Red ontogenesis	
ppa008622m	-1.25	0.016124253	*GroES-like zinc-binding alcohol dehydrogenase family protein*	Red ontogenesis	
ppa004965m	-1.25	0.030764284	*Protein kinase superfamily protein*	Red ontogenesis	
ppa009563m	-1.25	0.002269716	*uridine-ribohydrolase 2*	Red ontogenesis	
ppa024511m	-1.26	0.041351584	*Adenine nucleotide alpha hydrolases-like superfamily protein*	Red ontogenesis	
ppa010038m	-1.26	0.006556773	*dehydroascorbate reductase 1*	Red ontogenesis	
ppa010281m	-1.26	0.003781072	*SGNH hydrolase-type esterase superfamily protein*	Red ontogenesis	
ppa015773m	-1.26	0.012923782	*ribosome recycling factor, chloroplast precursor*	Red ontogenesis	
ppa002230m	-1.26	0.04754988	*auxin response factor 11*	Red ontogenesis	
ppa004908m	-1.26	0.025197531	*Metallo-hydrolase/oxidoreductase superfamily protein*	Red ontogenesis	
ppa026503m	-1.26	0.03537645		Red ontogenesis	
ppa002871m	-1.26	0.01457888	*somatic embryogenesis receptor-like kinase 1*	Red ontogenesis	
ppa002062m	-1.26	0.00287954	*translocon at the outer envelope membrane of chloroplasts 75-III*	Red ontogenesis	
ppa005962m	-1.26	0.00276138	*PHYTOENE SYNTHASE*	Red ontogenesis	Biotic Stress / Secondary metabolism involved into stress
ppa003168m	-1.26	0.003518082	*Leucine-rich repeat protein kinase family protein*	Red ontogenesis	
ppa007030m	-1.26	0.033889481	*OSBP(oxysterol binding protein)-related protein 4C*	Red ontogenesis	
ppa009873m	-1.26	0.036537867	*heme oxygenase 2*	Red ontogenesis	
ppa006638m	-1.27	0.000985547	*Nucleotidylyl transferase superfamily protein*	Red ontogenesis	
ppa006328m	-1.27	0.000858964	*fatty acyl-ACP thioesterases B*	Red ontogenesis	
ppa016812m	-1.27	0.026945086	*RNA-binding S4 domain-containing protein*	Red ontogenesis	
ppa006788m	-1.27	0.030756493	*protochlorophyllide oxidoreductase A*	Red ontogenesis	
ppa004544m	-1.27	0.004150664	*cinnamate-4-hydroxylase*	Red ontogenesis	
ppa017864m	-1.27	0.021580423	*TEOSINTE BRANCHED, cycloidea and PCF (TCP) 14*	Red ontogenesis	
ppa022268m	-1.27	0.030407898	*PsbP-like protein 2*	Red ontogenesis	Photosynthesis / Photosystem II
ppa007368m	-1.27	0.000923189	*geranylgeranyl pyrophosphate synthase 1*	Red ontogenesis	Biotic Stress / Secondary metabolism involved into stress
ppa001548m	-1.27	0.027883188	*paralog of ARC6*	Red ontogenesis	
ppa009989m	-1.28	0.005191256	*methyl-CPG-binding domain 10*	Red ontogenesis	
ppa011822m	-1.28	0.019424809	*Uncharacterised BCR, YbaB family COG0718*	Red ontogenesis	
ppa008947m	-1.28	0.028415995	*endoplasmic reticulum-adenine nucleotide transporter 1*	Red ontogenesis	
ppa005306m	-1.28	0.002277091	*Arabinanase/levansucrase/invertase*	Red ontogenesis	
ppa018924m	-1.28	0.011145239	*Ankyrin repeat family protein*	Red ontogenesis	
ppa011737m	-1.28	0.024668936	*RAC-like 3*	Red ontogenesis	
ppa023890m	-1.28	0.013329791	*D-isomer specific 2-hydroxyacid dehydrogenase family protein*	Red ontogenesis	
ppa011583m	-1.28	0.010210907	*Protein of unknown function (DUF1118)*	Red ontogenesis	
ppa000739m	-1.28	0.043285151	*Leucine-rich receptor-like protein kinase family protein*	Red ontogenesis	
ppa019522m	-1.28	0.040681696	*myb domain protein 4*	Red ontogenesis	
ppa013029m	-1.28	0.00104421	*RING/U-box superfamily protein*	Red ontogenesis	
ppa004150m	-1.28	0.00709784	*O-fucosyltransferase family protein*	Red ontogenesis	
ppa014706m	-1.29	0.025889884	*Di-glucose binding protein with Kinesin motor domain*	Red ontogenesis	
ppa008374m	-1.29	0.030771502		Red ontogenesis	
ppa003821m	-1.29	0.004548453	*CYTOCHROME P450 51G1*	Red ontogenesis	
ppa004900m	-1.29	0.037992815		Red ontogenesis	
ppa019898m	-1.29	0.0143516	*Protein of unknown function (DUF3464)*	Red ontogenesis	
ppa001633m	-1.29	0.04223656	*Transducin/WD40 repeat-like superfamily protein*	Red ontogenesis	
ppa000354m	-1.30	0.030825265		Red ontogenesis	
ppa003081m	-1.30	0.010953295	*receptor like protein 4*	Red ontogenesis	
ppa004409m	-1.30	0.031088336	*2-oxoglutarate (2OG) and Fe(II)-dependent oxygenase superfami*	Red ontogenesis	
ppa006497m	-1.30	0.004265027	*shaggy-like kinase 13*	Red ontogenesis	
ppa019477m	-1.30	0.001868113		Red ontogenesis	
ppa019198m	-1.30	0.045574089	*homeodomain GLABROUS 2*	Red ontogenesis	
ppa019654m	-1.30	0.023837892		Red ontogenesis	
ppa003485m	-1.30	0.008586821	*Plant protein of unknown function (DUF869)*	Red ontogenesis	
ppa005312m	-1.31	0.021344464	*2-oxoacid dehydrogenases acyltransferase family protein*	Red ontogenesis	
ppa001134m	-1.31	0.010028548	*CRM family member 3A*	Red ontogenesis	
ppa007509m	-1.31	0.03320287	*RING/FYVE/PHD zinc finger superfamily protein*	Red ontogenesis	
ppa007097m	-1.31	0.018312682	*Mitochondrial transcription termination factor family protein*	Red ontogenesis	
ppa008475m	-1.31	0.001025841	*Protein kinase superfamily protein*	Red ontogenesis	
ppa002982m	-1.31	0.013442103	*dehydratase family*	Red ontogenesis	
ppa004063m	-1.31	0.00581261	*galacturonosyltransferase 10*	Red ontogenesis	
ppa007725m	-1.31	0.004439652	*NDH-dependent cyclic electron flow 1*	Red ontogenesis	
ppa005677m	-1.31	0.007553212	*Fatty acid/sphingolipid desaturase*	Red ontogenesis	
ppa009432m	-1.31	0.001349402	*RNA-binding KH domain-containing protein*	Red ontogenesis	
ppa013064m	-1.31	0.021592007	*photosystem I subunit O*	Red ontogenesis	Photosynthesis / Photosystem I
ppa009831m	-1.31	0.030208582		Red ontogenesis	
ppa004359m	-1.31	0.013918626	*indeterminate(ID)-domain 4*	Red ontogenesis	
ppa001487m	-1.32	0.0010278	*Chaperone protein htpG family protein*	Red ontogenesis	
ppa019318m	-1.32	0.021791191	*F-box/RNI-like superfamily protein*	Red ontogenesis	
ppa020906m	-1.32	0.012026061	*nodulin MtN21 /EamA-like transporter family protein*	Red ontogenesis	
ppa020890m	-1.32	0.003632142	*Uncharacterised conserved protein UCP015417, vWA*	Red ontogenesis	
ppa010327m	-1.32	0.025113001	*Chaperone DnaJ-domain superfamily protein*	Red ontogenesis	
ppa013406m	-1.32	0.021984005	*photosystem II subunit X*	Red ontogenesis	
ppa006575m	-1.32	0.010678469	*Protein of unknown function (DUF506)*	Red ontogenesis	
ppa022037m	-1.32	0.00387322	*Uncharacterized protein family (UPF0016)*	Red ontogenesis	
ppa000680m	-1.33	0.018241241	*P-loop containing nucleoside triphosphate hydrolases superfamily*	Red ontogenesis	
ppa008991m	-1.33	0.018274288	*Mitochondrial transcription termination factor family protein*	Red ontogenesis	
ppa003161m	-1.33	0.0037165	*protein kinase family protein / protein phosphatase 2C (PP2C) fa*	Red ontogenesis	
ppa002725m	-1.33	0.009910414	*Pyridoxal phosphate (PLP)-dependent transferases superfamily p*	Red ontogenesis	
ppa006721m	-1.33	0.02724031	*G protein coupled receptor*	Red ontogenesis	
ppa001326m	-1.33	0.004937329	*Cyclophilin-like peptidyl-prolyl cis-trans isomerase family protein*	Red ontogenesis	
ppa025416m	-1.33	0.011992323	*FK506 BINDING PROTEIN 53*	Red ontogenesis	
ppa001654m	-1.33	0.0159787	*Tetratricopeptide repeat (TPR)-like superfamily protein*	Red ontogenesis	
ppa013473m	-1.33	0.023629319		Red ontogenesis	
ppa019890m	-1.33	0.001872839		Red ontogenesis	
ppa011499m	-1.33	0.006122869	*Drought-responsive family protein*	Red ontogenesis	
ppa014677m	-1.33	0.027783376	*Mitochondrial transcription termination factor family protein*	Red ontogenesis	
ppa001634m	-1.33	0.021665885	*lipoxygenase 2*	Red ontogenesis	
ppa007540m	-1.34	0.031140475	*Protein kinase superfamily protein*	Red ontogenesis	
ppa000541m	-1.34	0.037579272	*SU(VAR)3-9 homolog 6*	Red ontogenesis	
ppa002200m	-1.34	0.008029436	*white-brown complex homolog protein 11*	Red ontogenesis	
ppa012111m	-1.34	0.048146951	*RS-containing zinc finger protein 21*	Red ontogenesis	
ppa000178m	-1.34	0.012333465	*global transcription factor group A2*	Red ontogenesis	
ppa018271m	-1.34	0.045949505	*NAD(P)-linked oxidoreductase superfamily protein*	Red ontogenesis	
ppa014212m	-1.34	0.005823232	*metallothionein 2B*	Red ontogenesis	
ppa002207m	-1.34	0.009562978	*white-brown complex homolog protein 11*	Red ontogenesis	
ppa008250m	-1.34	0.002170803	*glyceraldehyde-3-phosphate dehydrogenase C subunit 1*	Red ontogenesis	
ppa021648m	-1.34	0.003848315	*Ankyrin repeat family protein*	Red ontogenesis	
ppa005084m	-1.34	0.003354538	*Tubulin/FtsZ family protein*	Red ontogenesis	
ppa005518m	-1.34	0.011495431	*P-loop containing nucleoside triphosphate hydrolases superfamily*	Red ontogenesis	
ppa002345m	-1.34	0.001419009	*Galactosyltransferase family protein*	Red ontogenesis	
ppa003471m	-1.34	0.000560234	*rotamase FKBP 1*	Red ontogenesis	
ppa026050m	-1.34	0.04828652	*HXXXD-type acyl-transferase family protein*	Red ontogenesis	Secondary metabolism / Anthocyanins - Biotic Stress / Secondary metabolism involved into stress
ppa015862m	-1.35	0.019987345	*Nuclear transport factor 2 (NTF2) family protein*	Red ontogenesis	

ppa012252m	-1.35	0.001135429	AIG2-like (avirulence induced gene) family protein	Red ontogenesis	
ppa018625m	-1.35	0.000389073	Dynamin related protein 4C	Red ontogenesis	
ppa000088m	-1.35	0.010846026	nuclear RNA polymerase D1B	Red ontogenesis	
ppa003018m	-1.35	0.043782859	S-adenosyl-L-methionine-dependent methyltransferases superfar	Red ontogenesis	
ppa004867m	-1.35	0.038402692	adenylate kinase family protein	Red ontogenesis	
ppa006463m	-1.35	0.017266592	ARG1-like 1	Red ontogenesis	
ppa017090m	-1.35	0.018605471	NagB/RpiA/CoA transferase-like superfamily protein	Red ontogenesis	
ppa010691m	-1.35	0.007046625	DNAJ heat shock family protein	Red ontogenesis	
ppa007969m	-1.35	0.001508846	2-oxoglutarate (2OG) and Fe(II)-dependent oxygenase superfami	Red ontogenesis	
ppa002053m	-1.35	0.031416697	DHHC-type zinc finger family protein	Red ontogenesis	
ppa006131m	-1.36	0.003693151	RING/U-box superfamily protein	Red ontogenesis	
ppa026994m	-1.36	0.011606676		Red ontogenesis	
ppa002240m	-1.36	0.008237542	Protein kinase superfamily protein	Red ontogenesis	
ppa002880m	-1.37	0.026149478	tRNA/rRNA methyltransferase (SpoU) family protein	Red ontogenesis	
ppa011740m	-1.37	0.010421754	NADPH:quinone oxidoreductase	Red ontogenesis	
ppa000060m	-1.37	0.00508659	binding	Red ontogenesis	
ppa025847m	-1.37	0.007263397		Red ontogenesis	
ppa003449m	-1.37	0.022953805	Mitochondrial transcription termination factor family protein	Red ontogenesis	
ppa009589m	-1.37	0.002180417	alpha/beta-Hydrolases superfamily protein	Red ontogenesis	
ppa002520m	-1.37	0.008725417	RNA-binding CRS1 / YhbY (CRM) domain protein	Red ontogenesis	
ppa000275m	-1.37	0.016319028	DNA LIGASE 6	Red ontogenesis	
ppa018551m	-1.38	0.00868799	auxin-like 1 protein	Red ontogenesis	
ppa001780m	-1.38	0.018101145	transducin family protein / WD-40 repeat family protein	Red ontogenesis	
ppa004066m	-1.38	0.005768161	aluminum-activated, malate transporter 12	Red ontogenesis	
ppa1027172m	-1.38	0.010441587	AIG2-like (avirulence induced gene) family protein	Red ontogenesis	
ppa007738m	-1.38	0.006154562	leucoanthocyanidin dioxygenase	Red ontogenesis	
ppa011853m	-1.38	0.005441837		Red ontogenesis	
ppa023049m	-1.38	0.009875695	Tic22-like family protein	Red ontogenesis	
ppa006214m	-1.38	0.008573524	embryo defective 2737	Red ontogenesis	
ppa003564m	-1.38	0.029772143	CCCH-type zinc fingerfamily protein with RNA-binding domain	Red ontogenesis	
ppa010962m	-1.39	0.045695088	Polynucleotidyl transferase, ribonuclease H-like superfamily prote	Red ontogenesis	
ppa018571m	-1.39	0.001670754	ATP binding;protein kinases;protein serine/threonine kinases	Red ontogenesis	
ppa011404m	-1.39	0.003454955	Calcium-binding EF-hand family protein	Red ontogenesis	
ppa009910m	-1.39	0.035809213	Dihydrodipicolinate reductase, bacterial/plant	Red ontogenesis	
ppa000194m	-1.39	0.00273694	Chaperone DnaJ-domain superfamily protein	Red ontogenesis	
ppa012325m	-1.40	0.01502431		Red ontogenesis	
ppa016728m	-1.40	0.025197531	proteolysis 1	Red ontogenesis	
ppa011104m	-1.40	0.02723342		Red ontogenesis	
ppa025594m	-1.40	0.005358359	Ankyrin repeat family protein	Red ontogenesis	
ppa003382m	-1.40	0.02007682	Pyruvate kinase family protein	Red ontogenesis	
ppa021687m	-1.40	0.0465849	Plant protein of unknown function (DUF247)	Red ontogenesis	
ppa012521m	-1.40	0.003837818	plastocyanin 1	Red ontogenesis	Photosynthesis / Redox chain
ppa024953m	-1.40	0.00138478	enzyme binding;tetrapyrrole binding	Red ontogenesis	
ppa013310m	-1.40	0.008607722	Ribosomal protein S26e family protein	Red ontogenesis	
ppa022078m	-1.40	0.029692691		Red ontogenesis	
ppa002421m	-1.41	0.046127673	tyrosyl-DNA phosphodiesterase-related	Red ontogenesis	
ppa006239m	-1.41	0.004569712	Glycosyl hydrolase superfamily protein	Red ontogenesis	Biotic Stress / Betaglucanase
ppa006379m	-1.41	0.032388879		Red ontogenesis	
ppa000852m	-1.41	0.001419009	Protein of unknown function (DUF3741)	Red ontogenesis	
ppa003560m	-1.41	0.007242909	proteinaceous RNase P 1	Red ontogenesis	
ppa008058m	-1.41	0.009811549	Zinc-binding dehydrogenase family protein	Red ontogenesis	
ppa005107m	-1.41	0.03553163	UDP-Glycosyltransferase superfamily protein	Red ontogenesis	
ppa002735m	-1.41	0.00747161	Plant U-Box 15	Red ontogenesis	
ppa006089m	-1.41	0.009608265	Glucose-6-phosphate/phosphate translocator-related	Red ontogenesis	
ppa013006m	-1.41	0.023091109	RmlC-like cupins superfamily protein	Red ontogenesis	
ppa017055m	-1.42	0.006417088	UDP-glucosyl transferase 85A2	Red ontogenesis	
ppa006888m	-1.42	0.046951042	Chalcone and stilbene synthase family protein	Red ontogenesis	Biotic Stress / Secondary metabolism involved into stress
ppa007182m	-1.42	0.011145239	GroES-like zinc-binding dehydrogenase family protein	Red ontogenesis	
ppa003476m	-1.42	0.011973472	fucosyltransferase 1	Red ontogenesis	
ppa009049m	-1.42	0.000466229		Red ontogenesis	
ppa003429m	-1.42	0.004912384	ACT-like protein tyrosine kinase family protein	Red ontogenesis	
ppa010693m	-1.42	0.002207808		Red ontogenesis	
ppa001468m	-1.42	0.033464081	CRS1 / YhbY (CRM) domain-containing protein	Red ontogenesis	
ppa001943m	-1.42	0.014163017	vernalization5/VIN3-like	Red ontogenesis	
ppa008703m	-1.42	0.00776761	Rho termination factor	Red ontogenesis	
ppa002897m	-1.43	0.005511767	NSP-interacting kinase 1	Red ontogenesis	
ppa019578m	-1.43	0.008715609		Red ontogenesis	
ppa006389m	-1.43	0.049588251	TEOSINTE BRANCHED, cycloidea and PCF (TCP) 14	Red ontogenesis	
ppa000333m	-1.43	0.036169325	FtsH extracellular protease family	Red ontogenesis	
ppa011323m	-1.43	0.00434126		Red ontogenesis	
ppa009985m	-1.43	0.047544305	Alkaline phytoceramidase (aPHC)	Red ontogenesis	
ppa006770m	-1.43	0.010573852	AAR2 protein family	Red ontogenesis	
ppa001962m	-1.43	0.033430727	Plant protein of unknown function (DUF936)	Red ontogenesis	
ppa002899m	-1.43	0.000616381	microtubule-associated proteins 70-1	Red ontogenesis	
ppa003880m	-1.43	0.002222237	beta-hexosaminidase 1	Red ontogenesis	
ppa008650m	-1.43	0.000956963	chorismate mutase 1	Red ontogenesis	
ppa013112m	-1.43	0.004251202	Calcium-binding EF-hand family protein	Red ontogenesis	
ppa023556m	-1.43	0.012520914	peptide deformylase 1A	Red ontogenesis	
ppa008621m	-1.43	0.00328591	LEM3 (ligand-effect modulator 3) family protein / CDC50 family pr	Red ontogenesis	
ppa010716m	-1.44	0.000913244	myb domain protein 4	Red ontogenesis	
ppa005004m	-1.44	0.006652565	Ubiquitin-specific protease family C19-related protein	Red ontogenesis	
ppa007223m	-1.44	0.032282906		Red ontogenesis	
ppa007537m	-1.44	0.015319255	Afadin/alpha-actinin-binding protein	Red ontogenesis	
ppa017043m	-1.44	0.049836121	F-box and associated interaction domains-containing protein	Red ontogenesis	
ppa024043m	-1.44	0.044240359	Embryo-specific protein 3, (ATS3)	Red ontogenesis	
ppa003851m	-1.44	0.00116441	glycosyltransferase 18	Red ontogenesis	
ppa023601m	-1.45	0.048477457		Red ontogenesis	
ppa023497m	-1.45	0.037853168	Late embryogenesis abundant (LEA) hydroxyproline-rich glycopro	Red ontogenesis	
ppa001687m	-1.45	0.037565053	LEUNIG_homolog	Red ontogenesis	
ppa012418m	-1.45	0.003634077	2Fe-2S ferredoxin-like superfamily protein	Red ontogenesis	
ppa002599m	-1.45	0.020838694	pumilio 24	Red ontogenesis	
ppa016888m	-1.45	0.002699472		Red ontogenesis	
ppa003504m	-1.45	0.024172005		Red ontogenesis	
ppa001657m	-1.46	0.042293744	P-loop containing nucleoside triphosphate hydrolases superfamily	Red ontogenesis	
ppa007936m	-1.46	0.006163435	Plant-specific GATA-type zinc finger transcription factor family pro	Red ontogenesis	
ppa021045m	-1.46	0.026389079	Mitochondrial transcription termination factor family protein	Red ontogenesis	
ppa026402m	-1.46	0.018302172	Ankyrin repeat family protein	Red ontogenesis	
ppa007775m	-1.46	0.002304553	2-oxoglutarate (2OG) and Fe(II)-dependent oxygenase superfami	Red ontogenesis	
ppa005264m	-1.46	0.006173697	serine carboxypeptidase-like 2	Red ontogenesis	
ppa016283m	-1.46	0.047491399	Class II aaRS and biotin synthetases superfamily protein	Red ontogenesis	
ppa016365m	-1.46	0.021099014	UDP-Glycosyltransferase superfamily protein	Red ontogenesis	Secondary Metabolism / Dihydroflavonols -Biotic Stress / Secondary metabolism involved into stress
ppa015818m	-1.46	0.020016068		Red ontogenesis	
ppa005289m	-1.46	0.013631431	Eukaryotic aspartyl protease family protein	Red ontogenesis	
ppa010021m	-1.46	0.016579659	Ribosomal protein L19 family protein	Red ontogenesis	
ppa009466m	-1.46	0.015339428	DHBP synthase RibB-like alpha/beta domain	Red ontogenesis	
ppa006812m	-1.46	0.016739899	Homeodomain-like superfamily protein	Red ontogenesis	
ppa000799m	-1.47	0.04821958	Disease resistance protein (TIR-NBS class)	Red ontogenesis	
ppa003443m	-1.47	0.028507179		Red ontogenesis	
ppa025930m	-1.47	0.039828289	aluminum-activated malate transporter 9	Red ontogenesis	
ppa020429m	-1.47	0.012492186	basic helix-loop-helix (bHLH) DNA-binding superfamily protein	Red ontogenesis	
ppa013571m	-1.47	0.022325886	Tautomerase/MIF superfamily protein	Red ontogenesis	
ppa007099m	-1.47	0.043563711	alcohol dehydrogenase 1	Red ontogenesis	
ppa004047m	-1.47	0.023837892	glycine-tRNA ligases	Red ontogenesis	
ppa001779m	-1.47	0.006154562	mechanosensitive channel of small conductance-like 4	Red ontogenesis	
ppa002799m	-1.47	0.024403373	ARM-repeat/Tetratricopeptide repeat (TPR)-like protein	Red ontogenesis	
ppa017025m	-1.47	0.014736535	sigma factor 4	Red ontogenesis	
ppa012494m	-1.47	0.013346953	Bifunctional inhibitor/lipid-transfer protein/seed storage 2S albumi	Red ontogenesis	
ppa001167m	-1.47	0.029506547	Plant protein of unknown function (DUF869)	Red ontogenesis	
ppa002099m	-1.47	0.012522088	PHE ammonia lyase 1	Red ontogenesis	Secondary Metabolism / Phenylpropanoids - Biotic Stress / Secondary metabolism involved into stress
ppa003000m	-1.48	0.032966774	Protein kinase superfamily protein	Red ontogenesis	
ppa005987m	-1.48	0.046176603	Lung seven transmembrane receptor family protein	Red ontogenesis	
ppa009514m	-1.48	0.038881375	Pentatricopeptide repeat (PPR) superfamily protein	Red ontogenesis	
ppa004866m	-1.48	0.044122393	Seven transmembrane MLO family protein	Red ontogenesis	
ppa002766m	-1.48	0.002501332	RH39	Red ontogenesis	
ppa013043m	-1.48	0.014820475	HSP20-like chaperones superfamily protein	Red ontogenesis	
ppa022562m	-1.48	0.031763147	AMP-dependent synthetase and ligase family protein	Red ontogenesis	Secondary Metabolism / Phenylpropanoids - Biotic Stress / Secondary metabolism involved into stress
ppa002079m	-1.48	0.012673819	GTP binding	Red ontogenesis	
ppa018798m	-1.48	0.00734767	Protein kinase family protein	Red ontogenesis	
ppa009739m	-1.48	0.000260625	Ribose 5-phosphate isomerase, type A protein	Red ontogenesis	
ppa005375m	-1.49	0.001502683	O-Glycosyl hydrolases family 17 protein	Red ontogenesis	Biotic Stress / Betaglucanase
ppa005854m	-1.49	0.021791191	P-loop containing nucleoside triphosphate hydrolases superfamily	Red ontogenesis	
ppa003070m	-1.49	0.029923569	F-box/RNI-like superfamily protein	Red ontogenesis	
ppa004791m	-1.49	0.015721947		Red ontogenesis	
ppa014610m	-1.49	0.002626694		Red ontogenesis	
ppa006640m	-1.49	0.00317533	prephenate dehydrogenase family protein	Red ontogenesis	
ppa011722m	-1.49	0.012492186	Caleosin-related family protein	Red ontogenesis	
ppa010423m	-1.49	0.000355835		Red ontogenesis	
ppb014879m	-1.49	0.013509177	Pectinacetylesterase family protein	Red ontogenesis	
ppa002885m	-1.49	0.004622335	inter-alpha-trypsin inhibitor heavy chain-related	Red ontogenesis	Biotic Stress / PR-proteins
ppa008655m	-1.49	0.032346078	magnesium-protoporphyrin IX methyltransferase	Red ontogenesis	
ppa002351m	-1.49	0.023960687	ABC-2 type transporter family protein	Red ontogenesis	
ppa007824m	-1.49	0.004487726	S-adenosyl-L-methionine-dependent methyltransferases superfar	Red ontogenesis	
ppa002383m	-1.49	0.005425499	RNA-binding (RRM/RBD/RNP motifs) family protein	Red ontogenesis	
ppa007919m	-1.50	0.000640294	basic helix-loop-helix (bHLH) DNA-binding superfamily protein	Red ontogenesis	
ppa006957m	-1.50	0.0010278	PLC-like phosphodiesterases superfamily protein	Red ontogenesis	
ppa003039m	-1.50	0.025508365	AAA-type ATPase family protein	Red ontogenesis	
ppa012997m	-1.50	0.002119528		Red ontogenesis	
ppa012096m	-1.50	0.01495342	high mobility group A	Red ontogenesis	

ppa024992m	-1.50	0.018222222	*Galactose oxidase/kelch repeat superfamily protein*	Red ontogenesis	
ppa009826m	-1.50	0.005104624	*TRAM, LAG1 and CLN8 (TLC) lipid-sensing domain containing pr*	Red ontogenesis	
ppa013765m	-1.50	0.02556279	*SAUR-like auxin-responsive protein family*	Red ontogenesis	
ppa018938m	-1.50	0.006866182	*5\'-AMP-activated protein kinase-related*	Red ontogenesis	
ppa023776m	-1.50	0.007525695	*myb domain protein 60*	Red ontogenesis	
ppa000512m	-1.50	0.022176277	*P-loop containing nucleoside triphosphate hydrolases superfamily*	Red ontogenesis	
ppa006209m	-1.51	0.038386183	*GTP-binding family protein*	Red ontogenesis	
ppa002026m	-1.51	0.0173963	*DEAD/DEAH box RNA helicase family protein*	Red ontogenesis	
ppa023767m	-1.51	0.033213204		Red ontogenesis	
ppa008696m	-1.51	0.00829426	*Survival protein SurE-like phosphatase/nucleotidase*	Red ontogenesis	
ppa009593m	-1.51	0.012025969	*biotin carboxyl carrier protein 2*	Red ontogenesis	
ppa003368m	-1.51	0.001569187	*Phototropic-responsive NPH3 family protein*	Red ontogenesis	
ppa001809m	-1.51	0.022384569	*formin homolog 6*	Red ontogenesis	
ppa008255m	-1.51	0.012198169	*glyoxylate reductase 2*	Red ontogenesis	
ppa001266m	-1.51	0.000528377		Red ontogenesis	
ppa013898m	-1.51	0.00475266	*GroES-like family protein*	Red ontogenesis	
ppa005217m	-1.52	0.007415081		Red ontogenesis	
ppa011918m	-1.52	0.002541954	*adenine phosphoribosyltransferase 5*	Red ontogenesis	
ppa005180m	-1.52	0.04769831	*UDP-Glycosyltransferase superfamily protein*	Red ontogenesis	
ppa021176m	-1.52	0.002876114	*IQ-domain 11*	Red ontogenesis	
ppa010347m	-1.52	0.000358847	*alfin-like 5*	Red ontogenesis	
ppa011968m	-1.52	0.000870816	*squamosa promoter binding protein-like 4*	Red ontogenesis	
ppa004745m	-1.52	0.040385164	*AGC (cAMP-dependent, cGMP-dependent and protein kinase C)*	Red ontogenesis	
ppa023764m	-1.53	0.009092059	*ADP glucose pyrophosphorylase large subunit 1*	Red ontogenesis	
ppa009308m	-1.53	0.017743072	*rRNA processing protein-related*	Red ontogenesis	
ppa000782m	-1.53	0.019087354	*disproportionating enzyme 2*	Red ontogenesis	
ppa006728m	-1.53	0.001443964	*BCL-2-associated athanogene 7*	Red ontogenesis	
ppa012978m	-1.53	0.001034993	*Heavy metal transport/detoxification superfamily protein*	Red ontogenesis	
ppa002825m	-1.53	0.000832225	*phosphoglucomutase*	Red ontogenesis	
ppa001590m	-1.53	0.004948598	*Chaperone protein htpG family protein*	Red ontogenesis	
ppa017844m	-1.53	0.027455767	*zinc ion binding*	Red ontogenesis	
ppa000593m	-1.53	0.001920863	*Cellulose synthase family protein*	Red ontogenesis	
ppa002111m	-1.53	0.000132586	*4-hydroxy-3-methylbut-2-enyl diphosphate synthase*	Red ontogenesis	Biotic Stress / Secondary metabolism involved into stress
ppa005719m	-1.53	0.012522088	*Protein of unknown function (DUF1005)*	Red ontogenesis	
ppa004761m	-1.53	0.038447535	*Remorin family protein*	Red ontogenesis	
ppa021736m	-1.53	0.043730435	*DOMAIN OF UNKNOWN FUNCTION 724 7*	Red ontogenesis	
ppa025102m	-1.53	0.001540825	*Thioredoxin z*	Red ontogenesis	
ppa007052m	-1.53	0.000941671	*ATP phosphoribosyl transferase 2*	Red ontogenesis	
ppa001146m	-1.54	0.00901934	*ATP binding microtubule motor family protein*	Red ontogenesis	
ppa011189m	-1.54	0.025889884	*Chalcone-flavanone isomerase family protein*	Red ontogenesis	Biotic Stress / Secondary metabolism involved into stress
ppa024772m	-1.54	0.022953805	*serine racemase*	Red ontogenesis	
ppa009046m	-1.54	0.032704906	*Uncharacterised protein family UPF0090*	Red ontogenesis	
ppa016103m	-1.54	0.000153486	*SC35-like splicing factor 33*	Red ontogenesis	
ppa003723m	-1.54	0.030957907	*acyl-activating enzyme 14*	Red ontogenesis	Secondary Metabolism / Phenylpropanoids - Biotic Stress / Secondary metabolism involved into stress
ppa007813m	-1.54	0.004822938	*triosephosphate isomerase*	Red ontogenesis	
ppa008753m	-1.54	0.011014659	*Cysteine proteinases superfamily protein*	Red ontogenesis	
ppa025117m	-1.54	0.00901934	*D-isomer specific 2-hydroxyacid dehydrogenase family protein*	Red ontogenesis	
ppa001584m	-1.54	0.008250611	*RNA-dependent RNA polymerase family protein*	Red ontogenesis	
ppa002337m	-1.54	0.006999916	*DNA photolyases;DNA photolyases*	Red ontogenesis	
ppa024623m	-1.54	0.010225245	*NB-ARC domain-containing disease resistance protein*	Red ontogenesis	
ppa002150m	-1.55	0.000944258	*STELAR K+ outward rectifier*	Red ontogenesis	
ppa001533m	-1.55	0.017132961	*ATP-dependent RNA helicase, mitochondrial, putative*	Red ontogenesis	
ppa004262m	-1.55	0.002099365	*Amidase family protein*	Red ontogenesis	
ppa008911m	-1.55	0.02029565	*Metallo-hydrolase/oxidoreductase superfamily protein*	Red ontogenesis	
ppa001940m	-1.55	0.003293386	*Leucine-rich repeat protein kinase family protein*	Red ontogenesis	
ppa023493m	-1.56	0.011719399	*Protein kinase superfamily protein*	Red ontogenesis	
ppa012844m	-1.56	0.012062433	*histone H2A 12*	Red ontogenesis	
ppa002503m	-1.56	0.010380403	*S-adenosyl-L-methionine-dependent methyltransferases superfar*	Red ontogenesis	
ppa002776m	-1.56	0.000530118	*XH/XS domain-containing protein*	Red ontogenesis	
ppa002328m	-1.56	0.018919918	*PHE ammonia lyase 1*	Red ontogenesis	Secondary Metabolism / Phenylpropanoids - Biotic Stress / Secondary metabolism involved into stress
ppa024470m	-1.56	0.008385045		Red ontogenesis	
ppa001766m	-1.56	0.006169373	*Protein kinase protein with adenine nucleotide alpha hydrolases-li*	Red ontogenesis	
ppb023753m	-1.57	0.009451232	*cyclic nucleotide gated channel 1*	Red ontogenesis	
ppa013084m	-1.57	0.007133443	*RNA-binding (RRM/RBD/RNP motifs) family protein*	Red ontogenesis	
ppa001041m	-1.57	0.021854531	*Leucine-rich repeat protein kinase family protein*	Red ontogenesis	
ppa002418m	-1.57	0.001556406	*galacturonosyltransferase 1*	Red ontogenesis	
ppa006085m	-1.57	0.043585224		Red ontogenesis	
ppa003996m	-1.57	0.007010921	*Major facilitator superfamily protein*	Red ontogenesis	
ppb023065m	-1.57	0.038939755		Red ontogenesis	
ppa005045m	-1.57	4.13E-05	*UDP-glucose 6-dehydrogenase family protein*	Red ontogenesis	
ppa005381m	-1.57	0.027633525		Red ontogenesis	
ppa000097m	-1.57	0.014015401	*guanyl-nucleotide exchange factors;GTPase binding;GTP binding*	Red ontogenesis	
ppa009735m	-1.58	0.022130672	*ferredoxin-related*	Red ontogenesis	
ppa009133m	-1.58	0.017800596	*RNA-binding (RRM/RBD/RNP motifs) family protein*	Red ontogenesis	
ppa003283m	-1.58	0.002584063	*like SEX4 1*	Red ontogenesis	
ppa001562m	-1.58	0.014213521	*ARM repeat superfamily protein*	Red ontogenesis	
ppa008098m	-1.58	0.009220662	*RNA-binding (RRM/RBD/RNP motifs) family protein*	Red ontogenesis	
ppa007489m	-1.58	0.008238706	*Rhomboid-related intramembrane serine protease family protein*	Red ontogenesis	
ppa007978m	-1.59	0.000193898	*DNAJ heat shock family protein*	Red ontogenesis	
ppa001888m	-1.59	0.001805831	*Leucine-rich repeat protein kinase family protein*	Red ontogenesis	
ppa012067m	-1.59	4.48E-05	*Protein of unknown function (DUF1218)*	Red ontogenesis	
ppa011425m	-1.59	0.010892883		Red ontogenesis	
ppa012625m	-1.59	0.045476964	*RHO guanyl-nucleotide exchange factor 11*	Red ontogenesis	
ppa026117m	-1.59	0.006695035	*Amino acid dehydrogenase family protein*	Red ontogenesis	
ppa024012m	-1.59	0.036361326	*Tetratricopeptide repeat (TPR)-like superfamily protein*	Red ontogenesis	
ppa008983m	-1.59	0.021002487	*S-adenosyl-L-methionine-dependent methyltransferases superfar*	Red ontogenesis	
ppb018970m	-1.59	0.040097313		Red ontogenesis	
ppa006807m	-1.59	0.020996286	*alpha/beta-Hydrolases superfamily protein*	Red ontogenesis	
ppa002356m	-1.59	0.024432333	*Leucine-rich repeat protein kinase family protein*	Red ontogenesis	
ppa003999m	-1.59	3.64E-05	*Xanthine/uracil permease family protein*	Red ontogenesis	
ppa008520m	-1.59	0.013043746	*Protein kinase superfamily protein*	Red ontogenesis	
ppa004654m	-1.60	0.034048801	*transferases, transferring hexosyl groups*	Red ontogenesis	
ppa007802m	-1.60	7.79E-05	*UDP-N-acetylglucosamine (UAA) transporter family*	Red ontogenesis	
ppa001635m	-1.60	0.000316123	*nucleolar protein gar2-related*	Red ontogenesis	
ppa014846m	-1.60	0.018366417	*cyclic nucleotide gated channel 1*	Red ontogenesis	
ppa023657m	-1.60	0.03454413	*Squamosa promoter-binding protein-like (SBP domain) transcripti*	Red ontogenesis	
ppa001312m	-1.60	0.010797581	*starch branching enzyme 2.2*	Red ontogenesis	
ppa002712m	-1.61	0.000202263	*XH/XS domain-containing protein*	Red ontogenesis	
ppa000586m	-1.61	0.014660705	*L-fucokinase/GDP-L-fucose pyrophosphorylase*	Red ontogenesis	
ppa007399m	-1.61	3.85E-05	*spermidine synthase 3*	Red ontogenesis	
ppa009691m	-1.61	0.009486973	*plasma membrane intrinsic protein 3*	Red ontogenesis	
ppa001774m	-1.61	0.003365116	*acid-amino acid ligases;ligases;ATP binding;ATP binding;ligases*	Red ontogenesis	
ppa001074m	-1.61	0.002580902	*starch synthase 3*	Red ontogenesis	
ppa003714m	-1.62	0.003247629	*laccase 17*	Red ontogenesis	Biotic Stress / Secondary metabolism involved into stress
ppa022650m	-1.62	0.001584911	*phosphoglycerate/bisphosphoglycerate mutase family protein*	Red ontogenesis	
ppa024617m	-1.62	0.021372063	*myb domain protein 113*	Red ontogenesis	
ppb025297m	-1.62	0.0164576	*UDP-glucosyl transferase 88A1*	Red ontogenesis	
ppa000300m	-1.62	0.024451444	*IKI3 family protein*	Red ontogenesis	
ppa013361m	-1.62	0.049409099	*phytochrome-associated protein 1*	Red ontogenesis	
ppa001268m	-1.62	0.008124743	*MORN (Membrane Occupation and Recognition Nexus) repeat-co*	Red ontogenesis	
ppa011816m	-1.62	0.045476964		Red ontogenesis	
ppa001428m	-1.62	0.039492061	*DNA topoisomerase, type IA, core*	Red ontogenesis	
ppa006879m	-1.63	0.003031476	*Homeodomain-like superfamily protein*	Red ontogenesis	
ppa002803m	-1.63	0.027418849	*Phototropic-responsive NPH3 family protein*	Red ontogenesis	
ppa013300m	-1.63	0.010163728	*Stress responsive A/B Barrel Domain*	Red ontogenesis	
ppa006724m	-1.63	0.01913683		Red ontogenesis	
ppa011316m	-1.63	0.003785075	*Thioredoxin superfamily protein*	Red ontogenesis	
ppa008215m	-1.63	0.019106449	*Protein kinase superfamily protein*	Red ontogenesis	
ppa015470m	-1.63	0.001868675	*pfkB-like carbohydrate kinase family protein*	Red ontogenesis	
ppa002226m	-1.63	0.001423477	*ATP binding microtubule motor family protein*	Red ontogenesis	
ppa014332m	-1.63	0.009455519		Red ontogenesis	
ppa019531m	-1.63	0.005655952	*BTB/POZ domain-containing protein*	Red ontogenesis	
ppa020758m	-1.64	0.014262679	*myosin 2*	Red ontogenesis	
ppa027016m	-1.64	0.044989633	*defective in meristem silencing 3*	Red ontogenesis	
ppa000651m	-1.64	0.0005045	*P-loop containing nucleoside triphosphate hydrolases superfamily*	Red ontogenesis	
ppa000430m	-1.64	0.019793295	*ATPase E1-E2 type family protein / haloacid dehalogenase-like hy*	Red ontogenesis	
ppa004548m	-1.64	0.004624305	*C2H2-like zinc finger protein*	Red ontogenesis	
ppa005171m	-1.64	0.006973085	*Nucleotidyltransferase family protein*	Red ontogenesis	
ppa000610m	-1.64	0.021172211		Red ontogenesis	
ppa013120m	-1.64	0.049121533	*Bifunctional inhibitor/lipid-transfer protein/seed storage 2S albumi*	Red ontogenesis	
ppa005389m	-1.64	0.034586823	*Protein of unknown function (DUF819)*	Red ontogenesis	
ppa026336m	-1.64	0.036331167	*RING/U-box superfamily protein*	Red ontogenesis	
ppa010942m	-1.64	0.00080588		Red ontogenesis	
ppa011390m	-1.64	0.001708018	*dehydroascorbate reductase 2*	Red ontogenesis	
ppa005609m	-1.65	0.000359707	*aldehyde dehydrogenase 3I1*	Red ontogenesis	
ppa021017m	-1.65	0.009987312	*P-loop containing nucleoside triphosphate hydrolases superfamily*	Red ontogenesis	
ppa016720m	-1.65	0.016432674	*origin recognition complex 1*	Red ontogenesis	
ppa006250m	-1.65	0.009811549	*Glutaredoxin family protein*	Red ontogenesis	
ppa017688m	-1.65	0.034730874	*cytochrome P450, family 71, subfamily A, polypeptide 14*	Red ontogenesis	
ppa000494m	-1.65	0.010896347	*formin homology 1*	Red ontogenesis	
ppa024571m	-1.65	0.031383077	*DNA glycosylase superfamily protein*	Red ontogenesis	
ppa006279m	-1.65	0.000904282	*Core-2/I-branching beta-1,6-N-acetylglucosaminyltransferase fam*	Red ontogenesis	
ppa007316m	-1.66	0.012168593	*zinc finger (C2H2 type) family protein*	Red ontogenesis	
ppa019579m	-1.66	0.006611377	*DnaJ/Hsp40 cysteine-rich domain superfamily protein*	Red ontogenesis	
ppa007662m	-1.66	0.016940456	*D-aminoacid aminotransferase-like PLP-dependent enzymes sup*	Red ontogenesis	
ppb020590m	-1.66	0.046684013	*Galactose oxidase/kelch repeat superfamily protein*	Red ontogenesis	
ppa021193m	-1.66	0.033966555	*Ubiquitin-specific protease family C19-related protein*	Red ontogenesis	
ppa024677m	-1.66	0.003871948	*TRICHOME BIREFRINGENCE-LIKE 7*	Red ontogenesis	

ppa001639m	-1.66	0.006449783	isoamylase 3	Red ontogenesis	
ppa004406m	-1.66	0.016546976	cytochrome P450, family 98, subfamily A, polypeptide 3	Red ontogenesis	
ppa004550m	-1.66	0.017373776	MATE efflux family protein	Red ontogenesis	
ppa005071m	-1.66	0.002816592	IQ-domain 2	Red ontogenesis	
ppa017564m	-1.66	0.017183892	hydroxyproline-rich glycoprotein family protein	Red ontogenesis	
ppa011922m	-1.66	0.008464523	Expressed protein	Red ontogenesis	
ppa013054m	-1.67	0.020778806	acyl carrier protein 4	Red ontogenesis	
ppa010884m	-1.67	0.004382613	photosystem II subunit Q-2	Red ontogenesis	Photosynthesis / Photosystem II
ppa010554m	-1.67	0.018216987	RING/U-box superfamily protein	Red ontogenesis	
ppa000611m	-1.67	0.00392123	cellulose synthase 1	Red ontogenesis	
ppa000550m	-1.67	0.012484154	Leucine-rich receptor-like protein kinase family protein	Red ontogenesis	
ppa000827m	-1.67	0.00741948	Glycosyl transferase, family 35	Red ontogenesis	
ppa006235m	-1.67	0.007555365	zinc finger (Ran-binding) family protein	Red ontogenesis	
ppa014982m	-1.67	0.041309187	Pectin lyase-like superfamily protein	Red ontogenesis	
ppa000513m	-1.67	0.0107225	RNA-dependent RNA polymerase 2	Red ontogenesis	
ppa010195m	-1.68	0.000856062	light harvesting complex photosystem II subunit 6	Red ontogenesis	Photosynthesis / Photosystem II
ppa023197m	-1.68	0.040734329	NAC 014	Red ontogenesis	
ppa008743m	-1.68	0.000117203	Chitinase family protein	Red ontogenesis	
ppa002445m	-1.68	0.001848303	P-loop containing nucleoside triphosphate hydrolases superfamily	Red ontogenesis	
ppa006815m	-1.68	0.019172523	Protein of unknown function (DUF581)	Red ontogenesis	
ppa007147m	-1.68	0.001497291		Red ontogenesis	
ppa007723m	-1.68	0.010046097	dicarboxylate diiron protein, putative (Crd1)	Red ontogenesis	
ppb024555m	-1.68	0.02130865	Myzus persicae-induced lipase 1	Red ontogenesis	
ppa001029m	-1.68	0.00485668	Leucine-rich repeat protein kinase family protein	Red ontogenesis	
ppa018989m	-1.69	0.024059649		Red ontogenesis	
ppa012103m	-1.69	0.000259205	alpha/beta-Hydrolases superfamily protein	Red ontogenesis	
ppa006284m	-1.69	6.42E-05	Protein of Unknown Function (DUF239)	Red ontogenesis	
ppa016276m	-1.69	0.001693435	PEP1 receptor 1	Red ontogenesis	
ppa013745m	-1.69	0.005640154	Yippee family putative zinc-binding protein	Red ontogenesis	
ppa008026m	-1.69	0.002666054	Plant protein 1589 of unknown function	Red ontogenesis	
ppa017128m	-1.69	0.04370945	metacaspase 1	Red ontogenesis	
ppa012982m	-1.70	0.031066651		Red ontogenesis	
ppa010719m	-1.70	0.013774179	elongation factor P (EF-P) family protein	Red ontogenesis	
ppa021993m	-1.70	0.049254438	chitinase A	Red ontogenesis	
ppa013405m	-1.70	0.025879558	RNA-binding (RRM/RBD/RNP motifs) family protein	Red ontogenesis	
ppa000901m	-1.70	0.018119803	ubiquitin-specific protease 15	Red ontogenesis	
ppa000811m	-1.70	0.009554477	poly(ADP-ribose) polymerase 2	Red ontogenesis	
ppa006875m	-1.70	0.000395363	NAD(P)-binding Rossmann-fold superfamily protein	Red ontogenesis	
ppa002387m	-1.70	0.002906332	Exostosin family protein	Red ontogenesis	
ppa003792m	-1.70	0.003812056		Red ontogenesis	
ppa025097m	-1.71	0.024318044	isoamylase 1	Red ontogenesis	
ppa002621m	-1.71	0.002899257	CTP synthase family protein	Red ontogenesis	
ppa002884m	-1.71	0.000294607	basic helix-loop-helix (bHLH) DNA-binding superfamily protein	Red ontogenesis	
ppa005599m	-1.71	0.009922968	Pectin lyase-like superfamily protein	Red ontogenesis	
ppa006774m	-1.71	0.000289256	Protein of unknown function (DUF506)	Red ontogenesis	
ppa007525m	-1.72	0.000129738	PLC-like phosphodiesterases superfamily protein	Red ontogenesis	
ppa022005m	-1.72	0.030771502	S-adenosyl-L-methionine-dependent methyltransferases superfar	Red ontogenesis	
ppa000429m	-1.72	0.049262532	catalytics;carbohydrate kinases;phosphoglucan, water dikinases	Red ontogenesis	
ppa021573m	-1.72	0.013398025	Auxin efflux carrier family protein	Red ontogenesis	
ppa001183m	-1.72	0.010920823	Forkhead-associated (FHA) domain-containing protein	Red ontogenesis	
ppa003138m	-1.72	0.028620091	Leucine-rich repeat protein kinase family protein	Red ontogenesis	
ppa011777m	-1.72	0.013259039		Red ontogenesis	
ppa020194m	-1.72	0.015682718		Red ontogenesis	
ppa011900m	-1.72	0.013774179	PsbQ-like 1	Red ontogenesis	Photosynthesis / Photosystem II
ppa010291m	-1.73	0.001650287	light-harvesting chlorophyll-protein complex I subunit A4	Red ontogenesis	Photosynthesis / Photosystem II
ppa015949m	-1.73	0.031240316	CYCLIN D3;1	Red ontogenesis	
ppa011261m	-1.73	0.008697794	long-chain acyl-CoA synthetase 2	Red ontogenesis	
ppa005653m	-1.73	0.031283177	lecithin:cholesterol acyltransferase 3	Red ontogenesis	
ppa008578m	-1.73	0.038063763	alpha/beta-Hydrolases superfamily protein	Red ontogenesis	
ppa010157m	-1.73	0.032637063	novel plant snare 11	Red ontogenesis	
ppa007056m	-1.73	0.014301436	squamosa promoter binding protein-like 9	Red ontogenesis	
ppa001800m	-1.73	0.003546386	Subtilase family protein	Red ontogenesis	
ppa003423m	-1.73	0.010153924	terpene synthase 02	Red ontogenesis	Biotic Stress / Secondary metabolism involved into stress
ppa008291m	-1.73	0.009955572	S-adenosyl-L-methionine-dependent methyltransferases superfar	Red ontogenesis	
ppa007107m	-1.73	0.018745087	GDSL-like Lipase/Acylhydrolase superfamily protein	Red ontogenesis	
ppa002659m	-1.73	0.003693151	mechanosensitive channel of small conductance-like 10	Red ontogenesis	
ppa025825m	-1.73	0.022300028		Red ontogenesis	
ppa012425m	-1.73	0.006128419	photosystem II family protein	Red ontogenesis	Photosynthesis / Photosystem II
ppa007149m	-1.74	0.005317733	alpha/beta-Hydrolases superfamily protein	Red ontogenesis	
ppa012061m	-1.74	0.032573023	tRNA (guanine-N-7) methyltransferase	Red ontogenesis	
ppa017562m	-1.74	0.01563522	AGAMOUS-like 30	Red ontogenesis	
ppa010905m	-1.74	0.037717001	Plant-specific transcription factor YABBY family protein	Red ontogenesis	
ppa003464m	-1.74	0.001518472	Plasma-membrane choline transporter family protein	Red ontogenesis	
ppa002919m	-1.74	0.004118209	Plant protein of unknown function (DUF828)	Red ontogenesis	
ppa000209m	-1.75	0.002670574	Pyruvate phosphate dikinase, PEP/pyruvate binding domain	Red ontogenesis	
ppa002670m	-1.75	0.000519765	terpene synthase 14	Red ontogenesis	Biotic Stress / Secondary metabolism involved into stress
ppa010901m	-1.75	0.006136744	Nucleic acid-binding, OB-fold-like protein	Red ontogenesis	
ppa024644m	-1.75	0.00488075	NB-ARC domain-containing disease resistance protein	Red ontogenesis	
ppa012823m	-1.75	0.018778467	photosystem II 11 kDa protein-related	Red ontogenesis	Photosynthesis / Photosystem II
ppa021028m	-1.75	0.039981151	UDP-N-acetylglucosamine (UAA) transporter family	Red ontogenesis	
ppa010388m	-1.76	0.020202578	TRICHOME BIREFRINGENCE-LIKE 41	Red ontogenesis	
ppa026007m	-1.76	0.015041045	RING/U-box superfamily protein	Red ontogenesis	
ppa000789m	-1.76	0.042795157	FTSH protease 12	Red ontogenesis	
ppa007609m	-1.76	0.017808446	Mitochondrial transcription termination factor family protein	Red ontogenesis	
ppa013885m	-1.76	0.031582116		Red ontogenesis	
ppa001746m	-1.76	0.033287205	Leucine-rich repeat protein kinase family protein	Red ontogenesis	
ppa002955m	-1.76	0.019987345	UDP-Glycosyltransferase superfamily protein	Red ontogenesis	
ppa001542m	-1.76	0.00963027	SET-domain containing protein lysine methyltransferase family pr	Red ontogenesis	
ppa000846m	-1.76	0.005358359	casein lytic proteinase B4	Red ontogenesis	
ppa024797m	-1.76	0.028466957	acyl carrier protein 4	Red ontogenesis	
ppa004124m	-1.76	0.016236996	Protein kinase superfamily protein	Red ontogenesis	
ppa009462m	-1.77	0.009454728	glyoxalase I homolog	Red ontogenesis	
ppa008515m	-1.77	0.005300417	Pseudouridine synthase family protein	Red ontogenesis	
ppa024164m	-1.77	0.02724031	Protein kinase superfamily protein	Red ontogenesis	
ppa004864m	-1.77	0.011145239	Protein of unknown function (DUF3754)	Red ontogenesis	
ppa010067m	-1.77	0.046851546	expansin A13	Red ontogenesis	
ppa016640m	-1.77	0.00011592	HXXXD-type acyl-transferase family protein	Red ontogenesis	Secondary Metabolism / Phenylpropanoids - Biotic Stress / Secondary metabolism involved into stress
ppa020264m	-1.77	0.008367598	BURP domain-containing protein	Red ontogenesis	
ppa008899m	-1.77	0.000424261	NAD(P)-linked oxidoreductase superfamily protein	Red ontogenesis	
ppa003438m	-1.77	0.008277044	DNAJ heat shock N-terminal domain-containing protein	Red ontogenesis	
ppa004576m	-1.77	0.005524294	Flavin containing amine oxidoreductase family	Red ontogenesis	
ppa013528m	-1.78	0.001093913	hydroxyproline-rich glycoprotein family protein	Red ontogenesis	
ppa018863m	-1.78	0.000707766	thylakoid rhodanese-like	Red ontogenesis	
ppa005446m	-1.78	0.028811844	Eukaryotic aspartyl protease family protein	Red ontogenesis	
ppa001207m	-1.78	3.72E-05	PLAT/LH2 domain-containing lipoxygenase family protein	Red ontogenesis	
ppa022175m	-1.78	0.000267819	FAD/NAD(P)-binding oxidoreductase family protein	Red ontogenesis	Biotic Stress / Secondary metabolism involved into stress
ppa003983m	-1.78	0.00434126	Kinase interacting (KIP1-like) family protein	Red ontogenesis	
ppa010846m	-1.79	0.014638549	myb domain protein 4	Red ontogenesis	
ppa002568m	-1.79	0.001489948	long-chain acyl-CoA synthetase 2	Red ontogenesis	
ppa010354m	-1.79	0.001008502	Haloacid dehalogenase-like hydrolase (HAD) superfamily protein	Red ontogenesis	
ppa021705m	-1.79	0.034003151	nodulin MtN21 /EamA-like transporter family protein	Red ontogenesis	
ppa024768m	-1.79	0.020219874	UDP-Glycosyltransferase superfamily protein	Red ontogenesis	
ppa001736m	-1.80	0.007567027	Pentatricopeptide repeat (PPR) superfamily protein	Red ontogenesis	
ppa007216m	-1.80	0.012147783	galacturonosyltransferase-like 7	Red ontogenesis	
ppa007778m	-1.80	0.001601605	NAD(P)-binding Rossmann-fold superfamily protein	Red ontogenesis	
ppa010073m	-1.80	0.019885165	6-phosphogluconolactonase 1	Red ontogenesis	
ppa026592m	-1.80	0.031857464	Leucine-rich repeat transmembrane protein kinase protein	Red ontogenesis	
ppa005114m	-1.80	0.004075765	Sulfite exporter TauE/SafE family protein	Red ontogenesis	
ppa007586m	-1.80	0.016485509	Undecaprenyl pyrophosphate synthetase family protein	Red ontogenesis	
ppa010611m	-1.80	0.00812136	ARF-GAP domain 15	Red ontogenesis	
ppa018681m	-1.80	0.000110757	galacturonosyltransferase 4	Red ontogenesis	
ppa002700m	-1.81	0.017412232	purple acid phosphatase 27	Red ontogenesis	
ppa013847m	-1.81	0.046812545	SAUR-like auxin-responsive protein family	Red ontogenesis	
ppa002843m	-1.81	0.032252498	O-Glycosyl hydrolases family 17 protein	Red ontogenesis	Biotic Stress / Betaglucanase
ppa003234m	-1.81	0.001268614	DC1 domain-containing protein	Red ontogenesis	
ppa002434m	-1.82	0.008277044	Nucleic acid-binding, OB-fold-like protein	Red ontogenesis	
ppa009254m	-1.82	0.002242913	indoleacetic acid-induced protein 16	Red ontogenesis	
ppa008395m	-1.82	0.026304035	casein kinase II, alpha chain 2	Red ontogenesis	
ppa023177m	-1.82	0.001605538	UDP-Glycosyltransferase superfamily protein	Red ontogenesis	
ppa003095m	-1.82	0.027615047	Phototropic-responsive NPH3 family protein	Red ontogenesis	
ppa000872m	-1.82	0.043783435	Protein kinase superfamily protein with octicosapeptide/Phox/Bem	Red ontogenesis	
ppa005063m	-1.82	0.000668676	VALINE-TOLERANT 1	Red ontogenesis	
ppa013844m	-1.82	0.015485796	SAUR-like auxin-responsive protein family	Red ontogenesis	
ppa011210m	-1.82	0.007305832	Polyketide cyclase/dehydrase and lipid transport superfamily prot	Red ontogenesis	
ppa009387m	-1.83	0.036504128	xyloglucan endotransglucosylase/hydrolase 5	Red ontogenesis	
ppa004757m	-1.83	0.000113413	Exostosin family protein	Red ontogenesis	
ppa009903m	-1.83	0.011546638	photosystem I light harvesting complex gene 2	Red ontogenesis	Photosynthesis / Photosystem II
ppa010953m	-1.83	0.001302458	photosystem I subunit F	Red ontogenesis	Photosynthesis / Photosystem I
ppa008378m	-1.83	0.001494616	S-adenosyl-L-methionine-dependent methyltransferases superfar	Red ontogenesis	
ppa003073m	-1.83	0.001083272	Peptide-N4-(N-acetyl-beta-glucosaminyl)asparagine amidase A p	Red ontogenesis	
ppa000927m	-1.83	0.000426655	heteroglycan glucosidase 1	Red ontogenesis	
ppa004919m	-1.84	0.002564105	zinc induced facilitator-like 1	Red ontogenesis	
ppa013612m	-1.84	0.014526299		Red ontogenesis	
ppa023463m	-1.84	0.008553751	Fasciclin-like arabinogalactan family protein	Red ontogenesis	
ppa002528m	-1.84	0.005801476	Auxin efflux carrier family protein	Red ontogenesis	
ppa000317m	-1.84	0.009748627	transcription activators	Red ontogenesis	
ppa001107m	-1.84	0.000128632	Myosin heavy chain-related protein	Red ontogenesis	
ppa004101m	-1.84	0.026250832	BURP domain-containing protein	Red ontogenesis	

ppa004334m	-1.84	0.000185002	beta-amylase 6	Red ontogenesis	
ppa004996m	-1.85	8.92E-05	Pectin lyase-like superfamily protein	Red ontogenesis	
ppa025573m	-1.85	0.003997117		Red ontogenesis	
ppa000780m	-1.85	0.042490029	male gametophyte defective 3	Red ontogenesis	
ppa015874m	-1.85	0.001257336		Red ontogenesis	
ppb017681m	-1.86	0.001246379	serine carboxypeptidase-like 18	Red ontogenesis	
ppa012060m	-1.86	0.000175287	Bifunctional inhibitor/lipid-transfer protein/seed storage 2S albumi	Red ontogenesis	
ppa002832m	-1.86	0.013228137	ROP interactive partner 5	Red ontogenesis	
ppa018715m	-1.86	0.023360248	Sulfite exporter TauE/SafE family protein	Red ontogenesis	
ppa003613m	-1.87	0.02934444	hydroxy methylglutaryl CoA reductase 1	Red ontogenesis	Biotic Stress / Secondary metabolism involved into stress
ppa006000m	-1.87	0.002372946	arogenate dehydratase 6	Red ontogenesis	
ppa004627m	-1.87	0.00068994	Exostosin family protein	Red ontogenesis	
ppa009382m	-1.87	0.005522857	long-chain acyl-CoA synthetase 2	Red ontogenesis	
ppa000515m	-1.87	0.012901883	CRM family member 2	Red ontogenesis	
ppa018930m	-1.87	0.019187639	GCK domain-containing protein	Red ontogenesis	
ppa004044m	-1.87	0.013384922	Xanthine/uracil permease family protein	Red ontogenesis	
ppa010553m	-1.87	0.027026669		Red ontogenesis	
ppa001669m	-1.87	0.001502683	phosphatidylinositol-4-phosphate 5-kinase 1	Red ontogenesis	
ppa005399m	-1.87	0.001354844	Plant protein of unknown function (DUF828) with plant pleckstrin	Red ontogenesis	
ppa009915m	-1.87	0.021695924	RNA-binding (RRM/RBD/RNP motifs) family protein	Red ontogenesis	
ppa003515m	-1.87	0.034694376	Mitochondrial transcription termination factor family protein	Red ontogenesis	
ppa002582m	-1.88	0.003789459	Pentatricopeptide repeat (PPR) superfamily protein	Red ontogenesis	
ppa004440m	-1.88	0.017839447	Protein phosphatase 2A regulatory B subunit family protein	Red ontogenesis	
ppa023358m	-1.88	0.024230557	Protein of unknown function (DUF579)	Red ontogenesis	
ppa018841m	-1.88	0.040921025	Protein kinase superfamily protein	Red ontogenesis	
ppa015700m	-1.89	0.02050995	TRICHOME BIREFRINGENCE-LIKE 6	Red ontogenesis	
ppa008126m	-1.89	0.036844983	beta-1,3-glucanase 1	Red ontogenesis	Biotic Stress / Betaglucanase
ppa011749m	-1.89	0.022330923	Polynucleotidyl transferase, ribonuclease H-like superfamily prote	Red ontogenesis	
ppa000113m	-1.89	0.011502509	Transcription factor jumonji (jmjC) domain-containing protein	Red ontogenesis	
ppa007237m	-1.89	0.000476179	Nucleotide/sugar transporter family protein	Red ontogenesis	
ppa021930m	-1.89	0.010239871		Red ontogenesis	
ppa022282m	-1.90	0.032758286		Red ontogenesis	
ppa010426m	-1.90	0.004310017	ascorbate peroxidase 2	Red ontogenesis	
ppa011732m	-1.90	0.009401276	NDH-dependent cyclic electron flow 1	Red ontogenesis	
ppa018100m	-1.90	0.012971811	IQ-domain 21	Red ontogenesis	
ppa007960m	-1.90	0.020136129	2-oxoglutarate (2OG) and Fe(II)-dependent oxygenase superfami	Red ontogenesis	
ppa027130m	-1.90	0.049121533	Acyl-CoA N-acyltransferase with RING/FYVE/PHD-type zinc finge	Red ontogenesis	
ppa017228m	-1.90	0.025921252	phytochrome interacting factor 4	Red ontogenesis	
ppa014503m	-1.90	0.0028218	arabinogalactan protein 15	Red ontogenesis	
ppa006034m	-1.90	0.013887752	Transmembrane amino acid transporter family protein	Red ontogenesis	
ppa000400m	-1.90	0.013442103	gamma-irradiation and mitomycin c induced 1	Red ontogenesis	
ppa027105m	-1.90	0.000708753	Myzus persicae-induced lipase 1	Red ontogenesis	
ppa016019m	-1.91	0.016579659	Ribosomal protein L21	Red ontogenesis	
ppa010249m	-1.91	0.003012948	K-box region and MADS-box transcription factor family protein	Red ontogenesis	
ppa017766m	-1.91	0.022554861	Protein kinase superfamily protein	Red ontogenesis	
ppa024837m	-1.91	0.003508762		Red ontogenesis	
ppa000185m	-1.92	0.00255949	pleiotropic drug resistance 11	Red ontogenesis	
ppa018071m	-1.92	0.000233194		Red ontogenesis	
ppa004666m	-1.92	0.00044163	Major facilitator superfamily protein	Red ontogenesis	
ppa006845m	-1.92	0.005465623	Oxidoreductase, zinc-binding dehydrogenase family protein	Red ontogenesis	
ppa009588m	-1.93	4.67E-05	plasma membrane intrinsic protein 1B	Red ontogenesis	
ppa011206m	-1.93	0.004662679	Leucine-rich repeat (LRR) family protein	Red ontogenesis	
ppa023231m	-1.93	0.000316123		Red ontogenesis	
ppa002125m	-1.93	0.005500543	Concanavalin A-like lectin protein kinase family protein	Red ontogenesis	
ppa017932m	-1.93	0.005422222	2-oxoglutarate (2OG) and Fe(II)-dependent oxygenase superfami	Red ontogenesis	
ppa005901m	-1.93	0.023732349	Amino acid permease family protein	Red ontogenesis	
ppa001194m	-1.93	0.002093803	Leucine-rich repeat protein kinase family protein	Red ontogenesis	
ppa000942m	-1.93	0.0012313	transmembrane kinase 1	Red ontogenesis	
ppa005403m	-1.93	0.026470668	methyltransferases;copper ion binding	Red ontogenesis	
ppa002869m	-1.93	0.005701619	glycosyltransferase family protein 47	Red ontogenesis	
ppa001386m	-1.94	0.025184352	homeobox gene 8	Red ontogenesis	
ppa021231m	-1.94	0.025845323	F-box and associated interaction domains-containing protein	Red ontogenesis	
ppa005263m	-1.94	0.003279768	RNI-like superfamily protein	Red ontogenesis	
ppa007057m	-1.95	0.0073886	fatty acid desaturase 8	Red ontogenesis	
ppa026719m	-1.95	0.020535756	hydroxyproline-rich glycoprotein family protein	Red ontogenesis	
ppa004399m	-1.95	0.033425607	cyclic nucleotide gated channel 1	Red ontogenesis	
ppa004073m	-1.95	0.005436103	plastid transcriptionally active 12	Red ontogenesis	
ppa023800m	-1.96	0.038148063		Red ontogenesis	
ppa009496m	-1.96	0.006701597	light harvesting complex of photosystem II 5	Red ontogenesis	Photosynthesis / Photosystem II
ppb012525m	-1.96	0.048550538	BTB/POZ domain-containing protein	Red ontogenesis	
ppa004803m	-1.96	0.006329689	lipoamide dehydrogenase 1	Red ontogenesis	
ppa009107m	-1.96	2.84E-05	NAD(P)-binding Rossmann-fold superfamily protein	Red ontogenesis	Secondary Metabolism / Dihydroflavonols -Biotic Stress / Secondary metabolism involved into stress
ppa000878m	-1.96	0.014559757	polymerase gamma 2	Red ontogenesis	
ppa023795m	-1.96	0.002933894	Zinc-binding alcohol dehydrogenase family protein	Red ontogenesis	
ppa008741m	-1.96	0.011799024		Red ontogenesis	
ppa004192m	-1.97	0.025170701	Glucose-1-phosphate adenylyltransferase family protein	Red ontogenesis	
ppa014749m	-1.97	0.018505116		Red ontogenesis	
ppa011438m	-1.97	6.55E-05	NDR1/HIN1-like 1	Red ontogenesis	
ppa011428m	-1.97	0.044995525	Receptor-like protein kinase-related family protein	Red ontogenesis	
ppa002092m	-1.97	0.000268829	STRUBBELIG-receptor family 6	Red ontogenesis	
ppa003008m	-1.97	0.000314837	glucan synthase-like 12	Red ontogenesis	
ppa009771m	-1.97	0.003282573	RING/U-box superfamily protein	Red ontogenesis	
ppa016622m	-1.97	0.015340766	cyclic nucleotide gated channel 1	Red ontogenesis	
ppa004429m	-1.98	0.000205502	Protein kinase superfamily protein	Red ontogenesis	
ppa001591m	-1.98	0.000336475	IQ-domain 32	Red ontogenesis	
ppa005184m	-1.98	0.03359972	rubisco activase	Red ontogenesis	
ppa014897m	-1.98	0.046283385	basic helix-loop-helix (bHLH) DNA-binding superfamily protein	Red ontogenesis	
ppa000652m	-1.98	0.017229772	phytosylfokine-alpha receptor 2	Red ontogenesis	
ppa010872m	-1.98	0.001462275	nucleoside diphosphate kinase 2	Red ontogenesis	
ppa005487m	-1.98	0.015988393	Transducin/WD40 repeat-like superfamily protein	Red ontogenesis	
ppa005824m	-1.98	0.003789459		Red ontogenesis	
ppa007039m	-1.99	0.004822938	Protein kinase superfamily protein	Red ontogenesis	
ppa023408m	-1.99	0.018319876	Tetratricopeptide repeat (TPR)-like superfamily protein	Red ontogenesis	
ppa007228m	-1.99	0.000159011	actin-11	Red ontogenesis	
ppa018548m	-1.99	0.006140317	Protein phosphatase 2C family protein	Red ontogenesis	
ppa000971m	-1.99	0.000341783	ARM repeat superfamily protein	Red ontogenesis	
ppa009901m	-1.99	0.00369261	myo-inositol oxygenase 1	Red ontogenesis	
ppa011831m	-2.00	0.03078297	Plant invertase/pectin methylesterase inhibitor superfamily protei	Red ontogenesis	
ppa005780m	-2.00	5.32E-05	Uridine diphosphate glycosyltransferase 74E2	Red ontogenesis	
ppa021099m	-2.00	0.000698368	BCL-2-associated athanogene 1	Red ontogenesis	
ppa001064m	-2.00	0.001666915	PLAT/LH2 domain-containing lipoxygenase family protein	Red ontogenesis	
ppa000341m	-2.00	0.00097974	kinesin-like calmodulin-binding protein (ZWICHEL)	Red ontogenesis	
ppa024006m	-2.00	0.019505645	Emsy N Terminus (ENT)/ plant Tudor-like domains-containing pro	Red ontogenesis	
ppa011433m	-2.00	0.000333945		Red ontogenesis	
ppa004672m	-2.00	0.027873886	Eukaryotic aspartyl protease family protein	Red ontogenesis	
ppa023648m	-2.01	0.004564772		Red ontogenesis	
ppa012166m	-2.01	0.033888919	Uncharacterised protein family (UPF0497)	Red ontogenesis	
ppa011347m	-2.01	0.006341646		Red ontogenesis	
ppa007994m	-2.01	0.000447591	NAD(P)-binding Rossmann-fold superfamily protein	Red ontogenesis	Secondary Metabolism / Flavonols-Isoflavonoid -Biotic Stress / Secondary metabolism involved into stress
ppa003672m	-2.01	0.004957424	Plant protein of unknown function (DUF828)	Red ontogenesis	
ppa007794m	-2.01	0.036287922	galacturonosyltransferase-like 3	Red ontogenesis	
ppa000735m	-2.01	0.004205445		Red ontogenesis	
ppa010963m	-2.02	0.00410398	Thioredoxin superfamily protein	Red ontogenesis	
ppa008023m	-2.02	0.018778618	EXORDIUM like 3	Red ontogenesis	
ppa008577m	-2.03	0.040921025	Peroxidase superfamily protein	Red ontogenesis	
ppa007965m	-2.03	0.000115165	Zinc-binding dehydrogenase family protein	Red ontogenesis	
ppa003956m	-2.03	0.00958753	XS domain-containing protein / XS zinc finger domain-containing	Red ontogenesis	
ppa016486m	-2.03	0.007367089	UDP-glucosyltransferase 74F2	Red ontogenesis	
ppa001056m	-2.03	0.006691776	Leucine-rich repeat protein kinase family protein	Red ontogenesis	
ppa010394m	-2.03	0.003847505	Nodulin MtN3 family protein	Red ontogenesis	
ppa010357m	-2.03	0.001246915	rotamase CYP 4	Red ontogenesis	
ppa003381m	-2.03	0.029340224		Red ontogenesis	
ppa020872m	-2.03	0.009037863	Heavy metal transport/detoxification superfamily protein	Red ontogenesis	
ppa016424m	-2.04	0.006172345	Agenet domain-containing protein	Red ontogenesis	
ppa003887m	-2.04	0.005006008	Phototropic-responsive NPH3 family protein	Red ontogenesis	
ppa007571m	-2.04	0.011145239	RING/U-box superfamily protein	Red ontogenesis	
ppa000905m	-2.04	0.003673355	limit dextrinase	Red ontogenesis	
ppa003531m	-2.04	0.005602333	Protein with RING/U-box and TRAF-like domains	Red ontogenesis	
ppa011099m	-2.04	0.00175258	Domain of unknown function (DUF1995)	Red ontogenesis	
ppa026887m	-2.04	0.015641432	ferritin 2	Red ontogenesis	
ppa013214m	-2.04	0.011143944		Red ontogenesis	
ppa002610m	-2.04	0.021431472	Protein of unknown function (DUF668)	Red ontogenesis	
ppa002531m	-2.04	2.53E-05	AMP-dependent synthetase and ligase family protein	Red ontogenesis	
ppa008158m	-2.05	0.00434126	nodulin MtN21 /EamA-like transporter family protein	Red ontogenesis	
ppa007506m	-2.05	0.002299488	Pectinacetylesterase family protein	Red ontogenesis	
ppa012248m	-2.05	0.004594514	early nodulin-like protein 17	Red ontogenesis	
ppa025751m	-2.05	0.024951919		Red ontogenesis	
ppa012668m	-2.05	0.000510246		Red ontogenesis	
ppa004028m	-2.05	0.000400095	Leucine-rich repeat protein kinase family protein	Red ontogenesis	
ppa009168m	-2.05	0.037769373	Protein of unknown function (DUF579)	Red ontogenesis	
ppa012892m	-2.05	0.001504559	UDP-Glycosyltransferase superfamily protein	Red ontogenesis	
ppa006990m	-2.05	8.51E-06	Serine protease inhibitor (SERPIN) family protein	Red ontogenesis	
ppa000837m	-2.06	0.031228258	Leucine-rich receptor-like protein kinase family protein	Red ontogenesis	
ppa017342m	-2.06	0.009626372		Red ontogenesis	
ppa008969m	-2.06	0.001923286	bifunctional nuclease i	Red ontogenesis	
ppa004730m	-2.06	0.000275079	O-acetyltransferase family protein	Red ontogenesis	
ppa016143m	-2.06	0.042653848		Red ontogenesis	
ppa006114m	-2.07	0.001716833	Pectinacetylesterase family protein	Red ontogenesis	

ppa010066m	-2.07	7.66E-05	*chlorophyll A/B binding protein 1*	Red ontogenesis	Photosynthesis / Photosystem II
ppa003414m	-2.07	0.001006336	*Glucose-methanol-choline (GMC) oxidoreductase family protein*	Red ontogenesis	
ppa005096m	-2.07	0.000258512	*Protein of unknown function (DUF604)*	Red ontogenesis	
ppa020728m	-2.08	0.00132617	*Protein of unknown function, DUF538*	Red ontogenesis	
ppa008617m	-2.08	0.007228792	*Vacuolar iron transporter (VIT) family protein*	Red ontogenesis	
ppa004083m	-2.08	0.01643525	*cytochrome P450, family 78, subfamily A, polypeptide 10*	Red ontogenesis	
ppa013448m	-2.08	0.016546976	*cyclic nucleotide gated channel 3*	Red ontogenesis	
ppa002710m	-2.08	0.015472131	*auxin response factor 16*	Red ontogenesis	
ppa003193m	-2.08	0.001259857	*Major facilitator superfamily protein*	Red ontogenesis	
ppa007518m	-2.08	0.000441618	*high mobility group B1*	Red ontogenesis	
ppa016194m	-2.09	0.006239321	*receptor lectin kinase*	Red ontogenesis	
ppa009043m	-2.09	0.003237251		Red ontogenesis	
ppa019480m	-2.09	0.006131338	*osmotin 34*	Red ontogenesis	
ppa010124m	-2.09	0.000717764	*ortholog of human splicing factor SC35*	Red ontogenesis	
ppa010460m	-2.09	0.042910504	*RING/U-box superfamily protein*	Red ontogenesis	
ppa009120m	-2.09	9.46E-05	*shikimate kinase 1*	Red ontogenesis	
ppa013712m	-2.09	0.006694789	*Photosystem II 5 kD protein*	Red ontogenesis	
ppa019454m	-2.09	0.009423108	*Calcium-binding EF-hand family protein*	Red ontogenesis	
ppa026283m	-2.10	0.01900871		Red ontogenesis	
ppa010144m	-2.10	0.007915733	*RING/FYVE/PHD zinc finger superfamily protein*	Red ontogenesis	
ppa009259m	-2.10	0.021671934	*NagB/RpiA/CoA transferase-like superfamily protein*	Red ontogenesis	
ppa002537m	-2.10	0.001946846	*Zinc finger (C3HC4-type RING finger) family protein*	Red ontogenesis	
ppa021898m	-2.10	0.003408975	*Leucine-rich receptor-like protein kinase family protein*	Red ontogenesis	
ppa009686m	-2.11	0.0012313	*light harvesting complex photosystem II*	Red ontogenesis	Photosynthesis / Photosystem II
ppa023322m	-2.11	0.027633525	*nuclear RNA polymerase C1*	Red ontogenesis	
ppa006194m	-2.11	0.007008682	*cytochrome P450, family 77, subfamily A, polypeptide 5 pseudoge*	Red ontogenesis	
ppa011829m	-2.11	0.012044362	*oxidoreductases, acting on the aldehyde or oxo group of donors, I*	Red ontogenesis	
ppa000559m	-2.11	0.000316123	*cellulose synthase A9*	Red ontogenesis	
ppa005895m	-2.11	0.004147497	*Class I glutamine amidotransferase-like superfamily protein*	Red ontogenesis	
ppa025458m	-2.12	0.001545101	*Embryo-specific protein 3, (ATS3)*	Red ontogenesis	
ppa010373m	-2.12	0.022948314	*response regulator 9*	Red ontogenesis	
ppa018145m	-2.12	0.024668936	*high mobility group A*	Red ontogenesis	
ppa023080m	-2.12	0.034262158	*Chalcone and stilbene synthase family protein*	Red ontogenesis	Biotic Stress / Secondary metabolism involved into stress
ppa000958m	-2.12	0.022873025	*Glycosyl transferase, family 35*	Red ontogenesis	
ppa011590m	-2.13	0.015686921	*adenylate cyclases*	Red ontogenesis	
ppa008723m	-2.13	0.017800596	*Peroxidase superfamily protein*	Red ontogenesis	
ppa003273m	-2.13	0.020310214	*Transducin/WD40 repeat-like superfamily protein*	Red ontogenesis	
ppa011630m	-2.13	0.007884655	*PLATZ transcription factor family protein*	Red ontogenesis	
ppa006648m	-2.13	0.025481959	*Tetratricopeptide repeat (TPR)-like superfamily protein*	Red ontogenesis	
ppa017177m	-2.13	0.00864047	*Protein of unknown function (DUF1191)*	Red ontogenesis	
ppa000868m	-2.13	0.011257337	*DNA/RNA polymerases superfamily protein*	Red ontogenesis	
ppa003352m	-2.13	0.002578537	*MATE efflux family protein*	Red ontogenesis	
ppa002216m	-2.13	0.031544637	*Plasma-membrane choline transporter family protein*	Red ontogenesis	
ppa011991m	-2.13	0.014432958		Red ontogenesis	
ppa000819m	-2.13	0.004755357	*Plant protein of unknown function (DUF869)*	Red ontogenesis	
ppa025949m	-2.13	0.007931315	*U2 snRNP auxiliary factor small subunit, putative*	Red ontogenesis	
ppa013480m	-2.14	0.036331167	*GAST1 protein homolog 1*	Red ontogenesis	
ppa022951m	-2.14	0.010701121	*receptor serine/threonine kinase, putative*	Red ontogenesis	
ppa003813m	-2.14	0.002522943	*glucuronidase 2*	Red ontogenesis	
ppa026726m	-2.14	0.040020784	*D-isomer specific 2-hydroxyacid dehydrogenase family protein*	Red ontogenesis	
ppa015744m	-2.14	0.007198338		Red ontogenesis	
ppa007636m	-2.14	3.48E-05	*flavanone 3-hydroxylase*	Red ontogenesis	
ppa019046m	-2.15	0.042869907	*Protein of unknown function, DUF593*	Red ontogenesis	
ppa018683m	-2.15	0.021821166		Red ontogenesis	
ppa003264m	-2.15	0.001269265	*Protein kinase superfamily protein*	Red ontogenesis	
ppa001410m	-2.15	0.001652187	*Protein kinase superfamily protein*	Red ontogenesis	
ppa010226m	-2.15	0.022134394	*Barwin-like endoglucanases superfamily protein*	Red ontogenesis	
ppa024172m	-2.16	0.00131838	*COBRA-like extracellular glycosyl-phosphatidyl inositol-anchored*	Red ontogenesis	
ppa003796m	-2.16	0.005823232	*Phototropic-responsive NPH3 family protein*	Red ontogenesis	
ppa001004m	-2.16	0.011755543	*Pyridoxal phosphate (PLP)-dependent transferases superfamily p*	Red ontogenesis	
ppa003145m	-2.16	0.000174638	*S-adenosyl-L-methionine-dependent methyltransferases superfar*	Red ontogenesis	
ppa023765m	-2.17	0.01254999	*toprim domain-containing protein*	Red ontogenesis	
ppa002586m	-2.17	0.016514754	*sulfate transporter 3;4*	Red ontogenesis	
ppa002059m	-2.17	0.000327783	*UDP-Glycosyltransferase superfamily protein*	Red ontogenesis	
ppa001081m	-2.17	0.004662679	*RECQ helicase L4B*	Red ontogenesis	
ppa000439m	-2.17	0.010910707		Red ontogenesis	
ppa006348m	-2.17	0.000561683	*Trypsin family protein with PDZ domain*	Red ontogenesis	
ppa005287m	-2.17	0.00215539	*Protein of unknown function (DUF620)*	Red ontogenesis	
ppa020832m	-2.18	0.002010972	*Phototropic-responsive NPH3 family protein*	Red ontogenesis	
ppa000968m	-2.18	0.006920646	*lipoxygenase 2*	Red ontogenesis	
ppa005939m	-2.18	0.003812056	*ankyrin repeat family protein*	Red ontogenesis	
ppa026241m	-2.18	0.000577717	*O-fucosyltransferase family protein*	Red ontogenesis	
ppa017046m	-2.18	0.007551581	*Phosphatidylinositol 3- and 4-kinase ;Ubiquitin family protein*	Red ontogenesis	
ppa008030m	-2.18	0.026664286	*6-phosphogluconate dehydrogenase family protein*	Red ontogenesis	
ppa012767m	-2.18	0.019529442	*Protein of unknown function (DUF581)*	Red ontogenesis	
ppa004921m	-2.19	2.09E-05	*O-fucosyltransferase family protein*	Red ontogenesis	
ppa007471m	-2.19	0.007798182		Red ontogenesis	
ppa001355m	-2.19	0.010920823	*Subtilase family protein*	Red ontogenesis	
ppa020308m	-2.19	0.004828845	*RNI-like superfamily protein*	Red ontogenesis	
ppa018057m	-2.19	0.002002004	*zinc finger protein 8*	Red ontogenesis	
ppa009489m	-2.20	0.01131911	*Protein of unknown function (DUF506)*	Red ontogenesis	
ppa002209m	-2.20	0.01704361	*Cellulose-synthase-like C12*	Red ontogenesis	
ppa024942m	-2.20	0.016644912	*F-BOX WITH WD-40 2*	Red ontogenesis	
ppa003629m	-2.20	6.17E-05	*ARM repeat superfamily protein*	Red ontogenesis	
ppa011905m	-2.21	0.017527079	*Maf-like protein*	Red ontogenesis	
ppa006961m	-2.21	0.008450498	*Glycosyltransferase family 29 (sialyltransferase) family protein*	Red ontogenesis	
ppa011294m	-2.21	0.000752075	*RAB GTPase homolog A2B*	Red ontogenesis	
ppa008512m	-2.21	0.002750851	*Homocysteine S-methyltransferase family protein*	Red ontogenesis	
ppa003708m	-2.21	0.008985079	*Protein of unknown function, DUF547*	Red ontogenesis	
ppa024312m	-2.22	0.000255569	*Sec14p-like phosphatidylinositol transfer family protein*	Red ontogenesis	
ppa023740m	-2.22	0.000112576	*Phosphorylase superfamily protein*	Red ontogenesis	
ppa019247m	-2.22	0.000875241	*Protein of unknown function (DUF1637)*	Red ontogenesis	
ppa020340m	-2.22	0.002449262	*Major facilitator superfamily protein*	Red ontogenesis	
ppa017605m	-2.22	0.009699298	*Exostosin family protein*	Red ontogenesis	
ppa008483m	-2.22	0.001066518	*Pathogenesis-related thaumatin superfamily protein*	Red ontogenesis	
ppa025853m	-2.22	0.022622011	*RING/U-box superfamily protein*	Red ontogenesis	
ppa011476m	-2.22	0.005522857	*Chalcone-flavanone isomerase family protein*	Red ontogenesis	Biotic Stress / Secondary metabolism involved into stress
ppb006523m	-2.23	0.001285884	*2-oxoglutarate (2OG) and Fe(II)-dependent oxygenase superfami*	Red ontogenesis	
ppa021654m	-2.23	0.018697233	*NADPH:quinone oxidoreductase*	Red ontogenesis	
ppa008069m	-2.23	5.57E-06	*dihydroflavonol 4-reductase*	Red ontogenesis	Secondary Metabolism / Dihydroflavonols -Biotic Stress / Secondary metabolism involved into stress
ppa002259m	-2.23	0.000695353	*ferric reduction oxidase 7*	Red ontogenesis	
ppa010472m	-2.24	0.003800524	*delta tonoplast integral protein*	Red ontogenesis	
ppa012696m	-2.24	0.010046097		Red ontogenesis	
ppa019227m	-2.24	0.024172005	*2-oxoglutarate (2OG) and Fe(II)-dependent oxygenase superfami*	Red ontogenesis	
ppa003391m	-2.24	3.48E-05	*heat shock protein 60*	Red ontogenesis	
ppa010456m	-2.25	0.000147776	*Heavy metal transport/detoxification superfamily protein*	Red ontogenesis	
ppa024722m	-2.25	0.00120818	*Dynamin related protein 4C*	Red ontogenesis	
ppa010510m	-2.25	0.002501332		Red ontogenesis	
ppa008626m	-2.25	0.025152484		Red ontogenesis	
ppa004896m	-2.25	0.01891609	*SET domain-containing protein*	Red ontogenesis	
ppa016864m	-2.25	0.033219817	*P-loop containing nucleoside triphosphate hydrolases superfamily*	Red ontogenesis	
ppa1027173m	-2.25	0.006634948		Red ontogenesis	
ppa001323m	-2.25	0.023964871	*armadillo repeat kinesin 2*	Red ontogenesis	
ppa023647m	-2.25	0.044641405	*MATE efflux family protein*	Red ontogenesis	
ppa001503m	-2.26	0.003783001	*HEAT SHOCK PROTEIN 89.1*	Red ontogenesis	
ppa026728m	-2.26	0.00256797	*Rhodanese/Cell cycle control phosphatase superfamily protein*	Red ontogenesis	
ppa004938m	-2.26	0.000345791	*2-oxoacid dehydrogenases acyltransferase family protein*	Red ontogenesis	
ppa000573m	-2.26	0.001175924	*Leucine-rich receptor-like protein kinase family protein*	Red ontogenesis	
ppa023179m	-2.26	0.04334396	*Arabidopsis thaliana protein of unknown function (DUF821)*	Red ontogenesis	
ppa003697m	-2.26	0.001335201	*methylesterase PCR A*	Red ontogenesis	
ppa009055m	-2.26	0.000262883	*alpha/beta-Hydrolases superfamily protein*	Red ontogenesis	
ppa003486m	-2.27	0.002940069	*Tetratricopeptide repeat (TPR)-like superfamily protein*	Red ontogenesis	
ppa011697m	-2.27	0.001693435	*nicotinamidase 2*	Red ontogenesis	Secondary Metabolism / Phenylpropanoids - Biotic Stress / Secondary metabolism involved into stress
ppa006760m	-2.27	0.006531312	*Aluminium activated malate transporter family protein*	Red ontogenesis	
ppa025782m	-2.27	0.009722709	*ARF-GAP domain 1*	Red ontogenesis	
ppa024039m	-2.27	0.006036291	*Protein kinase family protein*	Red ontogenesis	
ppa012417m	-2.28	0.000465515	*HVA22 homologue A*	Red ontogenesis	
ppa004251m	-2.28	0.002779296	*heat shock protein 70*	Red ontogenesis	
ppa001942m	-2.28	0.001571188	*DNA GYRASE B2*	Red ontogenesis	
ppa1027193m	-2.29	0.000683154	*TPX2 (targeting protein for Xklp2) protein family*	Red ontogenesis	
ppa000618m	-2.29	0.000784604	*Cellulose synthase family protein*	Red ontogenesis	
ppa000567m	-2.29	0.005754566	*cellulose synthase 6*	Red ontogenesis	
ppa002450m	-2.29	0.002756624	*Leucine-rich repeat protein kinase family protein*	Red ontogenesis	
ppa004783m	-2.29	0.002099365	*O-Glycosyl hydrolases family 17 protein*	Red ontogenesis	Biotic Stress / Betaglucanase
ppa011203m	-2.30	0.014357381	*TPX2 (targeting protein for Xklp2) protein family*	Red ontogenesis	
ppa008396m	-2.30	0.016644912	*cyclin d5;1*	Red ontogenesis	
ppa018535m	-2.30	0.002007359	*AUX/IAA transcriptional regulator family protein*	Red ontogenesis	
ppa016999m	-2.30	0.003488215	*P-loop containing nucleoside triphosphate hydrolases superfamily*	Red ontogenesis	
ppa012806m	-2.30	0.000620015	*HSP20-like chaperones superfamily protein*	Red ontogenesis	
ppa017311m	-2.30	0.001922894	*BTB/POZ domain-containing protein*	Red ontogenesis	
ppa020997m	-2.30	0.017020513	*SPFH/Band 7/PHB domain-containing membrane-associated pro*	Red ontogenesis	
ppa026947m	-2.31	0.000672422	*nitrate excretion transporter1*	Red ontogenesis	
ppa001580m	-2.31	0.005315398	*early-responsive to dehydration stress protein (ERD4)*	Red ontogenesis	
ppa026470m	-2.31	0.002372946	*BTB/POZ domain-containing protein*	Red ontogenesis	
ppa006616m	-2.31	3.73E-05	*non-specific phospholipase C6*	Red ontogenesis	
ppa002368m	-2.31	0.000102704	*YELLOW STRIPE like 7*	Red ontogenesis	
ppa009993m	-2.32	0.003235846	*PapD-like superfamily protein*	Red ontogenesis	
ppa020357m	-2.32	0.001896222	*Frigida-like protein*	Red ontogenesis	

ppa015619m	-2.32	0.001352818	beta glucosidase 40	Red ontogenesis	
ppa001617m	-2.32	0.000314402	STRUBBELIG-receptor family 3	Red ontogenesis	
ppa001692m	-2.32	0.004333354	beta-D-xylosidase 4	Red ontogenesis	
ppa010333m	-2.33	5.23E-05	chorismate mutase 2	Red ontogenesis	
ppa010271m	-2.33	0.000597459	BCL-2-associated athanogene 2	Red ontogenesis	
ppa003017m	-2.34	0.024573009	growth-regulating factor 1	Red ontogenesis	
ppa012082m	-2.34	0.003088111	Thioesterase superfamily protein	Red ontogenesis	
ppa017272m	-2.34	0.006085729	Protein kinase superfamily protein	Red ontogenesis	
ppa002898m	-2.34	0.001160337	Protein of unknown function (DUF3049)	Red ontogenesis	
ppa013016m	-2.35	5.56E-05	multiprotein bridging factor 1C	Red ontogenesis	
ppa005581m	-2.35	0.001041975	HXXXD-type acyl-transferase family protein	Red ontogenesis	
ppa019239m	-2.35	0.008483608	ATP binding microtubule motor family protein	Red ontogenesis	
ppa000557m	-2.36	0.024985323	cellulose synthase 6	Red ontogenesis	
ppa014732m	-2.36	0.00205872	zinc transporter 5 precursor	Red ontogenesis	
ppa009599m	-2.36	1.40E-05	Aldolase-type TIM barrel family protein	Red ontogenesis	
ppa006906m	-2.36	0.000298039	alpha/beta-Hydrolases superfamily protein	Red ontogenesis	
ppa016904m	-2.36	0.009506709	cytochrome P450, family 704, subfamily A, polypeptide 2	Red ontogenesis	
ppa016045m	-2.36	0.032758286	FRIGIDA-like protein	Red ontogenesis	
ppa014455m	-2.36	0.015576413		Red ontogenesis	
ppa003163m	-2.36	0.032573023	Auxin-responsive GH3 family protein	Red ontogenesis	
ppa024543m	-2.37	0.018312682	osmotin 34	Red ontogenesis	
ppa001638m	-2.37	0.000474246	potassium transporter 2	Red ontogenesis	
ppa003590m	-2.37	0.010486349	laccase 11	Red ontogenesis	Biotic Stress / Secondary metabolism involved into stress
ppa004500m	-2.38	0.020589576	3-ketoacyl-CoA synthase 4	Red ontogenesis	
ppa1027229m	-2.38	0.018319876	C2H2-like zinc finger protein	Red ontogenesis	
ppa018533m	-2.39	0.003785388	DNA binding;ATP binding	Red ontogenesis	
ppa017874m	-2.39	0.009127897	PLAC8 family protein	Red ontogenesis	
ppa022290m	-2.39	0.002131497	BRI1-like 2	Red ontogenesis	
ppa023263m	-2.39	0.005397765	Zinc finger (CCCH-type) family protein / RNA recognition motif (R	Red ontogenesis	
ppa002094m	-2.39	0.002691564	Protein kinase protein with adenine nucleotide alpha hydrolases-l	Red ontogenesis	
ppa005616m	-2.39	0.00829426	Eukaryotic aspartyl protease family protein	Red ontogenesis	
ppa008754m	-2.40	0.00227757	alpha/beta-Hydrolases superfamily protein	Red ontogenesis	
ppa002367m	-2.40	0.006448958	tetratricopetide-repeat thioredoxin-like 1	Red ontogenesis	
ppa002995m	-2.40	0.00387322	UDP-Glycosyltransferase superfamily protein	Red ontogenesis	
ppa008330m	-2.40	0.000961951	TRICHOME BIREFRINGENCE-LIKE 19	Red ontogenesis	
ppa019670m	-2.40	0.002372946	Leucine-rich repeat protein kinase family protein	Red ontogenesis	
ppa017507m	-2.40	0.010698274		Red ontogenesis	
ppa001469m	-2.41	0.000484331	subtilisin-like serine protease 3	Red ontogenesis	
ppa008813m	-2.41	0.000533768	ethylene-forming enzyme	Red ontogenesis	
ppa015358m	-2.41	0.043232069	Ubiquitin-like superfamily protein	Red ontogenesis	
ppa013690m	-2.41	0.000932694	response to low sulfur 2	Red ontogenesis	
ppa023214m	-2.42	0.002072556		Red ontogenesis	
ppa026918m	-2.42	0.004130381	P-loop containing nucleoside triphosphate hydrolases superfamily	Red ontogenesis	
ppa019344m	-2.42	0.003247629	rotamase FKBP 1	Red ontogenesis	
ppa010371m	-2.42	0.001531171	RING/U-box superfamily protein	Red ontogenesis	
ppa010790m	-2.42	0.008024543		Red ontogenesis	
ppa004889m	-2.42	0.002963096	sphere organelles protein-related	Red ontogenesis	
ppa002521m	-2.43	0.002737556	Family of unknown function (DUF566)	Red ontogenesis	
ppa009388m	-2.43	0.001018919	Brassinosteroid signalling positive regulator (BZR1) family protein	Red ontogenesis	
ppa009987m	-2.43	0.000333945	light-harvesting chlorophyll B-binding protein 3	Red ontogenesis	Photosynthesis / Photosystem II
ppa020795m	-2.43	0.048050986	RING/U-box superfamily protein	Red ontogenesis	
ppa007562m	-2.43	0.001221352	myb domain protein 31	Red ontogenesis	
ppa022916m	-2.43	0.004226911	Glucose-methanol-choline (GMC) oxidoreductase family protein	Red ontogenesis	
ppa026277m	-2.43	0.036359938	FAR1-related sequence 5	Red ontogenesis	
ppa022205m	-2.44	0.008241792	myb domain protein 111	Red ontogenesis	
ppa001750m	-2.44	0.000305177	S-adenosyl-L-methionine-dependent methyltransferases superfar	Red ontogenesis	
ppa001341m	-2.44	0.01668424	AAA-type ATPase family protein	Red ontogenesis	
ppa007221m	-2.44	0.000598437	Haloacid dehalogenase-like hydrolase (HAD) superfamily protein	Red ontogenesis	
ppa005633m	-2.44	0.000180847	tubulin alpha-4 chain	Red ontogenesis	
ppa011649m	-2.44	0.023489632		Red ontogenesis	
ppa008517m	-2.44	0.000458403	N-acetyl-l-glutamate kinase	Red ontogenesis	
ppa026273m	-2.44	0.026119006	Abscisic acid-responsive (TB2/DP1, HVA22) family protein	Red ontogenesis	
ppa006304m	-2.46	0.010441587	Core-2/I-branching beta-1,6-N-acetylglucosaminyltransferase fam	Red ontogenesis	
ppa010657m	-2.46	0.016480117	Protein of unknown function (DUF1997)	Red ontogenesis	
ppa022089m	-2.46	0.000999291		Red ontogenesis	
ppa017074m	-2.47	0.001571188	dynamin-like protein	Red ontogenesis	
ppa001918m	-2.47	0.001217936	Subtilisin-like serine endopeptidase family protein	Red ontogenesis	
ppa001030m	-2.47	0.041115154	Protein of unknown function (DUF3741)	Red ontogenesis	
ppa011843m	-2.47	0.003728349	AUX/IAA transcriptional regulator family protein	Red ontogenesis	
ppa010430m	-2.47	0.00388034	Protein of unknown function (DUF179)	Red ontogenesis	
ppa019128m	-2.47	0.009825699	PLAC8 family protein	Red ontogenesis	
ppa005328m	-2.47	0.001122307	Granulin repeat cysteine protease family protein	Red ontogenesis	
ppa021606m	-2.47	0.035487918	Serine/threonine-protein kinase WNK (With No Lysine)-related	Red ontogenesis	
ppa022969m	-2.48	0.018014266	UDP-Glycosyltransferase superfamily protein	Red ontogenesis	
ppa024838m	-2.48	0.004985132	Oxidoreductase family protein	Red ontogenesis	
ppa025017m	-2.48	0.044641405	RING/U-box superfamily protein	Red ontogenesis	
ppa025396m	-2.48	0.004114836	glycosyl hydrolase family 81 protein	Red ontogenesis	
ppa020175m	-2.48	0.001269303		Red ontogenesis	
ppa004743m	-2.49	0.040020784	glycerol-3-phosphate acyltransferase 5	Red ontogenesis	
ppa019928m	-2.49	0.023838949		Red ontogenesis	
ppa012607m	-2.49	0.000753398	squamosa promoter binding protein-like 3	Red ontogenesis	
ppa026837m	-2.49	0.003882906	Protein of unknown function (DUF594)	Red ontogenesis	
ppa009494m	-2.50	0.00706358	Concanavalin A-like lectin family protein	Red ontogenesis	
ppa024411m	-2.50	0.000815993	Eukaryotic aspartyl protease family protein	Red ontogenesis	
ppa004719m	-2.51	0.010550187	cellulase 3	Red ontogenesis	
ppa022997m	-2.51	0.002634803	Leucine-rich repeat protein kinase family protein	Red ontogenesis	
ppa001340m	-2.51	0.000820265	beta-galactosidase 8	Red ontogenesis	
ppa003741m	-2.51	0.000358562	hydroxyproline-rich glycoprotein family protein	Red ontogenesis	
ppa012360m	-2.51	9.85E-05	Polyketide cyclase/dehydrase and lipid transport superfamily prot	Red ontogenesis	
ppa007480m	-2.52	0.000378909	Eukaryotic aspartyl protease family protein	Red ontogenesis	
ppa022356m	-2.52	7.86E-05	UDP-Glycosyltransferase superfamily protein	Red ontogenesis	
ppa016519m	-2.52	0.048904143	Regulator of chromosome condensation (RCC1) family with FYVE	Red ontogenesis	
ppa006213m	-2.52	0.035107447		Red ontogenesis	
ppa004814m	-2.52	8.45E-05	O-Glycosyl hydrolases family 17 protein	Red ontogenesis	Biotic Stress / Betaglucanase
ppa000904m	-2.53	0.002131148	Leucine-rich receptor-like protein kinase family protein	Red ontogenesis	
ppa022570m	-2.53	0.002955359	Cell cycle regulated microtubule associated protein	Red ontogenesis	
ppa020538m	-2.53	0.000909575	heat shock protein 70	Red ontogenesis	
ppa010490m	-2.53	0.004069472	PPPDE putative thiol peptidase family protein	Red ontogenesis	
ppa003436m	-2.53	0.001964493	basic helix-loop-helix (bHLH) DNA-binding superfamily protein	Red ontogenesis	
ppa021297m	-2.54	0.032053415	Ankyrin repeat family protein	Red ontogenesis	
ppa011791m	-2.54	0.003741472	GLNB1 homolog	Red ontogenesis	
ppa026104m	-2.54	0.003151393	Protein of unknown function, DUF547	Red ontogenesis	
ppa003519m	-2.54	0.000426716	homeobox gene 1	Red ontogenesis	
ppa000719m	-2.54	0.048960701	DNA/RNA polymerases superfamily protein	Red ontogenesis	
ppa021935m	-2.54	0.00111615	Auxin-responsive family protein	Red ontogenesis	
ppa005472m	-2.54	0.004407276	P-loop containing nucleoside triphosphate hydrolases superfamily	Red ontogenesis	
ppa012465m	-2.54	0.000989922	Lactoylglutathione lyase / glyoxalase I family protein	Red ontogenesis	
ppa020402m	-2.54	0.000436475	heat shock protein 70	Red ontogenesis	
ppa011114m	-2.55	0.000254172		Red ontogenesis	
ppa010365m	-2.55	6.55E-05	gamma tonoplast intrinsic protein	Red ontogenesis	
ppa022063m	-2.55	0.001031421	Disease resistance-responsive (dirigent-like protein) family protei	Red ontogenesis	Biotic Stress / PR-proteins
ppa022353m	-2.56	0.001652187		Red ontogenesis	
ppa012437m	-2.56	0.002755783		Red ontogenesis	
ppa026356m	-2.56	0.029873935	5\'-3\' exoribonuclease 3	Red ontogenesis	
ppa004799m	-2.56	2.42E-05	hydroperoxide lyase 1	Red ontogenesis	
ppa004732m	-2.56	0.002299488	O-Glycosyl hydrolases family 17 protein	Red ontogenesis	Biotic Stress / Betaglucanase
ppa004412m	-2.56	8.78E-05	DA1-related protein 2	Red ontogenesis	
ppa008834m	-2.56	0.048127218	Haloacid dehalogenase-like hydrolase (HAD) superfamily protein	Red ontogenesis	
ppa013280m	-2.56	0.021289474	GATA transcription factor 16	Red ontogenesis	
ppa010826m	-2.56	0.001037675	RAB GTPase homolog A3	Red ontogenesis	
ppa012874m	-2.56	0.00135218	Calcium-dependent lipid-binding (CaLB domain) family protein	Red ontogenesis	
ppa010243m	-2.57	0.003472194		Red ontogenesis	
ppa007625m	-2.57	0.001302242	Nucleotide-sugar transporter family protein	Red ontogenesis	
ppa004823m	-2.57	7.19E-05	sodium/calcium exchanger family protein / calcium-binding EF ha	Red ontogenesis	
ppa005866m	-2.58	0.003978893	TRICHOME BIREFRINGENCE-LIKE 25	Red ontogenesis	
ppa012876m	-2.58	0.048718268		Red ontogenesis	
ppa003289m	-2.58	0.001787705	S-adenosyl-L-methionine-dependent methyltransferases superfar	Red ontogenesis	
ppa025079m	-2.58	0.00741948		Red ontogenesis	
ppa002287m	-2.59	0.011395518	Leucine-rich repeat protein kinase family protein	Red ontogenesis	
ppa014823m	-2.59	0.000753127	purple acid phosphatase 16	Red ontogenesis	
ppa008881m	-2.59	5.99E-05	annexin 2	Red ontogenesis	
ppa000681m	-2.59	0.031549316	Leucine-rich repeat protein kinase family protein	Red ontogenesis	
ppa010283m	-2.59	0.039930364		Red ontogenesis	
ppa000793m	-2.59	0.009061633	P-loop containing nucleoside triphosphate hydrolases superfamily	Red ontogenesis	
ppa014583m	-2.60	0.042490029	GATA transcription factor 4	Red ontogenesis	
ppa003279m	-2.60	0.02029565	CVP2 like 1	Red ontogenesis	
ppa004144m	-2.60	0.004229316	Major facilitator superfamily protein	Red ontogenesis	
ppa009320m	-2.60	0.012419769	plasmodesmata-located protein 6	Red ontogenesis	
ppa013127m	-2.60	0.00016417	NADH-ubiquinone oxidoreductase-related	Red ontogenesis	
ppa004055m	-2.61	0.003441569	NHL domain-containing protein	Red ontogenesis	
ppa001345m	-2.61	3.04E-05	beta-galactosidase 10	Red ontogenesis	
ppa025195m	-2.61	0.000419911	fructose-bisphosphate aldolase 2	Red ontogenesis	
ppa012243m	-2.61	0.001199558	Protein of unknown function, DUF538	Red ontogenesis	
ppa008835m	-2.61	0.004612004	Protein of unknown function (DUF579)	Red ontogenesis	
ppa007959m	-2.62	4.58E-05	alpha/beta-Hydrolases superfamily protein	Red ontogenesis	
ppa004002m	-2.63	0.008427448	Pectin lyase-like superfamily protein	Red ontogenesis	
ppa003642m	-2.63	0.000304095	NSP-interacting kinase 3	Red ontogenesis	
ppa006769m	-2.63	0.00020832	myb domain protein 16	Red ontogenesis	

ppa006765m	-2.63	0.010875048	F-box family protein	Red ontogenesis	
ppa002215m	-2.64	0.004569712	mechanosensitive channel of small conductance-like 10	Red ontogenesis	
ppa025514m	-2.64	0.045580743	Subtilase family protein	Red ontogenesis	
ppa001398m	-2.64	0.000108948	1,2-alpha-L-fucosidases	Red ontogenesis	
ppa002595m	-2.64	0.000270875	heat shock protein 70B	Red ontogenesis	
ppa008472m	-2.64	0.047491399	Protein of unknown function (DUF677)	Red ontogenesis	
ppa008207m	-2.64	0.026581358	Exostosin family protein	Red ontogenesis	
ppa001089m	-2.64	0.000149365	glycosyl hydrolase family 10 protein / carbohydrate-binding domai	Red ontogenesis	
ppa007863m	-2.64	0.049320264	nodulin MtN21 /EamA-like transporter family protein	Red ontogenesis	
ppa026745m	-2.64	0.025052287	Peptidase M28 family protein	Red ontogenesis	
ppa022031m	-2.65	0.006263544	O-acyltransferase (WSD1-like) family protein	Red ontogenesis	
ppa005698m	-2.65	0.00018251	tubulin beta 8	Red ontogenesis	
ppa011639m	-2.65	3.74E-05	Glycoprotein membrane precursor GPI-anchored	Red ontogenesis	
ppa006597m	-2.65	0.044593712	Pyridoxal phosphate (PLP)-dependent transferases superfamily p	Red ontogenesis	Secondary Metabolism / Phenylpropanoids - Biotic Stress / Secondary metabolism involved into stress
ppa011589m	-2.65	0.041675728	basic leucine-zipper 58	Red ontogenesis	
ppa021465m	-2.65	0.014960922	NIMA (never in mitosis, gene A)-related 6	Red ontogenesis	
ppa024582m	-2.65	0.009376722		Red ontogenesis	
ppa002506m	-2.65	0.033646458	Phosphoribulokinase / Uridine kinase family	Red ontogenesis	
ppa008342m	-2.65	0.049121533	Exostosin family protein	Red ontogenesis	
ppa004080m	-2.66	0.011606676	IQ-domain 18	Red ontogenesis	
ppa008135m	-2.66	0.000441711	NAD(P)-linked oxidoreductase superfamily protein	Red ontogenesis	
ppa012204m	-2.66	0.009172422	early nodulin-like protein 15	Red ontogenesis	
ppa008080m	-2.66	0.024239137	BURP domain-containing protein	Red ontogenesis	
ppa003984m	-2.67	0.000175287	O-Glycosyl hydrolases family 17 protein	Red ontogenesis	Biotic Stress / Betaglucanase
ppa025759m	-2.67	0.005094492	Heavy metal transport/detoxification superfamily protein	Red ontogenesis	
ppa020782m	-2.67	0.000143468	transducin family protein / WD-40 repeat family protein	Red ontogenesis	
ppa020214m	-2.67	0.004421395		Red ontogenesis	
ppa001828m	-2.67	0.044140204	Subtilisin-like serine endopeptidase family protein	Red ontogenesis	
ppa009167m	-2.67	0.033536938	Plant protein of unknown function (DUF827)	Red ontogenesis	
ppa010900m	-2.67	0.049395887	oxidoreductases, acting on the aldehyde or oxo group of donors, I	Red ontogenesis	
ppa021003m	-2.68	0.043725949	tetratricopetide-repeat thioredoxin-like 3	Red ontogenesis	
ppa026998m	-2.68	0.001571188	DNA binding	Red ontogenesis	
ppa016415m	-2.68	0.00033606	Heavy metal transport/detoxification superfamily protein	Red ontogenesis	
ppa002022m	-2.69	0.000353248	Pleckstrin homology (PH) and lipid-binding START domains-conta	Red ontogenesis	
ppa025707m	-2.69	0.047138228	Leucine-rich repeat (LRR) family protein	Red ontogenesis	
ppa024549m	-2.69	0.039094637	cell wall / vacuolar inhibitor of fructosidase 2	Red ontogenesis	
ppa005360m	-2.69	0.000138863	serine carboxypeptidase-like 45	Red ontogenesis	
ppa002123m	-2.69	0.000568531	Leucine-rich repeat protein kinase family protein	Red ontogenesis	
ppa007313m	-2.69	0.001496569	Nucleotide-diphospho-sugar transferases superfamily protein	Red ontogenesis	
ppa1027230m	-2.69	0.032839317		Red ontogenesis	
ppa004282m	-2.69	1.42E-05	non-specific phospholipase C2	Red ontogenesis	
ppa013538m	-2.70	0.000172353		Red ontogenesis	
ppa025614m	-2.70	0.029681847	glutamine dumper 2	Red ontogenesis	
ppa004191m	-2.70	0.033479521	P-loop containing nucleoside triphosphate hydrolases superfamily	Red ontogenesis	
ppa003801m	-2.70	0.007840287		Red ontogenesis	
ppa006135m	-2.70	0.000119475	Phototropic-responsive NPH3 family protein	Red ontogenesis	
ppa016413m	-2.70	0.009699298		Red ontogenesis	
ppa016399m	-2.71	0.000944482	heat shock protein 70	Red ontogenesis	
ppa001070m	-2.71	0.020350597	armadillo repeat kinesin 3	Red ontogenesis	
ppa001294m	-2.71	0.041021381	hercules receptor kinase 2	Red ontogenesis	
ppa025624m	-2.71	0.003644409	glucuronidase 1	Red ontogenesis	
ppa002527m	-2.72	2.62E-05	S-adenosyl-L-methionine-dependent methyltransferases superfar	Red ontogenesis	
ppa004287m	-2.72	0.034226515	glucosidase 1	Red ontogenesis	
ppa017879m	-2.72	5.51E-05	TRICHOME BIREFRINGENCE-LIKE 6	Red ontogenesis	
ppa014426m	-2.72	0.009561327		Red ontogenesis	
ppa018399m	-2.73	0.000175203	senescence associated gene 20	Red ontogenesis	
ppa013937m	-2.73	0.000772371	cAMP-regulated phosphoprotein 19-related protein	Red ontogenesis	
ppa004971m	-2.73	0.000771627	aldehyde dehydrogenase 3F1	Red ontogenesis	
ppa003123m	-2.73	0.004989804		Red ontogenesis	
ppa017738m	-2.73	0.000384862	heat shock protein 70	Red ontogenesis	
ppa007235m	-2.73	3.72E-05	jasmonate-zim-domain protein 3	Red ontogenesis	
ppa002373m	-2.73	0.002299891	Leucine-rich repeat protein kinase family protein	Red ontogenesis	
ppa026189m	-2.73	0.000459562	tRNA/rRNA methyltransferase (SpoU) family protein	Red ontogenesis	
ppa020149m	-2.73	0.006209105	alpha dioxygenase	Red ontogenesis	
ppa020960m	-2.74	0.004656287	Cyclin family protein	Red ontogenesis	
ppa004838m	-2.74	0.003424325	Exostosin family protein	Red ontogenesis	
ppa006341m	-2.74	4.84E-05	O-fucosyltransferase family protein	Red ontogenesis	
ppa025262m	-2.74	0.038585644	plastid transcriptionally active 17	Red ontogenesis	
ppa026088m	-2.74	0.02931516	alpha/beta-Hydrolases superfamily protein	Red ontogenesis	
ppa005706m	-2.74	2.00E-05	GTP binding Elongation factor Tu family protein	Red ontogenesis	
ppa004027m	-2.74	0.000436202	calcium-dependent protein kinase 29	Red ontogenesis	
ppa022322m	-2.74	0.002643248	RNA-dependent RNA polymerase 1	Red ontogenesis	
ppa018010m	-2.74	0.039038883	ovate family protein 13	Red ontogenesis	
ppa006630m	-2.74	0.002352664	Leucine-rich repeat (LRR) family protein	Red ontogenesis	
ppa024626m	-2.75	0.004941345	Disease resistance protein (TIR-NBS-LRR class) family	Red ontogenesis	Biotic Stress / PR-proteins
ppa013553m	-2.75	0.001053314		Red ontogenesis	
ppa016421m	-2.75	0.043300757	Ankyrin repeat family protein	Red ontogenesis	
ppa026228m	-2.75	0.000252921	glycosyl hydrolase 9A1	Red ontogenesis	
ppa003618m	-2.75	0.006761542	Plant protein of unknown function (DUF946)	Red ontogenesis	
ppa002841m	-2.76	0.026762304	Ankyrin repeat family protein	Red ontogenesis	
ppa005957m	-2.76	0.000129488	Transmembrane amino acid transporter family protein	Red ontogenesis	
ppa007574m	-2.76	0.003350194	GDSL-like Lipase/Acylhydrolase superfamily protein	Red ontogenesis	
ppa010795m	-2.76	0.006977895		Red ontogenesis	
ppa018588m	-2.76	0.047796208	Zinc finger (C3HC4-type RING finger) family protein	Red ontogenesis	
ppa010871m	-2.76	0.026712753	indole-3-acetic acid inducible 11	Red ontogenesis	
ppa024737m	-2.77	0.000312737	Sulfite exporter TauE/SafE family protein	Red ontogenesis	
ppa021525m	-2.77	0.033760829	Plant protein of unknown function (DUF936)	Red ontogenesis	
ppa026471m	-2.77	0.03114667	zinc finger (C3HC4-type RING finger) family protein	Red ontogenesis	
ppa018085m	-2.77	0.034698306		Red ontogenesis	
ppa020579m	-2.78	0.002128265	Glucose-methanol-choline (GMC) oxidoreductase family protein	Red ontogenesis	
ppa018036m	-2.78	0.000158832	Protein of unknown function (DUF594)	Red ontogenesis	
ppa006147m	-2.78	0.04599124	lysm domain GPI-anchored protein 1 precursor	Red ontogenesis	
ppa002901m	-2.79	4.90E-05	DHHC-type zinc finger family protein	Red ontogenesis	
ppa000855m	-2.79	0.000430129	casein lytic proteinase B3	Red ontogenesis	
ppa009338m	-2.79	0.002564105	HMG (high mobility group) box protein with ARID/BRIGHT DNA-b	Red ontogenesis	
ppa012570m	-2.79	1.53E-05		Red ontogenesis	
ppa005681m	-2.79	0.038367541	Galactosyl transferase GMA12/MNN10 family protein	Red ontogenesis	
ppa016858m	-2.79	0.031383077	zinc finger (C2H2 type) family protein	Red ontogenesis	
ppa013279m	-2.80	0.022110956	plantacyanin	Red ontogenesis	
ppa003798m	-2.80	0.000245443	FAD-binding Berberine family protein	Red ontogenesis	
ppa021457m	-2.80	0.003853278	GDSL-like Lipase/Acylhydrolase superfamily protein	Red ontogenesis	
ppa026434m	-2.80	0.043500313	fatty acid desaturase 5	Red ontogenesis	
ppa024453m	-2.80	0.000717764	UDP-Glycosyltransferase superfamily protein	Red ontogenesis	
ppa001838m	-2.80	0.029336449	Serine/threonine-protein kinase WNK (With No Lysine)-related	Red ontogenesis	
ppb022980m	-2.80	0.001268436	root hair specific 16	Red ontogenesis	
ppa011935m	-2.81	0.001275806	indole-3-acetic acid inducible 19	Red ontogenesis	
ppa006620m	-2.81	0.015509348	BURP domain-containing protein	Red ontogenesis	
ppa022401m	-2.81	1.52E-05	4-coumarate:CoA ligase 3	Red ontogenesis	Secondary Metabolism / Phenylpropanoids - Biotic Stress / Secondary metabolism involved into stress
ppa010877m	-2.81	0.000520915	Phospholipase A2 family protein	Red ontogenesis	
ppa003680m	-2.81	0.006957149	rho guanyl-nucleotide exchange factor 1	Red ontogenesis	
ppa008654m	-2.81	0.016637768	Ankyrin repeat family protein	Red ontogenesis	
ppa022440m	-2.81	0.018017347	Laccase/Diphenol oxidase family protein	Red ontogenesis	Biotic Stress / Secondary metabolism involved into stress
ppa006363m	-2.81	0.008823425	tubby like protein 6	Red ontogenesis	
ppa1027144m	-2.81	0.031383077	Glutaredoxin family protein	Red ontogenesis	
ppa005515m	-2.82	0.000833877	UDP-Glycosyltransferase superfamily protein	Red ontogenesis	
ppa020461m	-2.82	0.015418286	heat shock protein 70	Red ontogenesis	
ppa008640m	-2.82	0.015721947	chorismate mutase 3	Red ontogenesis	
ppa021011m	-2.82	0.002301578	Serine carboxypeptidase S28 family protein	Red ontogenesis	
ppa001042m	-2.82	0.006725496	Leucine-rich repeat transmembrane protein kinase family protein	Red ontogenesis	
ppa019436m	-2.83	0.017605947	eukaryotic initiation factor 4A-III	Red ontogenesis	
ppa004680m	-2.83	0.000160463	Basic helix-loop-helix (bHLH) DNA-binding family protein	Red ontogenesis	
ppa013213m	-2.84	0.000470854		Red ontogenesis	
ppa025664m	-2.84	0.02462616	peptidases	Red ontogenesis	
ppa009898m	-2.84	0.033464877	Homeodomain-like superfamily protein	Red ontogenesis	
ppa001675m	-2.84	0.00488075	Glycosyl hydrolase family protein	Red ontogenesis	
ppa015092m	-2.84	0.001275298	Ankyrin repeat family protein	Red ontogenesis	
ppa003769m	-2.85	0.007578601	rho guanyl-nucleotide exchange factor 1	Red ontogenesis	
ppa014689m	-2.85	0.035642722		Red ontogenesis	
ppa005721m	-2.85	0.000185557	FASCICLIN-like arabinogalactan protein 17 precursor	Red ontogenesis	
ppa006040m	-2.86	0.031034309	DHHC-type zinc finger family protein	Red ontogenesis	
ppa000491m	-2.86	0.001642732	phytochrome E	Red ontogenesis	
ppa011379m	-2.86	0.000336549	Protein of unknown function (DUF567)	Red ontogenesis	
ppb024523m	-2.86	0.022370764	Seven transmembrane MLO family protein	Red ontogenesis	
ppa016532m	-2.87	0.03604324	myb domain protein 82	Red ontogenesis	
ppa012574m	-2.87	4.39E-05	Protein of unknown function, DUF538	Red ontogenesis	
ppa019396m	-2.87	0.045876636	D-isomer specific 2-hydroxyacid dehydrogenase family protein	Red ontogenesis	
ppa005157m	-2.88	0.029428283	alpha/beta-Hydrolases superfamily protein	Red ontogenesis	
ppa001020m	-2.88	0.002131593	mechanosensitive channel of small conductance-like 6	Red ontogenesis	
ppa011914m	-2.88	0.012727213	PsbQ-like 3	Red ontogenesis	Photosynthesis / Photosystem II
ppa011894m	-2.88	0.026754332		Red ontogenesis	
ppa001793m	-2.88	0.000156024	Protein of unknown function (DUF288)	Red ontogenesis	
ppa010961m	-2.89	0.00012827	APS kinase	Red ontogenesis	
ppa003537m	-2.89	0.024425919	Protein of unknown function (DUF668)	Red ontogenesis	
ppa007753m	-2.90	0.025458563	myb-like HTH transcriptional regulator family protein	Red ontogenesis	
ppa018774m	-2.90	0.020092439	Leucine-rich repeat transmembrane protein kinase	Red ontogenesis	
ppa004343m	-2.90	0.004479645	cytochrome P450, family 71, subfamily B, polypeptide 36	Red ontogenesis	
ppa011460m	-2.90	0.024151242	germin 3	Red ontogenesis	
ppa013552m	-2.90	0.000763229	Heavy metal transport/detoxification superfamily protein	Red ontogenesis	

ppa021416m	-2.90	0.01900871	embryo defective 1703	Red ontogenesis	
ppa022564m	-2.91	0.047883234		Red ontogenesis	
ppa025772m	-2.91	0.017020513	SNF2 domain-containing protein / helicase domain-containing pro	Red ontogenesis	
ppa015759m	-2.91	0.020163288	Leucine-rich repeat (LRR) family protein	Red ontogenesis	
ppa021746m	-2.91	0.029029617		Red ontogenesis	
ppa020233m	-2.92	0.038140533	NAC domain containing protein 28	Red ontogenesis	
ppa000357m	-2.92	0.000355835	ketose-bisphosphate aldolase class-II family protein	Red ontogenesis	
ppa007817m	-2.92	0.032946884	alpha/beta-Hydrolases superfamily protein	Red ontogenesis	
ppa025791m	-2.92	0.029963465	Plant protein of unknown function (DUF247)	Red ontogenesis	
ppa022028m	-2.92	0.023848051	Ankyrin repeat family protein	Red ontogenesis	
ppa017434m	-2.93	0.00097974	Ankyrin repeat family protein	Red ontogenesis	
ppa012218m	-2.93	0.008134612	Bifunctional inhibitor/lipid-transfer protein/seed storage 2S albumi	Red ontogenesis	
ppa007323m	-2.93	0.001562905	S-adenosyl-L-methionine-dependent methyltransferases superfar	Red ontogenesis	
ppa005914m	-2.93	3.00E-05	microtubule-associated proteins 65-1	Red ontogenesis	
ppa015739m	-2.93	0.000854422	Protein kinase superfamily protein	Red ontogenesis	
ppa020243m	-2.93	0.000175203	2-oxoglutarate (2OG) and Fe(II)-dependent oxygenase superfami	Red ontogenesis	
ppa008726m	-2.93	0.000660623	NAD(P)-binding Rossmann-fold superfamily protein	Red ontogenesis	
ppa000034m	-2.94	0.000141584	acetyl-CoA carboxylase 1	Red ontogenesis	
ppa005790m	-2.94	0.026958308	Transmembrane amino acid transporter family protein	Red ontogenesis	
ppa012259m	-2.94	0.00151424		Red ontogenesis	
ppa021929m	-2.94	0.02620498	chromatin remodeling 4	Red ontogenesis	
ppa004534m	-2.94	0.032946884	O-Glycosyl hydrolases family 17 protein	Red ontogenesis	Biotic Stress / Betaglucanase
ppa022557m	-2.95	4.67E-05	receptor lectin kinase	Red ontogenesis	
ppa017360m	-2.95	0.022025338	Glucose-methanol-choline (GMC) oxidoreductase family protein	Red ontogenesis	
ppa008223m	-2.95	0.000228521	Auxin efflux carrier family protein	Red ontogenesis	
ppa012366m	-2.95	0.002580314	early nodulin-like protein 17	Red ontogenesis	
ppa011175m	-2.95	2.72E-05	Protein-tyrosine phosphatase-like, PTPLA	Red ontogenesis	
ppa008288m	-2.95	3.80E-05	arginase	Red ontogenesis	
ppa016034m	-2.96	0.004139475		Red ontogenesis	
ppa010180m	-2.96	0.005913657	expansin A4	Red ontogenesis	
ppa008797m	-2.96	6.75E-06	NAD(P)-binding Rossmann-fold superfamily protein	Red ontogenesis	Secondary Metabolism / Dihydroflavonols -Biotic Stress / Secondary metabolism involved into stress
ppa022417m	-2.96	0.000536921	Duplicated homeodomain-like superfamily protein	Red ontogenesis	
ppb024045m	-2.96	0.002485899	cyclic nucleotide gated channel 1	Red ontogenesis	
ppa023215m	-2.96	0.00195114	asparaginyl-tRNA synthetase 2	Red ontogenesis	
ppa019791m	-2.96	0.002372946	Serine protease inhibitor (SERPIN) family protein	Red ontogenesis	
ppa012459m	-2.97	0.000833877	brassinosteroid-responsive RING-H2	Red ontogenesis	
ppa018501m	-2.97	0.020880952	Acyl-CoA N-acyltransferase with RING/FYVE/PHD-type zinc finge	Red ontogenesis	
ppa003804m	-2.97	0.031409956	glucuronidase 2	Red ontogenesis	
ppa002939m	-2.97	0.001670754	glycosyl hydrolase 9C2	Red ontogenesis	
ppa014957m	-2.97	0.000759102		Red ontogenesis	
ppa000123m	-2.97	0.026618906	DNA binding;ATP binding	Red ontogenesis	
ppa004076m	-2.97	0.000573852	FAD-binding Berberine family protein	Red ontogenesis	
ppa003900m	-2.97	0.000202573	squalene epoxidase 2	Red ontogenesis	
ppa018436m	-2.97	0.040744721	RNAse THREE-like protein 1	Red ontogenesis	
ppa010643m	-2.98	0.000478115	Protein of unknown function (DUF674)	Red ontogenesis	
ppa009245m	-2.98	0.000519765	xyloglucan endotransglucosylase/hydrolase 8	Red ontogenesis	
ppa002138m	-2.98	0.000343471	kinesin 3	Red ontogenesis	
ppa014553m	-2.98	0.000861751	Eukaryotic aspartyl protease family protein	Red ontogenesis	
ppa023293m	-2.98	0.011730293	GroES-like zinc-binding dehydrogenase family protein	Red ontogenesis	
ppa018373m	-2.98	0.006708467	Plant protein of unknown function (DUF247)	Red ontogenesis	
ppa025070m	-2.99	0.031228258	formin 8	Red ontogenesis	
ppa000127m	-2.99	0.000718925	Outer arm dynein light chain 1 protein	Red ontogenesis	
ppa013330m	-2.99	0.00020087		Red ontogenesis	
ppa020132m	-3.00	0.000536921		Red ontogenesis	
ppa010340m	-3.00	0.001290143	Integrase-type DNA-binding superfamily protein	Red ontogenesis	
ppa017173m	-3.00	0.004731356		Red ontogenesis	
ppa002366m	-3.00	0.022176277	Leucine-rich repeat protein kinase family protein	Red ontogenesis	
ppa004734m	-3.01	0.000291001	RING/U-box superfamily protein	Red ontogenesis	
ppa012671m	-3.01	0.033126333	AWPM-19-like family protein	Red ontogenesis	
ppa004549m	-3.01	0.000179284	Protein kinase superfamily protein	Red ontogenesis	
ppa020555m	-3.02	2.05E-05	Uncharacterised protein family SERF	Red ontogenesis	
ppa017485m	-3.02	0.001749622	ubiquitin-specific protease 12	Red ontogenesis	
ppa022644m	-3.03	0.022953805	Protein of unknown function (DUF1677)	Red ontogenesis	
ppa014215m	-3.04	0.000682471		Red ontogenesis	
ppa003722m	-3.04	0.017372773	purple acid phosphatase 23	Red ontogenesis	
ppa019416m	-3.04	0.020160539	acyl-CoA sterol acyl transferase 1	Red ontogenesis	Biotic Stress / Secondary metabolism involved into stress
ppa000222m	-3.04	0.038370813		Red ontogenesis	
ppa006446m	-3.04	0.00025618	Pectinacetylesterase family protein	Red ontogenesis	
ppa009841m	-3.04	0.025289077	Pathogenesis-related thaumatin superfamily protein	Red ontogenesis	
ppa000583m	-3.05	0.000484331	Kinesin motor family protein	Red ontogenesis	
ppa005769m	-3.05	0.013314081	TRICHOME BIREFRINGENCE-LIKE 27	Red ontogenesis	
ppa011654m	-3.05	0.047207157		Red ontogenesis	
ppa011587m	-3.05	0.000112342		Red ontogenesis	
ppa024148m	-3.06	0.005379942	FAD-binding Berberine family protein	Red ontogenesis	
ppa012098m	-3.06	0.017584255	D-Tyr-tRNA(Tyr) deacylase family protein	Red ontogenesis	
ppa019210m	-3.07	0.015331835	alpha/beta-Hydrolases superfamily protein	Red ontogenesis	
ppa009995m	-3.07	0.00057753		Red ontogenesis	
ppa010379m	-3.07	0.017970839	expansin A1	Red ontogenesis	
ppa000847m	-3.07	0.000345364	Leucine-rich receptor-like protein kinase family protein	Red ontogenesis	
ppa009792m	-3.07	0.005640154	xyloglucan endotransglucosylase/hydrolase 15	Red ontogenesis	
ppa002579m	-3.07	0.00028017	Leucine-rich repeat protein kinase family protein	Red ontogenesis	
ppa007558m	-3.08	0.021976869	beta-1,4-N-acetylglucosaminyltransferase family protein	Red ontogenesis	
ppa017084m	-3.08	0.009228908	Peroxidase superfamily protein	Red ontogenesis	
ppa009253m	-3.08	0.000683459	ARM repeat superfamily protein	Red ontogenesis	
ppa008510m	-3.08	7.83E-06	plasma membrane intrinsic protein 1B	Red ontogenesis	
ppa002580m	-3.09	0.000161346	Reticulon family protein	Red ontogenesis	
ppa000320m	-3.09	0.021393523	Actin-binding FH2 (Formin Homology) protein	Red ontogenesis	
ppb019242m	-3.09	0.016755865	Polynucleotidyl transferase, ribonuclease H-like superfamily prote	Red ontogenesis	
ppa005761m	-3.09	0.028639555	Pectin lyase-like superfamily protein	Red ontogenesis	
ppa007771m	-3.09	0.027120755	glutamine synthetase 1;4	Red ontogenesis	
ppa006267m	-3.09	0.002755783	Protein of unknown function (DUF1005)	Red ontogenesis	
ppa010321m	-3.10	0.000480364	FASCICLIN-like arabinoogalactan 7	Red ontogenesis	
ppa014884m	-3.10	0.047456674	myb domain protein 66	Red ontogenesis	
ppa017132m	-3.10	0.021713421	HXXXD-type acyl-transferase family protein	Red ontogenesis	Secondary Metabolism / Phenylpropanoids - Biotic Stress / Secondary metabolism involved into stress
ppa016986m	-3.11	0.013764959	Cysteine/Histidine-rich C1 domain family protein	Red ontogenesis	
ppa004023m	-3.12	0.003763928	ARM repeat superfamily protein	Red ontogenesis	
ppa006351m	-3.12	3.78E-05	PLAC8 family protein	Red ontogenesis	
ppa001667m	-3.12	0.000943994	GDSL-like Lipase/Acylhydrolase superfamily protein	Red ontogenesis	
ppa009951m	-3.12	0.00259607	methyl esterase 1	Red ontogenesis	
ppa001720m	-3.12	0.016974468		Red ontogenesis	
ppa025830m	-3.13	0.039809449	Ovate family protein	Red ontogenesis	
ppa012222m	-3.13	7.19E-05	HVA22 homologue C	Red ontogenesis	
ppa013081m	-3.13	0.004416044	plant natriuretic peptide A	Red ontogenesis	
ppa015930m	-3.13	0.00010301		Red ontogenesis	
ppa016241m	-3.13	0.021308775	ortholog of maize chloroplast splicing factor CRS1	Red ontogenesis	
ppa002548m	-3.14	0.000644269	Leucine-rich repeat protein kinase family protein	Red ontogenesis	
ppa005992m	-3.14	0.004222071	Glycosyl hydrolase superfamily protein	Red ontogenesis	
ppa013785m	-3.14	0.026149478		Red ontogenesis	
ppa011288m	-3.14	0.0002111	PHD finger family protein / bromo-adjacent homology (BAH) doma	Red ontogenesis	
ppa003027m	-3.14	0.021849621	Plant protein of unknown function (DUF828)	Red ontogenesis	
ppa008725m	-3.15	0.000639823	Protein of unknown function (DUF579)	Red ontogenesis	
ppa009058m	-3.15	0.000202263	syntaxin of plants 131	Red ontogenesis	
ppa019618m	-3.15	0.010559894	SET-domain containing protein lysine methyltransferase family pr	Red ontogenesis	
ppa003371m	-3.15	0.015380167	Leucine-rich repeat protein kinase family protein	Red ontogenesis	
ppa003047m	-3.16	5.72E-05	Plant invertase/pectin methylesterase inhibitor superfamily	Red ontogenesis	
ppa021568m	-3.16	0.004888746	myb domain protein 85	Red ontogenesis	
ppa017871m	-3.16	0.016065842	PEP1 receptor 1	Red ontogenesis	
ppa009714m	-3.16	0.024986977	alpha carbonic anhydrase 4	Red ontogenesis	
ppa021630m	-3.17	0.022370764	Protein kinase superfamily protein	Red ontogenesis	
ppa027182m	-3.17	0.018159502	Basic helix-loop-helix (bHLH) DNA-binding family protein	Red ontogenesis	
ppb013860m	-3.17	0.002132248		Red ontogenesis	
ppa004152m	-3.17	0.003781072	cytochrome P450, family 71, subfamily A, polypeptide 20	Red ontogenesis	
ppa002257m	-3.17	0.021172211	Protein kinase protein with adenine nucleotide alpha hydrolases-li	Red ontogenesis	
ppb008505m	-3.18	0.028290678	Pectin lyase-like superfamily protein	Red ontogenesis	
ppa017984m	-3.18	0.017800596	Frigida-like protein	Red ontogenesis	
ppa007566m	-3.18	0.041474102	Cysteine proteinases superfamily protein	Red ontogenesis	
ppa013286m	-3.18	0.000696378	Bifunctional inhibitor/lipid-transfer protein/seed storage 2S albumi	Red ontogenesis	
ppa001770m	-3.18	0.000326961	Subtilisin-like serine endopeptidase family protein	Red ontogenesis	
ppa017224m	-3.19	0.027809195	homologue of bacterial MinE 1	Red ontogenesis	
ppa009533m	-3.19	0.008237542	Integrase-type DNA-binding superfamily protein	Red ontogenesis	
ppa009419m	-3.19	8.76E-05	homeobox protein 6	Red ontogenesis	
ppa010252m	-3.19	0.002602532	myb domain protein 4	Red ontogenesis	
ppa025987m	-3.19	0.01877312	alpha/beta-Hydrolases superfamily protein	Red ontogenesis	
ppa005482m	-3.20	0.012334185	isopentenyltransferase 9	Red ontogenesis	
ppa017289m	-3.20	0.001439603	terpene synthase 21	Red ontogenesis	Biotic Stress / Secondary metabolism involved into stress
ppa003867m	-3.21	0.013398025	phosphoinositide 4-kinase gamma 4	Red ontogenesis	
ppa015244m	-3.21	0.021102932	Myosin heavy chain-related protein	Red ontogenesis	
ppa020393m	-3.21	0.040840825	Glycosyl hydrolase superfamily protein	Red ontogenesis	Biotic Stress / Betaglucanase
ppa1027164m	-3.21	0.044546552	alpha/beta-Hydrolases superfamily protein	Red ontogenesis	
ppa013748m	-3.21	0.000389093		Red ontogenesis	
ppa022777m	-3.21	0.000634899	Cytochrome P450 superfamily protein	Red ontogenesis	
ppa003595m	-3.21	0.007506816	Glucose-methanol-choline (GMC) oxidoreductase family protein	Red ontogenesis	
ppa024008m	-3.21	0.003800524	Survival protein SurE-like phosphatase/nucleotidase	Red ontogenesis	
ppa000340m	-3.22	0.002315621	P-glycoprotein 2	Red ontogenesis	
ppa022150m	-3.22	0.018919918	F-box and associated interaction domains-containing protein	Red ontogenesis	
ppb008932m	-3.22	0.045990939		Red ontogenesis	
ppa024566m	-3.23	0.018670672	heat shock cognate protein 70-1	Red ontogenesis	
ppa003860m	-3.23	0.000175301	SKU5 similar 5	Red ontogenesis	

ppa022210m	-3.24	0.018394155	*IQ-domain 19*	Red ontogenesis	
ppa000646m	-3.24	0.018366417	*Double Clp-N motif-containing P-loop nucleoside triphosphate hyd*	Red ontogenesis	
ppa005434m	-3.24	0.000394691	*UDP-D-glucuronate 4-epimerase 6*	Red ontogenesis	
ppa023124m	-3.24	0.017584255	*CCT motif family protein*	Red ontogenesis	
ppa006718m	-3.24	5.58E-05	*Pectinacetylesterase family protein*	Red ontogenesis	
ppa012284m	-3.24	0.010639771	*LOB domain-containing protein 21*	Red ontogenesis	
ppa018641m	-3.25	0.013411184	*Pectinacetylesterase family protein*	Red ontogenesis	
ppa020668m	-3.25	0.019864328	*F-box family protein*	Red ontogenesis	
ppa024188m	-3.25	2.09E-05		Red ontogenesis	
ppa003709m	-3.26	0.009591097	*Plant protein of unknown function (DUF827)*	Red ontogenesis	
ppa003152m	-3.26	0.005718106		Red ontogenesis	
ppa017814m	-3.26	0.031906781	*Disease resistance protein (TIR-NBS-LRR class) family*	Red ontogenesis	Biotic Stress / PR-proteins
ppa003634m	-3.26	0.000313712	*Protein of unknown function, DUF547*	Red ontogenesis	
ppa001116m	-3.26	0.000807629	*formin homology 1*	Red ontogenesis	
ppa010342m	-3.27	0.001360682	*indole-3-acetic acid 7*	Red ontogenesis	
ppa022171m	-3.27	0.00391626	*heat shock protein 70*	Red ontogenesis	
ppa018789m	-3.27	0.001504949	*Leucine-rich receptor-like protein kinase family protein*	Red ontogenesis	
ppa002487m	-3.27	0.026150784	*Galactosyltransferase family protein*	Red ontogenesis	
ppb013628m	-3.27	0.01928644	*cyclic nucleotide gated channel 1*	Red ontogenesis	
ppa022313m	-3.28	0.016644912	*FASCICLIN-like arabinogalactan 6*	Red ontogenesis	
ppa012666m	-3.28	0.023213115	*Thioesterase superfamily protein*	Red ontogenesis	
ppa003256m	-3.28	0.000136901	*IQ-domain 31*	Red ontogenesis	
ppa006492m	-3.28	0.007767546	*Ubiquitin carboxyl-terminal hydrolase family protein*	Red ontogenesis	
ppa007755m	-3.28	8.09E-05	*decapping 1*	Red ontogenesis	
ppb024787m	-3.29	0.034245358		Red ontogenesis	
ppa011572m	-3.29	0.045412759	*cyclin p4;1*	Red ontogenesis	
ppa000661m	-3.29	4.46E-05		Red ontogenesis	
ppa003224m	-3.30	1.70E-06	*Cupredoxin superfamily protein*	Red ontogenesis	
ppa001660m	-3.30	0.02756566	*DENN (AEX-3) domain-containing protein*	Red ontogenesis	
ppa026648m	-3.30	0.035455043	*BED zinc finger ;hAT family dimerisation domain*	Red ontogenesis	
ppa022026m	-3.31	0.013931779	*Double Clp-N motif-containing P-loop nucleoside triphosphate hyd*	Red ontogenesis	
ppa009969m	-3.32	0.014567398	*Tetraspanin family protein*	Red ontogenesis	
ppa000606m	-3.32	0.000386504	*aspartate kinase-homoserine dehydrogenase ii*	Red ontogenesis	
ppa001331m	-3.32	0.042275157	*Protein kinase superfamily protein*	Red ontogenesis	
ppa003987m	-3.33	6.09E-05	*Pre-mRNA splicing Prp18-interacting factor*	Red ontogenesis	
ppa009912m	-3.33	0.000777908	*TPX2 (targeting protein for Xklp2) protein family*	Red ontogenesis	
ppa019278m	-3.34	0.001497555	*wall-associated kinase 2*	Red ontogenesis	
ppa000107m	-3.34	3.74E-05	*Kinase interacting (KIP1-like) family protein*	Red ontogenesis	
ppa019440m	-3.35	0.034124225		Red ontogenesis	
ppa022897m	-3.35	0.00044163		Red ontogenesis	
ppa005626m	-3.35	0.025197531	*tubulin alpha-2 chain*	Red ontogenesis	
ppa003735m	-3.36	0.018119803	*transmembrane kinase-like 1*	Red ontogenesis	
ppa024506m	-3.36	0.000999503	*Protein of unknown function (DUF579)*	Red ontogenesis	
ppa011549m	-3.36	0.03788928	*myb domain protein 42*	Red ontogenesis	
ppa022548m	-3.36	0.009794946	*BR-signaling kinase 2*	Red ontogenesis	
ppb004684m	-3.36	4.75E-05	*Bifunctional inhibitor/lipid-transfer protein/seed storage 2S albumi*	Red ontogenesis	
ppa001252m	-3.36	0.014569671	*Double Clp-N motif-containing P-loop nucleoside triphosphate hyd*	Red ontogenesis	
ppa006611m	-3.37	0.000108352	*Squamosa promoter-binding protein-like (SBP domain) transcripti*	Red ontogenesis	
ppa018700m	-3.37	0.000253071	*Plant basic secretory protein (BSP) family protein*	Red ontogenesis	
ppa019678m	-3.37	0.037298667	*Ankyrin repeat family protein*	Red ontogenesis	
ppa004571m	-3.37	0.000106817	*glycerol-3-phosphate acyltransferase 8*	Red ontogenesis	
ppa002755m	-3.38	0.009384042	*Exostosin family protein*	Red ontogenesis	
ppa005564m	-3.38	3.92E-05	*CVP2 like 1*	Red ontogenesis	
ppa018952m	-3.38	0.01040866		Red ontogenesis	
ppa011205m	-3.39	0.000683154	*serine carboxypeptidase-like 40*	Red ontogenesis	
ppa013764m	-3.39	0.018808759	*SAUR-like auxin-responsive protein family*	Red ontogenesis	
ppa009645m	-3.40	0.000106136	*general regulatory factor 11*	Red ontogenesis	
ppa001732m	-3.41	6.31E-05	*Subtilisin-like serine endopeptidase family protein*	Red ontogenesis	
ppa011353m	-3.41	0.038820912	*Arabidopsis RAC-like 9*	Red ontogenesis	
ppa006702m	-3.42	0.016485509	*RHOMBOID-like 1*	Red ontogenesis	
ppa003089m	-3.42	0.012423556	*Leucine-rich repeat protein kinase family protein*	Red ontogenesis	
ppa024696m	-3.42	0.020163288	*TRAF-like family protein*	Red ontogenesis	
ppa012078m	-3.42	0.029408289		Red ontogenesis	
ppa001023m	-3.43	0.001708018		Red ontogenesis	
ppa026172m	-3.43	0.024434715	*SAUR-like auxin-responsive protein family*	Red ontogenesis	
ppa013981m	-3.43	0.021301354	*SAUR-like auxin-responsive protein family*	Red ontogenesis	
ppa026548m	-3.44	0.011470488		Red ontogenesis	
ppa012336m	-3.44	0.001092665	*hydroxyproline-rich glycoprotein family protein*	Red ontogenesis	
ppa017321m	-3.44	0.00328399	*serine carboxypeptidase-like 25*	Red ontogenesis	
ppa024718m	-3.45	0.008277044	*negative regulator of systemic acquired resistance (SNI1)*	Red ontogenesis	
ppa015371m	-3.45	0.007569572	*Protein of unknown function (DUF594)*	Red ontogenesis	
ppa026898m	-3.45	0.02909084	*Plant protein of unknown function (DUF868)*	Red ontogenesis	
ppb004066m	-3.45	0.012429823	*O-Glycosyl hydrolases family 17 protein*	Red ontogenesis	Biotic Stress / Betaglucanase
ppa010654m	-3.45	0.014137762	*expansin A15*	Red ontogenesis	
ppa011989m	-3.45	3.72E-05	*Lipase/lipooxygenase, PLAT/LH2 family protein*	Red ontogenesis	
ppa019781m	-3.45	0.0012313	*TRAM, LAG1 and CLN8 (TLC) lipid-sensing domain containing pr*	Red ontogenesis	
ppa026075m	-3.46	0.013233959	*Carbohydrate-binding X8 domain superfamily protein*	Red ontogenesis	Biotic Stress / Betaglucanase
ppa005621m	-3.47	0.012040137	*Protein kinase superfamily protein*	Red ontogenesis	
ppa011466m	-3.47	0.000185511	*HSP20-like chaperones superfamily protein*	Red ontogenesis	
ppa026800m	-3.48	0.037785189	*2-oxoglutarate (2OG) and Fe(II)-dependent oxygenase superfami*	Red ontogenesis	
ppa009823m	-3.48	0.014736535	*Protein kinase superfamily protein*	Red ontogenesis	
ppa019577m	-3.48	0.000177138	*Eukaryotic aspartyl protease family protein*	Red ontogenesis	
ppa008289m	-3.48	0.00043728	*carbonic anhydrase 1*	Red ontogenesis	
ppa001701m	-3.48	0.002170803	*Subtilase family protein*	Red ontogenesis	
ppa008820m	-3.48	0.011435197	*Peroxidase superfamily protein*	Red ontogenesis	
ppa004726m	-3.50	0.000129488	*Eukaryotic aspartyl protease family protein*	Red ontogenesis	
ppa003972m	-3.50	0.006863818	*EPS15 homology domain 2*	Red ontogenesis	
ppa004832m	-3.51	0.023815126	*Cytochrome P450 superfamily protein*	Red ontogenesis	
ppa011715m	-3.51	7.19E-05	*cyclin p1;1*	Red ontogenesis	
ppa020813m	-3.51	0.025113001	*Carbohydrate-binding X8 domain superfamily protein*	Red ontogenesis	Biotic Stress / Betaglucanase
ppa001662m	-3.51	0.000921682	*EXS (ERD1/XPR1/SYG1) family protein*	Red ontogenesis	
ppa009688m	-3.51	0.010985838	*plasmodesmata-located protein 7*	Red ontogenesis	
ppa016335m	-3.52	0.032472938	*glycerol-3-phosphate acyltransferase 6*	Red ontogenesis	
ppa024794m	-3.53	0.015319255	*Homeodomain-like superfamily protein*	Red ontogenesis	
ppa001174m	-3.53	0.014159302	*Leucine-rich repeat protein kinase family protein*	Red ontogenesis	
ppa026620m	-3.53	0.021258426	*Plant protein of unknown function (DUF247)*	Red ontogenesis	
ppa010695m	-3.53	0.010463581	*plasmodesmata callose-binding protein 3*	Red ontogenesis	Biotic Stress / Betaglucanase
ppa012687m	-3.53	0.030839789	*Ran BP2/NZF zinc finger-like superfamily protein*	Red ontogenesis	
ppa012203m	-3.54	0.022740645		Red ontogenesis	
ppa010387m	-3.54	0.026762304	*vacuolar iron transporter 1*	Red ontogenesis	
ppa001102m	-3.54	9.25E-05	*heat shock protein 101*	Red ontogenesis	
ppa016850m	-3.54	0.00068108	*BURP domain-containing protein*	Red ontogenesis	
ppa016161m	-3.55	0.027544115	*Plant protein of unknown function (DUF868)*	Red ontogenesis	
ppa009272m	-3.55	0.012334185	*Plant protein of unknown function (DUF868)*	Red ontogenesis	
ppa011610m	-3.56	7.79E-05	*Late embryogenesis abundant (LEA) hydroxyproline-rich glycopro*	Red ontogenesis	
ppa022321m	-3.56	0.006143153		Red ontogenesis	
ppa000511m	-3.57	2.00E-05	*BCL-2-associated athanogene 6*	Red ontogenesis	
ppa026129m	-3.58	0.004786751	*RNI-like superfamily protein*	Red ontogenesis	
ppa008391m	-3.58	0.000108001	*alpha/beta-Hydrolases superfamily protein*	Red ontogenesis	
ppa001878m	-3.58	0.015813689	*ABC-2 type transporter family protein*	Red ontogenesis	
ppa003399m	-3.58	0.000215562	*FKBP-type peptidyl-prolyl cis-trans isomerase family protein*	Red ontogenesis	
ppa006883m	-3.59	0.006816153	*Protein of unknown function (DUF620)*	Red ontogenesis	
ppa004915m	-3.59	2.73E-05	*Major facilitator superfamily protein*	Red ontogenesis	
ppa004142m	-3.59	0.00015327	*Protein of unknown function (DUF604)*	Red ontogenesis	
ppa019644m	-3.59	0.009306466	*2-oxoglutarate (2OG) and Fe(II)-dependent oxygenase superfami*	Red ontogenesis	
ppa003135m	-3.60	0.006496079	*NIMA-related kinase 2*	Red ontogenesis	
ppa011783m	-3.60	0.0002391	*Matrixin family protein*	Red ontogenesis	
ppa017178m	-3.60	0.010163728		Red ontogenesis	
ppa020936m	-3.60	0.004199937	*BURP domain-containing protein*	Red ontogenesis	
ppa007109m	-3.61	0.0191849	*Kelch repeat-containing F-box family protein*	Red ontogenesis	
ppa014979m	-3.61	0.018152873	*Root hair defective 3 GTP-binding protein (RHD3)*	Red ontogenesis	
ppa026134m	-3.61	0.003135964	*Leucine-rich receptor-like protein kinase family protein*	Red ontogenesis	
ppa006511m	-3.61	0.000144806	*NAD(P)-binding Rossmann-fold superfamily protein*	Red ontogenesis	
ppa002298m	-3.61	0.000203684	*white-brown complex homolog protein 11*	Red ontogenesis	
ppa015726m	-3.61	0.00195852		Red ontogenesis	
ppa008597m	-3.62	0.026476085	*metacaspase 9*	Red ontogenesis	
ppa008713m	-3.62	1.66E-05	*NAD(P)-binding Rossmann-fold superfamily protein*	Red ontogenesis	
ppa012200m	-3.62	0.014655493	*ubiquitin-conjugating enzyme19*	Red ontogenesis	
ppa015189m	-3.63	0.015154657	*SAUR-like auxin-responsive protein family*	Red ontogenesis	
ppa024999m	-3.63	0.042258194	*glyoxal oxidase-related protein*	Red ontogenesis	
ppa000762m	-3.63	0.008464523	*Leucine-rich receptor-like protein kinase family protein*	Red ontogenesis	
ppa008267m	-3.63	0.002578537	*Cysteine proteinases superfamily protein*	Red ontogenesis	
ppa005531m	-3.63	4.20E-05	*Protein kinase superfamily protein*	Red ontogenesis	
ppa005153m	-3.64	0.007537879	*Protein of Unknown Function (DUF239)*	Red ontogenesis	
ppa007955m	-3.64	7.79E-05	*N-MYC downregulated-like 2*	Red ontogenesis	
ppa010083m	-3.65	3.64E-05	*BCL-2-associated athanogene 4*	Red ontogenesis	
ppa004223m	-3.66	0.009026494	*Transmembrane amino acid transporter family protein*	Red ontogenesis	
ppa023849m	-3.66	0.001006336	*ubiquitin-like protein 5*	Red ontogenesis	
ppa007134m	-3.66	0.004415508	*Protein of unknown function, DUF547*	Red ontogenesis	
ppa010130m	-3.66	0.014849707	*Integrase-type DNA-binding superfamily protein*	Red ontogenesis	
ppa025804m	-3.67	0.006917169	*cytokinin response factor 2*	Red ontogenesis	
ppa019802m	-3.67	0.000104213	*gated outwardly-rectifying K+ channel*	Red ontogenesis	
ppa014688m	-3.68	1.41E-05	*TRICHOME BIREFRINGENCE-LIKE 43*	Red ontogenesis	
ppa008103m	-3.68	0.010046097	*Phosphoglycerate mutase family protein*	Red ontogenesis	
ppa023539m	-3.68	0.010906854	*amino acid permease 2*	Red ontogenesis	
ppa023171m	-3.68	0.010292089	*isopentenyltransferase 3*	Red ontogenesis	
ppa004844m	-3.68	0.013558087	*Exostosin family protein*	Red ontogenesis	
ppa005414m	-3.68	0.006202287	*cation exchanger 5*	Red ontogenesis	

ppa011761m	-3.69	0.005655952	*GATA type zinc finger transcription factor family protein*	Red ontogenesis	
ppa022272m	-3.69	0.013939101		Red ontogenesis	
ppa017769m	-3.69	0.000175203	*BURP domain-containing protein*	Red ontogenesis	
ppa002413m	-3.69	0.006611377	*Zinc finger (CCCH-type) family protein / RNA recognition motif (R*	Red ontogenesis	
ppb023961m	-3.69	0.035139296	*Protein of unknown function (DUF594)*	Red ontogenesis	
ppa006581m	-3.70	0.006603986	*Xanthine/uracil permease family protein*	Red ontogenesis	
ppa025098m	-3.70	0.02005606	*Pectin lyase-like superfamily protein*	Red ontogenesis	
ppa025232m	-3.70	8.50E-05	*alpha/beta-Hydrolases superfamily protein*	Red ontogenesis	
ppa005629m	-3.70	0.002768969	*basic helix-loop-helix (bHLH) DNA-binding superfamily protein*	Red ontogenesis	
ppa020549m	-3.70	0.00318059	*Protein phosphatase 2C family protein*	Red ontogenesis	
ppa014010m	-3.71	0.007893289	*arabinogalactan protein 16*	Red ontogenesis	
ppa004311m	-3.71	0.005873826	*cytochrome P450, family 94, subfamily B, polypeptide 3*	Red ontogenesis	
ppa025611m	-3.71	0.007994993	*Myzus persicae-induced lipase 1*	Red ontogenesis	
ppa023552m	-3.71	0.000389047	*alkenal reductase*	Red ontogenesis	
ppa007966m	-3.72	0.000183408	*EXORDIUM like 5*	Red ontogenesis	
ppa016212m	-3.74	0.017389107		Red ontogenesis	
ppa011642m	-3.74	0.007497442	*cyclin p4;1*	Red ontogenesis	
ppa021755m	-3.74	0.009056255	*serine carboxypeptidase-like 18*	Red ontogenesis	
ppa016976m	-3.74	0.00114415		Red ontogenesis	
ppa020464m	-3.75	0.042867999	*PHYTOSULFOKINE 3 PRECURSOR*	Red ontogenesis	
ppa002254m	-3.75	0.000209812	*Cellulose-synthase-like C5*	Red ontogenesis	
ppa004238m	-3.75	0.001725431	*Domain of unknown function (DUF23)*	Red ontogenesis	
ppa026926m	-3.75	0.017290239	*agenet domain-containing protein*	Red ontogenesis	
ppa006139m	-3.75	0.015035965	*DNA glycosylase superfamily protein*	Red ontogenesis	
ppa013602m	-3.76	0.000487193	*arabinogalactan protein 1*	Red ontogenesis	
ppa010228m	-3.78	0.006830228	*2-oxoglutarate (2OG) and Fe(II)-dependent oxygenase superfami*	Red ontogenesis	
ppa005613m	-3.78	0.000108011	*tubulin alpha-4 chain*	Red ontogenesis	
ppa019215m	-3.79	0.018312682	*Cytochrome P450 superfamily protein*	Red ontogenesis	
ppa004618m	-3.79	0.0002753	*serine carboxypeptidase-like 40*	Red ontogenesis	
ppa002694m	-3.80	0.000181792	*plant glycogenin-like starch initiation protein 2*	Red ontogenesis	
ppa006039m	-3.81	0.004344503	*zinc finger protein-related*	Red ontogenesis	
ppa010820m	-3.81	0.001848303	*early nodulin-like protein 18*	Red ontogenesis	
ppa018164m	-3.81	0.008252369		Red ontogenesis	
ppa022380m	-3.82	0.002372946	*B-box zinc finger family protein*	Red ontogenesis	
ppa002357m	-3.82	0.005421542	*Leucine-rich repeat protein kinase family protein*	Red ontogenesis	
ppa017614m	-3.83	0.000662615		Red ontogenesis	
ppa007005m	-3.83	0.015216161	*jasmonic acid carboxyl methyltransferase*	Red ontogenesis	
ppa013837m	-3.83	0.00978604	*SAUR-like auxin-responsive protein family*	Red ontogenesis	
ppa025978m	-3.83	0.007584646	*Cupredoxin superfamily protein*	Red ontogenesis	
ppa003968m	-3.83	0.005033974	*Tetratricopeptide repeat (TPR)-like superfamily protein*	Red ontogenesis	
ppa017822m	-3.84	0.016747969	*Ankyrin repeat family protein*	Red ontogenesis	
ppa004177m	-3.84	3.14E-06		Red ontogenesis	
ppa010836m	-3.84	0.001283177	*HSP20-like chaperones superfamily protein*	Red ontogenesis	
ppa002905m	-3.84	5.83E-05	*Rhamnogalacturonate lyase family protein*	Red ontogenesis	
ppa018358m	-3.85	0.006340787		Red ontogenesis	
ppa026717m	-3.85	0.001856513		Red ontogenesis	
ppa000796m	-3.85	0.005701619	*P-loop nucleoside triphosphate hydrolases superfamily protein wit*	Red ontogenesis	
ppa003389m	-3.85	0.004598776		Red ontogenesis	
ppa014446m	-3.86	0.003871948	*Protein of unknown function (DUF 3339)*	Red ontogenesis	
ppa001334m	-3.86	0.00958753	*beta-galactosidase 3*	Red ontogenesis	
ppa014614m	-3.86	0.006343028	*Uncharacterised protein family (UPF0497)*	Red ontogenesis	
ppa007250m	-3.86	0.001234755	*galacturonic acid kinase*	Red ontogenesis	
ppa012290m	-3.86	9.46E-05		Red ontogenesis	
ppa020645m	-3.87	0.009699853	*Haloacid dehalogenase-like hydrolase (HAD) superfamily protein*	Red ontogenesis	
ppa002547m	-3.87	0.005017466	*rhamnose biosynthesis 1*	Red ontogenesis	
ppa000821m	-3.87	0.005531465	*P-loop nucleoside triphosphate hydrolases superfamily protein wit*	Red ontogenesis	
ppa027011m	-3.88	0.040573713	*endoxyloglucan transferase A3*	Red ontogenesis	
ppa022581m	-3.88	0.011606676	*Zinc finger (C3HC4-type RING finger) family protein*	Red ontogenesis	
ppa001903m	-3.88	1.96E-05		Red ontogenesis	
ppa001727m	-3.89	0.001666915	*Subtilisin-like serine endopeptidase family protein*	Red ontogenesis	
ppa010555m	-3.90	0.003695792	*Cytochrome b561/ferric reductase transmembrane protein family*	Red ontogenesis	
ppa004043m	-3.90	6.36E-05	*galacturonosyltransferase 12*	Red ontogenesis	
ppa009365m	-3.91	0.012921064	*xyloglucan endotransglucosylase/hydrolase 10*	Red ontogenesis	
ppa020152m	-3.91	0.028159444	*plastid division1*	Red ontogenesis	
ppa017471m	-3.92	0.00371725	*Remorin family protein*	Red ontogenesis	
ppa016112m	-3.92	0.012490063	*GRAS family transcription factor*	Red ontogenesis	
ppa007096m	-3.93	0.000512269	*PQ-loop repeat family protein / transmembrane family protein*	Red ontogenesis	
ppa002756m	-3.93	0.010430019	*ARM repeat superfamily protein*	Red ontogenesis	
ppa020715m	-3.93	0.000685135		Red ontogenesis	
ppa000641m	-3.93	1.10E-05	*cellulose synthase A4*	Red ontogenesis	
ppa026981m	-3.94	0.00755291	*GDSL-like Lipase/Acylhydrolase superfamily protein*	Red ontogenesis	
ppa008035m	-3.94	0.007797447	*Pathogenesis-related thaumatin superfamily protein*	Red ontogenesis	
ppa016983m	-3.94	0.027544115	*Glucose-methanol-choline (GMC) oxidoreductase family protein*	Red ontogenesis	
ppa015817m	-3.94	0.00337908	*heat shock protein 70*	Red ontogenesis	
ppa012084m	-3.95	0.016212372		Red ontogenesis	
ppa006443m	-3.95	0.000306556	*SEC14 cytosolic factor family protein / phosphoglyceride transfer*	Red ontogenesis	
ppa002341m	-3.95	0.003270332	*Protein of unknown function (DUF740)*	Red ontogenesis	
ppa006355m	-3.95	0.000266296		Red ontogenesis	
ppa020558m	-3.95	0.005971935	*oxidoreductases, acting on NADH or NADPH*	Red ontogenesis	
ppa008295m	-3.96	7.47E-07	*NAD(P)-binding Rossmann-fold superfamily protein*	Red ontogenesis	Secondary Metabolism / Dihydroflavonols -Biotic Stress / Secondary metabolism involved into stress
ppa017015m	-3.96	0.000983507	*Eukaryotic aspartyl protease family protein*	Red ontogenesis	
ppa008896m	-3.96	5.32E-05	*Peroxidase superfamily protein*	Red ontogenesis	
ppa002732m	-3.96	2.61E-05	*Glycosyl hydrolases family 32 protein*	Red ontogenesis	
ppa011790m	-3.96	0.01090684	*Cytochrome b561/ferric reductase transmembrane protein family*	Red ontogenesis	
ppa023617m	-3.97	0.021972037	*hydroxyproline-rich glycoprotein family protein*	Red ontogenesis	
ppa003992m	-3.97	0.003225437	*glyoxal oxidase-related protein*	Red ontogenesis	
ppa003712m	-3.97	0.003472085	*polygalacturonase 2*	Red ontogenesis	
ppa004243m	-3.97	0.005322513	*3-ketoacyl-CoA synthase 2*	Red ontogenesis	
ppa022655m	-3.98	0.001760075		Red ontogenesis	
ppa009266m	-3.98	0.001518472		Red ontogenesis	
ppa025838m	-3.98	0.005128292	*AT hook motif DNA-binding family protein*	Red ontogenesis	
ppa007461m	-3.98	0.00027323	*Protein of unknown function, DUF642*	Red ontogenesis	
ppa010997m	-3.99	0.002579556		Red ontogenesis	
ppa008689m	-3.99	0.012763709	*Exostosin family protein*	Red ontogenesis	
ppa022264m	-3.99	8.28E-05	*vascular related NAC-domain protein 1*	Red ontogenesis	
ppa022001m	-3.99	0.018100096	*nitrate excretion transporter1*	Red ontogenesis	
ppa021144m	-4.00	0.001095268	*alpha carbonic anhydrase 1*	Red ontogenesis	
ppa022029m	-4.00	0.031859275	*ovate family protein 6*	Red ontogenesis	
ppa018259m	-4.00	0.008191595	*RNI-like superfamily protein*	Red ontogenesis	
ppa018181m	-4.01	0.007115273	*Matrixin family protein*	Red ontogenesis	
ppa017157m	-4.01	0.004004942	*monodehydroascorbate reductase 1*	Red ontogenesis	
ppa024325m	-4.01	0.00434126	*Matrixin family protein*	Red ontogenesis	
ppa003924m	-4.01	2.84E-05	*aspartic proteinase A1*	Red ontogenesis	
ppa024900m	-4.02	0.000389047	*2-oxoglutarate (2OG) and Fe(II)-dependent oxygenase superfami*	Red ontogenesis	
ppb025553m	-4.02	0.032614095	*Protein of unknown function, DUF547*	Red ontogenesis	
ppa020196m	-4.02	0.005227216		Red ontogenesis	
ppa011745m	-4.03	0.010277897	*Late embryogenesis abundant (LEA) hydroxyproline-rich glycopro*	Red ontogenesis	
ppb013299m	-4.04	5.11E-05	*terpene synthase 21*	Red ontogenesis	Biotic Stress / Secondary metabolism involved into stress
ppa007891m	-4.05	3.78E-05	*xylem cysteine peptidase 1*	Red ontogenesis	
ppa007546m	-4.05	0.004128579	*GDSL-like Lipase/Acylhydrolase superfamily protein*	Red ontogenesis	
ppa019060m	-4.07	0.043756228	*RING/U-box superfamily protein*	Red ontogenesis	
ppa015510m	-4.07	0.005385627	*strictosidine synthase-like 2*	Red ontogenesis	Biotic Stress / Secondary metabolism involved into stress
ppa001382m	-4.07	1.95E-05	*beta galactosidase 1*	Red ontogenesis	
ppa019862m	-4.08	0.016644912	*HSP20-like chaperones superfamily protein*	Red ontogenesis	
ppa000370m	-4.08	0.009699298		Red ontogenesis	
ppa022254m	-4.09	0.011000661	*TRAF-like family protein*	Red ontogenesis	
ppa021560m	-4.09	0.006438966	*NB-ARC domain-containing disease resistance protein*	Red ontogenesis	
ppa015753m	-4.09	0.040054861	*GATA transcription factor 12*	Red ontogenesis	
ppa025912m	-4.10	0.007774672	*WRKY DNA-binding protein 70*	Red ontogenesis	
ppa011192m	-4.10	0.004982943		Red ontogenesis	
ppa023074m	-4.10	0.005012031	*Glycosyl hydrolase family protein with chitinase insertion domain*	Red ontogenesis	
ppa023646m	-4.10	0.010144142	*early nodulin-like protein 9*	Red ontogenesis	
ppa026145m	-4.11	0.002755783		Red ontogenesis	
ppa020278m	-4.11	0.011045028		Red ontogenesis	
ppa002591m	-4.11	3.22E-06	*heat shock protein 70*	Red ontogenesis	
ppa016180m	-4.12	0.040393758	*Protein of unknown function (DUF 3339)*	Red ontogenesis	
ppa007982m	-4.13	0.00071636	*galactinol synthase 1*	Red ontogenesis	
ppa012383m	-4.13	0.006015243	*Family of unknown function (DUF662)*	Red ontogenesis	
ppa004428m	-4.13	0.002372946	*Eukaryotic aspartyl protease family protein*	Red ontogenesis	
ppa002088m	-4.13	0.001055444	*STRUBBELIG-receptor family 8*	Red ontogenesis	
ppa002598m	-4.13	2.08E-05	*heat shock protein 70*	Red ontogenesis	
ppa002645m	-4.13	0.000155002	*basic helix-loop-helix (bHLH) DNA-binding superfamily protein*	Red ontogenesis	
ppa016768m	-4.14	1.60E-05	*TRICHOME BIREFRINGENCE-LIKE 43*	Red ontogenesis	
ppa021258m	-4.14	0.015280807		Red ontogenesis	
ppa003051m	-4.14	5.97E-05	*Tetratricopeptide repeat (TPR)-like superfamily protein*	Red ontogenesis	
ppa006097m	-4.15	0.000206222	*Basic-leucine zipper (bZIP) transcription factor family protein*	Red ontogenesis	
ppa006439m	-4.15	0.002564105	*ovate family protein 2*	Red ontogenesis	
ppa020983m	-4.15	0.005602333		Red ontogenesis	
ppa016309m	-4.15	0.038270823	*Ankyrin repeat family protein*	Red ontogenesis	
ppa023327m	-4.17	0.002854028	*glycosyl hydrolase family 81 protein*	Red ontogenesis	
ppa014393m	-4.18	0.039828289		Red ontogenesis	
ppa006601m	-4.18	0.003289613		Red ontogenesis	
ppa024053m	-4.18	0.015470127	*serine carboxypeptidase-like 6*	Red ontogenesis	
ppa017363m	-4.19	0.003568039	*Ankyrin repeat family protein*	Red ontogenesis	
ppa024492m	-4.19	0.007457566	*Carbohydrate-binding X8 domain superfamily protein*	Red ontogenesis	Biotic Stress / Betaglucanase
ppa003559m	-4.20	0.002093803	*Plant protein of unknown function (DUF946)*	Red ontogenesis	
ppa003380m	-4.20	0.021172211	*terpene synthase 14*	Red ontogenesis	Biotic Stress / Secondary metabolism involved into stress
ppa002271m	-4.20	0.005282403	*beta-galactosidase 3*	Red ontogenesis	

ppa018188m	-4.21	0.006449783	OBF binding protein 4	Red ontogenesis	
ppa002978m	-4.21	0.003822436	basic helix-loop-helix (bHLH) DNA-binding superfamily protein	Red ontogenesis	
ppa026237m	-4.22	0.001167397	RING/U-box superfamily protein	Red ontogenesis	
ppa009534m	-4.23	3.00E-06	NmrA-like negative transcriptional regulator family protein	Red ontogenesis	Secondary Metabolism / Flavonols-Isoflavonoid -Biotic Stress / Secondary metabolism involved into stress
ppa006868m	-4.23	0.007836617	NAD(P)-binding Rossmann-fold superfamily protein	Red ontogenesis	
ppa019338m	-4.23	0.014143574	CCCH-type zinc fingerfamily protein with RNA-binding domain	Red ontogenesis	
ppa001661m	-4.23	0.000780005	Subtilase family protein	Red ontogenesis	
ppa023407m	-4.24	0.006521308	Reticulon family protein	Red ontogenesis	
ppa005208m	-4.24	0.001760075	Plant invertase/pectin methylesterase inhibitor superfamily	Red ontogenesis	
ppa017486m	-4.24	0.000254861	Plant protein of unknown function (DUF247)	Red ontogenesis	
ppa008179m	-4.25	0.0091171		Red ontogenesis	
ppa026307m	-4.26	0.001187145	Rhamnogalacturonate lyase family protein	Red ontogenesis	
ppa005869m	-4.26	0.001601869	Protein of unknown function (DUF3411)	Red ontogenesis	
ppa004569m	-4.27	0.012327301	cytochrome P450, family 82, subfamily G, polypeptide 1	Red ontogenesis	
ppa025376m	-4.27	0.004335399	UDP-Glycosyltransferase superfamily protein	Red ontogenesis	
ppa027202m	-4.28	0.003392053	GDSL-like Lipase/Acylhydrolase superfamily protein	Red ontogenesis	
ppa002538m	-4.28	0.002642549	Rhamnogalacturonate lyase family protein	Red ontogenesis	
ppb011681m	-4.28	0.003982253	Major facilitator superfamily protein	Red ontogenesis	
ppa010958m	-4.29	5.23E-05	Bifunctional inhibitor/lipid-transfer protein/seed storage 2S albumi	Red ontogenesis	
ppa019713m	-4.29	0.000903097	nucleolar essential protein-related	Red ontogenesis	
ppa013464m	-4.29	0.040938883	Bifunctional inhibitor/lipid-transfer protein/seed storage 2S albumi	Red ontogenesis	
ppa007155m	-4.31	0.000777908	Protein of unknown function (DUF3049)	Red ontogenesis	
ppa005818m	-4.32	0.006897971	Pectin lyase-like superfamily protein	Red ontogenesis	
ppa013244m	-4.32	0.002242913	Ran BP2/NZF zinc finger-like superfamily protein	Red ontogenesis	
ppa002612m	-4.33	0.002664995	Integrase-type DNA-binding superfamily protein	Red ontogenesis	
ppa003865m	-4.33	4.43E-05	Protein of unknown function (DUF616)	Red ontogenesis	
ppa015435m	-4.33	0.002891821	serine carboxypeptidase-like 6	Red ontogenesis	
ppa000338m	-4.33	3.78E-05	P-glycoprotein 9	Red ontogenesis	
ppa016481m	-4.34	0.015340766		Red ontogenesis	
ppa000192m	-4.34	0.005199065	multidrug resistance-associated protein 14	Red ontogenesis	
ppa008966m	-4.35	0.001101288		Red ontogenesis	
ppa018962m	-4.35	0.002005598	Leucine-rich repeat (LRR) family protein	Red ontogenesis	
ppa003524m	-4.35	0.003790037	acyl-CoA oxidase 1	Red ontogenesis	
ppa003520m	-4.35	0.000135737	cell elongation protein / DWARF1 / DIMINUTO (DIM)	Red ontogenesis	
ppa011275m	-4.35	0.003860007	Cupredoxin superfamily protein	Red ontogenesis	
ppa000838m	-4.35	7.98E-06	Leucine-rich receptor-like protein kinase family protein	Red ontogenesis	
ppa011554m	-4.36	0.009760216	Transcriptional factor B3 family protein	Red ontogenesis	
ppa020432m	-4.36	0.014704129	O-Glycosyl hydrolases family 17 protein	Red ontogenesis	Biotic Stress / Betaglucanase
ppa018224m	-4.37	7.19E-05	Pectin lyase-like superfamily protein	Red ontogenesis	
ppa004875m	-4.37	0.002083609	HXXXD-type acyl-transferase family protein	Red ontogenesis	Secondary Metabolism / Phenylpropanoids - Biotic Stress / Secondary metabolism involved into stress
ppa011156m	-4.38	0.000134884	17.6 kDa class II heat shock protein	Red ontogenesis	
ppa025287m	-4.38	0.021503477	HSP20-like chaperones superfamily protein	Red ontogenesis	
ppa004909m	-4.38	0.002835129	UDP-glucosyl transferase 85A2	Red ontogenesis	
ppa014618m	-4.38	5.13E-05	Protein of Unknown Function (DUF239)	Red ontogenesis	
ppa018520m	-4.40	0.045963356	acyl carrier protein 5	Red ontogenesis	
ppa014213m	-4.40	0.024951919		Red ontogenesis	
ppa008302m	-4.40	0.000647573	chloroplastic lipocalin	Red ontogenesis	
ppa016247m	-4.40	0.000309383	wall-associated kinase 2	Red ontogenesis	
ppa025193m	-4.40	0.008217481		Red ontogenesis	
ppa018694m	-4.41	0.004281024	HSP20-like chaperones superfamily protein	Red ontogenesis	
ppa012320m	-4.42	0.008562368	PEBP (phosphatidylethanolamine-binding protein) family protein	Red ontogenesis	
ppa001743m	-4.42	0.001227991	Calcium-dependent lipid-binding (CaLB domain) plant phosphorib	Red ontogenesis	
ppa022122m	-4.42	0.00725116	crinkly4	Red ontogenesis	
ppa002812m	-4.42	0.002026108	Leucine-rich repeat protein kinase family protein	Red ontogenesis	
ppa020302m	-4.43	0.013268004	phy rapidly regulated 2	Red ontogenesis	
ppa025548m	-4.43	0.016072354	Domain of unknown function (DUF23)	Red ontogenesis	
ppa015812m	-4.44	0.021791191	chitinase A	Red ontogenesis	
ppa026775m	-4.45	0.00488075	FAD-binding Berberine family protein	Red ontogenesis	
ppa021800m	-4.45	0.000212162		Red ontogenesis	
ppa019426m	-4.46	0.004463681	ADP-glucose pyrophosphorylase small subunit 2	Red ontogenesis	
ppa011954m	-4.46	1.45E-05	myb domain protein 4	Red ontogenesis	
ppa009108m	-4.46	0.003434858		Red ontogenesis	
ppa021498m	-4.47	2.21E-05	fatty alcohol oxidase 3	Red ontogenesis	
ppa008733m	-4.47	7.81E-06		Red ontogenesis	
ppa003540m	-4.48	0.001098873	polyamine oxidase 5	Red ontogenesis	
ppa013265m	-4.48	0.007817811	hydroxyproline-rich glycoprotein family protein	Red ontogenesis	
ppa004976m	-4.48	0.001835757	O-Glycosyl hydrolases family 17 protein	Red ontogenesis	Biotic Stress / Betaglucanase
ppa021580m	-4.48	0.015814381	annexin 1	Red ontogenesis	
ppa019429m	-4.49	0.017220577	Glycosyl hydrolase family 35 protein	Red ontogenesis	
ppa011562m	-4.50	0.005337638	putative endonuclease or glycosyl hydrolase with C2H2-type zinc	Red ontogenesis	
ppa015010m	-4.50	0.01153622	geranylgeranyl reductase	Red ontogenesis	Biotic Stress / Secondary metabolism involved into stress
ppa019022m	-4.50	0.007754944	Disease resistance-responsive (dirigent-like protein) family protei	Red ontogenesis	Biotic Stress / PR-proteins
ppa001824m	-4.50	0.000605823	S-locus lectin protein kinase family protein	Red ontogenesis	
ppa001571m	-4.50	0.00175258	Exostosin family protein	Red ontogenesis	
ppa002417m	-4.50	1.52E-05	rhamnose biosynthesis 1	Red ontogenesis	
ppa017136m	-4.51	0.000719262	myb domain protein 12	Red ontogenesis	
ppa021281m	-4.51	0.002112901	Pollen Ole e 1 allergen and extensin family protein	Red ontogenesis	
ppa003101m	-4.52	0.002173573	Protein of unknown function, DUF547	Red ontogenesis	
ppa012615m	-4.52	0.002152559	Polyketide cyclase/dehydrase and lipid transport superfamily prot	Red ontogenesis	
ppa026255m	-4.52	0.001826465	heat shock protein 70	Red ontogenesis	
ppa021738m	-4.53	0.049854225		Red ontogenesis	
ppa004291m	-4.53	0.000539027	MATE efflux family protein	Red ontogenesis	
ppa009812m	-4.53	0.00210521	Integrase-type DNA-binding superfamily protein	Red ontogenesis	
ppb017373m	-4.53	0.013508572	Protein of unknown function (DUF1677)	Red ontogenesis	
ppa023388m	-4.54	0.001646426	PAK-box/P21-Rho-binding family protein	Red ontogenesis	Biotic Stress / PR-proteins
ppa012975m	-4.54	0.002755662		Red ontogenesis	
ppa023858m	-4.54	0.007329363		Red ontogenesis	
ppa003034m	-4.55	0.000225047	ROP interactive partner 3	Red ontogenesis	
ppa004226m	-4.55	0.014705124	beta glucosidase 13	Red ontogenesis	
ppa016265m	-4.56	0.001712647	Plant protein of unknown function (DUF247)	Red ontogenesis	
ppa002850m	-4.56	0.001693435	microtubule-associated proteins 70-5	Red ontogenesis	
ppa007765m	-4.57	0.000155863	Leucine-rich repeat (LRR) family protein	Red ontogenesis	
ppa019516m	-4.57	0.002563637	Cysteine/Histidine-rich C1 domain family protein	Red ontogenesis	
ppa004515m	-4.57	0.000108948	dehydroquinate dehydratase, putative / shikimate dehydrogenase	Red ontogenesis	
ppa004068m	-4.58	9.13E-06	Class-II DAHP synthetase family protein	Red ontogenesis	
ppa014919m	-4.58	0.001135352	Glycoprotein membrane precursor GPI-anchored	Red ontogenesis	
ppa021980m	-4.58	0.00173942	3-ketoacyl-CoA synthase 19	Red ontogenesis	
ppa012691m	-4.60	0.021102932	HSP20-like chaperones superfamily protein	Red ontogenesis	
ppa023961m	-4.60	0.000155003	Subtilase family protein	Red ontogenesis	
ppa020240m	-4.60	1.07E-06	PLAC8 family protein	Red ontogenesis	
ppa005206m	-4.61	6.94E-06	O-Glycosyl hydrolases family 17 protein	Red ontogenesis	Biotic Stress / Betaglucanase
ppa003105m	-4.61	0.006136744	Leucine-rich repeat protein kinase family protein	Red ontogenesis	
ppa017629m	-4.62	0.000141042	PLAC8 family protein	Red ontogenesis	
ppa021513m	-4.62	2.69E-05	Glycosyl hydrolase family 38 protein	Red ontogenesis	
ppa014571m	-4.62	0.017820651	B-cell receptor-associated protein 31-like	Red ontogenesis	
ppb011733m	-4.63	0.002307002	ankyrin repeat family protein	Red ontogenesis	
ppa001484m	-4.63	7.79E-05	Ribonuclease P protein subunit P38-related	Red ontogenesis	
ppa023652m	-4.64	0.003451339	Root hair defective 3 GTP-binding protein (RHD3)	Red ontogenesis	
ppa010908m	-4.64	0.002920989	myb domain protein 66	Red ontogenesis	
ppa014687m	-4.65	9.47E-05	ROP-interactive CRIB motif-containing protein 4	Red ontogenesis	
ppa023759m	-4.65	0.01382798		Red ontogenesis	
ppa001674m	-4.65	8.30E-05	Subtilase family protein	Red ontogenesis	
ppa020764m	-4.66	0.003663896	Gibberellin-regulated family protein	Red ontogenesis	
ppa025896m	-4.67	0.001601869	HSP20-like chaperones superfamily protein	Red ontogenesis	
ppa024894m	-4.67	0.003827267	RNA-binding protein	Red ontogenesis	
ppa004437m	-4.67	0.01563522	Plant invertase/pectin methylesterase inhibitor superfamily	Red ontogenesis	
ppa019976m	-4.68	0.001393118		Red ontogenesis	
ppa002781m	-4.68	0.000750321	Leucine-rich repeat protein kinase family protein	Red ontogenesis	
ppa008831m	-4.69	0.022849209	Peroxidase superfamily protein	Red ontogenesis	
ppa026419m	-4.69	0.004502012		Red ontogenesis	
ppa023468m	-4.69	0.000215187	wall associated kinase 4	Red ontogenesis	
ppa026995m	-4.70	0.005230442	cytochrome P450, family 705, subfamily A, polypeptide 33	Red ontogenesis	
ppa006621m	-4.70	4.42E-05	ethylene-dependent gravitropism-deficient and yellow-green-like	Red ontogenesis	
ppb018490m	-4.71	0.015679367		Red ontogenesis	
ppa019204m	-4.71	0.021432713	Gibberellin-regulated family protein	Red ontogenesis	
ppa013476m	-4.71	3.74E-05	thioredoxin H-type 1	Red ontogenesis	
ppa005789m	-4.73	0.000903223	Eukaryotic aspartyl protease family protein	Red ontogenesis	
ppa012692m	-4.73	8.73E-06	HSP20-like chaperones superfamily protein	Red ontogenesis	
ppa004095m	-4.73	0.013813178	cytochrome P450, family 82, subfamily G, polypeptide 1	Red ontogenesis	
ppa021720m	-4.74	0.028945081	Auxin-responsive family protein	Red ontogenesis	
ppa017444m	-4.74	0.000235658	FAD-binding Berberine family protein	Red ontogenesis	
ppa026062m	-4.75	6.42E-05		Red ontogenesis	
ppa012702m	-4.76	7.83E-06	HSP20-like chaperones superfamily protein	Red ontogenesis	
ppa015786m	-4.76	0.003330615	NAC 007	Red ontogenesis	
ppa011209m	-4.77	0.001038769	Plant-specific transcription factor YABBY family protein	Red ontogenesis	
ppa025923m	-4.77	0.006806194	NAD(P)-binding Rossmann-fold superfamily protein	Red ontogenesis	
ppa026704m	-4.77	0.0022805	WRKY DNA-binding protein 70	Red ontogenesis	
ppa005970m	-4.77	0.013342432	Sec14p-like phosphatidylinositol transfer family protein	Red ontogenesis	
ppa001135m	-4.77	0.002377595	sucrose synthase 6	Red ontogenesis	
ppa011519m	-4.78	2.69E-05	Lactoylglutathione lyase / glyoxalase I family protein	Red ontogenesis	
ppa010781m	-4.78	7.69E-05	Pathogenesis-related thaumatin superfamily protein	Red ontogenesis	
ppa012052m	-4.79	0.000828789	RING/U-box superfamily protein	Red ontogenesis	
ppa019350m	-4.80	0.01660294	TRICHOME BIREFRINGENCE-LIKE 19	Red ontogenesis	
ppa019553m	-4.80	0.017586056	Plant protein of unknown function (DUF247)	Red ontogenesis	
ppa019292m	-4.80	0.00061451	3-ketoacyl-CoA synthase 19	Red ontogenesis	
ppa007826m	-4.80	8.01E-05	peroxidase 2	Red ontogenesis	
ppa022256m	-4.80	2.69E-05	Eukaryotic aspartyl protease family protein	Red ontogenesis	
ppb021634m	-4.80	0.015188626	tracheary element differentiation-related 6	Red ontogenesis	

ppa001798m	-4.80	0.002582709	Subtilase family protein	Red ontogenesis	
ppa019275m	-4.81	0.001203974	AGC (cAMP-dependent, cGMP-dependent and protein kinase C)	Red ontogenesis	
ppa018856m	-4.81	0.001468947	O-acyltransferase (WSD1-like) family protein	Red ontogenesis	
ppa026811m	-4.81	0.029513984		Red ontogenesis	
ppa026226m	-4.81	0.000258813	FAD-binding Berberine family protein	Red ontogenesis	
ppa003971m	-4.81	2.09E-05	glycerol-3-phosphate acyltransferase 3	Red ontogenesis	
ppa024897m	-4.82	0.00503752	cyclin p3;1	Red ontogenesis	
ppa024745m	-4.83	0.002358587	Plant protein of unknown function (DUF827)	Red ontogenesis	
ppa019735m	-4.84	0.002906781		Red ontogenesis	
ppb019990m	-4.84	0.001884646	UDP-Glycosyltransferase superfamily protein	Red ontogenesis	
ppa006612m	-4.84	0.004307632	Galactosyltransferase family protein	Red ontogenesis	
ppa013863m	-4.84	0.002011547	SAUR-like auxin-responsive protein family	Red ontogenesis	
ppa003643m	-4.84	0.000543372	RHO guanyl-nucleotide exchange factor 14	Red ontogenesis	
ppa003577m	-4.85	0.000348266	laccase 11	Red ontogenesis	Biotic Stress / Secondary metabolism involved into stress
ppa011878m	-4.85	0.006026341	Embryo-specific protein 3, (ATS3)	Red ontogenesis	
ppa002258m	-4.86	0.003285286	Rhamnogalacturonate lyase family protein	Red ontogenesis	
ppa011163m	-4.86	4.60E-06	arabinogalactan protein 18	Red ontogenesis	
ppa018008m	-4.87	0.001295615	Pathogenesis-related thaumatin superfamily protein	Red ontogenesis	
ppb018294m	-4.87	0.000753127	O-acyltransferase (WSD1-like) family protein	Red ontogenesis	
ppb013713m	-4.88	0.003472194		Red ontogenesis	
ppa018295m	-4.89	0.017608068	P-loop containing nucleoside triphosphate hydrolases superfamily	Red ontogenesis	
ppa001604m	-4.90	0.000719259	Leucine-rich repeat receptor-like protein kinase family protein	Red ontogenesis	
ppa002358m	-4.90	0.000187799	ABC-2 type transporter family protein	Red ontogenesis	
ppa016501m	-4.90	2.23E-05	subtilase family protein	Red ontogenesis	
ppa007240m	-4.91	0.002606313	Papain family cysteine protease	Red ontogenesis	
ppa022222m	-4.92	0.002643248	AWPM-19-like family protein	Red ontogenesis	
ppa023784m	-4.92	0.00538016	Pectin lyase-like superfamily protein	Red ontogenesis	
ppa010264m	-4.92	1.42E-05	NC domain-containing protein-related	Red ontogenesis	
ppa020310m	-4.93	8.18E-06	PLAC8 family protein	Red ontogenesis	
ppa012712m	-4.94	0.004913145	HVA22-like protein F	Red ontogenesis	
ppa004380m	-4.94	0.000715476	B-S glucosidase 44	Red ontogenesis	
ppa009350m	-4.94	0.001949343	xyloglucan endotransglucosylase/hydrolase 9	Red ontogenesis	
ppa023990m	-4.94	0.006681244	UDP-glucosyltransferase 74F2	Red ontogenesis	
ppa021567m	-4.94	0.001813499	GDSL-like Lipase/Acylhydrolase superfamily protein	Red ontogenesis	
ppa019163m	-4.96	0.002000928	17.6 kDa class II heat shock protein	Red ontogenesis	
ppa017606m	-4.97	0.004814646	sucrose synthase 6	Red ontogenesis	
ppa003411m	-4.98	1.31E-05	Plant L-ascorbate oxidase	Red ontogenesis	Biotic Stress / Secondary metabolism involved into stress
ppb015715m	-4.98	0.027024085	receptor like protein 51	Red ontogenesis	
ppa000236m	-4.98	0.001755567	pleiotropic drug resistance 4	Red ontogenesis	
ppa004944m	-4.99	0.023837892	3-ketoacyl-CoA synthase 19	Red ontogenesis	
ppa001082m	-5.01	0.000425217	lipoxygenase 3	Red ontogenesis	
ppa021990m	-5.01	0.024177789		Red ontogenesis	
ppa008167m	-5.01	0.001083272	Nucleotide-sugar transporter family protein	Red ontogenesis	
ppa016155m	-5.01	2.93E-05	Plant protein of unknown function (DUF247)	Red ontogenesis	
ppa012620m	-5.02	4.20E-05	HSP20-like chaperones superfamily protein	Red ontogenesis	
ppa022086m	-5.03	0.026376329	Heavy metal transport/detoxification superfamily protein	Red ontogenesis	
ppb015877m	-5.05	0.002131497	SAUR-like auxin-responsive protein family	Red ontogenesis	
ppa004109m	-5.05	0.000104947	Leucine-rich repeat (LRR) family protein	Red ontogenesis	
ppa024552m	-5.05	0.005595402	ubiquitin-specific protease 12	Red ontogenesis	
ppa020971m	-5.07	0.01490622	RHOMBOID-like protein 5	Red ontogenesis	
ppa026680m	-5.07	0.000974657		Red ontogenesis	
ppa023335m	-5.08	0.001391135	Cytochrome P450 superfamily protein	Red ontogenesis	
ppa010831m	-5.08	2.49E-06	Glutathione S-transferase family protein	Red ontogenesis	Biotic Stress / Glutathione-S-Transferase
ppa016605m	-5.10	0.004484808	zeaxanthin epoxidase (ZEP) (ABA1)	Red ontogenesis	
ppa014189m	-5.12	0.003065951	Gibberellin-regulated family protein	Red ontogenesis	
ppa015879m	-5.13	2.18E-05	O-acyltransferase (WSD1-like) family protein	Red ontogenesis	
ppa004236m	-5.14	0.000205102	Plant protein of unknown function (DUF828)	Red ontogenesis	
ppa007015m	-5.15	0.00212101	Heavy metal transport/detoxification superfamily protein	Red ontogenesis	
ppa019321m	-5.16	0.000994682	DNA binding;ATP binding	Red ontogenesis	
ppa006773m	-5.16	0.003518082	CYCLIN D3;1	Red ontogenesis	
ppa005454m	-5.17	0.005598653	3-ketoacyl-CoA synthase 19	Red ontogenesis	
ppa022332m	-5.18	0.002443899	HXXXD-type acyl-transferase family protein	Red ontogenesis	Secondary metabolism / Anthocyanins - Biotic Stress / Secondary metabolism involved into stress
ppa012348m	-5.19	8.30E-05	Protein of unknown function, DUF538	Red ontogenesis	
ppa021107m	-5.19	0.001315217	HSP20-like chaperones superfamily protein	Red ontogenesis	
ppa014737m	-5.20	0.000884965	PAPA-1-like family protein / zinc finger (HIT type) family protein	Red ontogenesis	
ppb015420m	-5.20	0.000488369	Terpenoid cyclases/Protein prenyltransferases superfamily protei	Red ontogenesis	Biotic Stress / Secondary metabolism involved into stress
ppa010954m	-5.20	0.000150253	ferredoxin-related	Red ontogenesis	
ppa020867m	-5.21	0.000288732	UDP-Glycosyltransferase superfamily protein	Red ontogenesis	
ppa003285m	-5.22	6.94E-06	pectin methylesterase 3	Red ontogenesis	
ppa025288m	-5.23	0.00132617	IQ-domain 26	Red ontogenesis	
ppa010383m	-5.23	0.00752108	LOB domain-containing protein 18	Red ontogenesis	
ppa011735m	-5.24	0.002766194	Integrase-type DNA-binding superfamily protein	Red ontogenesis	
ppa017898m	-5.25	0.000373158	ankyrin repeat family protein	Red ontogenesis	
ppa025053m	-5.25	0.000422024	nodulin MtN21 /EamA-like transporter family protein	Red ontogenesis	
ppa004056m	-5.26	0.001847077	TRICHOME BIREFRINGENCE-LIKE 6	Red ontogenesis	
ppa009088m	-5.27	0.000432098	NmrA-like negative transcriptional regulator family protein	Red ontogenesis	Secondary Metabolism / Flavonols-Isoflavonoid -Biotic Stress / Secondary metabolism involved into stress
ppa021616m	-5.27	0.000570987	HSP20-like chaperones superfamily protein	Red ontogenesis	
ppa017673m	-5.27	0.000483266	kinesin-like protein 1	Red ontogenesis	
ppa024465m	-5.28	0.004525075	COBRA-like extracellular glycosyl-phosphatidyl inositol-anchored	Red ontogenesis	
ppa017529m	-5.28	0.000460626	REF4-related 1	Red ontogenesis	
ppa002172m	-5.31	0.001447528	subtilase family protein	Red ontogenesis	
ppa023699m	-5.31	0.001028836		Red ontogenesis	
ppa015093m	-5.32	0.000848881	Cyclin D6;1	Red ontogenesis	
ppa022226m	-5.34	0.024060204	S-adenosyl-L-methionine-dependent methyltransferases superfar	Red ontogenesis	
ppa005150m	-5.34	0.000306556	6-phosphogluconate dehydrogenase family protein	Red ontogenesis	
ppa020173m	-5.35	0.001026711	dsRNA-binding protein 5	Red ontogenesis	
ppa022053m	-5.36	0.002564105	O-acyltransferase (WSD1-like) family protein	Red ontogenesis	
ppa013100m	-5.37	0.000239377		Red ontogenesis	
ppa015939m	-5.37	1.26E-05	UDP-glucosyl transferase 78D2	Red ontogenesis	
ppb010066m	-5.37	0.000172366		Red ontogenesis	
ppa013692m	-5.39	0.046851546	GAST1 protein homolog 4	Red ontogenesis	
ppa026814m	-5.39	0.016890726	MATE efflux family protein	Red ontogenesis	
ppa016669m	-5.40	0.001127409	P-loop containing nucleoside triphosphate hydrolases superfamily	Red ontogenesis	
ppa012205m	-5.40	0.002088259		Red ontogenesis	
ppa022094m	-5.41	0.00111615		Red ontogenesis	
ppa022459m	-5.42	0.001571188	TRICHOME BIREFRINGENCE-LIKE 38	Red ontogenesis	
ppa023451m	-5.44	0.035007398		Red ontogenesis	
ppa015151m	-5.44	8.66E-07	HSP20-like chaperones superfamily protein	Red ontogenesis	
ppb012860m	-5.45	0.009215449		Red ontogenesis	
ppa002275m	-5.45	0.00044744	Cellulose-synthase-like C5	Red ontogenesis	
ppa011318m	-5.47	0.000208434	Plant invertase/pectin methylesterase inhibitor superfamily protein	Red ontogenesis	
ppa026118m	-5.47	0.009056255		Red ontogenesis	
ppa022833m	-5.48	0.00430015	18S pre-ribosomal assembly protein gar2-related	Red ontogenesis	
ppb022195m	-5.48	0.0043567	TRAF-like family protein	Red ontogenesis	
ppa003917m	-5.48	2.08E-06	Eukaryotic aspartyl protease family protein	Red ontogenesis	
ppa007411m	-5.48	0.000630493	GDSL-like Lipase/Acylhydrolase superfamily protein	Red ontogenesis	
ppa016757m	-5.49	0.000788197	beta glucosidase 17	Red ontogenesis	
ppa021488m	-5.50	0.000221999		Red ontogenesis	
ppa026023m	-5.50	0.000777908	protein kinase 2B	Red ontogenesis	
ppa015344m	-5.50	2.36E-07	HSP20-like chaperones superfamily protein	Red ontogenesis	
ppa023387m	-5.50	0.001326085	serine carboxypeptidase-like 40	Red ontogenesis	
ppa015634m	-5.52	0.000346651	Basic helix-loop-helix (bHLH) DNA-binding family protein	Red ontogenesis	
ppa020338m	-5.53	0.012795587	BED zinc finger ;hAT family dimerisation domain	Red ontogenesis	
ppa003164m	-5.53	0.000773829	microtubule-associated protein 65-8	Red ontogenesis	
ppa005457m	-5.54	0.00079445	serine carboxypeptidase-like 45	Red ontogenesis	
ppa024671m	-5.54	0.012299308		Red ontogenesis	
ppa014596m	-5.55	0.000228687	Cytochrome P450 superfamily protein	Red ontogenesis	
ppa025159m	-5.55	0.000128978	glycosyl hydrolase 9B18	Red ontogenesis	
ppa027081m	-5.55	0.017298685		Red ontogenesis	
ppa002536m	-5.56	0.000487095	receptor-like kinase 1	Red ontogenesis	
ppa007530m	-5.57	0.003847505	Leucine-rich repeat (LRR) family protein	Red ontogenesis	
ppa002319m	-5.57	0.000283261	RHO guanyl-nucleotide exchange factor 7	Red ontogenesis	
ppa005920m	-5.58	0.00307225	TRICHOME BIREFRINGENCE-LIKE 34	Red ontogenesis	
ppa008503m	-5.58	4.82E-05	Peroxidase superfamily protein	Red ontogenesis	
ppa025166m	-5.59	0.000253335	cytochrome P450, family 94, subfamily C, polypeptide 1	Red ontogenesis	
ppa006726m	-5.60	0.000162608	FASCICLIN-like arabinogalactan 2	Red ontogenesis	
ppa020413m	-5.61	0.004251202		Red ontogenesis	
ppa002893m	-5.62	6.17E-05	Fatty acid hydroxylase superfamily	Red ontogenesis	Biotic Stress / Secondary metabolism involved into stress
ppa019404m	-5.63	0.003945234	carboxyesterase 18	Red ontogenesis	
ppb023607m	-5.63	0.0226419	SAUR-like auxin-responsive protein family	Red ontogenesis	
ppa001168m	-5.64	1.42E-05	alpha-xylosidase 1	Red ontogenesis	
ppa023135m	-5.66	0.015197111		Red ontogenesis	
ppa003918m	-5.66	0.000195919	cellulose synthase-like A02	Red ontogenesis	
ppa016050m	-5.67	0.001799813	cytochrome P450, family 82, subfamily G, polypeptide 1	Red ontogenesis	
ppa010418m	-5.68	0.002657483	Pathogenesis-related thaumatin superfamily protein	Red ontogenesis	
ppa021753m	-5.69	0.000133204	cell wall / vacuolar inhibitor of fructosidase 2	Red ontogenesis	
ppa008629m	-5.69	0.005421341		Red ontogenesis	
ppa009972m	-5.70	0.002854968		Red ontogenesis	
ppa025685m	-5.71	0.002596111	receptor like protein 55	Red ontogenesis	
ppa016111m	-5.71	0.001502683		Red ontogenesis	
ppa013704m	-5.72	0.011380332	Gibberellin-regulated family protein	Red ontogenesis	
ppa004172m	-5.72	0.001997797	glycosyl hydrolase 9B8	Red ontogenesis	
ppa008850m	-5.73	0.005425499	Dof-type zinc finger DNA-binding family protein	Red ontogenesis	
ppa004600m	-5.73	0.033827573	Terpenoid cyclases/Protein prenyltransferases superfamily protei	Red ontogenesis	Biotic Stress / Secondary metabolism involved into stress
ppa026375m	-5.76	0.000233581	Leucine-rich repeat protein kinase family protein	Red ontogenesis	
ppa013960m	-5.76	0.001000983	Plant protein 1589 of unknown function	Red ontogenesis	
ppa005749m	-5.78	0.01411383	purple acid phosphatase 22	Red ontogenesis	
ppa001818m	-5.78	0.00111615	phospholipase D alpha 1	Red ontogenesis	

ppa004126m	-5.79	6.32E-06	*Galactose oxidase/kelch repeat superfamily protein*	Red ontogenesis	
ppa008643m	-5.80	9.06E-05	*chitinase-like protein 2*	Red ontogenesis	
ppa004724m	-5.81	0.000108769	*ROP guanine nucleotide exchange factor 5*	Red ontogenesis	
ppa009726m	-5.81	0.004273155	*ABI-1-like 1*	Red ontogenesis	
ppa007249m	-5.81	0.000343582	*Protein kinase superfamily protein*	Red ontogenesis	
ppa019395m	-5.81	0.038797473	*Carbohydrate-binding X8 domain superfamily protein*	Red ontogenesis	Biotic Stress / Betaglucanase
ppa012886m	-5.84	0.00043728	*Polyketide cyclase/dehydrase and lipid transport superfamily prot*	Red ontogenesis	
ppa000889m	-5.87	0.000138863	*Leucine-rich repeat protein kinase family protein*	Red ontogenesis	
ppa008891m	-5.93	0.008204302	*Aldolase-type TIM barrel family protein*	Red ontogenesis	
ppa004373m	-5.93	1.15E-06	*cytochrome P450, family 77, subfamily A, polypeptide 4*	Red ontogenesis	
ppa023347m	-5.93	0.033997219	*GDSL-like Lipase/Acylhydrolase superfamily protein*	Red ontogenesis	
ppa015648m	-5.94	0.001113705	*Dof-type zinc finger DNA-binding family protein*	Red ontogenesis	
ppa012444m	-5.96	0.000282	*Plant invertase/pectin methylesterase inhibitor superfamily protei*	Red ontogenesis	
ppa020430m	-5.96	1.66E-06	*subtilase family protein*	Red ontogenesis	
ppa004300m	-5.97	0.000100302	*Plant invertase/pectin methylesterase inhibitor superfamily*	Red ontogenesis	
ppa008951m	-5.97	0.000244823	*TRAF-like family protein*	Red ontogenesis	
ppa019260m	-5.98	0.020765399	*Polynucleotidyl transferase, ribonuclease H-like superfamily prote*	Red ontogenesis	
ppa006158m	-6.00	2.48E-05	*FASCICLIN-like arabinogalactan protein 8*	Red ontogenesis	
ppa012771m	-6.01	0.013233959	*Heavy metal transport/detoxification superfamily protein*	Red ontogenesis	
ppa014773m	-6.01	0.000653557		Red ontogenesis	
ppa010041m	-6.02	0.00018577	*expansin B3*	Red ontogenesis	
ppa013615m	-6.02	3.75E-05		Red ontogenesis	
ppa022831m	-6.02	0.013334005	*beta glucosidase 17*	Red ontogenesis	
ppa010289m	-6.03	0.00020718	*Plant protein of unknown function (DUF828)*	Red ontogenesis	
ppa020084m	-6.03	0.000175287	*Plant protein of unknown function (DUF827)*	Red ontogenesis	
ppa021836m	-6.04	0.000175205	*3-ketoacyl-CoA synthase 3*	Red ontogenesis	
ppa013195m	-6.04	0.00028017	*ralf-like 34*	Red ontogenesis	
ppa025603m	-6.04	0.002662787	*O-acyltransferase (WSD1-like) family protein*	Red ontogenesis	
ppa007138m	-6.05	0.001717853	*Serine/threonine-protein kinase WNK (With No Lysine)-related*	Red ontogenesis	
ppa017145m	-6.06	9.00E-05	*Transducin/WD40 repeat-like superfamily protein*	Red ontogenesis	
ppa006897m	-6.08	1.76E-06	*GDSL-like Lipase/Acylhydrolase superfamily protein*	Red ontogenesis	
ppa003433m	-6.08	0.000112246	*pectin methylesterase 61*	Red ontogenesis	
ppa000363m	-6.09	1.21E-05	*P-glycoprotein 13*	Red ontogenesis	
ppa007777m	-6.09	0.001122307	*galacturonosyltransferase-like 2*	Red ontogenesis	
ppa026934m	-6.09	0.001896047	*HXXXD-type acyl-transferase family protein*	Red ontogenesis	Secondary Metabolism / Phenylpropanoids - Biotic Stress / Secondary metabolism involved into stress
ppa015286m	-6.12	0.000401575	*PLAC8 family protein*	Red ontogenesis	
ppa021557m	-6.13	0.008612523	*Transducin/WD40 repeat-like superfamily protein*	Red ontogenesis	
ppa002683m	-6.13	9.69E-06	*Leucine-rich repeat protein kinase family protein*	Red ontogenesis	
ppa014001m	-6.14	0.002345175	*pathogenesis-related protein-1-like*	Red ontogenesis	
ppa006626m	-6.14	0.000140772	*Galactosyltransferase family protein*	Red ontogenesis	
ppa020139m	-6.15	0.001462275	*NAC 007*	Red ontogenesis	
ppa016054m	-6.16	0.038791316	*RING/U-box superfamily protein*	Red ontogenesis	
ppa009439m	-6.16	0.000101984	*myb domain protein 7*	Red ontogenesis	
ppa025588m	-6.18	0.012673819		Red ontogenesis	
ppa015959m	-6.18	0.000117203	*Calcium-binding EF-hand family protein*	Red ontogenesis	
ppa022308m	-6.19	0.001656553	*Plant invertase/pectin methylesterase inhibitor superfamily*	Red ontogenesis	
ppa007112m	-6.20	0.000860438	*Pectin lyase-like superfamily protein*	Red ontogenesis	
ppa002363m	-6.20	4.88E-06		Red ontogenesis	
ppa025020m	-6.21	0.000290137	*O-acyltransferase (WSD1-like) family protein*	Red ontogenesis	
ppa023320m	-6.22	0.043378116		Red ontogenesis	
ppa1027118m	-6.23	0.016323512	*Ankyrin repeat family protein*	Red ontogenesis	
ppa002187m	-6.24	0.000133454	*heat shock protein 90.1*	Red ontogenesis	
ppa020970m	-6.28	0.000108352	*Peroxidase superfamily protein*	Red ontogenesis	
ppa006758m	-6.31	1.42E-05	*3-ketoacyl-CoA synthase 6*	Red ontogenesis	
ppa024725m	-6.32	0.000150593	*cellulose synthase family protein*	Red ontogenesis	
ppa007640m	-6.33	0.000103576	*Nucleotide-diphospho-sugar transferases superfamily protein*	Red ontogenesis	
ppa003926m	-6.34	0.000129544	*SKU5 similar 17*	Red ontogenesis	
ppa013833m	-6.37	0.002646757	*Bifunctional inhibitor/lipid-transfer protein/seed storage 2S albumi*	Red ontogenesis	
ppa019700m	-6.37	0.000244795	*Aldolase-type TIM barrel family protein*	Red ontogenesis	
ppa020044m	-6.38	0.00020087		Red ontogenesis	
ppa017323m	-6.40	1.42E-05	*Cupredoxin superfamily protein*	Red ontogenesis	
ppa017446m	-6.40	7.30E-05	*pinoresinol reductase 1*	Red ontogenesis	Secondary Metabolism / Flavonols-Isoflavonoid -Biotic Stress / Secondary metabolism involved into stress
ppa015351m	-6.42	0.020643701	*Late embryogenesis abundant (LEA) hydroxyproline-rich glycopro*	Red ontogenesis	
ppa019384m	-6.44	0.000180847	*germin-like protein 10*	Red ontogenesis	
ppa002007m	-6.44	7.33E-06		Red ontogenesis	
ppa017021m	-6.45	0.021796985	*Protein kinase protein with adenine nucleotide alpha hydrolases-l*	Red ontogenesis	
ppa001579m	-6.45	5.22E-06		Red ontogenesis	
ppa022341m	-6.45	0.049054557	*basic helix-loop-helix (bHLH) DNA-binding superfamily protein*	Red ontogenesis	
ppa010275m	-6.47	0.001275388	*HAD superfamily, subfamily IIIB acid phosphatase*	Red ontogenesis	
ppa006041m	-6.49	9.39E-05	*basic helix-loop-helix (bHLH) DNA-binding superfamily protein*	Red ontogenesis	
ppa012022m	-6.49	9.81E-06	*glycosylphosphatidylinositol-anchored lipid protein transfer 1*	Red ontogenesis	
ppa019092m	-6.49	6.69E-06	*GDSL-like Lipase/Acylhydrolase superfamily protein*	Red ontogenesis	
ppa005774m	-6.50	2.37E-06	*HXXXD-type acyl-transferase family protein*	Red ontogenesis	Secondary Metabolism / Phenylpropanoids - Biotic Stress / Secondary metabolism involved into stress
ppa025377m	-6.52	0.000214978		Red ontogenesis	
ppa017853m	-6.52	0.000138051	*Leucine-rich repeat protein kinase family protein*	Red ontogenesis	
ppa023908m	-6.53	0.000121115	*Integrase-type DNA-binding superfamily protein*	Red ontogenesis	
ppb010594m	-6.54	0.040378129	*cinnamyl alcohol dehydrogenase 9*	Red ontogenesis	Secondary Metabolism / Phenylpropanoids - Biotic Stress / Secondary metabolism involved into stress
ppa001363m	-6.54	3.78E-05	*beta-galactosidase 3*	Red ontogenesis	
ppa013630m	-6.60	0.033745207		Red ontogenesis	
ppa011821m	-6.61	0.01635446	*indole-3-acetic acid inducible 31*	Red ontogenesis	
ppb013496m	-6.61	0.000421489	*plasma membrane intrinsic protein 1A*	Red ontogenesis	
ppa023317m	-6.62	0.002902581	*Protein kinase protein with adenine nucleotide alpha hydrolases-l*	Red ontogenesis	
ppa024287m	-6.64	0.001907521	*CYCLIN D1;1*	Red ontogenesis	
ppa003171m	-6.64	4.95E-05		Red ontogenesis	
ppa003831m	-6.65	4.11E-06	*beta glucosidase 15*	Red ontogenesis	
ppb023573m	-6.66	0.010644561	*Ankyrin repeat family protein*	Red ontogenesis	
ppa021173m	-6.66	0.023343952	*Heavy metal transport/detoxification superfamily protein*	Red ontogenesis	
ppa018761m	-6.67	0.0188712	*alpha 1,4-glycosyltransferase family protein*	Red ontogenesis	
ppa005040m	-6.68	0.000373158	*Eukaryotic aspartyl protease family protein*	Red ontogenesis	
ppa026139m	-6.70	1.31E-05	*Integrase-type DNA-binding superfamily protein*	Red ontogenesis	
ppa026663m	-6.71	0.000266296	*actin-related protein C2B*	Red ontogenesis	
ppa023047m	-6.71	0.022364537	*vacuolar ATP synthase G3*	Red ontogenesis	
ppa012538m	-6.74	9.11E-07	*HSP20-like chaperones superfamily protein*	Red ontogenesis	
ppa007085m	-6.74	0.000193898	*PATATIN-like protein 9*	Red ontogenesis	
ppa025334m	-6.75	1.64E-05	*Transmembrane amino acid transporter family protein*	Red ontogenesis	
ppa024319m	-6.75	0.016692076		Red ontogenesis	
ppa023420m	-6.76	0.014730449		Red ontogenesis	
ppa021817m	-6.79	2.65E-05	*Protein phosphatase 2A regulatory B subunit family protein*	Red ontogenesis	
ppb019226m	-6.79	0.000468551	*Plant invertase/pectin methylesterase inhibitor superfamily protei*	Red ontogenesis	
ppa010152m	-6.82	8.59E-05	*HAD superfamily, subfamily IIIB acid phosphatase*	Red ontogenesis	
ppa024121m	-6.83	0.000977909	*Nucleotide excision repair, TFIIH, subunit TTDA*	Red ontogenesis	
ppa009020m	-6.83	0.000943058	*DNA glycosylase superfamily protein*	Red ontogenesis	
ppa005388m	-6.84	8.24E-05	*alpha/beta-Hydrolases superfamily protein*	Red ontogenesis	
ppa019861m	-6.85	0.004322316	*P-loop containing nucleoside triphosphate hydrolases superfamily*	Red ontogenesis	
ppa014138m	-6.89	7.47E-07		Red ontogenesis	
ppa020996m	-6.91	0.000229551	*Fatty acid hydroxylase superfamily*	Red ontogenesis	Biotic Stress / Secondary metabolism involved into stress
ppa025821m	-6.91	0.009699853	*HSP20-like chaperones superfamily protein*	Red ontogenesis	
ppb013718m	-6.91	0.042826124	*paralog of ARC6*	Red ontogenesis	
ppa020124m	-6.92	0.009561327	*AP2/B3-like transcriptional factor family protein*	Red ontogenesis	
ppa023379m	-6.93	0.000172084	*FASCICLIN-like arabinogalactan-protein 12*	Red ontogenesis	
ppa017398m	-6.93	0.000314402	*ERECTA-like 1*	Red ontogenesis	
ppa008709m	-6.95	6.26E-05	*NAD(P)-binding Rossmann-fold superfamily protein*	Red ontogenesis	
ppa010050m	-6.95	0.000171336	*ovate family protein 13*	Red ontogenesis	
ppa015232m	-6.96	0.000121081		Red ontogenesis	
ppa022307m	-6.97	1.14E-05	*HXXXD-type acyl-transferase family protein*	Red ontogenesis	
ppa021240m	-6.98	3.32E-05	*xylem serine peptidase 1*	Red ontogenesis	
ppa014598m	-6.98	0.000140244		Red ontogenesis	
ppa010951m	-6.98	0.000235348	*WUSCHEL related homeobox 4*	Red ontogenesis	
ppa019552m	-6.99	0.006166988	*basic helix-loop-helix (bHLH) DNA-binding superfamily protein*	Red ontogenesis	
ppa006588m	-7.00	0.000119475	*Core-2/I-branching beta-1,6-N-acetylglucosaminyltransferase fam*	Red ontogenesis	
ppa022906m	-7.02	0.005435643	*Ankyrin-repeat containing protein*	Red ontogenesis	
ppa010123m	-7.06	0.003223492	*Protein of unknown function (DUF1218)*	Red ontogenesis	
ppa011656m	-7.06	1.49E-06	*HSP20-like chaperones superfamily protein*	Red ontogenesis	
ppa000546m	-7.06	8.67E-05	*Myosin heavy chain-related protein*	Red ontogenesis	
ppa014086m	-7.07	0.000110442	*Gibberellin-regulated family protein*	Red ontogenesis	
ppa027143m	-7.08	0.000186932	*subtilase family protein*	Red ontogenesis	
ppa024832m	-7.09	0.001732375	*Ankyrin repeat family protein*	Red ontogenesis	
ppa019556m	-7.09	0.008172409	*Bifunctional inhibitor/lipid-transfer protein/seed storage 2S albumi*	Red ontogenesis	
ppa011385m	-7.10	3.40E-05	*germin 3*	Red ontogenesis	
ppa023313m	-7.11	0.003451339	*hydroxyproline-rich glycoprotein family protein*	Red ontogenesis	
ppa007950m	-7.13	0.000242724	*S-adenosyl-L-methionine-dependent methyltransferases superfar*	Red ontogenesis	
ppa019083m	-7.14	9.90E-05	*alpha 1,4-glycosyltransferase family protein*	Red ontogenesis	
ppa026294m	-7.15	0.001873483	*GDSL-like Lipase/Acylhydrolase family protein*	Red ontogenesis	
ppa003891m	-7.16	9.73E-06	*beta glucosidase 13*	Red ontogenesis	
ppa011846m	-7.18	0.000145905	*Disease resistance-responsive (dirigent-like protein) family protei*	Red ontogenesis	Biotic Stress / PR-proteins
ppa015782m	-7.19	0.013268004		Red ontogenesis	
ppa002834m	-7.23	4.13E-05	*Plant protein of unknown function (DUF828)*	Red ontogenesis	
ppa005373m	-7.24	7.80E-05	*Flavin-binding monooxygenase family protein*	Red ontogenesis	
ppa017396m	-7.25	0.022899133		Red ontogenesis	
ppa010762m	-7.28	7.45E-06	*heat shock protein 21*	Red ontogenesis	
ppa023210m	-7.29	0.004878629		Red ontogenesis	
ppa022667m	-7.32	0.000144806	*IQ calmodulin-binding motif family protein*	Red ontogenesis	
ppa016292m	-7.33	0.00086837	*terpene synthase 03*	Red ontogenesis	Biotic Stress / Secondary metabolism involved into stress
ppa019309m	-7.34	0.00318059	*GDSL-like Lipase/Acylhydrolase superfamily protein*	Red ontogenesis	
ppa023259m	-7.36	0.005900417		Red ontogenesis	
ppa022385m	-7.41	0.000125425	*Basic-leucine zipper (bZIP) transcription factor family protein*	Red ontogenesis	
ppa003422m	-7.42	4.48E-05	*Glucose-methanol-choline (GMC) oxidoreductase family protein*	Red ontogenesis	
ppa011467m	-7.44	1.91E-06	*germin 3*	Red ontogenesis	

ppa009543m	-7.48	0.002638854	*BIG PETAL P*	Red ontogenesis	
ppa001123m	-7.50	0.000192804	*Leucine-rich repeat protein kinase family protein*	Red ontogenesis	
ppa022739m	-7.50	2.60E-05	*squamosa promoter binding protein-like 5*	Red ontogenesis	
ppa025470m	-7.50	0.003849406	*HXXXD-type acyl-transferase family protein*	Red ontogenesis	Secondary Metabolism / Phenylpropanoids - Biotic Stress / Secondary metabolism involved into stress
ppa005104m	-7.50	0.000245443	*Eukaryotic aspartyl protease family protein*	Red ontogenesis	
ppa023338m	-7.50	0.003089308	*Cupredoxin superfamily protein*	Red ontogenesis	
ppa012613m	-7.50	0.002448636	*Bifunctional inhibitor/lipid-transfer protein/seed storage 2S albumi*	Red ontogenesis	
ppa007654m	-7.52	1.76E-06	*peroxidase 2*	Red ontogenesis	
ppa008634m	-7.54	0.001966943	*Peroxidase family protein*	Red ontogenesis	
ppa020401m	-7.55	0.008483608	*hydroxyproline-rich glycoprotein family protein*	Red ontogenesis	
ppa020509m	-7.55	0.002962762	*RNA polymerase II, Rpb4, core protein*	Red ontogenesis	
ppa010133m	-7.55	9.45E-05	*NAD(P)-binding Rossmann-fold superfamily protein*	Red ontogenesis	Secondary Metabolism / Dihydroflavonols -Biotic Stress / Secondary metabolism involved into stress
ppa023532m	-7.56	0.003817352	*terpene synthase-like sequence-1,8-cineole*	Red ontogenesis	Biotic Stress / Secondary metabolism involved into stress
ppa018616m	-7.56	0.00177127	*GDSL-like Lipase/Acylhydrolase superfamily protein*	Red ontogenesis	
ppa007495m	-7.58	0.005107005	*S-adenosyl-L-methionine-dependent methyltransferases superfa*	Red ontogenesis	
ppa011533m	-7.58	1.12E-06	*germin-like protein 7*	Red ontogenesis	
ppa012737m	-7.59	0.003277709	*Ran BP2/NZF zinc finger-like superfamily protein*	Red ontogenesis	
ppa013554m	-7.61	0.000343195	*lipid transfer protein 3*	Red ontogenesis	
ppa012795m	-7.61	9.81E-06	*HSP20-like chaperones superfamily protein*	Red ontogenesis	
ppa006195m	-7.62	0.0065365	*purple acid phosphatase 15*	Red ontogenesis	
ppa023616m	-7.62	0.000125277	*Gibberellin-regulated family protein*	Red ontogenesis	
ppa005430m	-7.63	2.69E-05	*COBRA-like extracellular glycosyl-phosphatidyl inositol-anchored*	Red ontogenesis	
ppb006257m	-7.64	0.005953646	*Li-tolerant lipase 1*	Red ontogenesis	
ppa005849m	-7.66	1.91E-06	*Glycosyl hydrolase superfamily protein*	Red ontogenesis	
ppa023110m	-7.67	0.000405104	*lipid transfer protein 1*	Red ontogenesis	
ppa026133m	-7.68	0.008197816	*Arabidopsis protein of unknown function (DUF241)*	Red ontogenesis	
ppa004498m	-7.68	8.59E-07	*glycerol-3-phosphate acyltransferase 6*	Red ontogenesis	
ppa013113m	-7.69	1.01E-05	*Nuclear transport factor 2 (NTF2) family protein*	Red ontogenesis	
ppa020548m	-7.73	0.000136999		Red ontogenesis	
ppa008177m	-7.74	0.000406029	*Nucleotide/sugar transporter family protein*	Red ontogenesis	
ppa003856m	-7.75	0.000500088	*beta glucosidase 16*	Red ontogenesis	
ppa010306m	-7.77	4.55E-05	*Immunoglobulin E-set superfamily protein*	Red ontogenesis	
ppa026213m	-7.78	0.00594636	*Ankyrin repeat family protein*	Red ontogenesis	
ppa005699m	-7.78	8.27E-05	*BURP domain-containing protein*	Red ontogenesis	
ppa007492m	-7.79	0.004789108	*S-adenosyl-L-methionine-dependent methyltransferases superfa*	Red ontogenesis	
ppa023525m	-7.81	0.022727754	*Plant protein of unknown function (DUF247)*	Red ontogenesis	
ppa022172m	-7.83	1.74E-05	*Protein kinase superfamily protein*	Red ontogenesis	
ppa021795m	-7.84	0.001199558		Red ontogenesis	
ppa004880m	-7.84	0.005641238	*glyoxal oxidase-related protein*	Red ontogenesis	
ppa022279m	-7.86	0.005288268	*S-adenosyl-L-methionine-dependent methyltransferases superfa*	Red ontogenesis	
ppa004644m	-7.86	0.00017465	*glycosyl hydrolase 9B1*	Red ontogenesis	
ppa023678m	-7.87	0.000161071		Red ontogenesis	
ppa012403m	-7.87	0.00030679	*alpha/beta-Hydrolases superfamily protein*	Red ontogenesis	
ppa003296m	-7.92	0.003131886	*laccase 2*	Red ontogenesis	Biotic Stress / Secondary metabolism involved into stress
ppa011450m	-7.92	0.0002111		Red ontogenesis	
ppa004910m	-7.95	0.005106497	*cytochrome P450, family 88, subfamily A, polypeptide 3*	Red ontogenesis	
ppa002824m	-7.99	0.00044913	*Fatty acid hydroxylase superfamily*	Red ontogenesis	Biotic Stress / Secondary metabolism involved into stress
ppa025683m	-8.01	0.002808515	*S-adenosyl-L-methionine-dependent methyltransferases superfa*	Red ontogenesis	
ppa008464m	-8.05	0.000190566	*Pollen Ole e 1 allergen and extensin family protein*	Red ontogenesis	
ppa011144m	-8.05	7.81E-06	*RmlC-like cupins superfamily protein*	Red ontogenesis	
ppa024025m	-8.06	0.00294754	*terpene synthase-like sequence-1,8-cineole*	Red ontogenesis	Biotic Stress / Secondary metabolism involved into stress
ppa006359m	-8.14	0.006026341	*Plant protein of unknown function (DUF828)*	Red ontogenesis	
ppa015037m	-8.16	8.90E-06	*Glycosyl hydrolase family protein*	Red ontogenesis	
ppa003165m	-8.17	3.00E-05	*plant glycogenin-like starch initiation protein 3*	Red ontogenesis	
ppa006506m	-8.18	0.003067948	*Calcium-dependent phosphotriesterase superfamily protein*	Red ontogenesis	Biotic Stress / Secondary metabolism involved into stress
ppa003572m	-8.20	2.69E-05	*laccase 11*	Red ontogenesis	Biotic Stress / Secondary metabolism involved into stress
ppa004037m	-8.21	8.18E-06	*Nucleotide-diphospho-sugar transferases superfamily protein*	Red ontogenesis	
ppb022512m	-8.21	0.003964209	*Thioredoxin superfamily protein*	Red ontogenesis	
ppa015992m	-8.25	0.000583902	*Protein kinase superfamily protein*	Red ontogenesis	
ppa023537m	-8.27	3.09E-05	*alpha-L-fucosidase 1*	Red ontogenesis	
ppa012890m	-8.27	1.42E-05	*Polyketide cyclase/dehydrase and lipid transport superfamily prot*	Red ontogenesis	
ppa015008m	-8.28	0.001793659	*Cystathionine beta-synthase (CBS) family protein*	Red ontogenesis	
ppa026358m	-8.29	1.32E-06	*beta glucosidase 15*	Red ontogenesis	
ppa007650m	-8.30	4.60E-06	*GDSL-like Lipase/Acylhydrolase superfamily protein*	Red ontogenesis	
ppa007123m	-8.32	0.002669471	*nodulin MtN21 /EamA-like transporter family protein*	Red ontogenesis	
ppa015046m	-8.34	0.002607975	*CAP (Cysteine-rich secretory proteins, Antigen 5, and Pathogene*	Red ontogenesis	
ppa016923m	-8.35	0.000920852	*myb domain protein 83*	Red ontogenesis	
ppa012870m	-8.36	0.002193633	*MLP-like protein 31*	Red ontogenesis	
ppa019658m	-8.36	0.000458462	*Disease resistance-responsive (dirigent-like protein) family protei*	Red ontogenesis	Biotic Stress / PR-proteins
ppa003308m	-8.37	0.001536652	*laccase 17*	Red ontogenesis	Biotic Stress / Secondary metabolism involved into stress
ppa015783m	-8.38	6.95E-06		Red ontogenesis	
ppa020420m	-8.39	0.006163435	*Pyridoxal phosphate (PLP)-dependent transferases superfamily p*	Red ontogenesis	Biotic Stress / Secondary metabolism involved into stress
ppa022641m	-8.42	0.000892712	*hydroxysteroid dehydrogenase 7*	Red ontogenesis	
ppa018980m	-8.42	4.37E-06	*RmlC-like cupins superfamily protein*	Red ontogenesis	
ppa015890m	-8.46	0.00317519	*D-arabinono-1,4-lactone oxidase family protein*	Red ontogenesis	
ppa010517m	-8.48	0.000285251	*TRICHOME BIREFRINGENCE-LIKE 43*	Red ontogenesis	
ppa017473m	-8.53	2.12E-05	*Bifunctional inhibitor/lipid-transfer protein/seed storage 2S albumi*	Red ontogenesis	
ppa021579m	-8.57	0.000387409	*fatty acid amide hydrolase*	Red ontogenesis	
ppa026308m	-8.58	0.000158832	*O-fucosyltransferase family protein*	Red ontogenesis	
ppa021829m	-8.58	0.000512269	*PEBP (phosphatidylethanolamine-binding protein) family protein*	Red ontogenesis	
ppa025815m	-8.63	0.00136682	*Pyridoxal phosphate (PLP)-dependent transferases superfamily p*	Red ontogenesis	Biotic Stress / Secondary metabolism involved into stress
ppa006799m	-8.63	0.000552781	*Cytochrome b561/ferric reductase transmembrane with DOMON*	Red ontogenesis	
ppa018413m	-8.66	0.000149365	*Pyridoxal phosphate (PLP)-dependent transferases superfamily p*	Red ontogenesis	Biotic Stress / Secondary metabolism involved into stress
ppa002391m	-8.67	0.000723919		Red ontogenesis	
ppa023341m	-8.71	0.004920499	*terpene synthase 03*	Red ontogenesis	Biotic Stress / Secondary metabolism involved into stress
ppa022423m	-8.73	1.10E-05		Red ontogenesis	
ppa006117m	-8.73	7.20E-06	*HXXXD-type acyl-transferase family protein*	Red ontogenesis	
ppa015904m	-8.76	0.000131418	*nodulin MtN21 /EamA-like transporter family protein*	Red ontogenesis	
ppa003816m	-8.80	0.000137666	*NRAMP metal ion transporter 6*	Red ontogenesis	
ppa026712m	-8.91	5.52E-06	*beta glucosidase 31*	Red ontogenesis	
ppa007610m	-8.96	2.74E-05	*Protein of Unknown Function (DUF239)*	Red ontogenesis	
ppa004968m	-8.96	1.91E-05	*UDP-glucosyl transferase 85A2*	Red ontogenesis	
ppa004817m	-8.96	7.26E-07	*Jojoba acyl CoA reductase-related male sterility protein*	Red ontogenesis	
ppa012775m	-8.99	0.000116331		Red ontogenesis	
ppa024899m	-9.06	1.68E-05	*Gibberellin-regulated family protein*	Red ontogenesis	
ppa005535m	-9.11	1.31E-06	*BURP domain-containing protein*	Red ontogenesis	
ppa020184m	-9.22	0.000121115	*Aldolase-type TIM barrel family protein*	Red ontogenesis	
ppa007652m	-9.25	7.38E-05	*GDSL-like Lipase/Acylhydrolase superfamily protein*	Red ontogenesis	
ppa009531m	-9.32	3.78E-05	*GDSL-like Lipase/Acylhydrolase superfamily protein*	Red ontogenesis	
ppa003319m	-9.33	3.28E-05	*laccase 17*	Red ontogenesis	Biotic Stress / Secondary metabolism involved into stress
ppa009528m	-9.35	4.62E-07	*GDSL-like Lipase/Acylhydrolase superfamily protein*	Red ontogenesis	
ppa002624m	-9.35	0.000193898		Red ontogenesis	
ppa016893m	-9.40	0.000736777	*phosphate transporter 4;6*	Red ontogenesis	
ppa001656m	-9.41	0.000102964	*beta-xylosidase 2*	Red ontogenesis	
ppa018933m	-9.41	8.92E-05	*beta glucosidase 13*	Red ontogenesis	
ppa004011m	-9.44	1.93E-06	*3-ketoacyl-CoA synthase 10*	Red ontogenesis	
ppa012742m	-9.46	5.10E-06	*MLP-like protein 28*	Red ontogenesis	
ppa015128m	-9.47	8.59E-07	*O-fucosyltransferase family protein*	Red ontogenesis	
ppa009979m	-9.50	0.000872001	*GNS1/SUR4 membrane protein family*	Red ontogenesis	
ppa006707m	-9.58	0.000112134	*Auxin-responsive family protein*	Red ontogenesis	
ppa023240m	-9.61	1.04E-05	*MLP-like protein 28*	Red ontogenesis	
ppa017225m	-9.62	0.000316334		Red ontogenesis	
ppa010149m	-9.69	9.67E-05	*Arabidopsis protein of unknown function (DUF241)*	Red ontogenesis	
ppa021389m	-9.71	0.000877414	*Fatty acid hydroxylase superfamily*	Red ontogenesis	Biotic Stress / Secondary metabolism involved into stress
ppa015159m	-9.78	1.97E-06	*Plant basic secretory protein (BSP) family protein*	Red ontogenesis	
ppa010302m	-9.83	8.34E-07	*carbonic anhydrase 2*	Red ontogenesis	
ppa015166m	-9.89	0.000133204	*L-O-methylthreonine resistant 1*	Red ontogenesis	
ppa014775m	-10.12	2.79E-06	*Leucine-rich repeat (LRR) family protein*	Red ontogenesis	
ppa025024m	-10.17	0.000131418	*Bifunctional inhibitor/lipid-transfer protein/seed storage 2S albumi*	Red ontogenesis	
ppa019518m	-10.25	5.33E-05	*beta glucosidase 15*	Red ontogenesis	
ppa003646m	-10.33	2.86E-05	*Laccase/Diphenol oxidase family protein*	Red ontogenesis	Biotic Stress / Secondary metabolism involved into stress
ppa011103m	-10.33	6.54E-06	*heat shock protein 21*	Red ontogenesis	
ppa019259m	-10.34	0.000232561		Red ontogenesis	
ppa007395m	-10.35	6.83E-07	*proline-rich protein 2*	Red ontogenesis	
ppa005351m	-10.37	6.87E-07	*Pollen Ole e 1 allergen and extensin family protein*	Red ontogenesis	
ppa007768m	-10.39	3.15E-05	*ARIA-interacting double AP2 domain protein*	Red ontogenesis	
ppa007953m	-10.43	9.74E-05	*GDSL-like Lipase/Acylhydrolase superfamily protein*	Red ontogenesis	
ppa007004m	-10.45	3.65E-05	*beta-hydroxyisobutyryl-CoA hydrolase 1*	Red ontogenesis	
ppa026448m	-10.47	0.000156024	*beta glucosidase 17*	Red ontogenesis	
ppa009061m	-10.53	0.000185002	*fatty acid desaturase 5*	Red ontogenesis	
ppa003501m	-10.56	2.36E-07	*heat-shock protein 70T-2*	Red ontogenesis	
ppa012312m	-10.61	6.87E-07	*Pollen Ole e 1 allergen and extensin family protein*	Red ontogenesis	
ppa007642m	-10.63	5.72E-05	*GDSL-like Lipase/Acylhydrolase superfamily protein*	Red ontogenesis	
ppa005391m	-10.84	4.79E-06	*Pectin lyase-like superfamily protein*	Red ontogenesis	
ppa007678m	-10.99	0.000103787	*cinnamyl alcohol dehydrogenase 9*	Red ontogenesis	Secondary Metabolism / Phenylpropanoids - Biotic Stress / Secondary metabolism involved into stress
ppa023999m	-11.00	2.13E-05	*FASCICLIN-like arabinogalactan-protein 11*	Red ontogenesis	
ppa019380m	-11.02	1.45E-05	*Duplicated homeodomain-like superfamily protein*	Red ontogenesis	
ppa009815m	-11.17	3.22E-05	*GDSL-like Lipase/Acylhydrolase superfamily protein*	Red ontogenesis	
ppa004078m	-11.26	8.53E-08	*cytochrome P450, family 86, subfamily A, polypeptide 8*	Red ontogenesis	
ppa012112m	-11.33	2.04E-05	*Pollen Ole e 1 allergen and extensin family protein*	Red ontogenesis	
ppa026621m	-11.47	1.42E-05		Red ontogenesis	
ppa012898m	-11.59	3.05E-06	*Polyketide cyclase/dehydrase and lipid transport superfamily prot*	Red ontogenesis	
ppa007991m	-11.77	7.26E-07	*GDSL-like Lipase/Acylhydrolase superfamily protein*	Red ontogenesis	
ppa019383m	-11.79	2.92E-05	*Pollen Ole e 1 allergen and extensin family protein*	Red ontogenesis	
ppa012828m	-11.80	8.37E-07	*Uncharacterised protein family (UPF0497)*	Red ontogenesis	
ppa013440m	-11.98	8.04E-06	*CAP (Cysteine-rich secretory proteins, Antigen 5, and Pathogene*	Red ontogenesis	
ppa010601m	-12.09	5.99E-05	*Integrase-type DNA-binding superfamily protein*	Red ontogenesis	
ppa010487m	-12.11	0.014356337		Red ontogenesis	

ppa017555m	-12.36	4.76E-05	*alpha 1,4-glycosyltransferase family protein*	Red ontogenesis	
ppa007427m	-13.81	2.84E-05	*GDSL-like Lipase/Acylhydrolase superfamily protein*	Red ontogenesis	
ppa018693m	-13.97	1.78E-06	*TRICHOME BIREFRINGENCE-LIKE 43*	Red ontogenesis	
ppa012809m	-14.92	2.36E-07	*MLP-like protein 423*	Red ontogenesis	
ppa007958m	-15.32	2.79E-07	*GDSL-like Lipase/Acylhydrolase superfamily protein*	Red ontogenesis	
